# Identification and validation of key modules and hub genes associated with the pathological stage of oral squamous cell carcinoma by weighted gene co-expression network analysis

**DOI:** 10.7717/peerj.8505

**Published:** 2020-02-04

**Authors:** Xuegang Hu, Guanwen Sun, Zhiqiang Shi, Hui Ni, Shan Jiang

**Affiliations:** 1 Department of Stomatology, Shenzhen Hospital, University of Chinese Academy of Sciences, Shenzhen, Guangdong, China; 2 Department of Endodontics and Operative Dentistry, School and Hospital of Stomatology, Fujian Medical University, Fuhou, Fujian, China; 3 Department of Stomatology, Ningbo Medical Center Lihuili Eastern Hospital, Ningbo, Zhejiang, China; 4 Restorative Dental Sciences, Faculty of Dentistry, The University of Hong Kong, Hong Kong SAR, China

**Keywords:** Oral squamous cell carcinoma (OSCC), Weighted gene co-expression network analysis (WGCNA), Hub gene, Pathologic stage, Overall survival

## Abstract

**Background:**

Oral squamous cell carcinoma (OSCC) is a major lethal malignant cancer of the head and neck region, yet its molecular mechanisms of tumourigenesis are still unclear.

**Patients and methods:**

We performed weighted gene co-expression network analysis (WGCNA) on RNA-sequencing data with clinical information obtained from The Cancer Genome Atlas (TCGA) database. The relationship between co-expression modules and clinical traits was investigated by Pearson correlation analysis. Furthermore, the prognostic value and expression level of the hub genes of these modules were validated based on data from the TCGA database and other independent datasets from the Gene Expression Omnibus (GEO) database and the Human Protein Atlas database. The significant modules and hub genes were also assessed by functional analysis and gene set enrichment analysis (GSEA).

**Results:**

We found that the turquoise module was strongly correlated with pathologic T stage and significantly enriched in critical functions and pathways related to tumourigenesis. PPP1R12B, CFD, CRYAB, FAM189A2 and ANGPTL1 were identified and statistically validated as hub genes in the turquoise module and were closely implicated in the prognosis of OSCC. GSEA indicated that five hub genes were significantly involved in many well-known cancer-related biological functions and signaling pathways.

**Conclusion:**

In brief, we systematically discovered a co-expressed turquoise module and five hub genes associated with the pathologic T stage for the first time, which provided further insight that WGCNA may reveal the molecular regulatory mechanism involved in the carcinogenesis and progression of OSCC. In addition, the five hub genes may be considered candidate prognostic biomarkers and potential therapeutic targets for the precise early diagnosis, clinical treatment and prognosis of OSCC in the future.

## Introduction

Oral squamous cell carcinoma (OSCC) is the most common malignancy of head and neck squamous cell carcinoma (HNSCC) and has poor prognosis and survival (Bozec et al., 2009; Ferlay et al., 2015). The main treatment for OSCC is comprehensive treatment based on surgical operation, which prolongs the survival time of patients and improves the quality of life (Kim et al., 2017; Verusingam et al., 2017). However, due to the lack of early diagnostic markers, patients are often in an advanced clinical stage at the time of diagnosis, and the 5-year overall survival of patients with OSCC remains low (Bland, Clarke & Harden, 1976; Omar, 2013). Therefore, effective biomarkers are needed to explore diagnostic and therapeutic targets for OSCC (Mehrotra & Gupta, 2011).

The occurrence and development of OSCC is an extremely complex progressive process involving multiple molecular mechanisms (Kamangar, Dores & Anderson, 2006). Due to the limitations of traditional studies, most previous studies focused on individual genes or pathways, and the relationship between genes was ignored (Sun et al., 2017). With the advent and rapid development of RNA sequencing technologies in various tumours, bioinformatics analysis has been widely and rapidly used to identify novel and more effective potential biomarkers for the diagnosis, therapy and prognosis of many diseases (Hinchcliff et al., 2019). For example, one study by Ahluwalia et al. (2019) identified a novel 4-gene prognostic signature that has clinical utility in colorectal cancer using The Cancer Genome Atlas (TCGA) database and Gene Expression Omnibus (GEO) database.

Weighted gene co-expression network analysis (WGCNA) is an efficient systematic biological approach that can highlight co-expressed gene modules and investigate the relationships between gene modules and phenotypes more effectively (Langfelder & Horvath, 2008). WGCNA has been successfully and comprehensively used to explore targeted modules and hub genes in cancer-related research, such as clear cell renal cell carcinoma (Wang et al., 2019a; Wang et al., 2019b) and pancreatic carcinoma (Zhou et al., 2018a; Zhou et al., 2018b).

In the current study, we used WGCNA and other bioinformatics analysis methods to explore RNA-Seq data and clinical phenotypes of OSCC patients. Ultimately, we identified that the turquoise module was significantly associated with pathologic T stage for the first time. Five hub genes (PPP1R12B, CFD, CRYAB, FAM189A2 and ANGPTL1) related to prognosis at the transcriptional level were identified and validated in other independent datasets. Further functional analysis indicated that these genes were significantly enriched in critical biological functions and pathways related to the tumourigenesis and development of OSCC.

## Materials & Methods

### Study design

**Figure 1:**
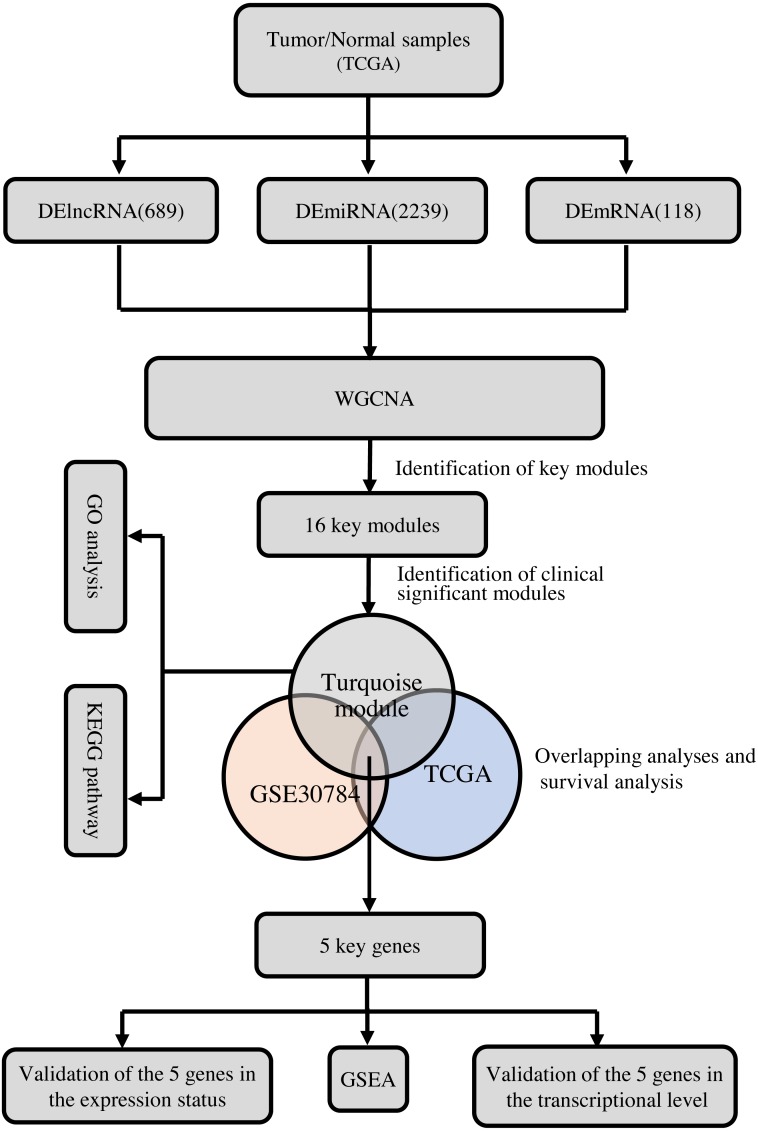
The flow chart of data preparation, processing, analysis, and validation.

To clarify the research process, the workflow of our study is presented in Fig. 1.

### Data acquisition

The RNA-seq expression data and relative clinical information of OSCC patients were retrieved from the TCGA database (https://portal.gdc.cancer.gov/) and GEO database (http://www.ncbi.nlm.nih.gov/geo/). A total of 373 patients were obtained from TCGA and used as a discovery group to construct a co-expression model of which 44 normal ones and 329 OSCC. Also, 229 patients (167 OSCC samples, 17 dysplasia samples and 45 normal samples) from GSE30784 had available data on clinical characteristics.

### Data preprocessing

Data preprocessing and analysis procedures were used to process the raw data, including robust multi-array average (RMA) background correction and the “affy” R package. The Affymetrix annotation files were used to annotate probes, and probes with no annotation were removed. False discovery rate (FDR) <0.05 and —log2FC— ≥ 2 were set as the cut-off values for screening differentially expressed mRNAs (DEmiRNA), lncRNAs (DElncRNA), and miRNA (DEmiRNA).

### Differential gene expression analysis

The “edger” R package was used to screen differentially expressed genes (DEGs) in the TCGA dataset between normal and OSCC samples and “limma” R package was used to screen DEGs from GSE30784 (Robinson, McCarthy & Smyth, 2010). The threshold was set as log2FC ≥2, and FDR <0.05 was considered significant (Lai, 2017; McCarthy, Chen & Smyth, 2012; Robinson, McCarthy & Smyth, 2010). DEGs that met this criteria were chosen for further analysis. The construction of a volcano plot and hierarchical clustering analysis were also performed by the R packages “ggplot2” and “pheatmap”, respectively.

### Weighted gene co-expression network construction (WGCNA)

The construction of scale-free gene co-expression modules and identification of highly correlated genes of the DEGs, including lncRNAs, miRNAs and mRNAs, were conducted by the “WGCNA” package in R software (http://www.r-project.org/) (Chen & Boutros, 2011; Zhang & Horvath, 2005). Similar expression modules can be demonstrated by genes that have the same pathway or function. The cut-off of the co-expression module was set as *P* < 0.05. Then, we further calculated and visualized the dissimilarity of module eigengenes (MEs), chose a cut line for the module dendrogram and merged some modules.

### Identification of clinically significant modules

Module-trait associations between MEs and clinical traits, including sex, age, grade, clinical stage (TNM) and pathological stage, were assessed by the Pearson test. In principal component analysis, MEs are considered the principal component of each gene module, and the expression patterns of all genes can be summarized as a single characteristic expression profile within a given module. The module with the absolute module significance (MS) ranked first among all the selected modules was considered to be related to a clinical trait (Shi et al., 2015). The module significantly correlated (*P* < 0.05) with the phenotype was selected for further investigation.

### Identifying hub genes and survival analysis

To identify overlapping genes among the significant modules and the TCGA and GEO (GSE30784) datasets, a Venn diagram (http://jvenn.toulouse.inra.fr/app/example.html) was constructed. The overlapping genes were chosen as the potential genes for overall survival analysis and validation, which were performed using the log-rank test (*p* < 0.05).

### Validation of the hub genes

The key genes overlapping among the significant modules and TCGA and GEO datasets that were also significant in survival analysis were chosen as the potential genes for further analysis and validation. *P*-values less than 0.05 were regarded as statistically significant. Furthermore, the Human Protein Atlas database (https://www.proteinatlas.org/) (Uhlén et al., 2015) was used to validate the protein expression level of the hub genes.

### Functional enrichment analysis of meaningful modules and key genes

To further explore the biological functions of the clinically significant modules and hub genes, we used the “clusterprofiler” package to perform Gene Ontology (GO) term analysis, Kyoto Encyclopedia of Genes and Genomes (KEGG) pathway enrichment analysis and gene set enrichment analysis (GSEA, https://software.broadinstitute.org/gsea/index.jsp). The enrichment analysis of biological functions and pathways can be described and visualized. The significance level was set as *p*-value <0.01 and FDR <0.05.

## Results

### Differentially expressed RNAs between OSCC and control samples

**Figure 2:**
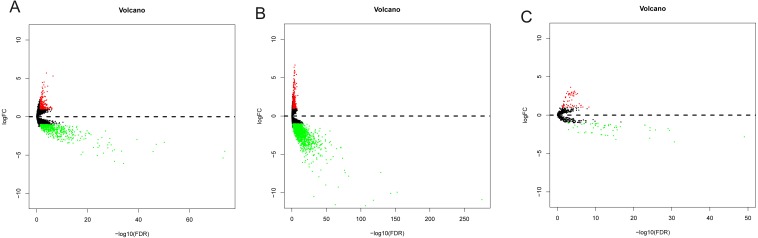
The volcano plot of differentially expressed RNAs in patients with OSCC. (A) DElncNAs; (B) DEmiRNAs; (C) DEmRNAs. Up-regulated RNA and down-regulated was represented in red dot and green dot respectively.

**Figure 3:**
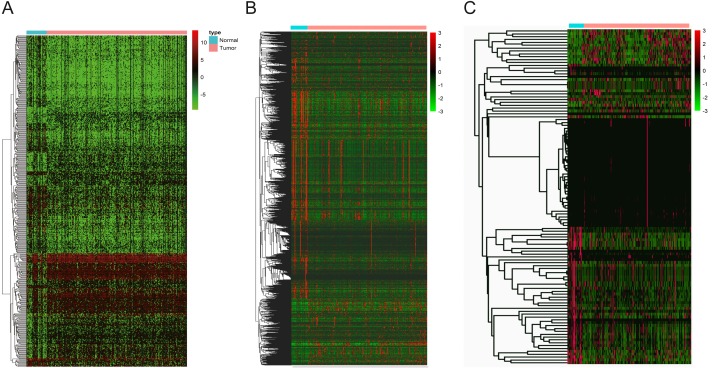
The heatmaps of DEGs between OSCC and normal tissues. (A) DElncNAs; (B) DEmiRNAs; (C) DEmRNAs. Each row represented a kind of RNA, and each column referred to one sample. Expression values are represented by the color scale. Redder grids are higher in expression values, while greener ones are lower.

Data from 44 normal and 329 OSCC patients were obtained from the TCGA database. The clinical characteristics of patients with OSCC from TCGA were showed in Table 1. Based on the differential analysis, including 689 lncRNAs, 2239 mRNAs and 118 miRNAs, were left after filtering with thresholds of —log2FC—>2 and adjusted *P* < 0.05 with edger R package (Robinson, McCarthy & Smyth, 2010). The volcano map and expression heat map of the differential RNAs were constructed to illustrate the distribution in each category and are presented in Figs. 2 and 3.

**Table 1:** Clinical characteristics of patients with OSCC from TCGA.

**Variables**	**Patients, n (%)**
Age, years	
≤50	52 (17.5)
>50	245 (82.5)
Gender	
Male	93(31.3)
Female	204(68.7)
Stage	
I	10 (3.4)
II	73 (24.6)
III	64 (21.5)
IV	150 (50.5)
Lymph Node	
N0	158 (53.2)
N1	56 (18.8)
N2	80 (26.9)
N3	3 (3.1)
T stage	
T1	19 (6.4)
T2	97 (32.6)
T3	75 (25.3)
T4	106 (35.7)
Metastasis	
Yes	295 (99.3)
No	2 (0.7)

**Notes.**

Abbreviations OSCC oral squamous cell carcinoma TCGA The Cancer Genome Atlas TNM tumor nodes metastasis

### Construction of the co-expression modules of OSCC

**Figure 4:**
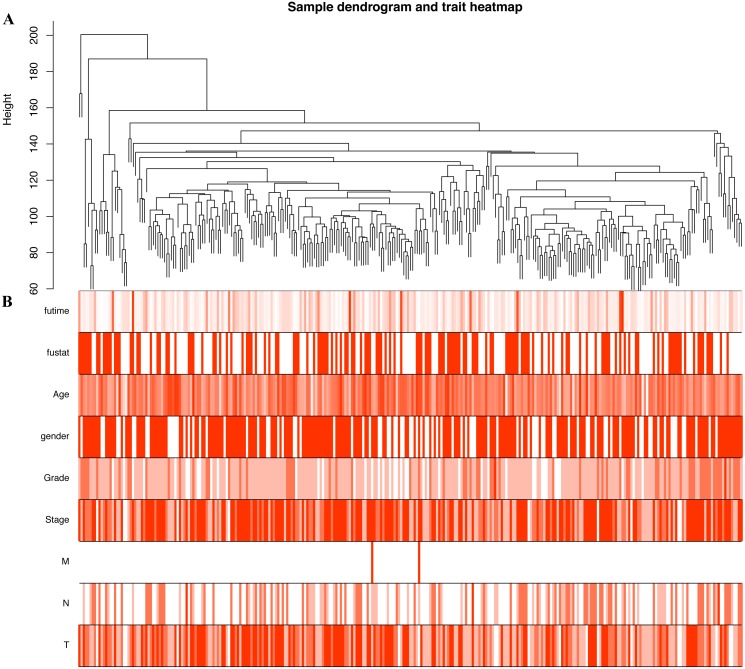
Construction of co-expression modules by the “WGCNA” package. (A) Sample clustering to detect outliers. (B) Sample cluster dendrogram and trait indicators. T, primary tumor; N, lymph node; M, distant metastasis.

**Figure 5:**
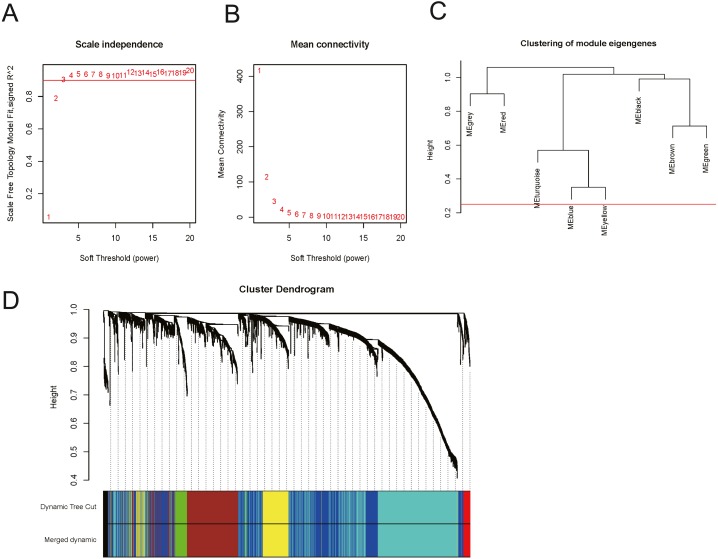
Construction of co-expression modules by the “WGCNA” package. (A) and (B) Analysis of network topology for various soft-thresholding powers. (A) The scale-free fit index as a function of the soft-thresholding power is shown. (B) The mean connectivity as a function of the soft-thresholding power is displayed. (C) The cluster dendrogram of module eigengenes. (D) The cluster dendrogram of genes in TCGA. Each branch in the figure represents one gene, and every colour below represents one co-expression module.

In order to explore the relationship between dysregulated RNAs and clinical parameters. The “WGCNA” package was used to construct co-expression networks and modules with differentially expressed RNAs based on OSCC from TCGA. Then, these samples were used for cluster analysis by the “flashClust tools” package, and the results are shown in Fig. 4. As shown in Figs. 5A and 5B, when the soft thresholding power value was chosen as 3 (*β* = 3), a hierarchical clustering tree (dendrogram) and consensus module eigengenes (Figs. 5C and 5D), including 8 merged co-expression modules, were produced.

### Gene Co-expression Modules Correspond to Clinical Traits

**Figure 6:**
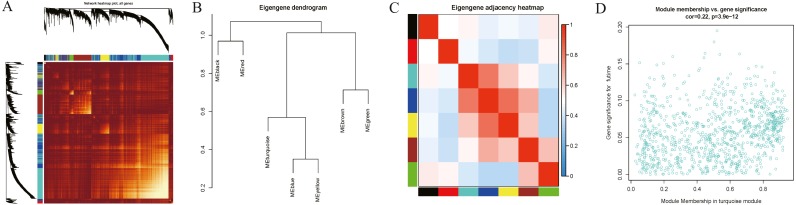
Functional gene modules detected by co-expression network analysis. (A) Topological overlap heatmap of the gene co-expression network. Each row and column represents a gene. Light color indicates low topological overlap, and on the contrary dark color denotes high topological overlap. The different side colors indicate different modules. The dendrogram suggests the clustering of these genes based on the similarity of their gene expression profiles. (B) and (C) The correlation between each module demonstrated based on eigengene. Blue represents a negative correlation, while red represents a positive correlation. (D) Scatter diagram of pathologic T stage vs. module membership for the significant genes in the turquoise module.

**Figure 7:**
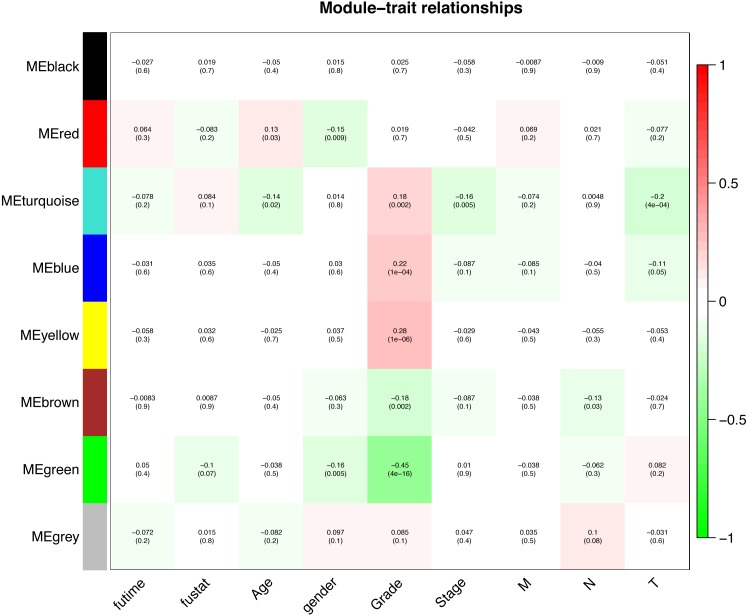
Module-trait associations were evaluated by correlations between MEs and clinical traits. Each row corresponds to a module eigengene, column to a trait. Each cell contains the corresponding correlation (first line) and *p*-value (second line). The cells are color coded by the correlation according to the color legend. Red module positively correlated to age (*p* < 0.05). Turquoise module negatively correlated to age (*p* < 0.05). Red and green modules negatively correlated to gender (*p* < 0.05). Turquoise, blue and yellow modules positively correlated to grade (*p* < 0.05). Green and brown modules negatively correlated to grade (*p* < 0.05). Turquoise module negatively correlated to stage (*p* < 0.05). Brown module negatively correlated to pathologic N stage (*p* < 0.05). Turquoise and blue modules negatively correlated to pathologic T stage (*p* < 0.05).

Interaction relationships of the 8 co-expression modules were identified and are shown in Fig. 6A, which revealed that each co-expression module independently validated each other in the network. As shown in Fig. 7, the module-feature relationship of the turquoise module revealed a highly negative correlation (*r* =  − 0.2, *P* = 4e−04) with pathologic T stage compared with other modules by Pearson’s correlation analysis. In addition, the eigengene dendrogram and heatmap were constructed to explore groups of correlated eigengenes and the dendrogram of all modules (Figs. 6B and 6C). The 7 modules were found to be mainly divided into two clusters. In addition, we constructed a scatterplot of pathologic T stage vs. module membership in the turquoise module, which illustrated that they were highly correlated (cor = 0.22, *p* = 3.9e−12) (Fig. 6D).

### Functional enrichment analysis of genes in the turquoise module

**Figure 8:**
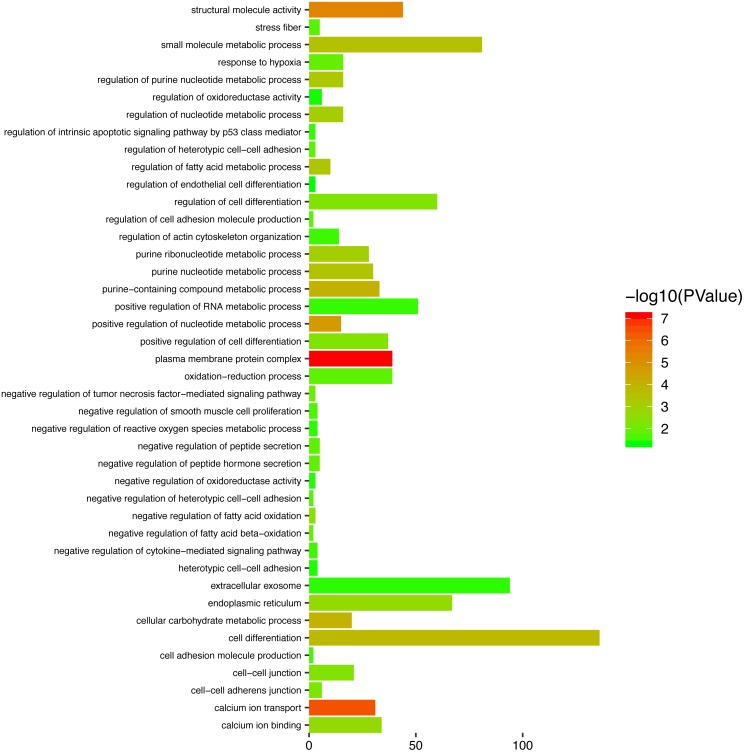
Top enriched GO terms for the turquoise module.

**Figure 9:**
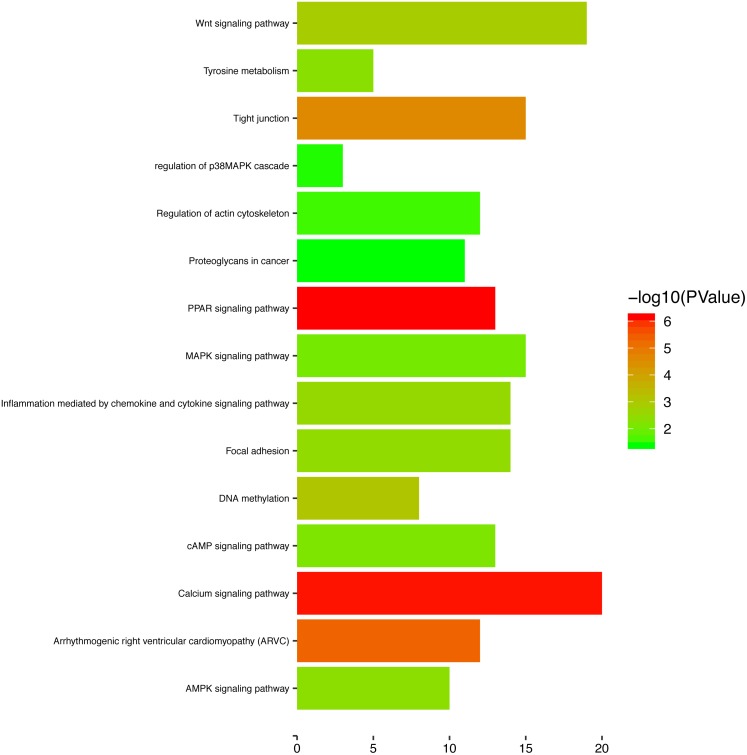
The enriched KEGG pathways for the turquoise module.

To gain a primary understanding of the biological functions and pathway relevance of the turquoise module, GO enrichment analysis and KEGG pathway enrichment analysis were conducted. The results of GO enrichment analysis showed that the turquoise module was significantly enriched in critical biological functions, such as cell differentiation, cell–cell adhesion, and cell–cell junctions (Fig. 8). KEGG enrichment analysis revealed that the genes are significantly involved in many pathways that are correlated with tumourigenesis, including the MAPK signalling pathway, intrinsic apoptotic signalling pathway, AMPK signalling pathway, Wnt signalling pathway and Calcium signalling pathway (Fig. 9).

### Identification of hub genes by overlapping analyses and survival analysis

**Figure 10:**
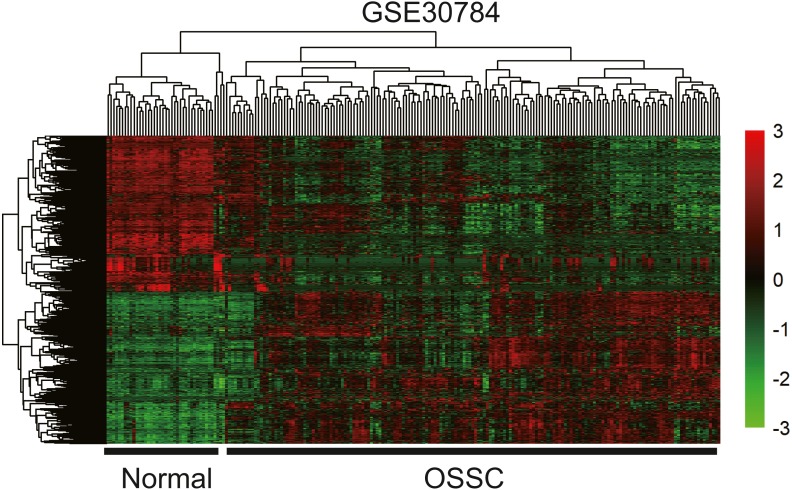
The hierarchical clustering heat map of differently expressed genes in the GEO database (GSE30784). (—log2FC—> 2 and *P* < 0.05). Significantly upregulated RNAs and downregulated RNAs are represented by red lines and green lines, respectively.

**Figure 11:**
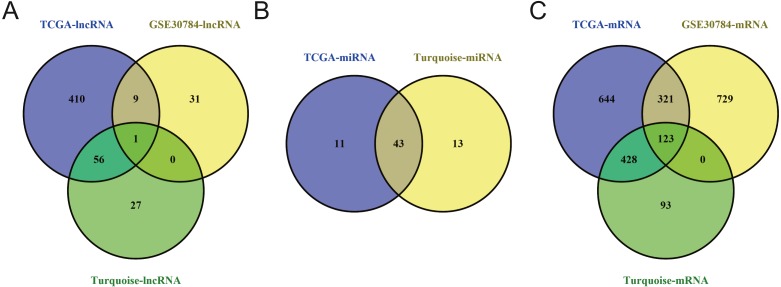
Venn diagram of overlapping genes among the turquoise module and TCGA and GEO (GSE30784) datasets. (A) lncRNA. (B) miRNA. (C) mRNA.

**Figure 12:**
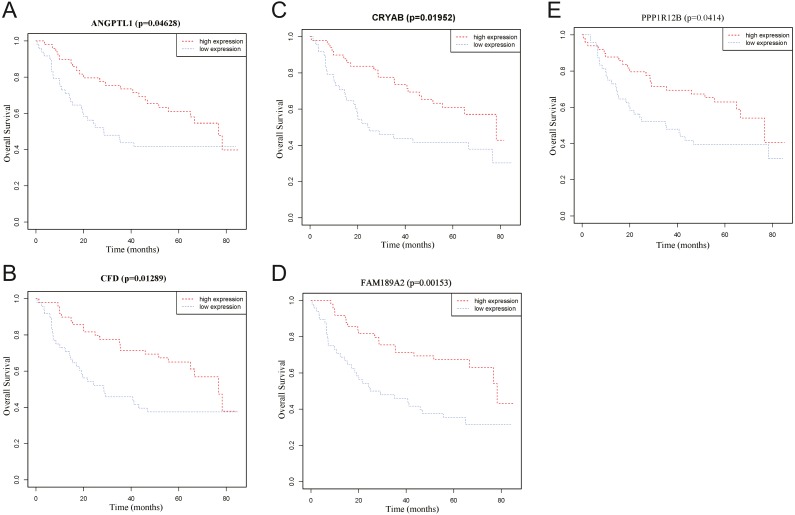
Overall survival analyses of hub genes in the turquoise module. (A) Overall survival analyses of ANGPTL1. (B) Overall survival analyses of CFD. (C) Overall survival analyses of CRYAB. (D) Overall survival analyses of FAM189A2. (E) Overall survival analyses of PPP1R12B. The red line represents samples with high gene expression, and the blue line represents samples with low gene expression.

To identify the hub genes of the turquoise module, overlapping analyses and survival analysis were performed. The differentially expressed genes in 212 patients (167 OSCC samples and 45 normal samples) were screened and extracted from the GEO database (GSE30784) with limma R package. The heat map of differential RNAs is shown in Fig. 10 which including xxx mRNAs and xxx lncRNAs. Then, a Venn diagram was constructed for overlapping analysis to identify overlapping genes among the turquoise module and the TCGA and GEO (GSE30784) databases. As shown in the Venn diagram, 1 lncRNA (Fig. 11A) and 123 mRNAs (Fig. 11C) were present in the turquoise module, the TCGA and GEO (GSE30784) datasets. 43 miRNAs were present in the turquoise module and the TCGA (Fig. 11B). Then, the overlapping genes including 1 lncRNA, 43 miRNAs and 123 mRNAs were selected as the potential genes by the log-rank test (*p* < 0.05) for further overall survival analysis. Eventually, 5 hub mRNAs (ANGPTL1) (Fig. 12A), CFD (Fig. 12B), CRYAB (Fig. 12C), FAM189A2 (Fig. 12D) and PPP1R12B (Fig. 12E) were identified. The Kaplan–Meier survival curve of the overall survival analysis revealed that OSCC patients with low expression levels of the 5 hub genes tended to have a poor outcome.

### Validation of the hub genes

**Figure 13:**
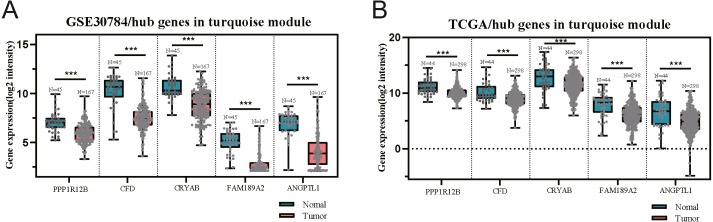
Validation of five hub genes between OSCC samples and normal tissue in the GSE30784 (A) and TCGA (B) datasets. The five hub genes were significantly downregulated in tumourtissues compared with normal samples.

**Figure 14:**
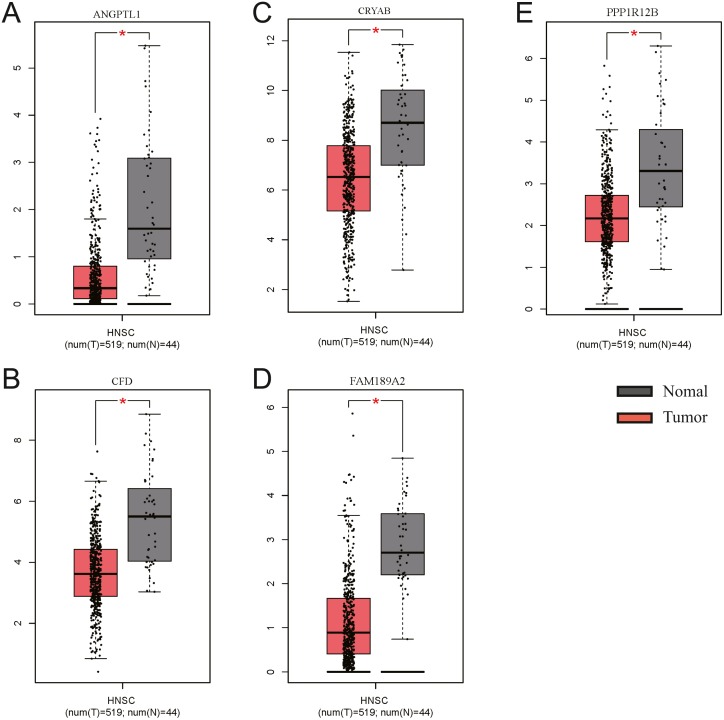
Validation of the gene expression of the five hub genes between OSCC samples and normal tissue from GEPIA. We obtained similar results as above. (A) The gene expression of ANGPTL1 between OSCC samples and normal tissue from GEPIA. (B) The gene expression of CFD between OSCC samples and normal tissue from GEPIA. (C) The gene expression of CRTAB between OSCC samples and normal tissue from GEPIA. (D) The gene expression of FAM189A2 between OSCC samples and normal tissue from GEPIA. (E) The gene expression of PPP1R12B between OSCC samples and normal tissue from GEPIA.

**Figure 15:**
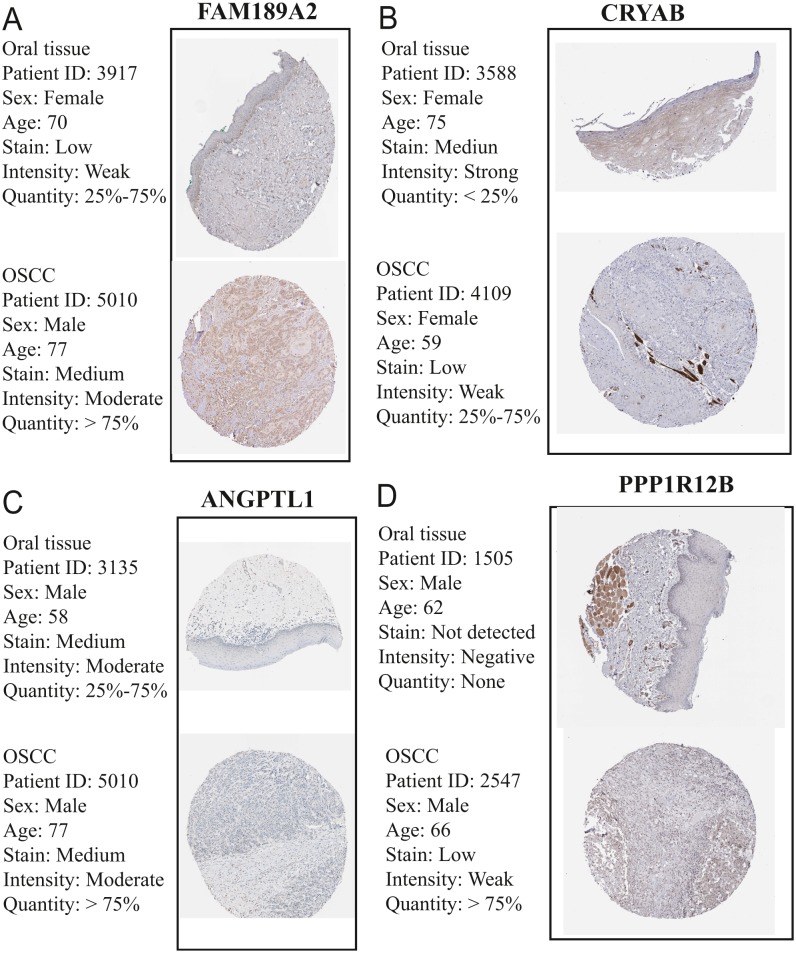
Validation of hub genes in the translational level. (A) Validation of FAM189A2 in turquoise module by The Human Protein Atlas database (IHC). (B) Validation of CRYAB in turquoise module by The Human Protein Atlas database (IHC). (C) Validation of ANGPTL1 in turquoise module by The Human Protein Atlas database (IHC). (D) Validation of PPP1R12B in turquoise module by The Human Protein Atlas database (IHC). There was no related IHC samples for CFD in The Human Protein Atlas database (IHC).

The GEO (GSE30784) and TCGA datasets were used to validate the expression status of the 5 hub genes (ANGPTL1, CFD, CRYAB, FAM189A2, and PPP1R12B). Compared with that in adjacent normal tissues, the 5 hub genes were significantly downregulated in tumour tissues from the GEO datasets (GSE30784) (Fig. 13A) and TCGA (Fig. 13B). This result mainly indicates that the expression status was consistent with the pathologic T stage, namely, that the expression of the 5 hub genes of the turquoise module was negatively correlated with the pathologic T stage. In addition, HNSCCs was analysed by the GEPIA online tool (519 OSCC samples and 44 normal samples) (http://gepia.cancer-pku.cn/) to validate the expression status of the 5 hub genes, which showed that the results were consistent with those described earlier, indicating that the results above are convincing and reliable (Figs. 14A–14E). Moreover, the protein levels of immunohistochemistry (IHC) staining obtained from the Human Protein Atlas (HPA) database showed that the expression of four of the hub genes (CRYAB, FAM189A2, ANGPTL1 and PPP1R12B) were significantly lower in tumour tissues than in normal tissues (Fig. 15), which was consistent with that at the transcriptional level. Among the 5 hub genes, one hub gene (CFD) was not reported in the HPA database.

### GSEA of the Hub Genes

**Figure 16:**
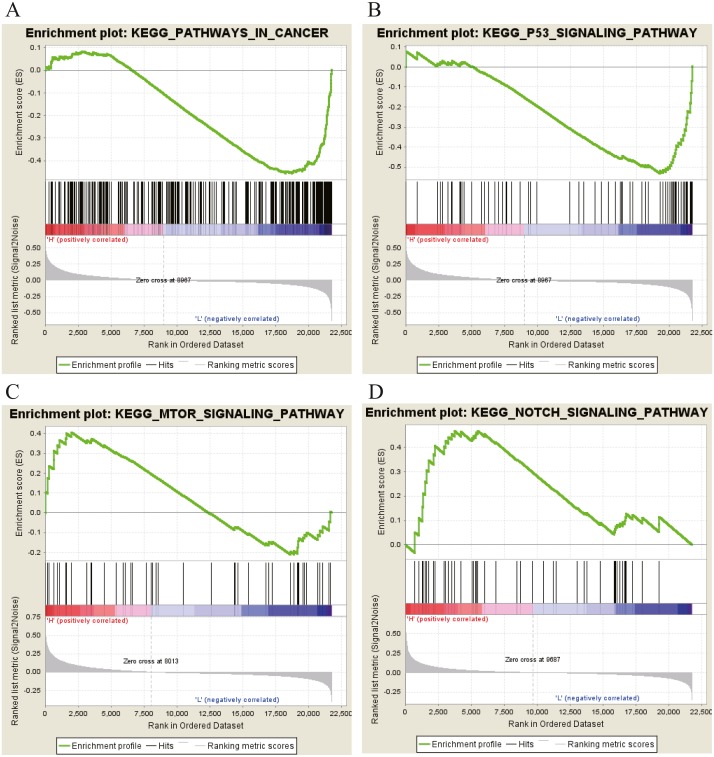
KEGG pathway enrichment analysis of five key genes. (A) Enrichment of genes in the KEGG PATHWAYS IN CANCER by GSEA. (B) Enrichment of genes in the KEGG P53 SIGNALING PATHWAY by GSEA. (C) Enrichment of genes in the KEGG MTOR SIGNALING PATHWAY by GSEA. (D) Enrichment of genes in the KEGG NOTCH SIGNALING PATHWAY by GSEA. The GSEA software was used to calculate enrichment levels.

**Figure 17:**
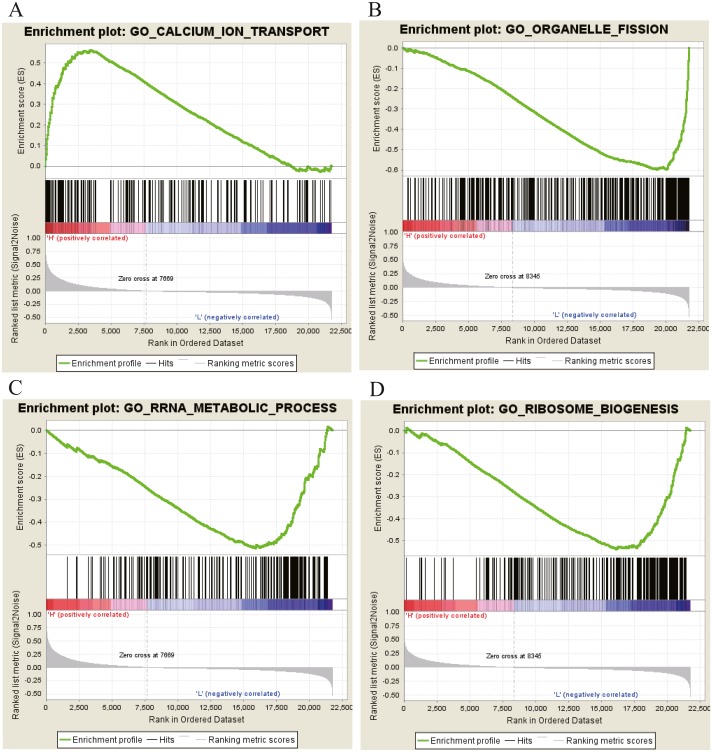
GO enrichment analysis of 5 key genes. (A) Enrichment of genes in GO CALCIUM ION TRANSPORT by GSEA. (B) Enrichment of genes in GO ORGANELLE FISSION by GSEA. (C) Enrichment of genes in GO RRRA METABOLIC PROCESS by GSEA. (D) Enrichment of genes in GO RIBOSOME BIOGENESIS by GSEA. The GSEA software was used to calculate enrichment levels.

Moreover, GSEA was conducted to search the potential biological function and signaling pathway of the above five hub genes (PPP1R12B, CFD, CRYAB, FAM189A2 and ANGPTL1). The result of GESA indicated that the 5 hub genes were significantly involved in critical biological functions and signal pathways that were correlated with carcinogenesis and progression of tumor, such as pathway in cancer, P53 signal pathway, MTOR signal pathway, Notch signal pathway, cell cycle, RRNA metabolic process, ribosome biogenesis and calcium ion transport (Figs. 16 and 17).

## Discussion

OSCC is one of the most complex and common malignant cancers. Despite significant improvements in the diagnosis, prognosis, and treatment of OSCC during the last decades, the 5-year overall survival rate is still very poor at approximately 50% due to local recurrence and metastasis (Scott, Grunfeld & McGurk, 2005; Omar, 2013). Therefore, to better explore novel and precise molecular biomarkers that could accurately and specifically predict the progression, recurrence and prognosis of OSCC patients (Mehrotra & Gupta, 2011), we used RNA sequencing data with clinical information from the TCGA and GEO databases to investigate and validate potential key modules and hub genes by bioinformatics analysis with WGCNA.

WGCNA is a powerful tool for analysing multiple genes in large-scale datasets. It has been extensively used to explore gene co-expression modules and hub genes as potential target biomarkers in many cancers (Foroughi et al., 2018; Chen et al., 2018; Giulietti et al., 2016; Liu et al., 2019; Wang et al., 2019a; Wang et al., 2019b; Xu et al., 2018; Zhai et al., 2019; Zhang et al., 2019a; Zhang et al., 2019b; Zhang et al., 2019c). In the current study, we applied WGCNA and systematically identified the turquoise module as the most significantly negatively associated (*r* =  − 0.2, *P* = 4e−04) with pathologic stage for the first time. It is well known that the pathologic stage is significantly correlated with the survival of patients and mainly affects the proliferation rate and tissue invasion ability of tumours. One study by Zhou et al. (2018a) and Zhou et al. (2018b) suggested that patients with a higher pathologic stage were associated with a significantly higher risk of recurrence and worse survival. Moreover, it has been reported that the clinicopathological stage in OSCC has survival implications (Kılıç et al., 2018).

Through overlap analysis and Kaplan–Meier survival analysis between the turquoise module and the TCGA and GEO (GSE30784) datasets, 5 common hub genes (PPP1R12B, CFD, CRYAB, FAM189A2 and ANGPTL1) had high connectivity with the overall survival of OSCC patients and were selected from the turquoise module. Survival analyses showed that low expression levels of the 5 hub genes were significantly correlated with poorer prognoses in OSCC patients.

At present, it has been reported in previous studies that these five hub genes are related to cancer, and their expression has been confirmed to play an important role in tumourigenesis, the malignant phenotype and disease prognosis. Another study (Ding et al., 2019) have also reported that the pseudopodium-enriched atypical kinase 1-PPP1R12B axis inhibits colorectal tumourigenesis and metastasis through the deactivation of the Grb2/PI3K/Akt pathway, which might provide a novel therapeutic strategy for CRC treatment. The hub gene CFD was identified and validated as a potential target gene in papillary thyroid cancer (Zhang et al., 2019a; Zhang et al., 2019b; Zhang et al., 2019c). CRYAB is a very important protein involved in a variety of signal transduction pathways, including apoptosis, inflammation and oxidative stress (Zhang et al., 2019a; Zhang et al., 2019b; Zhang et al., 2019c). CRYAB is a member of the small heat shock protein family (Zhang et al., 2019a; Zhang et al., 2019b; Zhang et al., 2019c), and many studies have confirmed that CRYAB plays an important role in a variety of tumours, such as OSCC (Annertz et al., 2014), colorectal cancer (Li et al., 2017), breast cancer (Kim et al., 2011), and hepatocellular carcinoma (Tang et al., 2009). One study (Wojtas et al., 2017) confirmed that the differential expression of FAM189A2 can serve as a gene expression marker in thyroid tumours. ANGPTL1 repressed the migration and invasion of colorectal cancer cells and was inversely correlated with poor survival (Chen et al., 2017). The results show that OSCC may be regulated by multiple genes, which will provide more ideas for the evaluation of prognostic value.

In the validation dataset of TCGA and GEO, the results indicated that the 5 hub genes were significantly downregulated in OSCC tissues. Moreover, two of the five genes in the turquoise module were also successfully validated by the HPA database, and the results were consistent with those at the transcriptional level. The above results indicate that the analysis results are reliable and convincing.

To further study the function and pathway regulation mechanism of tumourigenesis, we carried out GO annotation analysis, KEGG pathway analysis and GSEA. Then, some significant biological functions and signalling pathways related to tumourigenesis were identified. Functional annotation analysis showed that the genes were significantly enriched in cell differentiation, RRNA metabolic process, ribosome biogenesis, and cell–cell junctions. Moreover, KEGG pathway analysis showed that the genes are mostly involved in the MAPK signalling pathway, P53 signal pathway, MTOR signal pathway, Notch signal pathway, Wnt signalling pathway and calcium signalling pathway. At the same time, we also found that mutations or abnormal expression levels of these functional annotations and signalling pathways have been reported in OSCC and many other cancers (Guo et al., 2017; Hu et al., 2018; Huang et al., 2017; Jiang et al., 2011; Kim et al., 2019; Mo et al., 2019). These results provide more clues for further exploring the molecular regulation mechanism of the occurrence and development in OSCC.

## Conclusions

In summary, our research attempts to explore the potential molecular regulatory mechanism of OSCC on the basis of comprehensive bioinformatics analysis. We first discovered that the turquoise module was significantly negatively correlated with the pathologic stage for the first time. Moreover, PPP1R12B, CFD, CRYAB, FAM189A2 and ANGPTL1, as potential targets in OSCC, were from the turquoise module and their low expression levels were related to the poor survival prognosis of OSCC patients. Despite these findings having enormous potential value, there were some limitations to our study. These results still need further verification by detailed laboratory experiments and large-scale studies.

##  Supplemental Information

10.7717/peerj.8505/supp-1 File S1 The raw data of lncRNA obtained from the TCGA database

10.7717/peerj.8505/supp-2 File S2 The raw data of miRNA and mRNA obtained from the TCGA database

10.7717/peerj.8505/supp-3 File S3 WGCNA data

10.7717/peerj.8505/supp-4 File S4 The data of overlapping analyses

10.7717/peerj.8505/supp-5 File S5 The data of KEGG and GO about the turquoise module

10.7717/peerj.8505/supp-6 File S6 The data of GSEA about the five hub genes

## Additional Information and Declarations

### Data Availability

The following information was supplied regarding data availability: The raw measurements are available in the Supplemental Files.
