# Supplementary figures and images for: Identification and validation of key modules and hub genes associated with the pathological stage of oral squamous cell carcinoma by weighted gene co-expression network analysis
Source: PeerJ. 2020 Feb 4;8:e8505. doi: 10.7717/peerj.8505 (PMC7006519; doi:10.7717/peerj.8505)

**Module membership vs. gene significance**  
**cor=-0.17, p=0.3**

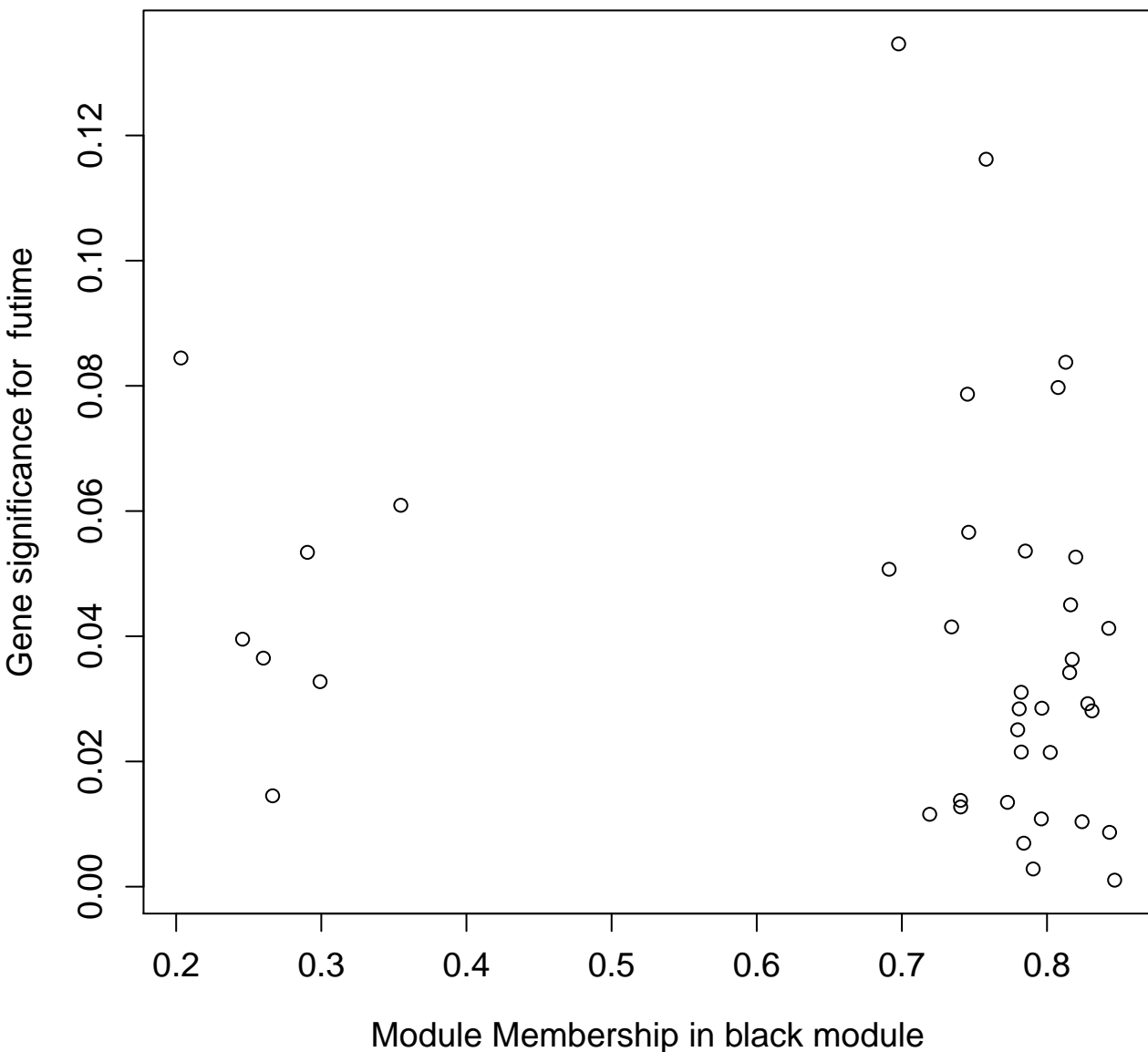

Supplement: File S3 [file peerj-08-8505-s003.zip › module_trait/9_futime_black.pdf]

**Module membership vs. gene significance**  
**cor=-0.15, p=1.2e-05**

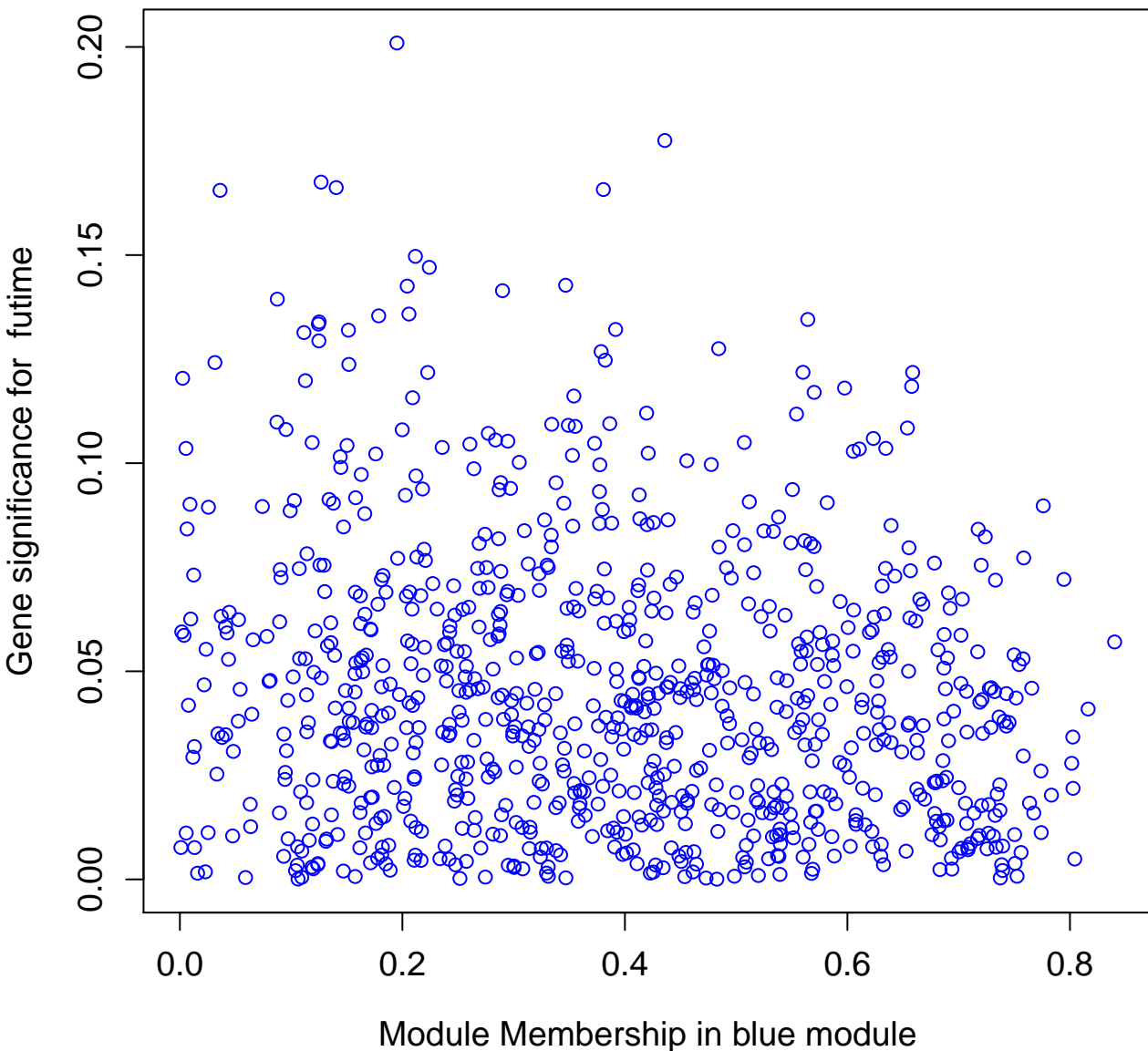

Supplement: File S3 [file peerj-08-8505-s003.zip › module_trait/9_futime_blue.pdf]

**Module membership vs. gene significance**  
**cor=-0.2, p=2e-05**

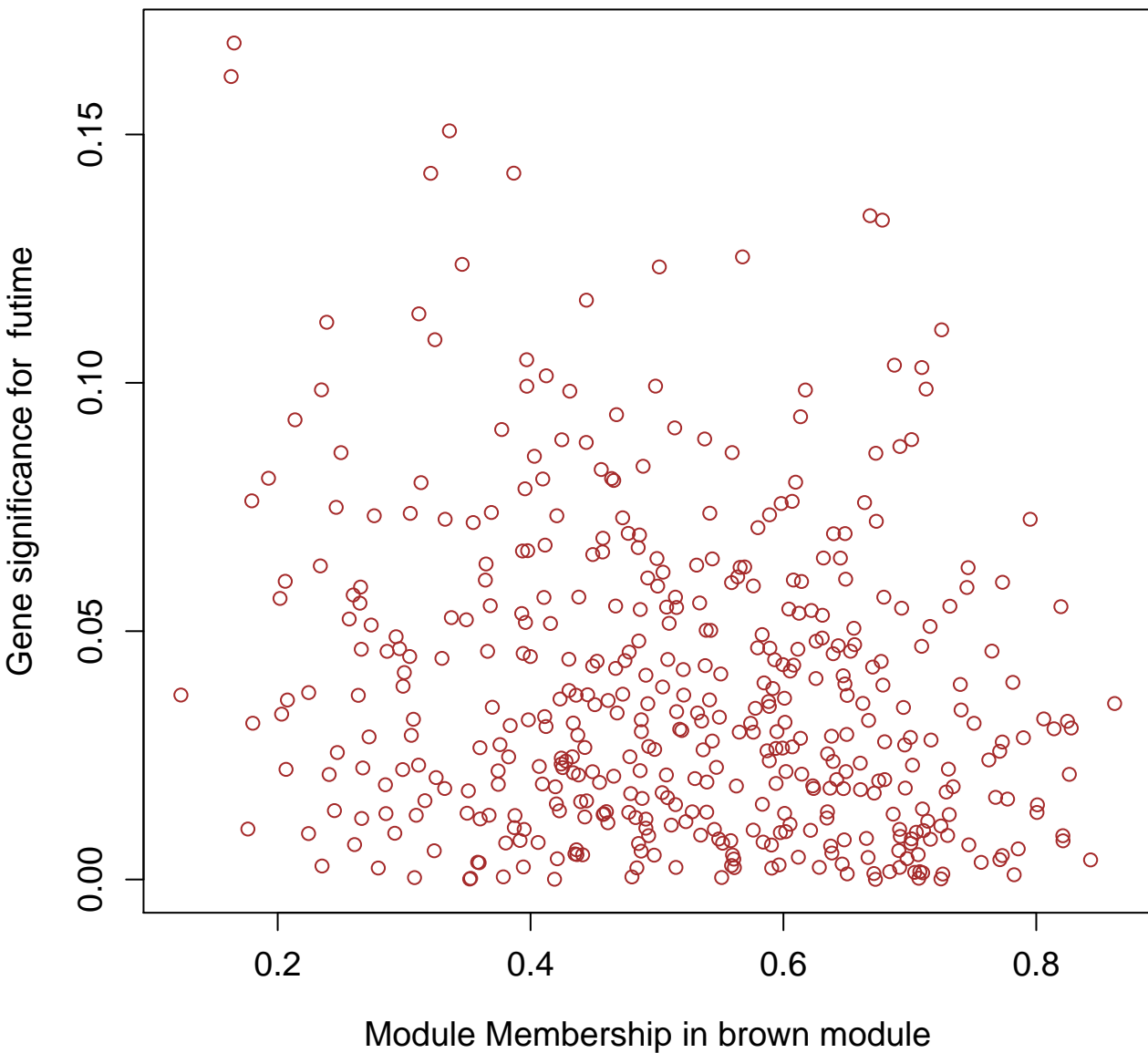

Supplement: File S3 [file peerj-08-8505-s003.zip › module_trait/9_futime_brown.pdf]

**Module membership vs. gene significance**  
**cor=-0.039, p=0.69**

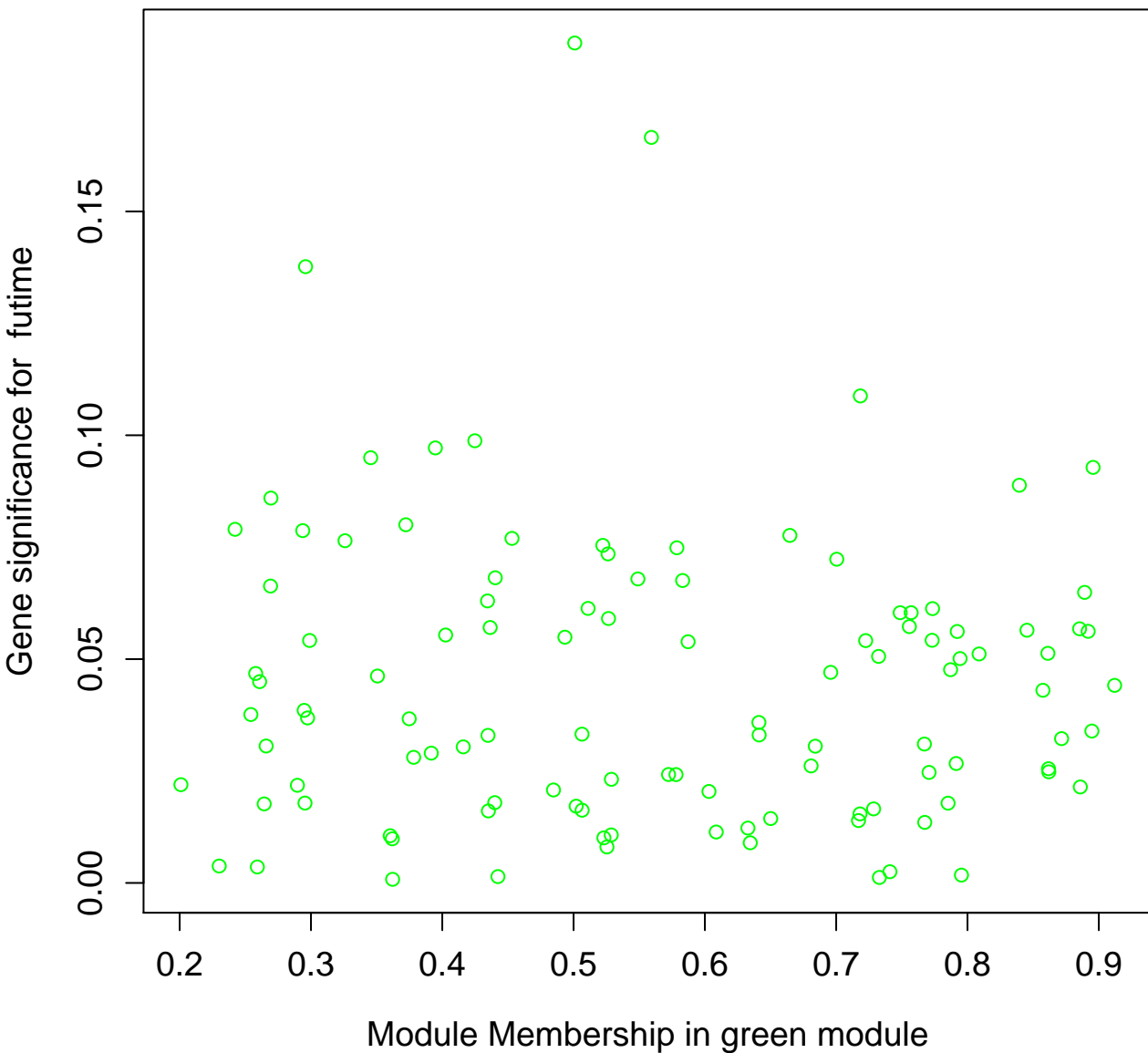

Supplement: File S3 [file peerj-08-8505-s003.zip › module_trait/9_futime_green.pdf]

**Module membership vs. gene significance**  
**cor=0.11, p=0.45**

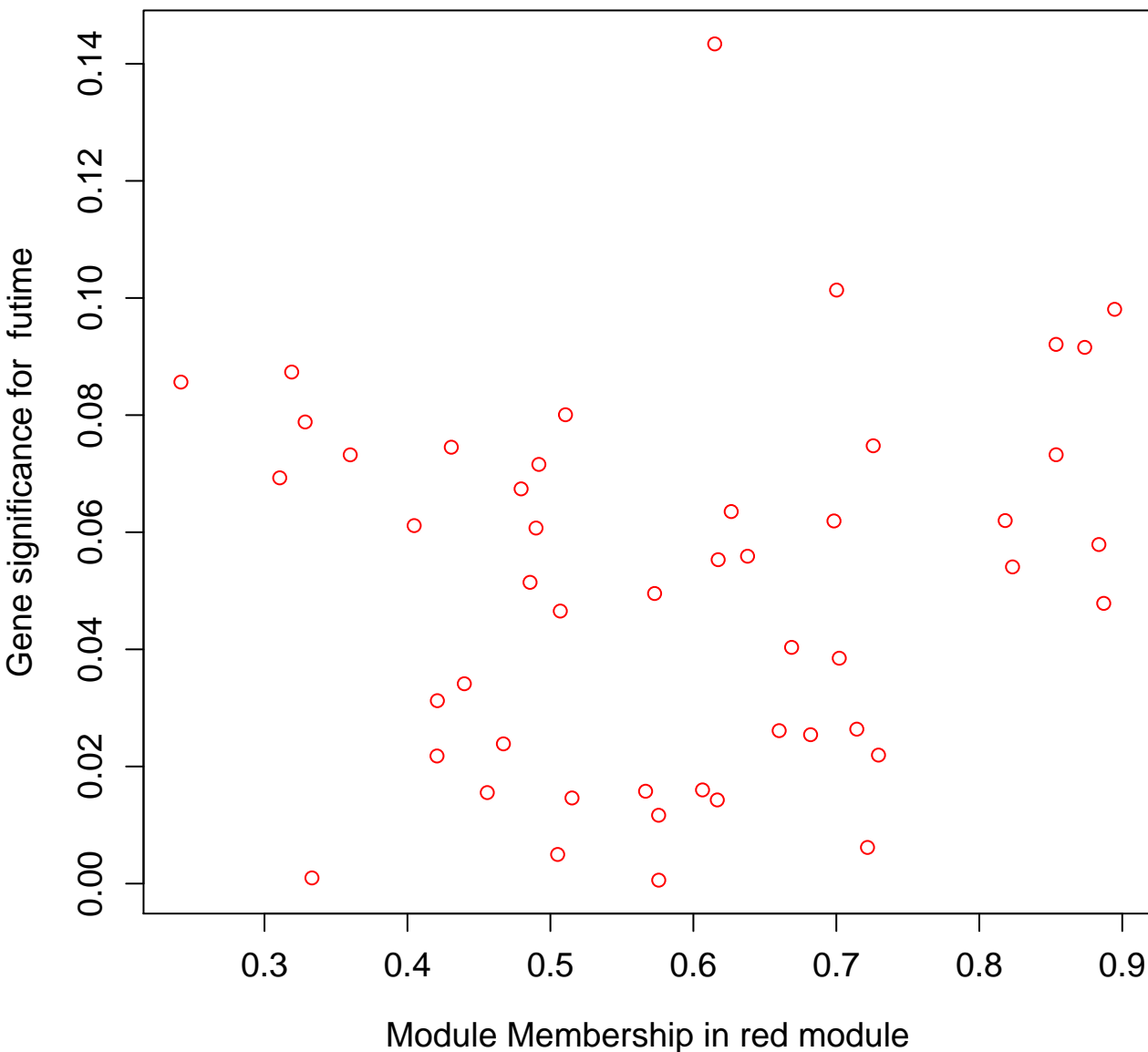

Supplement: File S3 [file peerj-08-8505-s003.zip › module_trait/9_futime_red.pdf]

**Module membership vs. gene significance**  
**cor=0.22, p=3.9e-12**

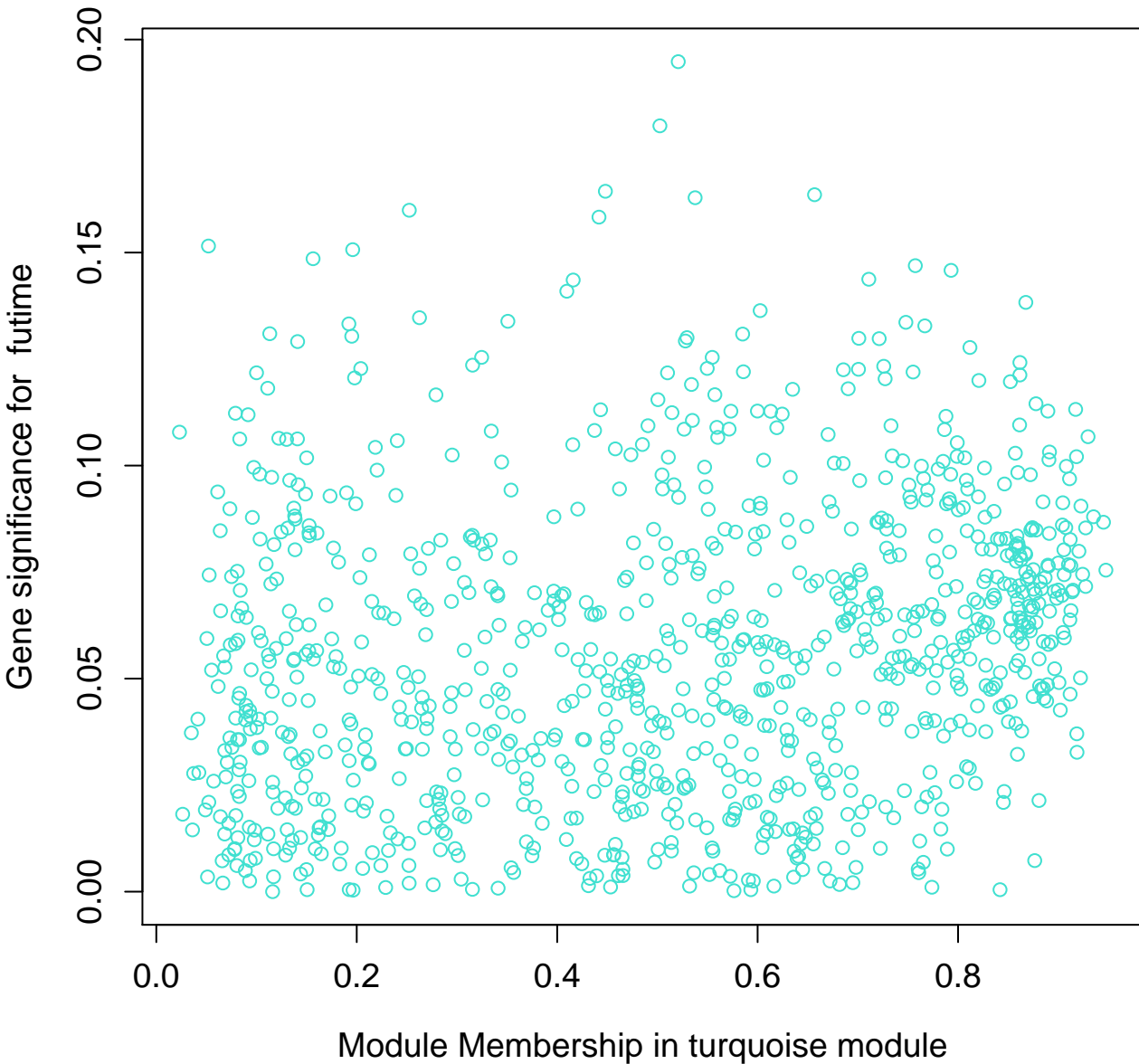

Supplement: File S3 [file peerj-08-8505-s003.zip › module_trait/9_futime_turquoise.pdf]

**Module membership vs. gene significance**  
**cor=-0.2, p=0.00076**

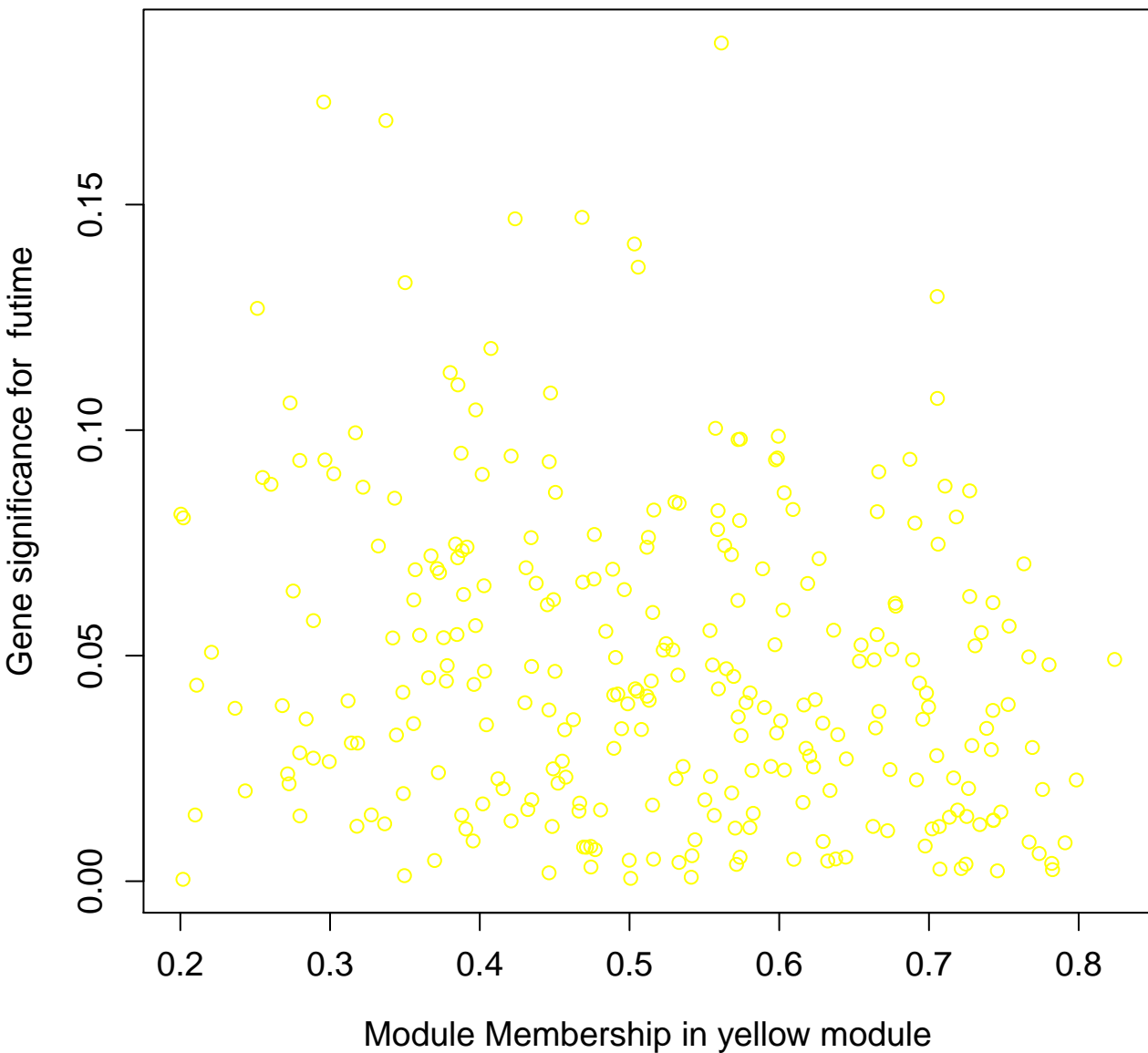

Supplement: File S3 [file peerj-08-8505-s003.zip › module_trait/9_futime_yellow.pdf]

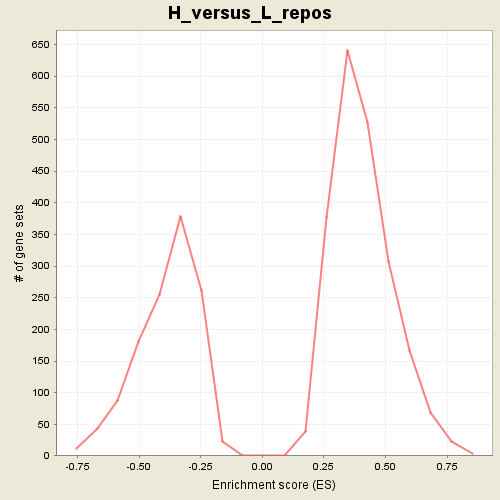

Supplement: File S6 [file peerj-08-8505-s006.zip › my_analysis_201957_BP.Gsea.1570106074072/global_es_histogram.png]

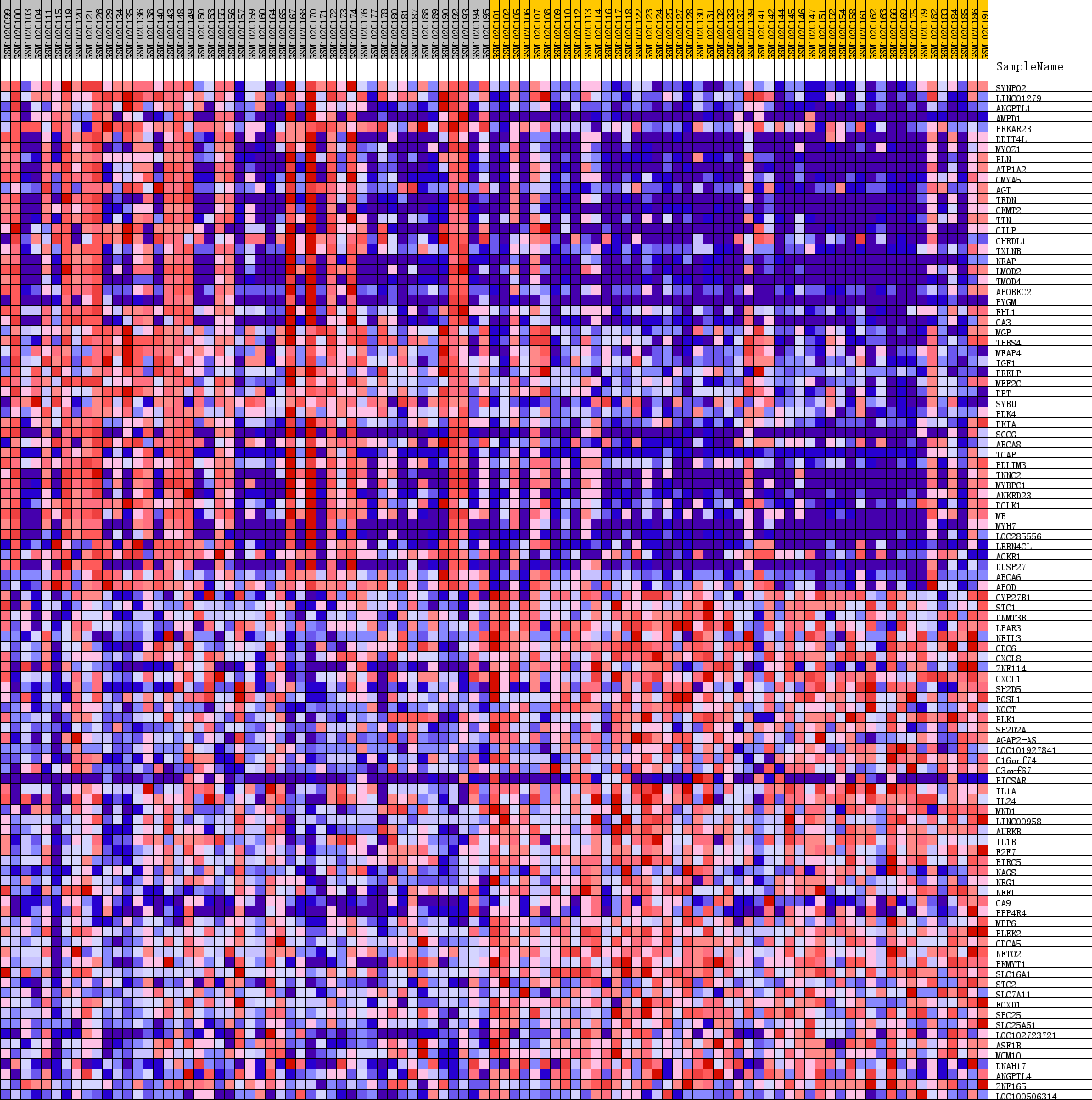

Supplement: File S6 [file peerj-08-8505-s006.zip › my_analysis_201957_BP.Gsea.1570106074072/heat_map_123.png]

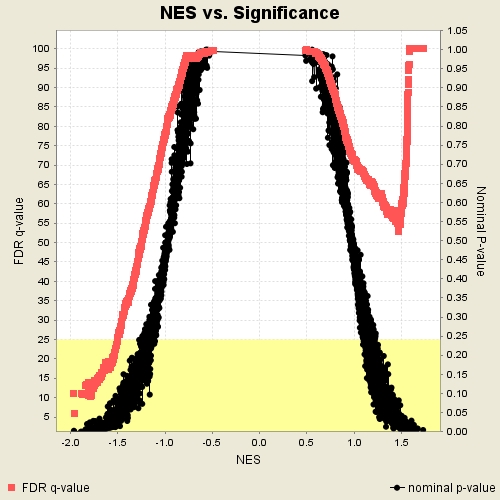

Supplement: File S6 [file peerj-08-8505-s006.zip › my_analysis_201957_BP.Gsea.1570106074072/pvalues_vs_nes_plot.png]

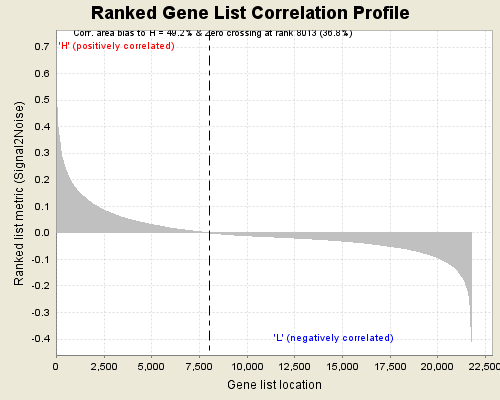

Supplement: File S6 [file peerj-08-8505-s006.zip › my_analysis_201957_BP.Gsea.1570106074072/ranked_list_corr_124.png]

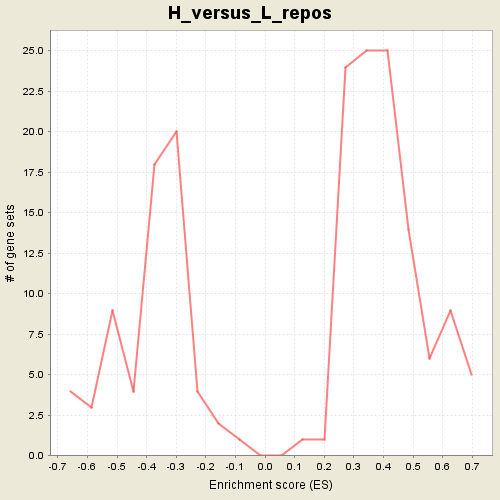

Supplement: File S6 [file peerj-08-8505-s006.zip › my_analysis_201957_KEGG.Gsea.1570105865824/global_es_histogram.png]

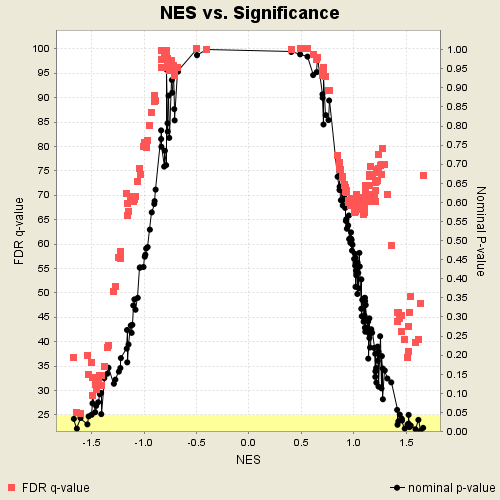

Supplement: File S6 [file peerj-08-8505-s006.zip › my_analysis_201957_KEGG.Gsea.1570105865824/pvalues_vs_nes_plot.png]

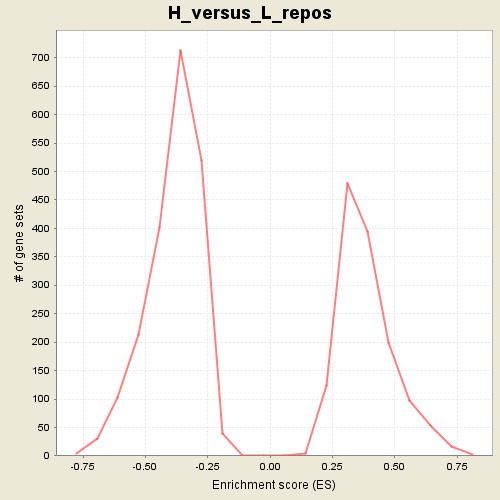

Supplement: File S6 [file peerj-08-8505-s006.zip › my_analysis_205382_BP.Gsea.1570106097809/global_es_histogram.png]

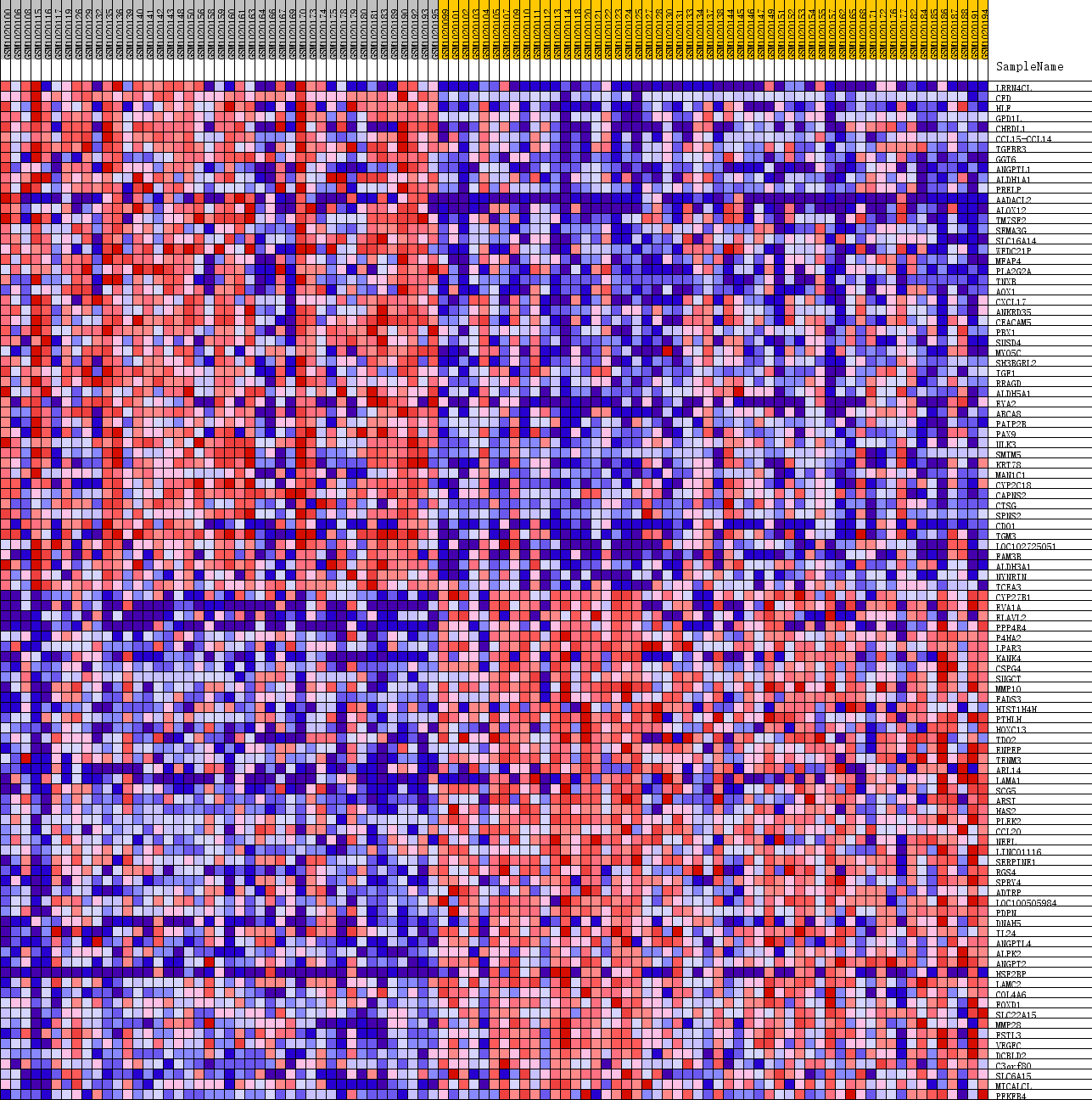

Supplement: File S6 [file peerj-08-8505-s006.zip › my_analysis_205382_BP.Gsea.1570106097809/heat_map_123.png]

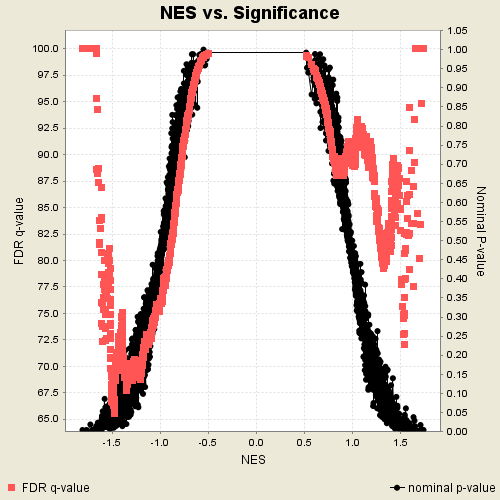

Supplement: File S6 [file peerj-08-8505-s006.zip › my_analysis_205382_BP.Gsea.1570106097809/pvalues_vs_nes_plot.png]

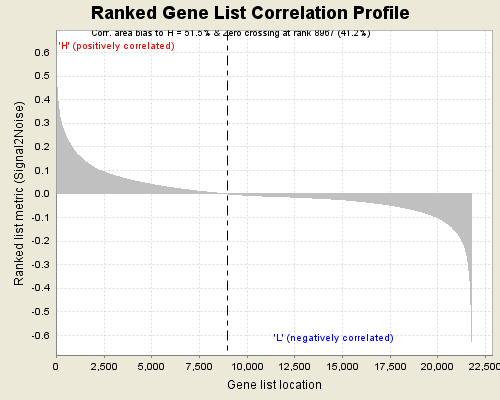

Supplement: File S6 [file peerj-08-8505-s006.zip › my_analysis_205382_BP.Gsea.1570106097809/ranked_list_corr_124.png]

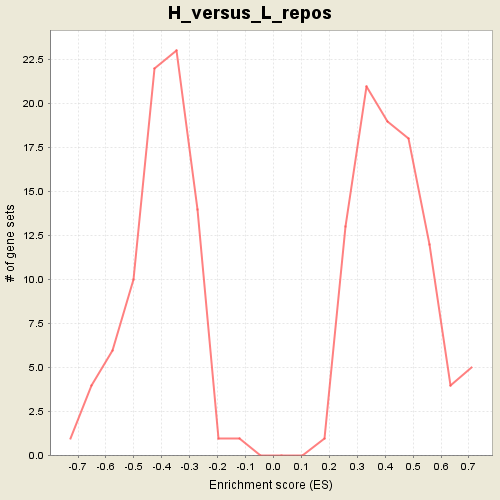

Supplement: File S6 [file peerj-08-8505-s006.zip › my_analysis_205382_KEGG.Gsea.1570105897088/global_es_histogram.png]

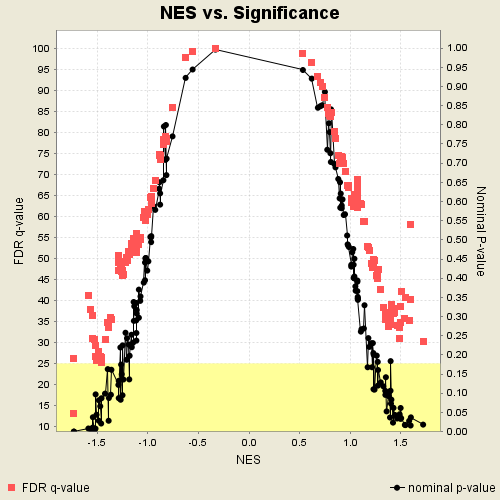

Supplement: File S6 [file peerj-08-8505-s006.zip › my_analysis_205382_KEGG.Gsea.1570105897088/pvalues_vs_nes_plot.png]

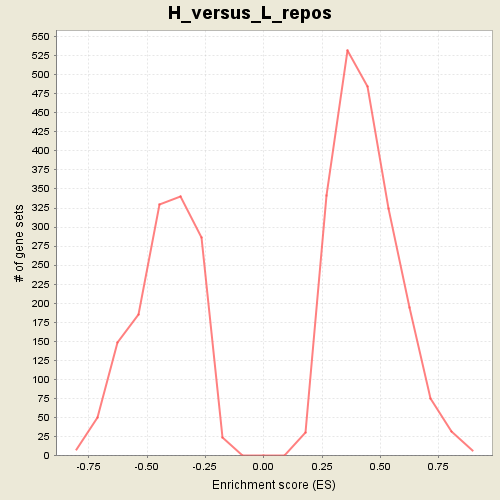

Supplement: File S6 [file peerj-08-8505-s006.zip › my_analysis_209283_BP.Gsea.1570106107776/global_es_histogram.png]

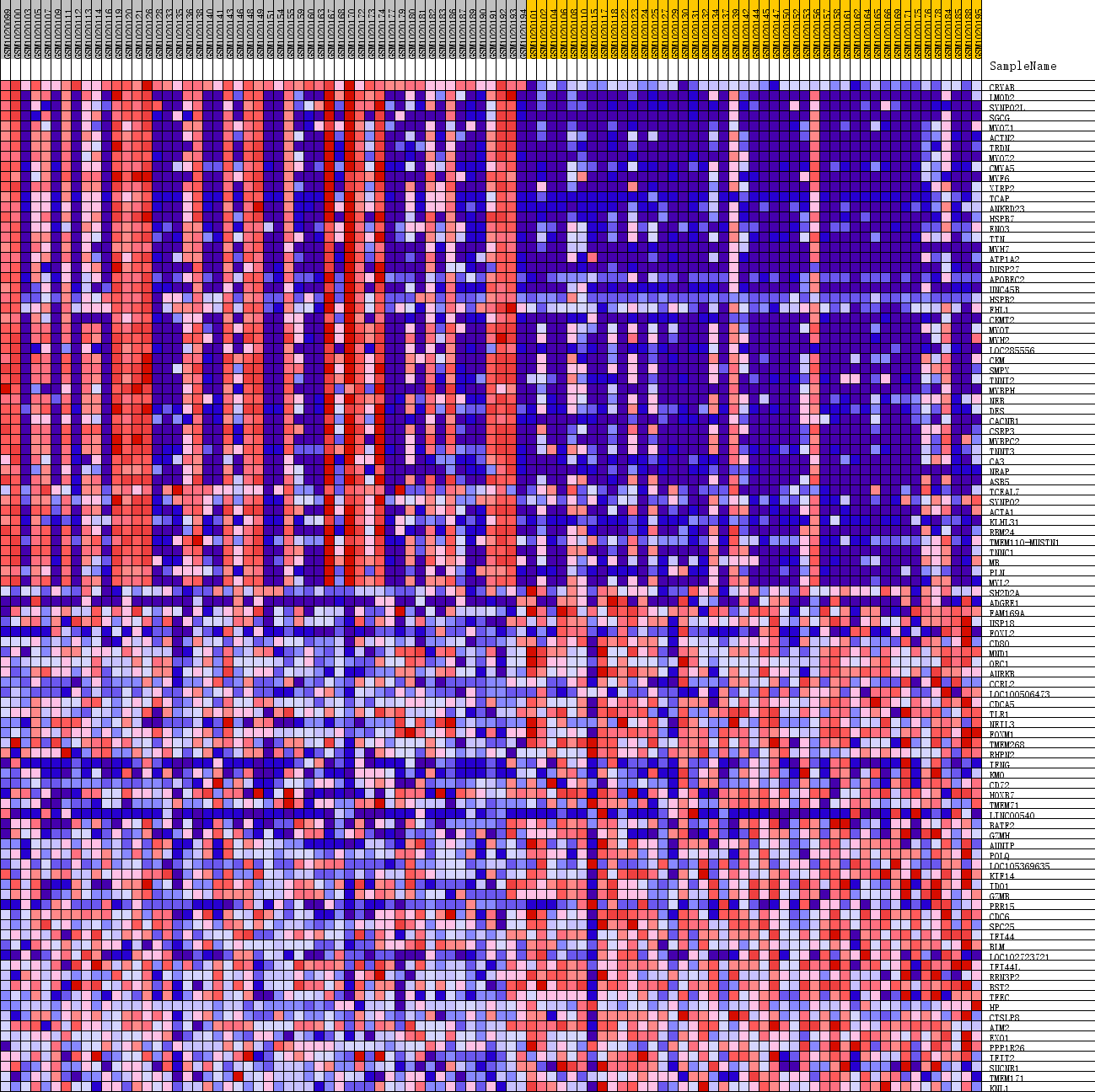

Supplement: File S6 [file peerj-08-8505-s006.zip › my_analysis_209283_BP.Gsea.1570106107776/heat_map_123.png]

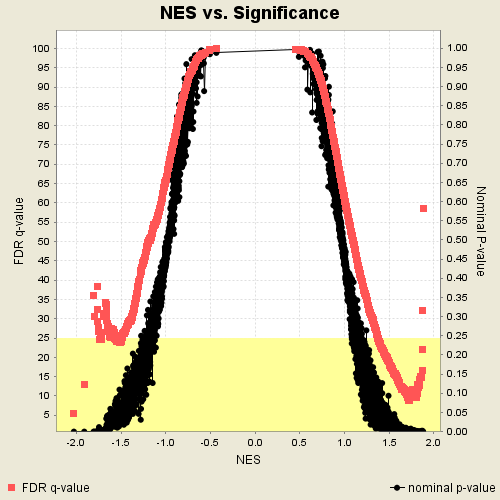

Supplement: File S6 [file peerj-08-8505-s006.zip › my_analysis_209283_BP.Gsea.1570106107776/pvalues_vs_nes_plot.png]

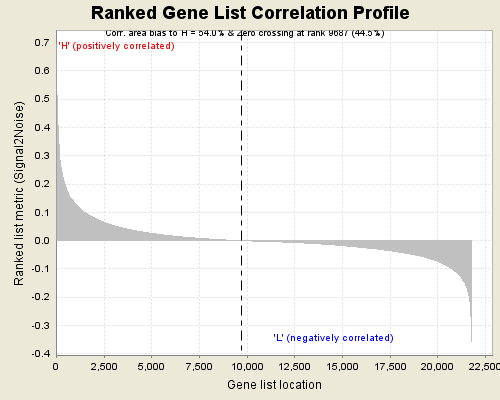

Supplement: File S6 [file peerj-08-8505-s006.zip › my_analysis_209283_BP.Gsea.1570106107776/ranked_list_corr_124.png]

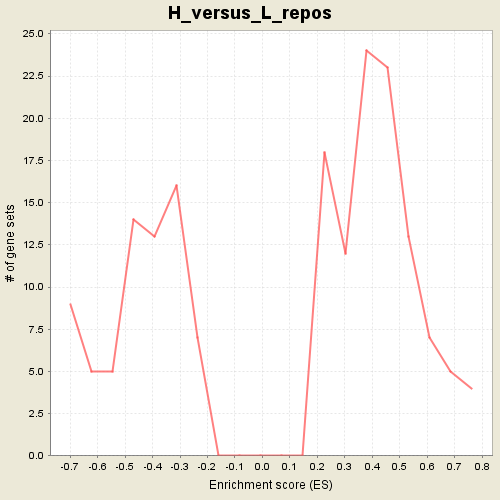

Supplement: File S6 [file peerj-08-8505-s006.zip › my_analysis_209283_KEGG.Gsea.1570105930256/global_es_histogram.png]

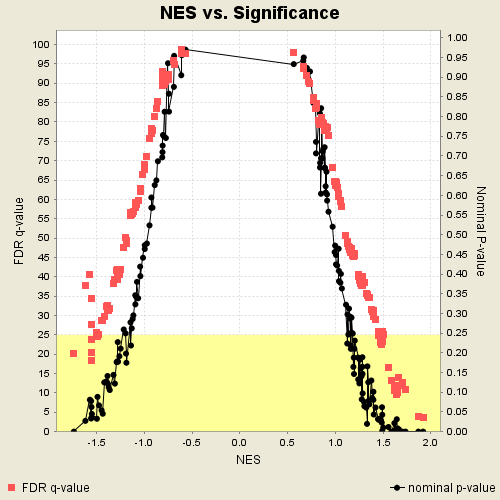

Supplement: File S6 [file peerj-08-8505-s006.zip › my_analysis_209283_KEGG.Gsea.1570105930256/pvalues_vs_nes_plot.png]

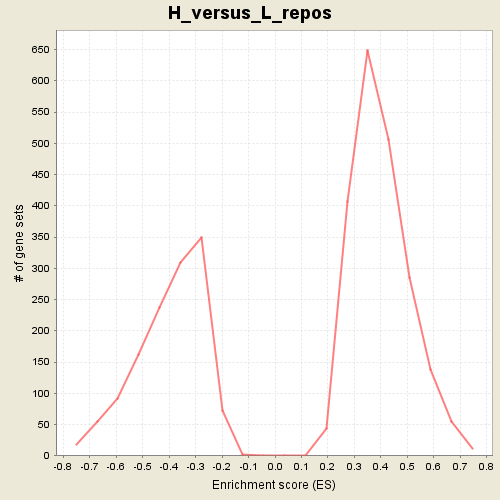

Supplement: File S6 [file peerj-08-8505-s006.zip › my_analysis_213900_BP.Gsea.1570106614819/global_es_histogram.png]

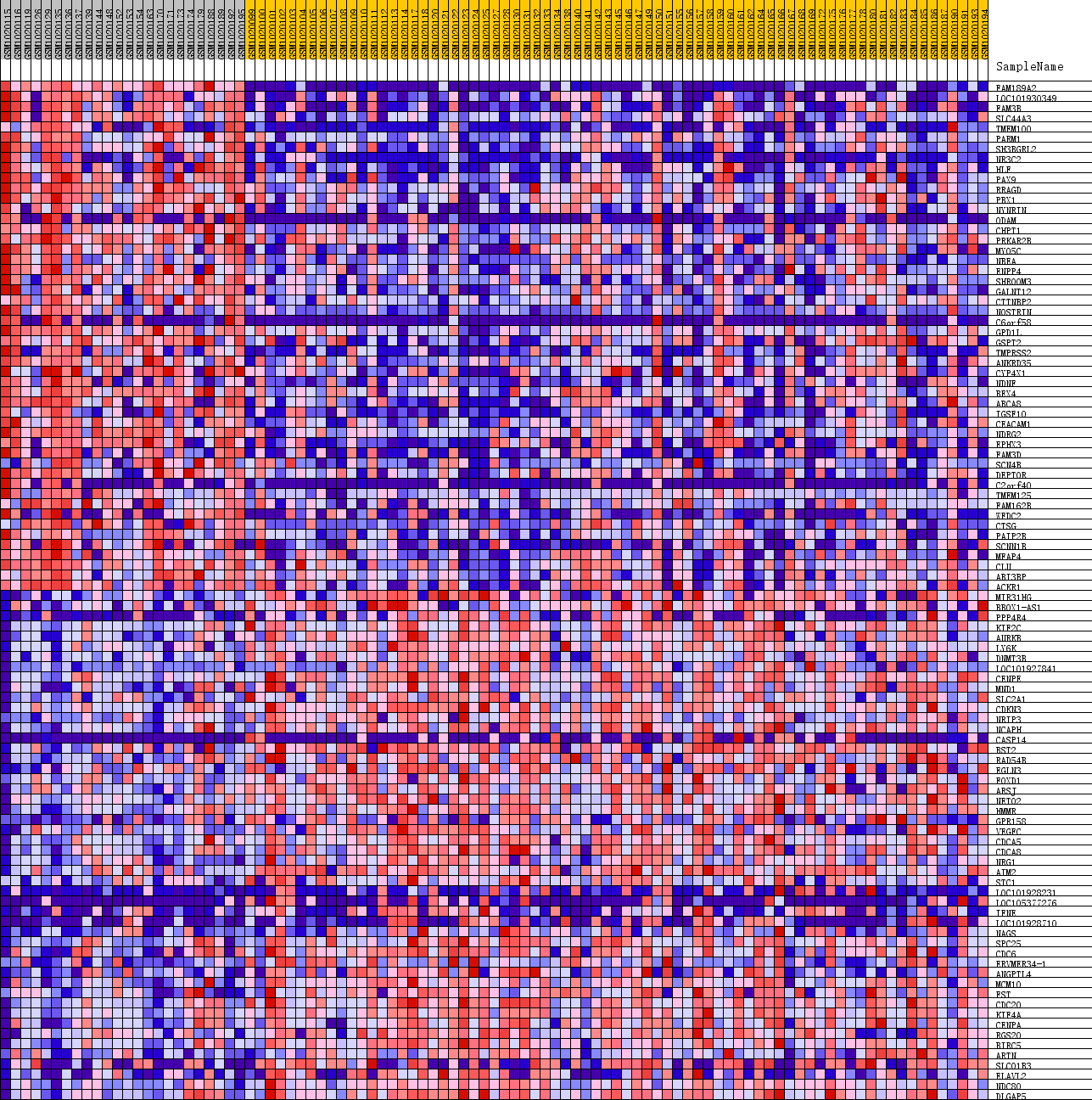

Supplement: File S6 [file peerj-08-8505-s006.zip › my_analysis_213900_BP.Gsea.1570106614819/heat_map_245.png]

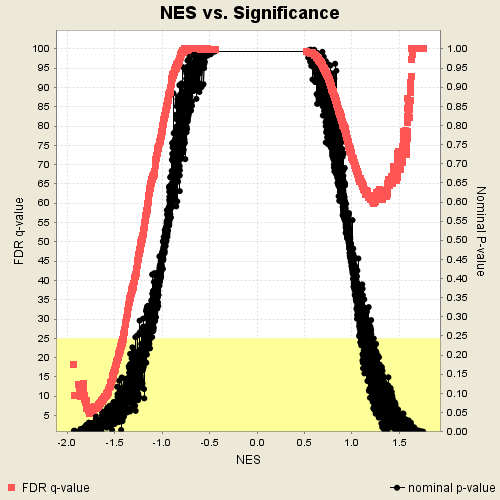

Supplement: File S6 [file peerj-08-8505-s006.zip › my_analysis_213900_BP.Gsea.1570106614819/pvalues_vs_nes_plot.png]

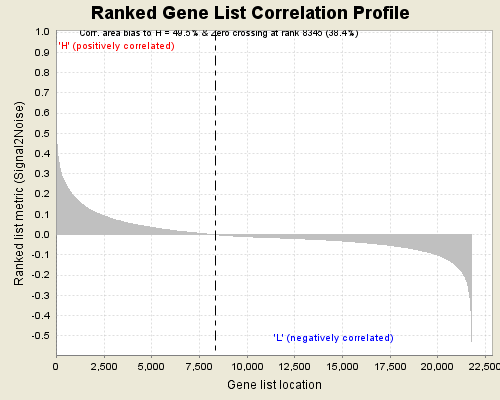

Supplement: File S6 [file peerj-08-8505-s006.zip › my_analysis_213900_BP.Gsea.1570106614819/ranked_list_corr_246.png]

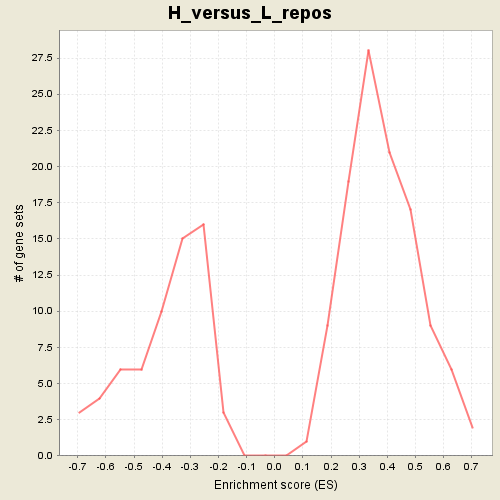

Supplement: File S6 [file peerj-08-8505-s006.zip › my_analysis_213900_KEGG.Gsea.1570107185647/global_es_histogram.png]

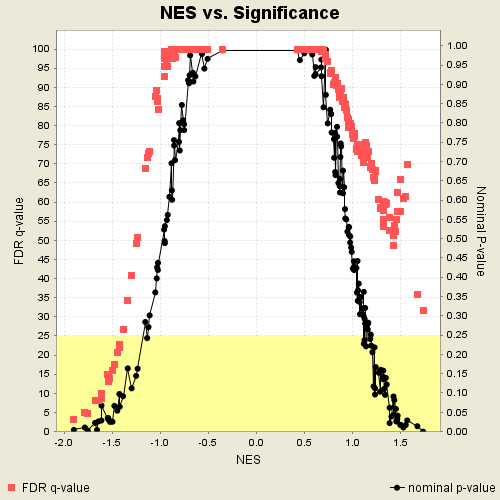

Supplement: File S6 [file peerj-08-8505-s006.zip › my_analysis_213900_KEGG.Gsea.1570107185647/pvalues_vs_nes_plot.png]

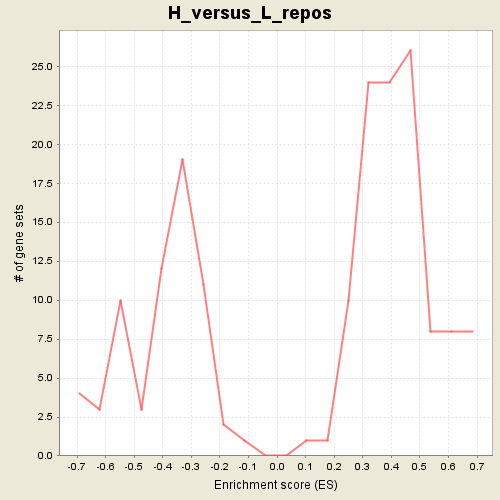

Supplement: File S6 [file peerj-08-8505-s006.zip › my_analysis_231773_kegg.Gsea.1570107186948/global_es_histogram.png]

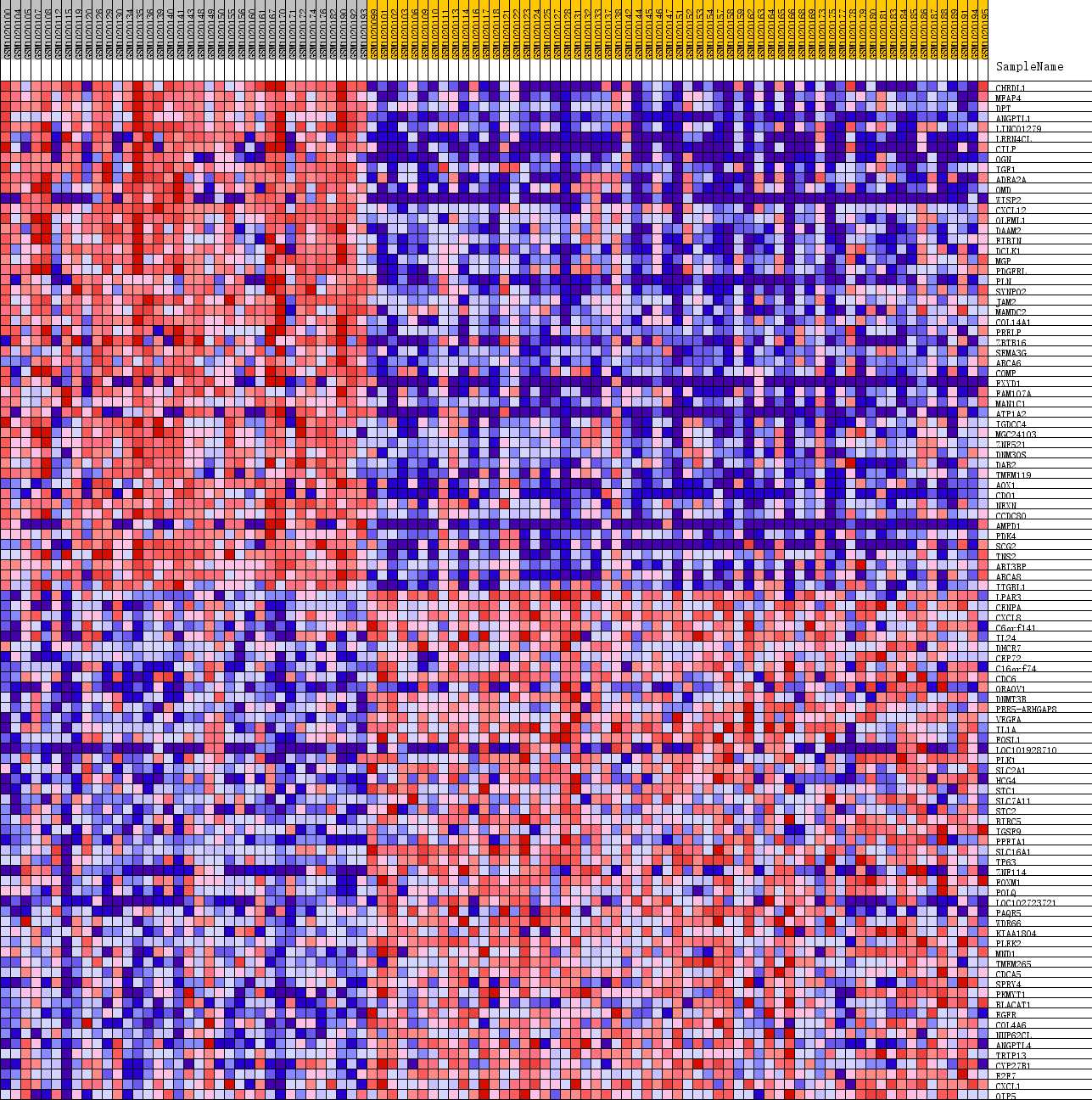

Supplement: File S6 [file peerj-08-8505-s006.zip › my_analysis_231773_kegg.Gsea.1570107186948/heat_map_367.png]

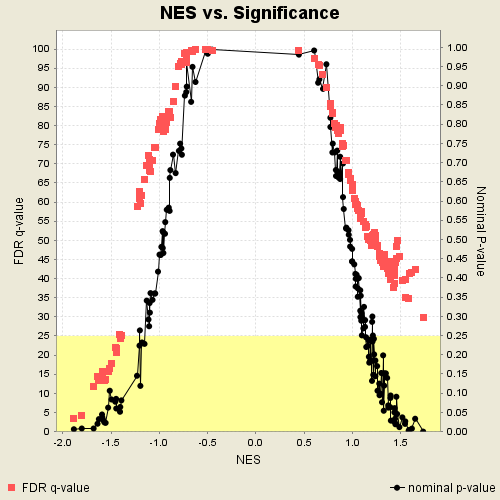

Supplement: File S6 [file peerj-08-8505-s006.zip › my_analysis_231773_kegg.Gsea.1570107186948/pvalues_vs_nes_plot.png]

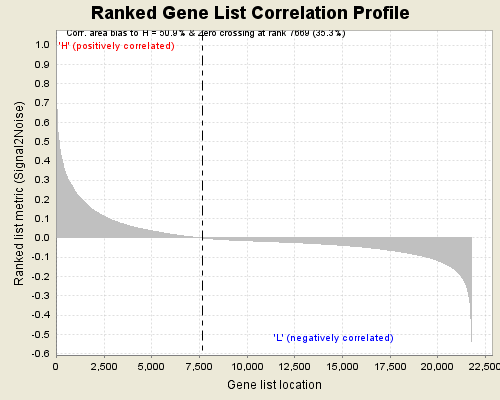

Supplement: File S6 [file peerj-08-8505-s006.zip › my_analysis_231773_kegg.Gsea.1570107186948/ranked_list_corr_368.png]

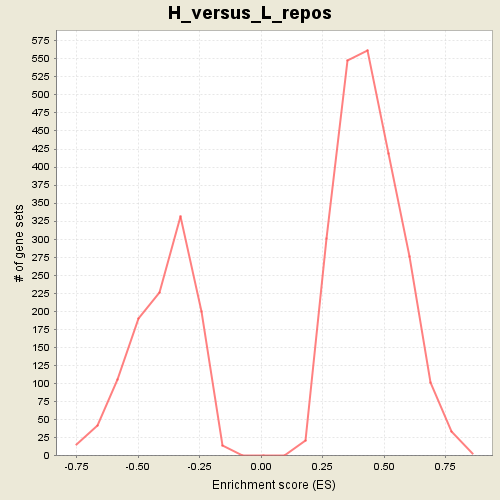

Supplement: File S6 [file peerj-08-8505-s006.zip › my_analysis_23177_BP.Gsea.1570106638719/global_es_histogram.png]

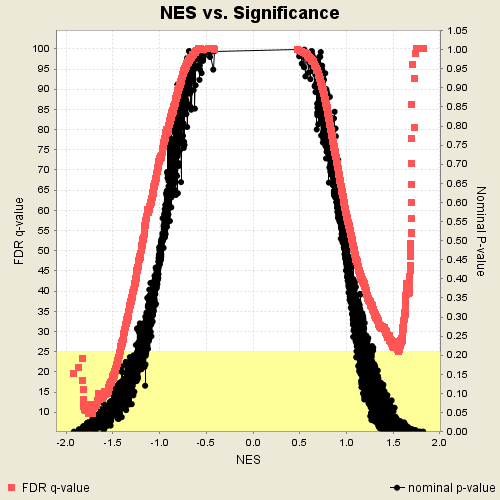

Supplement: File S6 [file peerj-08-8505-s006.zip › my_analysis_23177_BP.Gsea.1570106638719/pvalues_vs_nes_plot.png]
