# Supplementary material for: Identification and validation of key modules and hub genes associated with the pathological stage of oral squamous cell carcinoma by weighted gene co-expression network analysis
Source: PeerJ. 2020 Feb 4;8:e8505. doi: 10.7717/peerj.8505 (PMC7006519; doi:10.7717/peerj.8505)
Supplement: File S6 [file peerj-08-8505-s006.zip › my_analysis_201957_BP.Gsea.1570106074072/gsea_report_for_L_1570106074072.html]

Report for L 1570106074072 [GSEA]

| GS  follow link to MSigDB | GS DETAILS | SIZE | ES | NES | NOM p-val | FDR q-val | FWER p-val | RANK AT MAX | LEADING EDGE || 1 | GO\_RIBOSOMAL\_SMALL\_SUBUNIT\_BIOGENESIS | Details ... | 52 | -0.68 | -1.96 | 0.002 | 0.111 | 0.095 | 3894 | tags=52%, list=18%, signal=63% |
| 2 | GO\_RIBOSOMAL\_SMALL\_SUBUNIT\_ASSEMBLY | Details ... | 15 | -0.72 | -1.96 | 0.000 | 0.060 | 0.101 | 3815 | tags=40%, list=18%, signal=48% |
| 3 | GO\_POSITIVE\_REGULATION\_OF\_LIGASE\_ACTIVITY | Details ... | 102 | -0.61 | -1.89 | 0.000 | 0.110 | 0.223 | 4066 | tags=47%, list=19%, signal=58% |
| 4 | GO\_REGULATION\_OF\_LIGASE\_ACTIVITY | Details ... | 122 | -0.58 | -1.87 | 0.000 | 0.107 | 0.275 | 4120 | tags=44%, list=19%, signal=54% |
| 5 | GO\_RIBOSOME\_BIOGENESIS | Details ... | 265 | -0.54 | -1.84 | 0.006 | 0.130 | 0.364 | 5154 | tags=45%, list=24%, signal=58% |
| 6 | GO\_RRNA\_METABOLIC\_PROCESS | Details ... | 217 | -0.52 | -1.83 | 0.020 | 0.134 | 0.414 | 5240 | tags=42%, list=24%, signal=55% |
| 7 | GO\_TRANSCRIPTION\_ELONGATION\_FROM\_RNA\_POLYMERASE\_II\_PROMOTER | Details ... | 73 | -0.55 | -1.81 | 0.006 | 0.139 | 0.470 | 5931 | tags=49%, list=27%, signal=68% |
| 8 | GO\_RNA\_CAPPING | Details ... | 33 | -0.65 | -1.81 | 0.002 | 0.127 | 0.486 | 5807 | tags=67%, list=27%, signal=91% |
| 9 | GO\_MATURATION\_OF\_SSU\_RRNA | Details ... | 38 | -0.69 | -1.81 | 0.004 | 0.115 | 0.492 | 3894 | tags=61%, list=18%, signal=74% |
| 10 | GO\_PYRIMIDINE\_DEOXYRIBONUCLEOTIDE\_METABOLIC\_PROCESS | Details ... | 15 | -0.73 | -1.81 | 0.000 | 0.107 | 0.497 | 4678 | tags=73%, list=22%, signal=93% |
| 11 | GO\_NCRNA\_PROCESSING | Details ... | 332 | -0.52 | -1.80 | 0.024 | 0.110 | 0.532 | 5173 | tags=45%, list=24%, signal=59% |
| 12 | GO\_RIBONUCLEOPROTEIN\_COMPLEX\_BIOGENESIS | Details ... | 383 | -0.51 | -1.79 | 0.012 | 0.105 | 0.550 | 5244 | tags=43%, list=24%, signal=56% |
| 13 | GO\_DNA\_TEMPLATED\_TRANSCRIPTION\_ELONGATION | Details ... | 88 | -0.56 | -1.79 | 0.010 | 0.102 | 0.563 | 5304 | tags=49%, list=24%, signal=64% |
| 14 | GO\_MITOCHONDRIAL\_TRANSLATION | Details ... | 101 | -0.62 | -1.78 | 0.022 | 0.106 | 0.591 | 5995 | tags=69%, list=28%, signal=95% |
| 15 | GO\_ANAPHASE\_PROMOTING\_COMPLEX\_DEPENDENT\_CATABOLIC\_PROCESS | Details ... | 72 | -0.69 | -1.76 | 0.002 | 0.127 | 0.681 | 3980 | tags=53%, list=18%, signal=64% |
| 16 | GO\_TRANSLATIONAL\_TERMINATION | Details ... | 90 | -0.59 | -1.76 | 0.028 | 0.125 | 0.690 | 5995 | tags=71%, list=28%, signal=98% |
| 17 | GO\_RIBOSOME\_ASSEMBLY | Details ... | 46 | -0.57 | -1.74 | 0.011 | 0.143 | 0.754 | 4271 | tags=39%, list=20%, signal=49% |
| 18 | GO\_RIBONUCLEOPROTEIN\_COMPLEX\_SUBUNIT\_ORGANIZATION | Details ... | 177 | -0.47 | -1.74 | 0.028 | 0.137 | 0.761 | 5267 | tags=42%, list=24%, signal=55% |
| 19 | GO\_FEMALE\_MEIOTIC\_DIVISION | Details ... | 22 | -0.75 | -1.74 | 0.002 | 0.132 | 0.766 | 2504 | tags=50%, list=12%, signal=56% |
| 20 | GO\_NCRNA\_METABOLIC\_PROCESS | Details ... | 462 | -0.49 | -1.73 | 0.024 | 0.137 | 0.795 | 5304 | tags=44%, list=24%, signal=57% |
| 21 | GO\_POSITIVE\_REGULATION\_OF\_DNA\_TEMPLATED\_TRANSCRIPTION\_ELONGATION |  | 21 | -0.63 | -1.73 | 0.008 | 0.137 | 0.807 | 4385 | tags=48%, list=20%, signal=60% |
| 22 | GO\_MACROMOLECULAR\_COMPLEX\_DISASSEMBLY |  | 175 | -0.48 | -1.73 | 0.010 | 0.140 | 0.820 | 5028 | tags=48%, list=23%, signal=62% |
| 23 | GO\_SPLICEOSOMAL\_SNRNP\_ASSEMBLY |  | 36 | -0.62 | -1.72 | 0.008 | 0.139 | 0.826 | 4859 | tags=64%, list=22%, signal=82% |
| 24 | GO\_CELLULAR\_PROTEIN\_COMPLEX\_DISASSEMBLY |  | 118 | -0.55 | -1.72 | 0.026 | 0.139 | 0.840 | 5995 | tags=64%, list=28%, signal=87% |
| 25 | GO\_DNA\_DAMAGE\_RESPONSE\_DETECTION\_OF\_DNA\_DAMAGE |  | 36 | -0.65 | -1.71 | 0.006 | 0.148 | 0.871 | 4373 | tags=50%, list=20%, signal=62% |
| 26 | GO\_REGULATION\_OF\_SISTER\_CHROMATID\_SEGREGATION |  | 63 | -0.66 | -1.71 | 0.008 | 0.151 | 0.878 | 1914 | tags=33%, list=9%, signal=36% |
| 27 | GO\_DEOXYRIBONUCLEOTIDE\_METABOLIC\_PROCESS |  | 32 | -0.62 | -1.70 | 0.008 | 0.152 | 0.890 | 4678 | tags=66%, list=22%, signal=83% |
| 28 | GO\_PROTEIN\_K11\_LINKED\_UBIQUITINATION |  | 26 | -0.58 | -1.70 | 0.010 | 0.156 | 0.897 | 2942 | tags=31%, list=14%, signal=36% |
| 29 | GO\_MATURATION\_OF\_SSU\_RRNA\_FROM\_TRICISTRONIC\_RRNA\_TRANSCRIPT\_SSU\_RRNA\_5\_8S\_RRNA\_LSU\_RRNA\_ |  | 32 | -0.65 | -1.70 | 0.026 | 0.154 | 0.900 | 3894 | tags=56%, list=18%, signal=68% |
| 30 | GO\_POSITIVE\_REGULATION\_OF\_VIRAL\_TRANSCRIPTION |  | 35 | -0.49 | -1.69 | 0.023 | 0.150 | 0.900 | 5849 | tags=49%, list=27%, signal=66% |
| 31 | GO\_DNA\_TEMPLATED\_TRANSCRIPTION\_TERMINATION |  | 88 | -0.54 | -1.69 | 0.034 | 0.146 | 0.901 | 5807 | tags=56%, list=27%, signal=76% |
| 32 | GO\_CYTOKINESIS |  | 79 | -0.57 | -1.69 | 0.002 | 0.152 | 0.916 | 2354 | tags=32%, list=11%, signal=35% |
| 33 | GO\_PHOTOTRANSDUCTION\_VISIBLE\_LIGHT |  | 20 | -0.65 | -1.69 | 0.008 | 0.152 | 0.922 | 3104 | tags=35%, list=14%, signal=41% |
| 34 | GO\_REGULATION\_OF\_NUCLEAR\_DIVISION |  | 154 | -0.56 | -1.68 | 0.004 | 0.165 | 0.935 | 1914 | tags=31%, list=9%, signal=34% |
| 35 | GO\_NEGATIVE\_REGULATION\_OF\_PROTEIN\_MODIFICATION\_BY\_SMALL\_PROTEIN\_CONJUGATION\_OR\_REMOVAL |  | 126 | -0.51 | -1.67 | 0.014 | 0.166 | 0.938 | 4076 | tags=40%, list=19%, signal=50% |
| 36 | GO\_MATURATION\_OF\_5\_8S\_RRNA |  | 26 | -0.65 | -1.67 | 0.022 | 0.162 | 0.938 | 5154 | tags=73%, list=24%, signal=96% |
| 37 | GO\_REGULATION\_OF\_CHROMOSOME\_SEGREGATION |  | 80 | -0.66 | -1.67 | 0.006 | 0.157 | 0.938 | 1914 | tags=34%, list=9%, signal=37% |
| 38 | GO\_METAPHASE\_PLATE\_CONGRESSION |  | 40 | -0.73 | -1.67 | 0.002 | 0.155 | 0.938 | 1585 | tags=43%, list=7%, signal=46% |
| 39 | GO\_HISTONE\_MRNA\_METABOLIC\_PROCESS |  | 28 | -0.57 | -1.67 | 0.024 | 0.158 | 0.946 | 3700 | tags=46%, list=17%, signal=56% |
| 40 | GO\_TRANSLESION\_SYNTHESIS |  | 37 | -0.63 | -1.66 | 0.006 | 0.162 | 0.954 | 2997 | tags=41%, list=14%, signal=47% |
| 41 | GO\_ACTIVATION\_OF\_ANAPHASE\_PROMOTING\_COMPLEX\_ACTIVITY |  | 15 | -0.78 | -1.66 | 0.006 | 0.165 | 0.961 | 1796 | tags=47%, list=8%, signal=51% |
| 42 | GO\_REGULATION\_OF\_EXIT\_FROM\_MITOSIS |  | 15 | -0.80 | -1.66 | 0.002 | 0.170 | 0.965 | 1857 | tags=47%, list=9%, signal=51% |
| 43 | GO\_MITOTIC\_NUCLEAR\_DIVISION |  | 334 | -0.58 | -1.65 | 0.014 | 0.177 | 0.968 | 3598 | tags=40%, list=17%, signal=47% |
| 44 | GO\_ORGANELLE\_FISSION |  | 452 | -0.55 | -1.65 | 0.010 | 0.174 | 0.968 | 3257 | tags=35%, list=15%, signal=41% |
| 45 | GO\_CELL\_DIVISION |  | 422 | -0.53 | -1.65 | 0.010 | 0.177 | 0.973 | 3654 | tags=38%, list=17%, signal=45% |
| 46 | GO\_PROTEIN\_MONOUBIQUITINATION |  | 50 | -0.49 | -1.64 | 0.019 | 0.175 | 0.975 | 4385 | tags=36%, list=20%, signal=45% |
| 47 | GO\_NUCLEAR\_TRANSCRIBED\_MRNA\_CATABOLIC\_PROCESS\_EXONUCLEOLYTIC |  | 30 | -0.58 | -1.64 | 0.022 | 0.174 | 0.977 | 3233 | tags=47%, list=15%, signal=55% |
| 48 | GO\_NEGATIVE\_REGULATION\_OF\_NUCLEAR\_DIVISION |  | 45 | -0.63 | -1.64 | 0.010 | 0.173 | 0.978 | 1907 | tags=40%, list=9%, signal=44% |
| 49 | GO\_CYTOSKELETON\_DEPENDENT\_CYTOKINESIS |  | 38 | -0.65 | -1.64 | 0.008 | 0.169 | 0.978 | 2354 | tags=37%, list=11%, signal=41% |
| 50 | GO\_CHROMOSOME\_LOCALIZATION |  | 56 | -0.66 | -1.64 | 0.006 | 0.178 | 0.981 | 1585 | tags=36%, list=7%, signal=38% |
| 51 | GO\_PHOTOTRANSDUCTION |  | 39 | -0.51 | -1.63 | 0.014 | 0.191 | 0.984 | 3104 | tags=23%, list=14%, signal=27% |
| 52 | GO\_RIBOSOMAL\_LARGE\_SUBUNIT\_BIOGENESIS |  | 44 | -0.57 | -1.63 | 0.037 | 0.189 | 0.985 | 3283 | tags=39%, list=15%, signal=45% |
| 53 | GO\_MITOTIC\_SPINDLE\_ORGANIZATION |  | 62 | -0.63 | -1.63 | 0.024 | 0.187 | 0.986 | 1778 | tags=32%, list=8%, signal=35% |
| 54 | GO\_MEIOTIC\_CELL\_CYCLE\_PROCESS |  | 136 | -0.54 | -1.63 | 0.012 | 0.185 | 0.986 | 2876 | tags=35%, list=13%, signal=40% |
| 55 | GO\_TRANSCRIPTION\_FROM\_RNA\_POLYMERASE\_I\_PROMOTER |  | 34 | -0.60 | -1.62 | 0.025 | 0.186 | 0.986 | 5807 | tags=68%, list=27%, signal=92% |
| 56 | GO\_NUCLEAR\_CHROMOSOME\_SEGREGATION |  | 205 | -0.62 | -1.62 | 0.026 | 0.185 | 0.986 | 3107 | tags=40%, list=14%, signal=46% |
| 57 | GO\_CHROMOSOME\_SEGREGATION |  | 248 | -0.61 | -1.62 | 0.022 | 0.187 | 0.987 | 3692 | tags=42%, list=17%, signal=49% |
| 58 | GO\_INTERSTRAND\_CROSS\_LINK\_REPAIR |  | 38 | -0.59 | -1.62 | 0.021 | 0.188 | 0.988 | 3094 | tags=34%, list=14%, signal=40% |
| 59 | GO\_NEGATIVE\_REGULATION\_OF\_MITOTIC\_NUCLEAR\_DIVISION |  | 33 | -0.65 | -1.62 | 0.020 | 0.187 | 0.988 | 1602 | tags=39%, list=7%, signal=42% |
| 60 | GO\_BRANCHING\_INVOLVED\_IN\_MAMMARY\_GLAND\_DUCT\_MORPHOGENESIS |  | 19 | -0.67 | -1.62 | 0.018 | 0.184 | 0.988 | 3859 | tags=47%, list=18%, signal=58% |
| 61 | GO\_NEGATIVE\_REGULATION\_OF\_CELL\_DIVISION |  | 59 | -0.63 | -1.62 | 0.008 | 0.181 | 0.988 | 1907 | tags=41%, list=9%, signal=44% |
| 62 | GO\_CELL\_CYCLE\_G1\_S\_PHASE\_TRANSITION |  | 107 | -0.64 | -1.61 | 0.016 | 0.191 | 0.991 | 4253 | tags=56%, list=20%, signal=69% |
| 63 | GO\_PROTEIN\_TARGETING\_TO\_MITOCHONDRION |  | 44 | -0.52 | -1.61 | 0.046 | 0.189 | 0.991 | 5560 | tags=59%, list=26%, signal=79% |
| 64 | GO\_REGULATION\_OF\_TRANSCRIPTION\_ELONGATION\_FROM\_RNA\_POLYMERASE\_II\_PROMOTER |  | 21 | -0.54 | -1.61 | 0.046 | 0.189 | 0.992 | 8071 | tags=67%, list=37%, signal=106% |
| 65 | GO\_TRANSLATIONAL\_ELONGATION |  | 104 | -0.51 | -1.61 | 0.066 | 0.188 | 0.993 | 6094 | tags=65%, list=28%, signal=90% |
| 66 | GO\_CELL\_CYCLE\_CHECKPOINT |  | 182 | -0.55 | -1.60 | 0.025 | 0.192 | 0.995 | 3243 | tags=36%, list=15%, signal=42% |
| 67 | GO\_REGULATION\_OF\_UBIQUITIN\_PROTEIN\_LIGASE\_ACTIVITY |  | 17 | -0.69 | -1.60 | 0.016 | 0.193 | 0.995 | 1796 | tags=29%, list=8%, signal=32% |
| 68 | GO\_NEGATIVE\_REGULATION\_OF\_CHROMOSOME\_SEGREGATION |  | 27 | -0.69 | -1.60 | 0.024 | 0.192 | 0.995 | 1535 | tags=37%, list=7%, signal=40% |
| 69 | GO\_ERROR\_PRONE\_TRANSLESION\_SYNTHESIS |  | 19 | -0.69 | -1.60 | 0.023 | 0.189 | 0.995 | 1535 | tags=37%, list=7%, signal=40% |
| 70 | GO\_RETROGRADE\_VESICLE\_MEDIATED\_TRANSPORT\_GOLGI\_TO\_ER |  | 72 | -0.51 | -1.60 | 0.016 | 0.187 | 0.995 | 4857 | tags=35%, list=22%, signal=45% |
| 71 | GO\_SISTER\_CHROMATID\_SEGREGATION |  | 161 | -0.67 | -1.60 | 0.023 | 0.185 | 0.995 | 1781 | tags=39%, list=8%, signal=42% |
| 72 | GO\_REGULATION\_OF\_PROTEIN\_UBIQUITINATION\_INVOLVED\_IN\_UBIQUITIN\_DEPENDENT\_PROTEIN\_CATABOLIC\_PROCESS |  | 96 | -0.51 | -1.60 | 0.034 | 0.182 | 0.995 | 4186 | tags=44%, list=19%, signal=54% |
| 73 | GO\_MITOTIC\_SISTER\_CHROMATID\_SEGREGATION |  | 86 | -0.68 | -1.60 | 0.026 | 0.180 | 0.995 | 1781 | tags=41%, list=8%, signal=44% |
| 74 | GO\_POSITIVE\_REGULATION\_OF\_CHROMOSOME\_SEGREGATION |  | 23 | -0.67 | -1.60 | 0.022 | 0.178 | 0.995 | 431 | tags=22%, list=2%, signal=22% |
| 75 | GO\_NEGATIVE\_REGULATION\_OF\_CELLULAR\_PROTEIN\_CATABOLIC\_PROCESS |  | 57 | -0.51 | -1.60 | 0.016 | 0.176 | 0.995 | 1587 | tags=23%, list=7%, signal=25% |
| 76 | GO\_MITOCHONDRIAL\_RNA\_METABOLIC\_PROCESS |  | 23 | -0.66 | -1.60 | 0.031 | 0.175 | 0.995 | 4319 | tags=61%, list=20%, signal=76% |
| 77 | GO\_DNA\_REPLICATION\_INDEPENDENT\_NUCLEOSOME\_ORGANIZATION |  | 41 | -0.72 | -1.60 | 0.010 | 0.174 | 0.995 | 3881 | tags=63%, list=18%, signal=77% |
| 78 | GO\_POSTREPLICATION\_REPAIR |  | 48 | -0.59 | -1.60 | 0.019 | 0.172 | 0.995 | 4579 | tags=46%, list=21%, signal=58% |
| 79 | GO\_SISTER\_CHROMATID\_COHESION |  | 100 | -0.71 | -1.59 | 0.012 | 0.179 | 0.998 | 3687 | tags=53%, list=17%, signal=64% |
| 80 | GO\_DNA\_BIOSYNTHETIC\_PROCESS |  | 110 | -0.58 | -1.59 | 0.044 | 0.181 | 0.998 | 3086 | tags=42%, list=14%, signal=48% |
| 81 | GO\_SPINDLE\_CHECKPOINT |  | 23 | -0.78 | -1.59 | 0.010 | 0.186 | 0.999 | 1535 | tags=43%, list=7%, signal=47% |
| 82 | GO\_G2\_DNA\_DAMAGE\_CHECKPOINT |  | 33 | -0.65 | -1.59 | 0.031 | 0.185 | 0.999 | 1914 | tags=33%, list=9%, signal=36% |
| 83 | GO\_SNRNA\_PROCESSING |  | 21 | -0.61 | -1.59 | 0.043 | 0.183 | 0.999 | 2973 | tags=52%, list=14%, signal=61% |
| 84 | GO\_SNRNA\_METABOLIC\_PROCESS |  | 73 | -0.50 | -1.58 | 0.050 | 0.183 | 0.999 | 5304 | tags=45%, list=24%, signal=60% |
| 85 | GO\_TRNA\_METABOLIC\_PROCESS |  | 157 | -0.52 | -1.58 | 0.074 | 0.185 | 0.999 | 5029 | tags=46%, list=23%, signal=60% |
| 86 | GO\_TRNA\_PROCESSING |  | 102 | -0.54 | -1.58 | 0.073 | 0.185 | 0.999 | 4985 | tags=52%, list=23%, signal=67% |
| 87 | GO\_DEOXYRIBOSE\_PHOSPHATE\_CATABOLIC\_PROCESS |  | 20 | -0.57 | -1.58 | 0.021 | 0.184 | 0.999 | 4678 | tags=65%, list=22%, signal=83% |
| 88 | GO\_ATP\_DEPENDENT\_CHROMATIN\_REMODELING |  | 63 | -0.59 | -1.58 | 0.027 | 0.182 | 0.999 | 3774 | tags=41%, list=17%, signal=50% |
| 89 | GO\_MITOTIC\_CYTOKINESIS |  | 30 | -0.64 | -1.58 | 0.022 | 0.182 | 0.999 | 913 | tags=30%, list=4%, signal=31% |
| 90 | GO\_REGULATION\_OF\_CYCLIN\_DEPENDENT\_PROTEIN\_KINASE\_ACTIVITY |  | 90 | -0.52 | -1.58 | 0.010 | 0.181 | 0.999 | 2263 | tags=33%, list=10%, signal=37% |
| 91 | GO\_DNA\_RECOMBINATION |  | 190 | -0.55 | -1.58 | 0.045 | 0.180 | 0.999 | 4586 | tags=45%, list=21%, signal=57% |
| 92 | GO\_NCRNA\_TRANSCRIPTION |  | 79 | -0.48 | -1.58 | 0.038 | 0.180 | 0.999 | 5304 | tags=44%, list=24%, signal=58% |
| 93 | GO\_MITOCHONDRIAL\_FUSION |  | 15 | -0.67 | -1.58 | 0.014 | 0.180 | 0.999 | 4270 | tags=67%, list=20%, signal=83% |
| 94 | GO\_TRANSCRIPTION\_COUPLED\_NUCLEOTIDE\_EXCISION\_REPAIR |  | 71 | -0.50 | -1.58 | 0.058 | 0.180 | 0.999 | 5807 | tags=46%, list=27%, signal=63% |
| 95 | GO\_TRNA\_MODIFICATION |  | 52 | -0.57 | -1.57 | 0.051 | 0.179 | 1.000 | 4966 | tags=58%, list=23%, signal=75% |
| 96 | GO\_REGULATION\_OF\_RHODOPSIN\_MEDIATED\_SIGNALING\_PATHWAY |  | 27 | -0.58 | -1.57 | 0.015 | 0.179 | 1.000 | 2282 | tags=26%, list=10%, signal=29% |
| 97 | GO\_REGULATION\_OF\_CELLULAR\_AMINO\_ACID\_METABOLIC\_PROCESS |  | 63 | -0.48 | -1.57 | 0.052 | 0.189 | 1.000 | 6465 | tags=56%, list=30%, signal=79% |
| 98 | GO\_HISTONE\_EXCHANGE |  | 42 | -0.69 | -1.57 | 0.025 | 0.189 | 1.000 | 3774 | tags=55%, list=17%, signal=66% |
| 99 | GO\_SPINDLE\_ASSEMBLY |  | 66 | -0.60 | -1.57 | 0.027 | 0.188 | 1.000 | 1914 | tags=27%, list=9%, signal=30% |
| 100 | GO\_PYRIMIDINE\_NUCLEOTIDE\_METABOLIC\_PROCESS |  | 40 | -0.50 | -1.56 | 0.016 | 0.188 | 1.000 | 5010 | tags=48%, list=23%, signal=62% |
| 101 | GO\_DNA\_SYNTHESIS\_INVOLVED\_IN\_DNA\_REPAIR |  | 70 | -0.63 | -1.56 | 0.043 | 0.188 | 1.000 | 3269 | tags=46%, list=15%, signal=54% |
| 102 | GO\_POSITIVE\_REGULATION\_OF\_MITOTIC\_NUCLEAR\_DIVISION |  | 46 | -0.58 | -1.56 | 0.012 | 0.192 | 1.000 | 1137 | tags=28%, list=5%, signal=30% |
| 103 | GO\_MITOTIC\_SPINDLE\_ASSEMBLY |  | 38 | -0.65 | -1.56 | 0.033 | 0.197 | 1.000 | 1778 | tags=29%, list=8%, signal=31% |
| 104 | GO\_MEIOSIS\_I |  | 77 | -0.54 | -1.55 | 0.039 | 0.200 | 1.000 | 3986 | tags=39%, list=18%, signal=48% |
| 105 | GO\_OSTEOBLAST\_DEVELOPMENT |  | 17 | -0.70 | -1.55 | 0.012 | 0.201 | 1.000 | 1079 | tags=29%, list=5%, signal=31% |
| 106 | GO\_ERROR\_FREE\_TRANSLESION\_SYNTHESIS |  | 18 | -0.63 | -1.55 | 0.039 | 0.203 | 1.000 | 1430 | tags=28%, list=7%, signal=30% |
| 107 | GO\_REGULATION\_OF\_CENTROSOME\_CYCLE |  | 36 | -0.60 | -1.55 | 0.043 | 0.202 | 1.000 | 4685 | tags=56%, list=22%, signal=71% |
| 108 | GO\_CELL\_CYCLE\_PHASE\_TRANSITION |  | 245 | -0.53 | -1.55 | 0.032 | 0.201 | 1.000 | 3314 | tags=38%, list=15%, signal=44% |
| 109 | GO\_PROTEIN\_UBIQUITINATION\_INVOLVED\_IN\_UBIQUITIN\_DEPENDENT\_PROTEIN\_CATABOLIC\_PROCESS |  | 127 | -0.44 | -1.55 | 0.014 | 0.200 | 1.000 | 1887 | tags=22%, list=9%, signal=24% |
| 110 | GO\_NUCLEOTIDE\_EXCISION\_REPAIR |  | 108 | -0.46 | -1.55 | 0.045 | 0.201 | 1.000 | 5807 | tags=44%, list=27%, signal=59% |
| 111 | GO\_CENTROSOME\_CYCLE |  | 44 | -0.60 | -1.55 | 0.043 | 0.202 | 1.000 | 1588 | tags=27%, list=7%, signal=29% |
| 112 | GO\_METALLO\_SULFUR\_CLUSTER\_ASSEMBLY |  | 18 | -0.51 | -1.55 | 0.082 | 0.203 | 1.000 | 5212 | tags=44%, list=24%, signal=58% |
| 113 | GO\_PROTEIN\_LOCALIZATION\_TO\_CHROMOSOME |  | 37 | -0.63 | -1.54 | 0.031 | 0.206 | 1.000 | 5422 | tags=59%, list=25%, signal=79% |
| 114 | GO\_DNA\_REPAIR |  | 429 | -0.48 | -1.54 | 0.037 | 0.206 | 1.000 | 4875 | tags=40%, list=22%, signal=51% |
| 115 | GO\_REGULATION\_OF\_TRANSCRIPTION\_INVOLVED\_IN\_G1\_S\_TRANSITION\_OF\_MITOTIC\_CELL\_CYCLE |  | 25 | -0.75 | -1.54 | 0.026 | 0.205 | 1.000 | 692 | tags=44%, list=3%, signal=45% |
| 116 | GO\_REGULATION\_OF\_MITOTIC\_CELL\_CYCLE |  | 442 | -0.43 | -1.54 | 0.016 | 0.211 | 1.000 | 4120 | tags=36%, list=19%, signal=43% |
| 117 | GO\_MEIOTIC\_CELL\_CYCLE |  | 167 | -0.50 | -1.54 | 0.031 | 0.210 | 1.000 | 3090 | tags=33%, list=14%, signal=38% |
| 118 | GO\_MITOTIC\_CELL\_CYCLE\_CHECKPOINT |  | 131 | -0.51 | -1.53 | 0.036 | 0.218 | 1.000 | 4022 | tags=39%, list=18%, signal=47% |
| 119 | GO\_PREASSEMBLY\_OF\_GPI\_ANCHOR\_IN\_ER\_MEMBRANE |  | 15 | -0.61 | -1.53 | 0.049 | 0.216 | 1.000 | 3557 | tags=47%, list=16%, signal=56% |
| 120 | GO\_NCRNA\_3\_END\_PROCESSING |  | 17 | -0.62 | -1.53 | 0.047 | 0.224 | 1.000 | 2280 | tags=41%, list=10%, signal=46% |
| 121 | GO\_POSITIVE\_REGULATION\_OF\_NUCLEAR\_DIVISION |  | 57 | -0.53 | -1.53 | 0.012 | 0.226 | 1.000 | 1137 | tags=25%, list=5%, signal=26% |
| 122 | GO\_PROTEASOMAL\_PROTEIN\_CATABOLIC\_PROCESS |  | 259 | -0.38 | -1.53 | 0.022 | 0.225 | 1.000 | 4410 | tags=31%, list=20%, signal=38% |
| 123 | GO\_NUCLEAR\_TRANSCRIBED\_MRNA\_CATABOLIC\_PROCESS\_DEADENYLATION\_DEPENDENT\_DECAY |  | 52 | -0.46 | -1.52 | 0.055 | 0.224 | 1.000 | 3233 | tags=35%, list=15%, signal=41% |
| 124 | GO\_RNA\_SPLICING\_VIA\_TRANSESTERIFICATION\_REACTIONS |  | 247 | -0.41 | -1.52 | 0.093 | 0.224 | 1.000 | 5932 | tags=43%, list=27%, signal=58% |
| 125 | GO\_ASPARTATE\_FAMILY\_AMINO\_ACID\_BIOSYNTHETIC\_PROCESS |  | 23 | -0.56 | -1.52 | 0.059 | 0.227 | 1.000 | 2194 | tags=30%, list=10%, signal=34% |
| 126 | GO\_REGULATION\_OF\_CELL\_DIVISION |  | 252 | -0.49 | -1.52 | 0.014 | 0.237 | 1.000 | 1976 | tags=27%, list=9%, signal=30% |
| 127 | GO\_TELOMERE\_ORGANIZATION |  | 91 | -0.55 | -1.52 | 0.053 | 0.236 | 1.000 | 5196 | tags=51%, list=24%, signal=66% |
| 128 | GO\_CENTROMERE\_COMPLEX\_ASSEMBLY |  | 37 | -0.72 | -1.51 | 0.032 | 0.236 | 1.000 | 3687 | tags=65%, list=17%, signal=78% |
| 129 | GO\_RNA\_3\_END\_PROCESSING |  | 85 | -0.46 | -1.51 | 0.074 | 0.236 | 1.000 | 4385 | tags=35%, list=20%, signal=44% |
| 130 | GO\_PROTEIN\_LOCALIZATION\_TO\_MITOCHONDRION |  | 60 | -0.43 | -1.51 | 0.027 | 0.235 | 1.000 | 4111 | tags=45%, list=19%, signal=55% |
| 131 | GO\_NUCLEOSIDE\_MONOPHOSPHATE\_BIOSYNTHETIC\_PROCESS |  | 74 | -0.43 | -1.51 | 0.028 | 0.236 | 1.000 | 2981 | tags=32%, list=14%, signal=37% |
| 132 | GO\_POSITIVE\_REGULATION\_OF\_PROTEIN\_MODIFICATION\_BY\_SMALL\_PROTEIN\_CONJUGATION\_OR\_REMOVAL |  | 182 | -0.42 | -1.51 | 0.020 | 0.245 | 1.000 | 4066 | tags=37%, list=19%, signal=46% |
| 133 | GO\_REGULATION\_OF\_CELLULAR\_AMINE\_METABOLIC\_PROCESS |  | 85 | -0.42 | -1.51 | 0.038 | 0.246 | 1.000 | 6465 | tags=49%, list=30%, signal=70% |
| 134 | GO\_LENS\_FIBER\_CELL\_DIFFERENTIATION |  | 25 | -0.60 | -1.51 | 0.026 | 0.246 | 1.000 | 2234 | tags=44%, list=10%, signal=49% |
| 135 | GO\_DNA\_INTEGRITY\_CHECKPOINT |  | 139 | -0.50 | -1.50 | 0.050 | 0.244 | 1.000 | 3233 | tags=32%, list=15%, signal=38% |
| 136 | GO\_METHIONINE\_METABOLIC\_PROCESS |  | 18 | -0.56 | -1.50 | 0.052 | 0.248 | 1.000 | 2079 | tags=33%, list=10%, signal=37% |
| 137 | GO\_DNA\_REPLICATION\_INITIATION |  | 28 | -0.80 | -1.50 | 0.032 | 0.254 | 1.000 | 3232 | tags=75%, list=15%, signal=88% |
| 138 | GO\_MATURATION\_OF\_5\_8S\_RRNA\_FROM\_TRICISTRONIC\_RRNA\_TRANSCRIPT\_SSU\_RRNA\_5\_8S\_RRNA\_LSU\_RRNA\_ |  | 17 | -0.62 | -1.50 | 0.076 | 0.253 | 1.000 | 5133 | tags=71%, list=24%, signal=92% |
| 139 | GO\_RECOMBINATIONAL\_REPAIR |  | 70 | -0.57 | -1.49 | 0.075 | 0.268 | 1.000 | 5196 | tags=49%, list=24%, signal=64% |
| 140 | GO\_MICROTUBULE\_ORGANIZING\_CENTER\_ORGANIZATION |  | 79 | -0.51 | -1.49 | 0.058 | 0.267 | 1.000 | 1794 | tags=24%, list=8%, signal=26% |
| 141 | GO\_MULTI\_ORGANISM\_LOCALIZATION |  | 61 | -0.46 | -1.49 | 0.086 | 0.269 | 1.000 | 4059 | tags=30%, list=19%, signal=36% |
| 142 | GO\_DNA\_DEPENDENT\_DNA\_REPLICATION |  | 90 | -0.65 | -1.49 | 0.060 | 0.267 | 1.000 | 2151 | tags=42%, list=10%, signal=47% |
| 143 | GO\_POSITIVE\_REGULATION\_OF\_CELL\_CYCLE\_PHASE\_TRANSITION |  | 67 | -0.52 | -1.49 | 0.047 | 0.267 | 1.000 | 3243 | tags=34%, list=15%, signal=40% |
| 144 | GO\_NEUTRAL\_AMINO\_ACID\_TRANSPORT |  | 31 | -0.56 | -1.49 | 0.062 | 0.266 | 1.000 | 1590 | tags=23%, list=7%, signal=24% |
| 145 | GO\_POSITIVE\_REGULATION\_OF\_VIRAL\_PROCESS |  | 84 | -0.40 | -1.49 | 0.031 | 0.267 | 1.000 | 6667 | tags=45%, list=31%, signal=65% |
| 146 | GO\_REGULATION\_OF\_CELL\_CYCLE\_CHECKPOINT |  | 26 | -0.57 | -1.49 | 0.053 | 0.267 | 1.000 | 4358 | tags=46%, list=20%, signal=58% |
| 147 | GO\_NUCLEAR\_ENVELOPE\_REASSEMBLY |  | 17 | -0.50 | -1.49 | 0.079 | 0.265 | 1.000 | 4192 | tags=35%, list=19%, signal=44% |
| 148 | GO\_REGULATION\_OF\_PROTEIN\_PHOSPHATASE\_TYPE\_2A\_ACTIVITY |  | 23 | -0.54 | -1.49 | 0.048 | 0.263 | 1.000 | 4744 | tags=48%, list=22%, signal=61% |
| 149 | GO\_TERMINATION\_OF\_RNA\_POLYMERASE\_II\_TRANSCRIPTION |  | 53 | -0.48 | -1.48 | 0.113 | 0.268 | 1.000 | 6814 | tags=55%, list=31%, signal=79% |
| 150 | GO\_NEGATIVE\_REGULATION\_OF\_CELL\_AGING |  | 16 | -0.63 | -1.48 | 0.040 | 0.268 | 1.000 | 2932 | tags=38%, list=13%, signal=43% |
| 151 | GO\_AMINE\_BIOSYNTHETIC\_PROCESS |  | 20 | -0.53 | -1.48 | 0.079 | 0.274 | 1.000 | 2806 | tags=30%, list=13%, signal=34% |
| 152 | GO\_POSITIVE\_REGULATION\_OF\_CELL\_CYCLE |  | 311 | -0.42 | -1.48 | 0.014 | 0.274 | 1.000 | 3243 | tags=27%, list=15%, signal=31% |
| 153 | GO\_REGULATION\_OF\_CYTOKINESIS |  | 58 | -0.51 | -1.48 | 0.060 | 0.273 | 1.000 | 6404 | tags=47%, list=29%, signal=66% |
| 154 | GO\_RRNA\_MODIFICATION |  | 21 | -0.54 | -1.48 | 0.106 | 0.274 | 1.000 | 7994 | tags=71%, list=37%, signal=113% |
| 155 | GO\_DOUBLE\_STRAND\_BREAK\_REPAIR |  | 148 | -0.51 | -1.47 | 0.079 | 0.281 | 1.000 | 3207 | tags=32%, list=15%, signal=38% |
| 156 | GO\_POSITIVE\_REGULATION\_OF\_MITOTIC\_CELL\_CYCLE |  | 118 | -0.48 | -1.47 | 0.029 | 0.282 | 1.000 | 3086 | tags=31%, list=14%, signal=35% |
| 157 | GO\_NUCLEUS\_ORGANIZATION |  | 127 | -0.38 | -1.47 | 0.044 | 0.281 | 1.000 | 4192 | tags=33%, list=19%, signal=41% |
| 158 | GO\_REGULATION\_OF\_CELL\_CYCLE\_PHASE\_TRANSITION |  | 307 | -0.42 | -1.47 | 0.045 | 0.283 | 1.000 | 4120 | tags=35%, list=19%, signal=42% |
| 159 | GO\_NUCLEOTIDE\_EXCISION\_REPAIR\_DNA\_GAP\_FILLING |  | 24 | -0.59 | -1.47 | 0.098 | 0.285 | 1.000 | 5395 | tags=50%, list=25%, signal=66% |
| 160 | GO\_REGULATION\_OF\_MICROTUBULE\_POLYMERIZATION\_OR\_DEPOLYMERIZATION |  | 162 | -0.47 | -1.47 | 0.045 | 0.288 | 1.000 | 2180 | tags=23%, list=10%, signal=26% |
| 161 | GO\_POSITIVE\_REGULATION\_OF\_CELL\_CYCLE\_PROCESS |  | 231 | -0.44 | -1.46 | 0.038 | 0.287 | 1.000 | 1796 | tags=21%, list=8%, signal=22% |
| 162 | GO\_REGULATION\_OF\_PROTEIN\_MODIFICATION\_BY\_SMALL\_PROTEIN\_CONJUGATION\_OR\_REMOVAL |  | 258 | -0.38 | -1.46 | 0.028 | 0.286 | 1.000 | 4186 | tags=34%, list=19%, signal=42% |
| 163 | GO\_REGULATION\_OF\_MICROTUBULE\_BASED\_PROCESS |  | 220 | -0.44 | -1.46 | 0.053 | 0.297 | 1.000 | 4434 | tags=34%, list=20%, signal=42% |
| 164 | GO\_DNA\_STRAND\_ELONGATION |  | 30 | -0.70 | -1.45 | 0.081 | 0.306 | 1.000 | 2997 | tags=57%, list=14%, signal=66% |
| 165 | GO\_RNA\_SECONDARY\_STRUCTURE\_UNWINDING |  | 37 | -0.50 | -1.45 | 0.107 | 0.309 | 1.000 | 6108 | tags=57%, list=28%, signal=79% |
| 166 | GO\_MITOCHONDRIAL\_TRANSPORT |  | 156 | -0.37 | -1.45 | 0.042 | 0.313 | 1.000 | 4239 | tags=37%, list=19%, signal=46% |
| 167 | GO\_BASE\_EXCISION\_REPAIR |  | 38 | -0.60 | -1.45 | 0.067 | 0.315 | 1.000 | 4926 | tags=50%, list=23%, signal=65% |
| 168 | GO\_NUCLEOSIDE\_SALVAGE |  | 15 | -0.65 | -1.45 | 0.045 | 0.314 | 1.000 | 2073 | tags=47%, list=10%, signal=52% |
| 169 | GO\_TELOMERE\_MAINTENANCE\_VIA\_RECOMBINATION |  | 32 | -0.68 | -1.45 | 0.075 | 0.314 | 1.000 | 1760 | tags=47%, list=8%, signal=51% |
| 170 | GO\_REGULATION\_OF\_CENTROSOME\_DUPLICATION |  | 29 | -0.52 | -1.45 | 0.085 | 0.314 | 1.000 | 4685 | tags=48%, list=22%, signal=61% |
| 171 | GO\_MITOTIC\_DNA\_INTEGRITY\_CHECKPOINT |  | 95 | -0.46 | -1.45 | 0.066 | 0.313 | 1.000 | 4022 | tags=38%, list=18%, signal=46% |
| 172 | GO\_TRNA\_METHYLATION |  | 20 | -0.55 | -1.45 | 0.121 | 0.312 | 1.000 | 4771 | tags=60%, list=22%, signal=77% |
| 173 | GO\_HISTONE\_MONOUBIQUITINATION |  | 23 | -0.46 | -1.45 | 0.086 | 0.311 | 1.000 | 2530 | tags=22%, list=12%, signal=25% |
| 174 | GO\_REGULATION\_OF\_SPINDLE\_ORGANIZATION |  | 17 | -0.67 | -1.44 | 0.067 | 0.312 | 1.000 | 2737 | tags=35%, list=13%, signal=40% |
| 175 | GO\_REGULATION\_OF\_EXOSOMAL\_SECRETION |  | 16 | -0.56 | -1.44 | 0.091 | 0.312 | 1.000 | 5079 | tags=44%, list=23%, signal=57% |
| 176 | GO\_TOXIN\_TRANSPORT |  | 34 | -0.48 | -1.44 | 0.047 | 0.311 | 1.000 | 4686 | tags=44%, list=22%, signal=56% |
| 177 | GO\_BLASTOCYST\_DEVELOPMENT |  | 55 | -0.44 | -1.44 | 0.035 | 0.313 | 1.000 | 3678 | tags=40%, list=17%, signal=48% |
| 178 | GO\_PROTEIN\_POLYUBIQUITINATION |  | 230 | -0.37 | -1.44 | 0.046 | 0.313 | 1.000 | 4066 | tags=31%, list=19%, signal=38% |
| 179 | GO\_MRNA\_3\_END\_PROCESSING |  | 63 | -0.44 | -1.44 | 0.128 | 0.312 | 1.000 | 7052 | tags=49%, list=32%, signal=73% |
| 180 | GO\_RIBONUCLEOPROTEIN\_COMPLEX\_LOCALIZATION |  | 105 | -0.44 | -1.44 | 0.150 | 0.313 | 1.000 | 4464 | tags=39%, list=21%, signal=49% |
| 181 | GO\_POSITIVE\_REGULATION\_OF\_CYTOKINESIS |  | 31 | -0.57 | -1.44 | 0.099 | 0.312 | 1.000 | 2504 | tags=23%, list=12%, signal=25% |
| 182 | GO\_MATERNAL\_PLACENTA\_DEVELOPMENT |  | 30 | -0.54 | -1.44 | 0.044 | 0.311 | 1.000 | 2683 | tags=43%, list=12%, signal=49% |
| 183 | GO\_NEGATIVE\_REGULATION\_OF\_GENE\_EXPRESSION\_EPIGENETIC |  | 84 | -0.46 | -1.44 | 0.089 | 0.312 | 1.000 | 3026 | tags=27%, list=14%, signal=32% |
| 184 | GO\_NEGATIVE\_REGULATION\_OF\_CHROMOSOME\_ORGANIZATION |  | 93 | -0.43 | -1.43 | 0.081 | 0.322 | 1.000 | 3915 | tags=29%, list=18%, signal=35% |
| 185 | GO\_KETONE\_BIOSYNTHETIC\_PROCESS |  | 23 | -0.57 | -1.43 | 0.089 | 0.324 | 1.000 | 6109 | tags=57%, list=28%, signal=79% |
| 186 | GO\_NEGATIVE\_REGULATION\_OF\_DNA\_REPLICATION |  | 53 | -0.48 | -1.43 | 0.084 | 0.324 | 1.000 | 5175 | tags=43%, list=24%, signal=57% |
| 187 | GO\_NUCLEOSIDE\_TRIPHOSPHATE\_BIOSYNTHETIC\_PROCESS |  | 52 | -0.41 | -1.43 | 0.061 | 0.323 | 1.000 | 3196 | tags=25%, list=15%, signal=29% |
| 188 | GO\_ISOTYPE\_SWITCHING |  | 16 | -0.57 | -1.43 | 0.091 | 0.323 | 1.000 | 2650 | tags=38%, list=12%, signal=43% |
| 189 | GO\_MITOTIC\_RECOMBINATION |  | 41 | -0.64 | -1.42 | 0.103 | 0.333 | 1.000 | 4368 | tags=61%, list=20%, signal=76% |
| 190 | GO\_DNA\_STRAND\_ELONGATION\_INVOLVED\_IN\_DNA\_REPLICATION |  | 25 | -0.72 | -1.42 | 0.083 | 0.335 | 1.000 | 2151 | tags=56%, list=10%, signal=62% |
| 191 | GO\_\_DE\_NOVO\_PROTEIN\_FOLDING |  | 15 | -0.55 | -1.42 | 0.088 | 0.338 | 1.000 | 5466 | tags=73%, list=25%, signal=98% |
| 192 | GO\_CLEAVAGE\_INVOLVED\_IN\_RRNA\_PROCESSING |  | 17 | -0.60 | -1.42 | 0.106 | 0.337 | 1.000 | 3608 | tags=53%, list=17%, signal=63% |
| 193 | GO\_POSITIVE\_REGULATION\_OF\_NEURON\_APOPTOTIC\_PROCESS |  | 45 | -0.46 | -1.42 | 0.062 | 0.335 | 1.000 | 4022 | tags=33%, list=18%, signal=41% |
| 194 | GO\_PYRIMIDINE\_NUCLEOTIDE\_BIOSYNTHETIC\_PROCESS |  | 26 | -0.51 | -1.42 | 0.067 | 0.340 | 1.000 | 3584 | tags=38%, list=16%, signal=46% |
| 195 | GO\_RNA\_SPLICING |  | 331 | -0.36 | -1.42 | 0.132 | 0.343 | 1.000 | 5932 | tags=39%, list=27%, signal=53% |
| 196 | GO\_REGULATION\_OF\_CELL\_AGING |  | 32 | -0.53 | -1.42 | 0.081 | 0.343 | 1.000 | 2932 | tags=38%, list=13%, signal=43% |
| 197 | GO\_REGULATION\_OF\_DNA\_DEPENDENT\_DNA\_REPLICATION |  | 40 | -0.61 | -1.42 | 0.075 | 0.343 | 1.000 | 2886 | tags=43%, list=13%, signal=49% |
| 198 | GO\_REGULATION\_OF\_SIGNAL\_TRANSDUCTION\_BY\_P53\_CLASS\_MEDIATOR |  | 158 | -0.44 | -1.41 | 0.076 | 0.341 | 1.000 | 3287 | tags=32%, list=15%, signal=37% |
| 199 | GO\_NEGATIVE\_REGULATION\_OF\_ALCOHOL\_BIOSYNTHETIC\_PROCESS |  | 17 | -0.64 | -1.41 | 0.063 | 0.341 | 1.000 | 2090 | tags=35%, list=10%, signal=39% |
| 200 | GO\_REGULATION\_OF\_ARP2\_3\_COMPLEX\_MEDIATED\_ACTIN\_NUCLEATION |  | 15 | -0.47 | -1.41 | 0.073 | 0.339 | 1.000 | 4836 | tags=40%, list=22%, signal=51% |
| 201 | GO\_NEGATIVE\_REGULATION\_OF\_CELL\_CYCLE\_PROCESS |  | 202 | -0.42 | -1.41 | 0.051 | 0.338 | 1.000 | 3549 | tags=31%, list=16%, signal=37% |
| 202 | GO\_SPLICEOSOMAL\_COMPLEX\_ASSEMBLY |  | 49 | -0.42 | -1.41 | 0.119 | 0.337 | 1.000 | 5932 | tags=41%, list=27%, signal=56% |
| 203 | GO\_MICROTUBULE\_BASED\_MOVEMENT |  | 189 | -0.39 | -1.41 | 0.048 | 0.342 | 1.000 | 2117 | tags=17%, list=10%, signal=19% |
| 204 | GO\_NUCLEOTIDE\_BINDING\_DOMAIN\_LEUCINE\_RICH\_REPEAT\_CONTAINING\_RECEPTOR\_SIGNALING\_PATHWAY |  | 26 | -0.47 | -1.41 | 0.106 | 0.341 | 1.000 | 4941 | tags=38%, list=23%, signal=50% |
| 205 | GO\_REGULATION\_OF\_CHROMOSOME\_ORGANIZATION |  | 261 | -0.38 | -1.41 | 0.055 | 0.346 | 1.000 | 4712 | tags=33%, list=22%, signal=42% |
| 206 | GO\_PTERIDINE\_CONTAINING\_COMPOUND\_BIOSYNTHETIC\_PROCESS |  | 15 | -0.59 | -1.41 | 0.105 | 0.347 | 1.000 | 5057 | tags=53%, list=23%, signal=69% |
| 207 | GO\_CELL\_CYCLE\_G2\_M\_PHASE\_TRANSITION |  | 132 | -0.45 | -1.41 | 0.072 | 0.347 | 1.000 | 3314 | tags=32%, list=15%, signal=37% |
| 208 | GO\_NUCLEAR\_ENVELOPE\_ORGANIZATION |  | 76 | -0.43 | -1.41 | 0.081 | 0.347 | 1.000 | 4192 | tags=38%, list=19%, signal=47% |
| 209 | GO\_MITOCHONDRIAL\_MEMBRANE\_ORGANIZATION |  | 86 | -0.39 | -1.41 | 0.050 | 0.346 | 1.000 | 4134 | tags=36%, list=19%, signal=44% |
| 210 | GO\_NUCLEAR\_EXPORT |  | 130 | -0.41 | -1.41 | 0.146 | 0.345 | 1.000 | 5497 | tags=42%, list=25%, signal=55% |
| 211 | GO\_LEUKOCYTE\_APOPTOTIC\_PROCESS |  | 21 | -0.58 | -1.40 | 0.073 | 0.346 | 1.000 | 2781 | tags=38%, list=13%, signal=44% |
| 212 | GO\_ANTEROGRADE\_AXONAL\_TRANSPORT |  | 21 | -0.49 | -1.40 | 0.101 | 0.350 | 1.000 | 120 | tags=10%, list=1%, signal=10% |
| 213 | GO\_PROTEIN\_K63\_LINKED\_UBIQUITINATION |  | 35 | -0.45 | -1.40 | 0.068 | 0.349 | 1.000 | 7293 | tags=63%, list=34%, signal=94% |
| 214 | GO\_DNA\_REPLICATION |  | 194 | -0.53 | -1.40 | 0.117 | 0.349 | 1.000 | 4520 | tags=51%, list=21%, signal=63% |
| 215 | GO\_PROTEIN\_TRANSMEMBRANE\_TRANSPORT |  | 45 | -0.44 | -1.40 | 0.091 | 0.349 | 1.000 | 6424 | tags=60%, list=30%, signal=85% |
| 216 | GO\_NUCLEAR\_TRANSPORT |  | 324 | -0.33 | -1.40 | 0.028 | 0.349 | 1.000 | 4700 | tags=36%, list=22%, signal=45% |
| 217 | GO\_MITOTIC\_G2\_M\_TRANSITION\_CHECKPOINT |  | 18 | -0.60 | -1.40 | 0.131 | 0.347 | 1.000 | 1318 | tags=33%, list=6%, signal=35% |
| 218 | GO\_CELL\_SEPARATION\_AFTER\_CYTOKINESIS |  | 16 | -0.54 | -1.40 | 0.107 | 0.348 | 1.000 | 5447 | tags=44%, list=25%, signal=58% |
| 219 | GO\_DETECTION\_OF\_VISIBLE\_LIGHT |  | 41 | -0.44 | -1.40 | 0.079 | 0.349 | 1.000 | 3104 | tags=20%, list=14%, signal=23% |
| 220 | GO\_NEGATIVE\_REGULATION\_OF\_DNA\_DEPENDENT\_DNA\_REPLICATION |  | 16 | -0.61 | -1.40 | 0.129 | 0.350 | 1.000 | 4517 | tags=44%, list=21%, signal=55% |
| 221 | GO\_RECIPROCAL\_DNA\_RECOMBINATION |  | 36 | -0.50 | -1.40 | 0.120 | 0.350 | 1.000 | 3086 | tags=31%, list=14%, signal=36% |
| 222 | GO\_TELOMERE\_CAPPING |  | 23 | -0.56 | -1.40 | 0.120 | 0.350 | 1.000 | 3094 | tags=35%, list=14%, signal=41% |
| 223 | GO\_DECIDUALIZATION |  | 20 | -0.62 | -1.39 | 0.063 | 0.350 | 1.000 | 3656 | tags=65%, list=17%, signal=78% |
| 224 | GO\_RNA\_PHOSPHODIESTER\_BOND\_HYDROLYSIS\_ENDONUCLEOLYTIC |  | 47 | -0.44 | -1.39 | 0.094 | 0.349 | 1.000 | 3283 | tags=40%, list=15%, signal=48% |
| 225 | GO\_POSITIVE\_REGULATION\_OF\_CELL\_DIVISION |  | 120 | -0.47 | -1.39 | 0.043 | 0.347 | 1.000 | 1976 | tags=24%, list=9%, signal=26% |
| 226 | GO\_SIGNAL\_TRANSDUCTION\_IN\_RESPONSE\_TO\_DNA\_DAMAGE |  | 91 | -0.44 | -1.39 | 0.082 | 0.347 | 1.000 | 4022 | tags=36%, list=18%, signal=44% |
| 227 | GO\_GLYCOSYL\_COMPOUND\_BIOSYNTHETIC\_PROCESS |  | 103 | -0.39 | -1.39 | 0.036 | 0.348 | 1.000 | 2804 | tags=26%, list=13%, signal=30% |
| 228 | GO\_RNA\_METHYLATION |  | 45 | -0.48 | -1.39 | 0.141 | 0.348 | 1.000 | 5810 | tags=49%, list=27%, signal=67% |
| 229 | GO\_G1\_DNA\_DAMAGE\_CHECKPOINT |  | 69 | -0.45 | -1.39 | 0.090 | 0.347 | 1.000 | 4022 | tags=36%, list=18%, signal=44% |
| 230 | GO\_SPINDLE\_LOCALIZATION |  | 37 | -0.48 | -1.39 | 0.108 | 0.347 | 1.000 | 2097 | tags=27%, list=10%, signal=30% |
| 231 | GO\_CYTOPLASMIC\_PATTERN\_RECOGNITION\_RECEPTOR\_SIGNALING\_PATHWAY |  | 31 | -0.52 | -1.39 | 0.135 | 0.346 | 1.000 | 4941 | tags=42%, list=23%, signal=54% |
| 232 | GO\_RIBOSOMAL\_LARGE\_SUBUNIT\_ASSEMBLY |  | 22 | -0.52 | -1.39 | 0.143 | 0.346 | 1.000 | 1766 | tags=23%, list=8%, signal=25% |
| 233 | GO\_STRAND\_DISPLACEMENT |  | 26 | -0.68 | -1.39 | 0.116 | 0.348 | 1.000 | 3269 | tags=58%, list=15%, signal=68% |
| 234 | GO\_RNA\_CATABOLIC\_PROCESS |  | 194 | -0.32 | -1.39 | 0.086 | 0.349 | 1.000 | 3233 | tags=20%, list=15%, signal=23% |
| 235 | GO\_REGULATION\_OF\_DNA\_TEMPLATED\_TRANSCRIPTION\_ELONGATION |  | 38 | -0.43 | -1.39 | 0.105 | 0.351 | 1.000 | 3645 | tags=34%, list=17%, signal=41% |
| 236 | GO\_DEOXYRIBONUCLEOSIDE\_TRIPHOSPHATE\_METABOLIC\_PROCESS |  | 16 | -0.60 | -1.39 | 0.120 | 0.351 | 1.000 | 3196 | tags=56%, list=15%, signal=66% |
| 237 | GO\_NEGATIVE\_REGULATION\_OF\_MITOTIC\_CELL\_CYCLE |  | 187 | -0.41 | -1.38 | 0.070 | 0.353 | 1.000 | 2327 | tags=25%, list=11%, signal=28% |
| 238 | GO\_REGULATION\_OF\_VIRAL\_TRANSCRIPTION |  | 56 | -0.41 | -1.38 | 0.094 | 0.356 | 1.000 | 6035 | tags=46%, list=28%, signal=64% |
| 239 | GO\_MEIOTIC\_CHROMOSOME\_SEGREGATION |  | 52 | -0.48 | -1.38 | 0.119 | 0.360 | 1.000 | 2876 | tags=27%, list=13%, signal=31% |
| 240 | GO\_REGULATION\_OF\_KERATINOCYTE\_DIFFERENTIATION |  | 26 | -0.57 | -1.38 | 0.095 | 0.361 | 1.000 | 1017 | tags=19%, list=5%, signal=20% |
| 241 | GO\_RNA\_PHOSPHODIESTER\_BOND\_HYDROLYSIS |  | 99 | -0.40 | -1.38 | 0.070 | 0.366 | 1.000 | 3283 | tags=34%, list=15%, signal=40% |
| 242 | GO\_SIGNAL\_TRANSDUCTION\_IN\_ABSENCE\_OF\_LIGAND |  | 33 | -0.50 | -1.38 | 0.094 | 0.366 | 1.000 | 649 | tags=21%, list=3%, signal=22% |
| 243 | GO\_SOMATIC\_DIVERSIFICATION\_OF\_IMMUNOGLOBULINS |  | 27 | -0.52 | -1.38 | 0.114 | 0.367 | 1.000 | 2650 | tags=30%, list=12%, signal=34% |
| 244 | GO\_RRNA\_CATABOLIC\_PROCESS |  | 16 | -0.55 | -1.38 | 0.129 | 0.366 | 1.000 | 2227 | tags=38%, list=10%, signal=42% |
| 245 | GO\_TRNA\_TRANSPORT |  | 31 | -0.51 | -1.37 | 0.186 | 0.367 | 1.000 | 4059 | tags=42%, list=19%, signal=51% |
| 246 | GO\_POSITIVE\_REGULATION\_OF\_CELL\_CYCLE\_G1\_S\_PHASE\_TRANSITION |  | 28 | -0.52 | -1.37 | 0.081 | 0.368 | 1.000 | 1602 | tags=21%, list=7%, signal=23% |
| 247 | GO\_DNA\_GEOMETRIC\_CHANGE |  | 78 | -0.51 | -1.37 | 0.145 | 0.367 | 1.000 | 5926 | tags=49%, list=27%, signal=67% |
| 248 | GO\_REGULATION\_OF\_DNA\_REPLICATION |  | 153 | -0.43 | -1.37 | 0.073 | 0.367 | 1.000 | 4779 | tags=44%, list=22%, signal=56% |
| 249 | GO\_REGULATION\_OF\_MITOPHAGY |  | 40 | -0.40 | -1.37 | 0.103 | 0.367 | 1.000 | 5309 | tags=43%, list=24%, signal=56% |
| 250 | GO\_REGULATION\_OF\_DNA\_RECOMBINATION |  | 54 | -0.49 | -1.37 | 0.099 | 0.369 | 1.000 | 4564 | tags=43%, list=21%, signal=54% |
| 251 | GO\_REGULATION\_OF\_TRANSCRIPTION\_FROM\_RNA\_POLYMERASE\_II\_PROMOTER\_IN\_RESPONSE\_TO\_HYPOXIA |  | 32 | -0.42 | -1.37 | 0.106 | 0.371 | 1.000 | 408 | tags=9%, list=2%, signal=10% |
| 252 | GO\_DNA\_CONFORMATION\_CHANGE |  | 235 | -0.48 | -1.37 | 0.111 | 0.375 | 1.000 | 3692 | tags=36%, list=17%, signal=43% |
| 253 | GO\_PROTEIN\_DNA\_COMPLEX\_SUBUNIT\_ORGANIZATION |  | 196 | -0.45 | -1.37 | 0.101 | 0.375 | 1.000 | 4607 | tags=38%, list=21%, signal=47% |
| 254 | GO\_VIRION\_ASSEMBLY |  | 35 | -0.43 | -1.37 | 0.113 | 0.373 | 1.000 | 5754 | tags=34%, list=26%, signal=47% |
| 255 | GO\_NEGATIVE\_REGULATION\_OF\_TELOMERE\_MAINTENANCE\_VIA\_TELOMERE\_LENGTHENING |  | 17 | -0.49 | -1.37 | 0.161 | 0.372 | 1.000 | 7356 | tags=65%, list=34%, signal=98% |
| 256 | GO\_NEGATIVE\_REGULATION\_OF\_EPIDERMIS\_DEVELOPMENT |  | 16 | -0.63 | -1.37 | 0.096 | 0.371 | 1.000 | 1654 | tags=31%, list=8%, signal=34% |
| 257 | GO\_TELOMERE\_MAINTENANCE\_VIA\_TELOMERASE |  | 16 | -0.49 | -1.37 | 0.126 | 0.371 | 1.000 | 6350 | tags=69%, list=29%, signal=97% |
| 258 | GO\_CHROMATIN\_ASSEMBLY\_OR\_DISASSEMBLY |  | 144 | -0.48 | -1.36 | 0.116 | 0.371 | 1.000 | 3881 | tags=35%, list=18%, signal=43% |
| 259 | GO\_POSITIVE\_REGULATION\_OF\_MONOOXYGENASE\_ACTIVITY |  | 27 | -0.53 | -1.36 | 0.058 | 0.370 | 1.000 | 4517 | tags=44%, list=21%, signal=56% |
| 260 | GO\_DSRNA\_FRAGMENTATION |  | 17 | -0.52 | -1.36 | 0.121 | 0.371 | 1.000 | 5173 | tags=53%, list=24%, signal=69% |
| 261 | GO\_MICROTUBULE\_CYTOSKELETON\_ORGANIZATION |  | 314 | -0.40 | -1.36 | 0.071 | 0.370 | 1.000 | 1588 | tags=18%, list=7%, signal=20% |
| 262 | GO\_PYRIMIDINE\_NUCLEOSIDE\_BIOSYNTHETIC\_PROCESS |  | 25 | -0.50 | -1.36 | 0.092 | 0.371 | 1.000 | 3155 | tags=36%, list=15%, signal=42% |
| 263 | GO\_RESPONSE\_TO\_X\_RAY |  | 30 | -0.53 | -1.36 | 0.080 | 0.373 | 1.000 | 2578 | tags=47%, list=12%, signal=53% |
| 264 | GO\_CELL\_DIFFERENTIATION\_INVOLVED\_IN\_EMBRYONIC\_PLACENTA\_DEVELOPMENT |  | 25 | -0.53 | -1.36 | 0.068 | 0.373 | 1.000 | 2595 | tags=36%, list=12%, signal=41% |
| 265 | GO\_POSITIVE\_REGULATION\_OF\_G1\_S\_TRANSITION\_OF\_MITOTIC\_CELL\_CYCLE |  | 24 | -0.51 | -1.36 | 0.087 | 0.374 | 1.000 | 1602 | tags=21%, list=7%, signal=22% |
| 266 | GO\_REGULATION\_OF\_DOUBLE\_STRAND\_BREAK\_REPAIR |  | 32 | -0.53 | -1.36 | 0.129 | 0.374 | 1.000 | 2066 | tags=34%, list=9%, signal=38% |
| 267 | GO\_SYNAPTONEMAL\_COMPLEX\_ORGANIZATION |  | 20 | -0.60 | -1.36 | 0.112 | 0.373 | 1.000 | 4481 | tags=40%, list=21%, signal=50% |
| 268 | GO\_NEGATIVE\_REGULATION\_OF\_CIRCADIAN\_RHYTHM |  | 16 | -0.51 | -1.36 | 0.087 | 0.375 | 1.000 | 1524 | tags=25%, list=7%, signal=27% |
| 269 | GO\_NEGATIVE\_REGULATION\_OF\_HISTONE\_MODIFICATION |  | 34 | -0.42 | -1.35 | 0.112 | 0.379 | 1.000 | 4613 | tags=32%, list=21%, signal=41% |
| 270 | GO\_RNA\_POLYADENYLATION |  | 28 | -0.40 | -1.35 | 0.170 | 0.378 | 1.000 | 7052 | tags=50%, list=32%, signal=74% |
| 271 | GO\_NUCLEIC\_ACID\_PHOSPHODIESTER\_BOND\_HYDROLYSIS |  | 221 | -0.38 | -1.35 | 0.080 | 0.381 | 1.000 | 4607 | tags=37%, list=21%, signal=47% |
| 272 | GO\_DNA\_DEPENDENT\_DNA\_REPLICATION\_MAINTENANCE\_OF\_FIDELITY |  | 19 | -0.58 | -1.35 | 0.149 | 0.380 | 1.000 | 2997 | tags=42%, list=14%, signal=49% |
| 273 | GO\_MICROTUBULE\_BASED\_PROCESS |  | 473 | -0.37 | -1.35 | 0.068 | 0.379 | 1.000 | 2180 | tags=18%, list=10%, signal=20% |
| 274 | GO\_OLIGOSACCHARIDE\_LIPID\_INTERMEDIATE\_BIOSYNTHETIC\_PROCESS |  | 20 | -0.52 | -1.35 | 0.127 | 0.379 | 1.000 | 4630 | tags=65%, list=21%, signal=82% |
| 275 | GO\_RRNA\_METHYLATION |  | 15 | -0.53 | -1.35 | 0.193 | 0.379 | 1.000 | 7994 | tags=67%, list=37%, signal=105% |
| 276 | GO\_REGULATION\_OF\_ALCOHOL\_BIOSYNTHETIC\_PROCESS |  | 44 | -0.52 | -1.35 | 0.067 | 0.378 | 1.000 | 2341 | tags=25%, list=11%, signal=28% |
| 277 | GO\_REGULATION\_OF\_TRANSCRIPTION\_FROM\_RNA\_POLYMERASE\_I\_PROMOTER |  | 23 | -0.46 | -1.35 | 0.169 | 0.379 | 1.000 | 3764 | tags=35%, list=17%, signal=42% |
| 278 | GO\_SYNAPTIC\_VESICLE\_RECYCLING |  | 22 | -0.51 | -1.35 | 0.063 | 0.382 | 1.000 | 1122 | tags=18%, list=5%, signal=19% |
| 279 | GO\_PEPTIDYL\_CYSTEINE\_MODIFICATION |  | 20 | -0.53 | -1.35 | 0.085 | 0.385 | 1.000 | 3451 | tags=30%, list=16%, signal=36% |
| 280 | GO\_POSITIVE\_REGULATION\_OF\_CHROMOSOME\_ORGANIZATION |  | 140 | -0.36 | -1.35 | 0.081 | 0.385 | 1.000 | 4779 | tags=34%, list=22%, signal=44% |
| 281 | GO\_NEGATIVE\_REGULATION\_OF\_CELL\_CYCLE\_PHASE\_TRANSITION |  | 139 | -0.40 | -1.35 | 0.086 | 0.385 | 1.000 | 4022 | tags=32%, list=18%, signal=39% |
| 282 | GO\_CHROMOSOME\_CONDENSATION |  | 29 | -0.60 | -1.34 | 0.160 | 0.387 | 1.000 | 1781 | tags=41%, list=8%, signal=45% |
| 283 | GO\_NUCLEOBASE\_BIOSYNTHETIC\_PROCESS |  | 18 | -0.53 | -1.34 | 0.111 | 0.387 | 1.000 | 3584 | tags=56%, list=16%, signal=66% |
| 284 | GO\_GENE\_SILENCING\_BY\_RNA |  | 116 | -0.41 | -1.34 | 0.122 | 0.388 | 1.000 | 5304 | tags=40%, list=24%, signal=52% |
| 285 | GO\_NIK\_NF\_KAPPAB\_SIGNALING |  | 80 | -0.44 | -1.34 | 0.162 | 0.393 | 1.000 | 5341 | tags=48%, list=25%, signal=63% |
| 286 | GO\_LYMPHOCYTE\_APOPTOTIC\_PROCESS |  | 18 | -0.54 | -1.34 | 0.110 | 0.392 | 1.000 | 3747 | tags=44%, list=17%, signal=54% |
| 287 | GO\_DNA\_PACKAGING |  | 159 | -0.49 | -1.34 | 0.135 | 0.391 | 1.000 | 3881 | tags=38%, list=18%, signal=46% |
| 288 | GO\_RNA\_SPLICING\_VIA\_ENDONUCLEOLYTIC\_CLEAVAGE\_AND\_LIGATION |  | 15 | -0.47 | -1.34 | 0.169 | 0.391 | 1.000 | 6292 | tags=60%, list=29%, signal=84% |
| 289 | GO\_AMINO\_ACID\_ACTIVATION |  | 46 | -0.47 | -1.34 | 0.191 | 0.390 | 1.000 | 5417 | tags=39%, list=25%, signal=52% |
| 290 | GO\_INTESTINAL\_EPITHELIAL\_CELL\_DIFFERENTIATION |  | 16 | -0.53 | -1.34 | 0.083 | 0.394 | 1.000 | 2310 | tags=44%, list=11%, signal=49% |
| 291 | GO\_DNA\_REPLICATION\_DEPENDENT\_NUCLEOSOME\_ORGANIZATION |  | 24 | -0.64 | -1.34 | 0.173 | 0.396 | 1.000 | 2964 | tags=42%, list=14%, signal=48% |
| 292 | GO\_PROTEIN\_FOLDING |  | 196 | -0.32 | -1.33 | 0.065 | 0.398 | 1.000 | 5790 | tags=44%, list=27%, signal=59% |
| 293 | GO\_REGULATION\_OF\_GENE\_EXPRESSION\_EPIGENETIC |  | 193 | -0.37 | -1.33 | 0.126 | 0.397 | 1.000 | 5961 | tags=40%, list=27%, signal=54% |
| 294 | GO\_POSITIVE\_REGULATION\_OF\_MITOCHONDRIAL\_OUTER\_MEMBRANE\_PERMEABILIZATION\_INVOLVED\_IN\_APOPTOTIC\_SIGNALING\_PATHWAY |  | 35 | -0.47 | -1.33 | 0.119 | 0.405 | 1.000 | 4109 | tags=40%, list=19%, signal=49% |
| 295 | GO\_RESPONSE\_TO\_IONIZING\_RADIATION |  | 137 | -0.42 | -1.33 | 0.082 | 0.405 | 1.000 | 2781 | tags=33%, list=13%, signal=37% |
| 296 | GO\_NEGATIVE\_REGULATION\_OF\_PROTEIN\_CATABOLIC\_PROCESS |  | 101 | -0.37 | -1.33 | 0.064 | 0.405 | 1.000 | 1833 | tags=18%, list=8%, signal=19% |
| 297 | GO\_RESPONSE\_TO\_UV |  | 119 | -0.40 | -1.33 | 0.065 | 0.405 | 1.000 | 3261 | tags=34%, list=15%, signal=40% |
| 298 | GO\_NUCLEOTIDE\_SUGAR\_BIOSYNTHETIC\_PROCESS |  | 17 | -0.55 | -1.33 | 0.114 | 0.407 | 1.000 | 4024 | tags=53%, list=18%, signal=65% |
| 299 | GO\_REGULATION\_OF\_HAIR\_CYCLE |  | 21 | -0.57 | -1.33 | 0.090 | 0.407 | 1.000 | 2932 | tags=43%, list=13%, signal=49% |
| 300 | GO\_CELLULAR\_RESPONSE\_TO\_LIGHT\_STIMULUS |  | 87 | -0.38 | -1.33 | 0.067 | 0.408 | 1.000 | 3121 | tags=28%, list=14%, signal=32% |
| 301 | GO\_PYRIMIDINE\_NUCLEOSIDE\_TRIPHOSPHATE\_BIOSYNTHETIC\_PROCESS |  | 15 | -0.56 | -1.33 | 0.121 | 0.408 | 1.000 | 1408 | tags=33%, list=6%, signal=36% |
| 302 | GO\_MALE\_MEIOSIS |  | 35 | -0.51 | -1.32 | 0.067 | 0.409 | 1.000 | 2542 | tags=34%, list=12%, signal=39% |
| 303 | GO\_MATERNAL\_PROCESS\_INVOLVED\_IN\_FEMALE\_PREGNANCY |  | 58 | -0.45 | -1.32 | 0.076 | 0.411 | 1.000 | 3656 | tags=38%, list=17%, signal=45% |
| 304 | GO\_SYNAPTIC\_VESICLE\_ENDOCYTOSIS |  | 17 | -0.52 | -1.32 | 0.114 | 0.414 | 1.000 | 972 | tags=18%, list=4%, signal=18% |
| 305 | GO\_NUCLEOBASE\_CONTAINING\_SMALL\_MOLECULE\_INTERCONVERSION |  | 19 | -0.49 | -1.32 | 0.144 | 0.415 | 1.000 | 3345 | tags=37%, list=15%, signal=43% |
| 306 | GO\_REGULATION\_OF\_CELLULAR\_PROTEIN\_CATABOLIC\_PROCESS |  | 256 | -0.33 | -1.32 | 0.090 | 0.417 | 1.000 | 4212 | tags=30%, list=19%, signal=37% |
| 307 | GO\_SOMATIC\_RECOMBINATION\_OF\_IMMUNOGLOBULIN\_GENE\_SEGMENTS |  | 21 | -0.48 | -1.32 | 0.152 | 0.419 | 1.000 | 2650 | tags=29%, list=12%, signal=33% |
| 308 | GO\_PROTEIN\_DEPOLYMERIZATION |  | 23 | -0.57 | -1.32 | 0.164 | 0.419 | 1.000 | 1253 | tags=26%, list=6%, signal=28% |
| 309 | GO\_CENTROSOME\_DUPLICATION |  | 31 | -0.52 | -1.31 | 0.151 | 0.427 | 1.000 | 1588 | tags=23%, list=7%, signal=24% |
| 310 | GO\_NEGATIVE\_REGULATION\_OF\_SIGNAL\_TRANSDUCTION\_BY\_P53\_CLASS\_MEDIATOR |  | 26 | -0.46 | -1.31 | 0.117 | 0.428 | 1.000 | 3235 | tags=38%, list=15%, signal=45% |
| 311 | GO\_AMIDE\_BIOSYNTHETIC\_PROCESS |  | 449 | -0.32 | -1.31 | 0.131 | 0.428 | 1.000 | 5057 | tags=33%, list=23%, signal=42% |
| 312 | GO\_NEGATIVE\_REGULATION\_OF\_LIPID\_BIOSYNTHETIC\_PROCESS |  | 42 | -0.46 | -1.31 | 0.086 | 0.428 | 1.000 | 2090 | tags=21%, list=10%, signal=24% |
| 313 | GO\_GENE\_SILENCING |  | 173 | -0.37 | -1.31 | 0.142 | 0.428 | 1.000 | 5173 | tags=36%, list=24%, signal=47% |
| 314 | GO\_NEGATIVE\_REGULATION\_OF\_MEIOTIC\_CELL\_CYCLE |  | 18 | -0.54 | -1.31 | 0.097 | 0.426 | 1.000 | 2876 | tags=39%, list=13%, signal=45% |
| 315 | GO\_NEGATIVE\_REGULATION\_OF\_SIGNAL\_TRANSDUCTION\_IN\_ABSENCE\_OF\_LIGAND |  | 30 | -0.55 | -1.31 | 0.109 | 0.427 | 1.000 | 3303 | tags=47%, list=15%, signal=55% |
| 316 | GO\_REGULATION\_OF\_DNA\_METABOLIC\_PROCESS |  | 316 | -0.37 | -1.31 | 0.098 | 0.428 | 1.000 | 4779 | tags=38%, list=22%, signal=48% |
| 317 | GO\_PYRIMIDINE\_CONTAINING\_COMPOUND\_BIOSYNTHETIC\_PROCESS |  | 35 | -0.46 | -1.31 | 0.106 | 0.429 | 1.000 | 3584 | tags=37%, list=16%, signal=44% |
| 318 | GO\_FOLIC\_ACID\_CONTAINING\_COMPOUND\_METABOLIC\_PROCESS |  | 26 | -0.47 | -1.31 | 0.162 | 0.428 | 1.000 | 3439 | tags=42%, list=16%, signal=50% |
| 319 | GO\_NCRNA\_CATABOLIC\_PROCESS |  | 21 | -0.51 | -1.31 | 0.149 | 0.428 | 1.000 | 2356 | tags=38%, list=11%, signal=43% |
| 320 | GO\_MITOCHONDRIAL\_TRANSMEMBRANE\_TRANSPORT |  | 49 | -0.41 | -1.31 | 0.184 | 0.427 | 1.000 | 4999 | tags=43%, list=23%, signal=56% |
| 321 | GO\_DETECTION\_OF\_LIGHT\_STIMULUS |  | 55 | -0.38 | -1.31 | 0.118 | 0.429 | 1.000 | 2442 | tags=15%, list=11%, signal=16% |
| 322 | GO\_RRNA\_TRANSCRIPTION |  | 16 | -0.51 | -1.31 | 0.144 | 0.428 | 1.000 | 4892 | tags=50%, list=22%, signal=64% |
| 323 | GO\_NUCLEOBASE\_CONTAINING\_COMPOUND\_TRANSPORT |  | 176 | -0.35 | -1.31 | 0.151 | 0.428 | 1.000 | 4704 | tags=33%, list=22%, signal=42% |
| 324 | GO\_POSITIVE\_REGULATION\_OF\_CELL\_CYCLE\_ARREST |  | 81 | -0.42 | -1.31 | 0.096 | 0.428 | 1.000 | 4022 | tags=36%, list=18%, signal=44% |
| 325 | GO\_REGULATION\_OF\_CYSTEINE\_TYPE\_ENDOPEPTIDASE\_ACTIVITY\_INVOLVED\_IN\_APOPTOTIC\_SIGNALING\_PATHWAY |  | 22 | -0.54 | -1.30 | 0.143 | 0.428 | 1.000 | 3299 | tags=41%, list=15%, signal=48% |
| 326 | GO\_RELEASE\_OF\_CYTOCHROME\_C\_FROM\_MITOCHONDRIA |  | 21 | -0.53 | -1.30 | 0.146 | 0.427 | 1.000 | 4111 | tags=67%, list=19%, signal=82% |
| 327 | GO\_MITOCHONDRION\_MORPHOGENESIS |  | 19 | -0.45 | -1.30 | 0.149 | 0.427 | 1.000 | 4111 | tags=37%, list=19%, signal=45% |
| 328 | GO\_PURINE\_NUCLEOSIDE\_MONOPHOSPHATE\_BIOSYNTHETIC\_PROCESS |  | 55 | -0.39 | -1.30 | 0.129 | 0.434 | 1.000 | 2730 | tags=31%, list=13%, signal=35% |
| 329 | GO\_POSITIVE\_REGULATION\_OF\_EPIDERMIS\_DEVELOPMENT |  | 30 | -0.54 | -1.30 | 0.107 | 0.434 | 1.000 | 3538 | tags=37%, list=16%, signal=44% |
| 330 | GO\_RNA\_LOCALIZATION |  | 162 | -0.37 | -1.30 | 0.180 | 0.439 | 1.000 | 4704 | tags=36%, list=22%, signal=46% |
| 331 | GO\_HISTONE\_UBIQUITINATION |  | 34 | -0.35 | -1.30 | 0.167 | 0.442 | 1.000 | 6758 | tags=38%, list=31%, signal=55% |
| 332 | GO\_POSITIVE\_REGULATION\_OF\_EPIDERMAL\_CELL\_DIFFERENTIATION |  | 18 | -0.53 | -1.29 | 0.112 | 0.445 | 1.000 | 977 | tags=17%, list=4%, signal=17% |
| 333 | GO\_REGULATION\_OF\_EXTRINSIC\_APOPTOTIC\_SIGNALING\_PATHWAY\_IN\_ABSENCE\_OF\_LIGAND |  | 44 | -0.52 | -1.29 | 0.131 | 0.443 | 1.000 | 3362 | tags=43%, list=15%, signal=51% |
| 334 | GO\_POSITIVE\_REGULATION\_OF\_DNA\_METABOLIC\_PROCESS |  | 174 | -0.38 | -1.29 | 0.117 | 0.444 | 1.000 | 4779 | tags=42%, list=22%, signal=53% |
| 335 | GO\_TELOMERE\_MAINTENANCE\_VIA\_TELOMERE\_LENGTHENING |  | 25 | -0.45 | -1.29 | 0.203 | 0.450 | 1.000 | 6350 | tags=56%, list=29%, signal=79% |
| 336 | GO\_MRNA\_PROCESSING |  | 387 | -0.31 | -1.29 | 0.197 | 0.450 | 1.000 | 5932 | tags=37%, list=27%, signal=50% |
| 337 | GO\_NEURON\_APOPTOTIC\_PROCESS |  | 34 | -0.44 | -1.29 | 0.143 | 0.450 | 1.000 | 3856 | tags=32%, list=18%, signal=39% |
| 338 | GO\_CENTROSOME\_LOCALIZATION |  | 17 | -0.52 | -1.29 | 0.191 | 0.449 | 1.000 | 5095 | tags=53%, list=23%, signal=69% |
| 339 | GO\_CEREBELLAR\_CORTEX\_DEVELOPMENT |  | 45 | -0.45 | -1.29 | 0.175 | 0.449 | 1.000 | 3597 | tags=31%, list=17%, signal=37% |
| 340 | GO\_REGULATION\_OF\_DNA\_REPAIR |  | 67 | -0.42 | -1.29 | 0.170 | 0.449 | 1.000 | 5568 | tags=43%, list=26%, signal=58% |
| 341 | GO\_MEMBRANE\_DISASSEMBLY |  | 44 | -0.46 | -1.29 | 0.191 | 0.452 | 1.000 | 4059 | tags=43%, list=19%, signal=53% |
| 342 | GO\_INTRINSIC\_APOPTOTIC\_SIGNALING\_PATHWAY\_IN\_RESPONSE\_TO\_DNA\_DAMAGE |  | 69 | -0.40 | -1.28 | 0.115 | 0.460 | 1.000 | 4732 | tags=41%, list=22%, signal=52% |
| 343 | GO\_POSITIVE\_REGULATION\_OF\_MRNA\_METABOLIC\_PROCESS |  | 39 | -0.36 | -1.28 | 0.174 | 0.462 | 1.000 | 4737 | tags=33%, list=22%, signal=43% |
| 344 | GO\_PLACENTA\_DEVELOPMENT |  | 135 | -0.39 | -1.28 | 0.061 | 0.462 | 1.000 | 2865 | tags=26%, list=13%, signal=30% |
| 345 | GO\_NEGATIVE\_REGULATION\_OF\_HISTONE\_METHYLATION |  | 16 | -0.49 | -1.28 | 0.200 | 0.471 | 1.000 | 2066 | tags=25%, list=9%, signal=28% |
| 346 | GO\_MODULATION\_OF\_TRANSCRIPTION\_IN\_OTHER\_ORGANISM\_INVOLVED\_IN\_SYMBIOTIC\_INTERACTION |  | 21 | -0.47 | -1.28 | 0.174 | 0.474 | 1.000 | 4996 | tags=43%, list=23%, signal=56% |
| 347 | GO\_PROTEIN\_REFOLDING |  | 17 | -0.50 | -1.28 | 0.172 | 0.474 | 1.000 | 6702 | tags=59%, list=31%, signal=85% |
| 348 | GO\_POSITIVE\_REGULATION\_OF\_PROTEIN\_AUTOPHOSPHORYLATION |  | 21 | -0.53 | -1.28 | 0.138 | 0.473 | 1.000 | 1478 | tags=29%, list=7%, signal=31% |
| 349 | GO\_REGULATION\_OF\_CELL\_CYCLE\_ARREST |  | 102 | -0.40 | -1.27 | 0.157 | 0.472 | 1.000 | 4022 | tags=34%, list=18%, signal=42% |
| 350 | GO\_RNA\_MODIFICATION |  | 103 | -0.39 | -1.27 | 0.240 | 0.471 | 1.000 | 5810 | tags=50%, list=27%, signal=67% |
| 351 | GO\_CELLULAR\_RESPONSE\_TO\_HEAT |  | 33 | -0.49 | -1.27 | 0.115 | 0.471 | 1.000 | 4256 | tags=52%, list=20%, signal=64% |
| 352 | GO\_PYRIDINE\_CONTAINING\_COMPOUND\_BIOSYNTHETIC\_PROCESS |  | 18 | -0.54 | -1.27 | 0.147 | 0.477 | 1.000 | 3494 | tags=39%, list=16%, signal=46% |
| 353 | GO\_REGULATION\_OF\_PROTEIN\_CATABOLIC\_PROCESS |  | 370 | -0.31 | -1.27 | 0.082 | 0.481 | 1.000 | 4212 | tags=29%, list=19%, signal=35% |
| 354 | GO\_ESTABLISHMENT\_OF\_PROTEIN\_LOCALIZATION\_TO\_ORGANELLE |  | 318 | -0.27 | -1.27 | 0.100 | 0.484 | 1.000 | 5069 | tags=29%, list=23%, signal=37% |
| 355 | GO\_EMBRYONIC\_AXIS\_SPECIFICATION |  | 32 | -0.45 | -1.27 | 0.159 | 0.483 | 1.000 | 5737 | tags=44%, list=26%, signal=59% |
| 356 | GO\_NUCLEOSIDE\_PHOSPHATE\_BIOSYNTHETIC\_PROCESS |  | 167 | -0.34 | -1.27 | 0.074 | 0.485 | 1.000 | 2981 | tags=28%, list=14%, signal=32% |
| 357 | GO\_PROTEIN\_AUTOUBIQUITINATION |  | 48 | -0.36 | -1.27 | 0.177 | 0.484 | 1.000 | 3261 | tags=21%, list=15%, signal=24% |
| 358 | GO\_REGULATION\_OF\_MITOCHONDRIAL\_OUTER\_MEMBRANE\_PERMEABILIZATION\_INVOLVED\_IN\_APOPTOTIC\_SIGNALING\_PATHWAY |  | 40 | -0.42 | -1.27 | 0.151 | 0.483 | 1.000 | 4109 | tags=38%, list=19%, signal=46% |
| 359 | GO\_CHROMATIN\_SILENCING\_AT\_RDNA |  | 25 | -0.54 | -1.27 | 0.230 | 0.483 | 1.000 | 2977 | tags=36%, list=14%, signal=42% |
| 360 | GO\_SECRETORY\_GRANULE\_ORGANIZATION |  | 24 | -0.44 | -1.27 | 0.186 | 0.482 | 1.000 | 5910 | tags=46%, list=27%, signal=63% |
| 361 | GO\_SOMATIC\_DIVERSIFICATION\_OF\_IMMUNE\_RECEPTORS |  | 39 | -0.44 | -1.26 | 0.207 | 0.483 | 1.000 | 2650 | tags=26%, list=12%, signal=29% |
| 362 | GO\_NEGATIVE\_REGULATION\_OF\_LIPID\_STORAGE |  | 17 | -0.53 | -1.26 | 0.157 | 0.486 | 1.000 | 4384 | tags=41%, list=20%, signal=52% |
| 363 | GO\_POSITIVE\_REGULATION\_OF\_PROTEIN\_DEACETYLATION |  | 18 | -0.49 | -1.26 | 0.177 | 0.487 | 1.000 | 2341 | tags=28%, list=11%, signal=31% |
| 364 | GO\_REGULATION\_OF\_TELOMERE\_MAINTENANCE\_VIA\_TELOMERE\_LENGTHENING |  | 46 | -0.39 | -1.26 | 0.193 | 0.486 | 1.000 | 5037 | tags=43%, list=23%, signal=56% |
| 365 | GO\_STEROID\_CATABOLIC\_PROCESS |  | 24 | -0.50 | -1.26 | 0.160 | 0.486 | 1.000 | 443 | tags=13%, list=2%, signal=13% |
| 366 | GO\_POST\_TRANSLATIONAL\_PROTEIN\_MODIFICATION |  | 34 | -0.39 | -1.26 | 0.140 | 0.487 | 1.000 | 5666 | tags=41%, list=26%, signal=56% |
| 367 | GO\_PROTEIN\_SUMOYLATION |  | 110 | -0.40 | -1.26 | 0.199 | 0.487 | 1.000 | 5395 | tags=43%, list=25%, signal=57% |
| 368 | GO\_REGULATION\_OF\_CELLULAR\_SENESCENCE |  | 26 | -0.49 | -1.26 | 0.186 | 0.490 | 1.000 | 4517 | tags=50%, list=21%, signal=63% |
| 369 | GO\_CARBOHYDRATE\_PHOSPHORYLATION |  | 20 | -0.47 | -1.26 | 0.136 | 0.490 | 1.000 | 2140 | tags=25%, list=10%, signal=28% |
| 370 | GO\_PIGMENT\_BIOSYNTHETIC\_PROCESS |  | 46 | -0.38 | -1.26 | 0.135 | 0.490 | 1.000 | 4590 | tags=39%, list=21%, signal=49% |
| 371 | GO\_NUCLEOSIDE\_TRANSPORT |  | 15 | -0.50 | -1.26 | 0.185 | 0.489 | 1.000 | 1510 | tags=20%, list=7%, signal=21% |
| 372 | GO\_POSITIVE\_REGULATION\_OF\_VASCULAR\_ENDOTHELIAL\_GROWTH\_FACTOR\_RECEPTOR\_SIGNALING\_PATHWAY |  | 15 | -0.55 | -1.26 | 0.176 | 0.487 | 1.000 | 2372 | tags=33%, list=11%, signal=37% |
| 373 | GO\_T\_CELL\_APOPTOTIC\_PROCESS |  | 15 | -0.54 | -1.26 | 0.196 | 0.487 | 1.000 | 3747 | tags=47%, list=17%, signal=56% |
| 374 | GO\_NUCLEOTIDE\_EXCISION\_REPAIR\_DNA\_INCISION |  | 38 | -0.42 | -1.25 | 0.223 | 0.493 | 1.000 | 5725 | tags=39%, list=26%, signal=53% |
| 375 | GO\_NEUTROPHIL\_MEDIATED\_IMMUNITY |  | 21 | -0.58 | -1.25 | 0.187 | 0.493 | 1.000 | 3586 | tags=43%, list=16%, signal=51% |
| 376 | GO\_GUANOSINE\_CONTAINING\_COMPOUND\_METABOLIC\_PROCESS |  | 42 | -0.41 | -1.25 | 0.120 | 0.492 | 1.000 | 1438 | tags=21%, list=7%, signal=23% |
| 377 | GO\_RESPONSE\_TO\_VITAMIN\_E |  | 15 | -0.55 | -1.25 | 0.148 | 0.491 | 1.000 | 2263 | tags=40%, list=10%, signal=45% |
| 378 | GO\_TRANSCRIPTION\_FROM\_RNA\_POLYMERASE\_III\_PROMOTER |  | 38 | -0.40 | -1.25 | 0.185 | 0.490 | 1.000 | 6576 | tags=50%, list=30%, signal=72% |
| 379 | GO\_CENTRIOLE\_ASSEMBLY |  | 18 | -0.54 | -1.25 | 0.203 | 0.492 | 1.000 | 821 | tags=17%, list=4%, signal=17% |
| 380 | GO\_MALE\_MEIOSIS\_I |  | 16 | -0.53 | -1.25 | 0.162 | 0.495 | 1.000 | 2542 | tags=38%, list=12%, signal=42% |
| 381 | GO\_POSITIVE\_REGULATION\_OF\_CELLULAR\_PROTEIN\_CATABOLIC\_PROCESS |  | 182 | -0.33 | -1.25 | 0.152 | 0.496 | 1.000 | 4066 | tags=32%, list=19%, signal=40% |
| 382 | GO\_REGULATION\_OF\_DNA\_METHYLATION |  | 16 | -0.50 | -1.25 | 0.199 | 0.501 | 1.000 | 2349 | tags=31%, list=11%, signal=35% |
| 383 | GO\_MAGNESIUM\_ION\_TRANSMEMBRANE\_TRANSPORT |  | 15 | -0.50 | -1.25 | 0.157 | 0.501 | 1.000 | 4577 | tags=33%, list=21%, signal=42% |
| 384 | GO\_CELLULAR\_RESPONSE\_TO\_EPIDERMAL\_GROWTH\_FACTOR\_STIMULUS |  | 22 | -0.45 | -1.25 | 0.207 | 0.503 | 1.000 | 3368 | tags=32%, list=15%, signal=38% |
| 385 | GO\_REGULATION\_OF\_CELL\_CYCLE\_G1\_S\_PHASE\_TRANSITION |  | 139 | -0.36 | -1.24 | 0.117 | 0.509 | 1.000 | 4022 | tags=31%, list=18%, signal=38% |
| 386 | GO\_CHROMATIN\_SILENCING |  | 68 | -0.42 | -1.24 | 0.250 | 0.510 | 1.000 | 3385 | tags=29%, list=16%, signal=35% |
| 387 | GO\_PYRIMIDINE\_NUCLEOSIDE\_TRIPHOSPHATE\_METABOLIC\_PROCESS |  | 17 | -0.50 | -1.24 | 0.186 | 0.508 | 1.000 | 1408 | tags=29%, list=6%, signal=31% |
| 388 | GO\_NEGATIVE\_REGULATION\_OF\_APOPTOTIC\_SIGNALING\_PATHWAY |  | 184 | -0.35 | -1.24 | 0.072 | 0.508 | 1.000 | 3303 | tags=31%, list=15%, signal=36% |
| 389 | GO\_MRNA\_CIS\_SPLICING\_VIA\_SPLICEOSOME |  | 16 | -0.46 | -1.24 | 0.242 | 0.508 | 1.000 | 5886 | tags=44%, list=27%, signal=60% |
| 390 | GO\_CHROMATIN\_REMODELING |  | 137 | -0.37 | -1.24 | 0.192 | 0.507 | 1.000 | 3774 | tags=31%, list=17%, signal=37% |
| 391 | GO\_REGULATION\_OF\_PROTEASOMAL\_UBIQUITIN\_DEPENDENT\_PROTEIN\_CATABOLIC\_PROCESS |  | 140 | -0.33 | -1.24 | 0.135 | 0.517 | 1.000 | 2017 | tags=19%, list=9%, signal=21% |
| 392 | GO\_MISMATCH\_REPAIR |  | 28 | -0.48 | -1.24 | 0.246 | 0.517 | 1.000 | 5597 | tags=50%, list=26%, signal=67% |
| 393 | GO\_REGULATION\_OF\_TUMOR\_NECROSIS\_FACTOR\_MEDIATED\_SIGNALING\_PATHWAY |  | 45 | -0.39 | -1.23 | 0.167 | 0.523 | 1.000 | 5533 | tags=42%, list=25%, signal=57% |
| 394 | GO\_REGULATION\_OF\_MEMBRANE\_PERMEABILITY |  | 67 | -0.36 | -1.23 | 0.166 | 0.523 | 1.000 | 4365 | tags=33%, list=20%, signal=41% |
| 395 | GO\_NEGATIVE\_REGULATION\_OF\_EXTRINSIC\_APOPTOTIC\_SIGNALING\_PATHWAY |  | 93 | -0.41 | -1.23 | 0.148 | 0.523 | 1.000 | 2967 | tags=33%, list=14%, signal=38% |
| 396 | GO\_RNA\_DEPENDENT\_DNA\_BIOSYNTHETIC\_PROCESS |  | 20 | -0.39 | -1.23 | 0.211 | 0.522 | 1.000 | 6702 | tags=65%, list=31%, signal=94% |
| 397 | GO\_HEME\_BIOSYNTHETIC\_PROCESS |  | 20 | -0.40 | -1.23 | 0.213 | 0.522 | 1.000 | 5309 | tags=40%, list=24%, signal=53% |
| 398 | GO\_SIGNAL\_TRANSDUCTION\_BY\_P53\_CLASS\_MEDIATOR |  | 120 | -0.36 | -1.23 | 0.145 | 0.526 | 1.000 | 4022 | tags=31%, list=18%, signal=38% |
| 399 | GO\_INNER\_MITOCHONDRIAL\_MEMBRANE\_ORGANIZATION |  | 16 | -0.47 | -1.23 | 0.252 | 0.527 | 1.000 | 3729 | tags=44%, list=17%, signal=53% |
| 400 | GO\_REGULATION\_OF\_DOUBLE\_STRAND\_BREAK\_REPAIR\_VIA\_HOMOLOGOUS\_RECOMBINATION |  | 16 | -0.56 | -1.23 | 0.250 | 0.527 | 1.000 | 3165 | tags=44%, list=15%, signal=51% |
| 401 | GO\_RNA\_PHOSPHODIESTER\_BOND\_HYDROLYSIS\_EXONUCLEOLYTIC |  | 32 | -0.41 | -1.23 | 0.204 | 0.526 | 1.000 | 5154 | tags=41%, list=24%, signal=53% |
| 402 | GO\_NUCLEAR\_IMPORT |  | 122 | -0.32 | -1.23 | 0.144 | 0.525 | 1.000 | 5264 | tags=44%, list=24%, signal=58% |
| 403 | GO\_DNA\_TEMPLATED\_TRANSCRIPTION\_INITIATION |  | 192 | -0.30 | -1.23 | 0.143 | 0.529 | 1.000 | 4760 | tags=31%, list=22%, signal=40% |
| 404 | GO\_PTERIDINE\_CONTAINING\_COMPOUND\_METABOLIC\_PROCESS |  | 32 | -0.43 | -1.23 | 0.212 | 0.529 | 1.000 | 3439 | tags=38%, list=16%, signal=44% |
| 405 | GO\_POSITIVE\_REGULATION\_OF\_TELOMERE\_MAINTENANCE |  | 42 | -0.37 | -1.23 | 0.199 | 0.529 | 1.000 | 4779 | tags=40%, list=22%, signal=52% |
| 406 | GO\_REGULATION\_OF\_PROTEASOMAL\_PROTEIN\_CATABOLIC\_PROCESS |  | 171 | -0.31 | -1.22 | 0.134 | 0.528 | 1.000 | 2946 | tags=22%, list=14%, signal=25% |
| 407 | GO\_RESPONSE\_TO\_FOOD |  | 19 | -0.55 | -1.22 | 0.208 | 0.530 | 1.000 | 2595 | tags=37%, list=12%, signal=42% |
| 408 | GO\_MULTI\_ORGANISM\_ORGANELLE\_ORGANIZATION |  | 22 | -0.44 | -1.22 | 0.245 | 0.533 | 1.000 | 5447 | tags=36%, list=25%, signal=48% |
| 409 | GO\_POSITIVE\_REGULATION\_OF\_DNA\_REPLICATION |  | 83 | -0.40 | -1.22 | 0.168 | 0.532 | 1.000 | 3933 | tags=40%, list=18%, signal=48% |
| 410 | GO\_POSITIVE\_REGULATION\_OF\_GENE\_EXPRESSION\_EPIGENETIC |  | 65 | -0.40 | -1.22 | 0.229 | 0.532 | 1.000 | 5854 | tags=49%, list=27%, signal=67% |
| 411 | GO\_REGULATION\_OF\_TELOMERE\_MAINTENANCE |  | 61 | -0.35 | -1.22 | 0.207 | 0.536 | 1.000 | 5037 | tags=38%, list=23%, signal=49% |
| 412 | GO\_PROTEIN\_K48\_LINKED\_UBIQUITINATION |  | 44 | -0.33 | -1.22 | 0.185 | 0.536 | 1.000 | 2405 | tags=18%, list=11%, signal=20% |
| 413 | GO\_REGULATION\_OF\_HAIR\_FOLLICLE\_DEVELOPMENT |  | 15 | -0.58 | -1.22 | 0.194 | 0.535 | 1.000 | 2554 | tags=40%, list=12%, signal=45% |
| 414 | GO\_PSEUDOURIDINE\_SYNTHESIS |  | 16 | -0.50 | -1.22 | 0.258 | 0.535 | 1.000 | 2370 | tags=44%, list=11%, signal=49% |
| 415 | GO\_RESPONSE\_TO\_CADMIUM\_ION |  | 39 | -0.39 | -1.22 | 0.175 | 0.535 | 1.000 | 5560 | tags=49%, list=26%, signal=65% |
| 416 | GO\_GLUCOSE\_6\_PHOSPHATE\_METABOLIC\_PROCESS |  | 21 | -0.48 | -1.22 | 0.247 | 0.534 | 1.000 | 7720 | tags=71%, list=35%, signal=111% |
| 417 | GO\_ESTABLISHMENT\_OF\_MITOTIC\_SPINDLE\_LOCALIZATION |  | 23 | -0.47 | -1.22 | 0.202 | 0.534 | 1.000 | 1794 | tags=26%, list=8%, signal=28% |
| 418 | GO\_L\_AMINO\_ACID\_TRANSPORT |  | 55 | -0.38 | -1.22 | 0.192 | 0.534 | 1.000 | 1817 | tags=18%, list=8%, signal=20% |
| 419 | GO\_REGULATION\_OF\_PEPTIDYL\_SERINE\_PHOSPHORYLATION\_OF\_STAT\_PROTEIN |  | 21 | -0.53 | -1.22 | 0.238 | 0.533 | 1.000 | 1907 | tags=19%, list=9%, signal=21% |
| 420 | GO\_PROTEIN\_LOCALIZATION\_TO\_CENTROSOME |  | 15 | -0.53 | -1.22 | 0.243 | 0.533 | 1.000 | 3257 | tags=33%, list=15%, signal=39% |
| 421 | GO\_GPI\_ANCHOR\_METABOLIC\_PROCESS |  | 32 | -0.43 | -1.21 | 0.202 | 0.539 | 1.000 | 3557 | tags=31%, list=16%, signal=37% |
| 422 | GO\_POSITIVE\_REGULATION\_OF\_VIRAL\_GENOME\_REPLICATION |  | 29 | -0.41 | -1.21 | 0.268 | 0.542 | 1.000 | 6667 | tags=48%, list=31%, signal=70% |
| 423 | GO\_DNA\_LIGATION |  | 16 | -0.51 | -1.21 | 0.255 | 0.546 | 1.000 | 2151 | tags=25%, list=10%, signal=28% |
| 424 | GO\_SYNAPSIS |  | 29 | -0.44 | -1.21 | 0.233 | 0.546 | 1.000 | 7293 | tags=45%, list=34%, signal=67% |
| 425 | GO\_POSITIVE\_REGULATION\_OF\_PROTEIN\_CATABOLIC\_PROCESS |  | 249 | -0.31 | -1.21 | 0.154 | 0.551 | 1.000 | 4075 | tags=30%, list=19%, signal=37% |
| 426 | GO\_REGULATION\_OF\_TRANSLATION\_IN\_RESPONSE\_TO\_STRESS |  | 19 | -0.42 | -1.21 | 0.267 | 0.551 | 1.000 | 4140 | tags=42%, list=19%, signal=52% |
| 427 | GO\_HISTONE\_PHOSPHORYLATION |  | 24 | -0.50 | -1.21 | 0.230 | 0.552 | 1.000 | 2741 | tags=50%, list=13%, signal=57% |
| 428 | GO\_PROTEIN\_DESTABILIZATION |  | 33 | -0.42 | -1.20 | 0.205 | 0.555 | 1.000 | 1587 | tags=21%, list=7%, signal=23% |
| 429 | GO\_OOCYTE\_MATURATION |  | 18 | -0.47 | -1.20 | 0.203 | 0.556 | 1.000 | 2504 | tags=33%, list=12%, signal=38% |
| 430 | GO\_PIGMENT\_GRANULE\_ORGANIZATION |  | 20 | -0.46 | -1.20 | 0.216 | 0.559 | 1.000 | 1713 | tags=25%, list=8%, signal=27% |
| 431 | GO\_CEREBELLAR\_CORTEX\_MORPHOGENESIS |  | 29 | -0.49 | -1.20 | 0.253 | 0.558 | 1.000 | 3597 | tags=38%, list=17%, signal=45% |
| 432 | GO\_NEGATIVE\_REGULATION\_OF\_CYCLIN\_DEPENDENT\_PROTEIN\_KINASE\_ACTIVITY |  | 29 | -0.38 | -1.20 | 0.160 | 0.559 | 1.000 | 1796 | tags=28%, list=8%, signal=30% |
| 433 | GO\_NEGATIVE\_REGULATION\_OF\_TYPE\_I\_INTERFERON\_PRODUCTION |  | 37 | -0.52 | -1.20 | 0.281 | 0.561 | 1.000 | 3541 | tags=35%, list=16%, signal=42% |
| 434 | GO\_HOMOLOGOUS\_CHROMOSOME\_SEGREGATION |  | 39 | -0.44 | -1.20 | 0.240 | 0.560 | 1.000 | 3915 | tags=28%, list=18%, signal=34% |
| 435 | GO\_INTRINSIC\_APOPTOTIC\_SIGNALING\_PATHWAY\_IN\_RESPONSE\_TO\_DNA\_DAMAGE\_BY\_P53\_CLASS\_MEDIATOR |  | 28 | -0.42 | -1.20 | 0.226 | 0.559 | 1.000 | 1753 | tags=21%, list=8%, signal=23% |
| 436 | GO\_NEGATIVE\_REGULATION\_OF\_MRNA\_METABOLIC\_PROCESS |  | 32 | -0.32 | -1.20 | 0.263 | 0.561 | 1.000 | 8590 | tags=47%, list=39%, signal=77% |
| 437 | GO\_REGULATION\_OF\_EPIDERMIS\_DEVELOPMENT |  | 60 | -0.44 | -1.20 | 0.193 | 0.566 | 1.000 | 3538 | tags=28%, list=16%, signal=34% |
| 438 | GO\_COFACTOR\_BIOSYNTHETIC\_PROCESS |  | 149 | -0.35 | -1.20 | 0.196 | 0.566 | 1.000 | 5880 | tags=40%, list=27%, signal=54% |
| 439 | GO\_CELLULAR\_COMPONENT\_DISASSEMBLY |  | 490 | -0.30 | -1.19 | 0.152 | 0.565 | 1.000 | 3892 | tags=30%, list=18%, signal=36% |
| 440 | GO\_ESTABLISHMENT\_OF\_SPINDLE\_ORIENTATION |  | 25 | -0.46 | -1.19 | 0.239 | 0.566 | 1.000 | 1794 | tags=24%, list=8%, signal=26% |
| 441 | GO\_PURINE\_NUCLEOSIDE\_BIOSYNTHETIC\_PROCESS |  | 78 | -0.34 | -1.19 | 0.175 | 0.565 | 1.000 | 2373 | tags=22%, list=11%, signal=24% |
| 442 | GO\_POSITIVE\_REGULATION\_OF\_PROTEOLYSIS |  | 344 | -0.31 | -1.19 | 0.125 | 0.570 | 1.000 | 3423 | tags=28%, list=16%, signal=32% |
| 443 | GO\_ALPHA\_AMINO\_ACID\_BIOSYNTHETIC\_PROCESS |  | 71 | -0.40 | -1.19 | 0.236 | 0.570 | 1.000 | 2657 | tags=27%, list=12%, signal=30% |
| 444 | GO\_CELLULAR\_RESPONSE\_TO\_RADIATION |  | 131 | -0.34 | -1.19 | 0.178 | 0.570 | 1.000 | 2090 | tags=23%, list=10%, signal=25% |
| 445 | GO\_AMINO\_ACID\_TRANSMEMBRANE\_TRANSPORT |  | 61 | -0.39 | -1.19 | 0.184 | 0.573 | 1.000 | 2508 | tags=21%, list=12%, signal=24% |
| 446 | GO\_RESPONSE\_TO\_VITAMIN |  | 97 | -0.40 | -1.19 | 0.133 | 0.573 | 1.000 | 2047 | tags=23%, list=9%, signal=25% |
| 447 | GO\_AZOLE\_TRANSPORT |  | 15 | -0.49 | -1.19 | 0.222 | 0.572 | 1.000 | 3662 | tags=40%, list=17%, signal=48% |
| 448 | GO\_TETRAHYDROFOLATE\_METABOLIC\_PROCESS |  | 18 | -0.46 | -1.19 | 0.247 | 0.572 | 1.000 | 3439 | tags=39%, list=16%, signal=46% |
| 449 | GO\_POSITIVE\_REGULATION\_OF\_LAMELLIPODIUM\_ORGANIZATION |  | 22 | -0.39 | -1.19 | 0.261 | 0.572 | 1.000 | 3855 | tags=27%, list=18%, signal=33% |
| 450 | GO\_NEGATIVE\_REGULATION\_OF\_STEROID\_METABOLIC\_PROCESS |  | 22 | -0.48 | -1.19 | 0.214 | 0.576 | 1.000 | 2990 | tags=23%, list=14%, signal=26% |
| 451 | GO\_MAMMARY\_GLAND\_DUCT\_MORPHOGENESIS |  | 27 | -0.48 | -1.19 | 0.220 | 0.577 | 1.000 | 3859 | tags=37%, list=18%, signal=45% |
| 452 | GO\_CELLULAR\_RESPONSE\_TO\_OXYGEN\_LEVELS |  | 137 | -0.35 | -1.18 | 0.151 | 0.576 | 1.000 | 2967 | tags=23%, list=14%, signal=27% |
| 453 | GO\_MULTI\_ORGANISM\_METABOLIC\_PROCESS |  | 119 | -0.28 | -1.18 | 0.281 | 0.575 | 1.000 | 4546 | tags=18%, list=21%, signal=23% |
| 454 | GO\_REGULATION\_OF\_EPIDERMAL\_CELL\_DIFFERENTIATION |  | 42 | -0.43 | -1.18 | 0.211 | 0.575 | 1.000 | 1654 | tags=17%, list=8%, signal=18% |
| 455 | GO\_CYTOSKELETON\_DEPENDENT\_INTRACELLULAR\_TRANSPORT |  | 107 | -0.33 | -1.18 | 0.196 | 0.574 | 1.000 | 2117 | tags=16%, list=10%, signal=18% |
| 456 | GO\_POSITIVE\_REGULATION\_OF\_INTERLEUKIN\_6\_SECRETION |  | 16 | -0.50 | -1.18 | 0.235 | 0.577 | 1.000 | 128 | tags=13%, list=1%, signal=13% |
| 457 | GO\_ACTIVATION\_OF\_CYSTEINE\_TYPE\_ENDOPEPTIDASE\_ACTIVITY |  | 91 | -0.38 | -1.18 | 0.167 | 0.578 | 1.000 | 3280 | tags=30%, list=15%, signal=35% |
| 458 | GO\_POSITIVE\_REGULATION\_OF\_OXIDOREDUCTASE\_ACTIVITY |  | 44 | -0.41 | -1.18 | 0.158 | 0.576 | 1.000 | 3769 | tags=36%, list=17%, signal=44% |
| 459 | GO\_REGULATION\_OF\_LAMELLIPODIUM\_ASSEMBLY |  | 25 | -0.39 | -1.18 | 0.233 | 0.576 | 1.000 | 3515 | tags=28%, list=16%, signal=33% |
| 460 | GO\_NON\_RECOMBINATIONAL\_REPAIR |  | 61 | -0.40 | -1.18 | 0.255 | 0.578 | 1.000 | 6730 | tags=46%, list=31%, signal=66% |
| 461 | GO\_NEGATIVE\_REGULATION\_OF\_CELL\_CYCLE |  | 405 | -0.31 | -1.18 | 0.132 | 0.577 | 1.000 | 3245 | tags=26%, list=15%, signal=30% |
| 462 | GO\_FEMALE\_GAMETE\_GENERATION |  | 88 | -0.37 | -1.18 | 0.154 | 0.580 | 1.000 | 2147 | tags=22%, list=10%, signal=24% |
| 463 | GO\_RESPONSE\_TO\_LIGHT\_STIMULUS |  | 264 | -0.29 | -1.18 | 0.133 | 0.579 | 1.000 | 3261 | tags=23%, list=15%, signal=26% |
| 464 | GO\_POSITIVE\_REGULATION\_OF\_LAMELLIPODIUM\_ASSEMBLY |  | 15 | -0.43 | -1.18 | 0.267 | 0.579 | 1.000 | 4999 | tags=27%, list=23%, signal=35% |
| 465 | GO\_POSITIVE\_REGULATION\_OF\_TELOMERE\_MAINTENANCE\_VIA\_TELOMERE\_LENGTHENING |  | 30 | -0.38 | -1.18 | 0.261 | 0.578 | 1.000 | 4779 | tags=43%, list=22%, signal=55% |
| 466 | GO\_REGULATION\_OF\_LAMELLIPODIUM\_ORGANIZATION |  | 33 | -0.36 | -1.18 | 0.228 | 0.579 | 1.000 | 3855 | tags=30%, list=18%, signal=37% |
| 467 | GO\_POSITIVE\_REGULATION\_OF\_APOPTOTIC\_SIGNALING\_PATHWAY |  | 163 | -0.34 | -1.18 | 0.147 | 0.581 | 1.000 | 2490 | tags=23%, list=11%, signal=25% |
| 468 | GO\_REGULATION\_OF\_PROTEIN\_EXPORT\_FROM\_NUCLEUS |  | 33 | -0.40 | -1.17 | 0.245 | 0.583 | 1.000 | 1664 | tags=18%, list=8%, signal=20% |
| 469 | GO\_DNA\_DAMAGE\_RESPONSE\_SIGNAL\_TRANSDUCTION\_RESULTING\_IN\_TRANSCRIPTION |  | 15 | -0.57 | -1.17 | 0.248 | 0.583 | 1.000 | 2539 | tags=40%, list=12%, signal=45% |
| 470 | GO\_CHROMOSOME\_ORGANIZATION\_INVOLVED\_IN\_MEIOTIC\_CELL\_CYCLE |  | 41 | -0.41 | -1.17 | 0.281 | 0.584 | 1.000 | 2876 | tags=24%, list=13%, signal=28% |
| 471 | GO\_REGULATION\_OF\_GENE\_SILENCING |  | 47 | -0.39 | -1.17 | 0.285 | 0.585 | 1.000 | 5173 | tags=34%, list=24%, signal=45% |
| 472 | GO\_POSITIVE\_REGULATION\_OF\_HISTONE\_METHYLATION |  | 30 | -0.36 | -1.17 | 0.245 | 0.584 | 1.000 | 575 | tags=10%, list=3%, signal=10% |
| 473 | GO\_PROTEIN\_EXPORT\_FROM\_NUCLEUS |  | 27 | -0.37 | -1.17 | 0.274 | 0.587 | 1.000 | 6960 | tags=48%, list=32%, signal=71% |
| 474 | GO\_ESTABLISHMENT\_OF\_MITOTIC\_SPINDLE\_ORIENTATION |  | 19 | -0.46 | -1.17 | 0.249 | 0.589 | 1.000 | 1794 | tags=26%, list=8%, signal=29% |
| 475 | GO\_NEGATIVE\_REGULATION\_OF\_CHROMATIN\_MODIFICATION |  | 43 | -0.36 | -1.17 | 0.227 | 0.588 | 1.000 | 4613 | tags=30%, list=21%, signal=38% |
| 476 | GO\_L\_ALPHA\_AMINO\_ACID\_TRANSMEMBRANE\_TRANSPORT |  | 31 | -0.41 | -1.17 | 0.221 | 0.593 | 1.000 | 1523 | tags=19%, list=7%, signal=21% |
| 477 | GO\_REGULATION\_OF\_PROTEIN\_INSERTION\_INTO\_MITOCHONDRIAL\_MEMBRANE\_INVOLVED\_IN\_APOPTOTIC\_SIGNALING\_PATHWAY |  | 28 | -0.42 | -1.17 | 0.250 | 0.593 | 1.000 | 4109 | tags=39%, list=19%, signal=48% |
| 478 | GO\_POSITIVE\_REGULATION\_OF\_MITOCHONDRION\_ORGANIZATION |  | 150 | -0.31 | -1.17 | 0.179 | 0.592 | 1.000 | 4206 | tags=32%, list=19%, signal=39% |
| 479 | GO\_POSITIVE\_REGULATION\_OF\_TRANSLATIONAL\_INITIATION |  | 21 | -0.42 | -1.17 | 0.280 | 0.592 | 1.000 | 6885 | tags=52%, list=32%, signal=77% |
| 480 | GO\_POSITIVE\_REGULATION\_OF\_MRNA\_PROCESSING |  | 27 | -0.35 | -1.17 | 0.292 | 0.592 | 1.000 | 4533 | tags=33%, list=21%, signal=42% |
| 481 | GO\_NOTOCHORD\_DEVELOPMENT |  | 17 | -0.55 | -1.17 | 0.236 | 0.592 | 1.000 | 3976 | tags=47%, list=18%, signal=58% |
| 482 | GO\_REGULATION\_OF\_RESPONSE\_TO\_DNA\_DAMAGE\_STIMULUS |  | 130 | -0.33 | -1.17 | 0.214 | 0.591 | 1.000 | 3626 | tags=31%, list=17%, signal=37% |
| 483 | GO\_CYTOPLASMIC\_TRANSLATION |  | 39 | -0.33 | -1.17 | 0.301 | 0.590 | 1.000 | 7386 | tags=49%, list=34%, signal=74% |
| 484 | GO\_REGULATION\_OF\_APOPTOTIC\_SIGNALING\_PATHWAY |  | 339 | -0.31 | -1.16 | 0.144 | 0.593 | 1.000 | 3303 | tags=27%, list=15%, signal=31% |
| 485 | GO\_REGULATION\_OF\_TRANSLATIONAL\_INITIATION |  | 76 | -0.30 | -1.16 | 0.262 | 0.592 | 1.000 | 4280 | tags=29%, list=20%, signal=36% |
| 486 | GO\_NECROTIC\_CELL\_DEATH |  | 27 | -0.47 | -1.16 | 0.254 | 0.594 | 1.000 | 2781 | tags=30%, list=13%, signal=34% |
| 487 | GO\_ORGANELLE\_LOCALIZATION |  | 386 | -0.29 | -1.16 | 0.097 | 0.593 | 1.000 | 1794 | tags=15%, list=8%, signal=16% |
| 488 | GO\_POSITIVE\_REGULATION\_OF\_CYCLIN\_DEPENDENT\_PROTEIN\_KINASE\_ACTIVITY |  | 32 | -0.42 | -1.16 | 0.266 | 0.592 | 1.000 | 2595 | tags=25%, list=12%, signal=28% |
| 489 | GO\_POSITIVE\_REGULATION\_OF\_CHROMATIN\_MODIFICATION |  | 82 | -0.32 | -1.16 | 0.217 | 0.592 | 1.000 | 4613 | tags=33%, list=21%, signal=42% |
| 490 | GO\_DENDRITIC\_SPINE\_ORGANIZATION |  | 17 | -0.49 | -1.16 | 0.280 | 0.598 | 1.000 | 1746 | tags=24%, list=8%, signal=26% |
| 491 | GO\_POSITIVE\_REGULATION\_OF\_CELL\_GROWTH |  | 139 | -0.32 | -1.16 | 0.151 | 0.600 | 1.000 | 3183 | tags=27%, list=15%, signal=31% |
| 492 | GO\_EMBRYO\_IMPLANTATION |  | 35 | -0.46 | -1.16 | 0.236 | 0.602 | 1.000 | 1418 | tags=31%, list=7%, signal=34% |
| 493 | GO\_SOMATIC\_CELL\_DNA\_RECOMBINATION |  | 32 | -0.40 | -1.16 | 0.279 | 0.602 | 1.000 | 2650 | tags=25%, list=12%, signal=28% |
| 494 | GO\_RESPONSE\_TO\_RADIATION |  | 388 | -0.29 | -1.16 | 0.164 | 0.601 | 1.000 | 3450 | tags=26%, list=16%, signal=30% |
| 495 | GO\_BLASTOCYST\_FORMATION |  | 28 | -0.36 | -1.16 | 0.235 | 0.600 | 1.000 | 2506 | tags=29%, list=12%, signal=32% |
| 496 | GO\_POSITIVE\_REGULATION\_OF\_SMOOTH\_MUSCLE\_CONTRACTION |  | 29 | -0.47 | -1.16 | 0.261 | 0.600 | 1.000 | 3769 | tags=31%, list=17%, signal=37% |
| 497 | GO\_PROTEIN\_LOCALIZATION\_TO\_ORGANELLE |  | 498 | -0.25 | -1.16 | 0.187 | 0.599 | 1.000 | 5069 | tags=29%, list=23%, signal=37% |
| 498 | GO\_NONMOTILE\_PRIMARY\_CILIUM\_ASSEMBLY |  | 22 | -0.42 | -1.15 | 0.286 | 0.602 | 1.000 | 1078 | tags=14%, list=5%, signal=14% |
| 499 | GO\_NECROPTOTIC\_PROCESS |  | 20 | -0.49 | -1.15 | 0.280 | 0.601 | 1.000 | 2333 | tags=30%, list=11%, signal=34% |
| 500 | GO\_NEGATIVE\_REGULATION\_OF\_ORGANELLE\_ORGANIZATION |  | 364 | -0.31 | -1.15 | 0.224 | 0.601 | 1.000 | 2338 | tags=20%, list=11%, signal=22% |
| 501 | GO\_PROTEIN\_LOCALIZATION\_TO\_CYTOSKELETON |  | 28 | -0.39 | -1.15 | 0.280 | 0.602 | 1.000 | 5025 | tags=36%, list=23%, signal=46% |
| 502 | GO\_REGULATION\_OF\_ISOTYPE\_SWITCHING |  | 24 | -0.46 | -1.15 | 0.287 | 0.603 | 1.000 | 2703 | tags=29%, list=12%, signal=33% |
| 503 | GO\_POSITIVE\_REGULATION\_OF\_NEUROLOGICAL\_SYSTEM\_PROCESS |  | 20 | -0.46 | -1.15 | 0.290 | 0.602 | 1.000 | 3446 | tags=25%, list=16%, signal=30% |
| 504 | GO\_GLUTAMINE\_FAMILY\_AMINO\_ACID\_BIOSYNTHETIC\_PROCESS |  | 19 | -0.48 | -1.15 | 0.239 | 0.602 | 1.000 | 5970 | tags=47%, list=27%, signal=65% |
| 505 | GO\_ORGANIC\_ACID\_TRANSMEMBRANE\_TRANSPORT |  | 92 | -0.36 | -1.15 | 0.187 | 0.601 | 1.000 | 2772 | tags=25%, list=13%, signal=29% |
| 506 | GO\_NEGATIVE\_REGULATION\_OF\_DNA\_RECOMBINATION |  | 15 | -0.51 | -1.15 | 0.332 | 0.606 | 1.000 | 1769 | tags=33%, list=8%, signal=36% |
| 507 | GO\_RESPONSE\_TO\_VITAMIN\_D |  | 32 | -0.48 | -1.15 | 0.218 | 0.607 | 1.000 | 1418 | tags=28%, list=7%, signal=30% |
| 508 | GO\_NUCLEOSIDE\_PHOSPHATE\_CATABOLIC\_PROCESS |  | 65 | -0.35 | -1.15 | 0.199 | 0.606 | 1.000 | 2383 | tags=23%, list=11%, signal=26% |
| 509 | GO\_RESPONSE\_TO\_GAMMA\_RADIATION |  | 49 | -0.40 | -1.15 | 0.242 | 0.605 | 1.000 | 2781 | tags=31%, list=13%, signal=35% |
| 510 | GO\_NEGATIVE\_REGULATION\_OF\_HISTONE\_ACETYLATION |  | 15 | -0.44 | -1.15 | 0.294 | 0.607 | 1.000 | 4546 | tags=40%, list=21%, signal=51% |
| 511 | GO\_VITAMIN\_TRANSPORT |  | 31 | -0.42 | -1.15 | 0.249 | 0.608 | 1.000 | 4339 | tags=42%, list=20%, signal=52% |
| 512 | GO\_REGULATION\_OF\_CYSTEINE\_TYPE\_ENDOPEPTIDASE\_ACTIVITY |  | 204 | -0.33 | -1.14 | 0.221 | 0.611 | 1.000 | 3299 | tags=27%, list=15%, signal=31% |
| 513 | GO\_UBIQUITIN\_DEPENDENT\_PROTEIN\_CATABOLIC\_PROCESS\_VIA\_THE\_MULTIVESICULAR\_BODY\_SORTING\_PATHWAY |  | 16 | -0.40 | -1.14 | 0.293 | 0.613 | 1.000 | 3947 | tags=38%, list=18%, signal=46% |
| 514 | GO\_PEPTIDYL\_PROLINE\_MODIFICATION |  | 46 | -0.35 | -1.14 | 0.255 | 0.613 | 1.000 | 3520 | tags=26%, list=16%, signal=31% |
| 515 | GO\_REGULATION\_OF\_NUCLEASE\_ACTIVITY |  | 21 | -0.46 | -1.14 | 0.319 | 0.622 | 1.000 | 3627 | tags=33%, list=17%, signal=40% |
| 516 | GO\_REGULATION\_OF\_RNA\_STABILITY |  | 129 | -0.28 | -1.14 | 0.294 | 0.622 | 1.000 | 4946 | tags=33%, list=23%, signal=43% |
| 517 | GO\_HIPPO\_SIGNALING |  | 27 | -0.40 | -1.14 | 0.285 | 0.624 | 1.000 | 4000 | tags=41%, list=18%, signal=50% |
| 518 | GO\_REGULATION\_OF\_DNA\_TEMPLATED\_TRANSCRIPTION\_INITIATION |  | 31 | -0.36 | -1.14 | 0.261 | 0.624 | 1.000 | 4337 | tags=32%, list=20%, signal=40% |
| 519 | GO\_PYRUVATE\_METABOLIC\_PROCESS |  | 59 | -0.36 | -1.14 | 0.243 | 0.624 | 1.000 | 2647 | tags=27%, list=12%, signal=31% |
| 520 | GO\_VESICLE\_DOCKING |  | 54 | -0.35 | -1.14 | 0.247 | 0.623 | 1.000 | 4479 | tags=33%, list=21%, signal=42% |
| 521 | GO\_ZINC\_II\_ION\_TRANSPORT |  | 26 | -0.38 | -1.14 | 0.289 | 0.623 | 1.000 | 7562 | tags=54%, list=35%, signal=82% |
| 522 | GO\_POSITIVE\_REGULATION\_OF\_NEURON\_DEATH |  | 65 | -0.33 | -1.13 | 0.237 | 0.625 | 1.000 | 4702 | tags=32%, list=22%, signal=41% |
| 523 | GO\_HEME\_METABOLIC\_PROCESS |  | 28 | -0.37 | -1.13 | 0.283 | 0.625 | 1.000 | 4590 | tags=36%, list=21%, signal=45% |
| 524 | GO\_POLYOL\_BIOSYNTHETIC\_PROCESS |  | 25 | -0.45 | -1.13 | 0.268 | 0.629 | 1.000 | 3080 | tags=36%, list=14%, signal=42% |
| 525 | GO\_REGULATION\_OF\_STEROID\_BIOSYNTHETIC\_PROCESS |  | 48 | -0.42 | -1.13 | 0.257 | 0.628 | 1.000 | 4050 | tags=27%, list=19%, signal=33% |
| 526 | GO\_RESPONSE\_TO\_GONADOTROPIN |  | 26 | -0.47 | -1.13 | 0.266 | 0.629 | 1.000 | 4050 | tags=46%, list=19%, signal=57% |
| 527 | GO\_INNATE\_IMMUNE\_RESPONSE\_ACTIVATING\_CELL\_SURFACE\_RECEPTOR\_SIGNALING\_PATHWAY |  | 99 | -0.36 | -1.13 | 0.301 | 0.628 | 1.000 | 4066 | tags=34%, list=19%, signal=42% |
| 528 | GO\_PEPTIDYL\_LYSINE\_MODIFICATION |  | 280 | -0.29 | -1.13 | 0.290 | 0.638 | 1.000 | 4568 | tags=29%, list=21%, signal=36% |
| 529 | GO\_POSITIVE\_REGULATION\_OF\_DNA\_TEMPLATED\_TRANSCRIPTION\_INITIATION |  | 24 | -0.38 | -1.13 | 0.280 | 0.639 | 1.000 | 4337 | tags=38%, list=20%, signal=47% |
| 530 | GO\_SULFUR\_AMINO\_ACID\_BIOSYNTHETIC\_PROCESS |  | 19 | -0.44 | -1.12 | 0.298 | 0.642 | 1.000 | 2079 | tags=32%, list=10%, signal=35% |
| 531 | GO\_NEGATIVE\_REGULATION\_OF\_STRESS\_ACTIVATED\_PROTEIN\_KINASE\_SIGNALING\_CASCADE |  | 40 | -0.40 | -1.12 | 0.273 | 0.648 | 1.000 | 1919 | tags=28%, list=9%, signal=30% |
| 532 | GO\_NUCLEOSIDE\_DIPHOSPHATE\_METABOLIC\_PROCESS |  | 75 | -0.33 | -1.12 | 0.238 | 0.648 | 1.000 | 2005 | tags=21%, list=9%, signal=23% |
| 533 | GO\_ORGANELLE\_TRANSPORT\_ALONG\_MICROTUBULE |  | 53 | -0.33 | -1.12 | 0.255 | 0.647 | 1.000 | 2117 | tags=17%, list=10%, signal=19% |
| 534 | GO\_MULTIVESICULAR\_BODY\_ORGANIZATION |  | 29 | -0.38 | -1.12 | 0.337 | 0.646 | 1.000 | 5384 | tags=34%, list=25%, signal=46% |
| 535 | GO\_HYALURONAN\_METABOLIC\_PROCESS |  | 29 | -0.45 | -1.12 | 0.288 | 0.646 | 1.000 | 1606 | tags=28%, list=7%, signal=30% |
| 536 | GO\_REGULATION\_OF\_MEIOTIC\_NUCLEAR\_DIVISION |  | 28 | -0.43 | -1.12 | 0.277 | 0.647 | 1.000 | 1907 | tags=25%, list=9%, signal=27% |
| 537 | GO\_FEMALE\_GENITALIA\_DEVELOPMENT |  | 16 | -0.46 | -1.12 | 0.270 | 0.646 | 1.000 | 3859 | tags=44%, list=18%, signal=53% |
| 538 | GO\_PROTEIN\_IMPORT\_INTO\_NUCLEUS\_TRANSLOCATION |  | 27 | -0.39 | -1.12 | 0.328 | 0.646 | 1.000 | 4538 | tags=44%, list=21%, signal=56% |
| 539 | GO\_RESPONSE\_TO\_CORTICOSTERONE |  | 25 | -0.48 | -1.12 | 0.283 | 0.645 | 1.000 | 3236 | tags=36%, list=15%, signal=42% |
| 540 | GO\_GMP\_METABOLIC\_PROCESS |  | 20 | -0.38 | -1.12 | 0.315 | 0.648 | 1.000 | 1438 | tags=15%, list=7%, signal=16% |
| 541 | GO\_EPHRIN\_RECEPTOR\_SIGNALING\_PATHWAY |  | 84 | -0.34 | -1.12 | 0.258 | 0.647 | 1.000 | 4993 | tags=38%, list=23%, signal=49% |
| 542 | GO\_REGULATION\_OF\_POSTTRANSCRIPTIONAL\_GENE\_SILENCING |  | 18 | -0.35 | -1.12 | 0.311 | 0.648 | 1.000 | 6737 | tags=33%, list=31%, signal=48% |
| 543 | GO\_POSTTRANSCRIPTIONAL\_GENE\_SILENCING |  | 36 | -0.34 | -1.12 | 0.341 | 0.650 | 1.000 | 5173 | tags=39%, list=24%, signal=51% |
| 544 | GO\_CELLULAR\_RESPONSE\_TO\_REACTIVE\_OXYGEN\_SPECIES |  | 102 | -0.33 | -1.12 | 0.256 | 0.650 | 1.000 | 3855 | tags=28%, list=18%, signal=34% |
| 545 | GO\_ESTABLISHMENT\_OF\_LOCALIZATION\_BY\_MOVEMENT\_ALONG\_MICROTUBULE |  | 88 | -0.32 | -1.12 | 0.273 | 0.649 | 1.000 | 5910 | tags=35%, list=27%, signal=48% |
| 546 | GO\_SULFUR\_AMINO\_ACID\_METABOLIC\_PROCESS |  | 39 | -0.39 | -1.11 | 0.276 | 0.649 | 1.000 | 2079 | tags=23%, list=10%, signal=25% |
| 547 | GO\_REGULATION\_OF\_MITOCHONDRION\_ORGANIZATION |  | 196 | -0.29 | -1.11 | 0.230 | 0.648 | 1.000 | 4206 | tags=31%, list=19%, signal=38% |
| 548 | GO\_REGULATION\_OF\_MEIOTIC\_CELL\_CYCLE |  | 38 | -0.39 | -1.11 | 0.246 | 0.647 | 1.000 | 1991 | tags=24%, list=9%, signal=26% |
| 549 | GO\_OOGENESIS |  | 59 | -0.37 | -1.11 | 0.255 | 0.650 | 1.000 | 1410 | tags=19%, list=6%, signal=20% |
| 550 | GO\_RESPONSE\_TO\_EPIDERMAL\_GROWTH\_FACTOR |  | 27 | -0.37 | -1.11 | 0.337 | 0.650 | 1.000 | 4779 | tags=33%, list=22%, signal=43% |
| 551 | GO\_COENZYME\_BIOSYNTHETIC\_PROCESS |  | 110 | -0.35 | -1.11 | 0.292 | 0.652 | 1.000 | 5999 | tags=41%, list=28%, signal=56% |
| 552 | GO\_TETRAPYRROLE\_BIOSYNTHETIC\_PROCESS |  | 27 | -0.36 | -1.11 | 0.336 | 0.656 | 1.000 | 5309 | tags=33%, list=24%, signal=44% |
| 553 | GO\_AMELOGENESIS |  | 19 | -0.47 | -1.11 | 0.306 | 0.657 | 1.000 | 2811 | tags=32%, list=13%, signal=36% |
| 554 | GO\_FOLIC\_ACID\_METABOLIC\_PROCESS |  | 16 | -0.44 | -1.11 | 0.343 | 0.660 | 1.000 | 3439 | tags=50%, list=16%, signal=59% |
| 555 | GO\_POSITIVE\_REGULATION\_OF\_TYROSINE\_PHOSPHORYLATION\_OF\_STAT3\_PROTEIN |  | 37 | -0.44 | -1.11 | 0.265 | 0.659 | 1.000 | 1907 | tags=27%, list=9%, signal=30% |
| 556 | GO\_IN\_UTERO\_EMBRYONIC\_DEVELOPMENT |  | 296 | -0.30 | -1.11 | 0.237 | 0.662 | 1.000 | 2876 | tags=23%, list=13%, signal=26% |
| 557 | GO\_REGULATION\_OF\_TYPE\_I\_INTERFERON\_MEDIATED\_SIGNALING\_PATHWAY |  | 38 | -0.39 | -1.11 | 0.374 | 0.661 | 1.000 | 8022 | tags=47%, list=37%, signal=75% |
| 558 | GO\_REGULATION\_OF\_TYROSINE\_PHOSPHORYLATION\_OF\_STAT3\_PROTEIN |  | 44 | -0.42 | -1.11 | 0.273 | 0.662 | 1.000 | 3970 | tags=41%, list=18%, signal=50% |
| 559 | GO\_NUCLEOTIDE\_PHOSPHORYLATION |  | 53 | -0.36 | -1.11 | 0.290 | 0.661 | 1.000 | 2005 | tags=25%, list=9%, signal=27% |
| 560 | GO\_PROTEIN\_MODIFICATION\_BY\_SMALL\_PROTEIN\_REMOVAL |  | 105 | -0.29 | -1.11 | 0.282 | 0.660 | 1.000 | 5011 | tags=29%, list=23%, signal=37% |
| 561 | GO\_CHROMOSOME\_SEPARATION |  | 17 | -0.45 | -1.10 | 0.394 | 0.660 | 1.000 | 2054 | tags=24%, list=9%, signal=26% |
| 562 | GO\_REGULATION\_OF\_CELLULAR\_RESPONSE\_TO\_HEAT |  | 70 | -0.33 | -1.10 | 0.339 | 0.660 | 1.000 | 5466 | tags=47%, list=25%, signal=63% |
| 563 | GO\_RETINOIC\_ACID\_RECEPTOR\_SIGNALING\_PATHWAY |  | 16 | -0.43 | -1.10 | 0.342 | 0.659 | 1.000 | 1883 | tags=25%, list=9%, signal=27% |
| 564 | GO\_CHROMATIN\_MODIFICATION |  | 492 | -0.27 | -1.10 | 0.275 | 0.659 | 1.000 | 4678 | tags=27%, list=22%, signal=34% |
| 565 | GO\_POSITIVE\_REGULATION\_OF\_DNA\_RECOMBINATION |  | 18 | -0.49 | -1.10 | 0.342 | 0.663 | 1.000 | 1677 | tags=28%, list=8%, signal=30% |
| 566 | GO\_ENDOTHELIAL\_CELL\_DEVELOPMENT |  | 44 | -0.38 | -1.10 | 0.307 | 0.663 | 1.000 | 1239 | tags=16%, list=6%, signal=17% |
| 567 | GO\_ESTABLISHMENT\_OF\_PROTEIN\_LOCALIZATION\_TO\_VACUOLE |  | 29 | -0.32 | -1.10 | 0.343 | 0.662 | 1.000 | 6845 | tags=55%, list=31%, signal=80% |
| 568 | GO\_ESTABLISHMENT\_OF\_ENDOTHELIAL\_BARRIER |  | 29 | -0.38 | -1.10 | 0.312 | 0.661 | 1.000 | 1122 | tags=17%, list=5%, signal=18% |
| 569 | GO\_CYTOCHROME\_COMPLEX\_ASSEMBLY |  | 15 | -0.41 | -1.10 | 0.349 | 0.660 | 1.000 | 4674 | tags=27%, list=21%, signal=34% |
| 570 | GO\_ENDOCYTIC\_RECYCLING |  | 23 | -0.38 | -1.10 | 0.329 | 0.660 | 1.000 | 2992 | tags=30%, list=14%, signal=35% |
| 571 | GO\_CARBOHYDRATE\_TRANSMEMBRANE\_TRANSPORT |  | 23 | -0.47 | -1.10 | 0.301 | 0.659 | 1.000 | 2069 | tags=30%, list=10%, signal=34% |
| 572 | GO\_POSITIVE\_REGULATION\_OF\_CYSTEINE\_TYPE\_ENDOPEPTIDASE\_ACTIVITY\_INVOLVED\_IN\_APOPTOTIC\_SIGNALING\_PATHWAY |  | 17 | -0.47 | -1.10 | 0.349 | 0.659 | 1.000 | 2781 | tags=35%, list=13%, signal=40% |
| 573 | GO\_REGULATION\_OF\_HISTONE\_H3\_K9\_METHYLATION |  | 17 | -0.41 | -1.10 | 0.332 | 0.658 | 1.000 | 575 | tags=12%, list=3%, signal=12% |
| 574 | GO\_REGULATION\_OF\_TRANSCRIPTION\_FROM\_RNA\_POLYMERASE\_III\_PROMOTER |  | 20 | -0.39 | -1.10 | 0.329 | 0.660 | 1.000 | 1287 | tags=20%, list=6%, signal=21% |
| 575 | GO\_ORGANIC\_CATION\_TRANSPORT |  | 18 | -0.47 | -1.10 | 0.298 | 0.663 | 1.000 | 2748 | tags=33%, list=13%, signal=38% |
| 576 | GO\_REGULATION\_OF\_HISTONE\_DEACETYLATION |  | 23 | -0.38 | -1.10 | 0.336 | 0.663 | 1.000 | 2341 | tags=17%, list=11%, signal=19% |
| 577 | GO\_POSITIVE\_REGULATION\_OF\_PEPTIDASE\_ACTIVITY |  | 147 | -0.32 | -1.10 | 0.255 | 0.661 | 1.000 | 2882 | tags=24%, list=13%, signal=28% |
| 578 | GO\_PROTEIN\_IMPORT |  | 144 | -0.28 | -1.10 | 0.264 | 0.663 | 1.000 | 5264 | tags=39%, list=24%, signal=51% |
| 579 | GO\_ORGANIC\_CYCLIC\_COMPOUND\_CATABOLIC\_PROCESS |  | 380 | -0.26 | -1.09 | 0.252 | 0.668 | 1.000 | 3233 | tags=19%, list=15%, signal=22% |
| 580 | GO\_PROTEIN\_COMPLEX\_LOCALIZATION |  | 50 | -0.37 | -1.09 | 0.308 | 0.669 | 1.000 | 2290 | tags=20%, list=11%, signal=22% |
| 581 | GO\_RESPONSE\_TO\_INCREASED\_OXYGEN\_LEVELS |  | 23 | -0.42 | -1.09 | 0.300 | 0.671 | 1.000 | 298 | tags=13%, list=1%, signal=13% |
| 582 | GO\_MALE\_GAMETE\_GENERATION |  | 416 | -0.28 | -1.09 | 0.288 | 0.670 | 1.000 | 2234 | tags=15%, list=10%, signal=17% |
| 583 | GO\_CELLULAR\_RESPONSE\_TO\_UV |  | 65 | -0.34 | -1.09 | 0.278 | 0.669 | 1.000 | 3121 | tags=28%, list=14%, signal=32% |
| 584 | GO\_HISTONE\_H2A\_UBIQUITINATION |  | 16 | -0.35 | -1.09 | 0.345 | 0.671 | 1.000 | 1326 | tags=13%, list=6%, signal=13% |
| 585 | GO\_POSTTRANSCRIPTIONAL\_REGULATION\_OF\_GENE\_EXPRESSION |  | 400 | -0.24 | -1.09 | 0.275 | 0.672 | 1.000 | 4946 | tags=30%, list=23%, signal=38% |
| 586 | GO\_MEMBRANE\_DOCKING |  | 65 | -0.33 | -1.09 | 0.304 | 0.672 | 1.000 | 4145 | tags=31%, list=19%, signal=38% |
| 587 | GO\_POSITIVE\_REGULATION\_OF\_NIK\_NF\_KAPPAB\_SIGNALING |  | 29 | -0.43 | -1.09 | 0.333 | 0.673 | 1.000 | 2490 | tags=31%, list=11%, signal=35% |
| 588 | GO\_MONOSACCHARIDE\_TRANSPORT |  | 53 | -0.36 | -1.09 | 0.283 | 0.679 | 1.000 | 4241 | tags=38%, list=19%, signal=47% |
| 589 | GO\_ATP\_BIOSYNTHETIC\_PROCESS |  | 30 | -0.35 | -1.09 | 0.354 | 0.679 | 1.000 | 4767 | tags=30%, list=22%, signal=38% |
| 590 | GO\_NEGATIVE\_REGULATION\_OF\_CELL\_CYCLE\_G2\_M\_PHASE\_TRANSITION |  | 24 | -0.40 | -1.08 | 0.377 | 0.683 | 1.000 | 1318 | tags=25%, list=6%, signal=27% |
| 591 | GO\_REGULATION\_OF\_PROTEIN\_COMPLEX\_DISASSEMBLY |  | 196 | -0.33 | -1.08 | 0.364 | 0.684 | 1.000 | 2338 | tags=22%, list=11%, signal=25% |
| 592 | GO\_PROTEIN\_LOCALIZATION\_TO\_VACUOLE |  | 41 | -0.31 | -1.08 | 0.358 | 0.687 | 1.000 | 6845 | tags=46%, list=31%, signal=67% |
| 593 | GO\_PYRIMIDINE\_NUCLEOSIDE\_METABOLIC\_PROCESS |  | 41 | -0.36 | -1.08 | 0.292 | 0.689 | 1.000 | 2033 | tags=22%, list=9%, signal=24% |
| 594 | GO\_RESPONSE\_TO\_AMMONIUM\_ION |  | 49 | -0.33 | -1.08 | 0.318 | 0.688 | 1.000 | 486 | tags=10%, list=2%, signal=10% |
| 595 | GO\_POLYOL\_METABOLIC\_PROCESS |  | 92 | -0.31 | -1.08 | 0.276 | 0.687 | 1.000 | 3474 | tags=27%, list=16%, signal=32% |
| 596 | GO\_CELLULAR\_PROCESS\_INVOLVED\_IN\_REPRODUCTION\_IN\_MULTICELLULAR\_ORGANISM |  | 229 | -0.28 | -1.08 | 0.275 | 0.688 | 1.000 | 1576 | tags=14%, list=7%, signal=14% |
| 597 | GO\_PROTEIN\_ACYLATION |  | 141 | -0.26 | -1.08 | 0.305 | 0.689 | 1.000 | 4611 | tags=28%, list=21%, signal=36% |
| 598 | GO\_ECTODERM\_DEVELOPMENT |  | 20 | -0.40 | -1.08 | 0.354 | 0.690 | 1.000 | 3664 | tags=40%, list=17%, signal=48% |
| 599 | GO\_AXO\_DENDRITIC\_TRANSPORT |  | 32 | -0.34 | -1.08 | 0.363 | 0.690 | 1.000 | 120 | tags=6%, list=1%, signal=6% |
| 600 | GO\_REGULATION\_OF\_EXTRINSIC\_APOPTOTIC\_SIGNALING\_PATHWAY |  | 145 | -0.35 | -1.08 | 0.322 | 0.691 | 1.000 | 3362 | tags=32%, list=15%, signal=38% |
| 601 | GO\_HISTONE\_H3\_ACETYLATION |  | 39 | -0.32 | -1.08 | 0.342 | 0.691 | 1.000 | 5516 | tags=38%, list=25%, signal=51% |
| 602 | GO\_NEGATIVE\_REGULATION\_OF\_EXTRINSIC\_APOPTOTIC\_SIGNALING\_PATHWAY\_VIA\_DEATH\_DOMAIN\_RECEPTORS |  | 32 | -0.40 | -1.08 | 0.348 | 0.692 | 1.000 | 1217 | tags=22%, list=6%, signal=23% |
| 603 | GO\_POSITIVE\_REGULATION\_OF\_RELEASE\_OF\_CYTOCHROME\_C\_FROM\_MITOCHONDRIA |  | 26 | -0.42 | -1.08 | 0.357 | 0.691 | 1.000 | 4111 | tags=42%, list=19%, signal=52% |
| 604 | GO\_NEGATIVE\_REGULATION\_OF\_INTRINSIC\_APOPTOTIC\_SIGNALING\_PATHWAY\_BY\_P53\_CLASS\_MEDIATOR |  | 17 | -0.43 | -1.08 | 0.385 | 0.690 | 1.000 | 3235 | tags=41%, list=15%, signal=48% |
| 605 | GO\_MICROTUBULE\_POLYMERIZATION\_OR\_DEPOLYMERIZATION |  | 40 | -0.39 | -1.07 | 0.373 | 0.689 | 1.000 | 1291 | tags=20%, list=6%, signal=21% |
| 606 | GO\_ZYMOGEN\_ACTIVATION |  | 108 | -0.33 | -1.07 | 0.274 | 0.689 | 1.000 | 2882 | tags=25%, list=13%, signal=29% |
| 607 | GO\_POSITIVE\_REGULATION\_OF\_TELOMERE\_CAPPING |  | 15 | -0.41 | -1.07 | 0.389 | 0.688 | 1.000 | 420 | tags=13%, list=2%, signal=14% |
| 608 | GO\_METHYLATION |  | 241 | -0.29 | -1.07 | 0.334 | 0.688 | 1.000 | 4954 | tags=35%, list=23%, signal=45% |
| 609 | GO\_ACTIN\_FILAMENT\_BUNDLE\_ORGANIZATION |  | 46 | -0.36 | -1.07 | 0.343 | 0.690 | 1.000 | 4400 | tags=48%, list=20%, signal=60% |
| 610 | GO\_PIGMENT\_METABOLIC\_PROCESS |  | 56 | -0.32 | -1.07 | 0.337 | 0.690 | 1.000 | 4590 | tags=38%, list=21%, signal=47% |
| 611 | GO\_POSITIVE\_REGULATION\_OF\_NITRIC\_OXIDE\_SYNTHASE\_ACTIVITY |  | 20 | -0.40 | -1.07 | 0.365 | 0.689 | 1.000 | 5159 | tags=45%, list=24%, signal=59% |
| 612 | GO\_REGULATION\_OF\_CGMP\_METABOLIC\_PROCESS |  | 28 | -0.43 | -1.07 | 0.373 | 0.688 | 1.000 | 2067 | tags=18%, list=10%, signal=20% |
| 613 | GO\_NEGATIVE\_REGULATION\_OF\_DNA\_METABOLIC\_PROCESS |  | 104 | -0.30 | -1.07 | 0.343 | 0.688 | 1.000 | 2386 | tags=19%, list=11%, signal=21% |
| 614 | GO\_REGULATION\_OF\_KERATINOCYTE\_PROLIFERATION |  | 26 | -0.43 | -1.07 | 0.356 | 0.689 | 1.000 | 3697 | tags=50%, list=17%, signal=60% |
| 615 | GO\_REGULATION\_OF\_TRANSLATIONAL\_ELONGATION |  | 21 | -0.36 | -1.07 | 0.345 | 0.693 | 1.000 | 2587 | tags=19%, list=12%, signal=22% |
| 616 | GO\_SERINE\_FAMILY\_AMINO\_ACID\_METABOLIC\_PROCESS |  | 39 | -0.40 | -1.07 | 0.348 | 0.696 | 1.000 | 2984 | tags=38%, list=14%, signal=44% |
| 617 | GO\_PYRIMIDINE\_CONTAINING\_COMPOUND\_METABOLIC\_PROCESS |  | 66 | -0.32 | -1.07 | 0.327 | 0.698 | 1.000 | 3662 | tags=32%, list=17%, signal=38% |
| 618 | GO\_PROTEIN\_TARGETING |  | 363 | -0.22 | -1.07 | 0.299 | 0.699 | 1.000 | 4610 | tags=26%, list=21%, signal=32% |
| 619 | GO\_CYTOPLASMIC\_SEQUESTERING\_OF\_PROTEIN |  | 39 | -0.32 | -1.07 | 0.397 | 0.698 | 1.000 | 6182 | tags=38%, list=28%, signal=54% |
| 620 | GO\_MITOCHONDRIAL\_ATP\_SYNTHESIS\_COUPLED\_PROTON\_TRANSPORT |  | 17 | -0.40 | -1.07 | 0.410 | 0.698 | 1.000 | 4239 | tags=24%, list=19%, signal=29% |
| 621 | GO\_REGULATION\_OF\_MRNA\_METABOLIC\_PROCESS |  | 106 | -0.25 | -1.06 | 0.368 | 0.700 | 1.000 | 4737 | tags=27%, list=22%, signal=35% |
| 622 | GO\_REGULATION\_OF\_DEFENSE\_RESPONSE\_TO\_VIRUS\_BY\_HOST |  | 125 | -0.31 | -1.06 | 0.353 | 0.702 | 1.000 | 3130 | tags=27%, list=14%, signal=32% |
| 623 | GO\_DNA\_MODIFICATION |  | 73 | -0.35 | -1.06 | 0.370 | 0.705 | 1.000 | 4875 | tags=42%, list=22%, signal=55% |
| 624 | GO\_GTP\_METABOLIC\_PROCESS |  | 19 | -0.44 | -1.06 | 0.362 | 0.704 | 1.000 | 1408 | tags=26%, list=6%, signal=28% |
| 625 | GO\_REGULATION\_OF\_CHROMATIN\_ORGANIZATION |  | 145 | -0.27 | -1.06 | 0.324 | 0.707 | 1.000 | 4613 | tags=28%, list=21%, signal=35% |
| 626 | GO\_REGULATION\_OF\_NITRIC\_OXIDE\_SYNTHASE\_BIOSYNTHETIC\_PROCESS |  | 18 | -0.46 | -1.06 | 0.374 | 0.711 | 1.000 | 1919 | tags=33%, list=9%, signal=37% |
| 627 | GO\_INTRA\_GOLGI\_VESICLE\_MEDIATED\_TRANSPORT |  | 40 | -0.33 | -1.06 | 0.366 | 0.711 | 1.000 | 6964 | tags=43%, list=32%, signal=62% |
| 628 | GO\_APOPTOTIC\_MITOCHONDRIAL\_CHANGES |  | 55 | -0.34 | -1.06 | 0.343 | 0.712 | 1.000 | 4134 | tags=45%, list=19%, signal=56% |
| 629 | GO\_HINDBRAIN\_MORPHOGENESIS |  | 38 | -0.39 | -1.05 | 0.387 | 0.717 | 1.000 | 3597 | tags=29%, list=17%, signal=35% |
| 630 | GO\_PHOTORECEPTOR\_CELL\_DEVELOPMENT |  | 38 | -0.34 | -1.05 | 0.383 | 0.721 | 1.000 | 3977 | tags=24%, list=18%, signal=29% |
| 631 | GO\_RESPONSE\_TO\_DSRNA |  | 66 | -0.34 | -1.05 | 0.360 | 0.721 | 1.000 | 3402 | tags=30%, list=16%, signal=36% |
| 632 | GO\_PORPHYRIN\_CONTAINING\_COMPOUND\_METABOLIC\_PROCESS |  | 35 | -0.35 | -1.05 | 0.408 | 0.721 | 1.000 | 4590 | tags=34%, list=21%, signal=43% |
| 633 | GO\_POSITIVE\_REGULATION\_OF\_ACTIN\_FILAMENT\_POLYMERIZATION |  | 63 | -0.34 | -1.05 | 0.370 | 0.720 | 1.000 | 3409 | tags=32%, list=16%, signal=38% |
| 634 | GO\_CELLULAR\_RESPONSE\_TO\_AMINO\_ACID\_STARVATION |  | 24 | -0.38 | -1.05 | 0.378 | 0.720 | 1.000 | 3121 | tags=33%, list=14%, signal=39% |
| 635 | GO\_REGULATION\_OF\_HORMONE\_BIOSYNTHETIC\_PROCESS |  | 16 | -0.44 | -1.05 | 0.379 | 0.723 | 1.000 | 2066 | tags=25%, list=9%, signal=28% |
| 636 | GO\_TOR\_SIGNALING |  | 16 | -0.39 | -1.05 | 0.399 | 0.723 | 1.000 | 1452 | tags=19%, list=7%, signal=20% |
| 637 | GO\_REGULATION\_OF\_VIRAL\_RELEASE\_FROM\_HOST\_CELL |  | 30 | -0.36 | -1.05 | 0.384 | 0.729 | 1.000 | 5642 | tags=30%, list=26%, signal=40% |
| 638 | GO\_POSITIVE\_REGULATION\_OF\_TISSUE\_REMODELING |  | 24 | -0.43 | -1.05 | 0.380 | 0.729 | 1.000 | 2781 | tags=29%, list=13%, signal=33% |
| 639 | GO\_NEGATIVE\_REGULATION\_OF\_CELL\_CYCLE\_G1\_S\_PHASE\_TRANSITION |  | 93 | -0.30 | -1.05 | 0.369 | 0.729 | 1.000 | 4022 | tags=30%, list=18%, signal=37% |
| 640 | GO\_EXTRINSIC\_APOPTOTIC\_SIGNALING\_PATHWAY |  | 96 | -0.35 | -1.05 | 0.369 | 0.730 | 1.000 | 1112 | tags=19%, list=5%, signal=20% |
| 641 | GO\_CELLULAR\_METABOLIC\_COMPOUND\_SALVAGE |  | 35 | -0.36 | -1.04 | 0.372 | 0.730 | 1.000 | 2073 | tags=29%, list=10%, signal=32% |
| 642 | GO\_APOPTOTIC\_NUCLEAR\_CHANGES |  | 24 | -0.39 | -1.04 | 0.379 | 0.729 | 1.000 | 3747 | tags=29%, list=17%, signal=35% |
| 643 | GO\_CEREBELLAR\_PURKINJE\_CELL\_LAYER\_DEVELOPMENT |  | 23 | -0.34 | -1.04 | 0.394 | 0.728 | 1.000 | 3597 | tags=22%, list=17%, signal=26% |
| 644 | GO\_NEGATIVE\_REGULATION\_OF\_CYSTEINE\_TYPE\_ENDOPEPTIDASE\_ACTIVITY |  | 84 | -0.31 | -1.04 | 0.375 | 0.727 | 1.000 | 3571 | tags=29%, list=16%, signal=34% |
| 645 | GO\_POSITIVE\_REGULATION\_OF\_TRANSCRIPTION\_INITIATION\_FROM\_RNA\_POLYMERASE\_II\_PROMOTER |  | 17 | -0.37 | -1.04 | 0.404 | 0.727 | 1.000 | 5928 | tags=53%, list=27%, signal=73% |
| 646 | GO\_MACROMITOPHAGY |  | 126 | -0.28 | -1.04 | 0.348 | 0.726 | 1.000 | 3403 | tags=27%, list=16%, signal=32% |
| 647 | GO\_ESTABLISHMENT\_OF\_MITOCHONDRION\_LOCALIZATION |  | 15 | -0.44 | -1.04 | 0.409 | 0.727 | 1.000 | 2063 | tags=20%, list=9%, signal=22% |
| 648 | GO\_CYTOPLASMIC\_SEQUESTERING\_OF\_TRANSCRIPTION\_FACTOR |  | 18 | -0.36 | -1.04 | 0.392 | 0.730 | 1.000 | 6862 | tags=50%, list=32%, signal=73% |
| 649 | GO\_FORMATION\_OF\_TRANSLATION\_PREINITIATION\_COMPLEX |  | 20 | -0.37 | -1.04 | 0.429 | 0.731 | 1.000 | 7386 | tags=45%, list=34%, signal=68% |
| 650 | GO\_CELLULAR\_RESPONSE\_TO\_PH |  | 15 | -0.41 | -1.04 | 0.419 | 0.732 | 1.000 | 3121 | tags=27%, list=14%, signal=31% |
| 651 | GO\_CELLULAR\_RESPONSE\_TO\_DSRNA |  | 33 | -0.36 | -1.04 | 0.374 | 0.732 | 1.000 | 5440 | tags=48%, list=25%, signal=65% |
| 652 | GO\_POSITIVE\_REGULATION\_OF\_PEPTIDYL\_SERINE\_PHOSPHORYLATION |  | 86 | -0.32 | -1.04 | 0.357 | 0.732 | 1.000 | 3933 | tags=30%, list=18%, signal=37% |
| 653 | GO\_NEGATIVE\_REGULATION\_OF\_MRNA\_SPLICING\_VIA\_SPLICEOSOME |  | 17 | -0.35 | -1.04 | 0.403 | 0.731 | 1.000 | 9533 | tags=65%, list=44%, signal=115% |
| 654 | GO\_LACTATION |  | 38 | -0.33 | -1.04 | 0.389 | 0.730 | 1.000 | 3368 | tags=32%, list=15%, signal=37% |
| 655 | GO\_POSITIVE\_REGULATION\_OF\_PROTEIN\_COMPLEX\_ASSEMBLY |  | 179 | -0.30 | -1.04 | 0.366 | 0.732 | 1.000 | 3591 | tags=26%, list=17%, signal=31% |
| 656 | GO\_LIPOPROTEIN\_BIOSYNTHETIC\_PROCESS |  | 82 | -0.29 | -1.04 | 0.385 | 0.736 | 1.000 | 6119 | tags=34%, list=28%, signal=47% |
| 657 | GO\_CELLULAR\_AMINO\_ACID\_BIOSYNTHETIC\_PROCESS |  | 86 | -0.34 | -1.04 | 0.403 | 0.737 | 1.000 | 2657 | tags=23%, list=12%, signal=26% |
| 658 | GO\_AMINO\_ACID\_TRANSPORT |  | 115 | -0.31 | -1.03 | 0.378 | 0.738 | 1.000 | 3362 | tags=23%, list=15%, signal=27% |
| 659 | GO\_POSITIVE\_REGULATION\_OF\_RESPONSE\_TO\_DNA\_DAMAGE\_STIMULUS |  | 59 | -0.31 | -1.03 | 0.382 | 0.742 | 1.000 | 1855 | tags=17%, list=9%, signal=18% |
| 660 | GO\_POSITIVE\_REGULATION\_OF\_VASCULAR\_ENDOTHELIAL\_GROWTH\_FACTOR\_PRODUCTION |  | 26 | -0.45 | -1.03 | 0.381 | 0.744 | 1.000 | 1225 | tags=27%, list=6%, signal=28% |
| 661 | GO\_NEGATIVE\_REGULATION\_OF\_CYTOSKELETON\_ORGANIZATION |  | 207 | -0.29 | -1.03 | 0.412 | 0.745 | 1.000 | 1463 | tags=16%, list=7%, signal=17% |
| 662 | GO\_MACROMOLECULE\_METHYLATION |  | 175 | -0.29 | -1.03 | 0.386 | 0.746 | 1.000 | 4613 | tags=30%, list=21%, signal=38% |
| 663 | GO\_REGULATION\_OF\_CHROMATIN\_SILENCING |  | 20 | -0.41 | -1.03 | 0.448 | 0.746 | 1.000 | 4613 | tags=40%, list=21%, signal=51% |
| 664 | GO\_DNA\_ALKYLATION |  | 42 | -0.40 | -1.03 | 0.402 | 0.748 | 1.000 | 4613 | tags=36%, list=21%, signal=45% |
| 665 | GO\_ALDITOL\_METABOLIC\_PROCESS |  | 19 | -0.41 | -1.03 | 0.424 | 0.748 | 1.000 | 6479 | tags=58%, list=30%, signal=82% |
| 666 | GO\_REGULATION\_OF\_DEPHOSPHORYLATION |  | 142 | -0.28 | -1.03 | 0.382 | 0.749 | 1.000 | 1768 | tags=17%, list=8%, signal=18% |
| 667 | GO\_EMBRYONIC\_PLACENTA\_DEVELOPMENT |  | 82 | -0.31 | -1.03 | 0.392 | 0.750 | 1.000 | 2865 | tags=23%, list=13%, signal=27% |
| 668 | GO\_DEVELOPMENTAL\_PROGRAMMED\_CELL\_DEATH |  | 26 | -0.40 | -1.03 | 0.397 | 0.751 | 1.000 | 253 | tags=12%, list=1%, signal=12% |
| 669 | GO\_PROTEIN\_ACETYLATION |  | 112 | -0.26 | -1.02 | 0.394 | 0.751 | 1.000 | 4611 | tags=29%, list=21%, signal=37% |
| 670 | GO\_NOTCH\_RECEPTOR\_PROCESSING |  | 16 | -0.42 | -1.02 | 0.435 | 0.752 | 1.000 | 4758 | tags=44%, list=22%, signal=56% |
| 671 | GO\_NEGATIVE\_REGULATION\_OF\_PROTEIN\_COMPLEX\_DISASSEMBLY |  | 157 | -0.32 | -1.02 | 0.429 | 0.752 | 1.000 | 2467 | tags=22%, list=11%, signal=25% |
| 672 | GO\_ONE\_CARBON\_METABOLIC\_PROCESS |  | 33 | -0.36 | -1.02 | 0.436 | 0.753 | 1.000 | 3439 | tags=30%, list=16%, signal=36% |
| 673 | GO\_OOCYTE\_DIFFERENTIATION |  | 34 | -0.38 | -1.02 | 0.426 | 0.753 | 1.000 | 1410 | tags=21%, list=6%, signal=22% |
| 674 | GO\_INTERLEUKIN\_1\_PRODUCTION |  | 15 | -0.52 | -1.02 | 0.481 | 0.759 | 1.000 | 484 | tags=20%, list=2%, signal=20% |
| 675 | GO\_BETA\_CATENIN\_TCF\_COMPLEX\_ASSEMBLY |  | 36 | -0.34 | -1.02 | 0.433 | 0.763 | 1.000 | 2964 | tags=25%, list=14%, signal=29% |
| 676 | GO\_REGULATION\_OF\_MRNA\_3\_END\_PROCESSING |  | 26 | -0.31 | -1.02 | 0.413 | 0.763 | 1.000 | 2909 | tags=19%, list=13%, signal=22% |
| 677 | GO\_NITROGEN\_COMPOUND\_TRANSPORT |  | 462 | -0.25 | -1.02 | 0.391 | 0.764 | 1.000 | 3212 | tags=21%, list=15%, signal=24% |
| 678 | GO\_PYRIMIDINE\_RIBONUCLEOTIDE\_METABOLIC\_PROCESS |  | 20 | -0.38 | -1.02 | 0.435 | 0.765 | 1.000 | 2784 | tags=25%, list=13%, signal=29% |
| 679 | GO\_REPRODUCTIVE\_SYSTEM\_DEVELOPMENT |  | 392 | -0.28 | -1.02 | 0.384 | 0.766 | 1.000 | 4075 | tags=27%, list=19%, signal=33% |
| 680 | GO\_CELL\_AGING |  | 65 | -0.33 | -1.02 | 0.401 | 0.765 | 1.000 | 3287 | tags=31%, list=15%, signal=36% |
| 681 | GO\_PEROXISOMAL\_TRANSPORT |  | 17 | -0.40 | -1.01 | 0.456 | 0.765 | 1.000 | 7021 | tags=53%, list=32%, signal=78% |
| 682 | GO\_N\_TERMINAL\_PROTEIN\_AMINO\_ACID\_MODIFICATION |  | 22 | -0.34 | -1.01 | 0.416 | 0.764 | 1.000 | 4254 | tags=32%, list=20%, signal=40% |
| 683 | GO\_RESPONSE\_TO\_PLATELET\_DERIVED\_GROWTH\_FACTOR |  | 18 | -0.47 | -1.01 | 0.432 | 0.767 | 1.000 | 1741 | tags=33%, list=8%, signal=36% |
| 684 | GO\_RESPONSE\_TO\_MINERALOCORTICOID |  | 33 | -0.40 | -1.01 | 0.423 | 0.767 | 1.000 | 5242 | tags=42%, list=24%, signal=56% |
| 685 | GO\_REGULATION\_OF\_CGMP\_BIOSYNTHETIC\_PROCESS |  | 21 | -0.41 | -1.01 | 0.437 | 0.766 | 1.000 | 1280 | tags=14%, list=6%, signal=15% |
| 686 | GO\_REGULATION\_OF\_HISTONE\_METHYLATION |  | 52 | -0.28 | -1.01 | 0.431 | 0.765 | 1.000 | 575 | tags=8%, list=3%, signal=8% |
| 687 | GO\_ERBB2\_SIGNALING\_PATHWAY |  | 38 | -0.36 | -1.01 | 0.440 | 0.766 | 1.000 | 4999 | tags=39%, list=23%, signal=51% |
| 688 | GO\_POSITIVE\_REGULATION\_OF\_PROTEIN\_OLIGOMERIZATION |  | 16 | -0.49 | -1.01 | 0.431 | 0.765 | 1.000 | 3280 | tags=50%, list=15%, signal=59% |
| 689 | GO\_NEGATIVE\_REGULATION\_OF\_RESPONSE\_TO\_DNA\_DAMAGE\_STIMULUS |  | 45 | -0.32 | -1.01 | 0.428 | 0.766 | 1.000 | 2542 | tags=31%, list=12%, signal=35% |
| 690 | GO\_REGULATION\_OF\_PROTEIN\_AUTOPHOSPHORYLATION |  | 34 | -0.39 | -1.01 | 0.456 | 0.768 | 1.000 | 1612 | tags=21%, list=7%, signal=22% |
| 691 | GO\_ACUTE\_PHASE\_RESPONSE |  | 41 | -0.39 | -1.01 | 0.445 | 0.767 | 1.000 | 2363 | tags=20%, list=11%, signal=22% |
| 692 | GO\_POSITIVE\_REGULATION\_OF\_DNA\_REPAIR |  | 34 | -0.33 | -1.01 | 0.412 | 0.767 | 1.000 | 5837 | tags=38%, list=27%, signal=52% |
| 693 | GO\_REGULATION\_OF\_ESTABLISHMENT\_OF\_PROTEIN\_LOCALIZATION\_TO\_MITOCHONDRION |  | 119 | -0.26 | -1.01 | 0.399 | 0.769 | 1.000 | 4438 | tags=31%, list=20%, signal=39% |
| 694 | GO\_REGULATION\_OF\_THYMOCYTE\_AGGREGATION |  | 25 | -0.42 | -1.01 | 0.455 | 0.768 | 1.000 | 1587 | tags=28%, list=7%, signal=30% |
| 695 | GO\_GLUTAMATE\_RECEPTOR\_SIGNALING\_PATHWAY |  | 39 | -0.32 | -1.01 | 0.460 | 0.770 | 1.000 | 1419 | tags=13%, list=7%, signal=14% |
| 696 | GO\_PYRIMIDINE\_NUCLEOBASE\_METABOLIC\_PROCESS |  | 18 | -0.39 | -1.01 | 0.464 | 0.770 | 1.000 | 3345 | tags=33%, list=15%, signal=39% |
| 697 | GO\_RIBONUCLEOSIDE\_TRIPHOSPHATE\_BIOSYNTHETIC\_PROCESS |  | 41 | -0.31 | -1.01 | 0.436 | 0.772 | 1.000 | 2730 | tags=20%, list=13%, signal=22% |
| 698 | GO\_INTRINSIC\_APOPTOTIC\_SIGNALING\_PATHWAY |  | 147 | -0.26 | -1.01 | 0.436 | 0.772 | 1.000 | 4206 | tags=29%, list=19%, signal=36% |
| 699 | GO\_POSITIVE\_REGULATION\_OF\_CATABOLIC\_PROCESS |  | 379 | -0.23 | -1.00 | 0.434 | 0.772 | 1.000 | 4109 | tags=25%, list=19%, signal=30% |
| 700 | GO\_PURINE\_CONTAINING\_COMPOUND\_BIOSYNTHETIC\_PROCESS |  | 127 | -0.28 | -1.00 | 0.455 | 0.773 | 1.000 | 2981 | tags=25%, list=14%, signal=29% |
| 701 | GO\_CEREBELLAR\_CORTEX\_FORMATION |  | 21 | -0.38 | -1.00 | 0.434 | 0.772 | 1.000 | 3597 | tags=33%, list=17%, signal=40% |
| 702 | GO\_REGULATION\_OF\_TELOMERE\_CAPPING |  | 21 | -0.34 | -1.00 | 0.469 | 0.773 | 1.000 | 420 | tags=10%, list=2%, signal=10% |
| 703 | GO\_RESPONSE\_TO\_VITAMIN\_A |  | 20 | -0.41 | -1.00 | 0.461 | 0.779 | 1.000 | 1418 | tags=15%, list=7%, signal=16% |
| 704 | GO\_NUCLEAR\_TRANSCRIBED\_MRNA\_POLY\_A\_TAIL\_SHORTENING |  | 22 | -0.30 | -1.00 | 0.437 | 0.778 | 1.000 | 4946 | tags=36%, list=23%, signal=47% |
| 705 | GO\_ATP\_GENERATION\_FROM\_ADP |  | 35 | -0.36 | -1.00 | 0.451 | 0.781 | 1.000 | 2503 | tags=31%, list=12%, signal=35% |
| 706 | GO\_ANTIGEN\_PROCESSING\_AND\_PRESENTATION\_OF\_PEPTIDE\_OR\_POLYSACCHARIDE\_ANTIGEN\_VIA\_MHC\_CLASS\_II |  | 86 | -0.32 | -1.00 | 0.423 | 0.780 | 1.000 | 2893 | tags=23%, list=13%, signal=27% |
| 707 | GO\_TRANSLATIONAL\_INITIATION |  | 125 | -0.24 | -1.00 | 0.470 | 0.779 | 1.000 | 6806 | tags=27%, list=31%, signal=39% |
| 708 | GO\_RESPONSE\_TO\_INTERFERON\_BETA |  | 21 | -0.51 | -1.00 | 0.514 | 0.779 | 1.000 | 2446 | tags=29%, list=11%, signal=32% |
| 709 | GO\_MITOCHONDRIAL\_RESPIRATORY\_CHAIN\_COMPLEX\_ASSEMBLY |  | 60 | -0.30 | -1.00 | 0.450 | 0.780 | 1.000 | 7499 | tags=37%, list=34%, signal=56% |
| 710 | GO\_REGULATION\_OF\_POSITIVE\_CHEMOTAXIS |  | 22 | -0.44 | -1.00 | 0.470 | 0.780 | 1.000 | 743 | tags=27%, list=3%, signal=28% |
| 711 | GO\_NEGATIVE\_REGULATION\_OF\_CELL\_CYCLE\_ARREST |  | 18 | -0.35 | -1.00 | 0.476 | 0.781 | 1.000 | 3075 | tags=33%, list=14%, signal=39% |
| 712 | GO\_CELLULAR\_KETONE\_METABOLIC\_PROCESS |  | 62 | -0.36 | -1.00 | 0.433 | 0.780 | 1.000 | 4175 | tags=29%, list=19%, signal=36% |
| 713 | GO\_QUINONE\_METABOLIC\_PROCESS |  | 27 | -0.47 | -1.00 | 0.495 | 0.779 | 1.000 | 3891 | tags=30%, list=18%, signal=36% |
| 714 | GO\_REGULATION\_OF\_HISTONE\_H3\_K4\_METHYLATION |  | 24 | -0.36 | -1.00 | 0.468 | 0.778 | 1.000 | 575 | tags=8%, list=3%, signal=9% |
| 715 | GO\_CARBOHYDRATE\_TRANSPORT |  | 90 | -0.30 | -1.00 | 0.456 | 0.778 | 1.000 | 3731 | tags=29%, list=17%, signal=35% |
| 716 | GO\_MITOCHONDRIAL\_GENOME\_MAINTENANCE |  | 21 | -0.39 | -1.00 | 0.471 | 0.778 | 1.000 | 6045 | tags=52%, list=28%, signal=72% |
| 717 | GO\_PROTEIN\_HETEROTETRAMERIZATION |  | 29 | -0.42 | -1.00 | 0.495 | 0.778 | 1.000 | 6337 | tags=52%, list=29%, signal=73% |
| 718 | GO\_OVARIAN\_FOLLICLE\_DEVELOPMENT |  | 57 | -0.33 | -0.99 | 0.451 | 0.778 | 1.000 | 2147 | tags=19%, list=10%, signal=21% |
| 719 | GO\_ANTIGEN\_PROCESSING\_AND\_PRESENTATION\_OF\_EXOGENOUS\_PEPTIDE\_ANTIGEN\_VIA\_MHC\_CLASS\_I |  | 62 | -0.36 | -0.99 | 0.472 | 0.777 | 1.000 | 3980 | tags=35%, list=18%, signal=43% |
| 720 | GO\_REGULATION\_OF\_CELL\_CYCLE\_G2\_M\_PHASE\_TRANSITION |  | 58 | -0.35 | -0.99 | 0.465 | 0.781 | 1.000 | 3198 | tags=34%, list=15%, signal=40% |
| 721 | GO\_REGULATION\_OF\_MYELINATION |  | 29 | -0.35 | -0.99 | 0.493 | 0.780 | 1.000 | 3474 | tags=24%, list=16%, signal=29% |
| 722 | GO\_GOLGI\_VESICLE\_TRANSPORT |  | 299 | -0.24 | -0.99 | 0.454 | 0.783 | 1.000 | 4462 | tags=22%, list=21%, signal=27% |
| 723 | GO\_RESPONSE\_TO\_HYDROGEN\_PEROXIDE |  | 106 | -0.30 | -0.99 | 0.446 | 0.783 | 1.000 | 1612 | tags=17%, list=7%, signal=18% |
| 724 | GO\_RESPONSE\_TO\_TYPE\_I\_INTERFERON |  | 67 | -0.51 | -0.99 | 0.536 | 0.786 | 1.000 | 2470 | tags=27%, list=11%, signal=30% |
| 725 | GO\_POSITIVE\_REGULATION\_OF\_EXTRINSIC\_APOPTOTIC\_SIGNALING\_PATHWAY |  | 50 | -0.37 | -0.99 | 0.479 | 0.789 | 1.000 | 3594 | tags=38%, list=17%, signal=45% |
| 726 | GO\_COVALENT\_CHROMATIN\_MODIFICATION |  | 315 | -0.23 | -0.99 | 0.455 | 0.788 | 1.000 | 4452 | tags=25%, list=20%, signal=31% |
| 727 | GO\_RESPONSE\_TO\_ARSENIC\_CONTAINING\_SUBSTANCE |  | 28 | -0.39 | -0.99 | 0.458 | 0.794 | 1.000 | 4334 | tags=39%, list=20%, signal=49% |
| 728 | GO\_AMINE\_CATABOLIC\_PROCESS |  | 20 | -0.40 | -0.99 | 0.466 | 0.793 | 1.000 | 4362 | tags=35%, list=20%, signal=44% |
| 729 | GO\_VIRAL\_LIFE\_CYCLE |  | 256 | -0.23 | -0.99 | 0.469 | 0.792 | 1.000 | 4685 | tags=22%, list=22%, signal=28% |
| 730 | GO\_CALCIUM\_DEPENDENT\_CELL\_CELL\_ADHESION\_VIA\_PLASMA\_MEMBRANE\_CELL\_ADHESION\_MOLECULES |  | 23 | -0.43 | -0.98 | 0.476 | 0.792 | 1.000 | 4159 | tags=43%, list=19%, signal=54% |
| 731 | GO\_REGULATION\_OF\_DEFENSE\_RESPONSE\_TO\_VIRUS |  | 179 | -0.30 | -0.98 | 0.460 | 0.791 | 1.000 | 3130 | tags=26%, list=14%, signal=30% |
| 732 | GO\_MRNA\_SPLICE\_SITE\_SELECTION |  | 25 | -0.32 | -0.98 | 0.467 | 0.795 | 1.000 | 5932 | tags=32%, list=27%, signal=44% |
| 733 | GO\_CELLULAR\_AMINO\_ACID\_METABOLIC\_PROCESS |  | 306 | -0.28 | -0.98 | 0.484 | 0.799 | 1.000 | 2989 | tags=23%, list=14%, signal=26% |
| 734 | GO\_POSITIVE\_REGULATION\_OF\_INTERLEUKIN\_8\_PRODUCTION |  | 44 | -0.39 | -0.98 | 0.485 | 0.805 | 1.000 | 1243 | tags=20%, list=6%, signal=22% |
| 735 | GO\_DETECTION\_OF\_LIGHT\_STIMULUS\_INVOLVED\_IN\_SENSORY\_PERCEPTION |  | 18 | -0.34 | -0.98 | 0.489 | 0.806 | 1.000 | 169 | tags=6%, list=1%, signal=6% |
| 736 | GO\_NUCLEOSIDE\_MONOPHOSPHATE\_METABOLIC\_PROCESS |  | 219 | -0.24 | -0.98 | 0.477 | 0.805 | 1.000 | 2503 | tags=17%, list=12%, signal=19% |
| 737 | GO\_CLATHRIN\_MEDIATED\_ENDOCYTOSIS |  | 35 | -0.34 | -0.98 | 0.478 | 0.804 | 1.000 | 2859 | tags=20%, list=13%, signal=23% |
| 738 | GO\_REGULATION\_OF\_TYPE\_I\_INTERFERON\_PRODUCTION |  | 107 | -0.35 | -0.98 | 0.481 | 0.806 | 1.000 | 3541 | tags=31%, list=16%, signal=37% |
| 739 | GO\_REGULATION\_OF\_NEUROBLAST\_PROLIFERATION |  | 27 | -0.38 | -0.97 | 0.487 | 0.813 | 1.000 | 2652 | tags=26%, list=12%, signal=29% |
| 740 | GO\_CELLULAR\_PIGMENTATION |  | 43 | -0.32 | -0.97 | 0.485 | 0.815 | 1.000 | 4286 | tags=33%, list=20%, signal=40% |
| 741 | GO\_INTRACELLULAR\_STEROID\_HORMONE\_RECEPTOR\_SIGNALING\_PATHWAY |  | 67 | -0.26 | -0.97 | 0.510 | 0.818 | 1.000 | 4473 | tags=31%, list=21%, signal=39% |
| 742 | GO\_NEGATIVE\_REGULATION\_OF\_VIRAL\_GENOME\_REPLICATION |  | 45 | -0.44 | -0.97 | 0.503 | 0.820 | 1.000 | 3823 | tags=38%, list=18%, signal=46% |
| 743 | GO\_PEPTIDYL\_LYSINE\_TRIMETHYLATION |  | 18 | -0.39 | -0.97 | 0.489 | 0.819 | 1.000 | 1524 | tags=17%, list=7%, signal=18% |
| 744 | GO\_PLATELET\_AGGREGATION |  | 37 | -0.34 | -0.97 | 0.483 | 0.822 | 1.000 | 2097 | tags=19%, list=10%, signal=21% |
| 745 | GO\_ANDROGEN\_RECEPTOR\_SIGNALING\_PATHWAY |  | 40 | -0.30 | -0.97 | 0.501 | 0.822 | 1.000 | 4473 | tags=33%, list=21%, signal=41% |
| 746 | GO\_GLUCOSE\_CATABOLIC\_PROCESS |  | 28 | -0.39 | -0.97 | 0.517 | 0.823 | 1.000 | 2005 | tags=36%, list=9%, signal=39% |
| 747 | GO\_POSITIVE\_REGULATION\_OF\_PEPTIDYL\_THREONINE\_PHOSPHORYLATION |  | 23 | -0.40 | -0.97 | 0.515 | 0.822 | 1.000 | 2982 | tags=35%, list=14%, signal=40% |
| 748 | GO\_GERM\_CELL\_DEVELOPMENT |  | 190 | -0.25 | -0.97 | 0.545 | 0.821 | 1.000 | 2922 | tags=17%, list=13%, signal=19% |
| 749 | GO\_MAMMARY\_GLAND\_EPITHELIUM\_DEVELOPMENT |  | 51 | -0.36 | -0.97 | 0.497 | 0.822 | 1.000 | 2595 | tags=27%, list=12%, signal=31% |
| 750 | GO\_GLYCOSYL\_COMPOUND\_CATABOLIC\_PROCESS |  | 39 | -0.34 | -0.97 | 0.537 | 0.822 | 1.000 | 1438 | tags=23%, list=7%, signal=25% |
| 751 | GO\_NEGATIVE\_REGULATION\_OF\_PROTEIN\_ACETYLATION |  | 20 | -0.36 | -0.97 | 0.506 | 0.821 | 1.000 | 4546 | tags=35%, list=21%, signal=44% |
| 752 | GO\_EYE\_PHOTORECEPTOR\_CELL\_DEVELOPMENT |  | 30 | -0.33 | -0.96 | 0.482 | 0.823 | 1.000 | 3977 | tags=27%, list=18%, signal=33% |
| 753 | GO\_DNA\_DOUBLE\_STRAND\_BREAK\_PROCESSING |  | 19 | -0.41 | -0.96 | 0.507 | 0.824 | 1.000 | 7540 | tags=68%, list=35%, signal=105% |
| 754 | GO\_POSITIVE\_REGULATION\_OF\_ALCOHOL\_BIOSYNTHETIC\_PROCESS |  | 22 | -0.41 | -0.96 | 0.521 | 0.823 | 1.000 | 2341 | tags=18%, list=11%, signal=20% |
| 755 | GO\_CELLULAR\_RESPONSE\_TO\_HYDROGEN\_PEROXIDE |  | 60 | -0.31 | -0.96 | 0.485 | 0.823 | 1.000 | 3121 | tags=23%, list=14%, signal=27% |
| 756 | GO\_REGULATION\_OF\_MAMMARY\_GLAND\_EPITHELIAL\_CELL\_PROLIFERATION |  | 16 | -0.39 | -0.96 | 0.516 | 0.822 | 1.000 | 3368 | tags=44%, list=15%, signal=52% |
| 757 | GO\_VESICLE\_CYTOSKELETAL\_TRAFFICKING |  | 35 | -0.29 | -0.96 | 0.489 | 0.822 | 1.000 | 2117 | tags=14%, list=10%, signal=16% |
| 758 | GO\_ADP\_METABOLIC\_PROCESS |  | 42 | -0.33 | -0.96 | 0.502 | 0.823 | 1.000 | 2503 | tags=26%, list=12%, signal=30% |
| 759 | GO\_SYNAPTIC\_TRANSMISSION\_DOPAMINERGIC |  | 17 | -0.41 | -0.96 | 0.521 | 0.824 | 1.000 | 9838 | tags=65%, list=45%, signal=118% |
| 760 | GO\_ORGAN\_REGENERATION |  | 80 | -0.31 | -0.96 | 0.513 | 0.824 | 1.000 | 3584 | tags=34%, list=16%, signal=40% |
| 761 | GO\_REGULATION\_OF\_INTERLEUKIN\_2\_BIOSYNTHETIC\_PROCESS |  | 17 | -0.50 | -0.96 | 0.537 | 0.824 | 1.000 | 1264 | tags=24%, list=6%, signal=25% |
| 762 | GO\_REGULATION\_OF\_CELL\_MATURATION |  | 17 | -0.40 | -0.96 | 0.523 | 0.826 | 1.000 | 1371 | tags=29%, list=6%, signal=31% |
| 763 | GO\_VENTRICULAR\_CARDIAC\_MUSCLE\_CELL\_DIFFERENTIATION |  | 18 | -0.41 | -0.96 | 0.514 | 0.825 | 1.000 | 1551 | tags=33%, list=7%, signal=36% |
| 764 | GO\_POSITIVE\_REGULATION\_OF\_CELLULAR\_AMIDE\_METABOLIC\_PROCESS |  | 95 | -0.26 | -0.96 | 0.523 | 0.824 | 1.000 | 3997 | tags=26%, list=18%, signal=32% |
| 765 | GO\_REGULATION\_OF\_INTERLEUKIN\_8\_PRODUCTION |  | 56 | -0.36 | -0.96 | 0.532 | 0.823 | 1.000 | 1243 | tags=16%, list=6%, signal=17% |
| 766 | GO\_NEGATIVE\_REGULATION\_OF\_HEMATOPOIETIC\_PROGENITOR\_CELL\_DIFFERENTIATION |  | 18 | -0.41 | -0.96 | 0.510 | 0.824 | 1.000 | 4056 | tags=28%, list=19%, signal=34% |
| 767 | GO\_POSITIVE\_REGULATION\_OF\_CARDIAC\_MUSCLE\_TISSUE\_DEVELOPMENT |  | 27 | -0.40 | -0.96 | 0.519 | 0.824 | 1.000 | 2762 | tags=37%, list=13%, signal=42% |
| 768 | GO\_REGULATION\_OF\_FIBROBLAST\_MIGRATION |  | 27 | -0.37 | -0.96 | 0.531 | 0.824 | 1.000 | 3435 | tags=30%, list=16%, signal=35% |
| 769 | GO\_POSITIVE\_REGULATION\_OF\_RESPONSE\_TO\_EXTRACELLULAR\_STIMULUS |  | 47 | -0.28 | -0.96 | 0.519 | 0.824 | 1.000 | 3331 | tags=19%, list=15%, signal=23% |
| 770 | GO\_PEPTIDYL\_SERINE\_MODIFICATION |  | 140 | -0.26 | -0.96 | 0.537 | 0.827 | 1.000 | 3287 | tags=22%, list=15%, signal=26% |
| 771 | GO\_SYMPATHETIC\_NERVOUS\_SYSTEM\_DEVELOPMENT |  | 21 | -0.41 | -0.95 | 0.546 | 0.828 | 1.000 | 4989 | tags=33%, list=23%, signal=43% |
| 772 | GO\_FIBRINOLYSIS |  | 21 | -0.41 | -0.95 | 0.536 | 0.828 | 1.000 | 489 | tags=19%, list=2%, signal=19% |
| 773 | GO\_POSITIVE\_REGULATION\_OF\_PROTEIN\_TARGETING\_TO\_MEMBRANE |  | 15 | -0.37 | -0.95 | 0.536 | 0.828 | 1.000 | 4558 | tags=27%, list=21%, signal=34% |
| 774 | GO\_ESTABLISHMENT\_OF\_TISSUE\_POLARITY |  | 17 | -0.43 | -0.95 | 0.518 | 0.830 | 1.000 | 5095 | tags=53%, list=23%, signal=69% |
| 775 | GO\_NEGATIVE\_REGULATION\_OF\_RNA\_SPLICING |  | 20 | -0.30 | -0.95 | 0.476 | 0.829 | 1.000 | 9533 | tags=60%, list=44%, signal=107% |
| 776 | GO\_SIGNAL\_TRANSDUCTION\_INVOLVED\_IN\_REGULATION\_OF\_GENE\_EXPRESSION |  | 18 | -0.39 | -0.95 | 0.507 | 0.829 | 1.000 | 1017 | tags=17%, list=5%, signal=17% |
| 777 | GO\_FAT\_SOLUBLE\_VITAMIN\_METABOLIC\_PROCESS |  | 30 | -0.39 | -0.95 | 0.522 | 0.828 | 1.000 | 2683 | tags=23%, list=12%, signal=27% |
| 778 | GO\_REGULATION\_OF\_RESPONSE\_TO\_EXTRACELLULAR\_STIMULUS |  | 165 | -0.22 | -0.95 | 0.532 | 0.827 | 1.000 | 4682 | tags=26%, list=22%, signal=33% |
| 779 | GO\_REGULATION\_OF\_CYTOSKELETON\_ORGANIZATION |  | 471 | -0.25 | -0.95 | 0.592 | 0.827 | 1.000 | 2286 | tags=18%, list=11%, signal=20% |
| 780 | GO\_FIBROBLAST\_GROWTH\_FACTOR\_RECEPTOR\_SIGNALING\_PATHWAY |  | 81 | -0.28 | -0.95 | 0.521 | 0.828 | 1.000 | 6122 | tags=32%, list=28%, signal=45% |
| 781 | GO\_NAD\_METABOLIC\_PROCESS |  | 50 | -0.31 | -0.95 | 0.545 | 0.827 | 1.000 | 2005 | tags=26%, list=9%, signal=29% |
| 782 | GO\_RESPONSE\_TO\_EXOGENOUS\_DSRNA |  | 44 | -0.35 | -0.95 | 0.511 | 0.827 | 1.000 | 3402 | tags=32%, list=16%, signal=38% |
| 783 | GO\_PROTEIN\_DEALKYLATION |  | 27 | -0.32 | -0.95 | 0.512 | 0.827 | 1.000 | 3797 | tags=26%, list=17%, signal=31% |
| 784 | GO\_RESPONSE\_TO\_INTERFERON\_ALPHA |  | 20 | -0.51 | -0.95 | 0.579 | 0.827 | 1.000 | 2466 | tags=30%, list=11%, signal=34% |
| 785 | GO\_NEGATIVE\_REGULATION\_OF\_VIRAL\_TRANSCRIPTION |  | 23 | -0.37 | -0.95 | 0.512 | 0.829 | 1.000 | 6035 | tags=48%, list=28%, signal=66% |
| 786 | GO\_PIGMENT\_GRANULE\_LOCALIZATION |  | 23 | -0.36 | -0.95 | 0.524 | 0.828 | 1.000 | 4286 | tags=35%, list=20%, signal=43% |
| 787 | GO\_REGULATION\_OF\_FIBROBLAST\_PROLIFERATION |  | 78 | -0.31 | -0.95 | 0.550 | 0.827 | 1.000 | 3859 | tags=38%, list=18%, signal=47% |
| 788 | GO\_SINGLE\_ORGANISM\_MEMBRANE\_FUSION |  | 114 | -0.26 | -0.95 | 0.578 | 0.827 | 1.000 | 4270 | tags=25%, list=20%, signal=31% |
| 789 | GO\_POSITIVE\_REGULATION\_OF\_MRNA\_3\_END\_PROCESSING |  | 16 | -0.34 | -0.95 | 0.516 | 0.827 | 1.000 | 2909 | tags=25%, list=13%, signal=29% |
| 790 | GO\_REGULATION\_OF\_DNA\_DAMAGE\_RESPONSE\_SIGNAL\_TRANSDUCTION\_BY\_P53\_CLASS\_MEDIATOR |  | 27 | -0.35 | -0.95 | 0.521 | 0.826 | 1.000 | 2129 | tags=30%, list=10%, signal=33% |
| 791 | GO\_RESPONSE\_TO\_COPPER\_ION |  | 27 | -0.34 | -0.95 | 0.547 | 0.828 | 1.000 | 2781 | tags=30%, list=13%, signal=34% |
| 792 | GO\_REGULATION\_OF\_PHOSPHOPROTEIN\_PHOSPHATASE\_ACTIVITY |  | 55 | -0.29 | -0.95 | 0.545 | 0.828 | 1.000 | 4035 | tags=29%, list=19%, signal=36% |
| 793 | GO\_COFACTOR\_METABOLIC\_PROCESS |  | 308 | -0.27 | -0.95 | 0.529 | 0.827 | 1.000 | 5309 | tags=33%, list=24%, signal=44% |
| 794 | GO\_POSITIVE\_REGULATION\_OF\_DNA\_BIOSYNTHETIC\_PROCESS |  | 54 | -0.30 | -0.95 | 0.540 | 0.828 | 1.000 | 4779 | tags=41%, list=22%, signal=52% |
| 795 | GO\_POSITIVE\_REGULATION\_OF\_FIBROBLAST\_PROLIFERATION |  | 51 | -0.35 | -0.94 | 0.574 | 0.829 | 1.000 | 3859 | tags=45%, list=18%, signal=55% |
| 796 | GO\_MICROTUBULE\_NUCLEATION |  | 18 | -0.35 | -0.94 | 0.523 | 0.828 | 1.000 | 7277 | tags=50%, list=33%, signal=75% |
| 797 | GO\_NUCLEOBASE\_METABOLIC\_PROCESS |  | 37 | -0.34 | -0.94 | 0.551 | 0.828 | 1.000 | 3584 | tags=41%, list=16%, signal=48% |
| 798 | GO\_PERICARDIUM\_DEVELOPMENT |  | 17 | -0.38 | -0.94 | 0.521 | 0.829 | 1.000 | 3859 | tags=35%, list=18%, signal=43% |
| 799 | GO\_POSITIVE\_REGULATION\_OF\_PROTEIN\_EXPORT\_FROM\_NUCLEUS |  | 19 | -0.35 | -0.94 | 0.553 | 0.829 | 1.000 | 1034 | tags=11%, list=5%, signal=11% |
| 800 | GO\_ORGANELLE\_DISASSEMBLY |  | 176 | -0.24 | -0.94 | 0.600 | 0.829 | 1.000 | 3403 | tags=23%, list=16%, signal=27% |
| 801 | GO\_POSITIVE\_REGULATION\_OF\_PROTEIN\_POLYMERIZATION |  | 83 | -0.28 | -0.94 | 0.609 | 0.833 | 1.000 | 3591 | tags=27%, list=17%, signal=32% |
| 802 | GO\_ACIDIC\_AMINO\_ACID\_TRANSPORT |  | 21 | -0.34 | -0.94 | 0.575 | 0.840 | 1.000 | 658 | tags=10%, list=3%, signal=10% |
| 803 | GO\_TEMPERATURE\_HOMEOSTASIS |  | 26 | -0.35 | -0.94 | 0.559 | 0.841 | 1.000 | 26 | tags=8%, list=0%, signal=8% |
| 804 | GO\_REGULATION\_OF\_HORMONE\_METABOLIC\_PROCESS |  | 24 | -0.36 | -0.94 | 0.569 | 0.840 | 1.000 | 2066 | tags=21%, list=9%, signal=23% |
| 805 | GO\_LIPOPOLYSACCHARIDE\_MEDIATED\_SIGNALING\_PATHWAY |  | 30 | -0.41 | -0.94 | 0.535 | 0.841 | 1.000 | 4248 | tags=40%, list=20%, signal=50% |
| 806 | GO\_POLYOL\_CATABOLIC\_PROCESS |  | 18 | -0.33 | -0.93 | 0.530 | 0.844 | 1.000 | 3474 | tags=28%, list=16%, signal=33% |
| 807 | GO\_REGULATION\_OF\_VASCULAR\_ENDOTHELIAL\_GROWTH\_FACTOR\_RECEPTOR\_SIGNALING\_PATHWAY |  | 25 | -0.37 | -0.93 | 0.547 | 0.845 | 1.000 | 2372 | tags=24%, list=11%, signal=27% |
| 808 | GO\_POSITIVE\_REGULATION\_OF\_AXONOGENESIS |  | 66 | -0.32 | -0.93 | 0.570 | 0.850 | 1.000 | 209 | tags=9%, list=1%, signal=9% |
| 809 | GO\_RESPONSE\_TO\_EXTRACELLULAR\_STIMULUS |  | 426 | -0.26 | -0.93 | 0.703 | 0.850 | 1.000 | 3455 | tags=22%, list=16%, signal=26% |
| 810 | GO\_ORGANOPHOSPHATE\_BIOSYNTHETIC\_PROCESS |  | 418 | -0.23 | -0.93 | 0.713 | 0.849 | 1.000 | 3592 | tags=24%, list=17%, signal=29% |
| 811 | GO\_REGULATION\_OF\_DENDRITE\_EXTENSION |  | 18 | -0.38 | -0.93 | 0.563 | 0.849 | 1.000 | 1621 | tags=22%, list=7%, signal=24% |
| 812 | GO\_REGULATION\_OF\_MACROPHAGE\_DIFFERENTIATION |  | 20 | -0.35 | -0.93 | 0.569 | 0.848 | 1.000 | 1907 | tags=25%, list=9%, signal=27% |
| 813 | GO\_RESPONSE\_TO\_PROGESTERONE |  | 46 | -0.36 | -0.93 | 0.576 | 0.847 | 1.000 | 4109 | tags=37%, list=19%, signal=45% |
| 814 | GO\_NEGATIVE\_REGULATION\_OF\_CATABOLIC\_PROCESS |  | 189 | -0.23 | -0.93 | 0.680 | 0.850 | 1.000 | 2946 | tags=16%, list=14%, signal=19% |
| 815 | GO\_LIPID\_STORAGE |  | 25 | -0.38 | -0.93 | 0.561 | 0.849 | 1.000 | 2923 | tags=28%, list=13%, signal=32% |
| 816 | GO\_ORGANELLE\_FUSION |  | 116 | -0.25 | -0.93 | 0.630 | 0.849 | 1.000 | 4270 | tags=25%, list=20%, signal=31% |
| 817 | GO\_ASPARTATE\_FAMILY\_AMINO\_ACID\_METABOLIC\_PROCESS |  | 53 | -0.32 | -0.93 | 0.584 | 0.850 | 1.000 | 3366 | tags=25%, list=15%, signal=29% |
| 818 | GO\_NITROGEN\_CYCLE\_METABOLIC\_PROCESS |  | 15 | -0.42 | -0.93 | 0.576 | 0.849 | 1.000 | 2524 | tags=27%, list=12%, signal=30% |
| 819 | GO\_POSITIVE\_REGULATION\_OF\_AXON\_EXTENSION |  | 35 | -0.34 | -0.93 | 0.547 | 0.850 | 1.000 | 209 | tags=9%, list=1%, signal=9% |
| 820 | GO\_INTRACELLULAR\_RECEPTOR\_SIGNALING\_PATHWAY |  | 159 | -0.24 | -0.93 | 0.598 | 0.849 | 1.000 | 4579 | tags=26%, list=21%, signal=33% |
| 821 | GO\_REGULATION\_OF\_NFAT\_PROTEIN\_IMPORT\_INTO\_NUCLEUS |  | 17 | -0.35 | -0.93 | 0.568 | 0.849 | 1.000 | 3630 | tags=24%, list=17%, signal=28% |
| 822 | GO\_POSITIVE\_REGULATION\_OF\_NEUROBLAST\_PROLIFERATION |  | 21 | -0.38 | -0.93 | 0.579 | 0.849 | 1.000 | 2652 | tags=24%, list=12%, signal=27% |
| 823 | GO\_ANATOMICAL\_STRUCTURE\_ARRANGEMENT |  | 17 | -0.36 | -0.92 | 0.573 | 0.850 | 1.000 | 1654 | tags=18%, list=8%, signal=19% |
| 824 | GO\_ERBB\_SIGNALING\_PATHWAY |  | 76 | -0.29 | -0.92 | 0.606 | 0.850 | 1.000 | 2938 | tags=24%, list=14%, signal=27% |
| 825 | GO\_NUCLEOSIDE\_TRIPHOSPHATE\_METABOLIC\_PROCESS |  | 204 | -0.24 | -0.92 | 0.568 | 0.851 | 1.000 | 2503 | tags=17%, list=12%, signal=19% |
| 826 | GO\_SUBSTRATE\_DEPENDENT\_CELL\_MIGRATION |  | 26 | -0.37 | -0.92 | 0.605 | 0.850 | 1.000 | 913 | tags=15%, list=4%, signal=16% |
| 827 | GO\_MICROTUBULE\_ANCHORING |  | 17 | -0.33 | -0.92 | 0.561 | 0.850 | 1.000 | 4331 | tags=29%, list=20%, signal=37% |
| 828 | GO\_REGULATION\_OF\_MONOOXYGENASE\_ACTIVITY |  | 58 | -0.30 | -0.92 | 0.647 | 0.851 | 1.000 | 3439 | tags=24%, list=16%, signal=29% |
| 829 | GO\_PLACENTA\_BLOOD\_VESSEL\_DEVELOPMENT |  | 27 | -0.33 | -0.92 | 0.586 | 0.852 | 1.000 | 3245 | tags=30%, list=15%, signal=35% |
| 830 | GO\_MODULATION\_BY\_VIRUS\_OF\_HOST\_MORPHOLOGY\_OR\_PHYSIOLOGY |  | 35 | -0.29 | -0.92 | 0.547 | 0.854 | 1.000 | 4109 | tags=31%, list=19%, signal=39% |
| 831 | GO\_REGULATION\_OF\_EXTRINSIC\_APOPTOTIC\_SIGNALING\_PATHWAY\_VIA\_DEATH\_DOMAIN\_RECEPTORS |  | 52 | -0.32 | -0.92 | 0.573 | 0.853 | 1.000 | 2067 | tags=23%, list=10%, signal=25% |
| 832 | GO\_INACTIVATION\_OF\_MAPK\_ACTIVITY |  | 25 | -0.37 | -0.92 | 0.579 | 0.852 | 1.000 | 4366 | tags=44%, list=20%, signal=55% |
| 833 | GO\_REGULATION\_OF\_RETINOIC\_ACID\_RECEPTOR\_SIGNALING\_PATHWAY |  | 19 | -0.38 | -0.92 | 0.588 | 0.852 | 1.000 | 1374 | tags=21%, list=6%, signal=22% |
| 834 | GO\_MAMMARY\_GLAND\_LOBULE\_DEVELOPMENT |  | 16 | -0.41 | -0.92 | 0.614 | 0.854 | 1.000 | 2263 | tags=31%, list=10%, signal=35% |
| 835 | GO\_GENITALIA\_DEVELOPMENT |  | 41 | -0.32 | -0.92 | 0.556 | 0.853 | 1.000 | 3859 | tags=29%, list=18%, signal=36% |
| 836 | GO\_RIBONUCLEOSIDE\_DIPHOSPHATE\_METABOLIC\_PROCESS |  | 59 | -0.28 | -0.92 | 0.594 | 0.856 | 1.000 | 2005 | tags=19%, list=9%, signal=20% |
| 837 | GO\_MITOCHONDRIAL\_RESPIRATORY\_CHAIN\_COMPLEX\_I\_BIOGENESIS |  | 48 | -0.28 | -0.92 | 0.523 | 0.855 | 1.000 | 7499 | tags=38%, list=34%, signal=57% |
| 838 | GO\_POSITIVE\_REGULATION\_OF\_ORGANELLE\_ASSEMBLY |  | 43 | -0.28 | -0.92 | 0.598 | 0.857 | 1.000 | 5735 | tags=35%, list=26%, signal=47% |
| 839 | GO\_LENS\_DEVELOPMENT\_IN\_CAMERA\_TYPE\_EYE |  | 63 | -0.33 | -0.92 | 0.594 | 0.857 | 1.000 | 2447 | tags=29%, list=11%, signal=32% |
| 840 | GO\_BICARBONATE\_TRANSPORT |  | 41 | -0.32 | -0.92 | 0.581 | 0.858 | 1.000 | 2025 | tags=20%, list=9%, signal=21% |
| 841 | GO\_NEGATIVE\_REGULATION\_OF\_INTERLEUKIN\_1\_PRODUCTION |  | 16 | -0.41 | -0.92 | 0.587 | 0.857 | 1.000 | 1919 | tags=19%, list=9%, signal=21% |
| 842 | GO\_INTRINSIC\_APOPTOTIC\_SIGNALING\_PATHWAY\_BY\_P53\_CLASS\_MEDIATOR |  | 51 | -0.29 | -0.91 | 0.576 | 0.859 | 1.000 | 3977 | tags=25%, list=18%, signal=31% |
| 843 | GO\_REGULATION\_OF\_SYMBIOSIS\_ENCOMPASSING\_MUTUALISM\_THROUGH\_PARASITISM |  | 190 | -0.28 | -0.91 | 0.566 | 0.858 | 1.000 | 5849 | tags=36%, list=27%, signal=49% |
| 844 | GO\_ALPHA\_AMINO\_ACID\_METABOLIC\_PROCESS |  | 210 | -0.27 | -0.91 | 0.637 | 0.858 | 1.000 | 2989 | tags=24%, list=14%, signal=27% |
| 845 | GO\_REGULATION\_OF\_PROTEIN\_TYROSINE\_KINASE\_ACTIVITY |  | 57 | -0.29 | -0.91 | 0.661 | 0.859 | 1.000 | 1665 | tags=19%, list=8%, signal=21% |
| 846 | GO\_GLYCOPROTEIN\_CATABOLIC\_PROCESS |  | 15 | -0.36 | -0.91 | 0.626 | 0.861 | 1.000 | 3869 | tags=27%, list=18%, signal=32% |
| 847 | GO\_PROXIMAL\_DISTAL\_PATTERN\_FORMATION |  | 31 | -0.42 | -0.91 | 0.582 | 0.861 | 1.000 | 2652 | tags=35%, list=12%, signal=40% |
| 848 | GO\_POSITIVE\_REGULATION\_OF\_MUSCLE\_CONTRACTION |  | 43 | -0.33 | -0.91 | 0.615 | 0.864 | 1.000 | 859 | tags=14%, list=4%, signal=14% |
| 849 | GO\_NEUROMUSCULAR\_PROCESS\_CONTROLLING\_BALANCE |  | 47 | -0.26 | -0.91 | 0.615 | 0.867 | 1.000 | 1670 | tags=11%, list=8%, signal=11% |
| 850 | GO\_ACTIN\_NUCLEATION |  | 22 | -0.31 | -0.91 | 0.591 | 0.866 | 1.000 | 407 | tags=9%, list=2%, signal=9% |
| 851 | GO\_ANTIGEN\_PROCESSING\_AND\_PRESENTATION\_OF\_PEPTIDE\_ANTIGEN |  | 165 | -0.28 | -0.91 | 0.568 | 0.866 | 1.000 | 4023 | tags=28%, list=18%, signal=34% |
| 852 | GO\_LABYRINTHINE\_LAYER\_DEVELOPMENT |  | 44 | -0.29 | -0.91 | 0.655 | 0.866 | 1.000 | 4779 | tags=34%, list=22%, signal=44% |
| 853 | GO\_NEGATIVE\_REGULATION\_OF\_T\_CELL\_DIFFERENTIATION |  | 31 | -0.34 | -0.91 | 0.587 | 0.867 | 1.000 | 1587 | tags=19%, list=7%, signal=21% |
| 854 | GO\_REGULATION\_OF\_CIRCADIAN\_RHYTHM |  | 98 | -0.25 | -0.91 | 0.659 | 0.866 | 1.000 | 2037 | tags=15%, list=9%, signal=17% |
| 855 | GO\_ER\_ASSOCIATED\_UBIQUITIN\_DEPENDENT\_PROTEIN\_CATABOLIC\_PROCESS |  | 59 | -0.26 | -0.91 | 0.587 | 0.866 | 1.000 | 5011 | tags=29%, list=23%, signal=37% |
| 856 | GO\_PEPTIDYL\_THREONINE\_MODIFICATION |  | 44 | -0.26 | -0.90 | 0.634 | 0.870 | 1.000 | 2833 | tags=20%, list=13%, signal=23% |
| 857 | GO\_REGULATION\_OF\_EPIDERMAL\_GROWTH\_FACTOR\_ACTIVATED\_RECEPTOR\_ACTIVITY |  | 21 | -0.35 | -0.90 | 0.588 | 0.871 | 1.000 | 3856 | tags=33%, list=18%, signal=40% |
| 858 | GO\_ORGANIC\_ANION\_TRANSPORT |  | 352 | -0.25 | -0.90 | 0.745 | 0.871 | 1.000 | 3235 | tags=21%, list=15%, signal=25% |
| 859 | GO\_HISTONE\_H4\_ACETYLATION |  | 41 | -0.28 | -0.90 | 0.560 | 0.871 | 1.000 | 4164 | tags=29%, list=19%, signal=36% |
| 860 | GO\_INTERACTION\_WITH\_SYMBIONT |  | 50 | -0.27 | -0.90 | 0.596 | 0.871 | 1.000 | 5849 | tags=32%, list=27%, signal=44% |
| 861 | GO\_POSITIVE\_REGULATION\_OF\_GROWTH |  | 226 | -0.24 | -0.90 | 0.724 | 0.871 | 1.000 | 3183 | tags=23%, list=15%, signal=26% |
| 862 | GO\_REGULATION\_OF\_VIRAL\_GENOME\_REPLICATION |  | 69 | -0.35 | -0.90 | 0.567 | 0.870 | 1.000 | 5774 | tags=41%, list=27%, signal=55% |
| 863 | GO\_CELLULAR\_RESPONSE\_TO\_LEPTIN\_STIMULUS |  | 16 | -0.35 | -0.90 | 0.596 | 0.871 | 1.000 | 90 | tags=6%, list=0%, signal=6% |
| 864 | GO\_PROTEIN\_K63\_LINKED\_DEUBIQUITINATION |  | 23 | -0.31 | -0.90 | 0.616 | 0.876 | 1.000 | 3149 | tags=26%, list=14%, signal=30% |
| 865 | GO\_POSITIVE\_REGULATION\_OF\_PROTEASOMAL\_PROTEIN\_CATABOLIC\_PROCESS |  | 96 | -0.24 | -0.90 | 0.681 | 0.878 | 1.000 | 3243 | tags=23%, list=15%, signal=27% |
| 866 | GO\_POSITIVE\_REGULATION\_OF\_MYELOID\_CELL\_DIFFERENTIATION |  | 77 | -0.29 | -0.90 | 0.630 | 0.877 | 1.000 | 3970 | tags=29%, list=18%, signal=35% |
| 867 | GO\_REGULATION\_OF\_MRNA\_SPLICING\_VIA\_SPLICEOSOME |  | 55 | -0.23 | -0.90 | 0.546 | 0.878 | 1.000 | 4533 | tags=27%, list=21%, signal=34% |
| 868 | GO\_COFACTOR\_CATABOLIC\_PROCESS |  | 18 | -0.42 | -0.90 | 0.620 | 0.878 | 1.000 | 2967 | tags=33%, list=14%, signal=39% |
| 869 | GO\_DEFENSE\_RESPONSE\_TO\_VIRUS |  | 150 | -0.38 | -0.89 | 0.585 | 0.881 | 1.000 | 3320 | tags=29%, list=15%, signal=34% |
| 870 | GO\_REGULATION\_OF\_RELEASE\_OF\_CYTOCHROME\_C\_FROM\_MITOCHONDRIA |  | 42 | -0.30 | -0.89 | 0.646 | 0.880 | 1.000 | 3299 | tags=29%, list=15%, signal=34% |
| 871 | GO\_POSITIVE\_REGULATION\_OF\_MYELOID\_LEUKOCYTE\_DIFFERENTIATION |  | 46 | -0.32 | -0.89 | 0.633 | 0.880 | 1.000 | 2333 | tags=20%, list=11%, signal=22% |
| 872 | GO\_REGULATION\_OF\_VASCULAR\_ENDOTHELIAL\_GROWTH\_FACTOR\_PRODUCTION |  | 30 | -0.39 | -0.89 | 0.613 | 0.879 | 1.000 | 1225 | tags=27%, list=6%, signal=28% |
| 873 | GO\_CARBOHYDRATE\_DERIVATIVE\_TRANSPORT |  | 47 | -0.27 | -0.89 | 0.622 | 0.879 | 1.000 | 5856 | tags=30%, list=27%, signal=41% |
| 874 | GO\_REGULATION\_OF\_PROTEIN\_TARGETING\_TO\_MEMBRANE |  | 20 | -0.30 | -0.89 | 0.615 | 0.878 | 1.000 | 1289 | tags=10%, list=6%, signal=11% |
| 875 | GO\_DENDRITIC\_SPINE\_DEVELOPMENT |  | 19 | -0.33 | -0.89 | 0.625 | 0.878 | 1.000 | 5640 | tags=42%, list=26%, signal=57% |
| 876 | GO\_GLYCOSYL\_COMPOUND\_METABOLIC\_PROCESS |  | 334 | -0.22 | -0.89 | 0.683 | 0.878 | 1.000 | 2984 | tags=18%, list=14%, signal=21% |
| 877 | GO\_REGULATION\_OF\_TRANSCRIPTION\_INITIATION\_FROM\_RNA\_POLYMERASE\_II\_PROMOTER |  | 23 | -0.28 | -0.89 | 0.645 | 0.878 | 1.000 | 5928 | tags=39%, list=27%, signal=54% |
| 878 | GO\_REGULATION\_OF\_PHOSPHATASE\_ACTIVITY |  | 115 | -0.26 | -0.89 | 0.702 | 0.878 | 1.000 | 4179 | tags=28%, list=19%, signal=34% |
| 879 | GO\_NEUROBLAST\_PROLIFERATION |  | 28 | -0.36 | -0.89 | 0.613 | 0.877 | 1.000 | 2215 | tags=21%, list=10%, signal=24% |
| 880 | GO\_NUCLEOTIDE\_EXCISION\_REPAIR\_PREINCISION\_COMPLEX\_ASSEMBLY |  | 28 | -0.29 | -0.89 | 0.590 | 0.877 | 1.000 | 5725 | tags=32%, list=26%, signal=44% |
| 881 | GO\_ARGININE\_METABOLIC\_PROCESS |  | 17 | -0.39 | -0.89 | 0.637 | 0.876 | 1.000 | 2382 | tags=24%, list=11%, signal=26% |
| 882 | GO\_NEGATIVE\_REGULATION\_OF\_DEPHOSPHORYLATION |  | 68 | -0.28 | -0.89 | 0.667 | 0.875 | 1.000 | 2947 | tags=22%, list=14%, signal=25% |
| 883 | GO\_CELLULAR\_PROTEIN\_COMPLEX\_LOCALIZATION |  | 22 | -0.34 | -0.89 | 0.624 | 0.875 | 1.000 | 2290 | tags=23%, list=11%, signal=25% |
| 884 | GO\_RETROGRADE\_TRANSPORT\_ENDOSOME\_TO\_GOLGI |  | 64 | -0.26 | -0.89 | 0.647 | 0.875 | 1.000 | 6797 | tags=42%, list=31%, signal=61% |
| 885 | GO\_REGULATION\_OF\_TYROSINE\_PHOSPHORYLATION\_OF\_STAT\_PROTEIN |  | 67 | -0.32 | -0.89 | 0.691 | 0.875 | 1.000 | 2057 | tags=21%, list=9%, signal=23% |
| 886 | GO\_VESICLE\_DOCKING\_INVOLVED\_IN\_EXOCYTOSIS |  | 34 | -0.28 | -0.89 | 0.608 | 0.875 | 1.000 | 4479 | tags=32%, list=21%, signal=41% |
| 887 | GO\_NEGATIVE\_REGULATION\_OF\_VIRAL\_PROCESS |  | 83 | -0.35 | -0.89 | 0.582 | 0.876 | 1.000 | 5642 | tags=41%, list=26%, signal=55% |
| 888 | GO\_REGULATION\_OF\_CARDIAC\_MUSCLE\_CELL\_PROLIFERATION |  | 29 | -0.36 | -0.89 | 0.637 | 0.875 | 1.000 | 2653 | tags=31%, list=12%, signal=35% |
| 889 | GO\_RESPONSE\_TO\_STEROL |  | 22 | -0.35 | -0.89 | 0.676 | 0.878 | 1.000 | 1025 | tags=18%, list=5%, signal=19% |
| 890 | GO\_MEMBRANE\_FUSION |  | 139 | -0.24 | -0.89 | 0.714 | 0.878 | 1.000 | 4270 | tags=24%, list=20%, signal=30% |
| 891 | GO\_VESICLE\_TARGETING |  | 74 | -0.26 | -0.89 | 0.639 | 0.881 | 1.000 | 3405 | tags=24%, list=16%, signal=29% |
| 892 | GO\_POSITIVE\_REGULATION\_OF\_MULTI\_ORGANISM\_PROCESS |  | 145 | -0.24 | -0.88 | 0.696 | 0.882 | 1.000 | 4109 | tags=23%, list=19%, signal=29% |
| 893 | GO\_COFACTOR\_TRANSPORT |  | 26 | -0.32 | -0.88 | 0.676 | 0.882 | 1.000 | 3434 | tags=35%, list=16%, signal=41% |
| 894 | GO\_INTRINSIC\_APOPTOTIC\_SIGNALING\_PATHWAY\_IN\_RESPONSE\_TO\_ENDOPLASMIC\_RETICULUM\_STRESS |  | 30 | -0.29 | -0.88 | 0.639 | 0.883 | 1.000 | 1344 | tags=17%, list=6%, signal=18% |
| 895 | GO\_CELLULAR\_RESPONSE\_TO\_FLUID\_SHEAR\_STRESS |  | 19 | -0.38 | -0.88 | 0.658 | 0.882 | 1.000 | 859 | tags=26%, list=4%, signal=27% |
| 896 | GO\_NEGATIVE\_REGULATION\_OF\_TELOMERE\_MAINTENANCE |  | 26 | -0.27 | -0.88 | 0.593 | 0.881 | 1.000 | 7356 | tags=46%, list=34%, signal=70% |
| 897 | GO\_REGULATION\_OF\_PROTEIN\_DEACETYLATION |  | 32 | -0.29 | -0.88 | 0.658 | 0.884 | 1.000 | 1112 | tags=13%, list=5%, signal=13% |
| 898 | GO\_INDOLALKYLAMINE\_METABOLIC\_PROCESS |  | 16 | -0.41 | -0.88 | 0.611 | 0.884 | 1.000 | 2806 | tags=25%, list=13%, signal=29% |
| 899 | GO\_ACTIVATION\_OF\_NF\_KAPPAB\_INDUCING\_KINASE\_ACTIVITY |  | 17 | -0.37 | -0.88 | 0.623 | 0.885 | 1.000 | 2490 | tags=29%, list=11%, signal=33% |
| 900 | GO\_NEUROMUSCULAR\_PROCESS |  | 92 | -0.25 | -0.88 | 0.708 | 0.885 | 1.000 | 1698 | tags=12%, list=8%, signal=13% |
| 901 | GO\_REGULATION\_OF\_ANION\_TRANSMEMBRANE\_TRANSPORT |  | 27 | -0.30 | -0.88 | 0.627 | 0.890 | 1.000 | 3761 | tags=22%, list=17%, signal=27% |
| 902 | GO\_REGULATION\_OF\_PROTEIN\_HOMODIMERIZATION\_ACTIVITY |  | 22 | -0.34 | -0.88 | 0.681 | 0.893 | 1.000 | 2781 | tags=36%, list=13%, signal=42% |
| 903 | GO\_POSITIVE\_REGULATION\_OF\_LIPID\_CATABOLIC\_PROCESS |  | 25 | -0.32 | -0.88 | 0.644 | 0.894 | 1.000 | 3080 | tags=16%, list=14%, signal=19% |
| 904 | GO\_REGULATION\_OF\_DNA\_BIOSYNTHETIC\_PROCESS |  | 87 | -0.25 | -0.88 | 0.716 | 0.893 | 1.000 | 4779 | tags=37%, list=22%, signal=47% |
| 905 | GO\_OXIDOREDUCTION\_COENZYME\_METABOLIC\_PROCESS |  | 96 | -0.26 | -0.87 | 0.663 | 0.893 | 1.000 | 2005 | tags=20%, list=9%, signal=22% |
| 906 | GO\_POSITIVE\_REGULATION\_OF\_STAT\_CASCADE |  | 72 | -0.31 | -0.87 | 0.741 | 0.893 | 1.000 | 812 | tags=14%, list=4%, signal=14% |
| 907 | GO\_REGULATION\_OF\_ENDOCRINE\_PROCESS |  | 47 | -0.32 | -0.87 | 0.669 | 0.892 | 1.000 | 1907 | tags=15%, list=9%, signal=16% |
| 908 | GO\_MRNA\_TRANSCRIPTION |  | 20 | -0.33 | -0.87 | 0.640 | 0.892 | 1.000 | 3368 | tags=30%, list=15%, signal=35% |
| 909 | GO\_POSITIVE\_REGULATION\_OF\_CARDIAC\_MUSCLE\_CELL\_PROLIFERATION |  | 19 | -0.39 | -0.87 | 0.656 | 0.891 | 1.000 | 2653 | tags=37%, list=12%, signal=42% |
| 910 | GO\_VENTRICULAR\_SYSTEM\_DEVELOPMENT |  | 25 | -0.33 | -0.87 | 0.628 | 0.890 | 1.000 | 2870 | tags=28%, list=13%, signal=32% |
| 911 | GO\_NEGATIVE\_REGULATION\_OF\_DNA\_BINDING |  | 46 | -0.30 | -0.87 | 0.679 | 0.890 | 1.000 | 3888 | tags=28%, list=18%, signal=34% |
| 912 | GO\_POSITIVE\_REGULATION\_OF\_STEM\_CELL\_PROLIFERATION |  | 61 | -0.33 | -0.87 | 0.667 | 0.893 | 1.000 | 2932 | tags=25%, list=13%, signal=28% |
| 913 | GO\_REGULATION\_OF\_B\_CELL\_APOPTOTIC\_PROCESS |  | 18 | -0.38 | -0.87 | 0.626 | 0.893 | 1.000 | 3493 | tags=44%, list=16%, signal=53% |
| 914 | GO\_NEGATIVE\_REGULATION\_OF\_NEURAL\_PRECURSOR\_CELL\_PROLIFERATION |  | 20 | -0.34 | -0.87 | 0.659 | 0.897 | 1.000 | 1025 | tags=15%, list=5%, signal=16% |
| 915 | GO\_PYRIMIDINE\_CONTAINING\_COMPOUND\_CATABOLIC\_PROCESS |  | 28 | -0.29 | -0.87 | 0.717 | 0.897 | 1.000 | 4678 | tags=39%, list=22%, signal=50% |
| 916 | GO\_MODIFIED\_AMINO\_ACID\_TRANSPORT |  | 25 | -0.33 | -0.87 | 0.715 | 0.897 | 1.000 | 3106 | tags=32%, list=14%, signal=37% |
| 917 | GO\_CELLULAR\_SENESCENCE |  | 32 | -0.31 | -0.87 | 0.715 | 0.897 | 1.000 | 3625 | tags=31%, list=17%, signal=37% |
| 918 | GO\_ORGANIC\_ACID\_TRANSPORT |  | 240 | -0.25 | -0.87 | 0.851 | 0.897 | 1.000 | 3235 | tags=22%, list=15%, signal=26% |
| 919 | GO\_NEGATIVE\_REGULATION\_OF\_WOUND\_HEALING |  | 57 | -0.31 | -0.87 | 0.789 | 0.896 | 1.000 | 880 | tags=16%, list=4%, signal=16% |
| 920 | GO\_SCF\_DEPENDENT\_PROTEASOMAL\_UBIQUITIN\_DEPENDENT\_PROTEIN\_CATABOLIC\_PROCESS |  | 23 | -0.31 | -0.87 | 0.684 | 0.895 | 1.000 | 5811 | tags=43%, list=27%, signal=59% |
| 921 | GO\_POSITIVE\_REGULATION\_OF\_RESPONSE\_TO\_CYTOKINE\_STIMULUS |  | 29 | -0.34 | -0.87 | 0.663 | 0.896 | 1.000 | 3859 | tags=41%, list=18%, signal=50% |
| 922 | GO\_MODIFICATION\_BY\_SYMBIONT\_OF\_HOST\_MORPHOLOGY\_OR\_PHYSIOLOGY |  | 43 | -0.26 | -0.87 | 0.616 | 0.895 | 1.000 | 4109 | tags=26%, list=19%, signal=31% |
| 923 | GO\_REGULATION\_OF\_OXIDATIVE\_STRESS\_INDUCED\_INTRINSIC\_APOPTOTIC\_SIGNALING\_PATHWAY |  | 28 | -0.27 | -0.87 | 0.633 | 0.894 | 1.000 | 5887 | tags=39%, list=27%, signal=54% |
| 924 | GO\_REGULATION\_OF\_TELOMERASE\_ACTIVITY |  | 39 | -0.28 | -0.87 | 0.685 | 0.894 | 1.000 | 5037 | tags=38%, list=23%, signal=50% |
| 925 | GO\_ANTERIOR\_POSTERIOR\_AXIS\_SPECIFICATION |  | 46 | -0.28 | -0.87 | 0.707 | 0.895 | 1.000 | 5737 | tags=39%, list=26%, signal=53% |
| 926 | GO\_RESPONSE\_TO\_NUTRIENT |  | 189 | -0.26 | -0.86 | 0.835 | 0.897 | 1.000 | 3455 | tags=22%, list=16%, signal=26% |
| 927 | GO\_MYELOID\_LEUKOCYTE\_MEDIATED\_IMMUNITY |  | 40 | -0.35 | -0.86 | 0.651 | 0.897 | 1.000 | 2217 | tags=20%, list=10%, signal=22% |
| 928 | GO\_JNK\_CASCADE |  | 80 | -0.26 | -0.86 | 0.783 | 0.900 | 1.000 | 3268 | tags=26%, list=15%, signal=31% |
| 929 | GO\_POSITIVE\_REGULATION\_OF\_NEUROTRANSMITTER\_TRANSPORT |  | 15 | -0.38 | -0.86 | 0.661 | 0.900 | 1.000 | 707 | tags=13%, list=3%, signal=14% |
| 930 | GO\_REGULATION\_OF\_INTERLEUKIN\_6\_BIOSYNTHETIC\_PROCESS |  | 15 | -0.41 | -0.86 | 0.618 | 0.902 | 1.000 | 2486 | tags=20%, list=11%, signal=23% |
| 931 | GO\_REGULATION\_OF\_OXIDATIVE\_STRESS\_INDUCED\_CELL\_DEATH |  | 43 | -0.25 | -0.86 | 0.679 | 0.902 | 1.000 | 5887 | tags=42%, list=27%, signal=57% |
| 932 | GO\_SULFUR\_COMPOUND\_TRANSPORT |  | 30 | -0.30 | -0.86 | 0.679 | 0.904 | 1.000 | 3662 | tags=27%, list=17%, signal=32% |
| 933 | GO\_NEURAL\_PRECURSOR\_CELL\_PROLIFERATION |  | 69 | -0.29 | -0.86 | 0.684 | 0.903 | 1.000 | 2948 | tags=22%, list=14%, signal=25% |
| 934 | GO\_AXIS\_SPECIFICATION |  | 88 | -0.27 | -0.86 | 0.772 | 0.903 | 1.000 | 5423 | tags=34%, list=25%, signal=45% |
| 935 | GO\_CHROMATIN\_DISASSEMBLY |  | 17 | -0.30 | -0.86 | 0.627 | 0.903 | 1.000 | 3821 | tags=24%, list=18%, signal=29% |
| 936 | GO\_COENZYME\_METABOLIC\_PROCESS |  | 241 | -0.25 | -0.86 | 0.711 | 0.903 | 1.000 | 3050 | tags=20%, list=14%, signal=23% |
| 937 | GO\_CELLULAR\_COMPONENT\_MAINTENANCE |  | 18 | -0.34 | -0.86 | 0.714 | 0.903 | 1.000 | 3686 | tags=33%, list=17%, signal=40% |
| 938 | GO\_MISFOLDED\_OR\_INCOMPLETELY\_SYNTHESIZED\_PROTEIN\_CATABOLIC\_PROCESS |  | 15 | -0.28 | -0.86 | 0.619 | 0.903 | 1.000 | 7148 | tags=47%, list=33%, signal=69% |
| 939 | GO\_REGULATION\_OF\_NEURAL\_PRECURSOR\_CELL\_PROLIFERATION |  | 69 | -0.29 | -0.86 | 0.763 | 0.905 | 1.000 | 2652 | tags=22%, list=12%, signal=25% |
| 940 | GO\_MANNOSYLATION |  | 27 | -0.31 | -0.86 | 0.639 | 0.904 | 1.000 | 5489 | tags=48%, list=25%, signal=64% |
| 941 | GO\_MRNA\_CLEAVAGE |  | 16 | -0.30 | -0.86 | 0.675 | 0.905 | 1.000 | 3700 | tags=38%, list=17%, signal=45% |
| 942 | GO\_RESPONSE\_TO\_DEXAMETHASONE |  | 33 | -0.33 | -0.85 | 0.724 | 0.906 | 1.000 | 4334 | tags=39%, list=20%, signal=49% |
| 943 | GO\_NEGATIVE\_REGULATION\_OF\_LYMPHOCYTE\_DIFFERENTIATION |  | 39 | -0.30 | -0.85 | 0.718 | 0.908 | 1.000 | 1587 | tags=18%, list=7%, signal=19% |
| 944 | GO\_GLYCOLIPID\_BIOSYNTHETIC\_PROCESS |  | 59 | -0.26 | -0.85 | 0.752 | 0.908 | 1.000 | 3557 | tags=22%, list=16%, signal=26% |
| 945 | GO\_EMBRYONIC\_DIGIT\_MORPHOGENESIS |  | 59 | -0.31 | -0.85 | 0.692 | 0.908 | 1.000 | 3026 | tags=27%, list=14%, signal=31% |
| 946 | GO\_PEROXISOME\_ORGANIZATION |  | 32 | -0.28 | -0.85 | 0.627 | 0.907 | 1.000 | 6424 | tags=38%, list=30%, signal=53% |
| 947 | GO\_ENDOSOME\_ORGANIZATION |  | 58 | -0.25 | -0.85 | 0.658 | 0.906 | 1.000 | 5384 | tags=28%, list=25%, signal=37% |
| 948 | GO\_MICROTUBULE\_BUNDLE\_FORMATION |  | 56 | -0.28 | -0.85 | 0.740 | 0.906 | 1.000 | 993 | tags=9%, list=5%, signal=9% |
| 949 | GO\_REGULATION\_OF\_CHEMOKINE\_PRODUCTION |  | 61 | -0.32 | -0.85 | 0.731 | 0.905 | 1.000 | 3212 | tags=36%, list=15%, signal=42% |
| 950 | GO\_HIPPOCAMPUS\_DEVELOPMENT |  | 72 | -0.27 | -0.85 | 0.717 | 0.905 | 1.000 | 3797 | tags=22%, list=17%, signal=27% |
| 951 | GO\_NUCLEOTIDE\_SUGAR\_METABOLIC\_PROCESS |  | 31 | -0.31 | -0.85 | 0.658 | 0.905 | 1.000 | 4024 | tags=32%, list=18%, signal=40% |
| 952 | GO\_PYRIMIDINE\_NUCLEOSIDE\_CATABOLIC\_PROCESS |  | 18 | -0.36 | -0.85 | 0.684 | 0.904 | 1.000 | 1429 | tags=22%, list=7%, signal=24% |
| 953 | GO\_EPIDERMAL\_GROWTH\_FACTOR\_RECEPTOR\_SIGNALING\_PATHWAY |  | 52 | -0.29 | -0.85 | 0.734 | 0.908 | 1.000 | 1259 | tags=19%, list=6%, signal=20% |
| 954 | GO\_POSITIVE\_REGULATION\_OF\_TYPE\_I\_INTERFERON\_PRODUCTION |  | 70 | -0.31 | -0.85 | 0.611 | 0.909 | 1.000 | 3478 | tags=29%, list=16%, signal=34% |
| 955 | GO\_RESPONSE\_TO\_HEAT |  | 85 | -0.29 | -0.85 | 0.760 | 0.910 | 1.000 | 3362 | tags=31%, list=15%, signal=36% |
| 956 | GO\_MODIFICATION\_OF\_MORPHOLOGY\_OR\_PHYSIOLOGY\_OF\_OTHER\_ORGANISM |  | 95 | -0.26 | -0.85 | 0.745 | 0.911 | 1.000 | 4644 | tags=26%, list=21%, signal=33% |
| 957 | GO\_POSITIVE\_REGULATION\_OF\_INTERLEUKIN\_6\_PRODUCTION |  | 64 | -0.33 | -0.85 | 0.724 | 0.913 | 1.000 | 2872 | tags=25%, list=13%, signal=29% |
| 958 | GO\_POSITIVE\_REGULATION\_OF\_CHEMOKINE\_PRODUCTION |  | 47 | -0.34 | -0.85 | 0.712 | 0.912 | 1.000 | 3212 | tags=38%, list=15%, signal=45% |
| 959 | GO\_REGULATION\_OF\_INSULIN\_RECEPTOR\_SIGNALING\_PATHWAY |  | 41 | -0.27 | -0.84 | 0.770 | 0.915 | 1.000 | 3959 | tags=27%, list=18%, signal=33% |
| 960 | GO\_ENDOCRINE\_PANCREAS\_DEVELOPMENT |  | 39 | -0.31 | -0.84 | 0.729 | 0.919 | 1.000 | 2595 | tags=21%, list=12%, signal=23% |
| 961 | GO\_MYD88\_INDEPENDENT\_TOLL\_LIKE\_RECEPTOR\_SIGNALING\_PATHWAY |  | 29 | -0.34 | -0.84 | 0.638 | 0.919 | 1.000 | 1831 | tags=14%, list=8%, signal=15% |
| 962 | GO\_PIRNA\_METABOLIC\_PROCESS |  | 15 | -0.42 | -0.84 | 0.717 | 0.918 | 1.000 | 2514 | tags=33%, list=12%, signal=38% |
| 963 | GO\_REGULATION\_OF\_STEM\_CELL\_PROLIFERATION |  | 84 | -0.30 | -0.84 | 0.778 | 0.918 | 1.000 | 2652 | tags=25%, list=12%, signal=28% |
| 964 | GO\_NEGATIVE\_REGULATION\_OF\_DENDRITE\_DEVELOPMENT |  | 27 | -0.28 | -0.84 | 0.756 | 0.924 | 1.000 | 4993 | tags=37%, list=23%, signal=48% |
| 965 | GO\_NEGATIVE\_REGULATION\_OF\_RECEPTOR\_MEDIATED\_ENDOCYTOSIS |  | 17 | -0.28 | -0.84 | 0.688 | 0.923 | 1.000 | 7287 | tags=47%, list=33%, signal=71% |
| 966 | GO\_CEREBRAL\_CORTEX\_RADIALLY\_ORIENTED\_CELL\_MIGRATION |  | 27 | -0.28 | -0.84 | 0.701 | 0.923 | 1.000 | 2870 | tags=19%, list=13%, signal=21% |
| 967 | GO\_ALCOHOL\_CATABOLIC\_PROCESS |  | 57 | -0.27 | -0.84 | 0.783 | 0.923 | 1.000 | 3474 | tags=19%, list=16%, signal=23% |
| 968 | GO\_POSITIVE\_REGULATION\_OF\_HEART\_GROWTH |  | 26 | -0.34 | -0.84 | 0.697 | 0.924 | 1.000 | 3167 | tags=38%, list=15%, signal=45% |
| 969 | GO\_POSITIVE\_REGULATION\_OF\_NEURAL\_PRECURSOR\_CELL\_PROLIFERATION |  | 39 | -0.33 | -0.83 | 0.741 | 0.925 | 1.000 | 2652 | tags=23%, list=12%, signal=26% |
| 970 | GO\_NEGATIVE\_REGULATION\_OF\_PROTEIN\_BINDING |  | 78 | -0.26 | -0.83 | 0.810 | 0.926 | 1.000 | 2066 | tags=17%, list=9%, signal=18% |
| 971 | GO\_REGULATION\_OF\_ERBB\_SIGNALING\_PATHWAY |  | 78 | -0.24 | -0.83 | 0.798 | 0.925 | 1.000 | 4103 | tags=27%, list=19%, signal=33% |
| 972 | GO\_POSITIVE\_REGULATION\_OF\_OSTEOCLAST\_DIFFERENTIATION |  | 23 | -0.36 | -0.83 | 0.710 | 0.926 | 1.000 | 2333 | tags=22%, list=11%, signal=24% |
| 973 | GO\_CELLULAR\_RESPONSE\_TO\_DEXAMETHASONE\_STIMULUS |  | 27 | -0.32 | -0.83 | 0.782 | 0.930 | 1.000 | 4334 | tags=44%, list=20%, signal=55% |
| 974 | GO\_RESPONSE\_TO\_COCAINE |  | 45 | -0.26 | -0.83 | 0.793 | 0.931 | 1.000 | 4241 | tags=22%, list=19%, signal=28% |
| 975 | GO\_CELLULAR\_RESPONSE\_TO\_OXIDATIVE\_STRESS |  | 177 | -0.23 | -0.83 | 0.908 | 0.930 | 1.000 | 3888 | tags=25%, list=18%, signal=31% |
| 976 | GO\_MAMMARY\_GLAND\_DEVELOPMENT |  | 109 | -0.27 | -0.83 | 0.858 | 0.929 | 1.000 | 2784 | tags=24%, list=13%, signal=27% |
| 977 | GO\_ACETYL\_COA\_METABOLIC\_PROCESS |  | 24 | -0.30 | -0.83 | 0.744 | 0.930 | 1.000 | 3366 | tags=21%, list=15%, signal=25% |
| 978 | GO\_ACTIVATION\_OF\_MAPKK\_ACTIVITY |  | 51 | -0.27 | -0.83 | 0.830 | 0.929 | 1.000 | 3992 | tags=31%, list=18%, signal=38% |
| 979 | GO\_RESPONSE\_TO\_REACTIVE\_OXYGEN\_SPECIES |  | 186 | -0.25 | -0.83 | 0.856 | 0.929 | 1.000 | 1612 | tags=15%, list=7%, signal=16% |
| 980 | GO\_NEGATIVE\_REGULATION\_OF\_GENE\_SILENCING |  | 17 | -0.32 | -0.83 | 0.667 | 0.935 | 1.000 | 3881 | tags=24%, list=18%, signal=29% |
| 981 | GO\_RESPONSE\_TO\_INTERLEUKIN\_4 |  | 30 | -0.32 | -0.82 | 0.709 | 0.936 | 1.000 | 1578 | tags=17%, list=7%, signal=18% |
| 982 | GO\_POSITIVE\_REGULATION\_OF\_EXTRINSIC\_APOPTOTIC\_SIGNALING\_PATHWAY\_VIA\_DEATH\_DOMAIN\_RECEPTORS |  | 16 | -0.38 | -0.82 | 0.726 | 0.936 | 1.000 | 2067 | tags=38%, list=10%, signal=41% |
| 983 | GO\_CELLULAR\_GLUCOSE\_HOMEOSTASIS |  | 73 | -0.26 | -0.82 | 0.787 | 0.936 | 1.000 | 2295 | tags=15%, list=11%, signal=17% |
| 984 | GO\_MYELOID\_DENDRITIC\_CELL\_DIFFERENTIATION |  | 19 | -0.42 | -0.82 | 0.696 | 0.938 | 1.000 | 1072 | tags=21%, list=5%, signal=22% |
| 985 | GO\_PLATELET\_DERIVED\_GROWTH\_FACTOR\_RECEPTOR\_SIGNALING\_PATHWAY |  | 34 | -0.31 | -0.82 | 0.702 | 0.938 | 1.000 | 4075 | tags=35%, list=19%, signal=43% |
| 986 | GO\_NEGATIVE\_REGULATION\_OF\_LIPID\_METABOLIC\_PROCESS |  | 69 | -0.27 | -0.82 | 0.894 | 0.937 | 1.000 | 2595 | tags=19%, list=12%, signal=21% |
| 987 | GO\_MONOSACCHARIDE\_BIOSYNTHETIC\_PROCESS |  | 53 | -0.28 | -0.82 | 0.760 | 0.937 | 1.000 | 1952 | tags=21%, list=9%, signal=23% |
| 988 | GO\_NEGATIVE\_REGULATION\_OF\_VIRAL\_RELEASE\_FROM\_HOST\_CELL |  | 15 | -0.35 | -0.82 | 0.680 | 0.936 | 1.000 | 5642 | tags=47%, list=26%, signal=63% |
| 989 | GO\_REGULATION\_OF\_RESPONSE\_TO\_BIOTIC\_STIMULUS |  | 220 | -0.25 | -0.82 | 0.764 | 0.937 | 1.000 | 3130 | tags=24%, list=14%, signal=27% |
| 990 | GO\_GOLGI\_TO\_PLASMA\_MEMBRANE\_PROTEIN\_TRANSPORT |  | 25 | -0.27 | -0.82 | 0.739 | 0.942 | 1.000 | 599 | tags=8%, list=3%, signal=8% |
| 991 | GO\_VACUOLAR\_TRANSPORT |  | 228 | -0.20 | -0.82 | 0.857 | 0.943 | 1.000 | 4151 | tags=21%, list=19%, signal=25% |
| 992 | GO\_CELLULAR\_RESPONSE\_TO\_INTERLEUKIN\_4 |  | 25 | -0.33 | -0.82 | 0.708 | 0.943 | 1.000 | 1578 | tags=20%, list=7%, signal=22% |
| 993 | GO\_INNATE\_IMMUNE\_RESPONSE\_IN\_MUCOSA |  | 18 | -0.41 | -0.82 | 0.715 | 0.943 | 1.000 | 2200 | tags=28%, list=10%, signal=31% |
| 994 | GO\_VITAMIN\_METABOLIC\_PROCESS |  | 110 | -0.27 | -0.82 | 0.846 | 0.943 | 1.000 | 2902 | tags=25%, list=13%, signal=28% |
| 995 | GO\_REGULATION\_OF\_PEPTIDYL\_THREONINE\_PHOSPHORYLATION |  | 34 | -0.30 | -0.82 | 0.775 | 0.942 | 1.000 | 2982 | tags=24%, list=14%, signal=27% |
| 996 | GO\_TUMOR\_NECROSIS\_FACTOR\_MEDIATED\_SIGNALING\_PATHWAY |  | 112 | -0.27 | -0.81 | 0.723 | 0.947 | 1.000 | 4025 | tags=33%, list=19%, signal=40% |
| 997 | GO\_RESPONSE\_TO\_LEAD\_ION |  | 20 | -0.31 | -0.81 | 0.716 | 0.946 | 1.000 | 4613 | tags=45%, list=21%, signal=57% |
| 998 | GO\_DICARBOXYLIC\_ACID\_METABOLIC\_PROCESS |  | 97 | -0.24 | -0.81 | 0.834 | 0.947 | 1.000 | 2975 | tags=16%, list=14%, signal=19% |
| 999 | GO\_MULTICELLULAR\_ORGANISM\_AGING |  | 28 | -0.30 | -0.81 | 0.758 | 0.946 | 1.000 | 2975 | tags=29%, list=14%, signal=33% |
| 1000 | GO\_NEGATIVE\_REGULATION\_OF\_JNK\_CASCADE |  | 32 | -0.30 | -0.81 | 0.757 | 0.950 | 1.000 | 2829 | tags=28%, list=13%, signal=32% |
| 1001 | GO\_REGULATION\_OF\_LIPID\_BIOSYNTHETIC\_PROCESS |  | 121 | -0.25 | -0.81 | 0.924 | 0.950 | 1.000 | 2374 | tags=16%, list=11%, signal=18% |
| 1002 | GO\_REGULATION\_OF\_NIK\_NF\_KAPPAB\_SIGNALING |  | 41 | -0.29 | -0.81 | 0.780 | 0.951 | 1.000 | 3731 | tags=29%, list=17%, signal=35% |
| 1003 | GO\_SYNAPSE\_ASSEMBLY |  | 64 | -0.30 | -0.81 | 0.832 | 0.952 | 1.000 | 2934 | tags=25%, list=13%, signal=29% |
| 1004 | GO\_CELLULAR\_RESPONSE\_TO\_GLUCOSE\_STARVATION |  | 30 | -0.27 | -0.81 | 0.779 | 0.951 | 1.000 | 1344 | tags=13%, list=6%, signal=14% |
| 1005 | GO\_REGULATION\_OF\_MULTI\_ORGANISM\_PROCESS |  | 434 | -0.23 | -0.81 | 0.804 | 0.953 | 1.000 | 4067 | tags=25%, list=19%, signal=30% |
| 1006 | GO\_NEGATIVE\_REGULATION\_OF\_DEFENSE\_RESPONSE\_TO\_VIRUS |  | 17 | -0.36 | -0.81 | 0.748 | 0.952 | 1.000 | 2185 | tags=29%, list=10%, signal=33% |
| 1007 | GO\_TETRAPYRROLE\_METABOLIC\_PROCESS |  | 54 | -0.25 | -0.80 | 0.760 | 0.954 | 1.000 | 4590 | tags=30%, list=21%, signal=37% |
| 1008 | GO\_SPERMATID\_DIFFERENTIATION |  | 115 | -0.20 | -0.80 | 0.858 | 0.956 | 1.000 | 1488 | tags=10%, list=7%, signal=10% |
| 1009 | GO\_NEURON\_FATE\_SPECIFICATION |  | 30 | -0.35 | -0.80 | 0.750 | 0.955 | 1.000 | 2652 | tags=23%, list=12%, signal=27% |
| 1010 | GO\_AXONEMAL\_DYNEIN\_COMPLEX\_ASSEMBLY |  | 16 | -0.31 | -0.80 | 0.723 | 0.954 | 1.000 | 515 | tags=6%, list=2%, signal=6% |
| 1011 | GO\_PROTEIN\_ALKYLATION |  | 100 | -0.21 | -0.80 | 0.838 | 0.956 | 1.000 | 4452 | tags=27%, list=20%, signal=34% |
| 1012 | GO\_POSITIVE\_REGULATION\_OF\_INSULIN\_SECRETION\_INVOLVED\_IN\_CELLULAR\_RESPONSE\_TO\_GLUCOSE\_STIMULUS |  | 26 | -0.30 | -0.80 | 0.803 | 0.957 | 1.000 | 2063 | tags=15%, list=9%, signal=17% |
| 1013 | GO\_GLUTAMINE\_FAMILY\_AMINO\_ACID\_METABOLIC\_PROCESS |  | 61 | -0.28 | -0.80 | 0.852 | 0.956 | 1.000 | 2989 | tags=21%, list=14%, signal=25% |
| 1014 | GO\_ESTABLISHMENT\_OF\_EPITHELIAL\_CELL\_POLARITY |  | 22 | -0.30 | -0.80 | 0.763 | 0.955 | 1.000 | 5643 | tags=41%, list=26%, signal=55% |
| 1015 | GO\_PROTEIN\_K48\_LINKED\_DEUBIQUITINATION |  | 18 | -0.30 | -0.80 | 0.717 | 0.955 | 1.000 | 2465 | tags=17%, list=11%, signal=19% |
| 1016 | GO\_OVULATION |  | 17 | -0.37 | -0.80 | 0.768 | 0.958 | 1.000 | 1636 | tags=29%, list=8%, signal=32% |
| 1017 | GO\_CELLULAR\_CARBOHYDRATE\_BIOSYNTHETIC\_PROCESS |  | 49 | -0.27 | -0.79 | 0.843 | 0.965 | 1.000 | 2595 | tags=20%, list=12%, signal=23% |
| 1018 | GO\_POST\_GOLGI\_VESICLE\_MEDIATED\_TRANSPORT |  | 80 | -0.21 | -0.79 | 0.859 | 0.964 | 1.000 | 4353 | tags=23%, list=20%, signal=28% |
| 1019 | GO\_HOMOTYPIC\_CELL\_CELL\_ADHESION |  | 48 | -0.26 | -0.79 | 0.877 | 0.964 | 1.000 | 2097 | tags=17%, list=10%, signal=18% |
| 1020 | GO\_CARTILAGE\_DEVELOPMENT\_INVOLVED\_IN\_ENDOCHONDRAL\_BONE\_MORPHOGENESIS |  | 19 | -0.37 | -0.79 | 0.764 | 0.964 | 1.000 | 2014 | tags=21%, list=9%, signal=23% |
| 1021 | GO\_SPINAL\_CORD\_MOTOR\_NEURON\_DIFFERENTIATION |  | 33 | -0.32 | -0.79 | 0.779 | 0.963 | 1.000 | 2652 | tags=24%, list=12%, signal=28% |
| 1022 | GO\_POSITIVE\_REGULATION\_OF\_T\_CELL\_MEDIATED\_IMMUNITY |  | 30 | -0.33 | -0.79 | 0.734 | 0.965 | 1.000 | 2650 | tags=20%, list=12%, signal=23% |
| 1023 | GO\_PRODUCTION\_OF\_MOLECULAR\_MEDIATOR\_OF\_IMMUNE\_RESPONSE |  | 59 | -0.28 | -0.79 | 0.810 | 0.965 | 1.000 | 1944 | tags=19%, list=9%, signal=20% |
| 1024 | GO\_EPIBOLY |  | 19 | -0.34 | -0.79 | 0.789 | 0.965 | 1.000 | 1259 | tags=26%, list=6%, signal=28% |
| 1025 | GO\_ESTABLISHMENT\_OF\_PROTEIN\_LOCALIZATION\_TO\_MEMBRANE |  | 241 | -0.18 | -0.79 | 0.916 | 0.964 | 1.000 | 4570 | tags=20%, list=21%, signal=25% |
| 1026 | GO\_POSITIVE\_REGULATION\_OF\_ORGAN\_GROWTH |  | 36 | -0.31 | -0.79 | 0.843 | 0.964 | 1.000 | 3512 | tags=33%, list=16%, signal=40% |
| 1027 | GO\_MONOVALENT\_INORGANIC\_ANION\_HOMEOSTASIS |  | 18 | -0.32 | -0.79 | 0.810 | 0.963 | 1.000 | 1786 | tags=17%, list=8%, signal=18% |
| 1028 | GO\_REGULATION\_OF\_PRI\_MIRNA\_TRANSCRIPTION\_FROM\_RNA\_POLYMERASE\_II\_PROMOTER |  | 17 | -0.29 | -0.79 | 0.799 | 0.965 | 1.000 | 2932 | tags=24%, list=13%, signal=27% |
| 1029 | GO\_LIMBIC\_SYSTEM\_DEVELOPMENT |  | 98 | -0.24 | -0.79 | 0.868 | 0.967 | 1.000 | 4266 | tags=22%, list=20%, signal=28% |
| 1030 | GO\_ALCOHOL\_BIOSYNTHETIC\_PROCESS |  | 107 | -0.26 | -0.79 | 0.885 | 0.968 | 1.000 | 3080 | tags=21%, list=14%, signal=25% |
| 1031 | GO\_REGULATION\_OF\_STEROID\_METABOLIC\_PROCESS |  | 72 | -0.27 | -0.79 | 0.935 | 0.968 | 1.000 | 2474 | tags=15%, list=11%, signal=17% |
| 1032 | GO\_MICROVILLUS\_ORGANIZATION |  | 19 | -0.28 | -0.79 | 0.778 | 0.967 | 1.000 | 3731 | tags=26%, list=17%, signal=32% |
| 1033 | GO\_GLYCINE\_METABOLIC\_PROCESS |  | 15 | -0.35 | -0.78 | 0.767 | 0.971 | 1.000 | 3439 | tags=40%, list=16%, signal=47% |
| 1034 | GO\_MALE\_SEX\_DIFFERENTIATION |  | 141 | -0.22 | -0.78 | 0.964 | 0.972 | 1.000 | 4050 | tags=23%, list=19%, signal=28% |
| 1035 | GO\_REGULATION\_OF\_LIPOPROTEIN\_LIPASE\_ACTIVITY |  | 15 | -0.33 | -0.78 | 0.752 | 0.975 | 1.000 | 49 | tags=7%, list=0%, signal=7% |
| 1036 | GO\_VENOUS\_BLOOD\_VESSEL\_DEVELOPMENT |  | 15 | -0.35 | -0.78 | 0.805 | 0.975 | 1.000 | 1239 | tags=27%, list=6%, signal=28% |
| 1037 | GO\_NEGATIVE\_REGULATION\_OF\_ORGAN\_GROWTH |  | 21 | -0.29 | -0.78 | 0.799 | 0.975 | 1.000 | 2310 | tags=24%, list=11%, signal=27% |
| 1038 | GO\_NEGATIVE\_REGULATION\_OF\_COAGULATION |  | 47 | -0.29 | -0.78 | 0.920 | 0.975 | 1.000 | 880 | tags=15%, list=4%, signal=15% |
| 1039 | GO\_RESPONSE\_TO\_VIRUS |  | 228 | -0.30 | -0.78 | 0.742 | 0.977 | 1.000 | 2526 | tags=24%, list=12%, signal=27% |
| 1040 | GO\_REGULATION\_OF\_NECROTIC\_CELL\_DEATH |  | 25 | -0.28 | -0.78 | 0.782 | 0.978 | 1.000 | 5887 | tags=36%, list=27%, signal=49% |
| 1041 | GO\_SMOOTHENED\_SIGNALING\_PATHWAY |  | 68 | -0.28 | -0.77 | 0.820 | 0.980 | 1.000 | 2866 | tags=22%, list=13%, signal=25% |
| 1042 | GO\_MORPHOGENESIS\_OF\_AN\_EPITHELIAL\_FOLD |  | 15 | -0.37 | -0.77 | 0.836 | 0.983 | 1.000 | 2063 | tags=27%, list=9%, signal=29% |
| 1043 | GO\_EXECUTION\_PHASE\_OF\_APOPTOSIS |  | 52 | -0.22 | -0.77 | 0.882 | 0.983 | 1.000 | 5591 | tags=35%, list=26%, signal=46% |
| 1044 | GO\_CELL\_PROJECTION\_ASSEMBLY |  | 239 | -0.22 | -0.77 | 0.951 | 0.982 | 1.000 | 2866 | tags=16%, list=13%, signal=18% |
| 1045 | GO\_ZINC\_ION\_HOMEOSTASIS |  | 21 | -0.28 | -0.77 | 0.794 | 0.981 | 1.000 | 3493 | tags=24%, list=16%, signal=28% |
| 1046 | GO\_V\_D\_J\_RECOMBINATION |  | 15 | -0.28 | -0.77 | 0.761 | 0.983 | 1.000 | 8323 | tags=60%, list=38%, signal=97% |
| 1047 | GO\_POSITIVE\_REGULATION\_OF\_LEUKOCYTE\_DEGRANULATION |  | 18 | -0.33 | -0.77 | 0.792 | 0.983 | 1.000 | 128 | tags=11%, list=1%, signal=11% |
| 1048 | GO\_PROTEIN\_EXIT\_FROM\_ENDOPLASMIC\_RETICULUM |  | 18 | -0.28 | -0.77 | 0.701 | 0.982 | 1.000 | 9138 | tags=61%, list=42%, signal=105% |
| 1049 | GO\_REGULATION\_OF\_CATECHOLAMINE\_METABOLIC\_PROCESS |  | 17 | -0.32 | -0.77 | 0.778 | 0.981 | 1.000 | 5086 | tags=29%, list=23%, signal=38% |
| 1050 | GO\_RHYTHMIC\_BEHAVIOR |  | 17 | -0.32 | -0.77 | 0.843 | 0.981 | 1.000 | 5264 | tags=35%, list=24%, signal=47% |
| 1051 | GO\_REGULATION\_OF\_METANEPHROS\_DEVELOPMENT |  | 23 | -0.31 | -0.77 | 0.872 | 0.981 | 1.000 | 3512 | tags=35%, list=16%, signal=41% |
| 1052 | GO\_NEGATIVE\_REGULATION\_OF\_STEM\_CELL\_PROLIFERATION |  | 15 | -0.35 | -0.77 | 0.811 | 0.981 | 1.000 | 3859 | tags=47%, list=18%, signal=57% |
| 1053 | GO\_HISTONE\_METHYLATION |  | 75 | -0.22 | -0.77 | 0.859 | 0.980 | 1.000 | 3690 | tags=24%, list=17%, signal=29% |
| 1054 | GO\_ERAD\_PATHWAY |  | 71 | -0.21 | -0.77 | 0.810 | 0.979 | 1.000 | 5011 | tags=27%, list=23%, signal=35% |
| 1055 | GO\_VESICLE\_ORGANIZATION |  | 261 | -0.18 | -0.77 | 0.962 | 0.979 | 1.000 | 6819 | tags=35%, list=31%, signal=51% |
| 1056 | GO\_POSITIVE\_REGULATION\_OF\_GLIAL\_CELL\_DIFFERENTIATION |  | 31 | -0.28 | -0.77 | 0.859 | 0.979 | 1.000 | 3424 | tags=26%, list=16%, signal=31% |
| 1057 | GO\_CYTOKINE\_METABOLIC\_PROCESS |  | 17 | -0.34 | -0.77 | 0.832 | 0.978 | 1.000 | 2047 | tags=24%, list=9%, signal=26% |
| 1058 | GO\_REGULATION\_OF\_ACTIN\_NUCLEATION |  | 26 | -0.25 | -0.76 | 0.843 | 0.980 | 1.000 | 1741 | tags=15%, list=8%, signal=17% |
| 1059 | GO\_MEMBRANE\_BUDDING |  | 109 | -0.19 | -0.76 | 0.883 | 0.981 | 1.000 | 3880 | tags=18%, list=18%, signal=22% |
| 1060 | GO\_REGULATION\_OF\_AUTOPHAGOSOME\_ASSEMBLY |  | 34 | -0.24 | -0.76 | 0.846 | 0.980 | 1.000 | 2821 | tags=21%, list=13%, signal=24% |
| 1061 | GO\_REGULATION\_OF\_DNA\_TEMPLATED\_TRANSCRIPTION\_IN\_RESPONSE\_TO\_STRESS |  | 66 | -0.22 | -0.76 | 0.899 | 0.979 | 1.000 | 3947 | tags=21%, list=18%, signal=26% |
| 1062 | GO\_POST\_ANAL\_TAIL\_MORPHOGENESIS |  | 17 | -0.32 | -0.76 | 0.866 | 0.979 | 1.000 | 2148 | tags=24%, list=10%, signal=26% |
| 1063 | GO\_REGULATION\_OF\_RECEPTOR\_RECYCLING |  | 19 | -0.30 | -0.76 | 0.878 | 0.979 | 1.000 | 3880 | tags=37%, list=18%, signal=45% |
| 1064 | GO\_PHOTORECEPTOR\_CELL\_DIFFERENTIATION |  | 49 | -0.25 | -0.76 | 0.906 | 0.981 | 1.000 | 3977 | tags=20%, list=18%, signal=25% |
| 1065 | GO\_POSITIVE\_REGULATION\_OF\_INTERFERON\_ALPHA\_PRODUCTION |  | 16 | -0.40 | -0.76 | 0.733 | 0.982 | 1.000 | 2872 | tags=38%, list=13%, signal=43% |
| 1066 | GO\_DNA\_METHYLATION\_INVOLVED\_IN\_GAMETE\_GENERATION |  | 18 | -0.37 | -0.76 | 0.846 | 0.981 | 1.000 | 3363 | tags=33%, list=15%, signal=39% |
| 1067 | GO\_DICARBOXYLIC\_ACID\_TRANSPORT |  | 69 | -0.24 | -0.76 | 0.929 | 0.981 | 1.000 | 933 | tags=9%, list=4%, signal=9% |
| 1068 | GO\_CYTOKINE\_PRODUCTION\_INVOLVED\_IN\_IMMUNE\_RESPONSE |  | 17 | -0.33 | -0.76 | 0.846 | 0.980 | 1.000 | 3051 | tags=29%, list=14%, signal=34% |
| 1069 | GO\_ESTROGEN\_METABOLIC\_PROCESS |  | 21 | -0.31 | -0.76 | 0.802 | 0.980 | 1.000 | 449 | tags=10%, list=2%, signal=10% |
| 1070 | GO\_REGULATION\_OF\_RUFFLE\_ASSEMBLY |  | 19 | -0.29 | -0.76 | 0.868 | 0.980 | 1.000 | 3696 | tags=37%, list=17%, signal=44% |
| 1071 | GO\_PROTEIN\_HETEROOLIGOMERIZATION |  | 99 | -0.23 | -0.76 | 0.930 | 0.980 | 1.000 | 5926 | tags=38%, list=27%, signal=53% |
| 1072 | GO\_GLANDULAR\_EPITHELIAL\_CELL\_DIFFERENTIATION |  | 38 | -0.30 | -0.76 | 0.918 | 0.980 | 1.000 | 2270 | tags=21%, list=10%, signal=23% |
| 1073 | GO\_POSITIVE\_REGULATION\_OF\_BLOOD\_VESSEL\_ENDOTHELIAL\_CELL\_MIGRATION |  | 25 | -0.32 | -0.76 | 0.831 | 0.979 | 1.000 | 2653 | tags=28%, list=12%, signal=32% |
| 1074 | GO\_MICROTUBULE\_POLYMERIZATION |  | 27 | -0.27 | -0.76 | 0.823 | 0.979 | 1.000 | 1291 | tags=15%, list=6%, signal=16% |
| 1075 | GO\_ORGAN\_OR\_TISSUE\_SPECIFIC\_IMMUNE\_RESPONSE |  | 25 | -0.37 | -0.76 | 0.809 | 0.978 | 1.000 | 1051 | tags=20%, list=5%, signal=21% |
| 1076 | GO\_RESPONSE\_TO\_PROTOZOAN |  | 19 | -0.37 | -0.76 | 0.780 | 0.978 | 1.000 | 2238 | tags=26%, list=10%, signal=29% |
| 1077 | GO\_ANTIGEN\_PROCESSING\_AND\_PRESENTATION |  | 197 | -0.24 | -0.75 | 0.800 | 0.980 | 1.000 | 4023 | tags=27%, list=18%, signal=33% |
| 1078 | GO\_POSITIVE\_REGULATION\_OF\_IMMUNOGLOBULIN\_PRODUCTION |  | 31 | -0.31 | -0.75 | 0.828 | 0.979 | 1.000 | 1677 | tags=23%, list=8%, signal=24% |
| 1079 | GO\_REGULATION\_OF\_IMMUNOGLOBULIN\_PRODUCTION |  | 47 | -0.29 | -0.75 | 0.831 | 0.978 | 1.000 | 2703 | tags=23%, list=12%, signal=27% |
| 1080 | GO\_REGULATION\_OF\_NEURON\_APOPTOTIC\_PROCESS |  | 183 | -0.23 | -0.75 | 0.957 | 0.978 | 1.000 | 4191 | tags=25%, list=19%, signal=31% |
| 1081 | GO\_REGULATION\_OF\_HYDROGEN\_PEROXIDE\_INDUCED\_CELL\_DEATH |  | 17 | -0.28 | -0.75 | 0.855 | 0.978 | 1.000 | 5727 | tags=47%, list=26%, signal=64% |
| 1082 | GO\_MEMBRANE\_LIPID\_CATABOLIC\_PROCESS |  | 22 | -0.28 | -0.75 | 0.839 | 0.977 | 1.000 | 3869 | tags=23%, list=18%, signal=28% |
| 1083 | GO\_GRANULOCYTE\_MIGRATION |  | 66 | -0.35 | -0.75 | 0.819 | 0.977 | 1.000 | 886 | tags=18%, list=4%, signal=19% |
| 1084 | GO\_VESICLE\_COATING |  | 72 | -0.21 | -0.75 | 0.847 | 0.976 | 1.000 | 3405 | tags=18%, list=16%, signal=21% |
| 1085 | GO\_LABYRINTHINE\_LAYER\_BLOOD\_VESSEL\_DEVELOPMENT |  | 18 | -0.29 | -0.75 | 0.857 | 0.977 | 1.000 | 4779 | tags=44%, list=22%, signal=57% |
| 1086 | GO\_C21\_STEROID\_HORMONE\_METABOLIC\_PROCESS |  | 23 | -0.32 | -0.75 | 0.820 | 0.976 | 1.000 | 6930 | tags=57%, list=32%, signal=83% |
| 1087 | GO\_STRIATUM\_DEVELOPMENT |  | 16 | -0.31 | -0.75 | 0.865 | 0.975 | 1.000 | 2503 | tags=31%, list=12%, signal=35% |
| 1088 | GO\_NUCLEOTIDE\_TRANSPORT |  | 24 | -0.25 | -0.75 | 0.841 | 0.976 | 1.000 | 4122 | tags=21%, list=19%, signal=26% |
| 1089 | GO\_NEGATIVE\_REGULATION\_OF\_CHONDROCYTE\_DIFFERENTIATION |  | 19 | -0.36 | -0.75 | 0.814 | 0.977 | 1.000 | 2090 | tags=26%, list=10%, signal=29% |
| 1090 | GO\_HEMATOPOIETIC\_PROGENITOR\_CELL\_DIFFERENTIATION |  | 96 | -0.24 | -0.75 | 0.963 | 0.976 | 1.000 | 2994 | tags=19%, list=14%, signal=22% |
| 1091 | GO\_SPERMATID\_NUCLEUS\_DIFFERENTIATION |  | 17 | -0.23 | -0.75 | 0.761 | 0.978 | 1.000 | 10059 | tags=65%, list=46%, signal=120% |
| 1092 | GO\_CILIUM\_ORGANIZATION |  | 164 | -0.23 | -0.75 | 0.893 | 0.978 | 1.000 | 4943 | tags=25%, list=23%, signal=32% |
| 1093 | GO\_ENTEROENDOCRINE\_CELL\_DIFFERENTIATION |  | 19 | -0.32 | -0.75 | 0.870 | 0.978 | 1.000 | 2270 | tags=26%, list=10%, signal=29% |
| 1094 | GO\_METENCEPHALON\_DEVELOPMENT |  | 98 | -0.23 | -0.74 | 0.873 | 0.978 | 1.000 | 3597 | tags=19%, list=17%, signal=23% |
| 1095 | GO\_POSITIVE\_REGULATION\_OF\_CIRCADIAN\_RHYTHM |  | 20 | -0.28 | -0.74 | 0.847 | 0.978 | 1.000 | 6333 | tags=35%, list=29%, signal=49% |
| 1096 | GO\_REGULATION\_OF\_CELLULAR\_RESPIRATION |  | 22 | -0.27 | -0.74 | 0.859 | 0.977 | 1.000 | 2063 | tags=18%, list=9%, signal=20% |
| 1097 | GO\_NEGATIVE\_REGULATION\_OF\_ERBB\_SIGNALING\_PATHWAY |  | 44 | -0.23 | -0.74 | 0.851 | 0.981 | 1.000 | 7137 | tags=48%, list=33%, signal=71% |
| 1098 | GO\_AROMATIC\_AMINO\_ACID\_FAMILY\_METABOLIC\_PROCESS |  | 26 | -0.31 | -0.74 | 0.878 | 0.981 | 1.000 | 2975 | tags=23%, list=14%, signal=27% |
| 1099 | GO\_MAMMARY\_GLAND\_MORPHOGENESIS |  | 38 | -0.28 | -0.74 | 0.915 | 0.981 | 1.000 | 3859 | tags=29%, list=18%, signal=35% |
| 1100 | GO\_NEGATIVE\_REGULATION\_OF\_ALPHA\_BETA\_T\_CELL\_DIFFERENTIATION |  | 15 | -0.31 | -0.74 | 0.815 | 0.981 | 1.000 | 2652 | tags=20%, list=12%, signal=23% |
| 1101 | GO\_SPINAL\_CORD\_PATTERNING |  | 23 | -0.30 | -0.74 | 0.809 | 0.981 | 1.000 | 3560 | tags=26%, list=16%, signal=31% |
| 1102 | GO\_COPPER\_ION\_HOMEOSTASIS |  | 16 | -0.28 | -0.74 | 0.830 | 0.980 | 1.000 | 4761 | tags=38%, list=22%, signal=48% |
| 1103 | GO\_VENTRAL\_SPINAL\_CORD\_INTERNEURON\_DIFFERENTIATION |  | 16 | -0.31 | -0.74 | 0.808 | 0.979 | 1.000 | 3560 | tags=31%, list=16%, signal=37% |
| 1104 | GO\_REGULATION\_OF\_CARDIAC\_MUSCLE\_TISSUE\_DEVELOPMENT |  | 46 | -0.29 | -0.74 | 0.880 | 0.980 | 1.000 | 2762 | tags=26%, list=13%, signal=30% |
| 1105 | GO\_POSITIVE\_REGULATION\_OF\_ORGANIC\_ACID\_TRANSPORT |  | 28 | -0.29 | -0.74 | 0.929 | 0.980 | 1.000 | 3160 | tags=25%, list=15%, signal=29% |
| 1106 | GO\_POSITIVE\_REGULATION\_OF\_LEUKOCYTE\_MIGRATION |  | 105 | -0.31 | -0.74 | 0.870 | 0.979 | 1.000 | 2333 | tags=26%, list=11%, signal=29% |
| 1107 | GO\_PHOTORECEPTOR\_CELL\_MAINTENANCE |  | 33 | -0.25 | -0.73 | 0.879 | 0.981 | 1.000 | 975 | tags=9%, list=4%, signal=10% |
| 1108 | GO\_CIRCADIAN\_REGULATION\_OF\_GENE\_EXPRESSION |  | 53 | -0.22 | -0.73 | 0.933 | 0.983 | 1.000 | 3044 | tags=23%, list=14%, signal=26% |
| 1109 | GO\_ANDROGEN\_METABOLIC\_PROCESS |  | 29 | -0.29 | -0.73 | 0.862 | 0.982 | 1.000 | 4248 | tags=38%, list=20%, signal=47% |
| 1110 | GO\_REGULATION\_OF\_COLLATERAL\_SPROUTING |  | 16 | -0.34 | -0.73 | 0.899 | 0.982 | 1.000 | 1185 | tags=19%, list=5%, signal=20% |
| 1111 | GO\_MODULATION\_BY\_HOST\_OF\_VIRAL\_PROCESS |  | 17 | -0.29 | -0.73 | 0.824 | 0.982 | 1.000 | 65 | tags=6%, list=0%, signal=6% |
| 1112 | GO\_AROMATIC\_AMINO\_ACID\_FAMILY\_CATABOLIC\_PROCESS |  | 18 | -0.35 | -0.73 | 0.872 | 0.981 | 1.000 | 2814 | tags=28%, list=13%, signal=32% |
| 1113 | GO\_MORPHOGENESIS\_OF\_A\_POLARIZED\_EPITHELIUM |  | 27 | -0.29 | -0.73 | 0.865 | 0.981 | 1.000 | 5095 | tags=37%, list=23%, signal=48% |
| 1114 | GO\_OXIDATIVE\_PHOSPHORYLATION |  | 77 | -0.21 | -0.73 | 0.702 | 0.981 | 1.000 | 7663 | tags=32%, list=35%, signal=50% |
| 1115 | GO\_NEGATIVE\_REGULATION\_OF\_SMOOTHENED\_SIGNALING\_PATHWAY |  | 25 | -0.30 | -0.73 | 0.907 | 0.980 | 1.000 | 3424 | tags=32%, list=16%, signal=38% |
| 1116 | GO\_MESODERM\_MORPHOGENESIS |  | 63 | -0.24 | -0.73 | 0.939 | 0.979 | 1.000 | 5059 | tags=35%, list=23%, signal=45% |
| 1117 | GO\_CELLULAR\_COMPONENT\_DISASSEMBLY\_INVOLVED\_IN\_EXECUTION\_PHASE\_OF\_APOPTOSIS |  | 41 | -0.23 | -0.73 | 0.916 | 0.978 | 1.000 | 5591 | tags=34%, list=26%, signal=46% |
| 1118 | GO\_REGULATION\_OF\_INTERFERON\_ALPHA\_PRODUCTION |  | 19 | -0.38 | -0.73 | 0.755 | 0.978 | 1.000 | 3541 | tags=37%, list=16%, signal=44% |
| 1119 | GO\_NEGATIVE\_REGULATION\_OF\_OXIDATIVE\_STRESS\_INDUCED\_INTRINSIC\_APOPTOTIC\_SIGNALING\_PATHWAY |  | 20 | -0.26 | -0.73 | 0.853 | 0.977 | 1.000 | 5887 | tags=45%, list=27%, signal=62% |
| 1120 | GO\_NEGATIVE\_REGULATION\_OF\_LEUKOCYTE\_PROLIFERATION |  | 66 | -0.29 | -0.73 | 0.886 | 0.981 | 1.000 | 2047 | tags=20%, list=9%, signal=22% |
| 1121 | GO\_POSITIVE\_REGULATION\_OF\_INTERFERON\_BETA\_PRODUCTION |  | 30 | -0.32 | -0.72 | 0.798 | 0.981 | 1.000 | 2872 | tags=27%, list=13%, signal=31% |
| 1122 | GO\_DORSAL\_VENTRAL\_PATTERN\_FORMATION |  | 85 | -0.26 | -0.72 | 0.924 | 0.981 | 1.000 | 3856 | tags=27%, list=18%, signal=33% |
| 1123 | GO\_BRANCHING\_INVOLVED\_IN\_SALIVARY\_GLAND\_MORPHOGENESIS |  | 16 | -0.31 | -0.72 | 0.900 | 0.980 | 1.000 | 1045 | tags=13%, list=5%, signal=13% |
| 1124 | GO\_GLUTAMATE\_METABOLIC\_PROCESS |  | 26 | -0.28 | -0.72 | 0.928 | 0.980 | 1.000 | 2657 | tags=15%, list=12%, signal=18% |
| 1125 | GO\_REGULATION\_OF\_ICOSANOID\_SECRETION |  | 19 | -0.31 | -0.72 | 0.928 | 0.980 | 1.000 | 2643 | tags=21%, list=12%, signal=24% |
| 1126 | GO\_IONOTROPIC\_GLUTAMATE\_RECEPTOR\_SIGNALING\_PATHWAY |  | 23 | -0.26 | -0.72 | 0.835 | 0.980 | 1.000 | 8743 | tags=57%, list=40%, signal=94% |
| 1127 | GO\_RESPONSE\_TO\_ENDOPLASMIC\_RETICULUM\_STRESS |  | 220 | -0.18 | -0.72 | 0.957 | 0.979 | 1.000 | 4003 | tags=20%, list=18%, signal=25% |
| 1128 | GO\_PHAGOSOME\_MATURATION |  | 35 | -0.26 | -0.72 | 0.917 | 0.978 | 1.000 | 3792 | tags=31%, list=17%, signal=38% |
| 1129 | GO\_REGULATION\_OF\_INTERLEUKIN\_1\_PRODUCTION |  | 53 | -0.30 | -0.72 | 0.856 | 0.978 | 1.000 | 3026 | tags=26%, list=14%, signal=31% |
| 1130 | GO\_INNERVATION |  | 22 | -0.30 | -0.72 | 0.914 | 0.980 | 1.000 | 688 | tags=14%, list=3%, signal=14% |
| 1131 | GO\_REGULATION\_OF\_GENE\_EXPRESSION\_BY\_GENETIC\_IMPRINTING |  | 15 | -0.31 | -0.72 | 0.874 | 0.979 | 1.000 | 3363 | tags=20%, list=15%, signal=24% |
| 1132 | GO\_RECEPTOR\_CATABOLIC\_PROCESS |  | 16 | -0.30 | -0.72 | 0.846 | 0.979 | 1.000 | 5992 | tags=50%, list=28%, signal=69% |
| 1133 | GO\_LIPOPROTEIN\_METABOLIC\_PROCESS |  | 121 | -0.20 | -0.72 | 0.954 | 0.978 | 1.000 | 6119 | tags=31%, list=28%, signal=43% |
| 1134 | GO\_NEGATIVE\_REGULATION\_OF\_ORGANIC\_ACID\_TRANSPORT |  | 17 | -0.31 | -0.72 | 0.888 | 0.978 | 1.000 | 2643 | tags=24%, list=12%, signal=27% |
| 1135 | GO\_REGULATION\_OF\_CYTOKINE\_BIOSYNTHETIC\_PROCESS |  | 91 | -0.28 | -0.72 | 0.858 | 0.978 | 1.000 | 2486 | tags=16%, list=11%, signal=19% |
| 1136 | GO\_RESPONSE\_TO\_ATP |  | 28 | -0.31 | -0.72 | 0.857 | 0.978 | 1.000 | 4003 | tags=36%, list=18%, signal=44% |
| 1137 | GO\_LENS\_MORPHOGENESIS\_IN\_CAMERA\_TYPE\_EYE |  | 17 | -0.30 | -0.72 | 0.904 | 0.978 | 1.000 | 5423 | tags=41%, list=25%, signal=55% |
| 1138 | GO\_ORGANIC\_HYDROXY\_COMPOUND\_CATABOLIC\_PROCESS |  | 71 | -0.24 | -0.72 | 0.935 | 0.977 | 1.000 | 3474 | tags=20%, list=16%, signal=23% |
| 1139 | GO\_DNA\_METHYLATION\_OR\_DEMETHYLATION |  | 55 | -0.25 | -0.71 | 0.946 | 0.979 | 1.000 | 4798 | tags=33%, list=22%, signal=42% |
| 1140 | GO\_MEMBRANE\_LIPID\_BIOSYNTHETIC\_PROCESS |  | 103 | -0.22 | -0.71 | 0.973 | 0.980 | 1.000 | 3823 | tags=21%, list=18%, signal=26% |
| 1141 | GO\_PROTEIN\_TRANSPORT\_ALONG\_MICROTUBULE |  | 25 | -0.28 | -0.71 | 0.850 | 0.980 | 1.000 | 971 | tags=12%, list=4%, signal=13% |
| 1142 | GO\_CELL\_DIFFERENTIATION\_IN\_SPINAL\_CORD |  | 50 | -0.25 | -0.71 | 0.900 | 0.984 | 1.000 | 3597 | tags=20%, list=17%, signal=24% |
| 1143 | GO\_WNT\_SIGNALING\_PATHWAY\_CALCIUM\_MODULATING\_PATHWAY |  | 34 | -0.23 | -0.71 | 0.924 | 0.983 | 1.000 | 5293 | tags=29%, list=24%, signal=39% |
| 1144 | GO\_UTERUS\_DEVELOPMENT |  | 17 | -0.30 | -0.71 | 0.913 | 0.983 | 1.000 | 3859 | tags=35%, list=18%, signal=43% |
| 1145 | GO\_NEGATIVE\_REGULATION\_OF\_PROTEIN\_TYROSINE\_KINASE\_ACTIVITY |  | 19 | -0.27 | -0.71 | 0.845 | 0.982 | 1.000 | 3856 | tags=26%, list=18%, signal=32% |
| 1146 | GO\_SENSORY\_PERCEPTION\_OF\_LIGHT\_STIMULUS |  | 200 | -0.17 | -0.71 | 0.981 | 0.982 | 1.000 | 2230 | tags=9%, list=10%, signal=10% |
| 1147 | GO\_HISTONE\_DEUBIQUITINATION |  | 20 | -0.22 | -0.71 | 0.862 | 0.981 | 1.000 | 16948 | tags=100%, list=78%, signal=452% |
| 1148 | GO\_INTRASPECIES\_INTERACTION\_BETWEEN\_ORGANISMS |  | 45 | -0.25 | -0.70 | 0.920 | 0.981 | 1.000 | 2975 | tags=13%, list=14%, signal=15% |
| 1149 | GO\_ENTRAINMENT\_OF\_CIRCADIAN\_CLOCK |  | 25 | -0.24 | -0.70 | 0.880 | 0.981 | 1.000 | 7178 | tags=40%, list=33%, signal=60% |
| 1150 | GO\_HORMONE\_BIOSYNTHETIC\_PROCESS |  | 46 | -0.25 | -0.70 | 0.938 | 0.980 | 1.000 | 4248 | tags=30%, list=20%, signal=38% |
| 1151 | GO\_RESPONSE\_TO\_FUNGUS |  | 47 | -0.27 | -0.70 | 0.943 | 0.980 | 1.000 | 1243 | tags=11%, list=6%, signal=11% |
| 1152 | GO\_ANTIGEN\_PROCESSING\_AND\_PRESENTATION\_OF\_PEPTIDE\_ANTIGEN\_VIA\_MHC\_CLASS\_I |  | 87 | -0.24 | -0.70 | 0.792 | 0.981 | 1.000 | 4023 | tags=28%, list=18%, signal=34% |
| 1153 | GO\_REGULATION\_OF\_ACTIVIN\_RECEPTOR\_SIGNALING\_PATHWAY |  | 23 | -0.29 | -0.70 | 0.845 | 0.981 | 1.000 | 2554 | tags=17%, list=12%, signal=20% |
| 1154 | GO\_SULFATION |  | 15 | -0.33 | -0.70 | 0.872 | 0.980 | 1.000 | 4540 | tags=33%, list=21%, signal=42% |
| 1155 | GO\_NEGATIVE\_REGULATION\_OF\_BEHAVIOR |  | 17 | -0.23 | -0.70 | 0.826 | 0.981 | 1.000 | 9183 | tags=53%, list=42%, signal=92% |
| 1156 | GO\_TELENCEPHALON\_GLIAL\_CELL\_MIGRATION |  | 18 | -0.26 | -0.70 | 0.894 | 0.980 | 1.000 | 2870 | tags=17%, list=13%, signal=19% |
| 1157 | GO\_MESODERMAL\_CELL\_DIFFERENTIATION |  | 25 | -0.28 | -0.70 | 0.923 | 0.980 | 1.000 | 1004 | tags=16%, list=5%, signal=17% |
| 1158 | GO\_HEPARAN\_SULFATE\_PROTEOGLYCAN\_BIOSYNTHETIC\_PROCESS |  | 23 | -0.29 | -0.70 | 0.887 | 0.979 | 1.000 | 6159 | tags=48%, list=28%, signal=67% |
| 1159 | GO\_REGULATION\_OF\_VACUOLE\_ORGANIZATION |  | 40 | -0.21 | -0.70 | 0.933 | 0.981 | 1.000 | 2821 | tags=18%, list=13%, signal=20% |
| 1160 | GO\_SINGLE\_ORGANISM\_MEMBRANE\_BUDDING |  | 70 | -0.20 | -0.70 | 0.919 | 0.980 | 1.000 | 3405 | tags=19%, list=16%, signal=22% |
| 1161 | GO\_MAINTENANCE\_OF\_PROTEIN\_LOCALIZATION\_IN\_ORGANELLE |  | 28 | -0.21 | -0.69 | 0.889 | 0.981 | 1.000 | 5309 | tags=25%, list=24%, signal=33% |
| 1162 | GO\_NEGATIVE\_REGULATION\_OF\_BLOOD\_PRESSURE |  | 42 | -0.23 | -0.69 | 0.949 | 0.981 | 1.000 | 1508 | tags=10%, list=7%, signal=10% |
| 1163 | GO\_EPITHELIAL\_CELL\_MORPHOGENESIS |  | 42 | -0.26 | -0.69 | 0.984 | 0.981 | 1.000 | 4921 | tags=38%, list=23%, signal=49% |
| 1164 | GO\_INORGANIC\_ION\_IMPORT\_INTO\_CELL |  | 15 | -0.31 | -0.69 | 0.901 | 0.981 | 1.000 | 5019 | tags=40%, list=23%, signal=52% |
| 1165 | GO\_IRE1\_MEDIATED\_UNFOLDED\_PROTEIN\_RESPONSE |  | 51 | -0.22 | -0.69 | 0.836 | 0.981 | 1.000 | 3879 | tags=22%, list=18%, signal=26% |
| 1166 | GO\_SUBPALLIUM\_DEVELOPMENT |  | 22 | -0.29 | -0.69 | 0.930 | 0.980 | 1.000 | 2652 | tags=27%, list=12%, signal=31% |
| 1167 | GO\_REGULATION\_OF\_DEFENSE\_RESPONSE\_TO\_VIRUS\_BY\_VIRUS |  | 29 | -0.28 | -0.69 | 0.856 | 0.980 | 1.000 | 3908 | tags=31%, list=18%, signal=38% |
| 1168 | GO\_REGULATION\_OF\_INTERFERON\_BETA\_PRODUCTION |  | 43 | -0.28 | -0.69 | 0.828 | 0.980 | 1.000 | 3073 | tags=26%, list=14%, signal=30% |
| 1169 | GO\_CHONDROCYTE\_DEVELOPMENT |  | 21 | -0.36 | -0.69 | 0.819 | 0.979 | 1.000 | 2275 | tags=29%, list=10%, signal=32% |
| 1170 | GO\_APOPTOTIC\_DNA\_FRAGMENTATION |  | 15 | -0.29 | -0.69 | 0.901 | 0.979 | 1.000 | 3747 | tags=27%, list=17%, signal=32% |
| 1171 | GO\_REGULATION\_OF\_INTERLEUKIN\_1\_BETA\_PRODUCTION |  | 44 | -0.29 | -0.69 | 0.907 | 0.979 | 1.000 | 2773 | tags=27%, list=13%, signal=31% |
| 1172 | GO\_AMINO\_ACID\_BETAINE\_METABOLIC\_PROCESS |  | 18 | -0.28 | -0.69 | 0.937 | 0.979 | 1.000 | 2748 | tags=22%, list=13%, signal=25% |
| 1173 | GO\_MYELOID\_DENDRITIC\_CELL\_ACTIVATION |  | 25 | -0.34 | -0.68 | 0.867 | 0.980 | 1.000 | 1072 | tags=16%, list=5%, signal=17% |
| 1174 | GO\_NEURON\_FATE\_COMMITMENT |  | 65 | -0.25 | -0.68 | 0.966 | 0.980 | 1.000 | 2982 | tags=18%, list=14%, signal=21% |
| 1175 | GO\_RESPONSE\_TO\_TESTOSTERONE |  | 36 | -0.25 | -0.68 | 0.955 | 0.981 | 1.000 | 4109 | tags=28%, list=19%, signal=34% |
| 1176 | GO\_PLASMA\_MEMBRANE\_FUSION |  | 23 | -0.25 | -0.68 | 0.929 | 0.981 | 1.000 | 4145 | tags=22%, list=19%, signal=27% |
| 1177 | GO\_ESTABLISHMENT\_OR\_MAINTENANCE\_OF\_BIPOLAR\_CELL\_POLARITY |  | 34 | -0.25 | -0.68 | 0.948 | 0.980 | 1.000 | 4433 | tags=26%, list=20%, signal=33% |
| 1178 | GO\_NEGATIVE\_REGULATION\_OF\_CYTOKINE\_BIOSYNTHETIC\_PROCESS |  | 27 | -0.28 | -0.68 | 0.962 | 0.979 | 1.000 | 1004 | tags=7%, list=5%, signal=8% |
| 1179 | GO\_GRANULOCYTE\_ACTIVATION |  | 18 | -0.33 | -0.68 | 0.894 | 0.978 | 1.000 | 3238 | tags=28%, list=15%, signal=33% |
| 1180 | GO\_THIOESTER\_METABOLIC\_PROCESS |  | 73 | -0.21 | -0.68 | 0.982 | 0.978 | 1.000 | 2844 | tags=14%, list=13%, signal=16% |
| 1181 | GO\_ARP2\_3\_COMPLEX\_MEDIATED\_ACTIN\_NUCLEATION |  | 16 | -0.25 | -0.68 | 0.892 | 0.977 | 1.000 | 7004 | tags=44%, list=32%, signal=64% |
| 1182 | GO\_MACROMOLECULE\_DEACYLATION |  | 64 | -0.17 | -0.68 | 0.962 | 0.977 | 1.000 | 5599 | tags=27%, list=26%, signal=36% |
| 1183 | GO\_INTERACTION\_WITH\_HOST |  | 122 | -0.22 | -0.68 | 0.944 | 0.978 | 1.000 | 4679 | tags=29%, list=22%, signal=36% |
| 1184 | GO\_FOREBRAIN\_REGIONALIZATION |  | 22 | -0.31 | -0.68 | 0.940 | 0.977 | 1.000 | 5423 | tags=41%, list=25%, signal=54% |
| 1185 | GO\_RESPONSE\_TO\_FOLIC\_ACID |  | 15 | -0.29 | -0.68 | 0.911 | 0.978 | 1.000 | 2662 | tags=20%, list=12%, signal=23% |
| 1186 | GO\_REGULATION\_OF\_GRANULOCYTE\_MACROPHAGE\_COLONY\_STIMULATING\_FACTOR\_PRODUCTION |  | 15 | -0.35 | -0.68 | 0.941 | 0.977 | 1.000 | 1443 | tags=27%, list=7%, signal=29% |
| 1187 | GO\_POSITIVE\_REGULATION\_OF\_MESENCHYMAL\_CELL\_PROLIFERATION |  | 27 | -0.29 | -0.68 | 0.943 | 0.976 | 1.000 | 2447 | tags=22%, list=11%, signal=25% |
| 1188 | GO\_NEURON\_CELL\_CELL\_ADHESION |  | 16 | -0.31 | -0.68 | 0.964 | 0.976 | 1.000 | 519 | tags=13%, list=2%, signal=13% |
| 1189 | GO\_POSITIVE\_REGULATION\_OF\_INTERLEUKIN\_1\_PRODUCTION |  | 34 | -0.30 | -0.67 | 0.917 | 0.975 | 1.000 | 3026 | tags=29%, list=14%, signal=34% |
| 1190 | GO\_CELLULAR\_RESPONSE\_TO\_BIOTIC\_STIMULUS |  | 153 | -0.25 | -0.67 | 0.957 | 0.975 | 1.000 | 2927 | tags=20%, list=13%, signal=23% |
| 1191 | GO\_REGULATION\_OF\_HETEROTYPIC\_CELL\_CELL\_ADHESION |  | 18 | -0.30 | -0.67 | 0.970 | 0.975 | 1.000 | 2552 | tags=22%, list=12%, signal=25% |
| 1192 | GO\_NUCLEAR\_TRANSCRIBED\_MRNA\_CATABOLIC\_PROCESS\_NONSENSE\_MEDIATED\_DECAY |  | 98 | -0.15 | -0.67 | 0.819 | 0.975 | 1.000 | 6804 | tags=22%, list=31%, signal=33% |
| 1193 | GO\_SPERM\_MOTILITY |  | 40 | -0.19 | -0.67 | 0.891 | 0.975 | 1.000 | 9866 | tags=55%, list=45%, signal=100% |
| 1194 | GO\_REGULATION\_OF\_APPETITE |  | 23 | -0.26 | -0.67 | 0.918 | 0.975 | 1.000 | 7173 | tags=52%, list=33%, signal=78% |
| 1195 | GO\_SEX\_DETERMINATION |  | 21 | -0.27 | -0.67 | 0.958 | 0.976 | 1.000 | 3561 | tags=19%, list=16%, signal=23% |
| 1196 | GO\_POSITIVE\_REGULATION\_OF\_T\_HELPER\_CELL\_DIFFERENTIATION |  | 18 | -0.32 | -0.67 | 0.947 | 0.975 | 1.000 | 1578 | tags=22%, list=7%, signal=24% |
| 1197 | GO\_REGULATION\_OF\_T\_HELPER\_CELL\_DIFFERENTIATION |  | 26 | -0.30 | -0.67 | 0.928 | 0.975 | 1.000 | 1578 | tags=19%, list=7%, signal=21% |
| 1198 | GO\_STEROL\_BIOSYNTHETIC\_PROCESS |  | 41 | -0.26 | -0.67 | 0.903 | 0.974 | 1.000 | 5393 | tags=34%, list=25%, signal=45% |
| 1199 | GO\_POSITIVE\_REGULATION\_OF\_CYTOKINE\_BIOSYNTHETIC\_PROCESS |  | 57 | -0.29 | -0.67 | 0.885 | 0.975 | 1.000 | 2967 | tags=23%, list=14%, signal=26% |
| 1200 | GO\_REGULATION\_OF\_STEM\_CELL\_POPULATION\_MAINTENANCE |  | 17 | -0.25 | -0.67 | 0.891 | 0.974 | 1.000 | 4667 | tags=24%, list=21%, signal=30% |
| 1201 | GO\_ACTIVATION\_OF\_INNATE\_IMMUNE\_RESPONSE |  | 194 | -0.23 | -0.66 | 0.868 | 0.977 | 1.000 | 4066 | tags=28%, list=19%, signal=35% |
| 1202 | GO\_POSITIVE\_REGULATION\_OF\_T\_CELL\_MEDIATED\_CYTOTOXICITY |  | 15 | -0.29 | -0.65 | 0.947 | 0.981 | 1.000 | 1443 | tags=13%, list=7%, signal=14% |
| 1203 | GO\_DNA\_CATABOLIC\_PROCESS |  | 27 | -0.23 | -0.65 | 0.946 | 0.981 | 1.000 | 4141 | tags=22%, list=19%, signal=27% |
| 1204 | GO\_EYE\_PHOTORECEPTOR\_CELL\_DIFFERENTIATION |  | 41 | -0.23 | -0.65 | 0.977 | 0.981 | 1.000 | 3977 | tags=22%, list=18%, signal=27% |
| 1205 | GO\_REGULATION\_OF\_FEEDING\_BEHAVIOR |  | 19 | -0.23 | -0.65 | 0.859 | 0.980 | 1.000 | 11637 | tags=63%, list=53%, signal=136% |
| 1206 | GO\_NEGATIVE\_REGULATION\_OF\_DENDRITE\_MORPHOGENESIS |  | 15 | -0.26 | -0.65 | 0.921 | 0.983 | 1.000 | 4993 | tags=33%, list=23%, signal=43% |
| 1207 | GO\_COCHLEA\_DEVELOPMENT |  | 38 | -0.25 | -0.65 | 0.979 | 0.982 | 1.000 | 943 | tags=11%, list=4%, signal=11% |
| 1208 | GO\_RESPONSE\_TO\_FIBROBLAST\_GROWTH\_FACTOR |  | 111 | -0.19 | -0.64 | 0.994 | 0.982 | 1.000 | 6122 | tags=32%, list=28%, signal=44% |
| 1209 | GO\_REGULATION\_OF\_TYROSINE\_PHOSPHORYLATION\_OF\_STAT1\_PROTEIN |  | 16 | -0.29 | -0.64 | 0.935 | 0.982 | 1.000 | 3970 | tags=38%, list=18%, signal=46% |
| 1210 | GO\_CELL\_REDOX\_HOMEOSTASIS |  | 59 | -0.20 | -0.64 | 0.966 | 0.984 | 1.000 | 5548 | tags=27%, list=26%, signal=36% |
| 1211 | GO\_NATURAL\_KILLER\_CELL\_ACTIVATION\_INVOLVED\_IN\_IMMUNE\_RESPONSE |  | 24 | -0.23 | -0.63 | 0.895 | 0.985 | 1.000 | 1496 | tags=8%, list=7%, signal=9% |
| 1212 | GO\_CELLULAR\_RESPONSE\_TO\_TOPOLOGICALLY\_INCORRECT\_PROTEIN |  | 114 | -0.18 | -0.63 | 0.975 | 0.986 | 1.000 | 4037 | tags=19%, list=19%, signal=24% |
| 1213 | GO\_NERVE\_DEVELOPMENT |  | 63 | -0.22 | -0.62 | 0.995 | 0.989 | 1.000 | 4563 | tags=27%, list=21%, signal=34% |
| 1214 | GO\_REGULATION\_OF\_SYNAPTIC\_TRANSMISSION\_DOPAMINERGIC |  | 17 | -0.26 | -0.62 | 0.953 | 0.989 | 1.000 | 5440 | tags=35%, list=25%, signal=47% |
| 1215 | GO\_DENDRITIC\_CELL\_DIFFERENTIATION |  | 32 | -0.31 | -0.62 | 0.947 | 0.989 | 1.000 | 1072 | tags=16%, list=5%, signal=16% |
| 1216 | GO\_PEPTIDYL\_GLUTAMIC\_ACID\_MODIFICATION |  | 27 | -0.23 | -0.62 | 0.969 | 0.988 | 1.000 | 3065 | tags=15%, list=14%, signal=17% |
| 1217 | GO\_EATING\_BEHAVIOR |  | 29 | -0.23 | -0.62 | 0.980 | 0.988 | 1.000 | 7601 | tags=38%, list=35%, signal=58% |
| 1218 | GO\_THIOESTER\_BIOSYNTHETIC\_PROCESS |  | 46 | -0.20 | -0.61 | 0.990 | 0.989 | 1.000 | 2119 | tags=11%, list=10%, signal=12% |
| 1219 | GO\_REGULATION\_OF\_GLIAL\_CELL\_PROLIFERATION |  | 21 | -0.24 | -0.61 | 0.958 | 0.990 | 1.000 | 6777 | tags=38%, list=31%, signal=55% |
| 1220 | GO\_RESPONSE\_TO\_LEPTIN |  | 20 | -0.24 | -0.61 | 0.990 | 0.989 | 1.000 | 4050 | tags=20%, list=19%, signal=25% |
| 1221 | GO\_TRACHEA\_DEVELOPMENT |  | 20 | -0.23 | -0.61 | 0.979 | 0.988 | 1.000 | 5423 | tags=30%, list=25%, signal=40% |
| 1222 | GO\_FATTY\_ACID\_DERIVATIVE\_TRANSPORT |  | 20 | -0.28 | -0.61 | 0.990 | 0.988 | 1.000 | 3959 | tags=25%, list=18%, signal=31% |
| 1223 | GO\_ESTABLISHMENT\_OF\_PROTEIN\_LOCALIZATION\_TO\_GOLGI |  | 15 | -0.23 | -0.61 | 0.951 | 0.988 | 1.000 | 6964 | tags=40%, list=32%, signal=59% |
| 1224 | GO\_NEGATIVE\_REGULATION\_OF\_TRANSCRIPTION\_REGULATORY\_REGION\_DNA\_BINDING |  | 17 | -0.27 | -0.60 | 0.967 | 0.990 | 1.000 | 4572 | tags=29%, list=21%, signal=37% |
| 1225 | GO\_DETECTION\_OF\_OTHER\_ORGANISM |  | 17 | -0.28 | -0.60 | 0.966 | 0.990 | 1.000 | 3300 | tags=24%, list=15%, signal=28% |
| 1226 | GO\_INDOLE\_CONTAINING\_COMPOUND\_METABOLIC\_PROCESS |  | 25 | -0.25 | -0.59 | 0.975 | 0.990 | 1.000 | 3561 | tags=20%, list=16%, signal=24% |
| 1227 | GO\_HIGH\_DENSITY\_LIPOPROTEIN\_PARTICLE\_REMODELING |  | 15 | -0.26 | -0.59 | 0.982 | 0.991 | 1.000 | 198 | tags=7%, list=1%, signal=7% |
| 1228 | GO\_GLYCOSIDE\_METABOLIC\_PROCESS |  | 15 | -0.27 | -0.59 | 0.955 | 0.991 | 1.000 | 6479 | tags=40%, list=30%, signal=57% |
| 1229 | GO\_ENTRAINMENT\_OF\_CIRCADIAN\_CLOCK\_BY\_PHOTOPERIOD |  | 18 | -0.23 | -0.59 | 0.976 | 0.990 | 1.000 | 3044 | tags=17%, list=14%, signal=19% |
| 1230 | GO\_BINDING\_OF\_SPERM\_TO\_ZONA\_PELLUCIDA |  | 30 | -0.20 | -0.59 | 0.978 | 0.990 | 1.000 | 5512 | tags=30%, list=25%, signal=40% |
| 1231 | GO\_GRANULOCYTE\_DIFFERENTIATION |  | 15 | -0.24 | -0.58 | 0.978 | 0.990 | 1.000 | 6306 | tags=40%, list=29%, signal=56% |
| 1232 | GO\_RESPONSE\_TO\_ACETYLCHOLINE |  | 17 | -0.24 | -0.58 | 0.966 | 0.989 | 1.000 | 6848 | tags=41%, list=31%, signal=60% |
| 1233 | GO\_REGULATION\_OF\_INTERLEUKIN\_8\_SECRETION |  | 18 | -0.25 | -0.58 | 0.972 | 0.990 | 1.000 | 3859 | tags=22%, list=18%, signal=27% |
| 1234 | GO\_FERTILIZATION |  | 127 | -0.15 | -0.57 | 1.000 | 0.992 | 1.000 | 3846 | tags=17%, list=18%, signal=20% |
| 1235 | GO\_NUCLEOTIDE\_EXCISION\_REPAIR\_PREINCISION\_COMPLEX\_STABILIZATION |  | 20 | -0.20 | -0.56 | 0.958 | 0.993 | 1.000 | 8534 | tags=45%, list=39%, signal=74% |
| 1236 | GO\_BRANCH\_ELONGATION\_OF\_AN\_EPITHELIUM |  | 17 | -0.25 | -0.56 | 0.994 | 0.993 | 1.000 | 3859 | tags=29%, list=18%, signal=36% |
| 1237 | GO\_PROTEIN\_LOCALIZATION\_TO\_LYSOSOME |  | 16 | -0.22 | -0.55 | 0.952 | 0.992 | 1.000 | 6845 | tags=44%, list=31%, signal=64% |
| 1238 | GO\_NOSE\_DEVELOPMENT |  | 15 | -0.24 | -0.55 | 0.994 | 0.992 | 1.000 | 3572 | tags=27%, list=16%, signal=32% |
| 1239 | GO\_CATECHOLAMINE\_BIOSYNTHETIC\_PROCESS |  | 17 | -0.23 | -0.55 | 1.000 | 0.991 | 1.000 | 3724 | tags=24%, list=17%, signal=28% |
| 1240 | GO\_MALE\_GENITALIA\_DEVELOPMENT |  | 21 | -0.20 | -0.55 | 0.994 | 0.991 | 1.000 | 4910 | tags=29%, list=23%, signal=37% |
| 1241 | GO\_PROTEIN\_PALMITOYLATION |  | 23 | -0.19 | -0.53 | 0.984 | 0.993 | 1.000 | 2398 | tags=13%, list=11%, signal=15% |
| 1242 | GO\_ASPARTATE\_FAMILY\_AMINO\_ACID\_CATABOLIC\_PROCESS |  | 17 | -0.23 | -0.51 | 0.998 | 0.995 | 1.000 | 3366 | tags=24%, list=15%, signal=28% |
| 1243 | GO\_PHOTOPERIODISM |  | 23 | -0.19 | -0.49 | 0.996 | 0.996 | 1.000 | 3044 | tags=13%, list=14%, signal=15% |
Table: Gene sets enriched in phenotype **L (49 samples)**[plain text format]****

  
