# Supplementary material for: Identification and validation of key modules and hub genes associated with the pathological stage of oral squamous cell carcinoma by weighted gene co-expression network analysis
Source: PeerJ. 2020 Feb 4;8:e8505. doi: 10.7717/peerj.8505 (PMC7006519; doi:10.7717/peerj.8505)
Supplement: File S6 [file peerj-08-8505-s006.zip › my_analysis_201957_BP.Gsea.1570106074072/heat_map_corr_plot.html]

Heat map and correlation plot for input.201957.cls#H\_versus\_L  

Fig 1: heat\_map      
 Heat Map of the top 50 features for each phenotype in input.201957.cls#H\_versus\_L

  
  

Fig 2: Ranked Gene List Correlation Profile      
 Ranked list correlations for input.201957.cls#H\_versus\_L

  
  
    
