# Supplementary material for: Identification and validation of key modules and hub genes associated with the pathological stage of oral squamous cell carcinoma by weighted gene co-expression network analysis
Source: PeerJ. 2020 Feb 4;8:e8505. doi: 10.7717/peerj.8505 (PMC7006519; doi:10.7717/peerj.8505)
Supplement: File S6 [file peerj-08-8505-s006.zip › my_analysis_201957_BP.Gsea.1570106074072/index.html]

Index for xtools.gsea.Gsea my\_analysis\_201957\_BP.Gsea.1570106074072

### GSEA Report for Dataset input

#### Enrichment in phenotype: **H (48 samples)**

- 2152 / 3395 gene sets are upregulated in phenotype **H**- 0 gene sets are significant at FDR < 25%- 45 gene sets are significantly enriched at nominal pvalue < 1%- 208 gene sets are significantly enriched at nominal pvalue < 5%- Snapshot of enrichment results- Detailed enrichment results in html format- Detailed enrichment results in excel format (tab delimited text)- Guide to interpret results

#### Enrichment in phenotype: **L (49 samples)**

- 1243 / 3395 gene sets are upregulated in phenotype **L**- 136 gene sets are significantly enriched at FDR < 25%- 27 gene sets are significantly enriched at nominal pvalue < 1%- 143 gene sets are significantly enriched at nominal pvalue < 5%- Snapshot of enrichment results- Detailed enrichment results in html format- Detailed enrichment results in excel format (tab delimited text)- Guide to interpret results

#### Dataset details

- The dataset has 21753 features (genes)- No probe set => gene symbol collapsing was requested, so all 21753 features were used

#### Gene set details

- Gene set size filters (min=15, max=500) resulted in filtering out 1041 / 4436 gene sets- The remaining 3395 gene sets were used in the analysis- List of gene sets used and their sizes (restricted to features in the specified dataset)

#### Gene markers for the **H** *versus* **L** comparison

- The dataset has 21753 features (genes)- # of markers for phenotype **H**: 8013 (36.8% ) with correlation area 49.2%- # of markers for phenotype **L**: 13740 (63.2% ) with correlation area 50.8%- Detailed rank ordered gene list for all features in the dataset- Heat map and gene list correlation  profile for all features in the dataset- Buttefly plot of significant genes

#### Global statistics and plots

- Plot of p-values *vs.* NES- Global ES histogram

#### Other

- Parameters used for this analysis

#### Comments

- There were duplicate row identifiers in the specified dataset. One id was arbitarilly choosen. Details are below
  Generally, this is OK, but if you want to avoid this automagic, edit your dataset so that all row ids are unique
  # of row ids in original dataset: 21755
  # of row UNIQUE ids in original dataset: 21753
  # The duplicates were
  1-Mar
  2-Mar- Timestamp used as random seed: 1570106096659

---

Report: my\_analysis\_201957\_BP.Gsea.1570106074072.rpt   by user: admin

xtools.gsea.Gsea [Thu, Oct 3, '19 8 PM 34]

Website: www.gsea-msigdb.org/gsea
Questions & Suggestions: Contact page
