# Supplementary material for: Identification and validation of key modules and hub genes associated with the pathological stage of oral squamous cell carcinoma by weighted gene co-expression network analysis
Source: PeerJ. 2020 Feb 4;8:e8505. doi: 10.7717/peerj.8505 (PMC7006519; doi:10.7717/peerj.8505)
Supplement: File S6 [file peerj-08-8505-s006.zip › my_analysis_201957_KEGG.Gsea.1570105865824/gsea_report_for_H_1570105865824.html]

Report for H 1570105865824 [GSEA]

| GS  follow link to MSigDB | GS DETAILS | SIZE | ES | NES | NOM p-val | FDR q-val | FWER p-val | RANK AT MAX | LEADING EDGE || 1 | KEGG\_CARDIAC\_MUSCLE\_CONTRACTION | Details ... | 73 | 0.73 | 1.66 | 0.010 | 0.740 | 0.343 | 1290 | tags=25%, list=6%, signal=26% |
| 2 | KEGG\_ALDOSTERONE\_REGULATED\_SODIUM\_REABSORPTION | Details ... | 41 | 0.66 | 1.63 | 0.002 | 0.479 | 0.400 | 2019 | tags=37%, list=9%, signal=40% |
| 3 | KEGG\_TASTE\_TRANSDUCTION | Details ... | 43 | 0.55 | 1.61 | 0.031 | 0.404 | 0.483 | 3410 | tags=26%, list=16%, signal=30% |
| 4 | KEGG\_LEUKOCYTE\_TRANSENDOTHELIAL\_MIGRATION | Details ... | 113 | 0.60 | 1.59 | 0.006 | 0.398 | 0.572 | 2478 | tags=36%, list=11%, signal=41% |
| 5 | KEGG\_VASCULAR\_SMOOTH\_MUSCLE\_CONTRACTION | Details ... | 109 | 0.53 | 1.54 | 0.016 | 0.492 | 0.701 | 3860 | tags=39%, list=18%, signal=48% |
| 6 | KEGG\_DILATED\_CARDIOMYOPATHY | Details ... | 89 | 0.71 | 1.53 | 0.012 | 0.460 | 0.729 | 1614 | tags=36%, list=7%, signal=39% |
| 7 | KEGG\_PROXIMAL\_TUBULE\_BICARBONATE\_RECLAMATION | Details ... | 22 | 0.61 | 1.52 | 0.044 | 0.430 | 0.749 | 294 | tags=14%, list=1%, signal=14% |
| 8 | KEGG\_VIRAL\_MYOCARDITIS | Details ... | 67 | 0.65 | 1.52 | 0.021 | 0.379 | 0.749 | 2515 | tags=39%, list=12%, signal=44% |
| 9 | KEGG\_TIGHT\_JUNCTION | Details ... | 128 | 0.51 | 1.51 | 0.019 | 0.367 | 0.772 | 2637 | tags=28%, list=12%, signal=32% |
| 10 | KEGG\_CALCIUM\_SIGNALING\_PATHWAY | Details ... | 172 | 0.46 | 1.48 | 0.008 | 0.405 | 0.823 | 4527 | tags=35%, list=21%, signal=44% |
| 11 | KEGG\_PHOSPHATIDYLINOSITOL\_SIGNALING\_SYSTEM | Details ... | 75 | 0.44 | 1.46 | 0.029 | 0.454 | 0.867 | 3400 | tags=29%, list=16%, signal=35% |
| 12 | KEGG\_HYPERTROPHIC\_CARDIOMYOPATHY\_HCM | Details ... | 82 | 0.69 | 1.46 | 0.033 | 0.422 | 0.870 | 1481 | tags=35%, list=7%, signal=38% |
| 13 | KEGG\_ARRHYTHMOGENIC\_RIGHT\_VENTRICULAR\_CARDIOMYOPATHY\_ARVC | Details ... | 73 | 0.59 | 1.44 | 0.044 | 0.447 | 0.907 | 1481 | tags=23%, list=7%, signal=25% |
| 14 | KEGG\_GNRH\_SIGNALING\_PATHWAY | Details ... | 94 | 0.44 | 1.42 | 0.027 | 0.459 | 0.921 | 4674 | tags=37%, list=21%, signal=47% |
| 15 | KEGG\_INSULIN\_SIGNALING\_PATHWAY | Details ... | 135 | 0.41 | 1.42 | 0.018 | 0.460 | 0.926 | 3529 | tags=27%, list=16%, signal=33% |
| 16 | KEGG\_CELL\_ADHESION\_MOLECULES\_CAMS | Details ... | 126 | 0.57 | 1.41 | 0.057 | 0.441 | 0.931 | 3265 | tags=43%, list=15%, signal=50% |
| 17 | KEGG\_ASTHMA | Details ... | 27 | 0.67 | 1.36 | 0.129 | 0.596 | 0.964 | 3525 | tags=48%, list=16%, signal=57% |
| 18 | KEGG\_LINOLEIC\_ACID\_METABOLISM | Details ... | 24 | 0.68 | 1.32 | 0.139 | 0.702 | 0.976 | 2675 | tags=33%, list=12%, signal=38% |
| 19 | KEGG\_VALINE\_LEUCINE\_AND\_ISOLEUCINE\_DEGRADATION | Details ... | 44 | 0.50 | 1.29 | 0.160 | 0.763 | 0.985 | 4321 | tags=45%, list=20%, signal=57% |
| 20 | KEGG\_MTOR\_SIGNALING\_PATHWAY | Details ... | 50 | 0.41 | 1.28 | 0.085 | 0.795 | 0.992 | 1982 | tags=20%, list=9%, signal=22% |
| 21 | KEGG\_ARACHIDONIC\_ACID\_METABOLISM |  | 52 | 0.60 | 1.28 | 0.165 | 0.764 | 0.993 | 3068 | tags=37%, list=14%, signal=42% |
| 22 | KEGG\_INTESTINAL\_IMMUNE\_NETWORK\_FOR\_IGA\_PRODUCTION |  | 45 | 0.63 | 1.27 | 0.198 | 0.762 | 0.993 | 2515 | tags=47%, list=12%, signal=53% |
| 23 | KEGG\_LONG\_TERM\_DEPRESSION |  | 65 | 0.42 | 1.26 | 0.113 | 0.742 | 0.993 | 1922 | tags=18%, list=9%, signal=20% |
| 24 | KEGG\_PRIMARY\_IMMUNODEFICIENCY |  | 35 | 0.66 | 1.25 | 0.249 | 0.761 | 0.994 | 2786 | tags=46%, list=13%, signal=52% |
| 25 | KEGG\_MELANOGENESIS |  | 97 | 0.41 | 1.24 | 0.118 | 0.784 | 0.996 | 3736 | tags=29%, list=17%, signal=35% |
| 26 | KEGG\_ALPHA\_LINOLENIC\_ACID\_METABOLISM |  | 15 | 0.61 | 1.23 | 0.209 | 0.755 | 0.996 | 4736 | tags=47%, list=22%, signal=60% |
| 27 | KEGG\_VIBRIO\_CHOLERAE\_INFECTION |  | 52 | 0.38 | 1.23 | 0.185 | 0.753 | 0.997 | 4690 | tags=31%, list=22%, signal=39% |
| 28 | KEGG\_DRUG\_METABOLISM\_CYTOCHROME\_P450 |  | 59 | 0.59 | 1.23 | 0.222 | 0.730 | 0.997 | 4093 | tags=44%, list=19%, signal=54% |
| 29 | KEGG\_GLYCEROPHOSPHOLIPID\_METABOLISM |  | 66 | 0.39 | 1.21 | 0.128 | 0.746 | 1.000 | 3250 | tags=20%, list=15%, signal=23% |
| 30 | KEGG\_ETHER\_LIPID\_METABOLISM |  | 26 | 0.50 | 1.21 | 0.166 | 0.729 | 1.000 | 2992 | tags=27%, list=14%, signal=31% |
| 31 | KEGG\_AXON\_GUIDANCE |  | 127 | 0.37 | 1.20 | 0.158 | 0.726 | 1.000 | 4131 | tags=31%, list=19%, signal=39% |
| 32 | KEGG\_FC\_GAMMA\_R\_MEDIATED\_PHAGOCYTOSIS |  | 91 | 0.40 | 1.20 | 0.204 | 0.709 | 1.000 | 3654 | tags=31%, list=17%, signal=37% |
| 33 | KEGG\_LONG\_TERM\_POTENTIATION |  | 68 | 0.35 | 1.20 | 0.143 | 0.688 | 1.000 | 2946 | tags=22%, list=14%, signal=25% |
| 34 | KEGG\_FC\_EPSILON\_RI\_SIGNALING\_PATHWAY |  | 74 | 0.43 | 1.19 | 0.219 | 0.700 | 1.000 | 4469 | tags=38%, list=21%, signal=47% |
| 35 | KEGG\_HISTIDINE\_METABOLISM |  | 28 | 0.48 | 1.17 | 0.259 | 0.739 | 1.000 | 3475 | tags=43%, list=16%, signal=51% |
| 36 | KEGG\_B\_CELL\_RECEPTOR\_SIGNALING\_PATHWAY |  | 74 | 0.45 | 1.17 | 0.268 | 0.742 | 1.000 | 3822 | tags=36%, list=18%, signal=44% |
| 37 | KEGG\_ALZHEIMERS\_DISEASE |  | 154 | 0.29 | 1.15 | 0.220 | 0.758 | 1.000 | 2946 | tags=13%, list=14%, signal=15% |
| 38 | KEGG\_TYROSINE\_METABOLISM |  | 42 | 0.45 | 1.15 | 0.260 | 0.743 | 1.000 | 1686 | tags=14%, list=8%, signal=15% |
| 39 | KEGG\_PARKINSONS\_DISEASE |  | 113 | 0.29 | 1.15 | 0.296 | 0.737 | 1.000 | 1059 | tags=4%, list=5%, signal=5% |
| 40 | KEGG\_TYPE\_II\_DIABETES\_MELLITUS |  | 44 | 0.38 | 1.15 | 0.245 | 0.720 | 1.000 | 3529 | tags=27%, list=16%, signal=32% |
| 41 | KEGG\_COMPLEMENT\_AND\_COAGULATION\_CASCADES |  | 67 | 0.45 | 1.15 | 0.256 | 0.702 | 1.000 | 2890 | tags=30%, list=13%, signal=34% |
| 42 | KEGG\_ABC\_TRANSPORTERS |  | 42 | 0.45 | 1.15 | 0.263 | 0.686 | 1.000 | 4315 | tags=33%, list=20%, signal=42% |
| 43 | KEGG\_GLIOMA |  | 64 | 0.34 | 1.14 | 0.191 | 0.701 | 1.000 | 3529 | tags=27%, list=16%, signal=32% |
| 44 | KEGG\_FATTY\_ACID\_METABOLISM |  | 41 | 0.43 | 1.14 | 0.288 | 0.688 | 1.000 | 4910 | tags=49%, list=23%, signal=63% |
| 45 | KEGG\_PEROXISOME |  | 77 | 0.34 | 1.13 | 0.272 | 0.699 | 1.000 | 5805 | tags=47%, list=27%, signal=64% |
| 46 | KEGG\_PROPANOATE\_METABOLISM |  | 32 | 0.42 | 1.11 | 0.331 | 0.719 | 1.000 | 4112 | tags=44%, list=19%, signal=54% |
| 47 | KEGG\_ADIPOCYTOKINE\_SIGNALING\_PATHWAY |  | 66 | 0.34 | 1.11 | 0.261 | 0.715 | 1.000 | 2531 | tags=20%, list=12%, signal=22% |
| 48 | KEGG\_PHENYLALANINE\_METABOLISM |  | 17 | 0.51 | 1.11 | 0.342 | 0.705 | 1.000 | 3475 | tags=29%, list=16%, signal=35% |
| 49 | KEGG\_INOSITOL\_PHOSPHATE\_METABOLISM |  | 54 | 0.33 | 1.11 | 0.272 | 0.696 | 1.000 | 4595 | tags=35%, list=21%, signal=44% |
| 50 | KEGG\_RETINOL\_METABOLISM |  | 49 | 0.50 | 1.10 | 0.350 | 0.684 | 1.000 | 2675 | tags=24%, list=12%, signal=28% |
| 51 | KEGG\_ENDOMETRIAL\_CANCER |  | 52 | 0.34 | 1.10 | 0.303 | 0.675 | 1.000 | 4469 | tags=35%, list=21%, signal=43% |
| 52 | KEGG\_FOCAL\_ADHESION |  | 195 | 0.42 | 1.10 | 0.349 | 0.665 | 1.000 | 3273 | tags=29%, list=15%, signal=34% |
| 53 | KEGG\_ADHERENS\_JUNCTION |  | 67 | 0.32 | 1.09 | 0.288 | 0.672 | 1.000 | 3529 | tags=21%, list=16%, signal=25% |
| 54 | KEGG\_GAP\_JUNCTION |  | 87 | 0.33 | 1.09 | 0.299 | 0.661 | 1.000 | 3734 | tags=31%, list=17%, signal=37% |
| 55 | KEGG\_ACUTE\_MYELOID\_LEUKEMIA |  | 56 | 0.38 | 1.08 | 0.345 | 0.683 | 1.000 | 3529 | tags=29%, list=16%, signal=34% |
| 56 | KEGG\_PPAR\_SIGNALING\_PATHWAY |  | 67 | 0.39 | 1.07 | 0.302 | 0.683 | 1.000 | 4249 | tags=36%, list=20%, signal=44% |
| 57 | KEGG\_RENIN\_ANGIOTENSIN\_SYSTEM |  | 17 | 0.52 | 1.07 | 0.398 | 0.680 | 1.000 | 1565 | tags=24%, list=7%, signal=25% |
| 58 | KEGG\_TRYPTOPHAN\_METABOLISM |  | 39 | 0.41 | 1.07 | 0.322 | 0.671 | 1.000 | 3551 | tags=36%, list=16%, signal=43% |
| 59 | KEGG\_ALLOGRAFT\_REJECTION |  | 34 | 0.55 | 1.06 | 0.432 | 0.694 | 1.000 | 3941 | tags=44%, list=18%, signal=54% |
| 60 | KEGG\_AUTOIMMUNE\_THYROID\_DISEASE |  | 49 | 0.50 | 1.05 | 0.468 | 0.691 | 1.000 | 4080 | tags=33%, list=19%, signal=40% |
| 61 | KEGG\_REGULATION\_OF\_ACTIN\_CYTOSKELETON |  | 209 | 0.30 | 1.05 | 0.375 | 0.688 | 1.000 | 2258 | tags=16%, list=10%, signal=17% |
| 62 | KEGG\_PANTOTHENATE\_AND\_COA\_BIOSYNTHESIS |  | 16 | 0.45 | 1.04 | 0.415 | 0.692 | 1.000 | 2884 | tags=25%, list=13%, signal=29% |
| 63 | KEGG\_T\_CELL\_RECEPTOR\_SIGNALING\_PATHWAY |  | 106 | 0.40 | 1.04 | 0.443 | 0.695 | 1.000 | 3947 | tags=33%, list=18%, signal=40% |
| 64 | KEGG\_NEUROTROPHIN\_SIGNALING\_PATHWAY |  | 123 | 0.28 | 1.03 | 0.360 | 0.690 | 1.000 | 4682 | tags=33%, list=22%, signal=41% |
| 65 | KEGG\_PRIMARY\_BILE\_ACID\_BIOSYNTHESIS |  | 16 | 0.44 | 1.02 | 0.414 | 0.701 | 1.000 | 2142 | tags=25%, list=10%, signal=28% |
| 66 | KEGG\_OXIDATIVE\_PHOSPHORYLATION |  | 117 | 0.27 | 1.02 | 0.409 | 0.692 | 1.000 | 1055 | tags=4%, list=5%, signal=4% |
| 67 | KEGG\_NOTCH\_SIGNALING\_PATHWAY |  | 46 | 0.29 | 1.02 | 0.422 | 0.690 | 1.000 | 3672 | tags=20%, list=17%, signal=23% |
| 68 | KEGG\_O\_GLYCAN\_BIOSYNTHESIS |  | 26 | 0.45 | 1.02 | 0.455 | 0.683 | 1.000 | 3035 | tags=31%, list=14%, signal=36% |
| 69 | KEGG\_MAPK\_SIGNALING\_PATHWAY |  | 256 | 0.28 | 1.02 | 0.379 | 0.677 | 1.000 | 2946 | tags=18%, list=14%, signal=21% |
| 70 | KEGG\_REGULATION\_OF\_AUTOPHAGY |  | 34 | 0.31 | 1.02 | 0.442 | 0.669 | 1.000 | 5354 | tags=26%, list=25%, signal=35% |
| 71 | KEGG\_ECM\_RECEPTOR\_INTERACTION |  | 81 | 0.46 | 1.01 | 0.434 | 0.666 | 1.000 | 3354 | tags=38%, list=15%, signal=45% |
| 72 | KEGG\_ANTIGEN\_PROCESSING\_AND\_PRESENTATION |  | 80 | 0.40 | 1.01 | 0.466 | 0.664 | 1.000 | 2786 | tags=23%, list=13%, signal=26% |
| 73 | KEGG\_WNT\_SIGNALING\_PATHWAY |  | 145 | 0.31 | 1.00 | 0.452 | 0.669 | 1.000 | 2771 | tags=19%, list=13%, signal=21% |
| 74 | KEGG\_METABOLISM\_OF\_XENOBIOTICS\_BY\_CYTOCHROME\_P450 |  | 56 | 0.49 | 0.99 | 0.489 | 0.690 | 1.000 | 4093 | tags=39%, list=19%, signal=48% |
| 75 | KEGG\_NITROGEN\_METABOLISM |  | 22 | 0.40 | 0.98 | 0.474 | 0.693 | 1.000 | 2369 | tags=18%, list=11%, signal=20% |
| 76 | KEGG\_VEGF\_SIGNALING\_PATHWAY |  | 71 | 0.31 | 0.98 | 0.502 | 0.691 | 1.000 | 3860 | tags=28%, list=18%, signal=34% |
| 77 | KEGG\_PROSTATE\_CANCER |  | 89 | 0.29 | 0.97 | 0.495 | 0.690 | 1.000 | 3649 | tags=28%, list=17%, signal=34% |
| 78 | KEGG\_GLYCEROLIPID\_METABOLISM |  | 42 | 0.34 | 0.97 | 0.505 | 0.685 | 1.000 | 2964 | tags=24%, list=14%, signal=28% |
| 79 | KEGG\_NEUROACTIVE\_LIGAND\_RECEPTOR\_INTERACTION |  | 262 | 0.28 | 0.97 | 0.521 | 0.679 | 1.000 | 2537 | tags=11%, list=12%, signal=13% |
| 80 | KEGG\_TGF\_BETA\_SIGNALING\_PATHWAY |  | 82 | 0.33 | 0.97 | 0.494 | 0.681 | 1.000 | 2486 | tags=17%, list=11%, signal=19% |
| 81 | KEGG\_CHEMOKINE\_SIGNALING\_PATHWAY |  | 180 | 0.37 | 0.96 | 0.506 | 0.678 | 1.000 | 3171 | tags=29%, list=15%, signal=34% |
| 82 | KEGG\_HEMATOPOIETIC\_CELL\_LINEAGE |  | 84 | 0.41 | 0.95 | 0.504 | 0.688 | 1.000 | 2561 | tags=30%, list=12%, signal=34% |
| 83 | KEGG\_NON\_SMALL\_CELL\_LUNG\_CANCER |  | 54 | 0.28 | 0.95 | 0.566 | 0.685 | 1.000 | 4469 | tags=35%, list=21%, signal=44% |
| 84 | KEGG\_STEROID\_HORMONE\_BIOSYNTHESIS |  | 42 | 0.40 | 0.95 | 0.540 | 0.689 | 1.000 | 2774 | tags=24%, list=13%, signal=27% |
| 85 | KEGG\_GLYCOLYSIS\_GLUCONEOGENESIS |  | 60 | 0.33 | 0.93 | 0.531 | 0.705 | 1.000 | 4505 | tags=30%, list=21%, signal=38% |
| 86 | KEGG\_TYPE\_I\_DIABETES\_MELLITUS |  | 40 | 0.44 | 0.93 | 0.556 | 0.710 | 1.000 | 2665 | tags=38%, list=12%, signal=43% |
| 87 | KEGG\_LEISHMANIA\_INFECTION |  | 68 | 0.40 | 0.92 | 0.551 | 0.708 | 1.000 | 2665 | tags=31%, list=12%, signal=35% |
| 88 | KEGG\_BETA\_ALANINE\_METABOLISM |  | 22 | 0.33 | 0.92 | 0.586 | 0.715 | 1.000 | 3340 | tags=27%, list=15%, signal=32% |
| 89 | KEGG\_MELANOMA |  | 71 | 0.31 | 0.91 | 0.618 | 0.723 | 1.000 | 3529 | tags=25%, list=16%, signal=30% |
| 90 | KEGG\_APOPTOSIS |  | 86 | 0.29 | 0.91 | 0.608 | 0.719 | 1.000 | 2771 | tags=22%, list=13%, signal=25% |
| 91 | KEGG\_N\_GLYCAN\_BIOSYNTHESIS |  | 46 | 0.30 | 0.89 | 0.592 | 0.737 | 1.000 | 1678 | tags=11%, list=8%, signal=12% |
| 92 | KEGG\_VASOPRESSIN\_REGULATED\_WATER\_REABSORPTION |  | 44 | 0.29 | 0.89 | 0.631 | 0.738 | 1.000 | 3567 | tags=25%, list=16%, signal=30% |
| 93 | KEGG\_MATURITY\_ONSET\_DIABETES\_OF\_THE\_YOUNG |  | 24 | 0.36 | 0.88 | 0.606 | 0.754 | 1.000 | 843 | tags=8%, list=4%, signal=9% |
| 94 | KEGG\_RIBOFLAVIN\_METABOLISM |  | 16 | 0.33 | 0.86 | 0.670 | 0.768 | 1.000 | 2544 | tags=19%, list=12%, signal=21% |
| 95 | KEGG\_BUTANOATE\_METABOLISM |  | 33 | 0.33 | 0.86 | 0.641 | 0.762 | 1.000 | 4977 | tags=39%, list=23%, signal=51% |
| 96 | KEGG\_LYSOSOME |  | 114 | 0.28 | 0.86 | 0.633 | 0.756 | 1.000 | 4690 | tags=33%, list=22%, signal=42% |
| 97 | KEGG\_NATURAL\_KILLER\_CELL\_MEDIATED\_CYTOTOXICITY |  | 131 | 0.32 | 0.84 | 0.667 | 0.782 | 1.000 | 3909 | tags=27%, list=18%, signal=32% |
| 98 | KEGG\_JAK\_STAT\_SIGNALING\_PATHWAY |  | 151 | 0.26 | 0.76 | 0.867 | 0.914 | 1.000 | 2829 | tags=18%, list=13%, signal=20% |
| 99 | KEGG\_SELENOAMINO\_ACID\_METABOLISM |  | 25 | 0.28 | 0.76 | 0.815 | 0.915 | 1.000 | 3467 | tags=20%, list=16%, signal=24% |
| 100 | KEGG\_PYRUVATE\_METABOLISM |  | 38 | 0.24 | 0.73 | 0.829 | 0.943 | 1.000 | 3454 | tags=24%, list=16%, signal=28% |
| 101 | KEGG\_STARCH\_AND\_SUCROSE\_METABOLISM |  | 37 | 0.26 | 0.71 | 0.930 | 0.961 | 1.000 | 5087 | tags=32%, list=23%, signal=42% |
| 102 | KEGG\_GRAFT\_VERSUS\_HOST\_DISEASE |  | 37 | 0.35 | 0.71 | 0.804 | 0.953 | 1.000 | 2515 | tags=32%, list=12%, signal=37% |
| 103 | KEGG\_TOLL\_LIKE\_RECEPTOR\_SIGNALING\_PATHWAY |  | 98 | 0.27 | 0.70 | 0.874 | 0.954 | 1.000 | 3529 | tags=22%, list=16%, signal=27% |
| 104 | KEGG\_GLYCINE\_SERINE\_AND\_THREONINE\_METABOLISM |  | 31 | 0.30 | 0.70 | 0.883 | 0.946 | 1.000 | 1756 | tags=13%, list=8%, signal=14% |
| 105 | KEGG\_ALANINE\_ASPARTATE\_AND\_GLUTAMATE\_METABOLISM |  | 32 | 0.25 | 0.65 | 0.979 | 0.982 | 1.000 | 3478 | tags=16%, list=16%, signal=19% |
| 106 | KEGG\_GLUTATHIONE\_METABOLISM |  | 47 | 0.28 | 0.65 | 0.941 | 0.978 | 1.000 | 4155 | tags=32%, list=19%, signal=39% |
| 107 | KEGG\_ASCORBATE\_AND\_ALDARATE\_METABOLISM |  | 15 | 0.30 | 0.61 | 0.933 | 0.989 | 1.000 | 3686 | tags=33%, list=17%, signal=40% |
| 108 | KEGG\_GLYCOSPHINGOLIPID\_BIOSYNTHESIS\_LACTO\_AND\_NEOLACTO\_SERIES |  | 25 | 0.25 | 0.56 | 0.982 | 1.000 | 1.000 | 4858 | tags=36%, list=22%, signal=46% |
| 109 | KEGG\_PENTOSE\_AND\_GLUCURONATE\_INTERCONVERSIONS |  | 17 | 0.23 | 0.49 | 0.988 | 1.000 | 1.000 | 5730 | tags=41%, list=26%, signal=56% |
| 110 | KEGG\_RIBOSOME |  | 71 | 0.10 | 0.40 | 0.994 | 0.999 | 1.000 | 848 | tags=1%, list=4%, signal=1% |
Table: Gene sets enriched in phenotype **H (48 samples)**[plain text format]****

  
