# Supplementary material for: Identification and validation of key modules and hub genes associated with the pathological stage of oral squamous cell carcinoma by weighted gene co-expression network analysis
Source: PeerJ. 2020 Feb 4;8:e8505. doi: 10.7717/peerj.8505 (PMC7006519; doi:10.7717/peerj.8505)
Supplement: File S6 [file peerj-08-8505-s006.zip › my_analysis_201957_KEGG.Gsea.1570105865824/gsea_report_for_L_1570105865824.html]

Report for L 1570105865824 [GSEA]

| GS  follow link to MSigDB | GS DETAILS | SIZE | ES | NES | NOM p-val | FDR q-val | FWER p-val | RANK AT MAX | LEADING EDGE || 1 | KEGG\_SPLICEOSOME | Details ... | 114 | -0.51 | -1.67 | 0.033 | 0.368 | 0.320 | 7219 | tags=58%, list=33%, signal=86% |
| 2 | KEGG\_PYRIMIDINE\_METABOLISM | Details ... | 91 | -0.54 | -1.64 | 0.008 | 0.255 | 0.390 | 5098 | tags=53%, list=23%, signal=69% |
| 3 | KEGG\_PROTEASOME | Details ... | 41 | -0.63 | -1.61 | 0.035 | 0.253 | 0.504 | 5955 | tags=66%, list=27%, signal=91% |
| 4 | KEGG\_HOMOLOGOUS\_RECOMBINATION | Details ... | 28 | -0.68 | -1.54 | 0.019 | 0.371 | 0.706 | 4464 | tags=64%, list=21%, signal=81% |
| 5 | KEGG\_CELL\_CYCLE | Details ... | 122 | -0.59 | -1.53 | 0.040 | 0.332 | 0.746 | 3538 | tags=50%, list=16%, signal=59% |
| 6 | KEGG\_BASE\_EXCISION\_REPAIR | Details ... | 32 | -0.62 | -1.50 | 0.044 | 0.357 | 0.816 | 4926 | tags=59%, list=23%, signal=77% |
| 7 | KEGG\_RNA\_POLYMERASE | Details ... | 28 | -0.53 | -1.49 | 0.074 | 0.326 | 0.834 | 6885 | tags=68%, list=32%, signal=99% |
| 8 | KEGG\_BASAL\_TRANSCRIPTION\_FACTORS | Details ... | 32 | -0.51 | -1.49 | 0.094 | 0.291 | 0.835 | 5760 | tags=47%, list=26%, signal=64% |
| 9 | KEGG\_UBIQUITIN\_MEDIATED\_PROTEOLYSIS | Details ... | 129 | -0.39 | -1.47 | 0.051 | 0.313 | 0.877 | 5354 | tags=33%, list=25%, signal=44% |
| 10 | KEGG\_RNA\_DEGRADATION | Details ... | 56 | -0.47 | -1.46 | 0.067 | 0.302 | 0.890 | 3779 | tags=41%, list=17%, signal=50% |
| 11 | KEGG\_MISMATCH\_REPAIR | Details ... | 22 | -0.66 | -1.44 | 0.077 | 0.319 | 0.909 | 4368 | tags=59%, list=20%, signal=74% |
| 12 | KEGG\_DNA\_REPLICATION | Details ... | 36 | -0.69 | -1.42 | 0.098 | 0.331 | 0.929 | 3194 | tags=61%, list=15%, signal=72% |
| 13 | KEGG\_GLYCOSYLPHOSPHATIDYLINOSITOL\_GPI\_ANCHOR\_BIOSYNTHESIS | Details ... | 24 | -0.53 | -1.41 | 0.101 | 0.330 | 0.938 | 3557 | tags=38%, list=16%, signal=45% |
| 14 | KEGG\_OOCYTE\_MEIOSIS | Details ... | 107 | -0.40 | -1.41 | 0.046 | 0.310 | 0.939 | 3245 | tags=30%, list=15%, signal=35% |
| 15 | KEGG\_AMINOACYL\_TRNA\_BIOSYNTHESIS | Details ... | 37 | -0.50 | -1.38 | 0.140 | 0.349 | 0.956 | 5029 | tags=41%, list=23%, signal=53% |
| 16 | KEGG\_NUCLEOTIDE\_EXCISION\_REPAIR | Details ... | 43 | -0.52 | -1.35 | 0.152 | 0.388 | 0.973 | 5774 | tags=58%, list=27%, signal=79% |
| 17 | KEGG\_GLYOXYLATE\_AND\_DICARBOXYLATE\_METABOLISM | Details ... | 16 | -0.49 | -1.34 | 0.167 | 0.391 | 0.976 | 2079 | tags=25%, list=10%, signal=28% |
| 18 | KEGG\_GALACTOSE\_METABOLISM | Details ... | 25 | -0.45 | -1.29 | 0.125 | 0.502 | 0.993 | 7707 | tags=56%, list=35%, signal=87% |
| 19 | KEGG\_BLADDER\_CANCER | Details ... | 40 | -0.48 | -1.27 | 0.136 | 0.512 | 0.993 | 3299 | tags=45%, list=15%, signal=53% |
| 20 | KEGG\_RENAL\_CELL\_CARCINOMA | Details ... | 68 | -0.39 | -1.24 | 0.157 | 0.572 | 0.996 | 3933 | tags=35%, list=18%, signal=43% |
| 21 | KEGG\_P53\_SIGNALING\_PATHWAY |  | 65 | -0.43 | -1.23 | 0.166 | 0.584 | 0.996 | 2263 | tags=34%, list=10%, signal=38% |
| 22 | KEGG\_STEROID\_BIOSYNTHESIS |  | 15 | -0.56 | -1.22 | 0.192 | 0.571 | 0.996 | 192 | tags=20%, list=1%, signal=20% |
| 23 | KEGG\_PROGESTERONE\_MEDIATED\_OOCYTE\_MATURATION |  | 83 | -0.36 | -1.16 | 0.217 | 0.703 | 1.000 | 2942 | tags=31%, list=14%, signal=36% |
| 24 | KEGG\_CYSTEINE\_AND\_METHIONINE\_METABOLISM |  | 34 | -0.40 | -1.16 | 0.266 | 0.683 | 1.000 | 421 | tags=12%, list=2%, signal=12% |
| 25 | KEGG\_ERBB\_SIGNALING\_PATHWAY |  | 86 | -0.33 | -1.16 | 0.181 | 0.659 | 1.000 | 4205 | tags=35%, list=19%, signal=43% |
| 26 | KEGG\_PANCREATIC\_CANCER |  | 69 | -0.37 | -1.15 | 0.228 | 0.666 | 1.000 | 3245 | tags=35%, list=15%, signal=41% |
| 27 | KEGG\_FRUCTOSE\_AND\_MANNOSE\_METABOLISM |  | 33 | -0.38 | -1.13 | 0.278 | 0.698 | 1.000 | 3726 | tags=30%, list=17%, signal=37% |
| 28 | KEGG\_LYSINE\_DEGRADATION |  | 41 | -0.36 | -1.12 | 0.258 | 0.695 | 1.000 | 3366 | tags=29%, list=15%, signal=35% |
| 29 | KEGG\_PURINE\_METABOLISM |  | 148 | -0.32 | -1.11 | 0.279 | 0.687 | 1.000 | 3345 | tags=28%, list=15%, signal=33% |
| 30 | KEGG\_THYROID\_CANCER |  | 29 | -0.40 | -1.10 | 0.330 | 0.687 | 1.000 | 4779 | tags=45%, list=22%, signal=57% |
| 31 | KEGG\_BIOSYNTHESIS\_OF\_UNSATURATED\_FATTY\_ACIDS |  | 18 | -0.44 | -1.09 | 0.346 | 0.691 | 1.000 | 471 | tags=11%, list=2%, signal=11% |
| 32 | KEGG\_AMYOTROPHIC\_LATERAL\_SCLEROSIS\_ALS |  | 51 | -0.34 | -1.08 | 0.319 | 0.697 | 1.000 | 4473 | tags=27%, list=21%, signal=34% |
| 33 | KEGG\_CHRONIC\_MYELOID\_LEUKEMIA |  | 72 | -0.31 | -1.06 | 0.350 | 0.728 | 1.000 | 4779 | tags=46%, list=22%, signal=59% |
| 34 | KEGG\_PROTEIN\_EXPORT |  | 22 | -0.35 | -1.04 | 0.429 | 0.755 | 1.000 | 7945 | tags=50%, list=37%, signal=79% |
| 35 | KEGG\_RIG\_I\_LIKE\_RECEPTOR\_SIGNALING\_PATHWAY |  | 69 | -0.36 | -1.04 | 0.430 | 0.743 | 1.000 | 5666 | tags=39%, list=26%, signal=53% |
| 36 | KEGG\_SNARE\_INTERACTIONS\_IN\_VESICULAR\_TRANSPORT |  | 38 | -0.31 | -1.01 | 0.431 | 0.800 | 1.000 | 4145 | tags=26%, list=19%, signal=32% |
| 37 | KEGG\_PRION\_DISEASES |  | 34 | -0.36 | -0.99 | 0.458 | 0.808 | 1.000 | 899 | tags=12%, list=4%, signal=12% |
| 38 | KEGG\_PENTOSE\_PHOSPHATE\_PATHWAY |  | 26 | -0.37 | -0.99 | 0.463 | 0.801 | 1.000 | 4767 | tags=38%, list=22%, signal=49% |
| 39 | KEGG\_PATHWAYS\_IN\_CANCER |  | 319 | -0.30 | -0.98 | 0.479 | 0.797 | 1.000 | 3346 | tags=28%, list=15%, signal=32% |
| 40 | KEGG\_NOD\_LIKE\_RECEPTOR\_SIGNALING\_PATHWAY |  | 59 | -0.39 | -0.97 | 0.483 | 0.813 | 1.000 | 2927 | tags=22%, list=13%, signal=25% |
| 41 | KEGG\_DRUG\_METABOLISM\_OTHER\_ENZYMES |  | 38 | -0.35 | -0.95 | 0.529 | 0.843 | 1.000 | 3155 | tags=26%, list=15%, signal=31% |
| 42 | KEGG\_SPHINGOLIPID\_METABOLISM |  | 32 | -0.33 | -0.93 | 0.574 | 0.869 | 1.000 | 3112 | tags=25%, list=14%, signal=29% |
| 43 | KEGG\_PATHOGENIC\_ESCHERICHIA\_COLI\_INFECTION |  | 53 | -0.26 | -0.90 | 0.596 | 0.905 | 1.000 | 4387 | tags=26%, list=20%, signal=33% |
| 44 | KEGG\_ONE\_CARBON\_POOL\_BY\_FOLATE |  | 16 | -0.35 | -0.90 | 0.603 | 0.892 | 1.000 | 2470 | tags=38%, list=11%, signal=42% |
| 45 | KEGG\_SMALL\_CELL\_LUNG\_CANCER |  | 84 | -0.32 | -0.89 | 0.634 | 0.895 | 1.000 | 3446 | tags=32%, list=16%, signal=38% |
| 46 | KEGG\_ARGININE\_AND\_PROLINE\_METABOLISM |  | 49 | -0.30 | -0.84 | 0.789 | 0.997 | 1.000 | 1553 | tags=18%, list=7%, signal=20% |
| 47 | KEGG\_EPITHELIAL\_CELL\_SIGNALING\_IN\_HELICOBACTER\_PYLORI\_INFECTION |  | 66 | -0.27 | -0.84 | 0.766 | 0.978 | 1.000 | 4365 | tags=35%, list=20%, signal=43% |
| 48 | KEGG\_COLORECTAL\_CANCER |  | 62 | -0.27 | -0.83 | 0.746 | 0.962 | 1.000 | 3245 | tags=26%, list=15%, signal=30% |
| 49 | KEGG\_CYTOSOLIC\_DNA\_SENSING\_PATHWAY |  | 53 | -0.32 | -0.81 | 0.694 | 0.997 | 1.000 | 3346 | tags=23%, list=15%, signal=27% |
| 50 | KEGG\_PORPHYRIN\_AND\_CHLOROPHYLL\_METABOLISM |  | 30 | -0.31 | -0.80 | 0.735 | 0.992 | 1.000 | 4628 | tags=40%, list=21%, signal=51% |
| 51 | KEGG\_CITRATE\_CYCLE\_TCA\_CYCLE |  | 30 | -0.26 | -0.79 | 0.697 | 0.996 | 1.000 | 2503 | tags=10%, list=12%, signal=11% |
| 52 | KEGG\_ENDOCYTOSIS |  | 171 | -0.21 | -0.78 | 0.947 | 0.988 | 1.000 | 4113 | tags=27%, list=19%, signal=34% |
| 53 | KEGG\_HEDGEHOG\_SIGNALING\_PATHWAY |  | 53 | -0.31 | -0.78 | 0.807 | 0.980 | 1.000 | 3914 | tags=25%, list=18%, signal=30% |
| 54 | KEGG\_GLYCOSAMINOGLYCAN\_BIOSYNTHESIS\_HEPARAN\_SULFATE |  | 26 | -0.32 | -0.77 | 0.785 | 0.967 | 1.000 | 3002 | tags=27%, list=14%, signal=31% |
| 55 | KEGG\_CYTOKINE\_CYTOKINE\_RECEPTOR\_INTERACTION |  | 250 | -0.30 | -0.77 | 0.880 | 0.962 | 1.000 | 1025 | tags=15%, list=5%, signal=16% |
| 56 | KEGG\_GLYCOSAMINOGLYCAN\_BIOSYNTHESIS\_CHONDROITIN\_SULFATE |  | 22 | -0.36 | -0.76 | 0.769 | 0.955 | 1.000 | 4071 | tags=36%, list=19%, signal=45% |
| 57 | KEGG\_DORSO\_VENTRAL\_AXIS\_FORMATION |  | 23 | -0.27 | -0.73 | 0.921 | 0.976 | 1.000 | 5342 | tags=35%, list=25%, signal=46% |
| 58 | KEGG\_HUNTINGTONS\_DISEASE |  | 168 | -0.17 | -0.73 | 0.887 | 0.965 | 1.000 | 5864 | tags=26%, list=27%, signal=36% |
| 59 | KEGG\_NICOTINATE\_AND\_NICOTINAMIDE\_METABOLISM |  | 21 | -0.29 | -0.72 | 0.944 | 0.965 | 1.000 | 1432 | tags=19%, list=7%, signal=20% |
| 60 | KEGG\_SYSTEMIC\_LUPUS\_ERYTHEMATOSUS |  | 102 | -0.28 | -0.71 | 0.844 | 0.957 | 1.000 | 3426 | tags=29%, list=16%, signal=35% |
| 61 | KEGG\_TERPENOID\_BACKBONE\_BIOSYNTHESIS |  | 15 | -0.32 | -0.71 | 0.815 | 0.945 | 1.000 | 5073 | tags=33%, list=23%, signal=43% |
| 62 | KEGG\_AMINO\_SUGAR\_AND\_NUCLEOTIDE\_SUGAR\_METABOLISM |  | 42 | -0.22 | -0.68 | 0.943 | 0.962 | 1.000 | 4532 | tags=29%, list=21%, signal=36% |
| 63 | KEGG\_BASAL\_CELL\_CARCINOMA |  | 52 | -0.22 | -0.50 | 1.000 | 1.000 | 1.000 | 5467 | tags=31%, list=25%, signal=41% |
| 64 | KEGG\_OLFACTORY\_TRANSDUCTION |  | 111 | -0.11 | -0.50 | 0.985 | 1.000 | 1.000 | 11007 | tags=61%, list=51%, signal=123% |
| 65 | KEGG\_GLYCOSAMINOGLYCAN\_DEGRADATION |  | 20 | -0.16 | -0.41 | 1.000 | 0.999 | 1.000 | 18191 | tags=100%, list=84%, signal=610% |
Table: Gene sets enriched in phenotype **L (49 samples)**[plain text format]****

  
