# Supplementary material for: Identification and validation of key modules and hub genes associated with the pathological stage of oral squamous cell carcinoma by weighted gene co-expression network analysis
Source: PeerJ. 2020 Feb 4;8:e8505. doi: 10.7717/peerj.8505 (PMC7006519; doi:10.7717/peerj.8505)
Supplement: File S6 [file peerj-08-8505-s006.zip › my_analysis_205382_KEGG.Gsea.1570105897088/gsea_report_for_H_1570105897088.html]

Report for H 1570105897088 [GSEA]

| GS  follow link to MSigDB | GS DETAILS | SIZE | ES | NES | NOM p-val | FDR q-val | FWER p-val | RANK AT MAX | LEADING EDGE || 1 | KEGG\_OXIDATIVE\_PHOSPHORYLATION | Details ... | 117 | 0.45 | 1.72 | 0.018 | 0.302 | 0.200 | 6939 | tags=50%, list=32%, signal=74% |
| 2 | KEGG\_PARKINSONS\_DISEASE | Details ... | 113 | 0.39 | 1.60 | 0.037 | 0.582 | 0.507 | 7157 | tags=49%, list=33%, signal=72% |
| 3 | KEGG\_TYROSINE\_METABOLISM | Details ... | 42 | 0.63 | 1.60 | 0.016 | 0.401 | 0.514 | 3051 | tags=33%, list=14%, signal=39% |
| 4 | KEGG\_MATURITY\_ONSET\_DIABETES\_OF\_THE\_YOUNG | Details ... | 24 | 0.65 | 1.58 | 0.028 | 0.353 | 0.562 | 1527 | tags=17%, list=7%, signal=18% |
| 5 | KEGG\_VALINE\_LEUCINE\_AND\_ISOLEUCINE\_DEGRADATION | Details ... | 44 | 0.59 | 1.54 | 0.017 | 0.407 | 0.670 | 3321 | tags=45%, list=15%, signal=54% |
| 6 | KEGG\_DRUG\_METABOLISM\_CYTOCHROME\_P450 | Details ... | 59 | 0.75 | 1.54 | 0.017 | 0.357 | 0.686 | 2510 | tags=44%, list=12%, signal=50% |
| 7 | KEGG\_FATTY\_ACID\_METABOLISM | Details ... | 41 | 0.57 | 1.50 | 0.035 | 0.422 | 0.777 | 3114 | tags=41%, list=14%, signal=48% |
| 8 | KEGG\_PROPANOATE\_METABOLISM | Details ... | 32 | 0.55 | 1.50 | 0.061 | 0.386 | 0.790 | 4393 | tags=53%, list=20%, signal=66% |
| 9 | KEGG\_ARACHIDONIC\_ACID\_METABOLISM | Details ... | 52 | 0.70 | 1.50 | 0.033 | 0.347 | 0.793 | 2316 | tags=42%, list=11%, signal=47% |
| 10 | KEGG\_METABOLISM\_OF\_XENOBIOTICS\_BY\_CYTOCHROME\_P450 | Details ... | 56 | 0.75 | 1.49 | 0.045 | 0.336 | 0.818 | 2510 | tags=46%, list=12%, signal=52% |
| 11 | KEGG\_RETINOL\_METABOLISM | Details ... | 49 | 0.67 | 1.49 | 0.039 | 0.308 | 0.823 | 2919 | tags=39%, list=13%, signal=45% |
| 12 | KEGG\_GLYCOLYSIS\_GLUCONEOGENESIS | Details ... | 60 | 0.51 | 1.46 | 0.034 | 0.341 | 0.874 | 2370 | tags=27%, list=11%, signal=30% |
| 13 | KEGG\_VASOPRESSIN\_REGULATED\_WATER\_REABSORPTION | Details ... | 44 | 0.46 | 1.44 | 0.043 | 0.370 | 0.898 | 4873 | tags=32%, list=22%, signal=41% |
| 14 | KEGG\_ABC\_TRANSPORTERS | Details ... | 42 | 0.56 | 1.43 | 0.062 | 0.382 | 0.919 | 3729 | tags=33%, list=17%, signal=40% |
| 15 | KEGG\_PPAR\_SIGNALING\_PATHWAY | Details ... | 67 | 0.51 | 1.42 | 0.023 | 0.369 | 0.928 | 3119 | tags=39%, list=14%, signal=45% |
| 16 | KEGG\_PHENYLALANINE\_METABOLISM | Details ... | 17 | 0.66 | 1.41 | 0.083 | 0.390 | 0.940 | 3051 | tags=41%, list=14%, signal=48% |
| 17 | KEGG\_LINOLEIC\_ACID\_METABOLISM | Details ... | 24 | 0.73 | 1.40 | 0.073 | 0.375 | 0.941 | 2316 | tags=46%, list=11%, signal=51% |
| 18 | KEGG\_RIBOSOME | Details ... | 71 | 0.35 | 1.40 | 0.184 | 0.366 | 0.950 | 8328 | tags=69%, list=38%, signal=111% |
| 19 | KEGG\_CITRATE\_CYCLE\_TCA\_CYCLE | Details ... | 30 | 0.45 | 1.40 | 0.107 | 0.349 | 0.950 | 6363 | tags=63%, list=29%, signal=89% |
| 20 | KEGG\_TIGHT\_JUNCTION | Details ... | 128 | 0.46 | 1.39 | 0.037 | 0.343 | 0.952 | 3104 | tags=30%, list=14%, signal=34% |
| 21 | KEGG\_REGULATION\_OF\_AUTOPHAGY |  | 34 | 0.42 | 1.38 | 0.104 | 0.347 | 0.956 | 4852 | tags=26%, list=22%, signal=34% |
| 22 | KEGG\_PEROXISOME |  | 77 | 0.43 | 1.38 | 0.092 | 0.339 | 0.957 | 4923 | tags=40%, list=23%, signal=52% |
| 23 | KEGG\_NITROGEN\_METABOLISM |  | 22 | 0.54 | 1.36 | 0.053 | 0.370 | 0.965 | 2279 | tags=23%, list=10%, signal=25% |
| 24 | KEGG\_TASTE\_TRANSDUCTION |  | 43 | 0.46 | 1.35 | 0.142 | 0.370 | 0.966 | 2921 | tags=16%, list=13%, signal=19% |
| 25 | KEGG\_RIBOFLAVIN\_METABOLISM |  | 16 | 0.52 | 1.35 | 0.096 | 0.367 | 0.969 | 1449 | tags=19%, list=7%, signal=20% |
| 26 | KEGG\_HISTIDINE\_METABOLISM |  | 28 | 0.57 | 1.35 | 0.106 | 0.354 | 0.969 | 3166 | tags=43%, list=15%, signal=50% |
| 27 | KEGG\_BUTANOATE\_METABOLISM |  | 33 | 0.51 | 1.33 | 0.118 | 0.384 | 0.983 | 3239 | tags=36%, list=15%, signal=43% |
| 28 | KEGG\_PYRUVATE\_METABOLISM |  | 38 | 0.42 | 1.30 | 0.128 | 0.426 | 0.989 | 6014 | tags=55%, list=28%, signal=76% |
| 29 | KEGG\_GLUTATHIONE\_METABOLISM |  | 47 | 0.56 | 1.27 | 0.160 | 0.474 | 0.995 | 2510 | tags=32%, list=12%, signal=36% |
| 30 | KEGG\_ALDOSTERONE\_REGULATED\_SODIUM\_REABSORPTION |  | 41 | 0.51 | 1.27 | 0.120 | 0.463 | 0.995 | 3451 | tags=32%, list=16%, signal=38% |
| 31 | KEGG\_ALPHA\_LINOLENIC\_ACID\_METABOLISM |  | 15 | 0.64 | 1.27 | 0.182 | 0.452 | 0.995 | 3854 | tags=60%, list=18%, signal=73% |
| 32 | KEGG\_PENTOSE\_AND\_GLUCURONATE\_INTERCONVERSIONS |  | 17 | 0.60 | 1.26 | 0.198 | 0.461 | 0.996 | 1577 | tags=29%, list=7%, signal=32% |
| 33 | KEGG\_INSULIN\_SIGNALING\_PATHWAY |  | 135 | 0.35 | 1.24 | 0.109 | 0.496 | 0.998 | 3622 | tags=27%, list=17%, signal=33% |
| 34 | KEGG\_PORPHYRIN\_AND\_CHLOROPHYLL\_METABOLISM |  | 30 | 0.48 | 1.23 | 0.200 | 0.497 | 0.998 | 2460 | tags=23%, list=11%, signal=26% |
| 35 | KEGG\_GLYCEROPHOSPHOLIPID\_METABOLISM |  | 66 | 0.40 | 1.23 | 0.110 | 0.488 | 0.998 | 3990 | tags=30%, list=18%, signal=37% |
| 36 | KEGG\_STEROID\_HORMONE\_BIOSYNTHESIS |  | 42 | 0.53 | 1.23 | 0.204 | 0.478 | 0.998 | 3558 | tags=36%, list=16%, signal=43% |
| 37 | KEGG\_PROXIMAL\_TUBULE\_BICARBONATE\_RECLAMATION |  | 22 | 0.48 | 1.22 | 0.231 | 0.479 | 0.998 | 2229 | tags=23%, list=10%, signal=25% |
| 38 | KEGG\_GNRH\_SIGNALING\_PATHWAY |  | 94 | 0.38 | 1.21 | 0.168 | 0.484 | 0.999 | 4677 | tags=34%, list=22%, signal=43% |
| 39 | KEGG\_GLYCINE\_SERINE\_AND\_THREONINE\_METABOLISM |  | 31 | 0.53 | 1.21 | 0.230 | 0.487 | 0.999 | 2325 | tags=29%, list=11%, signal=32% |
| 40 | KEGG\_VIBRIO\_CHOLERAE\_INFECTION |  | 52 | 0.36 | 1.19 | 0.221 | 0.519 | 1.000 | 304 | tags=8%, list=1%, signal=8% |
| 41 | KEGG\_HUNTINGTONS\_DISEASE |  | 168 | 0.28 | 1.18 | 0.244 | 0.526 | 1.000 | 5924 | tags=30%, list=27%, signal=41% |
| 42 | KEGG\_LONG\_TERM\_POTENTIATION |  | 68 | 0.33 | 1.17 | 0.168 | 0.529 | 1.000 | 4844 | tags=31%, list=22%, signal=40% |
| 43 | KEGG\_ASCORBATE\_AND\_ALDARATE\_METABOLISM |  | 15 | 0.56 | 1.14 | 0.329 | 0.587 | 1.000 | 1662 | tags=33%, list=8%, signal=36% |
| 44 | KEGG\_PRIMARY\_BILE\_ACID\_BIOSYNTHESIS |  | 16 | 0.48 | 1.13 | 0.269 | 0.588 | 1.000 | 4241 | tags=44%, list=19%, signal=54% |
| 45 | KEGG\_TRYPTOPHAN\_METABOLISM |  | 39 | 0.42 | 1.11 | 0.267 | 0.629 | 1.000 | 2512 | tags=26%, list=12%, signal=29% |
| 46 | KEGG\_CALCIUM\_SIGNALING\_PATHWAY |  | 172 | 0.33 | 1.10 | 0.261 | 0.631 | 1.000 | 5156 | tags=32%, list=24%, signal=42% |
| 47 | KEGG\_FC\_EPSILON\_RI\_SIGNALING\_PATHWAY |  | 74 | 0.39 | 1.07 | 0.347 | 0.688 | 1.000 | 3586 | tags=32%, list=16%, signal=39% |
| 48 | KEGG\_STARCH\_AND\_SUCROSE\_METABOLISM |  | 37 | 0.40 | 1.07 | 0.343 | 0.674 | 1.000 | 1577 | tags=16%, list=7%, signal=17% |
| 49 | KEGG\_GLYCOSYLPHOSPHATIDYLINOSITOL\_GPI\_ANCHOR\_BIOSYNTHESIS |  | 24 | 0.40 | 1.07 | 0.347 | 0.662 | 1.000 | 3936 | tags=25%, list=18%, signal=30% |
| 50 | KEGG\_OLFACTORY\_TRANSDUCTION |  | 111 | 0.24 | 1.07 | 0.351 | 0.650 | 1.000 | 793 | tags=3%, list=4%, signal=3% |
| 51 | KEGG\_BIOSYNTHESIS\_OF\_UNSATURATED\_FATTY\_ACIDS |  | 18 | 0.44 | 1.07 | 0.391 | 0.645 | 1.000 | 3119 | tags=39%, list=14%, signal=45% |
| 52 | KEGG\_O\_GLYCAN\_BIOSYNTHESIS |  | 26 | 0.49 | 1.07 | 0.394 | 0.632 | 1.000 | 3877 | tags=38%, list=18%, signal=47% |
| 53 | KEGG\_PENTOSE\_PHOSPHATE\_PATHWAY |  | 26 | 0.40 | 1.07 | 0.366 | 0.621 | 1.000 | 3202 | tags=23%, list=15%, signal=27% |
| 54 | KEGG\_ETHER\_LIPID\_METABOLISM |  | 26 | 0.45 | 1.05 | 0.367 | 0.648 | 1.000 | 3990 | tags=38%, list=18%, signal=47% |
| 55 | KEGG\_VASCULAR\_SMOOTH\_MUSCLE\_CONTRACTION |  | 109 | 0.36 | 1.05 | 0.379 | 0.642 | 1.000 | 2581 | tags=20%, list=12%, signal=23% |
| 56 | KEGG\_LONG\_TERM\_DEPRESSION |  | 65 | 0.34 | 1.04 | 0.405 | 0.652 | 1.000 | 3446 | tags=25%, list=16%, signal=29% |
| 57 | KEGG\_GLYCOSPHINGOLIPID\_BIOSYNTHESIS\_LACTO\_AND\_NEOLACTO\_SERIES |  | 25 | 0.48 | 1.04 | 0.451 | 0.641 | 1.000 | 1100 | tags=20%, list=5%, signal=21% |
| 58 | KEGG\_ALANINE\_ASPARTATE\_AND\_GLUTAMATE\_METABOLISM |  | 32 | 0.40 | 1.04 | 0.400 | 0.631 | 1.000 | 3166 | tags=25%, list=15%, signal=29% |
| 59 | KEGG\_ONE\_CARBON\_POOL\_BY\_FOLATE |  | 16 | 0.40 | 1.03 | 0.401 | 0.634 | 1.000 | 637 | tags=13%, list=3%, signal=13% |
| 60 | KEGG\_RENIN\_ANGIOTENSIN\_SYSTEM |  | 17 | 0.51 | 1.03 | 0.435 | 0.626 | 1.000 | 2232 | tags=35%, list=10%, signal=39% |
| 61 | KEGG\_CARDIAC\_MUSCLE\_CONTRACTION |  | 73 | 0.44 | 1.03 | 0.476 | 0.623 | 1.000 | 5206 | tags=37%, list=24%, signal=48% |
| 62 | KEGG\_TERPENOID\_BACKBONE\_BIOSYNTHESIS |  | 15 | 0.47 | 1.02 | 0.468 | 0.633 | 1.000 | 6260 | tags=67%, list=29%, signal=94% |
| 63 | KEGG\_PANTOTHENATE\_AND\_COA\_BIOSYNTHESIS |  | 16 | 0.44 | 1.01 | 0.430 | 0.643 | 1.000 | 2980 | tags=25%, list=14%, signal=29% |
| 64 | KEGG\_ALZHEIMERS\_DISEASE |  | 154 | 0.26 | 1.01 | 0.435 | 0.634 | 1.000 | 5924 | tags=34%, list=27%, signal=47% |
| 65 | KEGG\_NEUROACTIVE\_LIGAND\_RECEPTOR\_INTERACTION |  | 262 | 0.28 | 0.99 | 0.481 | 0.669 | 1.000 | 2773 | tags=13%, list=13%, signal=14% |
| 66 | KEGG\_BETA\_ALANINE\_METABOLISM |  | 22 | 0.36 | 0.98 | 0.485 | 0.674 | 1.000 | 2745 | tags=27%, list=13%, signal=31% |
| 67 | KEGG\_MTOR\_SIGNALING\_PATHWAY |  | 50 | 0.31 | 0.97 | 0.488 | 0.675 | 1.000 | 4208 | tags=28%, list=19%, signal=35% |
| 68 | KEGG\_LEUKOCYTE\_TRANSENDOTHELIAL\_MIGRATION |  | 113 | 0.36 | 0.97 | 0.511 | 0.675 | 1.000 | 2563 | tags=23%, list=12%, signal=26% |
| 69 | KEGG\_GLYCEROLIPID\_METABOLISM |  | 42 | 0.33 | 0.95 | 0.567 | 0.708 | 1.000 | 2384 | tags=21%, list=11%, signal=24% |
| 70 | KEGG\_SELENOAMINO\_ACID\_METABOLISM |  | 25 | 0.36 | 0.94 | 0.565 | 0.725 | 1.000 | 7 | tags=4%, list=0%, signal=4% |
| 71 | KEGG\_ARGININE\_AND\_PROLINE\_METABOLISM |  | 49 | 0.32 | 0.92 | 0.605 | 0.741 | 1.000 | 3051 | tags=31%, list=14%, signal=36% |
| 72 | KEGG\_PRIMARY\_IMMUNODEFICIENCY |  | 35 | 0.49 | 0.92 | 0.605 | 0.742 | 1.000 | 2500 | tags=34%, list=11%, signal=39% |
| 73 | KEGG\_NICOTINATE\_AND\_NICOTINAMIDE\_METABOLISM |  | 21 | 0.38 | 0.92 | 0.589 | 0.734 | 1.000 | 1633 | tags=19%, list=8%, signal=21% |
| 74 | KEGG\_GLYCOSAMINOGLYCAN\_DEGRADATION |  | 20 | 0.37 | 0.92 | 0.582 | 0.726 | 1.000 | 4964 | tags=35%, list=23%, signal=45% |
| 75 | KEGG\_LYSINE\_DEGRADATION |  | 41 | 0.29 | 0.91 | 0.621 | 0.735 | 1.000 | 5526 | tags=49%, list=25%, signal=65% |
| 76 | KEGG\_CELL\_ADHESION\_MOLECULES\_CAMS |  | 126 | 0.36 | 0.90 | 0.584 | 0.734 | 1.000 | 2920 | tags=26%, list=13%, signal=30% |
| 77 | KEGG\_ADIPOCYTOKINE\_SIGNALING\_PATHWAY |  | 66 | 0.28 | 0.90 | 0.650 | 0.729 | 1.000 | 3859 | tags=24%, list=18%, signal=29% |
| 78 | KEGG\_DRUG\_METABOLISM\_OTHER\_ENZYMES |  | 38 | 0.32 | 0.90 | 0.608 | 0.728 | 1.000 | 1577 | tags=13%, list=7%, signal=14% |
| 79 | KEGG\_EPITHELIAL\_CELL\_SIGNALING\_IN\_HELICOBACTER\_PYLORI\_INFECTION |  | 66 | 0.28 | 0.88 | 0.659 | 0.745 | 1.000 | 1279 | tags=11%, list=6%, signal=11% |
| 80 | KEGG\_AMINO\_SUGAR\_AND\_NUCLEOTIDE\_SUGAR\_METABOLISM |  | 42 | 0.28 | 0.85 | 0.689 | 0.787 | 1.000 | 3370 | tags=19%, list=15%, signal=22% |
| 81 | KEGG\_STEROID\_BIOSYNTHESIS |  | 15 | 0.38 | 0.84 | 0.699 | 0.803 | 1.000 | 2995 | tags=40%, list=14%, signal=46% |
| 82 | KEGG\_PHOSPHATIDYLINOSITOL\_SIGNALING\_SYSTEM |  | 75 | 0.25 | 0.81 | 0.840 | 0.849 | 1.000 | 5173 | tags=35%, list=24%, signal=45% |
| 83 | KEGG\_INTESTINAL\_IMMUNE\_NETWORK\_FOR\_IGA\_PRODUCTION |  | 45 | 0.40 | 0.81 | 0.703 | 0.842 | 1.000 | 2893 | tags=27%, list=13%, signal=31% |
| 84 | KEGG\_LYSOSOME |  | 114 | 0.25 | 0.80 | 0.726 | 0.840 | 1.000 | 5819 | tags=32%, list=27%, signal=44% |
| 85 | KEGG\_FRUCTOSE\_AND\_MANNOSE\_METABOLISM |  | 33 | 0.28 | 0.80 | 0.781 | 0.840 | 1.000 | 3666 | tags=27%, list=17%, signal=33% |
| 86 | KEGG\_COMPLEMENT\_AND\_COAGULATION\_CASCADES |  | 67 | 0.31 | 0.79 | 0.804 | 0.846 | 1.000 | 2840 | tags=21%, list=13%, signal=24% |
| 87 | KEGG\_T\_CELL\_RECEPTOR\_SIGNALING\_PATHWAY |  | 106 | 0.30 | 0.77 | 0.735 | 0.859 | 1.000 | 3882 | tags=30%, list=18%, signal=37% |
| 88 | KEGG\_FC\_GAMMA\_R\_MEDIATED\_PHAGOCYTOSIS |  | 91 | 0.25 | 0.75 | 0.886 | 0.884 | 1.000 | 3451 | tags=22%, list=16%, signal=26% |
| 89 | KEGG\_VIRAL\_MYOCARDITIS |  | 67 | 0.31 | 0.72 | 0.851 | 0.910 | 1.000 | 3251 | tags=25%, list=15%, signal=30% |
| 90 | KEGG\_B\_CELL\_RECEPTOR\_SIGNALING\_PATHWAY |  | 74 | 0.27 | 0.70 | 0.848 | 0.920 | 1.000 | 3882 | tags=30%, list=18%, signal=36% |
| 91 | KEGG\_DILATED\_CARDIOMYOPATHY |  | 89 | 0.31 | 0.68 | 0.844 | 0.935 | 1.000 | 1830 | tags=18%, list=8%, signal=20% |
| 92 | KEGG\_ASTHMA |  | 27 | 0.30 | 0.62 | 0.920 | 0.968 | 1.000 | 2893 | tags=22%, list=13%, signal=26% |
| 93 | KEGG\_ANTIGEN\_PROCESSING\_AND\_PRESENTATION |  | 80 | 0.21 | 0.53 | 0.943 | 0.988 | 1.000 | 2893 | tags=14%, list=13%, signal=16% |
Table: Gene sets enriched in phenotype **H (43 samples)**[plain text format]****

  
