# Supplementary material for: Identification and validation of key modules and hub genes associated with the pathological stage of oral squamous cell carcinoma by weighted gene co-expression network analysis
Source: PeerJ. 2020 Feb 4;8:e8505. doi: 10.7717/peerj.8505 (PMC7006519; doi:10.7717/peerj.8505)
Supplement: File S6 [file peerj-08-8505-s006.zip › my_analysis_205382_KEGG.Gsea.1570105897088/gsea_report_for_L_1570105897088.html]

Report for L 1570105897088 [GSEA]

| GS  follow link to MSigDB | GS DETAILS | SIZE | ES | NES | NOM p-val | FDR q-val | FWER p-val | RANK AT MAX | LEADING EDGE || 1 | KEGG\_SMALL\_CELL\_LUNG\_CANCER | Details ... | 84 | -0.62 | -1.73 | 0.000 | 0.262 | 0.204 | 3116 | tags=42%, list=14%, signal=48% |
| 2 | KEGG\_RENAL\_CELL\_CARCINOMA | Details ... | 68 | -0.56 | -1.73 | 0.000 | 0.131 | 0.204 | 2625 | tags=35%, list=12%, signal=40% |
| 3 | KEGG\_GLYCOSAMINOGLYCAN\_BIOSYNTHESIS\_HEPARAN\_SULFATE | Details ... | 26 | -0.66 | -1.59 | 0.008 | 0.411 | 0.557 | 3273 | tags=58%, list=15%, signal=68% |
| 4 | KEGG\_GLYCOSAMINOGLYCAN\_BIOSYNTHESIS\_CHONDROITIN\_SULFATE | Details ... | 22 | -0.76 | -1.57 | 0.006 | 0.379 | 0.617 | 2633 | tags=64%, list=12%, signal=72% |
| 5 | KEGG\_CHRONIC\_MYELOID\_LEUKEMIA | Details ... | 72 | -0.46 | -1.54 | 0.010 | 0.365 | 0.674 | 3488 | tags=39%, list=16%, signal=46% |
| 6 | KEGG\_SNARE\_INTERACTIONS\_IN\_VESICULAR\_TRANSPORT | Details ... | 38 | -0.48 | -1.54 | 0.037 | 0.310 | 0.683 | 4300 | tags=55%, list=20%, signal=69% |
| 7 | KEGG\_P53\_SIGNALING\_PATHWAY | Details ... | 65 | -0.53 | -1.53 | 0.004 | 0.306 | 0.729 | 2468 | tags=34%, list=11%, signal=38% |
| 8 | KEGG\_PANCREATIC\_CANCER | Details ... | 69 | -0.49 | -1.52 | 0.008 | 0.293 | 0.744 | 3755 | tags=41%, list=17%, signal=49% |
| 9 | KEGG\_PROTEASOME | Details ... | 41 | -0.59 | -1.51 | 0.097 | 0.265 | 0.750 | 4908 | tags=56%, list=23%, signal=72% |
| 10 | KEGG\_UBIQUITIN\_MEDIATED\_PROTEOLYSIS | Details ... | 129 | -0.39 | -1.51 | 0.044 | 0.257 | 0.767 | 4427 | tags=30%, list=20%, signal=38% |
| 11 | KEGG\_BLADDER\_CANCER | Details ... | 40 | -0.56 | -1.48 | 0.028 | 0.279 | 0.815 | 3116 | tags=43%, list=14%, signal=50% |
| 12 | KEGG\_MISMATCH\_REPAIR | Details ... | 22 | -0.66 | -1.48 | 0.082 | 0.257 | 0.817 | 5138 | tags=59%, list=24%, signal=77% |
| 13 | KEGG\_BASAL\_TRANSCRIPTION\_FACTORS | Details ... | 32 | -0.49 | -1.47 | 0.066 | 0.267 | 0.845 | 4962 | tags=44%, list=23%, signal=57% |
| 14 | KEGG\_PATHWAYS\_IN\_CANCER | Details ... | 319 | -0.46 | -1.46 | 0.021 | 0.264 | 0.857 | 3009 | tags=31%, list=14%, signal=35% |
| 15 | KEGG\_CELL\_CYCLE | Details ... | 122 | -0.55 | -1.46 | 0.087 | 0.253 | 0.861 | 4689 | tags=52%, list=22%, signal=65% |
| 16 | KEGG\_DNA\_REPLICATION | Details ... | 36 | -0.68 | -1.42 | 0.099 | 0.306 | 0.921 | 5138 | tags=58%, list=24%, signal=76% |
| 17 | KEGG\_SPLICEOSOME | Details ... | 114 | -0.41 | -1.40 | 0.163 | 0.347 | 0.944 | 7019 | tags=53%, list=32%, signal=77% |
| 18 | KEGG\_ERBB\_SIGNALING\_PATHWAY | Details ... | 86 | -0.39 | -1.39 | 0.028 | 0.346 | 0.948 | 3488 | tags=34%, list=16%, signal=40% |
| 19 | KEGG\_PYRIMIDINE\_METABOLISM | Details ... | 91 | -0.43 | -1.38 | 0.088 | 0.335 | 0.951 | 5165 | tags=40%, list=24%, signal=52% |
| 20 | KEGG\_COLORECTAL\_CANCER | Details ... | 62 | -0.44 | -1.36 | 0.096 | 0.360 | 0.962 | 3503 | tags=32%, list=16%, signal=38% |
| 21 | KEGG\_NUCLEOTIDE\_EXCISION\_REPAIR |  | 43 | -0.50 | -1.36 | 0.161 | 0.354 | 0.966 | 5138 | tags=44%, list=24%, signal=58% |
| 22 | KEGG\_THYROID\_CANCER |  | 29 | -0.46 | -1.29 | 0.122 | 0.506 | 0.992 | 4507 | tags=41%, list=21%, signal=52% |
| 23 | KEGG\_PATHOGENIC\_ESCHERICHIA\_COLI\_INFECTION |  | 53 | -0.37 | -1.29 | 0.132 | 0.491 | 0.994 | 5929 | tags=58%, list=27%, signal=80% |
| 24 | KEGG\_GLIOMA |  | 64 | -0.39 | -1.29 | 0.087 | 0.471 | 0.994 | 2623 | tags=30%, list=12%, signal=34% |
| 25 | KEGG\_HOMOLOGOUS\_RECOMBINATION |  | 28 | -0.55 | -1.27 | 0.219 | 0.492 | 0.996 | 5293 | tags=54%, list=24%, signal=71% |
| 26 | KEGG\_NON\_SMALL\_CELL\_LUNG\_CANCER |  | 54 | -0.37 | -1.27 | 0.083 | 0.479 | 0.996 | 3219 | tags=26%, list=15%, signal=30% |
| 27 | KEGG\_FOCAL\_ADHESION |  | 195 | -0.48 | -1.26 | 0.175 | 0.474 | 0.996 | 2623 | tags=36%, list=12%, signal=40% |
| 28 | KEGG\_DORSO\_VENTRAL\_AXIS\_FORMATION |  | 23 | -0.47 | -1.26 | 0.149 | 0.470 | 0.996 | 2035 | tags=30%, list=9%, signal=34% |
| 29 | KEGG\_REGULATION\_OF\_ACTIN\_CYTOSKELETON |  | 209 | -0.37 | -1.25 | 0.095 | 0.471 | 0.997 | 3642 | tags=32%, list=17%, signal=38% |
| 30 | KEGG\_ECM\_RECEPTOR\_INTERACTION |  | 81 | -0.56 | -1.25 | 0.225 | 0.459 | 0.997 | 1948 | tags=44%, list=9%, signal=49% |
| 31 | KEGG\_ADHERENS\_JUNCTION |  | 67 | -0.38 | -1.24 | 0.136 | 0.461 | 0.998 | 3809 | tags=33%, list=18%, signal=40% |
| 32 | KEGG\_PROTEIN\_EXPORT |  | 22 | -0.40 | -1.22 | 0.258 | 0.491 | 0.998 | 3865 | tags=27%, list=18%, signal=33% |
| 33 | KEGG\_JAK\_STAT\_SIGNALING\_PATHWAY |  | 151 | -0.42 | -1.21 | 0.187 | 0.503 | 0.999 | 3488 | tags=27%, list=16%, signal=32% |
| 34 | KEGG\_NOD\_LIKE\_RECEPTOR\_SIGNALING\_PATHWAY |  | 59 | -0.49 | -1.20 | 0.243 | 0.496 | 0.999 | 2925 | tags=32%, list=13%, signal=37% |
| 35 | KEGG\_PRION\_DISEASES |  | 34 | -0.44 | -1.19 | 0.227 | 0.517 | 0.999 | 1254 | tags=21%, list=6%, signal=22% |
| 36 | KEGG\_MAPK\_SIGNALING\_PATHWAY |  | 256 | -0.32 | -1.18 | 0.136 | 0.515 | 0.999 | 3009 | tags=26%, list=14%, signal=30% |
| 37 | KEGG\_GAP\_JUNCTION |  | 87 | -0.36 | -1.18 | 0.198 | 0.509 | 0.999 | 4342 | tags=43%, list=20%, signal=53% |
| 38 | KEGG\_RIG\_I\_LIKE\_RECEPTOR\_SIGNALING\_PATHWAY |  | 69 | -0.39 | -1.16 | 0.253 | 0.537 | 0.999 | 2925 | tags=26%, list=13%, signal=30% |
| 39 | KEGG\_PURINE\_METABOLISM |  | 148 | -0.33 | -1.16 | 0.220 | 0.528 | 0.999 | 3750 | tags=29%, list=17%, signal=35% |
| 40 | KEGG\_ENDOMETRIAL\_CANCER |  | 52 | -0.34 | -1.14 | 0.232 | 0.547 | 0.999 | 2344 | tags=23%, list=11%, signal=26% |
| 41 | KEGG\_ARRHYTHMOGENIC\_RIGHT\_VENTRICULAR\_CARDIOMYOPATHY\_ARVC |  | 73 | -0.47 | -1.14 | 0.338 | 0.543 | 0.999 | 1080 | tags=21%, list=5%, signal=22% |
| 42 | KEGG\_GALACTOSE\_METABOLISM |  | 25 | -0.40 | -1.14 | 0.289 | 0.534 | 0.999 | 1416 | tags=12%, list=7%, signal=13% |
| 43 | KEGG\_BASE\_EXCISION\_REPAIR |  | 32 | -0.44 | -1.13 | 0.327 | 0.530 | 0.999 | 4196 | tags=34%, list=19%, signal=43% |
| 44 | KEGG\_SYSTEMIC\_LUPUS\_ERYTHEMATOSUS |  | 102 | -0.44 | -1.11 | 0.335 | 0.560 | 0.999 | 2788 | tags=26%, list=13%, signal=30% |
| 45 | KEGG\_OOCYTE\_MEIOSIS |  | 107 | -0.31 | -1.11 | 0.290 | 0.549 | 0.999 | 2848 | tags=25%, list=13%, signal=29% |
| 46 | KEGG\_PROSTATE\_CANCER |  | 89 | -0.34 | -1.11 | 0.257 | 0.539 | 0.999 | 3240 | tags=30%, list=15%, signal=36% |
| 47 | KEGG\_NEUROTROPHIN\_SIGNALING\_PATHWAY |  | 123 | -0.30 | -1.11 | 0.237 | 0.528 | 0.999 | 2935 | tags=25%, list=13%, signal=29% |
| 48 | KEGG\_BASAL\_CELL\_CARCINOMA |  | 52 | -0.48 | -1.11 | 0.317 | 0.523 | 0.999 | 2050 | tags=27%, list=9%, signal=30% |
| 49 | KEGG\_CYTOKINE\_CYTOKINE\_RECEPTOR\_INTERACTION |  | 250 | -0.44 | -1.11 | 0.308 | 0.514 | 0.999 | 2203 | tags=26%, list=10%, signal=29% |
| 50 | KEGG\_MELANOMA |  | 71 | -0.37 | -1.09 | 0.297 | 0.544 | 0.999 | 2623 | tags=30%, list=12%, signal=34% |
| 51 | KEGG\_RNA\_DEGRADATION |  | 56 | -0.35 | -1.09 | 0.370 | 0.534 | 0.999 | 5635 | tags=39%, list=26%, signal=53% |
| 52 | KEGG\_TGF\_BETA\_SIGNALING\_PATHWAY |  | 82 | -0.36 | -1.07 | 0.341 | 0.550 | 1.000 | 3673 | tags=34%, list=17%, signal=41% |
| 53 | KEGG\_CYSTEINE\_AND\_METHIONINE\_METABOLISM |  | 34 | -0.36 | -1.07 | 0.352 | 0.545 | 1.000 | 4461 | tags=38%, list=21%, signal=48% |
| 54 | KEGG\_TYPE\_II\_DIABETES\_MELLITUS |  | 44 | -0.35 | -1.04 | 0.389 | 0.598 | 1.000 | 2023 | tags=18%, list=9%, signal=20% |
| 55 | KEGG\_CYTOSOLIC\_DNA\_SENSING\_PATHWAY |  | 53 | -0.40 | -1.03 | 0.441 | 0.613 | 1.000 | 5780 | tags=40%, list=27%, signal=54% |
| 56 | KEGG\_HEDGEHOG\_SIGNALING\_PATHWAY |  | 53 | -0.40 | -1.03 | 0.395 | 0.603 | 1.000 | 4726 | tags=45%, list=22%, signal=58% |
| 57 | KEGG\_LEISHMANIA\_INFECTION |  | 68 | -0.44 | -1.02 | 0.452 | 0.601 | 1.000 | 2925 | tags=35%, list=13%, signal=41% |
| 58 | KEGG\_GLYOXYLATE\_AND\_DICARBOXYLATE\_METABOLISM |  | 16 | -0.37 | -1.02 | 0.451 | 0.595 | 1.000 | 3759 | tags=31%, list=17%, signal=38% |
| 59 | KEGG\_AMINOACYL\_TRNA\_BIOSYNTHESIS |  | 37 | -0.37 | -1.02 | 0.452 | 0.590 | 1.000 | 7218 | tags=59%, list=33%, signal=89% |
| 60 | KEGG\_PROGESTERONE\_MEDIATED\_OOCYTE\_MATURATION |  | 83 | -0.31 | -1.00 | 0.420 | 0.606 | 1.000 | 2211 | tags=23%, list=10%, signal=25% |
| 61 | KEGG\_AXON\_GUIDANCE |  | 127 | -0.30 | -0.99 | 0.444 | 0.616 | 1.000 | 3471 | tags=28%, list=16%, signal=34% |
| 62 | KEGG\_AMYOTROPHIC\_LATERAL\_SCLEROSIS\_ALS |  | 51 | -0.30 | -0.97 | 0.507 | 0.646 | 1.000 | 1420 | tags=10%, list=7%, signal=10% |
| 63 | KEGG\_WNT\_SIGNALING\_PATHWAY |  | 145 | -0.30 | -0.97 | 0.507 | 0.648 | 1.000 | 2945 | tags=24%, list=14%, signal=28% |
| 64 | KEGG\_APOPTOSIS |  | 86 | -0.32 | -0.97 | 0.494 | 0.638 | 1.000 | 1420 | tags=14%, list=7%, signal=15% |
| 65 | KEGG\_VEGF\_SIGNALING\_PATHWAY |  | 71 | -0.31 | -0.97 | 0.510 | 0.630 | 1.000 | 2211 | tags=21%, list=10%, signal=23% |
| 66 | KEGG\_INOSITOL\_PHOSPHATE\_METABOLISM |  | 54 | -0.29 | -0.94 | 0.587 | 0.667 | 1.000 | 2696 | tags=19%, list=12%, signal=21% |
| 67 | KEGG\_SPHINGOLIPID\_METABOLISM |  | 32 | -0.33 | -0.93 | 0.578 | 0.686 | 1.000 | 2072 | tags=22%, list=10%, signal=24% |
| 68 | KEGG\_GRAFT\_VERSUS\_HOST\_DISEASE |  | 37 | -0.44 | -0.89 | 0.633 | 0.749 | 1.000 | 1324 | tags=16%, list=6%, signal=17% |
| 69 | KEGG\_HEMATOPOIETIC\_CELL\_LINEAGE |  | 84 | -0.39 | -0.88 | 0.650 | 0.744 | 1.000 | 2363 | tags=25%, list=11%, signal=28% |
| 70 | KEGG\_N\_GLYCAN\_BIOSYNTHESIS |  | 46 | -0.29 | -0.88 | 0.592 | 0.746 | 1.000 | 6032 | tags=54%, list=28%, signal=75% |
| 71 | KEGG\_TOLL\_LIKE\_RECEPTOR\_SIGNALING\_PATHWAY |  | 98 | -0.33 | -0.88 | 0.621 | 0.736 | 1.000 | 3262 | tags=24%, list=15%, signal=29% |
| 72 | KEGG\_TYPE\_I\_DIABETES\_MELLITUS |  | 40 | -0.40 | -0.85 | 0.656 | 0.784 | 1.000 | 2083 | tags=20%, list=10%, signal=22% |
| 73 | KEGG\_ACUTE\_MYELOID\_LEUKEMIA |  | 56 | -0.29 | -0.85 | 0.709 | 0.773 | 1.000 | 3219 | tags=29%, list=15%, signal=33% |
| 74 | KEGG\_ENDOCYTOSIS |  | 171 | -0.23 | -0.84 | 0.796 | 0.771 | 1.000 | 4919 | tags=33%, list=23%, signal=42% |
| 75 | KEGG\_NOTCH\_SIGNALING\_PATHWAY |  | 46 | -0.24 | -0.82 | 0.709 | 0.791 | 1.000 | 3403 | tags=22%, list=16%, signal=26% |
| 76 | KEGG\_MELANOGENESIS |  | 97 | -0.27 | -0.82 | 0.800 | 0.787 | 1.000 | 2110 | tags=21%, list=10%, signal=23% |
| 77 | KEGG\_RNA\_POLYMERASE |  | 28 | -0.28 | -0.82 | 0.669 | 0.787 | 1.000 | 5395 | tags=36%, list=25%, signal=47% |
| 78 | KEGG\_CHEMOKINE\_SIGNALING\_PATHWAY |  | 180 | -0.31 | -0.81 | 0.712 | 0.780 | 1.000 | 2211 | tags=22%, list=10%, signal=25% |
| 79 | KEGG\_HYPERTROPHIC\_CARDIOMYOPATHY\_HCM |  | 82 | -0.36 | -0.76 | 0.770 | 0.861 | 1.000 | 1880 | tags=22%, list=9%, signal=24% |
| 80 | KEGG\_NATURAL\_KILLER\_CELL\_MEDIATED\_CYTOTOXICITY |  | 131 | -0.24 | -0.63 | 0.922 | 0.978 | 1.000 | 2184 | tags=14%, list=10%, signal=15% |
| 81 | KEGG\_ALLOGRAFT\_REJECTION |  | 34 | -0.29 | -0.56 | 0.944 | 0.994 | 1.000 | 3346 | tags=21%, list=15%, signal=24% |
| 82 | KEGG\_AUTOIMMUNE\_THYROID\_DISEASE |  | 49 | -0.16 | -0.33 | 0.996 | 1.000 | 1.000 | 3346 | tags=10%, list=15%, signal=12% |
Table: Gene sets enriched in phenotype **L (54 samples)**[plain text format]****

  
