# Supplementary material for: Identification and validation of key modules and hub genes associated with the pathological stage of oral squamous cell carcinoma by weighted gene co-expression network analysis
Source: PeerJ. 2020 Feb 4;8:e8505. doi: 10.7717/peerj.8505 (PMC7006519; doi:10.7717/peerj.8505)
Supplement: File S6 [file peerj-08-8505-s006.zip › my_analysis_209283_BP.Gsea.1570106107776/GO_BROWN_FAT_CELL_DIFFERENTIATION.html]

Details for gene set GO\_BROWN\_FAT\_CELL\_DIFFERENTIATION[GSEA]

|  || Dataset | input.209283.cls#H\_versus\_L.209283.cls#H\_versus\_L\_repos |
| Phenotype | 209283.cls#H\_versus\_L\_repos |
| Upregulated in class | H |
| GeneSet | GO\_BROWN\_FAT\_CELL\_DIFFERENTIATION |
| Enrichment Score (ES) | 0.7588992 |
| Normalized Enrichment Score (NES) | 1.8012915 |
| Nominal p-value | 0.0 |
| FDR q-value | 0.095229685 |
| FWER p-Value | 0.485 |
Table: GSEA Results Summary

  

Fig 1: Enrichment plot: GO\_BROWN\_FAT\_CELL\_DIFFERENTIATION      
 Profile of the Running ES Score & Positions of GeneSet Members on the Rank Ordered List

  

| PROBE | DESCRIPTION (from dataset) | GENE SYMBOL | GENE\_TITLE | RANK IN GENE LIST | RANK METRIC SCORE | RUNNING ES | CORE ENRICHMENT || 1 | MB | na |  |  | 47 | 0.483 | 0.2488 | Yes |
| 2 | EBF2 | na |  |  | 224 | 0.265 | 0.3784 | Yes |
| 3 | FABP4 | na |  |  | 466 | 0.197 | 0.4695 | Yes |
| 4 | ADIPOQ | na |  |  | 598 | 0.173 | 0.5532 | Yes |
| 5 | ALDH6A1 | na |  |  | 1284 | 0.110 | 0.5790 | Yes |
| 6 | PPARGC1A | na |  |  | 1437 | 0.101 | 0.6245 | Yes |
| 7 | NUDT7 | na |  |  | 1601 | 0.095 | 0.6661 | Yes |
| 8 | ADRB2 | na |  |  | 2001 | 0.079 | 0.6890 | Yes |
| 9 | CEBPA | na |  |  | 2014 | 0.079 | 0.7295 | Yes |
| 10 | ZNF516 | na |  |  | 2202 | 0.073 | 0.7589 | Yes |
| 11 | RARRES2 | na |  |  | 3578 | 0.043 | 0.7178 | No |
| 12 | PRDM16 | na |  |  | 5433 | 0.022 | 0.6438 | No |
| 13 | PTGS2 | na |  |  | 7570 | 0.008 | 0.5494 | No |
| 14 | MRAP | na |  |  | 9184 | 0.001 | 0.4758 | No |
| 15 | ADIG | na |  |  | 9311 | 0.001 | 0.4705 | No |
| 16 | PEX11A | na |  |  | 9319 | 0.001 | 0.4707 | No |
| 17 | ADRB1 | na |  |  | 9402 | 0.001 | 0.4673 | No |
| 18 | ADRB3 | na |  |  | 9500 | 0.000 | 0.4631 | No |
| 19 | BNIP3 | na |  |  | 10024 | -0.001 | 0.4394 | No |
| 20 | SLC2A4 | na |  |  | 10363 | -0.001 | 0.4245 | No |
| 21 | UCP1 | na |  |  | 10713 | -0.002 | 0.4095 | No |
| 22 | ARL4A | na |  |  | 11443 | -0.004 | 0.3778 | No |
| 23 | ITGA6 | na |  |  | 12522 | -0.007 | 0.3317 | No |
| 24 | METRNL | na |  |  | 13730 | -0.011 | 0.2819 | No |
| 25 | RGS2 | na |  |  | 14915 | -0.017 | 0.2362 | No |
| 26 | CEBPB | na |  |  | 15791 | -0.022 | 0.2076 | No |
| 27 | PLAC8 | na |  |  | 15833 | -0.023 | 0.2175 | No |
| 28 | LAMB3 | na |  |  | 18006 | -0.041 | 0.1388 | No |
| 29 | LRG1 | na |  |  | 19629 | -0.065 | 0.0977 | No |
Table: GSEA details [plain text format]

  

Fig 2: GO\_BROWN\_FAT\_CELL\_DIFFERENTIATION      
 Blue-Pink O' Gram in the Space of the Analyzed GeneSet

  

Fig 3: GO\_BROWN\_FAT\_CELL\_DIFFERENTIATION: Random ES distribution      
 Gene set null distribution of ES for **GO\_BROWN\_FAT\_CELL\_DIFFERENTIATION**

  
