# Supplementary material for: Identification and validation of key modules and hub genes associated with the pathological stage of oral squamous cell carcinoma by weighted gene co-expression network analysis
Source: PeerJ. 2020 Feb 4;8:e8505. doi: 10.7717/peerj.8505 (PMC7006519; doi:10.7717/peerj.8505)
Supplement: File S6 [file peerj-08-8505-s006.zip › my_analysis_209283_KEGG.Gsea.1570105930256/gsea_report_for_H_1570105930256.html]

Report for H 1570105930256 [GSEA]

| GS  follow link to MSigDB | GS DETAILS | SIZE | ES | NES | NOM p-val | FDR q-val | FWER p-val | RANK AT MAX | LEADING EDGE || 1 | KEGG\_TIGHT\_JUNCTION | Details ... | 128 | 0.64 | 1.92 | 0.000 | 0.036 | 0.024 | 2720 | tags=28%, list=13%, signal=32% |
| 2 | KEGG\_CARDIAC\_MUSCLE\_CONTRACTION | Details ... | 73 | 0.80 | 1.87 | 0.000 | 0.039 | 0.049 | 963 | tags=26%, list=4%, signal=27% |
| 3 | KEGG\_DILATED\_CARDIOMYOPATHY | Details ... | 89 | 0.79 | 1.73 | 0.000 | 0.109 | 0.180 | 1109 | tags=35%, list=5%, signal=37% |
| 4 | KEGG\_CALCIUM\_SIGNALING\_PATHWAY | Details ... | 172 | 0.52 | 1.70 | 0.000 | 0.126 | 0.251 | 2825 | tags=23%, list=13%, signal=27% |
| 5 | KEGG\_VASCULAR\_SMOOTH\_MUSCLE\_CONTRACTION | Details ... | 109 | 0.58 | 1.67 | 0.000 | 0.140 | 0.322 | 4060 | tags=40%, list=19%, signal=49% |
| 6 | KEGG\_HYPERTROPHIC\_CARDIOMYOPATHY\_HCM | Details ... | 82 | 0.77 | 1.66 | 0.006 | 0.118 | 0.323 | 1109 | tags=35%, list=5%, signal=37% |
| 7 | KEGG\_ARRHYTHMOGENIC\_RIGHT\_VENTRICULAR\_CARDIOMYOPATHY\_ARVC | Details ... | 73 | 0.68 | 1.65 | 0.004 | 0.109 | 0.346 | 2164 | tags=32%, list=10%, signal=35% |
| 8 | KEGG\_PROXIMAL\_TUBULE\_BICARBONATE\_RECLAMATION | Details ... | 22 | 0.66 | 1.65 | 0.008 | 0.101 | 0.356 | 1521 | tags=18%, list=7%, signal=20% |
| 9 | KEGG\_OLFACTORY\_TRANSDUCTION | Details ... | 111 | 0.37 | 1.64 | 0.031 | 0.094 | 0.369 | 4859 | tags=13%, list=22%, signal=16% |
| 10 | KEGG\_AXON\_GUIDANCE | Details ... | 127 | 0.50 | 1.62 | 0.002 | 0.115 | 0.455 | 4952 | tags=43%, list=23%, signal=56% |
| 11 | KEGG\_NOTCH\_SIGNALING\_PATHWAY | Details ... | 46 | 0.47 | 1.62 | 0.021 | 0.105 | 0.457 | 3727 | tags=30%, list=17%, signal=37% |
| 12 | KEGG\_INSULIN\_SIGNALING\_PATHWAY | Details ... | 135 | 0.45 | 1.59 | 0.000 | 0.133 | 0.550 | 4861 | tags=39%, list=22%, signal=50% |
| 13 | KEGG\_GLYCOLYSIS\_GLUCONEOGENESIS | Details ... | 60 | 0.55 | 1.55 | 0.012 | 0.165 | 0.626 | 1922 | tags=20%, list=9%, signal=22% |
| 14 | KEGG\_LONG\_TERM\_POTENTIATION | Details ... | 68 | 0.43 | 1.50 | 0.010 | 0.252 | 0.778 | 4634 | tags=38%, list=21%, signal=48% |
| 15 | KEGG\_DRUG\_METABOLISM\_CYTOCHROME\_P450 | Details ... | 59 | 0.73 | 1.49 | 0.043 | 0.258 | 0.807 | 2187 | tags=41%, list=10%, signal=45% |
| 16 | KEGG\_GLIOMA | Details ... | 64 | 0.45 | 1.49 | 0.002 | 0.242 | 0.807 | 4887 | tags=44%, list=22%, signal=56% |
| 17 | KEGG\_PROPANOATE\_METABOLISM | Details ... | 32 | 0.57 | 1.49 | 0.062 | 0.233 | 0.815 | 4743 | tags=47%, list=22%, signal=60% |
| 18 | KEGG\_ALDOSTERONE\_REGULATED\_SODIUM\_REABSORPTION | Details ... | 41 | 0.60 | 1.48 | 0.023 | 0.225 | 0.821 | 4060 | tags=44%, list=19%, signal=54% |
| 19 | KEGG\_GNRH\_SIGNALING\_PATHWAY | Details ... | 94 | 0.46 | 1.48 | 0.028 | 0.229 | 0.837 | 4910 | tags=36%, list=23%, signal=47% |
| 20 | KEGG\_ALZHEIMERS\_DISEASE | Details ... | 154 | 0.38 | 1.45 | 0.032 | 0.260 | 0.876 | 1887 | tags=10%, list=9%, signal=11% |
| 21 | KEGG\_LONG\_TERM\_DEPRESSION |  | 65 | 0.49 | 1.45 | 0.030 | 0.249 | 0.877 | 2468 | tags=22%, list=11%, signal=24% |
| 22 | KEGG\_MELANOGENESIS |  | 97 | 0.48 | 1.45 | 0.032 | 0.249 | 0.889 | 4602 | tags=38%, list=21%, signal=48% |
| 23 | KEGG\_RENIN\_ANGIOTENSIN\_SYSTEM |  | 17 | 0.69 | 1.42 | 0.061 | 0.289 | 0.926 | 3074 | tags=41%, list=14%, signal=48% |
| 24 | KEGG\_ADHERENS\_JUNCTION |  | 67 | 0.42 | 1.40 | 0.043 | 0.313 | 0.943 | 1547 | tags=12%, list=7%, signal=13% |
| 25 | KEGG\_METABOLISM\_OF\_XENOBIOTICS\_BY\_CYTOCHROME\_P450 |  | 56 | 0.70 | 1.40 | 0.101 | 0.308 | 0.944 | 2187 | tags=38%, list=10%, signal=42% |
| 26 | KEGG\_BETA\_ALANINE\_METABOLISM |  | 22 | 0.52 | 1.40 | 0.081 | 0.297 | 0.944 | 4743 | tags=36%, list=22%, signal=46% |
| 27 | KEGG\_LINOLEIC\_ACID\_METABOLISM |  | 24 | 0.71 | 1.38 | 0.085 | 0.309 | 0.953 | 1701 | tags=38%, list=8%, signal=41% |
| 28 | KEGG\_RETINOL\_METABOLISM |  | 49 | 0.61 | 1.38 | 0.130 | 0.314 | 0.963 | 2970 | tags=31%, list=14%, signal=35% |
| 29 | KEGG\_GAP\_JUNCTION |  | 87 | 0.42 | 1.35 | 0.069 | 0.347 | 0.976 | 4380 | tags=32%, list=20%, signal=40% |
| 30 | KEGG\_STARCH\_AND\_SUCROSE\_METABOLISM |  | 37 | 0.51 | 1.35 | 0.079 | 0.347 | 0.978 | 2190 | tags=27%, list=10%, signal=30% |
| 31 | KEGG\_PYRUVATE\_METABOLISM |  | 38 | 0.44 | 1.34 | 0.125 | 0.347 | 0.980 | 5953 | tags=47%, list=27%, signal=65% |
| 32 | KEGG\_TASTE\_TRANSDUCTION |  | 43 | 0.45 | 1.33 | 0.166 | 0.354 | 0.982 | 3998 | tags=21%, list=18%, signal=26% |
| 33 | KEGG\_MAPK\_SIGNALING\_PATHWAY |  | 256 | 0.36 | 1.33 | 0.020 | 0.351 | 0.983 | 2800 | tags=16%, list=13%, signal=18% |
| 34 | KEGG\_ENDOCYTOSIS |  | 171 | 0.36 | 1.32 | 0.061 | 0.358 | 0.987 | 4787 | tags=32%, list=22%, signal=40% |
| 35 | KEGG\_ARGININE\_AND\_PROLINE\_METABOLISM |  | 49 | 0.47 | 1.30 | 0.064 | 0.386 | 0.991 | 3254 | tags=29%, list=15%, signal=34% |
| 36 | KEGG\_TGF\_BETA\_SIGNALING\_PATHWAY |  | 82 | 0.43 | 1.30 | 0.075 | 0.385 | 0.993 | 3518 | tags=32%, list=16%, signal=38% |
| 37 | KEGG\_WNT\_SIGNALING\_PATHWAY |  | 145 | 0.40 | 1.28 | 0.097 | 0.401 | 0.994 | 4537 | tags=36%, list=21%, signal=45% |
| 38 | KEGG\_VIRAL\_MYOCARDITIS |  | 67 | 0.55 | 1.28 | 0.146 | 0.394 | 0.994 | 1047 | tags=18%, list=5%, signal=19% |
| 39 | KEGG\_ASCORBATE\_AND\_ALDARATE\_METABOLISM |  | 15 | 0.63 | 1.28 | 0.189 | 0.387 | 0.994 | 3750 | tags=40%, list=17%, signal=48% |
| 40 | KEGG\_PHENYLALANINE\_METABOLISM |  | 17 | 0.59 | 1.28 | 0.165 | 0.377 | 0.994 | 1492 | tags=29%, list=7%, signal=32% |
| 41 | KEGG\_REGULATION\_OF\_ACTIN\_CYTOSKELETON |  | 209 | 0.37 | 1.27 | 0.082 | 0.381 | 0.994 | 4777 | tags=32%, list=22%, signal=40% |
| 42 | KEGG\_FOCAL\_ADHESION |  | 195 | 0.48 | 1.27 | 0.141 | 0.377 | 0.995 | 2913 | tags=26%, list=13%, signal=30% |
| 43 | KEGG\_TYROSINE\_METABOLISM |  | 42 | 0.51 | 1.26 | 0.161 | 0.383 | 0.995 | 2452 | tags=24%, list=11%, signal=27% |
| 44 | KEGG\_PRIMARY\_BILE\_ACID\_BIOSYNTHESIS |  | 16 | 0.53 | 1.25 | 0.183 | 0.391 | 0.995 | 1567 | tags=13%, list=7%, signal=13% |
| 45 | KEGG\_GLYCEROPHOSPHOLIPID\_METABOLISM |  | 66 | 0.41 | 1.25 | 0.122 | 0.392 | 0.996 | 3438 | tags=26%, list=16%, signal=30% |
| 46 | KEGG\_ENDOMETRIAL\_CANCER |  | 52 | 0.37 | 1.24 | 0.133 | 0.405 | 0.997 | 5002 | tags=42%, list=23%, signal=55% |
| 47 | KEGG\_ARACHIDONIC\_ACID\_METABOLISM |  | 52 | 0.58 | 1.24 | 0.188 | 0.401 | 0.997 | 3564 | tags=40%, list=16%, signal=48% |
| 48 | KEGG\_FATTY\_ACID\_METABOLISM |  | 41 | 0.45 | 1.20 | 0.231 | 0.460 | 0.998 | 5044 | tags=54%, list=23%, signal=70% |
| 49 | KEGG\_ABC\_TRANSPORTERS |  | 42 | 0.46 | 1.20 | 0.210 | 0.457 | 0.998 | 1632 | tags=19%, list=8%, signal=21% |
| 50 | KEGG\_GLYCEROLIPID\_METABOLISM |  | 42 | 0.41 | 1.19 | 0.146 | 0.454 | 0.998 | 3984 | tags=29%, list=18%, signal=35% |
| 51 | KEGG\_MELANOMA |  | 71 | 0.41 | 1.19 | 0.188 | 0.453 | 0.998 | 4867 | tags=39%, list=22%, signal=51% |
| 52 | KEGG\_MTOR\_SIGNALING\_PATHWAY |  | 50 | 0.37 | 1.19 | 0.164 | 0.451 | 0.998 | 4861 | tags=32%, list=22%, signal=41% |
| 53 | KEGG\_GLUTATHIONE\_METABOLISM |  | 47 | 0.53 | 1.18 | 0.249 | 0.455 | 0.998 | 1923 | tags=26%, list=9%, signal=28% |
| 54 | KEGG\_ECM\_RECEPTOR\_INTERACTION |  | 81 | 0.54 | 1.17 | 0.289 | 0.474 | 0.998 | 2803 | tags=41%, list=13%, signal=47% |
| 55 | KEGG\_DRUG\_METABOLISM\_OTHER\_ENZYMES |  | 38 | 0.43 | 1.17 | 0.251 | 0.469 | 0.998 | 2187 | tags=13%, list=10%, signal=15% |
| 56 | KEGG\_PHOSPHATIDYLINOSITOL\_SIGNALING\_SYSTEM |  | 75 | 0.36 | 1.16 | 0.210 | 0.470 | 0.998 | 4979 | tags=40%, list=23%, signal=52% |
| 57 | KEGG\_NITROGEN\_METABOLISM |  | 22 | 0.47 | 1.16 | 0.219 | 0.464 | 0.998 | 3508 | tags=27%, list=16%, signal=32% |
| 58 | KEGG\_BASAL\_CELL\_CARCINOMA |  | 52 | 0.51 | 1.15 | 0.292 | 0.467 | 0.999 | 5002 | tags=48%, list=23%, signal=62% |
| 59 | KEGG\_GLYCOSAMINOGLYCAN\_BIOSYNTHESIS\_HEPARAN\_SULFATE |  | 26 | 0.48 | 1.15 | 0.288 | 0.471 | 0.999 | 4148 | tags=35%, list=19%, signal=43% |
| 60 | KEGG\_HISTIDINE\_METABOLISM |  | 28 | 0.48 | 1.14 | 0.312 | 0.475 | 0.999 | 4341 | tags=39%, list=20%, signal=49% |
| 61 | KEGG\_ACUTE\_MYELOID\_LEUKEMIA |  | 56 | 0.38 | 1.14 | 0.247 | 0.479 | 0.999 | 5002 | tags=45%, list=23%, signal=58% |
| 62 | KEGG\_LEUKOCYTE\_TRANSENDOTHELIAL\_MIGRATION |  | 113 | 0.42 | 1.13 | 0.298 | 0.491 | 0.999 | 2230 | tags=19%, list=10%, signal=21% |
| 63 | KEGG\_NEUROTROPHIN\_SIGNALING\_PATHWAY |  | 123 | 0.30 | 1.12 | 0.224 | 0.486 | 0.999 | 4887 | tags=33%, list=22%, signal=43% |
| 64 | KEGG\_BUTANOATE\_METABOLISM |  | 33 | 0.42 | 1.11 | 0.322 | 0.506 | 0.999 | 5069 | tags=42%, list=23%, signal=55% |
| 65 | KEGG\_THYROID\_CANCER |  | 29 | 0.38 | 1.07 | 0.363 | 0.582 | 1.000 | 5002 | tags=48%, list=23%, signal=63% |
| 66 | KEGG\_VALINE\_LEUCINE\_AND\_ISOLEUCINE\_DEGRADATION |  | 44 | 0.41 | 1.06 | 0.400 | 0.594 | 1.000 | 4765 | tags=45%, list=22%, signal=58% |
| 67 | KEGG\_HEDGEHOG\_SIGNALING\_PATHWAY |  | 53 | 0.41 | 1.05 | 0.378 | 0.596 | 1.000 | 3998 | tags=32%, list=18%, signal=39% |
| 68 | KEGG\_RIBOFLAVIN\_METABOLISM |  | 16 | 0.40 | 1.04 | 0.382 | 0.616 | 1.000 | 3493 | tags=25%, list=16%, signal=30% |
| 69 | KEGG\_PARKINSONS\_DISEASE |  | 113 | 0.26 | 1.04 | 0.408 | 0.609 | 1.000 | 1633 | tags=5%, list=8%, signal=6% |
| 70 | KEGG\_PENTOSE\_AND\_GLUCURONATE\_INTERCONVERSIONS |  | 17 | 0.48 | 1.03 | 0.464 | 0.606 | 1.000 | 3550 | tags=24%, list=16%, signal=28% |
| 71 | KEGG\_PPAR\_SIGNALING\_PATHWAY |  | 67 | 0.36 | 1.02 | 0.422 | 0.630 | 1.000 | 3956 | tags=30%, list=18%, signal=36% |
| 72 | KEGG\_NEUROACTIVE\_LIGAND\_RECEPTOR\_INTERACTION |  | 262 | 0.28 | 1.01 | 0.425 | 0.644 | 1.000 | 2751 | tags=12%, list=13%, signal=13% |
| 73 | KEGG\_GLYCOSAMINOGLYCAN\_BIOSYNTHESIS\_CHONDROITIN\_SULFATE |  | 22 | 0.49 | 1.01 | 0.449 | 0.635 | 1.000 | 2031 | tags=32%, list=9%, signal=35% |
| 74 | KEGG\_ALPHA\_LINOLENIC\_ACID\_METABOLISM |  | 15 | 0.50 | 1.00 | 0.472 | 0.645 | 1.000 | 1701 | tags=27%, list=8%, signal=29% |
| 75 | KEGG\_FRUCTOSE\_AND\_MANNOSE\_METABOLISM |  | 33 | 0.34 | 0.99 | 0.456 | 0.646 | 1.000 | 4196 | tags=30%, list=19%, signal=37% |
| 76 | KEGG\_NICOTINATE\_AND\_NICOTINAMIDE\_METABOLISM |  | 21 | 0.40 | 0.97 | 0.520 | 0.682 | 1.000 | 4848 | tags=43%, list=22%, signal=55% |
| 77 | KEGG\_ETHER\_LIPID\_METABOLISM |  | 26 | 0.38 | 0.93 | 0.558 | 0.765 | 1.000 | 2254 | tags=19%, list=10%, signal=21% |
| 78 | KEGG\_STEROID\_HORMONE\_BIOSYNTHESIS |  | 42 | 0.39 | 0.91 | 0.587 | 0.787 | 1.000 | 2310 | tags=17%, list=11%, signal=19% |
| 79 | KEGG\_REGULATION\_OF\_AUTOPHAGY |  | 34 | 0.28 | 0.91 | 0.602 | 0.780 | 1.000 | 4064 | tags=18%, list=19%, signal=22% |
| 80 | KEGG\_ERBB\_SIGNALING\_PATHWAY |  | 86 | 0.26 | 0.91 | 0.660 | 0.783 | 1.000 | 5631 | tags=40%, list=26%, signal=53% |
| 81 | KEGG\_BLADDER\_CANCER |  | 40 | 0.35 | 0.90 | 0.607 | 0.789 | 1.000 | 4711 | tags=40%, list=22%, signal=51% |
| 82 | KEGG\_P53\_SIGNALING\_PATHWAY |  | 65 | 0.31 | 0.90 | 0.623 | 0.782 | 1.000 | 2122 | tags=18%, list=10%, signal=20% |
| 83 | KEGG\_ADIPOCYTOKINE\_SIGNALING\_PATHWAY |  | 66 | 0.27 | 0.89 | 0.670 | 0.786 | 1.000 | 3515 | tags=23%, list=16%, signal=27% |
| 84 | KEGG\_VEGF\_SIGNALING\_PATHWAY |  | 71 | 0.28 | 0.89 | 0.722 | 0.785 | 1.000 | 4887 | tags=38%, list=22%, signal=49% |
| 85 | KEGG\_EPITHELIAL\_CELL\_SIGNALING\_IN\_HELICOBACTER\_PYLORI\_INFECTION |  | 66 | 0.28 | 0.88 | 0.669 | 0.778 | 1.000 | 2112 | tags=12%, list=10%, signal=13% |
| 86 | KEGG\_PATHWAYS\_IN\_CANCER |  | 319 | 0.27 | 0.87 | 0.718 | 0.795 | 1.000 | 4887 | tags=30%, list=22%, signal=38% |
| 87 | KEGG\_PURINE\_METABOLISM |  | 148 | 0.24 | 0.87 | 0.711 | 0.798 | 1.000 | 3832 | tags=19%, list=18%, signal=23% |
| 88 | KEGG\_TYPE\_II\_DIABETES\_MELLITUS |  | 44 | 0.28 | 0.86 | 0.694 | 0.795 | 1.000 | 4060 | tags=32%, list=19%, signal=39% |
| 89 | KEGG\_NON\_SMALL\_CELL\_LUNG\_CANCER |  | 54 | 0.25 | 0.85 | 0.821 | 0.812 | 1.000 | 4887 | tags=35%, list=22%, signal=45% |
| 90 | KEGG\_GLYCOSAMINOGLYCAN\_DEGRADATION |  | 20 | 0.33 | 0.85 | 0.694 | 0.805 | 1.000 | 4353 | tags=25%, list=20%, signal=31% |
| 91 | KEGG\_RIBOSOME |  | 71 | 0.21 | 0.85 | 0.604 | 0.797 | 1.000 | 281 | tags=1%, list=1%, signal=1% |
| 92 | KEGG\_HUNTINGTONS\_DISEASE |  | 168 | 0.20 | 0.84 | 0.682 | 0.804 | 1.000 | 2065 | tags=5%, list=9%, signal=6% |
| 93 | KEGG\_OXIDATIVE\_PHOSPHORYLATION |  | 117 | 0.22 | 0.84 | 0.671 | 0.799 | 1.000 | 1169 | tags=3%, list=5%, signal=4% |
| 94 | KEGG\_INOSITOL\_PHOSPHATE\_METABOLISM |  | 54 | 0.25 | 0.84 | 0.808 | 0.792 | 1.000 | 3291 | tags=20%, list=15%, signal=24% |
| 95 | KEGG\_PROSTATE\_CANCER |  | 89 | 0.25 | 0.82 | 0.785 | 0.805 | 1.000 | 5002 | tags=30%, list=23%, signal=39% |
| 96 | KEGG\_N\_GLYCAN\_BIOSYNTHESIS |  | 46 | 0.27 | 0.80 | 0.706 | 0.847 | 1.000 | 3372 | tags=22%, list=16%, signal=26% |
| 97 | KEGG\_PENTOSE\_PHOSPHATE\_PATHWAY |  | 26 | 0.31 | 0.80 | 0.736 | 0.838 | 1.000 | 4325 | tags=23%, list=20%, signal=29% |
| 98 | KEGG\_FC\_GAMMA\_R\_MEDIATED\_PHAGOCYTOSIS |  | 91 | 0.26 | 0.79 | 0.823 | 0.835 | 1.000 | 4887 | tags=35%, list=22%, signal=45% |
| 99 | KEGG\_VASOPRESSIN\_REGULATED\_WATER\_REABSORPTION |  | 44 | 0.25 | 0.77 | 0.843 | 0.864 | 1.000 | 6345 | tags=39%, list=29%, signal=54% |
| 100 | KEGG\_SNARE\_INTERACTIONS\_IN\_VESICULAR\_TRANSPORT |  | 38 | 0.23 | 0.77 | 0.834 | 0.856 | 1.000 | 5419 | tags=29%, list=25%, signal=38% |
| 101 | KEGG\_SPHINGOLIPID\_METABOLISM |  | 32 | 0.26 | 0.73 | 0.914 | 0.900 | 1.000 | 4359 | tags=28%, list=20%, signal=35% |
| 102 | KEGG\_AMINO\_SUGAR\_AND\_NUCLEOTIDE\_SUGAR\_METABOLISM |  | 42 | 0.24 | 0.72 | 0.893 | 0.905 | 1.000 | 4535 | tags=36%, list=21%, signal=45% |
| 103 | KEGG\_VIBRIO\_CHOLERAE\_INFECTION |  | 52 | 0.22 | 0.70 | 0.924 | 0.920 | 1.000 | 5746 | tags=29%, list=26%, signal=39% |
| 104 | KEGG\_PEROXISOME |  | 77 | 0.21 | 0.67 | 0.950 | 0.942 | 1.000 | 5964 | tags=39%, list=27%, signal=53% |
| 105 | KEGG\_LYSOSOME |  | 114 | 0.21 | 0.66 | 0.942 | 0.937 | 1.000 | 4359 | tags=20%, list=20%, signal=25% |
| 106 | KEGG\_RNA\_POLYMERASE |  | 28 | 0.20 | 0.56 | 0.933 | 0.980 | 1.000 | 1196 | tags=7%, list=5%, signal=8% |
Table: Gene sets enriched in phenotype **H (52 samples)**[plain text format]****

  
