# Supplementary material for: Identification and validation of key modules and hub genes associated with the pathological stage of oral squamous cell carcinoma by weighted gene co-expression network analysis
Source: PeerJ. 2020 Feb 4;8:e8505. doi: 10.7717/peerj.8505 (PMC7006519; doi:10.7717/peerj.8505)
Supplement: File S6 [file peerj-08-8505-s006.zip › my_analysis_209283_KEGG.Gsea.1570105930256/gsea_report_for_L_1570105930256.html]

Report for L 1570105930256 [GSEA]

| GS  follow link to MSigDB | GS DETAILS | SIZE | ES | NES | NOM p-val | FDR q-val | FWER p-val | RANK AT MAX | LEADING EDGE || 1 | KEGG\_ONE\_CARBON\_POOL\_BY\_FOLATE | Details ... | 16 | -0.69 | -1.74 | 0.000 | 0.202 | 0.190 | 2940 | tags=56%, list=14%, signal=65% |
| 2 | KEGG\_MISMATCH\_REPAIR | Details ... | 22 | -0.71 | -1.62 | 0.027 | 0.376 | 0.485 | 4228 | tags=59%, list=19%, signal=73% |
| 3 | KEGG\_PROTEASOME | Details ... | 41 | -0.60 | -1.57 | 0.081 | 0.406 | 0.635 | 7093 | tags=71%, list=33%, signal=105% |
| 4 | KEGG\_TOLL\_LIKE\_RECEPTOR\_SIGNALING\_PATHWAY | Details ... | 98 | -0.59 | -1.56 | 0.033 | 0.345 | 0.673 | 2744 | tags=32%, list=13%, signal=36% |
| 5 | KEGG\_SPLICEOSOME | Details ... | 114 | -0.46 | -1.56 | 0.078 | 0.277 | 0.675 | 6172 | tags=51%, list=28%, signal=71% |
| 6 | KEGG\_ANTIGEN\_PROCESSING\_AND\_PRESENTATION | Details ... | 80 | -0.59 | -1.56 | 0.062 | 0.237 | 0.684 | 5626 | tags=58%, list=26%, signal=77% |
| 7 | KEGG\_DNA\_REPLICATION | Details ... | 36 | -0.74 | -1.55 | 0.036 | 0.205 | 0.689 | 4228 | tags=78%, list=19%, signal=96% |
| 8 | KEGG\_HOMOLOGOUS\_RECOMBINATION | Details ... | 28 | -0.66 | -1.55 | 0.045 | 0.183 | 0.691 | 4176 | tags=50%, list=19%, signal=62% |
| 9 | KEGG\_GRAFT\_VERSUS\_HOST\_DISEASE | Details ... | 37 | -0.74 | -1.50 | 0.033 | 0.257 | 0.797 | 4446 | tags=78%, list=20%, signal=98% |
| 10 | KEGG\_CELL\_CYCLE | Details ... | 122 | -0.56 | -1.49 | 0.088 | 0.244 | 0.813 | 3323 | tags=42%, list=15%, signal=49% |
| 11 | KEGG\_GLYOXYLATE\_AND\_DICARBOXYLATE\_METABOLISM | Details ... | 16 | -0.54 | -1.48 | 0.066 | 0.251 | 0.835 | 3086 | tags=31%, list=14%, signal=36% |
| 12 | KEGG\_LEISHMANIA\_INFECTION | Details ... | 68 | -0.62 | -1.45 | 0.054 | 0.287 | 0.874 | 3250 | tags=47%, list=15%, signal=55% |
| 13 | KEGG\_TYPE\_I\_DIABETES\_MELLITUS | Details ... | 40 | -0.67 | -1.44 | 0.045 | 0.288 | 0.890 | 5008 | tags=73%, list=23%, signal=94% |
| 14 | KEGG\_AUTOIMMUNE\_THYROID\_DISEASE | Details ... | 49 | -0.66 | -1.42 | 0.125 | 0.298 | 0.913 | 5033 | tags=53%, list=23%, signal=69% |
| 15 | KEGG\_BASE\_EXCISION\_REPAIR | Details ... | 32 | -0.55 | -1.40 | 0.127 | 0.324 | 0.937 | 6175 | tags=56%, list=28%, signal=78% |
| 16 | KEGG\_AMINOACYL\_TRNA\_BIOSYNTHESIS | Details ... | 37 | -0.50 | -1.39 | 0.141 | 0.325 | 0.949 | 5661 | tags=51%, list=26%, signal=69% |
| 17 | KEGG\_NUCLEOTIDE\_EXCISION\_REPAIR | Details ... | 43 | -0.52 | -1.38 | 0.121 | 0.313 | 0.951 | 4452 | tags=49%, list=20%, signal=61% |
| 18 | KEGG\_ALLOGRAFT\_REJECTION | Details ... | 34 | -0.71 | -1.37 | 0.112 | 0.313 | 0.960 | 5033 | tags=76%, list=23%, signal=99% |
| 19 | KEGG\_RIG\_I\_LIKE\_RECEPTOR\_SIGNALING\_PATHWAY | Details ... | 69 | -0.46 | -1.36 | 0.105 | 0.319 | 0.965 | 5768 | tags=49%, list=27%, signal=67% |
| 20 | KEGG\_INTESTINAL\_IMMUNE\_NETWORK\_FOR\_IGA\_PRODUCTION | Details ... | 45 | -0.65 | -1.32 | 0.144 | 0.383 | 0.986 | 3250 | tags=47%, list=15%, signal=55% |
| 21 | KEGG\_CYSTEINE\_AND\_METHIONINE\_METABOLISM |  | 34 | -0.45 | -1.31 | 0.122 | 0.394 | 0.987 | 1109 | tags=15%, list=5%, signal=15% |
| 22 | KEGG\_PRIMARY\_IMMUNODEFICIENCY |  | 35 | -0.69 | -1.29 | 0.176 | 0.416 | 0.988 | 3822 | tags=57%, list=18%, signal=69% |
| 23 | KEGG\_PYRIMIDINE\_METABOLISM |  | 91 | -0.40 | -1.28 | 0.179 | 0.418 | 0.990 | 4151 | tags=35%, list=19%, signal=43% |
| 24 | KEGG\_PROTEIN\_EXPORT |  | 22 | -0.42 | -1.28 | 0.227 | 0.407 | 0.990 | 9065 | tags=64%, list=42%, signal=109% |
| 25 | KEGG\_NOD\_LIKE\_RECEPTOR\_SIGNALING\_PATHWAY |  | 59 | -0.53 | -1.28 | 0.177 | 0.393 | 0.990 | 2313 | tags=31%, list=11%, signal=34% |
| 26 | KEGG\_NATURAL\_KILLER\_CELL\_MEDIATED\_CYTOTOXICITY |  | 131 | -0.48 | -1.26 | 0.191 | 0.407 | 0.996 | 4446 | tags=44%, list=20%, signal=54% |
| 27 | KEGG\_RNA\_DEGRADATION |  | 56 | -0.39 | -1.25 | 0.211 | 0.419 | 0.997 | 4708 | tags=54%, list=22%, signal=68% |
| 28 | KEGG\_CYTOSOLIC\_DNA\_SENSING\_PATHWAY |  | 53 | -0.48 | -1.22 | 0.259 | 0.475 | 0.999 | 3849 | tags=36%, list=18%, signal=43% |
| 29 | KEGG\_SYSTEMIC\_LUPUS\_ERYTHEMATOSUS |  | 102 | -0.48 | -1.20 | 0.250 | 0.501 | 1.000 | 4674 | tags=48%, list=21%, signal=61% |
| 30 | KEGG\_CYTOKINE\_CYTOKINE\_RECEPTOR\_INTERACTION |  | 250 | -0.48 | -1.19 | 0.198 | 0.494 | 1.000 | 2615 | tags=34%, list=12%, signal=39% |
| 31 | KEGG\_TRYPTOPHAN\_METABOLISM |  | 39 | -0.45 | -1.19 | 0.175 | 0.486 | 1.000 | 2410 | tags=26%, list=11%, signal=29% |
| 32 | KEGG\_B\_CELL\_RECEPTOR\_SIGNALING\_PATHWAY |  | 74 | -0.45 | -1.15 | 0.277 | 0.565 | 1.000 | 4891 | tags=49%, list=22%, signal=63% |
| 33 | KEGG\_LYSINE\_DEGRADATION |  | 41 | -0.37 | -1.14 | 0.219 | 0.559 | 1.000 | 3199 | tags=27%, list=15%, signal=31% |
| 34 | KEGG\_UBIQUITIN\_MEDIATED\_PROTEOLYSIS |  | 129 | -0.29 | -1.13 | 0.262 | 0.562 | 1.000 | 3772 | tags=19%, list=17%, signal=23% |
| 35 | KEGG\_PROGESTERONE\_MEDIATED\_OOCYTE\_MATURATION |  | 83 | -0.35 | -1.12 | 0.286 | 0.564 | 1.000 | 1083 | tags=14%, list=5%, signal=15% |
| 36 | KEGG\_GALACTOSE\_METABOLISM |  | 25 | -0.39 | -1.12 | 0.295 | 0.568 | 1.000 | 1420 | tags=20%, list=7%, signal=21% |
| 37 | KEGG\_HEMATOPOIETIC\_CELL\_LINEAGE |  | 84 | -0.48 | -1.10 | 0.346 | 0.593 | 1.000 | 3331 | tags=40%, list=15%, signal=48% |
| 38 | KEGG\_JAK\_STAT\_SIGNALING\_PATHWAY |  | 151 | -0.39 | -1.10 | 0.323 | 0.580 | 1.000 | 3687 | tags=30%, list=17%, signal=36% |
| 39 | KEGG\_PRION\_DISEASES |  | 34 | -0.40 | -1.08 | 0.341 | 0.588 | 1.000 | 2139 | tags=24%, list=10%, signal=26% |
| 40 | KEGG\_MATURITY\_ONSET\_DIABETES\_OF\_THE\_YOUNG |  | 24 | -0.45 | -1.08 | 0.380 | 0.587 | 1.000 | 5414 | tags=33%, list=25%, signal=44% |
| 41 | KEGG\_APOPTOSIS |  | 86 | -0.36 | -1.07 | 0.339 | 0.597 | 1.000 | 3650 | tags=33%, list=17%, signal=39% |
| 42 | KEGG\_T\_CELL\_RECEPTOR\_SIGNALING\_PATHWAY |  | 106 | -0.40 | -1.05 | 0.419 | 0.629 | 1.000 | 4393 | tags=35%, list=20%, signal=44% |
| 43 | KEGG\_OOCYTE\_MEIOSIS |  | 107 | -0.29 | -1.04 | 0.395 | 0.620 | 1.000 | 1347 | tags=15%, list=6%, signal=16% |
| 44 | KEGG\_PANCREATIC\_CANCER |  | 69 | -0.32 | -1.02 | 0.441 | 0.663 | 1.000 | 2854 | tags=25%, list=13%, signal=28% |
| 45 | KEGG\_ASTHMA |  | 27 | -0.48 | -1.00 | 0.473 | 0.691 | 1.000 | 3250 | tags=44%, list=15%, signal=52% |
| 46 | KEGG\_FC\_EPSILON\_RI\_SIGNALING\_PATHWAY |  | 74 | -0.36 | -1.00 | 0.464 | 0.677 | 1.000 | 1521 | tags=19%, list=7%, signal=20% |
| 47 | KEGG\_CHEMOKINE\_SIGNALING\_PATHWAY |  | 180 | -0.37 | -0.97 | 0.477 | 0.710 | 1.000 | 4612 | tags=38%, list=21%, signal=48% |
| 48 | KEGG\_TERPENOID\_BACKBONE\_BIOSYNTHESIS |  | 15 | -0.44 | -0.95 | 0.524 | 0.757 | 1.000 | 4696 | tags=40%, list=22%, signal=51% |
| 49 | KEGG\_COLORECTAL\_CANCER |  | 62 | -0.30 | -0.93 | 0.568 | 0.784 | 1.000 | 5093 | tags=32%, list=23%, signal=42% |
| 50 | KEGG\_AMYOTROPHIC\_LATERAL\_SCLEROSIS\_ALS |  | 51 | -0.29 | -0.93 | 0.594 | 0.769 | 1.000 | 3530 | tags=24%, list=16%, signal=28% |
| 51 | KEGG\_PATHOGENIC\_ESCHERICHIA\_COLI\_INFECTION |  | 53 | -0.27 | -0.92 | 0.569 | 0.778 | 1.000 | 3635 | tags=15%, list=17%, signal=18% |
| 52 | KEGG\_ALANINE\_ASPARTATE\_AND\_GLUTAMATE\_METABOLISM |  | 32 | -0.34 | -0.89 | 0.626 | 0.813 | 1.000 | 1921 | tags=16%, list=9%, signal=17% |
| 53 | KEGG\_SMALL\_CELL\_LUNG\_CANCER |  | 84 | -0.31 | -0.88 | 0.638 | 0.834 | 1.000 | 3279 | tags=30%, list=15%, signal=35% |
| 54 | KEGG\_CELL\_ADHESION\_MOLECULES\_CAMS |  | 126 | -0.34 | -0.86 | 0.687 | 0.853 | 1.000 | 3262 | tags=33%, list=15%, signal=39% |
| 55 | KEGG\_PORPHYRIN\_AND\_CHLOROPHYLL\_METABOLISM |  | 30 | -0.31 | -0.81 | 0.696 | 0.929 | 1.000 | 3219 | tags=23%, list=15%, signal=27% |
| 56 | KEGG\_GLYCOSPHINGOLIPID\_BIOSYNTHESIS\_LACTO\_AND\_NEOLACTO\_SERIES |  | 25 | -0.37 | -0.81 | 0.710 | 0.919 | 1.000 | 3430 | tags=32%, list=16%, signal=38% |
| 57 | KEGG\_O\_GLYCAN\_BIOSYNTHESIS |  | 26 | -0.37 | -0.81 | 0.726 | 0.905 | 1.000 | 6791 | tags=50%, list=31%, signal=73% |
| 58 | KEGG\_COMPLEMENT\_AND\_COAGULATION\_CASCADES |  | 67 | -0.30 | -0.81 | 0.753 | 0.895 | 1.000 | 2080 | tags=19%, list=10%, signal=21% |
| 59 | KEGG\_GLYCINE\_SERINE\_AND\_THREONINE\_METABOLISM |  | 31 | -0.34 | -0.80 | 0.750 | 0.899 | 1.000 | 2739 | tags=26%, list=13%, signal=29% |
| 60 | KEGG\_DORSO\_VENTRAL\_AXIS\_FORMATION |  | 23 | -0.29 | -0.79 | 0.812 | 0.897 | 1.000 | 4311 | tags=39%, list=20%, signal=49% |
| 61 | KEGG\_BASAL\_TRANSCRIPTION\_FACTORS |  | 32 | -0.26 | -0.78 | 0.746 | 0.905 | 1.000 | 2747 | tags=19%, list=13%, signal=21% |
| 62 | KEGG\_CHRONIC\_MYELOID\_LEUKEMIA |  | 72 | -0.22 | -0.75 | 0.936 | 0.923 | 1.000 | 4891 | tags=32%, list=22%, signal=41% |
| 63 | KEGG\_PANTOTHENATE\_AND\_COA\_BIOSYNTHESIS |  | 16 | -0.32 | -0.74 | 0.858 | 0.924 | 1.000 | 2995 | tags=19%, list=14%, signal=22% |
| 64 | KEGG\_BIOSYNTHESIS\_OF\_UNSATURATED\_FATTY\_ACIDS |  | 18 | -0.30 | -0.74 | 0.813 | 0.910 | 1.000 | 1481 | tags=17%, list=7%, signal=18% |
| 65 | KEGG\_GLYCOSYLPHOSPHATIDYLINOSITOL\_GPI\_ANCHOR\_BIOSYNTHESIS |  | 24 | -0.25 | -0.69 | 0.876 | 0.958 | 1.000 | 5122 | tags=33%, list=24%, signal=44% |
| 66 | KEGG\_RENAL\_CELL\_CARCINOMA |  | 68 | -0.22 | -0.69 | 0.954 | 0.947 | 1.000 | 4919 | tags=26%, list=23%, signal=34% |
| 67 | KEGG\_CITRATE\_CYCLE\_TCA\_CYCLE |  | 30 | -0.21 | -0.62 | 0.905 | 0.988 | 1.000 | 3788 | tags=17%, list=17%, signal=20% |
| 68 | KEGG\_STEROID\_BIOSYNTHESIS |  | 15 | -0.28 | -0.61 | 0.956 | 0.977 | 1.000 | 3863 | tags=33%, list=18%, signal=41% |
| 69 | KEGG\_SELENOAMINO\_ACID\_METABOLISM |  | 25 | -0.22 | -0.57 | 0.970 | 0.978 | 1.000 | 3401 | tags=16%, list=16%, signal=19% |
Table: Gene sets enriched in phenotype **L (45 samples)**[plain text format]****

  
