# Supplementary material for: Identification and validation of key modules and hub genes associated with the pathological stage of oral squamous cell carcinoma by weighted gene co-expression network analysis
Source: PeerJ. 2020 Feb 4;8:e8505. doi: 10.7717/peerj.8505 (PMC7006519; doi:10.7717/peerj.8505)
Supplement: File S6 [file peerj-08-8505-s006.zip › my_analysis_213900_KEGG.Gsea.1570107185647/gsea_report_for_H_1570107185647.html]

Report for H 1570107185647 [GSEA]

| GS  follow link to MSigDB | GS DETAILS | SIZE | ES | NES | NOM p-val | FDR q-val | FWER p-val | RANK AT MAX | LEADING EDGE || 1 | KEGG\_VASOPRESSIN\_REGULATED\_WATER\_REABSORPTION | Details ... | 44 | 0.56 | 1.73 | 0.000 | 0.317 | 0.175 | 4552 | tags=34%, list=21%, signal=43% |
| 2 | KEGG\_TASTE\_TRANSDUCTION | Details ... | 43 | 0.57 | 1.67 | 0.014 | 0.359 | 0.344 | 3552 | tags=26%, list=16%, signal=31% |
| 3 | KEGG\_VALINE\_LEUCINE\_AND\_ISOLEUCINE\_DEGRADATION | Details ... | 44 | 0.58 | 1.57 | 0.029 | 0.698 | 0.629 | 4509 | tags=55%, list=21%, signal=69% |
| 4 | KEGG\_ALDOSTERONE\_REGULATED\_SODIUM\_REABSORPTION | Details ... | 41 | 0.61 | 1.55 | 0.017 | 0.614 | 0.670 | 2993 | tags=37%, list=14%, signal=42% |
| 5 | KEGG\_MELANOGENESIS | Details ... | 97 | 0.51 | 1.53 | 0.010 | 0.611 | 0.741 | 4199 | tags=36%, list=19%, signal=45% |
| 6 | KEGG\_VIBRIO\_CHOLERAE\_INFECTION | Details ... | 52 | 0.46 | 1.50 | 0.017 | 0.660 | 0.818 | 3575 | tags=23%, list=16%, signal=28% |
| 7 | KEGG\_INSULIN\_SIGNALING\_PATHWAY | Details ... | 135 | 0.42 | 1.50 | 0.017 | 0.575 | 0.824 | 5204 | tags=37%, list=24%, signal=48% |
| 8 | KEGG\_GNRH\_SIGNALING\_PATHWAY | Details ... | 94 | 0.45 | 1.47 | 0.041 | 0.626 | 0.875 | 5336 | tags=41%, list=25%, signal=55% |
| 9 | KEGG\_VASCULAR\_SMOOTH\_MUSCLE\_CONTRACTION | Details ... | 109 | 0.51 | 1.46 | 0.032 | 0.574 | 0.881 | 4020 | tags=34%, list=18%, signal=41% |
| 10 | KEGG\_TIGHT\_JUNCTION | Details ... | 128 | 0.48 | 1.45 | 0.026 | 0.556 | 0.886 | 3388 | tags=29%, list=16%, signal=34% |
| 11 | KEGG\_LINOLEIC\_ACID\_METABOLISM | Details ... | 24 | 0.74 | 1.45 | 0.059 | 0.522 | 0.895 | 1230 | tags=33%, list=6%, signal=35% |
| 12 | KEGG\_PROPANOATE\_METABOLISM | Details ... | 32 | 0.53 | 1.43 | 0.082 | 0.539 | 0.917 | 4476 | tags=50%, list=21%, signal=63% |
| 13 | KEGG\_CALCIUM\_SIGNALING\_PATHWAY | Details ... | 172 | 0.44 | 1.43 | 0.043 | 0.512 | 0.920 | 3841 | tags=26%, list=18%, signal=31% |
| 14 | KEGG\_SELENOAMINO\_ACID\_METABOLISM | Details ... | 25 | 0.52 | 1.42 | 0.091 | 0.488 | 0.921 | 4455 | tags=24%, list=20%, signal=30% |
| 15 | KEGG\_LONG\_TERM\_DEPRESSION | Details ... | 65 | 0.46 | 1.40 | 0.039 | 0.525 | 0.945 | 4551 | tags=37%, list=21%, signal=47% |
| 16 | KEGG\_LONG\_TERM\_POTENTIATION | Details ... | 68 | 0.39 | 1.38 | 0.022 | 0.559 | 0.959 | 5336 | tags=40%, list=25%, signal=52% |
| 17 | KEGG\_ARACHIDONIC\_ACID\_METABOLISM | Details ... | 52 | 0.64 | 1.38 | 0.062 | 0.526 | 0.959 | 3245 | tags=40%, list=15%, signal=47% |
| 18 | KEGG\_ALPHA\_LINOLENIC\_ACID\_METABOLISM | Details ... | 15 | 0.68 | 1.35 | 0.122 | 0.597 | 0.969 | 3245 | tags=47%, list=15%, signal=55% |
| 19 | KEGG\_MATURITY\_ONSET\_DIABETES\_OF\_THE\_YOUNG | Details ... | 24 | 0.57 | 1.34 | 0.139 | 0.600 | 0.971 | 1214 | tags=13%, list=6%, signal=13% |
| 20 | KEGG\_PEROXISOME | Details ... | 77 | 0.40 | 1.34 | 0.095 | 0.593 | 0.975 | 4921 | tags=35%, list=23%, signal=45% |
| 21 | KEGG\_RIBOFLAVIN\_METABOLISM |  | 16 | 0.50 | 1.33 | 0.111 | 0.599 | 0.980 | 1572 | tags=19%, list=7%, signal=20% |
| 22 | KEGG\_PHENYLALANINE\_METABOLISM |  | 17 | 0.60 | 1.32 | 0.142 | 0.602 | 0.981 | 2029 | tags=29%, list=9%, signal=32% |
| 23 | KEGG\_GLYCOSPHINGOLIPID\_BIOSYNTHESIS\_LACTO\_AND\_NEOLACTO\_SERIES |  | 25 | 0.59 | 1.32 | 0.138 | 0.577 | 0.982 | 1081 | tags=24%, list=5%, signal=25% |
| 24 | KEGG\_O\_GLYCAN\_BIOSYNTHESIS |  | 26 | 0.61 | 1.32 | 0.158 | 0.553 | 0.982 | 804 | tags=23%, list=4%, signal=24% |
| 25 | KEGG\_BUTANOATE\_METABOLISM |  | 33 | 0.49 | 1.32 | 0.136 | 0.535 | 0.982 | 4509 | tags=42%, list=21%, signal=53% |
| 26 | KEGG\_DRUG\_METABOLISM\_CYTOCHROME\_P450 |  | 59 | 0.62 | 1.29 | 0.160 | 0.585 | 0.990 | 4635 | tags=53%, list=21%, signal=67% |
| 27 | KEGG\_EPITHELIAL\_CELL\_SIGNALING\_IN\_HELICOBACTER\_PYLORI\_INFECTION |  | 66 | 0.40 | 1.28 | 0.103 | 0.584 | 0.991 | 1716 | tags=15%, list=8%, signal=16% |
| 28 | KEGG\_HISTIDINE\_METABOLISM |  | 28 | 0.51 | 1.27 | 0.153 | 0.606 | 0.991 | 4455 | tags=50%, list=20%, signal=63% |
| 29 | KEGG\_ABC\_TRANSPORTERS |  | 42 | 0.47 | 1.24 | 0.167 | 0.680 | 0.994 | 2530 | tags=24%, list=12%, signal=27% |
| 30 | KEGG\_PPAR\_SIGNALING\_PATHWAY |  | 67 | 0.43 | 1.23 | 0.112 | 0.683 | 0.997 | 4032 | tags=37%, list=19%, signal=46% |
| 31 | KEGG\_NEUROTROPHIN\_SIGNALING\_PATHWAY |  | 123 | 0.33 | 1.23 | 0.096 | 0.661 | 0.997 | 4708 | tags=31%, list=22%, signal=39% |
| 32 | KEGG\_GLYCINE\_SERINE\_AND\_THREONINE\_METABOLISM |  | 31 | 0.52 | 1.23 | 0.218 | 0.657 | 0.998 | 3272 | tags=32%, list=15%, signal=38% |
| 33 | KEGG\_AXON\_GUIDANCE |  | 127 | 0.37 | 1.22 | 0.117 | 0.664 | 0.998 | 3078 | tags=24%, list=14%, signal=28% |
| 34 | KEGG\_FATTY\_ACID\_METABOLISM |  | 41 | 0.44 | 1.20 | 0.206 | 0.684 | 0.999 | 4593 | tags=46%, list=21%, signal=59% |
| 35 | KEGG\_PRIMARY\_BILE\_ACID\_BIOSYNTHESIS |  | 16 | 0.50 | 1.19 | 0.223 | 0.700 | 0.999 | 2224 | tags=25%, list=10%, signal=28% |
| 36 | KEGG\_RETINOL\_METABOLISM |  | 49 | 0.53 | 1.19 | 0.251 | 0.692 | 0.999 | 4427 | tags=43%, list=20%, signal=54% |
| 37 | KEGG\_RENIN\_ANGIOTENSIN\_SYSTEM |  | 17 | 0.57 | 1.18 | 0.240 | 0.690 | 1.000 | 1189 | tags=29%, list=5%, signal=31% |
| 38 | KEGG\_REGULATION\_OF\_AUTOPHAGY |  | 34 | 0.35 | 1.16 | 0.282 | 0.732 | 1.000 | 4981 | tags=24%, list=23%, signal=30% |
| 39 | KEGG\_LEUKOCYTE\_TRANSENDOTHELIAL\_MIGRATION |  | 113 | 0.43 | 1.16 | 0.264 | 0.729 | 1.000 | 2683 | tags=25%, list=12%, signal=28% |
| 40 | KEGG\_OXIDATIVE\_PHOSPHORYLATION |  | 117 | 0.30 | 1.15 | 0.278 | 0.717 | 1.000 | 1206 | tags=6%, list=6%, signal=6% |
| 41 | KEGG\_MTOR\_SIGNALING\_PATHWAY |  | 50 | 0.36 | 1.14 | 0.221 | 0.747 | 1.000 | 3027 | tags=24%, list=14%, signal=28% |
| 42 | KEGG\_CELL\_ADHESION\_MOLECULES\_CAMS |  | 126 | 0.45 | 1.13 | 0.280 | 0.757 | 1.000 | 3985 | tags=34%, list=18%, signal=42% |
| 43 | KEGG\_METABOLISM\_OF\_XENOBIOTICS\_BY\_CYTOCHROME\_P450 |  | 56 | 0.56 | 1.13 | 0.320 | 0.740 | 1.000 | 4542 | tags=50%, list=21%, signal=63% |
| 44 | KEGG\_BETA\_ALANINE\_METABOLISM |  | 22 | 0.41 | 1.12 | 0.292 | 0.737 | 1.000 | 4476 | tags=41%, list=21%, signal=51% |
| 45 | KEGG\_PROSTATE\_CANCER |  | 89 | 0.34 | 1.12 | 0.237 | 0.726 | 1.000 | 4552 | tags=34%, list=21%, signal=42% |
| 46 | KEGG\_NEUROACTIVE\_LIGAND\_RECEPTOR\_INTERACTION |  | 262 | 0.31 | 1.12 | 0.227 | 0.726 | 1.000 | 4157 | tags=18%, list=19%, signal=22% |
| 47 | KEGG\_CARDIAC\_MUSCLE\_CONTRACTION |  | 73 | 0.47 | 1.12 | 0.362 | 0.714 | 1.000 | 4389 | tags=27%, list=20%, signal=34% |
| 48 | KEGG\_TYROSINE\_METABOLISM |  | 42 | 0.43 | 1.11 | 0.300 | 0.703 | 1.000 | 4455 | tags=36%, list=20%, signal=45% |
| 49 | KEGG\_GAP\_JUNCTION |  | 87 | 0.34 | 1.10 | 0.304 | 0.727 | 1.000 | 3766 | tags=24%, list=17%, signal=29% |
| 50 | KEGG\_PANTOTHENATE\_AND\_COA\_BIOSYNTHESIS |  | 16 | 0.47 | 1.10 | 0.320 | 0.721 | 1.000 | 541 | tags=13%, list=2%, signal=13% |
| 51 | KEGG\_ETHER\_LIPID\_METABOLISM |  | 26 | 0.46 | 1.09 | 0.349 | 0.733 | 1.000 | 3245 | tags=31%, list=15%, signal=36% |
| 52 | KEGG\_ENDOMETRIAL\_CANCER |  | 52 | 0.32 | 1.07 | 0.304 | 0.750 | 1.000 | 4551 | tags=33%, list=21%, signal=41% |
| 53 | KEGG\_AMINO\_SUGAR\_AND\_NUCLEOTIDE\_SUGAR\_METABOLISM |  | 42 | 0.35 | 1.07 | 0.335 | 0.743 | 1.000 | 5384 | tags=33%, list=25%, signal=44% |
| 54 | KEGG\_ACUTE\_MYELOID\_LEUKEMIA |  | 56 | 0.37 | 1.07 | 0.349 | 0.734 | 1.000 | 4020 | tags=34%, list=18%, signal=42% |
| 55 | KEGG\_GLYCOSAMINOGLYCAN\_DEGRADATION |  | 20 | 0.42 | 1.06 | 0.383 | 0.740 | 1.000 | 5486 | tags=45%, list=25%, signal=60% |
| 56 | KEGG\_PHOSPHATIDYLINOSITOL\_SIGNALING\_SYSTEM |  | 75 | 0.32 | 1.05 | 0.366 | 0.748 | 1.000 | 5336 | tags=35%, list=25%, signal=46% |
| 57 | KEGG\_WNT\_SIGNALING\_PATHWAY |  | 145 | 0.33 | 1.05 | 0.339 | 0.740 | 1.000 | 4185 | tags=25%, list=19%, signal=31% |
| 58 | KEGG\_DILATED\_CARDIOMYOPATHY |  | 89 | 0.47 | 1.05 | 0.442 | 0.733 | 1.000 | 4222 | tags=39%, list=19%, signal=49% |
| 59 | KEGG\_GLYCEROLIPID\_METABOLISM |  | 42 | 0.36 | 1.04 | 0.360 | 0.740 | 1.000 | 2884 | tags=26%, list=13%, signal=30% |
| 60 | KEGG\_PROXIMAL\_TUBULE\_BICARBONATE\_RECLAMATION |  | 22 | 0.41 | 1.04 | 0.424 | 0.738 | 1.000 | 4464 | tags=36%, list=21%, signal=46% |
| 61 | KEGG\_BASAL\_CELL\_CARCINOMA |  | 52 | 0.44 | 1.02 | 0.428 | 0.779 | 1.000 | 3943 | tags=29%, list=18%, signal=35% |
| 62 | KEGG\_GLYCEROPHOSPHOLIPID\_METABOLISM |  | 66 | 0.33 | 1.01 | 0.419 | 0.771 | 1.000 | 3740 | tags=21%, list=17%, signal=26% |
| 63 | KEGG\_ALANINE\_ASPARTATE\_AND\_GLUTAMATE\_METABOLISM |  | 32 | 0.38 | 1.01 | 0.441 | 0.766 | 1.000 | 3092 | tags=31%, list=14%, signal=36% |
| 64 | KEGG\_ALZHEIMERS\_DISEASE |  | 154 | 0.26 | 1.00 | 0.422 | 0.777 | 1.000 | 3476 | tags=14%, list=16%, signal=16% |
| 65 | KEGG\_N\_GLYCAN\_BIOSYNTHESIS |  | 46 | 0.34 | 0.99 | 0.466 | 0.794 | 1.000 | 1338 | tags=9%, list=6%, signal=9% |
| 66 | KEGG\_SPHINGOLIPID\_METABOLISM |  | 32 | 0.35 | 0.98 | 0.477 | 0.798 | 1.000 | 3267 | tags=25%, list=15%, signal=29% |
| 67 | KEGG\_MAPK\_SIGNALING\_PATHWAY |  | 256 | 0.27 | 0.97 | 0.506 | 0.806 | 1.000 | 3860 | tags=21%, list=18%, signal=26% |
| 68 | KEGG\_FC\_EPSILON\_RI\_SIGNALING\_PATHWAY |  | 74 | 0.35 | 0.97 | 0.490 | 0.799 | 1.000 | 4526 | tags=31%, list=21%, signal=39% |
| 69 | KEGG\_TYPE\_II\_DIABETES\_MELLITUS |  | 44 | 0.31 | 0.96 | 0.509 | 0.815 | 1.000 | 2993 | tags=18%, list=14%, signal=21% |
| 70 | KEGG\_ADHERENS\_JUNCTION |  | 67 | 0.29 | 0.96 | 0.529 | 0.806 | 1.000 | 4735 | tags=30%, list=22%, signal=38% |
| 71 | KEGG\_INOSITOL\_PHOSPHATE\_METABOLISM |  | 54 | 0.29 | 0.96 | 0.531 | 0.795 | 1.000 | 2515 | tags=15%, list=12%, signal=17% |
| 72 | KEGG\_PYRUVATE\_METABOLISM |  | 38 | 0.31 | 0.94 | 0.518 | 0.822 | 1.000 | 3641 | tags=24%, list=17%, signal=28% |
| 73 | KEGG\_GLUTATHIONE\_METABOLISM |  | 47 | 0.41 | 0.93 | 0.550 | 0.839 | 1.000 | 3708 | tags=28%, list=17%, signal=33% |
| 74 | KEGG\_ARRHYTHMOGENIC\_RIGHT\_VENTRICULAR\_CARDIOMYOPATHY\_ARVC |  | 73 | 0.37 | 0.92 | 0.552 | 0.854 | 1.000 | 4222 | tags=34%, list=19%, signal=42% |
| 75 | KEGG\_NICOTINATE\_AND\_NICOTINAMIDE\_METABOLISM |  | 21 | 0.38 | 0.92 | 0.577 | 0.848 | 1.000 | 1694 | tags=24%, list=8%, signal=26% |
| 76 | KEGG\_ADIPOCYTOKINE\_SIGNALING\_PATHWAY |  | 66 | 0.28 | 0.91 | 0.633 | 0.859 | 1.000 | 4876 | tags=32%, list=22%, signal=41% |
| 77 | KEGG\_ENDOCYTOSIS |  | 171 | 0.25 | 0.90 | 0.676 | 0.874 | 1.000 | 4233 | tags=20%, list=19%, signal=25% |
| 78 | KEGG\_MELANOMA |  | 71 | 0.30 | 0.90 | 0.618 | 0.864 | 1.000 | 4020 | tags=28%, list=18%, signal=34% |
| 79 | KEGG\_VEGF\_SIGNALING\_PATHWAY |  | 71 | 0.28 | 0.88 | 0.739 | 0.896 | 1.000 | 4197 | tags=27%, list=19%, signal=33% |
| 80 | KEGG\_GLIOMA |  | 64 | 0.26 | 0.87 | 0.747 | 0.895 | 1.000 | 4020 | tags=27%, list=18%, signal=32% |
| 81 | KEGG\_NITROGEN\_METABOLISM |  | 22 | 0.35 | 0.87 | 0.712 | 0.893 | 1.000 | 815 | tags=14%, list=4%, signal=14% |
| 82 | KEGG\_PARKINSONS\_DISEASE |  | 113 | 0.22 | 0.87 | 0.620 | 0.890 | 1.000 | 2585 | tags=8%, list=12%, signal=9% |
| 83 | KEGG\_HUNTINGTONS\_DISEASE |  | 168 | 0.20 | 0.87 | 0.655 | 0.881 | 1.000 | 1819 | tags=6%, list=8%, signal=6% |
| 84 | KEGG\_HYPERTROPHIC\_CARDIOMYOPATHY\_HCM |  | 82 | 0.40 | 0.86 | 0.636 | 0.875 | 1.000 | 4222 | tags=37%, list=19%, signal=45% |
| 85 | KEGG\_BIOSYNTHESIS\_OF\_UNSATURATED\_FATTY\_ACIDS |  | 18 | 0.34 | 0.85 | 0.644 | 0.895 | 1.000 | 2779 | tags=22%, list=13%, signal=25% |
| 86 | KEGG\_TGF\_BETA\_SIGNALING\_PATHWAY |  | 82 | 0.28 | 0.84 | 0.764 | 0.910 | 1.000 | 3397 | tags=21%, list=16%, signal=24% |
| 87 | KEGG\_TRYPTOPHAN\_METABOLISM |  | 39 | 0.31 | 0.84 | 0.790 | 0.904 | 1.000 | 4493 | tags=36%, list=21%, signal=45% |
| 88 | KEGG\_ASTHMA |  | 27 | 0.40 | 0.82 | 0.665 | 0.925 | 1.000 | 4511 | tags=37%, list=21%, signal=47% |
| 89 | KEGG\_GLYCOLYSIS\_GLUCONEOGENESIS |  | 60 | 0.28 | 0.82 | 0.762 | 0.916 | 1.000 | 1645 | tags=13%, list=8%, signal=14% |
| 90 | KEGG\_TERPENOID\_BACKBONE\_BIOSYNTHESIS |  | 15 | 0.38 | 0.82 | 0.673 | 0.911 | 1.000 | 290 | tags=7%, list=1%, signal=7% |
| 91 | KEGG\_STARCH\_AND\_SUCROSE\_METABOLISM |  | 37 | 0.29 | 0.81 | 0.774 | 0.908 | 1.000 | 509 | tags=8%, list=2%, signal=8% |
| 92 | KEGG\_INTESTINAL\_IMMUNE\_NETWORK\_FOR\_IGA\_PRODUCTION |  | 45 | 0.39 | 0.81 | 0.710 | 0.913 | 1.000 | 3134 | tags=22%, list=14%, signal=26% |
| 93 | KEGG\_COMPLEMENT\_AND\_COAGULATION\_CASCADES |  | 67 | 0.31 | 0.80 | 0.758 | 0.908 | 1.000 | 4068 | tags=31%, list=19%, signal=38% |
| 94 | KEGG\_LYSOSOME |  | 114 | 0.25 | 0.78 | 0.775 | 0.945 | 1.000 | 5608 | tags=35%, list=26%, signal=47% |
| 95 | KEGG\_HEDGEHOG\_SIGNALING\_PATHWAY |  | 53 | 0.30 | 0.77 | 0.823 | 0.943 | 1.000 | 1975 | tags=13%, list=9%, signal=14% |
| 96 | KEGG\_STEROID\_HORMONE\_BIOSYNTHESIS |  | 42 | 0.33 | 0.77 | 0.824 | 0.936 | 1.000 | 4189 | tags=31%, list=19%, signal=38% |
| 97 | KEGG\_NOTCH\_SIGNALING\_PATHWAY |  | 46 | 0.22 | 0.77 | 0.834 | 0.939 | 1.000 | 1121 | tags=7%, list=5%, signal=7% |
| 98 | KEGG\_B\_CELL\_RECEPTOR\_SIGNALING\_PATHWAY |  | 74 | 0.28 | 0.74 | 0.799 | 0.969 | 1.000 | 4597 | tags=31%, list=21%, signal=39% |
| 99 | KEGG\_CHEMOKINE\_SIGNALING\_PATHWAY |  | 180 | 0.28 | 0.72 | 0.873 | 0.985 | 1.000 | 2105 | tags=14%, list=10%, signal=15% |
| 100 | KEGG\_REGULATION\_OF\_ACTIN\_CYTOSKELETON |  | 209 | 0.21 | 0.72 | 0.990 | 0.975 | 1.000 | 4091 | tags=20%, list=19%, signal=25% |
| 101 | KEGG\_OLFACTORY\_TRANSDUCTION |  | 111 | 0.16 | 0.70 | 0.841 | 0.996 | 1.000 | 3406 | tags=7%, list=16%, signal=9% |
| 102 | KEGG\_VIRAL\_MYOCARDITIS |  | 67 | 0.29 | 0.68 | 0.921 | 1.000 | 1.000 | 3374 | tags=25%, list=16%, signal=30% |
| 103 | KEGG\_FC\_GAMMA\_R\_MEDIATED\_PHAGOCYTOSIS |  | 91 | 0.22 | 0.67 | 0.964 | 0.999 | 1.000 | 4711 | tags=25%, list=22%, signal=32% |
| 104 | KEGG\_THYROID\_CANCER |  | 29 | 0.24 | 0.67 | 0.944 | 0.993 | 1.000 | 4582 | tags=31%, list=21%, signal=39% |
| 105 | KEGG\_ASCORBATE\_AND\_ALDARATE\_METABOLISM |  | 15 | 0.30 | 0.61 | 0.926 | 1.000 | 1.000 | 3638 | tags=33%, list=17%, signal=40% |
| 106 | KEGG\_TYPE\_I\_DIABETES\_MELLITUS |  | 40 | 0.29 | 0.61 | 0.945 | 1.000 | 1.000 | 3134 | tags=20%, list=14%, signal=23% |
| 107 | KEGG\_PENTOSE\_AND\_GLUCURONATE\_INTERCONVERSIONS |  | 17 | 0.28 | 0.60 | 0.922 | 1.000 | 1.000 | 5481 | tags=41%, list=25%, signal=55% |
| 108 | KEGG\_GLYCOSYLPHOSPHATIDYLINOSITOL\_GPI\_ANCHOR\_BIOSYNTHESIS |  | 24 | 0.21 | 0.58 | 0.977 | 1.000 | 1.000 | 583 | tags=4%, list=3%, signal=4% |
| 109 | KEGG\_T\_CELL\_RECEPTOR\_SIGNALING\_PATHWAY |  | 106 | 0.21 | 0.55 | 0.988 | 1.000 | 1.000 | 4548 | tags=25%, list=21%, signal=31% |
| 110 | KEGG\_CITRATE\_CYCLE\_TCA\_CYCLE |  | 30 | 0.17 | 0.50 | 0.981 | 1.000 | 1.000 | 5667 | tags=27%, list=26%, signal=36% |
| 111 | KEGG\_RIBOSOME |  | 71 | 0.12 | 0.45 | 0.963 | 1.000 | 1.000 | 1352 | tags=3%, list=6%, signal=3% |
| 112 | KEGG\_ANTIGEN\_PROCESSING\_AND\_PRESENTATION |  | 80 | 0.16 | 0.42 | 0.989 | 0.998 | 1.000 | 4308 | tags=20%, list=20%, signal=25% |
Table: Gene sets enriched in phenotype **H (24 samples)**[plain text format]****

  
