# Supplementary material for: Identification and validation of key modules and hub genes associated with the pathological stage of oral squamous cell carcinoma by weighted gene co-expression network analysis
Source: PeerJ. 2020 Feb 4;8:e8505. doi: 10.7717/peerj.8505 (PMC7006519; doi:10.7717/peerj.8505)
Supplement: File S6 [file peerj-08-8505-s006.zip › my_analysis_213900_KEGG.Gsea.1570107185647/gsea_report_for_L_1570107185647.html]

Report for L 1570107185647 [GSEA]

| GS  follow link to MSigDB | GS DETAILS | SIZE | ES | NES | NOM p-val | FDR q-val | FWER p-val | RANK AT MAX | LEADING EDGE || 1 | KEGG\_SPLICEOSOME | Details ... | 114 | -0.55 | -1.90 | 0.004 | 0.030 | 0.038 | 6382 | tags=57%, list=29%, signal=80% |
| 2 | KEGG\_BASAL\_TRANSCRIPTION\_FACTORS | Details ... | 32 | -0.59 | -1.79 | 0.010 | 0.051 | 0.112 | 4240 | tags=53%, list=19%, signal=66% |
| 3 | KEGG\_CELL\_CYCLE | Details ... | 122 | -0.65 | -1.75 | 0.002 | 0.047 | 0.151 | 3883 | tags=54%, list=18%, signal=65% |
| 4 | KEGG\_RNA\_DEGRADATION | Details ... | 56 | -0.53 | -1.68 | 0.023 | 0.082 | 0.292 | 5531 | tags=59%, list=25%, signal=79% |
| 5 | KEGG\_HOMOLOGOUS\_RECOMBINATION | Details ... | 28 | -0.72 | -1.66 | 0.004 | 0.082 | 0.346 | 3183 | tags=64%, list=15%, signal=75% |
| 6 | KEGG\_AMINOACYL\_TRNA\_BIOSYNTHESIS | Details ... | 37 | -0.59 | -1.64 | 0.026 | 0.082 | 0.379 | 5639 | tags=57%, list=26%, signal=76% |
| 7 | KEGG\_RNA\_POLYMERASE | Details ... | 28 | -0.55 | -1.61 | 0.029 | 0.099 | 0.476 | 7436 | tags=79%, list=34%, signal=119% |
| 8 | KEGG\_PROTEASOME | Details ... | 41 | -0.61 | -1.61 | 0.067 | 0.088 | 0.477 | 5768 | tags=71%, list=27%, signal=96% |
| 9 | KEGG\_PYRIMIDINE\_METABOLISM | Details ... | 91 | -0.49 | -1.55 | 0.035 | 0.149 | 0.675 | 5313 | tags=55%, list=24%, signal=72% |
| 10 | KEGG\_DNA\_REPLICATION | Details ... | 36 | -0.73 | -1.54 | 0.035 | 0.141 | 0.690 | 4294 | tags=75%, list=20%, signal=93% |
| 11 | KEGG\_MISMATCH\_REPAIR | Details ... | 22 | -0.69 | -1.54 | 0.031 | 0.131 | 0.701 | 2650 | tags=55%, list=12%, signal=62% |
| 12 | KEGG\_P53\_SIGNALING\_PATHWAY | Details ... | 65 | -0.53 | -1.52 | 0.025 | 0.140 | 0.749 | 4206 | tags=46%, list=19%, signal=57% |
| 13 | KEGG\_OOCYTE\_MEIOSIS | Details ... | 107 | -0.41 | -1.50 | 0.025 | 0.160 | 0.806 | 2083 | tags=22%, list=10%, signal=25% |
| 14 | KEGG\_NUCLEOTIDE\_EXCISION\_REPAIR | Details ... | 43 | -0.56 | -1.48 | 0.067 | 0.175 | 0.846 | 4625 | tags=51%, list=21%, signal=65% |
| 15 | KEGG\_GALACTOSE\_METABOLISM | Details ... | 25 | -0.50 | -1.45 | 0.054 | 0.207 | 0.896 | 4153 | tags=28%, list=19%, signal=35% |
| 16 | KEGG\_GLYOXYLATE\_AND\_DICARBOXYLATE\_METABOLISM | Details ... | 16 | -0.51 | -1.43 | 0.097 | 0.229 | 0.927 | 4648 | tags=38%, list=21%, signal=48% |
| 17 | KEGG\_CYSTEINE\_AND\_METHIONINE\_METABOLISM | Details ... | 34 | -0.47 | -1.42 | 0.064 | 0.221 | 0.929 | 2290 | tags=21%, list=11%, signal=23% |
| 18 | KEGG\_UBIQUITIN\_MEDIATED\_PROTEOLYSIS | Details ... | 129 | -0.36 | -1.39 | 0.092 | 0.266 | 0.958 | 5129 | tags=35%, list=24%, signal=45% |
| 19 | KEGG\_BASE\_EXCISION\_REPAIR | Details ... | 32 | -0.53 | -1.34 | 0.164 | 0.342 | 0.981 | 4294 | tags=44%, list=20%, signal=54% |
| 20 | KEGG\_RENAL\_CELL\_CARCINOMA | Details ... | 68 | -0.41 | -1.30 | 0.112 | 0.407 | 0.989 | 4848 | tags=40%, list=22%, signal=51% |
| 21 | KEGG\_PROGESTERONE\_MEDIATED\_OOCYTE\_MATURATION |  | 83 | -0.38 | -1.25 | 0.144 | 0.492 | 0.996 | 1387 | tags=20%, list=6%, signal=22% |
| 22 | KEGG\_BLADDER\_CANCER |  | 40 | -0.46 | -1.24 | 0.163 | 0.509 | 0.998 | 3463 | tags=40%, list=16%, signal=47% |
| 23 | KEGG\_SYSTEMIC\_LUPUS\_ERYTHEMATOSUS |  | 102 | -0.45 | -1.16 | 0.284 | 0.689 | 0.999 | 3028 | tags=30%, list=14%, signal=35% |
| 24 | KEGG\_PANCREATIC\_CANCER |  | 69 | -0.36 | -1.14 | 0.242 | 0.715 | 0.999 | 3466 | tags=29%, list=16%, signal=34% |
| 25 | KEGG\_FRUCTOSE\_AND\_MANNOSE\_METABOLISM |  | 33 | -0.39 | -1.12 | 0.271 | 0.727 | 0.999 | 4153 | tags=30%, list=19%, signal=37% |
| 26 | KEGG\_SMALL\_CELL\_LUNG\_CANCER |  | 84 | -0.39 | -1.11 | 0.301 | 0.731 | 0.999 | 3015 | tags=29%, list=14%, signal=33% |
| 27 | KEGG\_AMYOTROPHIC\_LATERAL\_SCLEROSIS\_ALS |  | 51 | -0.33 | -1.05 | 0.361 | 0.876 | 1.000 | 956 | tags=12%, list=4%, signal=12% |
| 28 | KEGG\_PURINE\_METABOLISM |  | 148 | -0.29 | -1.04 | 0.397 | 0.892 | 1.000 | 4297 | tags=36%, list=20%, signal=45% |
| 29 | KEGG\_DRUG\_METABOLISM\_OTHER\_ENZYMES |  | 38 | -0.39 | -1.03 | 0.426 | 0.870 | 1.000 | 2834 | tags=26%, list=13%, signal=30% |
| 30 | KEGG\_NOD\_LIKE\_RECEPTOR\_SIGNALING\_PATHWAY |  | 59 | -0.42 | -1.03 | 0.419 | 0.863 | 1.000 | 6319 | tags=51%, list=29%, signal=71% |
| 31 | KEGG\_ERBB\_SIGNALING\_PATHWAY |  | 86 | -0.29 | -1.02 | 0.437 | 0.841 | 1.000 | 2061 | tags=17%, list=9%, signal=19% |
| 32 | KEGG\_PRION\_DISEASES |  | 34 | -0.35 | -0.96 | 0.524 | 0.993 | 1.000 | 1037 | tags=15%, list=5%, signal=15% |
| 33 | KEGG\_RIG\_I\_LIKE\_RECEPTOR\_SIGNALING\_PATHWAY |  | 69 | -0.32 | -0.96 | 0.494 | 0.977 | 1.000 | 1360 | tags=13%, list=6%, signal=14% |
| 34 | KEGG\_PROTEIN\_EXPORT |  | 22 | -0.32 | -0.95 | 0.488 | 0.954 | 1.000 | 4804 | tags=32%, list=22%, signal=41% |
| 35 | KEGG\_ONE\_CARBON\_POOL\_BY\_FOLATE |  | 16 | -0.38 | -0.95 | 0.533 | 0.930 | 1.000 | 4648 | tags=56%, list=21%, signal=71% |
| 36 | KEGG\_PENTOSE\_PHOSPHATE\_PATHWAY |  | 26 | -0.36 | -0.93 | 0.548 | 0.958 | 1.000 | 4153 | tags=38%, list=19%, signal=47% |
| 37 | KEGG\_CYTOSOLIC\_DNA\_SENSING\_PATHWAY |  | 53 | -0.36 | -0.92 | 0.562 | 0.956 | 1.000 | 2610 | tags=21%, list=12%, signal=24% |
| 38 | KEGG\_PATHWAYS\_IN\_CANCER |  | 319 | -0.28 | -0.91 | 0.608 | 0.975 | 1.000 | 3185 | tags=22%, list=15%, signal=25% |
| 39 | KEGG\_CHRONIC\_MYELOID\_LEUKEMIA |  | 72 | -0.26 | -0.88 | 0.695 | 1.000 | 1.000 | 4206 | tags=32%, list=19%, signal=39% |
| 40 | KEGG\_GLYCOSAMINOGLYCAN\_BIOSYNTHESIS\_CHONDROITIN\_SULFATE |  | 22 | -0.43 | -0.88 | 0.625 | 0.993 | 1.000 | 4087 | tags=41%, list=19%, signal=50% |
| 41 | KEGG\_TOLL\_LIKE\_RECEPTOR\_SIGNALING\_PATHWAY |  | 98 | -0.33 | -0.88 | 0.601 | 0.974 | 1.000 | 1632 | tags=16%, list=8%, signal=18% |
| 42 | KEGG\_ARGININE\_AND\_PROLINE\_METABOLISM |  | 49 | -0.31 | -0.86 | 0.755 | 1.000 | 1.000 | 2016 | tags=20%, list=9%, signal=22% |
| 43 | KEGG\_NON\_SMALL\_CELL\_LUNG\_CANCER |  | 54 | -0.25 | -0.86 | 0.741 | 0.981 | 1.000 | 2779 | tags=19%, list=13%, signal=21% |
| 44 | KEGG\_COLORECTAL\_CANCER |  | 62 | -0.27 | -0.85 | 0.703 | 0.979 | 1.000 | 3466 | tags=21%, list=16%, signal=25% |
| 45 | KEGG\_SNARE\_INTERACTIONS\_IN\_VESICULAR\_TRANSPORT |  | 38 | -0.24 | -0.81 | 0.751 | 1.000 | 1.000 | 3579 | tags=26%, list=16%, signal=31% |
| 46 | KEGG\_DORSO\_VENTRAL\_AXIS\_FORMATION |  | 23 | -0.29 | -0.80 | 0.800 | 1.000 | 1.000 | 3395 | tags=35%, list=16%, signal=41% |
| 47 | KEGG\_GRAFT\_VERSUS\_HOST\_DISEASE |  | 37 | -0.40 | -0.80 | 0.729 | 1.000 | 1.000 | 2420 | tags=24%, list=11%, signal=27% |
| 48 | KEGG\_JAK\_STAT\_SIGNALING\_PATHWAY |  | 151 | -0.28 | -0.79 | 0.781 | 1.000 | 1.000 | 3176 | tags=20%, list=15%, signal=23% |
| 49 | KEGG\_APOPTOSIS |  | 86 | -0.26 | -0.78 | 0.846 | 1.000 | 1.000 | 2531 | tags=19%, list=12%, signal=21% |
| 50 | KEGG\_GLYCOSAMINOGLYCAN\_BIOSYNTHESIS\_HEPARAN\_SULFATE |  | 26 | -0.31 | -0.76 | 0.807 | 1.000 | 1.000 | 2704 | tags=23%, list=12%, signal=26% |
| 51 | KEGG\_HEMATOPOIETIC\_CELL\_LINEAGE |  | 84 | -0.33 | -0.75 | 0.797 | 1.000 | 1.000 | 2118 | tags=21%, list=10%, signal=24% |
| 52 | KEGG\_ECM\_RECEPTOR\_INTERACTION |  | 81 | -0.33 | -0.75 | 0.781 | 1.000 | 1.000 | 1131 | tags=19%, list=5%, signal=19% |
| 53 | KEGG\_CYTOKINE\_CYTOKINE\_RECEPTOR\_INTERACTION |  | 250 | -0.29 | -0.71 | 0.911 | 1.000 | 1.000 | 1508 | tags=17%, list=7%, signal=18% |
| 54 | KEGG\_FOCAL\_ADHESION |  | 195 | -0.26 | -0.70 | 0.903 | 1.000 | 1.000 | 2596 | tags=19%, list=12%, signal=22% |
| 55 | KEGG\_PATHOGENIC\_ESCHERICHIA\_COLI\_INFECTION |  | 53 | -0.20 | -0.70 | 0.924 | 1.000 | 1.000 | 6834 | tags=43%, list=31%, signal=63% |
| 56 | KEGG\_LYSINE\_DEGRADATION |  | 41 | -0.23 | -0.69 | 0.975 | 1.000 | 1.000 | 5346 | tags=32%, list=25%, signal=42% |
| 57 | KEGG\_STEROID\_BIOSYNTHESIS |  | 15 | -0.31 | -0.66 | 0.930 | 1.000 | 1.000 | 573 | tags=13%, list=3%, signal=14% |
| 58 | KEGG\_PORPHYRIN\_AND\_CHLOROPHYLL\_METABOLISM |  | 30 | -0.26 | -0.66 | 0.907 | 1.000 | 1.000 | 2682 | tags=23%, list=12%, signal=27% |
| 59 | KEGG\_LEISHMANIA\_INFECTION |  | 68 | -0.27 | -0.64 | 0.921 | 1.000 | 1.000 | 5239 | tags=34%, list=24%, signal=44% |
| 60 | KEGG\_NATURAL\_KILLER\_CELL\_MEDIATED\_CYTOTOXICITY |  | 131 | -0.21 | -0.57 | 0.979 | 1.000 | 1.000 | 3655 | tags=18%, list=17%, signal=21% |
| 61 | KEGG\_PRIMARY\_IMMUNODEFICIENCY |  | 35 | -0.28 | -0.54 | 0.941 | 1.000 | 1.000 | 3373 | tags=29%, list=16%, signal=34% |
| 62 | KEGG\_ALLOGRAFT\_REJECTION |  | 34 | -0.26 | -0.51 | 0.966 | 1.000 | 1.000 | 4464 | tags=29%, list=21%, signal=37% |
| 63 | KEGG\_AUTOIMMUNE\_THYROID\_DISEASE |  | 49 | -0.16 | -0.36 | 0.988 | 1.000 | 1.000 | 7844 | tags=41%, list=36%, signal=64% |
Table: Gene sets enriched in phenotype **L (73 samples)**[plain text format]****

  
