# Supplementary material for: Identification and validation of key modules and hub genes associated with the pathological stage of oral squamous cell carcinoma by weighted gene co-expression network analysis
Source: PeerJ. 2020 Feb 4;8:e8505. doi: 10.7717/peerj.8505 (PMC7006519; doi:10.7717/peerj.8505)
Supplement: File S6 [file peerj-08-8505-s006.zip › my_analysis_231773_kegg.Gsea.1570107186948/gsea_report_for_H_1570107186948.html]

Report for H 1570107186948 [GSEA]

| GS  follow link to MSigDB | GS DETAILS | SIZE | ES | NES | NOM p-val | FDR q-val | FWER p-val | RANK AT MAX | LEADING EDGE || 1 | KEGG\_VASCULAR\_SMOOTH\_MUSCLE\_CONTRACTION | Details ... | 109 | 0.59 | 1.73 | 0.000 | 0.299 | 0.177 | 3691 | tags=40%, list=17%, signal=48% |
| 2 | KEGG\_CARDIAC\_MUSCLE\_CONTRACTION | Details ... | 73 | 0.72 | 1.65 | 0.033 | 0.424 | 0.394 | 1504 | tags=26%, list=7%, signal=28% |
| 3 | KEGG\_LEUKOCYTE\_TRANSENDOTHELIAL\_MIGRATION | Details ... | 113 | 0.60 | 1.62 | 0.008 | 0.415 | 0.495 | 2821 | tags=35%, list=13%, signal=40% |
| 4 | KEGG\_CALCIUM\_SIGNALING\_PATHWAY | Details ... | 172 | 0.49 | 1.59 | 0.004 | 0.412 | 0.581 | 3697 | tags=30%, list=17%, signal=36% |
| 5 | KEGG\_ALDOSTERONE\_REGULATED\_SODIUM\_REABSORPTION | Details ... | 41 | 0.64 | 1.58 | 0.002 | 0.347 | 0.597 | 1771 | tags=29%, list=8%, signal=32% |
| 6 | KEGG\_DILATED\_CARDIOMYOPATHY | Details ... | 89 | 0.70 | 1.55 | 0.027 | 0.398 | 0.672 | 2276 | tags=42%, list=10%, signal=46% |
| 7 | KEGG\_VIRAL\_MYOCARDITIS | Details ... | 67 | 0.65 | 1.55 | 0.020 | 0.350 | 0.680 | 2276 | tags=37%, list=10%, signal=42% |
| 8 | KEGG\_PROXIMAL\_TUBULE\_BICARBONATE\_RECLAMATION | Details ... | 22 | 0.60 | 1.52 | 0.037 | 0.395 | 0.741 | 401 | tags=14%, list=2%, signal=14% |
| 9 | KEGG\_PHOSPHATIDYLINOSITOL\_SIGNALING\_SYSTEM | Details ... | 75 | 0.45 | 1.49 | 0.012 | 0.458 | 0.823 | 4387 | tags=41%, list=20%, signal=52% |
| 10 | KEGG\_HYPERTROPHIC\_CARDIOMYOPATHY\_HCM | Details ... | 82 | 0.68 | 1.46 | 0.046 | 0.499 | 0.864 | 2276 | tags=41%, list=10%, signal=46% |
| 11 | KEGG\_REGULATION\_OF\_AUTOPHAGY | Details ... | 34 | 0.43 | 1.45 | 0.091 | 0.484 | 0.871 | 4492 | tags=26%, list=21%, signal=33% |
| 12 | KEGG\_LONG\_TERM\_DEPRESSION | Details ... | 65 | 0.48 | 1.45 | 0.020 | 0.453 | 0.879 | 4532 | tags=37%, list=21%, signal=47% |
| 13 | KEGG\_MTOR\_SIGNALING\_PATHWAY | Details ... | 50 | 0.45 | 1.44 | 0.019 | 0.443 | 0.893 | 1771 | tags=20%, list=8%, signal=22% |
| 14 | KEGG\_TIGHT\_JUNCTION | Details ... | 128 | 0.48 | 1.44 | 0.033 | 0.423 | 0.899 | 2464 | tags=23%, list=11%, signal=25% |
| 15 | KEGG\_CELL\_ADHESION\_MOLECULES\_CAMS | Details ... | 126 | 0.58 | 1.44 | 0.061 | 0.408 | 0.901 | 2595 | tags=37%, list=12%, signal=41% |
| 16 | KEGG\_FC\_GAMMA\_R\_MEDIATED\_PHAGOCYTOSIS | Details ... | 91 | 0.48 | 1.44 | 0.050 | 0.387 | 0.903 | 2709 | tags=29%, list=12%, signal=32% |
| 17 | KEGG\_INSULIN\_SIGNALING\_PATHWAY | Details ... | 135 | 0.41 | 1.43 | 0.026 | 0.377 | 0.908 | 2509 | tags=20%, list=12%, signal=22% |
| 18 | KEGG\_NEUROACTIVE\_LIGAND\_RECEPTOR\_INTERACTION | Details ... | 262 | 0.40 | 1.40 | 0.028 | 0.445 | 0.930 | 2818 | tags=15%, list=13%, signal=17% |
| 19 | KEGG\_LYSOSOME | Details ... | 114 | 0.43 | 1.40 | 0.094 | 0.431 | 0.932 | 4495 | tags=42%, list=21%, signal=53% |
| 20 | KEGG\_ARRHYTHMOGENIC\_RIGHT\_VENTRICULAR\_CARDIOMYOPATHY\_ARVC | Details ... | 73 | 0.57 | 1.39 | 0.087 | 0.415 | 0.934 | 2276 | tags=29%, list=10%, signal=32% |
| 21 | KEGG\_RENIN\_ANGIOTENSIN\_SYSTEM |  | 17 | 0.68 | 1.39 | 0.063 | 0.397 | 0.936 | 2102 | tags=47%, list=10%, signal=52% |
| 22 | KEGG\_TYPE\_II\_DIABETES\_MELLITUS |  | 44 | 0.46 | 1.37 | 0.062 | 0.429 | 0.950 | 2787 | tags=23%, list=13%, signal=26% |
| 23 | KEGG\_COMPLEMENT\_AND\_COAGULATION\_CASCADES |  | 67 | 0.54 | 1.37 | 0.068 | 0.412 | 0.950 | 2911 | tags=39%, list=13%, signal=45% |
| 24 | KEGG\_PRIMARY\_IMMUNODEFICIENCY |  | 35 | 0.70 | 1.36 | 0.140 | 0.425 | 0.961 | 3308 | tags=54%, list=15%, signal=64% |
| 25 | KEGG\_ASTHMA |  | 27 | 0.66 | 1.35 | 0.152 | 0.443 | 0.967 | 3753 | tags=63%, list=17%, signal=76% |
| 26 | KEGG\_INTESTINAL\_IMMUNE\_NETWORK\_FOR\_IGA\_PRODUCTION |  | 45 | 0.65 | 1.33 | 0.152 | 0.462 | 0.977 | 4005 | tags=60%, list=18%, signal=73% |
| 27 | KEGG\_FC\_EPSILON\_RI\_SIGNALING\_PATHWAY |  | 74 | 0.48 | 1.33 | 0.119 | 0.454 | 0.977 | 4408 | tags=42%, list=20%, signal=52% |
| 28 | KEGG\_AXON\_GUIDANCE |  | 127 | 0.41 | 1.32 | 0.054 | 0.448 | 0.979 | 2898 | tags=24%, list=13%, signal=28% |
| 29 | KEGG\_B\_CELL\_RECEPTOR\_SIGNALING\_PATHWAY |  | 74 | 0.51 | 1.32 | 0.151 | 0.434 | 0.979 | 3755 | tags=42%, list=17%, signal=50% |
| 30 | KEGG\_AUTOIMMUNE\_THYROID\_DISEASE |  | 49 | 0.62 | 1.32 | 0.198 | 0.431 | 0.981 | 4480 | tags=45%, list=21%, signal=56% |
| 31 | KEGG\_ACUTE\_MYELOID\_LEUKEMIA |  | 56 | 0.45 | 1.31 | 0.076 | 0.442 | 0.984 | 1771 | tags=20%, list=8%, signal=21% |
| 32 | KEGG\_FOCAL\_ADHESION |  | 195 | 0.48 | 1.30 | 0.152 | 0.447 | 0.986 | 2694 | tags=31%, list=12%, signal=35% |
| 33 | KEGG\_GAP\_JUNCTION |  | 87 | 0.40 | 1.29 | 0.098 | 0.448 | 0.987 | 3578 | tags=30%, list=16%, signal=36% |
| 34 | KEGG\_REGULATION\_OF\_ACTIN\_CYTOSKELETON |  | 209 | 0.37 | 1.28 | 0.094 | 0.464 | 0.989 | 1957 | tags=17%, list=9%, signal=19% |
| 35 | KEGG\_GNRH\_SIGNALING\_PATHWAY |  | 94 | 0.40 | 1.28 | 0.125 | 0.458 | 0.989 | 4532 | tags=33%, list=21%, signal=41% |
| 36 | KEGG\_LONG\_TERM\_POTENTIATION |  | 68 | 0.37 | 1.26 | 0.106 | 0.487 | 0.993 | 3697 | tags=28%, list=17%, signal=34% |
| 37 | KEGG\_ALPHA\_LINOLENIC\_ACID\_METABOLISM |  | 15 | 0.63 | 1.26 | 0.170 | 0.484 | 0.994 | 4408 | tags=40%, list=20%, signal=50% |
| 38 | KEGG\_PRIMARY\_BILE\_ACID\_BIOSYNTHESIS |  | 16 | 0.52 | 1.24 | 0.185 | 0.513 | 0.995 | 1518 | tags=25%, list=7%, signal=27% |
| 39 | KEGG\_NICOTINATE\_AND\_NICOTINAMIDE\_METABOLISM |  | 21 | 0.50 | 1.24 | 0.144 | 0.501 | 0.995 | 2031 | tags=24%, list=9%, signal=26% |
| 40 | KEGG\_CHEMOKINE\_SIGNALING\_PATHWAY |  | 180 | 0.47 | 1.22 | 0.201 | 0.520 | 0.995 | 2865 | tags=31%, list=13%, signal=35% |
| 41 | KEGG\_T\_CELL\_RECEPTOR\_SIGNALING\_PATHWAY |  | 106 | 0.46 | 1.22 | 0.241 | 0.512 | 0.995 | 2920 | tags=32%, list=13%, signal=37% |
| 42 | KEGG\_MELANOGENESIS |  | 97 | 0.41 | 1.22 | 0.148 | 0.515 | 0.995 | 3578 | tags=28%, list=16%, signal=33% |
| 43 | KEGG\_NATURAL\_KILLER\_CELL\_MEDIATED\_CYTOTOXICITY |  | 131 | 0.45 | 1.21 | 0.250 | 0.513 | 0.995 | 4214 | tags=37%, list=19%, signal=45% |
| 44 | KEGG\_ECM\_RECEPTOR\_INTERACTION |  | 81 | 0.54 | 1.21 | 0.234 | 0.510 | 0.995 | 2190 | tags=38%, list=10%, signal=42% |
| 45 | KEGG\_ALLOGRAFT\_REJECTION |  | 34 | 0.62 | 1.21 | 0.299 | 0.504 | 0.995 | 4480 | tags=65%, list=21%, signal=81% |
| 46 | KEGG\_ANTIGEN\_PROCESSING\_AND\_PRESENTATION |  | 80 | 0.46 | 1.20 | 0.285 | 0.499 | 0.995 | 2639 | tags=23%, list=12%, signal=26% |
| 47 | KEGG\_GLIOMA |  | 64 | 0.36 | 1.20 | 0.132 | 0.491 | 0.995 | 1840 | tags=19%, list=8%, signal=20% |
| 48 | KEGG\_ETHER\_LIPID\_METABOLISM |  | 26 | 0.50 | 1.20 | 0.184 | 0.485 | 0.996 | 651 | tags=12%, list=3%, signal=12% |
| 49 | KEGG\_INOSITOL\_PHOSPHATE\_METABOLISM |  | 54 | 0.36 | 1.18 | 0.185 | 0.508 | 0.998 | 4387 | tags=41%, list=20%, signal=51% |
| 50 | KEGG\_TASTE\_TRANSDUCTION |  | 43 | 0.40 | 1.18 | 0.239 | 0.500 | 0.998 | 2482 | tags=14%, list=11%, signal=16% |
| 51 | KEGG\_NEUROTROPHIN\_SIGNALING\_PATHWAY |  | 123 | 0.32 | 1.17 | 0.179 | 0.510 | 0.998 | 4204 | tags=29%, list=19%, signal=36% |
| 52 | KEGG\_ADIPOCYTOKINE\_SIGNALING\_PATHWAY |  | 66 | 0.37 | 1.17 | 0.195 | 0.508 | 0.998 | 2360 | tags=18%, list=11%, signal=20% |
| 53 | KEGG\_NITROGEN\_METABOLISM |  | 22 | 0.47 | 1.17 | 0.237 | 0.502 | 0.998 | 1968 | tags=23%, list=9%, signal=25% |
| 54 | KEGG\_ABC\_TRANSPORTERS |  | 42 | 0.45 | 1.16 | 0.223 | 0.509 | 0.999 | 3341 | tags=21%, list=15%, signal=25% |
| 55 | KEGG\_VEGF\_SIGNALING\_PATHWAY |  | 71 | 0.36 | 1.14 | 0.220 | 0.537 | 0.999 | 4448 | tags=38%, list=20%, signal=48% |
| 56 | KEGG\_MELANOMA |  | 71 | 0.39 | 1.14 | 0.246 | 0.534 | 0.999 | 1855 | tags=20%, list=9%, signal=21% |
| 57 | KEGG\_TYROSINE\_METABOLISM |  | 42 | 0.44 | 1.13 | 0.290 | 0.542 | 0.999 | 326 | tags=7%, list=1%, signal=7% |
| 58 | KEGG\_ALZHEIMERS\_DISEASE |  | 154 | 0.29 | 1.13 | 0.272 | 0.535 | 0.999 | 3347 | tags=16%, list=15%, signal=18% |
| 59 | KEGG\_HEMATOPOIETIC\_CELL\_LINEAGE |  | 84 | 0.49 | 1.12 | 0.324 | 0.548 | 0.999 | 3420 | tags=43%, list=16%, signal=51% |
| 60 | KEGG\_ENDOCYTOSIS |  | 171 | 0.31 | 1.11 | 0.269 | 0.550 | 0.999 | 3106 | tags=19%, list=14%, signal=22% |
| 61 | KEGG\_MAPK\_SIGNALING\_PATHWAY |  | 256 | 0.30 | 1.10 | 0.250 | 0.576 | 0.999 | 2512 | tags=15%, list=12%, signal=17% |
| 62 | KEGG\_PANTOTHENATE\_AND\_COA\_BIOSYNTHESIS |  | 16 | 0.47 | 1.09 | 0.308 | 0.576 | 0.999 | 3898 | tags=38%, list=18%, signal=46% |
| 63 | KEGG\_TGF\_BETA\_SIGNALING\_PATHWAY |  | 82 | 0.36 | 1.09 | 0.289 | 0.571 | 0.999 | 1823 | tags=15%, list=8%, signal=16% |
| 64 | KEGG\_MATURITY\_ONSET\_DIABETES\_OF\_THE\_YOUNG |  | 24 | 0.46 | 1.09 | 0.353 | 0.573 | 0.999 | 625 | tags=8%, list=3%, signal=9% |
| 65 | KEGG\_WNT\_SIGNALING\_PATHWAY |  | 145 | 0.34 | 1.08 | 0.298 | 0.571 | 0.999 | 2958 | tags=19%, list=14%, signal=22% |
| 66 | KEGG\_ARACHIDONIC\_ACID\_METABOLISM |  | 52 | 0.51 | 1.08 | 0.368 | 0.566 | 0.999 | 1924 | tags=23%, list=9%, signal=25% |
| 67 | KEGG\_PROSTATE\_CANCER |  | 89 | 0.33 | 1.08 | 0.314 | 0.558 | 0.999 | 3153 | tags=22%, list=14%, signal=26% |
| 68 | KEGG\_LEISHMANIA\_INFECTION |  | 68 | 0.46 | 1.07 | 0.399 | 0.579 | 0.999 | 2184 | tags=28%, list=10%, signal=31% |
| 69 | KEGG\_N\_GLYCAN\_BIOSYNTHESIS |  | 46 | 0.35 | 1.06 | 0.373 | 0.591 | 0.999 | 2230 | tags=13%, list=10%, signal=15% |
| 70 | KEGG\_ADHERENS\_JUNCTION |  | 67 | 0.31 | 1.05 | 0.351 | 0.585 | 0.999 | 2863 | tags=21%, list=13%, signal=24% |
| 71 | KEGG\_VIBRIO\_CHOLERAE\_INFECTION |  | 52 | 0.32 | 1.05 | 0.398 | 0.584 | 0.999 | 4622 | tags=29%, list=21%, signal=37% |
| 72 | KEGG\_GLYCOSAMINOGLYCAN\_DEGRADATION |  | 20 | 0.41 | 1.04 | 0.408 | 0.592 | 0.999 | 5852 | tags=60%, list=27%, signal=82% |
| 73 | KEGG\_TRYPTOPHAN\_METABOLISM |  | 39 | 0.39 | 1.03 | 0.398 | 0.602 | 0.999 | 1246 | tags=10%, list=6%, signal=11% |
| 74 | KEGG\_PPAR\_SIGNALING\_PATHWAY |  | 67 | 0.37 | 1.03 | 0.378 | 0.597 | 1.000 | 3006 | tags=21%, list=14%, signal=24% |
| 75 | KEGG\_GLYCOLYSIS\_GLUCONEOGENESIS |  | 60 | 0.36 | 1.03 | 0.410 | 0.594 | 1.000 | 1016 | tags=10%, list=5%, signal=10% |
| 76 | KEGG\_ENDOMETRIAL\_CANCER |  | 52 | 0.32 | 1.02 | 0.435 | 0.610 | 1.000 | 4325 | tags=35%, list=20%, signal=43% |
| 77 | KEGG\_PARKINSONS\_DISEASE |  | 113 | 0.25 | 1.00 | 0.442 | 0.645 | 1.000 | 2808 | tags=7%, list=13%, signal=8% |
| 78 | KEGG\_BETA\_ALANINE\_METABOLISM |  | 22 | 0.36 | 1.00 | 0.443 | 0.637 | 1.000 | 89 | tags=5%, list=0%, signal=5% |
| 79 | KEGG\_TYPE\_I\_DIABETES\_MELLITUS |  | 40 | 0.47 | 1.00 | 0.476 | 0.629 | 1.000 | 4200 | tags=55%, list=19%, signal=68% |
| 80 | KEGG\_STARCH\_AND\_SUCROSE\_METABOLISM |  | 37 | 0.36 | 0.98 | 0.482 | 0.663 | 1.000 | 563 | tags=8%, list=3%, signal=8% |
| 81 | KEGG\_NOTCH\_SIGNALING\_PATHWAY |  | 46 | 0.28 | 0.97 | 0.499 | 0.657 | 1.000 | 2436 | tags=13%, list=11%, signal=15% |
| 82 | KEGG\_RIBOFLAVIN\_METABOLISM |  | 16 | 0.37 | 0.96 | 0.512 | 0.676 | 1.000 | 4281 | tags=44%, list=20%, signal=54% |
| 83 | KEGG\_VALINE\_LEUCINE\_AND\_ISOLEUCINE\_DEGRADATION |  | 44 | 0.36 | 0.96 | 0.524 | 0.672 | 1.000 | 4815 | tags=41%, list=22%, signal=52% |
| 84 | KEGG\_COLORECTAL\_CANCER |  | 62 | 0.30 | 0.94 | 0.531 | 0.708 | 1.000 | 3153 | tags=23%, list=14%, signal=26% |
| 85 | KEGG\_PROPANOATE\_METABOLISM |  | 32 | 0.35 | 0.93 | 0.529 | 0.709 | 1.000 | 5437 | tags=44%, list=25%, signal=58% |
| 86 | KEGG\_LINOLEIC\_ACID\_METABOLISM |  | 24 | 0.48 | 0.91 | 0.579 | 0.747 | 1.000 | 5242 | tags=42%, list=24%, signal=55% |
| 87 | KEGG\_CYTOKINE\_CYTOKINE\_RECEPTOR\_INTERACTION |  | 250 | 0.36 | 0.90 | 0.610 | 0.754 | 1.000 | 3101 | tags=26%, list=14%, signal=30% |
| 88 | KEGG\_NON\_SMALL\_CELL\_LUNG\_CANCER |  | 54 | 0.27 | 0.90 | 0.698 | 0.745 | 1.000 | 4267 | tags=33%, list=20%, signal=41% |
| 89 | KEGG\_APOPTOSIS |  | 86 | 0.30 | 0.87 | 0.657 | 0.794 | 1.000 | 2377 | tags=17%, list=11%, signal=20% |
| 90 | KEGG\_GLYCEROPHOSPHOLIPID\_METABOLISM |  | 66 | 0.28 | 0.87 | 0.715 | 0.787 | 1.000 | 3282 | tags=15%, list=15%, signal=18% |
| 91 | KEGG\_JAK\_STAT\_SIGNALING\_PATHWAY |  | 151 | 0.30 | 0.87 | 0.672 | 0.788 | 1.000 | 2444 | tags=19%, list=11%, signal=21% |
| 92 | KEGG\_VASOPRESSIN\_REGULATED\_WATER\_REABSORPTION |  | 44 | 0.28 | 0.86 | 0.665 | 0.788 | 1.000 | 4648 | tags=25%, list=21%, signal=32% |
| 93 | KEGG\_GRAFT\_VERSUS\_HOST\_DISEASE |  | 37 | 0.43 | 0.86 | 0.661 | 0.792 | 1.000 | 4214 | tags=57%, list=19%, signal=70% |
| 94 | KEGG\_FATTY\_ACID\_METABOLISM |  | 41 | 0.32 | 0.85 | 0.672 | 0.786 | 1.000 | 5233 | tags=37%, list=24%, signal=48% |
| 95 | KEGG\_TOLL\_LIKE\_RECEPTOR\_SIGNALING\_PATHWAY |  | 98 | 0.33 | 0.85 | 0.669 | 0.779 | 1.000 | 2944 | tags=18%, list=14%, signal=21% |
| 96 | KEGG\_SYSTEMIC\_LUPUS\_ERYTHEMATOSUS |  | 102 | 0.34 | 0.84 | 0.677 | 0.794 | 1.000 | 3336 | tags=26%, list=15%, signal=31% |
| 97 | KEGG\_PRION\_DISEASES |  | 34 | 0.32 | 0.84 | 0.731 | 0.786 | 1.000 | 2612 | tags=26%, list=12%, signal=30% |
| 98 | KEGG\_OXIDATIVE\_PHOSPHORYLATION |  | 117 | 0.22 | 0.83 | 0.665 | 0.800 | 1.000 | 483 | tags=3%, list=2%, signal=3% |
| 99 | KEGG\_GLYCOSAMINOGLYCAN\_BIOSYNTHESIS\_CHONDROITIN\_SULFATE |  | 22 | 0.40 | 0.83 | 0.681 | 0.794 | 1.000 | 1325 | tags=14%, list=6%, signal=15% |
| 100 | KEGG\_PHENYLALANINE\_METABOLISM |  | 17 | 0.38 | 0.82 | 0.727 | 0.805 | 1.000 | 89 | tags=6%, list=0%, signal=6% |
| 101 | KEGG\_HISTIDINE\_METABOLISM |  | 28 | 0.33 | 0.80 | 0.749 | 0.834 | 1.000 | 3987 | tags=25%, list=18%, signal=31% |
| 102 | KEGG\_DRUG\_METABOLISM\_CYTOCHROME\_P450 |  | 59 | 0.39 | 0.79 | 0.727 | 0.831 | 1.000 | 4155 | tags=25%, list=19%, signal=31% |
| 103 | KEGG\_GLYCINE\_SERINE\_AND\_THREONINE\_METABOLISM |  | 31 | 0.33 | 0.77 | 0.793 | 0.858 | 1.000 | 440 | tags=6%, list=2%, signal=7% |
| 104 | KEGG\_AMINO\_SUGAR\_AND\_NUCLEOTIDE\_SUGAR\_METABOLISM |  | 42 | 0.25 | 0.77 | 0.817 | 0.850 | 1.000 | 4951 | tags=31%, list=23%, signal=40% |
| 105 | KEGG\_ARGININE\_AND\_PROLINE\_METABOLISM |  | 49 | 0.26 | 0.73 | 0.956 | 0.899 | 1.000 | 1697 | tags=10%, list=8%, signal=11% |
| 106 | KEGG\_PYRUVATE\_METABOLISM |  | 38 | 0.23 | 0.69 | 0.892 | 0.932 | 1.000 | 4043 | tags=18%, list=19%, signal=23% |
| 107 | KEGG\_SELENOAMINO\_ACID\_METABOLISM |  | 25 | 0.24 | 0.66 | 0.917 | 0.957 | 1.000 | 2774 | tags=16%, list=13%, signal=18% |
| 108 | KEGG\_O\_GLYCAN\_BIOSYNTHESIS |  | 26 | 0.29 | 0.64 | 0.908 | 0.959 | 1.000 | 5312 | tags=46%, list=24%, signal=61% |
| 109 | KEGG\_PEROXISOME |  | 77 | 0.18 | 0.60 | 0.992 | 0.974 | 1.000 | 5385 | tags=29%, list=25%, signal=38% |
| 110 | KEGG\_RIBOSOME |  | 71 | 0.11 | 0.45 | 0.981 | 0.997 | 1.000 | 17715 | tags=99%, list=81%, signal=529% |
Table: Gene sets enriched in phenotype **H (36 samples)**[plain text format]****

  
