# Supplementary material for: Identification and validation of key modules and hub genes associated with the pathological stage of oral squamous cell carcinoma by weighted gene co-expression network analysis
Source: PeerJ. 2020 Feb 4;8:e8505. doi: 10.7717/peerj.8505 (PMC7006519; doi:10.7717/peerj.8505)
Supplement: File S6 [file peerj-08-8505-s006.zip › my_analysis_231773_kegg.Gsea.1570107186948/gsea_report_for_L_1570107186948.html]

Report for L 1570107186948 [GSEA]

| GS  follow link to MSigDB | GS DETAILS | SIZE | ES | NES | NOM p-val | FDR q-val | FWER p-val | RANK AT MAX | LEADING EDGE || 1 | KEGG\_SPLICEOSOME | Details ... | 114 | -0.56 | -1.89 | 0.006 | 0.035 | 0.038 | 6114 | tags=58%, list=28%, signal=80% |
| 2 | KEGG\_BASAL\_TRANSCRIPTION\_FACTORS | Details ... | 32 | -0.60 | -1.80 | 0.008 | 0.043 | 0.093 | 5480 | tags=56%, list=25%, signal=75% |
| 3 | KEGG\_PYRIMIDINE\_METABOLISM | Details ... | 91 | -0.53 | -1.68 | 0.008 | 0.118 | 0.293 | 4710 | tags=56%, list=22%, signal=71% |
| 4 | KEGG\_BASE\_EXCISION\_REPAIR | Details ... | 32 | -0.64 | -1.64 | 0.020 | 0.143 | 0.417 | 5498 | tags=72%, list=25%, signal=96% |
| 5 | KEGG\_MISMATCH\_REPAIR | Details ... | 22 | -0.70 | -1.63 | 0.032 | 0.133 | 0.457 | 1938 | tags=50%, list=9%, signal=55% |
| 6 | KEGG\_RNA\_POLYMERASE | Details ... | 28 | -0.55 | -1.59 | 0.044 | 0.156 | 0.556 | 5463 | tags=61%, list=25%, signal=81% |
| 7 | KEGG\_CELL\_CYCLE | Details ... | 122 | -0.59 | -1.59 | 0.037 | 0.139 | 0.564 | 4178 | tags=57%, list=19%, signal=71% |
| 8 | KEGG\_DNA\_REPLICATION | Details ... | 36 | -0.73 | -1.58 | 0.030 | 0.133 | 0.593 | 3475 | tags=72%, list=16%, signal=86% |
| 9 | KEGG\_HOMOLOGOUS\_RECOMBINATION | Details ... | 28 | -0.67 | -1.57 | 0.024 | 0.134 | 0.627 | 3477 | tags=54%, list=16%, signal=64% |
| 10 | KEGG\_UBIQUITIN\_MEDIATED\_PROTEOLYSIS | Details ... | 129 | -0.41 | -1.56 | 0.023 | 0.137 | 0.660 | 5949 | tags=43%, list=27%, signal=58% |
| 11 | KEGG\_NUCLEOTIDE\_EXCISION\_REPAIR | Details ... | 43 | -0.57 | -1.53 | 0.062 | 0.156 | 0.724 | 5498 | tags=65%, list=25%, signal=87% |
| 12 | KEGG\_PROTEASOME | Details ... | 41 | -0.58 | -1.51 | 0.106 | 0.165 | 0.760 | 6464 | tags=71%, list=30%, signal=100% |
| 13 | KEGG\_AMINOACYL\_TRNA\_BIOSYNTHESIS | Details ... | 37 | -0.55 | -1.50 | 0.083 | 0.177 | 0.793 | 5346 | tags=49%, list=25%, signal=64% |
| 14 | KEGG\_RNA\_DEGRADATION | Details ... | 56 | -0.46 | -1.46 | 0.078 | 0.220 | 0.874 | 4610 | tags=48%, list=21%, signal=61% |
| 15 | KEGG\_GLYCOSYLPHOSPHATIDYLINOSITOL\_GPI\_ANCHOR\_BIOSYNTHESIS | Details ... | 24 | -0.54 | -1.45 | 0.085 | 0.217 | 0.882 | 6058 | tags=46%, list=28%, signal=63% |
| 16 | KEGG\_STEROID\_BIOSYNTHESIS | Details ... | 15 | -0.67 | -1.45 | 0.060 | 0.207 | 0.885 | 2858 | tags=40%, list=13%, signal=46% |
| 17 | KEGG\_BLADDER\_CANCER | Details ... | 40 | -0.53 | -1.41 | 0.051 | 0.254 | 0.933 | 4598 | tags=50%, list=21%, signal=63% |
| 18 | KEGG\_DRUG\_METABOLISM\_OTHER\_ENZYMES | Details ... | 38 | -0.52 | -1.41 | 0.064 | 0.244 | 0.933 | 2247 | tags=29%, list=10%, signal=32% |
| 19 | KEGG\_OOCYTE\_MEIOSIS | Details ... | 107 | -0.38 | -1.39 | 0.081 | 0.251 | 0.941 | 2114 | tags=23%, list=10%, signal=26% |
| 20 | KEGG\_P53\_SIGNALING\_PATHWAY | Details ... | 65 | -0.43 | -1.23 | 0.145 | 0.588 | 0.996 | 2227 | tags=34%, list=10%, signal=38% |
| 21 | KEGG\_PENTOSE\_PHOSPHATE\_PATHWAY |  | 26 | -0.46 | -1.21 | 0.224 | 0.627 | 0.996 | 3688 | tags=42%, list=17%, signal=51% |
| 22 | KEGG\_GLYOXYLATE\_AND\_DICARBOXYLATE\_METABOLISM |  | 16 | -0.43 | -1.20 | 0.263 | 0.610 | 0.997 | 4558 | tags=31%, list=21%, signal=40% |
| 23 | KEGG\_ERBB\_SIGNALING\_PATHWAY |  | 86 | -0.34 | -1.20 | 0.119 | 0.597 | 0.997 | 4007 | tags=35%, list=18%, signal=43% |
| 24 | KEGG\_THYROID\_CANCER |  | 29 | -0.42 | -1.18 | 0.232 | 0.616 | 0.998 | 5844 | tags=48%, list=27%, signal=66% |
| 25 | KEGG\_RENAL\_CELL\_CARCINOMA |  | 68 | -0.37 | -1.15 | 0.228 | 0.660 | 0.999 | 3920 | tags=34%, list=18%, signal=41% |
| 26 | KEGG\_TERPENOID\_BACKBONE\_BIOSYNTHESIS |  | 15 | -0.52 | -1.13 | 0.341 | 0.696 | 1.000 | 7247 | tags=73%, list=33%, signal=110% |
| 27 | KEGG\_FRUCTOSE\_AND\_MANNOSE\_METABOLISM |  | 33 | -0.38 | -1.11 | 0.292 | 0.721 | 1.000 | 4348 | tags=36%, list=20%, signal=45% |
| 28 | KEGG\_PANCREATIC\_CANCER |  | 69 | -0.36 | -1.11 | 0.274 | 0.712 | 1.000 | 5377 | tags=51%, list=25%, signal=67% |
| 29 | KEGG\_GLYCOSPHINGOLIPID\_BIOSYNTHESIS\_LACTO\_AND\_NEOLACTO\_SERIES |  | 25 | -0.49 | -1.10 | 0.335 | 0.701 | 1.000 | 6146 | tags=56%, list=28%, signal=78% |
| 30 | KEGG\_DORSO\_VENTRAL\_AXIS\_FORMATION |  | 23 | -0.40 | -1.10 | 0.310 | 0.683 | 1.000 | 3796 | tags=35%, list=17%, signal=42% |
| 31 | KEGG\_PORPHYRIN\_AND\_CHLOROPHYLL\_METABOLISM |  | 30 | -0.42 | -1.09 | 0.360 | 0.680 | 1.000 | 6081 | tags=47%, list=28%, signal=65% |
| 32 | KEGG\_HEDGEHOG\_SIGNALING\_PATHWAY |  | 53 | -0.41 | -1.07 | 0.343 | 0.709 | 1.000 | 2874 | tags=26%, list=13%, signal=30% |
| 33 | KEGG\_AMYOTROPHIC\_LATERAL\_SCLEROSIS\_ALS |  | 51 | -0.32 | -1.05 | 0.359 | 0.742 | 1.000 | 5254 | tags=31%, list=24%, signal=41% |
| 34 | KEGG\_PROGESTERONE\_MEDIATED\_OOCYTE\_MATURATION |  | 83 | -0.32 | -1.04 | 0.359 | 0.741 | 1.000 | 1702 | tags=22%, list=8%, signal=23% |
| 35 | KEGG\_PURINE\_METABOLISM |  | 148 | -0.28 | -1.01 | 0.416 | 0.790 | 1.000 | 4710 | tags=42%, list=22%, signal=53% |
| 36 | KEGG\_BIOSYNTHESIS\_OF\_UNSATURATED\_FATTY\_ACIDS |  | 18 | -0.41 | -1.00 | 0.461 | 0.805 | 1.000 | 478 | tags=11%, list=2%, signal=11% |
| 37 | KEGG\_PATHOGENIC\_ESCHERICHIA\_COLI\_INFECTION |  | 53 | -0.29 | -0.99 | 0.460 | 0.815 | 1.000 | 3873 | tags=26%, list=18%, signal=32% |
| 38 | KEGG\_CHRONIC\_MYELOID\_LEUKEMIA |  | 72 | -0.29 | -0.98 | 0.481 | 0.813 | 1.000 | 4129 | tags=42%, list=19%, signal=51% |
| 39 | KEGG\_EPITHELIAL\_CELL\_SIGNALING\_IN\_HELICOBACTER\_PYLORI\_INFECTION |  | 66 | -0.31 | -0.97 | 0.522 | 0.824 | 1.000 | 3886 | tags=32%, list=18%, signal=39% |
| 40 | KEGG\_PATHWAYS\_IN\_CANCER |  | 319 | -0.30 | -0.96 | 0.478 | 0.807 | 1.000 | 2233 | tags=22%, list=10%, signal=24% |
| 41 | KEGG\_ONE\_CARBON\_POOL\_BY\_FOLATE |  | 16 | -0.37 | -0.96 | 0.519 | 0.794 | 1.000 | 4558 | tags=50%, list=21%, signal=63% |
| 42 | KEGG\_SMALL\_CELL\_LUNG\_CANCER |  | 84 | -0.34 | -0.96 | 0.465 | 0.784 | 1.000 | 1383 | tags=20%, list=6%, signal=22% |
| 43 | KEGG\_CITRATE\_CYCLE\_TCA\_CYCLE |  | 30 | -0.31 | -0.94 | 0.515 | 0.802 | 1.000 | 5202 | tags=30%, list=24%, signal=39% |
| 44 | KEGG\_CYSTEINE\_AND\_METHIONINE\_METABOLISM |  | 34 | -0.32 | -0.94 | 0.545 | 0.789 | 1.000 | 1213 | tags=15%, list=6%, signal=16% |
| 45 | KEGG\_BUTANOATE\_METABOLISM |  | 33 | -0.35 | -0.92 | 0.577 | 0.808 | 1.000 | 2340 | tags=18%, list=11%, signal=20% |
| 46 | KEGG\_SNARE\_INTERACTIONS\_IN\_VESICULAR\_TRANSPORT |  | 38 | -0.27 | -0.90 | 0.583 | 0.837 | 1.000 | 4359 | tags=32%, list=20%, signal=39% |
| 47 | KEGG\_PROTEIN\_EXPORT |  | 22 | -0.30 | -0.89 | 0.574 | 0.838 | 1.000 | 7921 | tags=41%, list=36%, signal=64% |
| 48 | KEGG\_LYSINE\_DEGRADATION |  | 41 | -0.29 | -0.89 | 0.660 | 0.823 | 1.000 | 2340 | tags=17%, list=11%, signal=19% |
| 49 | KEGG\_SPHINGOLIPID\_METABOLISM |  | 32 | -0.32 | -0.89 | 0.680 | 0.822 | 1.000 | 4967 | tags=31%, list=23%, signal=40% |
| 50 | KEGG\_ALANINE\_ASPARTATE\_AND\_GLUTAMATE\_METABOLISM |  | 32 | -0.32 | -0.86 | 0.721 | 0.862 | 1.000 | 5371 | tags=47%, list=25%, signal=62% |
| 51 | KEGG\_RIG\_I\_LIKE\_RECEPTOR\_SIGNALING\_PATHWAY |  | 69 | -0.28 | -0.83 | 0.673 | 0.903 | 1.000 | 6428 | tags=35%, list=30%, signal=49% |
| 52 | KEGG\_GLYCOSAMINOGLYCAN\_BIOSYNTHESIS\_HEPARAN\_SULFATE |  | 26 | -0.33 | -0.80 | 0.731 | 0.956 | 1.000 | 2279 | tags=19%, list=10%, signal=21% |
| 53 | KEGG\_BASAL\_CELL\_CARCINOMA |  | 52 | -0.34 | -0.79 | 0.749 | 0.961 | 1.000 | 2344 | tags=25%, list=11%, signal=28% |
| 54 | KEGG\_ASCORBATE\_AND\_ALDARATE\_METABOLISM |  | 15 | -0.38 | -0.77 | 0.737 | 0.966 | 1.000 | 1929 | tags=20%, list=9%, signal=22% |
| 55 | KEGG\_NOD\_LIKE\_RECEPTOR\_SIGNALING\_PATHWAY |  | 59 | -0.31 | -0.77 | 0.721 | 0.959 | 1.000 | 4088 | tags=29%, list=19%, signal=35% |
| 56 | KEGG\_STEROID\_HORMONE\_BIOSYNTHESIS |  | 42 | -0.32 | -0.74 | 0.874 | 0.989 | 1.000 | 2136 | tags=24%, list=10%, signal=26% |
| 57 | KEGG\_HUNTINGTONS\_DISEASE |  | 168 | -0.17 | -0.72 | 0.884 | 0.992 | 1.000 | 5233 | tags=27%, list=24%, signal=36% |
| 58 | KEGG\_GALACTOSE\_METABOLISM |  | 25 | -0.25 | -0.72 | 0.898 | 0.982 | 1.000 | 5447 | tags=36%, list=25%, signal=48% |
| 59 | KEGG\_GLYCEROLIPID\_METABOLISM |  | 42 | -0.25 | -0.72 | 0.960 | 0.966 | 1.000 | 2559 | tags=21%, list=12%, signal=24% |
| 60 | KEGG\_PENTOSE\_AND\_GLUCURONATE\_INTERCONVERSIONS |  | 17 | -0.31 | -0.67 | 0.858 | 0.996 | 1.000 | 4920 | tags=35%, list=23%, signal=46% |
| 61 | KEGG\_GLUTATHIONE\_METABOLISM |  | 47 | -0.28 | -0.66 | 0.949 | 0.993 | 1.000 | 1635 | tags=21%, list=8%, signal=23% |
| 62 | KEGG\_CYTOSOLIC\_DNA\_SENSING\_PATHWAY |  | 53 | -0.25 | -0.63 | 0.910 | 0.998 | 1.000 | 4532 | tags=26%, list=21%, signal=33% |
| 63 | KEGG\_METABOLISM\_OF\_XENOBIOTICS\_BY\_CYTOCHROME\_P450 |  | 56 | -0.26 | -0.52 | 0.990 | 1.000 | 1.000 | 2189 | tags=18%, list=10%, signal=20% |
| 64 | KEGG\_OLFACTORY\_TRANSDUCTION |  | 111 | -0.11 | -0.50 | 0.983 | 1.000 | 1.000 | 12734 | tags=77%, list=59%, signal=186% |
| 65 | KEGG\_RETINOL\_METABOLISM |  | 49 | -0.21 | -0.45 | 0.994 | 0.997 | 1.000 | 5388 | tags=31%, list=25%, signal=41% |
Table: Gene sets enriched in phenotype **L (61 samples)**[plain text format]****

  
