# Supplementary material for: Identification and validation of key modules and hub genes associated with the pathological stage of oral squamous cell carcinoma by weighted gene co-expression network analysis
Source: PeerJ. 2020 Feb 4;8:e8505. doi: 10.7717/peerj.8505 (PMC7006519; doi:10.7717/peerj.8505)
Supplement: File S6 [file peerj-08-8505-s006.zip › my_analysis_23177_BP.Gsea.1570106638719/gsea_report_for_H_1570106638719.html]

Report for H 1570106638719 [GSEA]

| GS  follow link to MSigDB | GS DETAILS | SIZE | ES | NES | NOM p-val | FDR q-val | FWER p-val | RANK AT MAX | LEADING EDGE || 1 | GO\_AXON\_EXTENSION | Details ... | 36 | 0.74 | 1.82 | 0.000 | 1.000 | 0.448 | 895 | tags=22%, list=4%, signal=23% |
| 2 | GO\_NEGATIVE\_REGULATION\_OF\_CYCLIC\_NUCLEOTIDE\_METABOLIC\_PROCESS | Details ... | 41 | 0.68 | 1.79 | 0.000 | 1.000 | 0.596 | 1568 | tags=22%, list=7%, signal=24% |
| 3 | GO\_PHOSPHATIDYLINOSITOL\_3\_KINASE\_SIGNALING | Details ... | 23 | 0.71 | 1.77 | 0.000 | 1.000 | 0.667 | 2478 | tags=39%, list=11%, signal=44% |
| 4 | GO\_NEGATIVE\_REGULATION\_OF\_CYTOPLASMIC\_TRANSPORT | Details ... | 109 | 0.56 | 1.75 | 0.000 | 1.000 | 0.725 | 2133 | tags=22%, list=10%, signal=24% |
| 5 | GO\_REGULATION\_OF\_LONG\_TERM\_NEURONAL\_SYNAPTIC\_PLASTICITY | Details ... | 23 | 0.65 | 1.74 | 0.004 | 0.988 | 0.779 | 1559 | tags=17%, list=7%, signal=19% |
| 6 | GO\_NEGATIVE\_REGULATION\_OF\_INTRACELLULAR\_TRANSPORT | Details ... | 131 | 0.56 | 1.73 | 0.000 | 0.927 | 0.809 | 3814 | tags=34%, list=18%, signal=41% |
| 7 | GO\_NEGATIVE\_REGULATION\_OF\_NUCLEOCYTOPLASMIC\_TRANSPORT | Details ... | 67 | 0.56 | 1.73 | 0.000 | 0.805 | 0.815 | 3241 | tags=27%, list=15%, signal=31% |
| 8 | GO\_NEGATIVE\_REGULATION\_OF\_PROTEIN\_POLYMERIZATION | Details ... | 54 | 0.60 | 1.70 | 0.002 | 0.962 | 0.880 | 2464 | tags=30%, list=11%, signal=33% |
| 9 | GO\_NEURON\_PROJECTION\_EXTENSION | Details ... | 51 | 0.64 | 1.70 | 0.006 | 0.861 | 0.880 | 895 | tags=18%, list=4%, signal=18% |
| 10 | GO\_MIDDLE\_EAR\_MORPHOGENESIS | Details ... | 20 | 0.75 | 1.70 | 0.000 | 0.777 | 0.880 | 2732 | tags=45%, list=13%, signal=51% |
| 11 | GO\_REGULATION\_OF\_CARDIAC\_CONDUCTION | Details ... | 64 | 0.70 | 1.70 | 0.002 | 0.717 | 0.884 | 1530 | tags=30%, list=7%, signal=32% |
| 12 | GO\_NEGATIVE\_REGULATION\_OF\_BMP\_SIGNALING\_PATHWAY | Details ... | 40 | 0.69 | 1.70 | 0.000 | 0.665 | 0.888 | 3304 | tags=40%, list=15%, signal=47% |
| 13 | GO\_NEGATIVE\_REGULATION\_OF\_LYASE\_ACTIVITY | Details ... | 26 | 0.68 | 1.70 | 0.004 | 0.619 | 0.890 | 1568 | tags=23%, list=7%, signal=25% |
| 14 | GO\_REGULATION\_OF\_ION\_HOMEOSTASIS | Details ... | 190 | 0.60 | 1.70 | 0.000 | 0.580 | 0.890 | 3039 | tags=35%, list=14%, signal=41% |
| 15 | GO\_NEGATIVE\_REGULATION\_OF\_INTRACELLULAR\_PROTEIN\_TRANSPORT | Details ... | 89 | 0.54 | 1.70 | 0.000 | 0.546 | 0.890 | 1966 | tags=20%, list=9%, signal=22% |
| 16 | GO\_REGULATION\_OF\_ADENYLATE\_CYCLASE\_ACTIVITY | Details ... | 64 | 0.57 | 1.69 | 0.000 | 0.542 | 0.904 | 3691 | tags=31%, list=17%, signal=38% |
| 17 | GO\_NEGATIVE\_REGULATION\_OF\_ACTIN\_FILAMENT\_POLYMERIZATION | Details ... | 43 | 0.66 | 1.69 | 0.004 | 0.519 | 0.907 | 2464 | tags=35%, list=11%, signal=39% |
| 18 | GO\_SECOND\_MESSENGER\_MEDIATED\_SIGNALING | Details ... | 150 | 0.61 | 1.69 | 0.000 | 0.502 | 0.907 | 3016 | tags=39%, list=14%, signal=45% |
| 19 | GO\_CALCIUM\_ION\_TRANSPORT | Details ... | 197 | 0.56 | 1.69 | 0.000 | 0.486 | 0.908 | 3489 | tags=33%, list=16%, signal=39% |
| 20 | GO\_LAMELLIPODIUM\_ASSEMBLY | Details ... | 28 | 0.64 | 1.69 | 0.006 | 0.463 | 0.908 | 1371 | tags=25%, list=6%, signal=27% |
| 21 | GO\_NEGATIVE\_REGULATION\_OF\_CELLULAR\_PROTEIN\_LOCALIZATION |  | 127 | 0.52 | 1.68 | 0.000 | 0.453 | 0.912 | 3241 | tags=27%, list=15%, signal=31% |
| 22 | GO\_CGMP\_METABOLIC\_PROCESS |  | 23 | 0.73 | 1.68 | 0.006 | 0.452 | 0.915 | 1962 | tags=39%, list=9%, signal=43% |
| 23 | GO\_NEGATIVE\_REGULATION\_OF\_MYOTUBE\_DIFFERENTIATION |  | 17 | 0.77 | 1.68 | 0.004 | 0.436 | 0.916 | 3290 | tags=71%, list=15%, signal=83% |
| 24 | GO\_REGULATION\_OF\_TRANSPORTER\_ACTIVITY |  | 188 | 0.55 | 1.68 | 0.000 | 0.421 | 0.917 | 2956 | tags=25%, list=14%, signal=29% |
| 25 | GO\_NEGATIVE\_REGULATION\_OF\_NUCLEOTIDE\_METABOLIC\_PROCESS |  | 61 | 0.60 | 1.68 | 0.004 | 0.406 | 0.917 | 3035 | tags=28%, list=14%, signal=32% |
| 26 | GO\_ANATOMICAL\_STRUCTURE\_MATURATION |  | 38 | 0.66 | 1.67 | 0.002 | 0.408 | 0.921 | 2058 | tags=37%, list=9%, signal=41% |
| 27 | GO\_REGULATION\_OF\_CALCIUM\_ION\_TRANSMEMBRANE\_TRANSPORT |  | 109 | 0.65 | 1.67 | 0.002 | 0.401 | 0.922 | 2956 | tags=39%, list=14%, signal=44% |
| 28 | GO\_REGULATION\_OF\_MYOTUBE\_DIFFERENTIATION |  | 54 | 0.67 | 1.67 | 0.002 | 0.397 | 0.926 | 3290 | tags=48%, list=15%, signal=57% |
| 29 | GO\_CALCIUM\_ION\_TRANSMEMBRANE\_TRANSPORT |  | 141 | 0.53 | 1.67 | 0.002 | 0.394 | 0.928 | 3489 | tags=30%, list=16%, signal=36% |
| 30 | GO\_POSITIVE\_REGULATION\_OF\_GLUCOSE\_METABOLIC\_PROCESS |  | 32 | 0.63 | 1.67 | 0.010 | 0.396 | 0.932 | 2999 | tags=34%, list=14%, signal=40% |
| 31 | GO\_ADRENERGIC\_RECEPTOR\_SIGNALING\_PATHWAY |  | 18 | 0.68 | 1.66 | 0.012 | 0.410 | 0.940 | 19 | tags=11%, list=0%, signal=11% |
| 32 | GO\_CIRCULATORY\_SYSTEM\_PROCESS |  | 352 | 0.57 | 1.65 | 0.000 | 0.420 | 0.947 | 2242 | tags=29%, list=10%, signal=32% |
| 33 | GO\_REGULATION\_OF\_CALCIUM\_ION\_TRANSMEMBRANE\_TRANSPORTER\_ACTIVITY |  | 66 | 0.68 | 1.65 | 0.004 | 0.409 | 0.947 | 2956 | tags=36%, list=14%, signal=42% |
| 34 | GO\_REGULATION\_OF\_CATION\_TRANSMEMBRANE\_TRANSPORT |  | 199 | 0.56 | 1.65 | 0.000 | 0.405 | 0.950 | 3825 | tags=35%, list=18%, signal=42% |
| 35 | GO\_POSITIVE\_REGULATION\_OF\_CATION\_TRANSMEMBRANE\_TRANSPORT |  | 90 | 0.56 | 1.65 | 0.002 | 0.412 | 0.960 | 4129 | tags=42%, list=19%, signal=52% |
| 36 | GO\_NEGATIVE\_REGULATION\_OF\_ACTIN\_FILAMENT\_DEPOLYMERIZATION |  | 34 | 0.68 | 1.65 | 0.008 | 0.413 | 0.962 | 2284 | tags=35%, list=10%, signal=39% |
| 37 | GO\_REGULATION\_OF\_NOREPINEPHRINE\_SECRETION |  | 16 | 0.75 | 1.64 | 0.004 | 0.419 | 0.966 | 1410 | tags=25%, list=6%, signal=27% |
| 38 | GO\_N\_GLYCAN\_PROCESSING |  | 19 | 0.73 | 1.64 | 0.012 | 0.418 | 0.968 | 2230 | tags=37%, list=10%, signal=41% |
| 39 | GO\_CYCLIC\_NUCLEOTIDE\_MEDIATED\_SIGNALING |  | 45 | 0.61 | 1.64 | 0.006 | 0.413 | 0.969 | 2670 | tags=29%, list=12%, signal=33% |
| 40 | GO\_NEGATIVE\_REGULATION\_OF\_CATION\_TRANSMEMBRANE\_TRANSPORT |  | 57 | 0.63 | 1.64 | 0.004 | 0.408 | 0.971 | 1410 | tags=21%, list=6%, signal=22% |
| 41 | GO\_NEGATIVE\_REGULATION\_OF\_TRANSMEMBRANE\_TRANSPORT |  | 79 | 0.57 | 1.64 | 0.004 | 0.399 | 0.971 | 2133 | tags=22%, list=10%, signal=24% |
| 42 | GO\_RECEPTOR\_INTERNALIZATION |  | 45 | 0.62 | 1.64 | 0.002 | 0.392 | 0.971 | 3131 | tags=36%, list=14%, signal=41% |
| 43 | GO\_REGULATION\_OF\_RELEASE\_OF\_SEQUESTERED\_CALCIUM\_ION\_INTO\_CYTOSOL |  | 70 | 0.67 | 1.64 | 0.002 | 0.389 | 0.975 | 3800 | tags=50%, list=17%, signal=60% |
| 44 | GO\_REGULATION\_OF\_GLYCOGEN\_METABOLIC\_PROCESS |  | 32 | 0.61 | 1.64 | 0.006 | 0.382 | 0.975 | 1639 | tags=22%, list=8%, signal=24% |
| 45 | GO\_POSITIVE\_REGULATION\_OF\_LIPASE\_ACTIVITY |  | 66 | 0.56 | 1.63 | 0.006 | 0.380 | 0.975 | 2943 | tags=35%, list=14%, signal=40% |
| 46 | GO\_REGULATION\_OF\_CALCIUM\_ION\_TRANSPORT\_INTO\_CYTOSOL |  | 87 | 0.64 | 1.63 | 0.000 | 0.379 | 0.977 | 3800 | tags=46%, list=17%, signal=55% |
| 47 | GO\_AORTA\_DEVELOPMENT |  | 41 | 0.67 | 1.63 | 0.018 | 0.372 | 0.977 | 2732 | tags=37%, list=13%, signal=42% |
| 48 | GO\_CELLULAR\_RESPONSE\_TO\_GLUCAGON\_STIMULUS |  | 38 | 0.57 | 1.63 | 0.012 | 0.366 | 0.977 | 2188 | tags=26%, list=10%, signal=29% |
| 49 | GO\_REGULATION\_OF\_CALCIUM\_MEDIATED\_SIGNALING |  | 72 | 0.65 | 1.63 | 0.000 | 0.360 | 0.978 | 2133 | tags=33%, list=10%, signal=37% |
| 50 | GO\_REGULATION\_OF\_SEQUESTERING\_OF\_CALCIUM\_ION |  | 98 | 0.64 | 1.63 | 0.002 | 0.356 | 0.980 | 3852 | tags=51%, list=18%, signal=62% |
| 51 | GO\_REGULATION\_OF\_CAMP\_METABOLIC\_PROCESS |  | 122 | 0.51 | 1.63 | 0.004 | 0.350 | 0.980 | 2841 | tags=23%, list=13%, signal=26% |
| 52 | GO\_POSITIVE\_REGULATION\_OF\_PHOSPHATIDYLINOSITOL\_3\_KINASE\_SIGNALING |  | 60 | 0.64 | 1.63 | 0.002 | 0.347 | 0.980 | 1855 | tags=38%, list=9%, signal=42% |
| 53 | GO\_REGULATION\_OF\_ANDROGEN\_RECEPTOR\_SIGNALING\_PATHWAY |  | 22 | 0.59 | 1.63 | 0.002 | 0.343 | 0.981 | 4152 | tags=45%, list=19%, signal=56% |
| 54 | GO\_RESPONSE\_TO\_PROSTAGLANDIN\_E |  | 25 | 0.77 | 1.63 | 0.004 | 0.346 | 0.983 | 2646 | tags=52%, list=12%, signal=59% |
| 55 | GO\_FIBRIL\_ORGANIZATION |  | 18 | 0.81 | 1.62 | 0.000 | 0.347 | 0.983 | 1957 | tags=50%, list=9%, signal=55% |
| 56 | GO\_REGULATION\_OF\_N\_METHYL\_D\_ASPARTATE\_SELECTIVE\_GLUTAMATE\_RECEPTOR\_ACTIVITY |  | 15 | 0.73 | 1.62 | 0.010 | 0.342 | 0.983 | 1367 | tags=27%, list=6%, signal=28% |
| 57 | GO\_AUTOPHAGOSOME\_ORGANIZATION |  | 40 | 0.52 | 1.62 | 0.015 | 0.337 | 0.983 | 4290 | tags=48%, list=20%, signal=59% |
| 58 | GO\_NEGATIVE\_REGULATION\_OF\_VASCULATURE\_DEVELOPMENT |  | 75 | 0.59 | 1.62 | 0.002 | 0.332 | 0.983 | 3080 | tags=39%, list=14%, signal=45% |
| 59 | GO\_REGULATION\_OF\_PHOSPHOLIPASE\_C\_ACTIVITY |  | 39 | 0.65 | 1.62 | 0.008 | 0.331 | 0.986 | 2943 | tags=51%, list=14%, signal=59% |
| 60 | GO\_DEVELOPMENTAL\_CELL\_GROWTH |  | 74 | 0.57 | 1.62 | 0.011 | 0.340 | 0.986 | 895 | tags=16%, list=4%, signal=17% |
| 61 | GO\_NEGATIVE\_REGULATION\_OF\_CATECHOLAMINE\_SECRETION |  | 16 | 0.74 | 1.62 | 0.004 | 0.336 | 0.986 | 1568 | tags=25%, list=7%, signal=27% |
| 62 | GO\_REGULATION\_OF\_RENAL\_SODIUM\_EXCRETION |  | 23 | 0.70 | 1.62 | 0.020 | 0.333 | 0.986 | 2646 | tags=30%, list=12%, signal=35% |
| 63 | GO\_POSITIVE\_REGULATION\_OF\_NUCLEOSIDE\_METABOLIC\_PROCESS |  | 23 | 0.66 | 1.62 | 0.024 | 0.330 | 0.987 | 3035 | tags=35%, list=14%, signal=40% |
| 64 | GO\_NEGATIVE\_REGULATION\_OF\_REACTIVE\_OXYGEN\_SPECIES\_BIOSYNTHETIC\_PROCESS |  | 15 | 0.70 | 1.62 | 0.013 | 0.327 | 0.988 | 2280 | tags=40%, list=10%, signal=45% |
| 65 | GO\_RENAL\_SYSTEM\_PROCESS |  | 98 | 0.53 | 1.62 | 0.008 | 0.323 | 0.988 | 3800 | tags=36%, list=17%, signal=43% |
| 66 | GO\_POSITIVE\_REGULATION\_OF\_TRANSPORTER\_ACTIVITY |  | 70 | 0.54 | 1.62 | 0.010 | 0.318 | 0.988 | 3920 | tags=40%, list=18%, signal=49% |
| 67 | GO\_REGULATION\_OF\_NUCLEOTIDE\_METABOLIC\_PROCESS |  | 199 | 0.48 | 1.61 | 0.002 | 0.318 | 0.988 | 3584 | tags=26%, list=16%, signal=31% |
| 68 | GO\_REGULATION\_OF\_TRANSMEMBRANE\_TRANSPORT |  | 402 | 0.48 | 1.61 | 0.002 | 0.318 | 0.989 | 3090 | tags=23%, list=14%, signal=26% |
| 69 | GO\_TRABECULA\_MORPHOGENESIS |  | 37 | 0.63 | 1.61 | 0.002 | 0.314 | 0.989 | 2125 | tags=41%, list=10%, signal=45% |
| 70 | GO\_DIVALENT\_INORGANIC\_CATION\_TRANSPORT |  | 241 | 0.50 | 1.61 | 0.000 | 0.314 | 0.991 | 3413 | tags=28%, list=16%, signal=33% |
| 71 | GO\_REGULATION\_OF\_MACROPHAGE\_DERIVED\_FOAM\_CELL\_DIFFERENTIATION |  | 27 | 0.69 | 1.61 | 0.006 | 0.313 | 0.992 | 1165 | tags=30%, list=5%, signal=31% |
| 72 | GO\_POSITIVE\_REGULATION\_OF\_LIPID\_STORAGE |  | 16 | 0.77 | 1.61 | 0.010 | 0.312 | 0.992 | 755 | tags=38%, list=3%, signal=39% |
| 73 | GO\_MELANOCYTE\_DIFFERENTIATION |  | 19 | 0.72 | 1.61 | 0.008 | 0.309 | 0.992 | 3404 | tags=58%, list=16%, signal=69% |
| 74 | GO\_POSITIVE\_REGULATION\_OF\_GLYCOGEN\_METABOLIC\_PROCESS |  | 15 | 0.75 | 1.61 | 0.008 | 0.308 | 0.993 | 2999 | tags=47%, list=14%, signal=54% |
| 75 | GO\_REGULATION\_OF\_BMP\_SIGNALING\_PATHWAY |  | 75 | 0.60 | 1.61 | 0.002 | 0.304 | 0.993 | 3304 | tags=37%, list=15%, signal=44% |
| 76 | GO\_POSITIVE\_REGULATION\_OF\_POTASSIUM\_ION\_TRANSPORT |  | 37 | 0.55 | 1.60 | 0.016 | 0.310 | 0.993 | 124 | tags=11%, list=1%, signal=11% |
| 77 | GO\_NEGATIVE\_REGULATION\_OF\_G\_PROTEIN\_COUPLED\_RECEPTOR\_PROTEIN\_SIGNALING\_PATHWAY |  | 37 | 0.63 | 1.60 | 0.010 | 0.307 | 0.993 | 3658 | tags=38%, list=17%, signal=45% |
| 78 | GO\_POSITIVE\_REGULATION\_OF\_CALCIUM\_ION\_TRANSMEMBRANE\_TRANSPORTER\_ACTIVITY |  | 29 | 0.64 | 1.60 | 0.020 | 0.304 | 0.993 | 3402 | tags=45%, list=16%, signal=53% |
| 79 | GO\_REGULATION\_OF\_PHOSPHOLIPID\_METABOLIC\_PROCESS |  | 58 | 0.57 | 1.60 | 0.004 | 0.302 | 0.993 | 2478 | tags=36%, list=11%, signal=41% |
| 80 | GO\_BONE\_CELL\_DEVELOPMENT |  | 22 | 0.59 | 1.60 | 0.020 | 0.299 | 0.993 | 4065 | tags=45%, list=19%, signal=56% |
| 81 | GO\_RESPONSE\_TO\_BMP |  | 89 | 0.58 | 1.60 | 0.000 | 0.300 | 0.993 | 3058 | tags=33%, list=14%, signal=38% |
| 82 | GO\_NEGATIVE\_REGULATION\_OF\_TRANSPORTER\_ACTIVITY |  | 60 | 0.60 | 1.60 | 0.010 | 0.296 | 0.993 | 1206 | tags=18%, list=6%, signal=19% |
| 83 | GO\_REGULATION\_OF\_GLUCOSE\_IMPORT\_IN\_RESPONSE\_TO\_INSULIN\_STIMULUS |  | 15 | 0.71 | 1.60 | 0.006 | 0.294 | 0.994 | 3755 | tags=60%, list=17%, signal=72% |
| 84 | GO\_ACTIN\_FILAMENT\_BASED\_PROCESS |  | 434 | 0.56 | 1.60 | 0.006 | 0.291 | 0.994 | 2512 | tags=29%, list=12%, signal=32% |
| 85 | GO\_REGULATION\_OF\_NUCLEOTIDE\_CATABOLIC\_PROCESS |  | 34 | 0.57 | 1.60 | 0.015 | 0.290 | 0.994 | 3035 | tags=29%, list=14%, signal=34% |
| 86 | GO\_REGULATION\_OF\_MUSCLE\_ADAPTATION |  | 62 | 0.65 | 1.60 | 0.012 | 0.287 | 0.994 | 1322 | tags=27%, list=6%, signal=29% |
| 87 | GO\_REGULATION\_OF\_CATION\_CHANNEL\_ACTIVITY |  | 83 | 0.58 | 1.60 | 0.004 | 0.287 | 0.994 | 2956 | tags=28%, list=14%, signal=32% |
| 88 | GO\_REGULATION\_OF\_METAL\_ION\_TRANSPORT |  | 308 | 0.50 | 1.60 | 0.000 | 0.287 | 0.994 | 3080 | tags=27%, list=14%, signal=31% |
| 89 | GO\_ASTROCYTE\_DEVELOPMENT |  | 19 | 0.68 | 1.60 | 0.013 | 0.287 | 0.995 | 1557 | tags=26%, list=7%, signal=28% |
| 90 | GO\_MYELOID\_CELL\_DEVELOPMENT |  | 42 | 0.53 | 1.60 | 0.004 | 0.284 | 0.995 | 4065 | tags=40%, list=19%, signal=50% |
| 91 | GO\_CELL\_MATRIX\_ADHESION |  | 107 | 0.60 | 1.60 | 0.004 | 0.282 | 0.995 | 1905 | tags=33%, list=9%, signal=36% |
| 92 | GO\_RESPONSE\_TO\_PROSTAGLANDIN |  | 33 | 0.72 | 1.60 | 0.004 | 0.281 | 0.995 | 2188 | tags=39%, list=10%, signal=44% |
| 93 | GO\_NEGATIVE\_REGULATION\_OF\_CALCIUM\_ION\_TRANSMEMBRANE\_TRANSPORT |  | 26 | 0.77 | 1.60 | 0.006 | 0.280 | 0.995 | 2133 | tags=42%, list=10%, signal=47% |
| 94 | GO\_REGULATION\_OF\_CALCIUM\_ION\_IMPORT |  | 94 | 0.62 | 1.59 | 0.006 | 0.278 | 0.995 | 3200 | tags=39%, list=15%, signal=46% |
| 95 | GO\_NEGATIVE\_REGULATION\_OF\_PROTEIN\_COMPLEX\_ASSEMBLY |  | 103 | 0.49 | 1.59 | 0.002 | 0.282 | 0.997 | 3026 | tags=27%, list=14%, signal=31% |
| 96 | GO\_REGULATION\_OF\_PHOSPHOLIPASE\_ACTIVITY |  | 63 | 0.56 | 1.59 | 0.012 | 0.281 | 0.997 | 2943 | tags=38%, list=14%, signal=44% |
| 97 | GO\_POSITIVE\_REGULATION\_OF\_TRANSMEMBRANE\_TRANSPORT |  | 120 | 0.51 | 1.59 | 0.002 | 0.280 | 0.997 | 3691 | tags=35%, list=17%, signal=42% |
| 98 | GO\_CALCIUM\_MEDIATED\_SIGNALING |  | 82 | 0.61 | 1.59 | 0.000 | 0.280 | 0.997 | 3001 | tags=44%, list=14%, signal=51% |
| 99 | GO\_RESPONSE\_TO\_GLUCAGON |  | 48 | 0.53 | 1.59 | 0.012 | 0.278 | 0.997 | 2188 | tags=23%, list=10%, signal=25% |
| 100 | GO\_MYOTUBE\_DIFFERENTIATION |  | 51 | 0.65 | 1.59 | 0.016 | 0.281 | 0.997 | 3035 | tags=35%, list=14%, signal=41% |
| 101 | GO\_REGULATION\_OF\_NON\_CANONICAL\_WNT\_SIGNALING\_PATHWAY |  | 19 | 0.76 | 1.59 | 0.012 | 0.278 | 0.997 | 1376 | tags=42%, list=6%, signal=45% |
| 102 | GO\_POSITIVE\_REGULATION\_OF\_PHOSPHOLIPASE\_ACTIVITY |  | 53 | 0.57 | 1.59 | 0.014 | 0.276 | 0.997 | 2943 | tags=40%, list=14%, signal=46% |
| 103 | GO\_MUSCLE\_STRUCTURE\_DEVELOPMENT |  | 410 | 0.61 | 1.59 | 0.006 | 0.275 | 0.997 | 3309 | tags=40%, list=15%, signal=46% |
| 104 | GO\_POSITIVE\_REGULATION\_OF\_NUCLEOTIDE\_CATABOLIC\_PROCESS |  | 16 | 0.68 | 1.59 | 0.016 | 0.273 | 0.997 | 2787 | tags=31%, list=13%, signal=36% |
| 105 | GO\_REGULATION\_OF\_BLOOD\_CIRCULATION |  | 285 | 0.55 | 1.59 | 0.008 | 0.274 | 0.997 | 2233 | tags=25%, list=10%, signal=28% |
| 106 | GO\_CYTOSOLIC\_CALCIUM\_ION\_TRANSPORT |  | 49 | 0.65 | 1.58 | 0.010 | 0.277 | 0.998 | 3377 | tags=49%, list=16%, signal=58% |
| 107 | GO\_NEGATIVE\_REGULATION\_OF\_TRANSPORT |  | 427 | 0.45 | 1.58 | 0.002 | 0.275 | 0.998 | 3273 | tags=24%, list=15%, signal=28% |
| 108 | GO\_NITRIC\_OXIDE\_MEDIATED\_SIGNAL\_TRANSDUCTION |  | 18 | 0.80 | 1.58 | 0.006 | 0.274 | 0.998 | 1925 | tags=50%, list=9%, signal=55% |
| 109 | GO\_HETEROPHILIC\_CELL\_CELL\_ADHESION\_VIA\_PLASMA\_MEMBRANE\_CELL\_ADHESION\_MOLECULES |  | 38 | 0.67 | 1.58 | 0.002 | 0.273 | 0.998 | 862 | tags=26%, list=4%, signal=27% |
| 110 | GO\_CELL\_RECOGNITION |  | 125 | 0.59 | 1.58 | 0.004 | 0.272 | 0.998 | 2932 | tags=34%, list=13%, signal=39% |
| 111 | GO\_REGULATION\_OF\_CARDIAC\_MUSCLE\_CONTRACTION\_BY\_REGULATION\_OF\_THE\_RELEASE\_OF\_SEQUESTERED\_CALCIUM\_ION |  | 18 | 0.81 | 1.58 | 0.004 | 0.271 | 0.998 | 1294 | tags=44%, list=6%, signal=47% |
| 112 | GO\_REGULATION\_OF\_CATECHOLAMINE\_SECRETION |  | 41 | 0.58 | 1.58 | 0.000 | 0.270 | 0.998 | 1568 | tags=15%, list=7%, signal=16% |
| 113 | GO\_COMPLEMENT\_ACTIVATION |  | 51 | 0.74 | 1.58 | 0.016 | 0.271 | 0.998 | 3374 | tags=61%, list=16%, signal=72% |
| 114 | GO\_PROTEIN\_ACTIVATION\_CASCADE |  | 73 | 0.68 | 1.58 | 0.014 | 0.269 | 0.998 | 3374 | tags=51%, list=16%, signal=60% |
| 115 | GO\_BONE\_DEVELOPMENT |  | 149 | 0.54 | 1.58 | 0.011 | 0.269 | 0.998 | 3066 | tags=34%, list=14%, signal=40% |
| 116 | GO\_NEGATIVE\_REGULATION\_OF\_CELLULAR\_RESPONSE\_TO\_GROWTH\_FACTOR\_STIMULUS |  | 116 | 0.58 | 1.58 | 0.008 | 0.267 | 0.998 | 3619 | tags=34%, list=17%, signal=41% |
| 117 | GO\_REGULATION\_OF\_CALCIUM\_ION\_TRANSPORT |  | 196 | 0.56 | 1.58 | 0.002 | 0.266 | 0.998 | 3200 | tags=34%, list=15%, signal=40% |
| 118 | GO\_REGULATION\_OF\_HEART\_CONTRACTION |  | 214 | 0.57 | 1.58 | 0.014 | 0.265 | 0.998 | 2233 | tags=25%, list=10%, signal=28% |
| 119 | GO\_DETECTION\_OF\_MECHANICAL\_STIMULUS |  | 37 | 0.65 | 1.58 | 0.027 | 0.267 | 0.998 | 1966 | tags=27%, list=9%, signal=30% |
| 120 | GO\_POSITIVE\_REGULATION\_OF\_SMALL\_GTPASE\_MEDIATED\_SIGNAL\_TRANSDUCTION |  | 38 | 0.55 | 1.58 | 0.008 | 0.266 | 0.998 | 2920 | tags=37%, list=13%, signal=42% |
| 121 | GO\_POSITIVE\_REGULATION\_OF\_CALCIUM\_ION\_TRANSMEMBRANE\_TRANSPORT |  | 54 | 0.60 | 1.57 | 0.004 | 0.267 | 0.998 | 4129 | tags=50%, list=19%, signal=62% |
| 122 | GO\_ERYTHROCYTE\_HOMEOSTASIS |  | 70 | 0.49 | 1.57 | 0.002 | 0.268 | 0.999 | 3407 | tags=29%, list=16%, signal=34% |
| 123 | GO\_DEVELOPMENTAL\_PIGMENTATION |  | 38 | 0.55 | 1.57 | 0.012 | 0.268 | 0.999 | 3404 | tags=34%, list=16%, signal=40% |
| 124 | GO\_NEGATIVE\_REGULATION\_OF\_LOCOMOTION |  | 249 | 0.50 | 1.57 | 0.002 | 0.267 | 0.999 | 3194 | tags=33%, list=15%, signal=39% |
| 125 | GO\_REGULATION\_OF\_LIPID\_KINASE\_ACTIVITY |  | 47 | 0.59 | 1.57 | 0.006 | 0.266 | 0.999 | 3284 | tags=40%, list=15%, signal=48% |
| 126 | GO\_REGULATION\_OF\_GLUCOSE\_METABOLIC\_PROCESS |  | 97 | 0.52 | 1.57 | 0.009 | 0.269 | 0.999 | 2999 | tags=28%, list=14%, signal=32% |
| 127 | GO\_REGULATION\_OF\_GASTRULATION |  | 31 | 0.65 | 1.57 | 0.019 | 0.267 | 0.999 | 2074 | tags=32%, list=10%, signal=36% |
| 128 | GO\_RESPONSE\_TO\_THYROID\_HORMONE |  | 19 | 0.65 | 1.57 | 0.010 | 0.266 | 0.999 | 2956 | tags=37%, list=14%, signal=43% |
| 129 | GO\_MUSCLE\_CELL\_DIFFERENTIATION |  | 227 | 0.62 | 1.57 | 0.014 | 0.264 | 0.999 | 3293 | tags=39%, list=15%, signal=46% |
| 130 | GO\_NEGATIVE\_REGULATION\_OF\_KIDNEY\_DEVELOPMENT |  | 17 | 0.66 | 1.57 | 0.004 | 0.263 | 0.999 | 3138 | tags=35%, list=14%, signal=41% |
| 131 | GO\_CYCLIC\_NUCLEOTIDE\_METABOLIC\_PROCESS |  | 55 | 0.58 | 1.57 | 0.006 | 0.261 | 0.999 | 1962 | tags=29%, list=9%, signal=32% |
| 132 | GO\_REGULATION\_OF\_CYTOSOLIC\_CALCIUM\_ION\_CONCENTRATION |  | 191 | 0.51 | 1.57 | 0.006 | 0.260 | 0.999 | 3016 | tags=29%, list=14%, signal=33% |
| 133 | GO\_PROTEIN\_LOCALIZATION\_TO\_CELL\_SURFACE |  | 22 | 0.62 | 1.57 | 0.012 | 0.259 | 0.999 | 938 | tags=23%, list=4%, signal=24% |
| 134 | GO\_ACTIVATION\_OF\_PROTEIN\_KINASE\_B\_ACTIVITY |  | 21 | 0.58 | 1.57 | 0.019 | 0.259 | 0.999 | 4187 | tags=38%, list=19%, signal=47% |
| 135 | GO\_DEFINITIVE\_HEMOPOIESIS |  | 15 | 0.70 | 1.57 | 0.008 | 0.260 | 0.999 | 3075 | tags=47%, list=14%, signal=54% |
| 136 | GO\_REGULATION\_OF\_SYSTEM\_PROCESS |  | 488 | 0.52 | 1.57 | 0.006 | 0.259 | 0.999 | 2290 | tags=24%, list=11%, signal=26% |
| 137 | GO\_REGULATION\_OF\_CARDIAC\_MUSCLE\_CONTRACTION\_BY\_CALCIUM\_ION\_SIGNALING |  | 22 | 0.75 | 1.57 | 0.008 | 0.258 | 0.999 | 1294 | tags=36%, list=6%, signal=39% |
| 138 | GO\_RENAL\_WATER\_HOMEOSTASIS |  | 34 | 0.59 | 1.56 | 0.026 | 0.257 | 0.999 | 3578 | tags=35%, list=16%, signal=42% |
| 139 | GO\_REGULATION\_OF\_LIPASE\_ACTIVITY |  | 82 | 0.53 | 1.56 | 0.014 | 0.257 | 0.999 | 2943 | tags=34%, list=14%, signal=39% |
| 140 | GO\_REGULATION\_OF\_LYASE\_ACTIVITY |  | 79 | 0.49 | 1.56 | 0.013 | 0.256 | 0.999 | 3691 | tags=25%, list=17%, signal=30% |
| 141 | GO\_TRABECULA\_FORMATION |  | 22 | 0.71 | 1.56 | 0.006 | 0.260 | 0.999 | 2125 | tags=50%, list=10%, signal=55% |
| 142 | GO\_NEGATIVE\_REGULATION\_OF\_ESTABLISHMENT\_OF\_PROTEIN\_LOCALIZATION |  | 197 | 0.46 | 1.56 | 0.002 | 0.259 | 0.999 | 3814 | tags=29%, list=18%, signal=35% |
| 143 | GO\_CALCIUM\_ION\_IMPORT |  | 58 | 0.58 | 1.56 | 0.018 | 0.259 | 0.999 | 3016 | tags=34%, list=14%, signal=40% |
| 144 | GO\_REGULATION\_OF\_CARBOHYDRATE\_CATABOLIC\_PROCESS |  | 40 | 0.56 | 1.56 | 0.020 | 0.257 | 0.999 | 1556 | tags=20%, list=7%, signal=22% |
| 145 | GO\_REGULATION\_OF\_PLATELET\_AGGREGATION |  | 16 | 0.71 | 1.56 | 0.014 | 0.257 | 0.999 | 3526 | tags=56%, list=16%, signal=67% |
| 146 | GO\_POSITIVE\_REGULATION\_OF\_NUCLEOTIDE\_METABOLIC\_PROCESS |  | 125 | 0.48 | 1.56 | 0.009 | 0.257 | 0.999 | 3691 | tags=29%, list=17%, signal=34% |
| 147 | GO\_REGULATION\_OF\_MUSCLE\_SYSTEM\_PROCESS |  | 190 | 0.58 | 1.56 | 0.010 | 0.256 | 0.999 | 1447 | tags=26%, list=7%, signal=27% |
| 148 | GO\_REGULATION\_OF\_HOMEOSTATIC\_PROCESS |  | 424 | 0.46 | 1.56 | 0.000 | 0.257 | 0.999 | 3049 | tags=26%, list=14%, signal=30% |
| 149 | GO\_DIVALENT\_INORGANIC\_CATION\_HOMEOSTASIS |  | 321 | 0.48 | 1.56 | 0.002 | 0.256 | 0.999 | 2944 | tags=26%, list=14%, signal=29% |
| 150 | GO\_NEGATIVE\_REGULATION\_OF\_EXOCYTOSIS |  | 26 | 0.65 | 1.56 | 0.012 | 0.255 | 0.999 | 3506 | tags=42%, list=16%, signal=50% |
| 151 | GO\_CELL\_SUBSTRATE\_ADHESION |  | 152 | 0.56 | 1.56 | 0.008 | 0.254 | 0.999 | 1905 | tags=30%, list=9%, signal=33% |
| 152 | GO\_MEMBRANE\_DEPOLARIZATION\_DURING\_ACTION\_POTENTIAL |  | 36 | 0.62 | 1.56 | 0.020 | 0.255 | 0.999 | 3039 | tags=33%, list=14%, signal=39% |
| 153 | GO\_REGULATION\_OF\_PHOSPHATIDYLINOSITOL\_3\_KINASE\_SIGNALING |  | 136 | 0.52 | 1.55 | 0.000 | 0.257 | 0.999 | 2461 | tags=29%, list=11%, signal=32% |
| 154 | GO\_EMBRYONIC\_DIGESTIVE\_TRACT\_MORPHOGENESIS |  | 17 | 0.70 | 1.55 | 0.015 | 0.256 | 0.999 | 1829 | tags=35%, list=8%, signal=39% |
| 155 | GO\_REGULATION\_OF\_PHOSPHATIDYLINOSITOL\_3\_KINASE\_ACTIVITY |  | 39 | 0.62 | 1.55 | 0.010 | 0.254 | 0.999 | 3284 | tags=44%, list=15%, signal=51% |
| 156 | GO\_ACTIVATION\_OF\_PROTEIN\_KINASE\_A\_ACTIVITY |  | 17 | 0.65 | 1.55 | 0.022 | 0.253 | 0.999 | 2014 | tags=29%, list=9%, signal=32% |
| 157 | GO\_POSITIVE\_REGULATION\_OF\_GLUCOSE\_TRANSPORT |  | 40 | 0.61 | 1.55 | 0.013 | 0.252 | 0.999 | 2791 | tags=38%, list=13%, signal=43% |
| 158 | GO\_NEGATIVE\_REGULATION\_OF\_ION\_TRANSPORT |  | 118 | 0.53 | 1.55 | 0.002 | 0.256 | 0.999 | 2166 | tags=25%, list=10%, signal=27% |
| 159 | GO\_FOREBRAIN\_NEURON\_DEVELOPMENT |  | 34 | 0.55 | 1.55 | 0.020 | 0.256 | 0.999 | 2898 | tags=26%, list=13%, signal=30% |
| 160 | GO\_HEART\_PROCESS |  | 81 | 0.73 | 1.55 | 0.018 | 0.257 | 0.999 | 1389 | tags=40%, list=6%, signal=42% |
| 161 | GO\_MUSCLE\_ORGAN\_DEVELOPMENT |  | 258 | 0.63 | 1.55 | 0.012 | 0.256 | 0.999 | 2478 | tags=36%, list=11%, signal=41% |
| 162 | GO\_REGULATION\_OF\_ACTIN\_FILAMENT\_BASED\_PROCESS |  | 300 | 0.48 | 1.55 | 0.002 | 0.256 | 0.999 | 3170 | tags=30%, list=15%, signal=35% |
| 163 | GO\_REGULATION\_OF\_PROTEIN\_ACTIVATION\_CASCADE |  | 31 | 0.66 | 1.55 | 0.030 | 0.257 | 0.999 | 3217 | tags=45%, list=15%, signal=53% |
| 164 | GO\_ENDOCYTOSIS |  | 453 | 0.47 | 1.55 | 0.002 | 0.261 | 0.999 | 3420 | tags=28%, list=16%, signal=33% |
| 165 | GO\_MUSCLE\_SYSTEM\_PROCESS |  | 275 | 0.68 | 1.54 | 0.014 | 0.267 | 0.999 | 1969 | tags=36%, list=9%, signal=39% |
| 166 | GO\_STRIATED\_MUSCLE\_CONTRACTION |  | 94 | 0.74 | 1.54 | 0.022 | 0.266 | 0.999 | 1389 | tags=40%, list=6%, signal=43% |
| 167 | GO\_ACTIVATION\_OF\_PHOSPHOLIPASE\_C\_ACTIVITY |  | 27 | 0.62 | 1.54 | 0.014 | 0.266 | 0.999 | 2943 | tags=48%, list=14%, signal=56% |
| 168 | GO\_STRIATED\_MUSCLE\_ADAPTATION |  | 23 | 0.75 | 1.54 | 0.026 | 0.266 | 0.999 | 3035 | tags=57%, list=14%, signal=66% |
| 169 | GO\_CARBOHYDRATE\_DERIVATIVE\_CATABOLIC\_PROCESS |  | 164 | 0.47 | 1.54 | 0.002 | 0.264 | 0.999 | 3890 | tags=32%, list=18%, signal=38% |
| 170 | GO\_POSITIVE\_REGULATION\_OF\_PHOSPHOLIPID\_METABOLIC\_PROCESS |  | 40 | 0.59 | 1.54 | 0.016 | 0.267 | 0.999 | 3170 | tags=43%, list=15%, signal=50% |
| 171 | GO\_NEUROMUSCULAR\_JUNCTION\_DEVELOPMENT |  | 35 | 0.60 | 1.54 | 0.022 | 0.266 | 0.999 | 2834 | tags=29%, list=13%, signal=33% |
| 172 | GO\_REGULATED\_EXOCYTOSIS |  | 207 | 0.50 | 1.54 | 0.000 | 0.266 | 0.999 | 3085 | tags=28%, list=14%, signal=32% |
| 173 | GO\_REGULATION\_OF\_RELEASE\_OF\_SEQUESTERED\_CALCIUM\_ION\_INTO\_CYTOSOL\_BY\_SARCOPLASMIC\_RETICULUM |  | 24 | 0.80 | 1.54 | 0.016 | 0.265 | 0.999 | 1294 | tags=42%, list=6%, signal=44% |
| 174 | GO\_NEGATIVE\_REGULATION\_OF\_STRIATED\_MUSCLE\_CELL\_DIFFERENTIATION |  | 25 | 0.67 | 1.54 | 0.021 | 0.265 | 0.999 | 3290 | tags=60%, list=15%, signal=71% |
| 175 | GO\_REGULATION\_OF\_MUSCLE\_CELL\_DIFFERENTIATION |  | 146 | 0.53 | 1.54 | 0.008 | 0.264 | 0.999 | 3347 | tags=34%, list=15%, signal=39% |
| 176 | GO\_REGULATION\_OF\_ENERGY\_HOMEOSTASIS |  | 17 | 0.66 | 1.54 | 0.010 | 0.265 | 0.999 | 2052 | tags=29%, list=9%, signal=32% |
| 177 | GO\_ACTIN\_POLYMERIZATION\_OR\_DEPOLYMERIZATION |  | 35 | 0.58 | 1.54 | 0.022 | 0.265 | 0.999 | 3709 | tags=49%, list=17%, signal=58% |
| 178 | GO\_PLATELET\_DEGRANULATION |  | 101 | 0.58 | 1.54 | 0.018 | 0.266 | 0.999 | 3526 | tags=39%, list=16%, signal=46% |
| 179 | GO\_NEGATIVE\_REGULATION\_OF\_TRANSMEMBRANE\_RECEPTOR\_PROTEIN\_SERINE\_THREONINE\_KINASE\_SIGNALING\_PATHWAY |  | 97 | 0.55 | 1.53 | 0.016 | 0.266 | 0.999 | 3459 | tags=30%, list=16%, signal=35% |
| 180 | GO\_NEGATIVE\_REGULATION\_OF\_INTRACELLULAR\_STEROID\_HORMONE\_RECEPTOR\_SIGNALING\_PATHWAY |  | 28 | 0.55 | 1.53 | 0.014 | 0.266 | 0.999 | 1583 | tags=25%, list=7%, signal=27% |
| 181 | GO\_GLIAL\_CELL\_DEVELOPMENT |  | 73 | 0.50 | 1.53 | 0.018 | 0.266 | 0.999 | 2040 | tags=22%, list=9%, signal=24% |
| 182 | GO\_SKELETAL\_MUSCLE\_ORGAN\_DEVELOPMENT |  | 127 | 0.60 | 1.53 | 0.016 | 0.267 | 0.999 | 3699 | tags=41%, list=17%, signal=49% |
| 183 | GO\_LAMELLIPODIUM\_ORGANIZATION |  | 34 | 0.56 | 1.53 | 0.020 | 0.266 | 0.999 | 1371 | tags=24%, list=6%, signal=25% |
| 184 | GO\_REGULATION\_OF\_INSULIN\_LIKE\_GROWTH\_FACTOR\_RECEPTOR\_SIGNALING\_PATHWAY |  | 22 | 0.70 | 1.53 | 0.036 | 0.266 | 0.999 | 672 | tags=23%, list=3%, signal=23% |
| 185 | GO\_REGULATION\_OF\_SODIUM\_ION\_TRANSMEMBRANE\_TRANSPORTER\_ACTIVITY |  | 37 | 0.62 | 1.53 | 0.031 | 0.266 | 0.999 | 1523 | tags=24%, list=7%, signal=26% |
| 186 | GO\_RESPONSE\_TO\_ZINC\_ION |  | 51 | 0.55 | 1.53 | 0.030 | 0.265 | 0.999 | 2675 | tags=22%, list=12%, signal=25% |
| 187 | GO\_REGULATION\_OF\_CYCLIC\_NUCLEOTIDE\_METABOLIC\_PROCESS |  | 147 | 0.48 | 1.53 | 0.019 | 0.264 | 0.999 | 3691 | tags=27%, list=17%, signal=33% |
| 188 | GO\_NEGATIVE\_REGULATION\_OF\_HOMEOSTATIC\_PROCESS |  | 117 | 0.49 | 1.53 | 0.014 | 0.263 | 0.999 | 3016 | tags=29%, list=14%, signal=34% |
| 189 | GO\_PIGMENT\_CELL\_DIFFERENTIATION |  | 27 | 0.58 | 1.53 | 0.020 | 0.262 | 0.999 | 3404 | tags=41%, list=16%, signal=48% |
| 190 | GO\_MUSCLE\_CELL\_DEVELOPMENT |  | 122 | 0.70 | 1.53 | 0.018 | 0.261 | 0.999 | 2213 | tags=40%, list=10%, signal=44% |
| 191 | GO\_REGULATION\_OF\_RYANODINE\_SENSITIVE\_CALCIUM\_RELEASE\_CHANNEL\_ACTIVITY |  | 25 | 0.73 | 1.53 | 0.016 | 0.264 | 0.999 | 2133 | tags=40%, list=10%, signal=44% |
| 192 | GO\_MUSCLE\_CONTRACTION |  | 226 | 0.71 | 1.53 | 0.023 | 0.264 | 0.999 | 1969 | tags=39%, list=9%, signal=42% |
| 193 | GO\_NEGATIVE\_REGULATION\_OF\_LEUKOCYTE\_MIGRATION |  | 29 | 0.68 | 1.52 | 0.006 | 0.268 | 1.000 | 3347 | tags=59%, list=15%, signal=69% |
| 194 | GO\_ADAPTATION\_OF\_SIGNALING\_PATHWAY |  | 19 | 0.58 | 1.52 | 0.039 | 0.268 | 1.000 | 2512 | tags=32%, list=12%, signal=36% |
| 195 | GO\_HEART\_GROWTH |  | 24 | 0.62 | 1.52 | 0.029 | 0.269 | 1.000 | 3290 | tags=33%, list=15%, signal=39% |
| 196 | GO\_ACTIN\_FILAMENT\_BASED\_MOVEMENT |  | 91 | 0.75 | 1.52 | 0.026 | 0.270 | 1.000 | 1907 | tags=45%, list=9%, signal=49% |
| 197 | GO\_MULTICELLULAR\_ORGANISMAL\_SIGNALING |  | 115 | 0.50 | 1.52 | 0.014 | 0.270 | 1.000 | 2441 | tags=19%, list=11%, signal=21% |
| 198 | GO\_REGULATION\_OF\_SMALL\_GTPASE\_MEDIATED\_SIGNAL\_TRANSDUCTION |  | 266 | 0.43 | 1.52 | 0.004 | 0.271 | 1.000 | 3270 | tags=32%, list=15%, signal=37% |
| 199 | GO\_REGULATION\_OF\_ACTIN\_FILAMENT\_DEPOLYMERIZATION |  | 44 | 0.62 | 1.52 | 0.029 | 0.270 | 1.000 | 2284 | tags=32%, list=10%, signal=35% |
| 200 | GO\_MYOTUBE\_CELL\_DEVELOPMENT |  | 26 | 0.68 | 1.52 | 0.012 | 0.269 | 1.000 | 1530 | tags=31%, list=7%, signal=33% |
| 201 | GO\_POSITIVE\_REGULATION\_OF\_CELL\_SUBSTRATE\_ADHESION |  | 94 | 0.54 | 1.52 | 0.012 | 0.270 | 1.000 | 2568 | tags=30%, list=12%, signal=34% |
| 202 | GO\_REGULATION\_OF\_STRIATED\_MUSCLE\_CELL\_DIFFERENTIATION |  | 82 | 0.57 | 1.52 | 0.008 | 0.270 | 1.000 | 3290 | tags=41%, list=15%, signal=49% |
| 203 | GO\_POSITIVE\_REGULATION\_OF\_CELLULAR\_RESPONSE\_TO\_TRANSFORMING\_GROWTH\_FACTOR\_BETA\_STIMULUS |  | 24 | 0.58 | 1.52 | 0.021 | 0.272 | 1.000 | 1097 | tags=21%, list=5%, signal=22% |
| 204 | GO\_REGULATION\_OF\_FATTY\_ACID\_OXIDATION |  | 26 | 0.64 | 1.52 | 0.024 | 0.271 | 1.000 | 1569 | tags=27%, list=7%, signal=29% |
| 205 | GO\_CENTRAL\_NERVOUS\_SYSTEM\_PROJECTION\_NEURON\_AXONOGENESIS |  | 22 | 0.71 | 1.52 | 0.008 | 0.270 | 1.000 | 889 | tags=27%, list=4%, signal=28% |
| 206 | GO\_RESPONSE\_TO\_FATTY\_ACID |  | 80 | 0.56 | 1.52 | 0.008 | 0.272 | 1.000 | 2360 | tags=30%, list=11%, signal=34% |
| 207 | GO\_NUCLEOPHAGY |  | 17 | 0.54 | 1.52 | 0.062 | 0.272 | 1.000 | 4735 | tags=53%, list=22%, signal=68% |
| 208 | GO\_CARDIAC\_CHAMBER\_DEVELOPMENT |  | 141 | 0.58 | 1.51 | 0.016 | 0.272 | 1.000 | 3058 | tags=33%, list=14%, signal=39% |
| 209 | GO\_REGULATION\_OF\_STRIATED\_MUSCLE\_CONTRACTION |  | 76 | 0.63 | 1.51 | 0.027 | 0.273 | 1.000 | 2133 | tags=32%, list=10%, signal=35% |
| 210 | GO\_CELLULAR\_RESPONSE\_TO\_PROSTAGLANDIN\_E\_STIMULUS |  | 18 | 0.72 | 1.51 | 0.024 | 0.276 | 1.000 | 2646 | tags=50%, list=12%, signal=57% |
| 211 | GO\_POSITIVE\_REGULATION\_OF\_ION\_TRANSPORT |  | 223 | 0.46 | 1.51 | 0.002 | 0.275 | 1.000 | 3080 | tags=27%, list=14%, signal=32% |
| 212 | GO\_ENDOCARDIAL\_CUSHION\_FORMATION |  | 15 | 0.76 | 1.51 | 0.027 | 0.274 | 1.000 | 2430 | tags=47%, list=11%, signal=52% |
| 213 | GO\_REGULATION\_OF\_CYTOPLASMIC\_TRANSPORT |  | 453 | 0.42 | 1.51 | 0.004 | 0.275 | 1.000 | 3241 | tags=23%, list=15%, signal=26% |
| 214 | GO\_REGULATION\_OF\_MEMBRANE\_DEPOLARIZATION |  | 40 | 0.60 | 1.51 | 0.004 | 0.275 | 1.000 | 3039 | tags=43%, list=14%, signal=49% |
| 215 | GO\_NEGATIVE\_REGULATION\_OF\_CALCIUM\_ION\_TRANSPORT |  | 46 | 0.62 | 1.51 | 0.025 | 0.274 | 1.000 | 2133 | tags=33%, list=10%, signal=36% |
| 216 | GO\_REGULATION\_OF\_POLYSACCHARIDE\_METABOLIC\_PROCESS |  | 40 | 0.54 | 1.51 | 0.015 | 0.273 | 1.000 | 3042 | tags=28%, list=14%, signal=32% |
| 217 | GO\_CELL\_GROWTH |  | 128 | 0.50 | 1.51 | 0.021 | 0.272 | 1.000 | 1158 | tags=16%, list=5%, signal=16% |
| 218 | GO\_CARDIAC\_VENTRICLE\_MORPHOGENESIS |  | 60 | 0.66 | 1.51 | 0.018 | 0.274 | 1.000 | 2182 | tags=38%, list=10%, signal=42% |
| 219 | GO\_REGULATION\_OF\_CELLULAR\_RESPONSE\_TO\_GROWTH\_FACTOR\_STIMULUS |  | 220 | 0.52 | 1.51 | 0.015 | 0.274 | 1.000 | 3304 | tags=30%, list=15%, signal=34% |
| 220 | GO\_CARDIAC\_CHAMBER\_MORPHOGENESIS |  | 102 | 0.60 | 1.51 | 0.016 | 0.274 | 1.000 | 2182 | tags=33%, list=10%, signal=37% |
| 221 | GO\_REGULATION\_OF\_MUSCLE\_HYPERTROPHY |  | 36 | 0.57 | 1.51 | 0.037 | 0.273 | 1.000 | 716 | tags=17%, list=3%, signal=17% |
| 222 | GO\_REGULATION\_OF\_CLATHRIN\_MEDIATED\_ENDOCYTOSIS |  | 16 | 0.56 | 1.51 | 0.028 | 0.272 | 1.000 | 37 | tags=6%, list=0%, signal=6% |
| 223 | GO\_RELAXATION\_OF\_MUSCLE |  | 20 | 0.69 | 1.51 | 0.036 | 0.273 | 1.000 | 2133 | tags=35%, list=10%, signal=39% |
| 224 | GO\_MUSCLE\_TISSUE\_DEVELOPMENT |  | 261 | 0.57 | 1.51 | 0.018 | 0.272 | 1.000 | 2381 | tags=31%, list=11%, signal=34% |
| 225 | GO\_REGULATION\_OF\_COFACTOR\_METABOLIC\_PROCESS |  | 48 | 0.54 | 1.51 | 0.032 | 0.273 | 1.000 | 3035 | tags=25%, list=14%, signal=29% |
| 226 | GO\_MYOFIBRIL\_ASSEMBLY |  | 47 | 0.83 | 1.51 | 0.010 | 0.272 | 1.000 | 2344 | tags=62%, list=11%, signal=69% |
| 227 | GO\_REGULATION\_OF\_HUMORAL\_IMMUNE\_RESPONSE |  | 45 | 0.62 | 1.51 | 0.028 | 0.271 | 1.000 | 3457 | tags=49%, list=16%, signal=58% |
| 228 | GO\_EXOCYTOSIS |  | 292 | 0.44 | 1.50 | 0.000 | 0.270 | 1.000 | 3415 | tags=26%, list=16%, signal=31% |
| 229 | GO\_REGULATION\_OF\_ACTIN\_FILAMENT\_LENGTH |  | 147 | 0.47 | 1.50 | 0.006 | 0.269 | 1.000 | 3170 | tags=28%, list=15%, signal=32% |
| 230 | GO\_MONOAMINE\_TRANSPORT |  | 22 | 0.60 | 1.50 | 0.035 | 0.269 | 1.000 | 3526 | tags=36%, list=16%, signal=43% |
| 231 | GO\_NEGATIVE\_REGULATION\_OF\_GTPASE\_ACTIVITY |  | 39 | 0.55 | 1.50 | 0.014 | 0.268 | 1.000 | 2751 | tags=33%, list=13%, signal=38% |
| 232 | GO\_POSITIVE\_REGULATION\_OF\_CARBOHYDRATE\_METABOLIC\_PROCESS |  | 67 | 0.49 | 1.50 | 0.023 | 0.267 | 1.000 | 3042 | tags=22%, list=14%, signal=26% |
| 233 | GO\_REGULATION\_OF\_MUSCLE\_CONTRACTION |  | 143 | 0.57 | 1.50 | 0.022 | 0.266 | 1.000 | 2142 | tags=29%, list=10%, signal=32% |
| 234 | GO\_REGULATION\_OF\_AMINE\_TRANSPORT |  | 67 | 0.50 | 1.50 | 0.002 | 0.265 | 1.000 | 2386 | tags=19%, list=11%, signal=22% |
| 235 | GO\_POSITIVE\_REGULATION\_OF\_CATION\_CHANNEL\_ACTIVITY |  | 35 | 0.60 | 1.50 | 0.024 | 0.265 | 1.000 | 2956 | tags=40%, list=14%, signal=46% |
| 236 | GO\_MULTICELLULAR\_ORGANISMAL\_MOVEMENT |  | 40 | 0.79 | 1.50 | 0.032 | 0.264 | 1.000 | 1157 | tags=38%, list=5%, signal=40% |
| 237 | GO\_STRIATED\_MUSCLE\_CELL\_DIFFERENTIATION |  | 164 | 0.62 | 1.50 | 0.036 | 0.264 | 1.000 | 2376 | tags=32%, list=11%, signal=36% |
| 238 | GO\_CELL\_FATE\_DETERMINATION |  | 40 | 0.58 | 1.50 | 0.009 | 0.263 | 1.000 | 1963 | tags=18%, list=9%, signal=19% |
| 239 | GO\_REGULATION\_OF\_HEART\_RATE |  | 83 | 0.56 | 1.50 | 0.028 | 0.263 | 1.000 | 2376 | tags=25%, list=11%, signal=28% |
| 240 | GO\_CGMP\_BIOSYNTHETIC\_PROCESS |  | 15 | 0.71 | 1.50 | 0.053 | 0.261 | 1.000 | 1881 | tags=40%, list=9%, signal=44% |
| 241 | GO\_POSITIVE\_REGULATION\_OF\_MYOTUBE\_DIFFERENTIATION |  | 29 | 0.65 | 1.50 | 0.012 | 0.261 | 1.000 | 3531 | tags=45%, list=16%, signal=53% |
| 242 | GO\_ACTOMYOSIN\_STRUCTURE\_ORGANIZATION |  | 75 | 0.71 | 1.50 | 0.027 | 0.260 | 1.000 | 1847 | tags=41%, list=8%, signal=45% |
| 243 | GO\_SMOOTH\_MUSCLE\_CELL\_DIFFERENTIATION |  | 30 | 0.62 | 1.50 | 0.026 | 0.262 | 1.000 | 3290 | tags=47%, list=15%, signal=55% |
| 244 | GO\_REGULATION\_OF\_GLYCOPROTEIN\_METABOLIC\_PROCESS |  | 39 | 0.59 | 1.50 | 0.018 | 0.264 | 1.000 | 3441 | tags=41%, list=16%, signal=49% |
| 245 | GO\_POSITIVE\_REGULATION\_OF\_LIPID\_KINASE\_ACTIVITY |  | 32 | 0.61 | 1.50 | 0.020 | 0.263 | 1.000 | 3170 | tags=44%, list=15%, signal=51% |
| 246 | GO\_MUSCLE\_HYPERTROPHY |  | 28 | 0.70 | 1.50 | 0.027 | 0.263 | 1.000 | 3800 | tags=57%, list=17%, signal=69% |
| 247 | GO\_REGULATION\_OF\_OSSIFICATION |  | 170 | 0.53 | 1.50 | 0.015 | 0.265 | 1.000 | 3170 | tags=33%, list=15%, signal=38% |
| 248 | GO\_ADENYLATE\_CYCLASE\_INHIBITING\_G\_PROTEIN\_COUPLED\_RECEPTOR\_SIGNALING\_PATHWAY |  | 64 | 0.49 | 1.50 | 0.026 | 0.266 | 1.000 | 3452 | tags=23%, list=16%, signal=28% |
| 249 | GO\_ACTIN\_FILAMENT\_ORGANIZATION |  | 164 | 0.50 | 1.50 | 0.014 | 0.266 | 1.000 | 3170 | tags=30%, list=15%, signal=35% |
| 250 | GO\_AMINOGLYCAN\_CATABOLIC\_PROCESS |  | 65 | 0.57 | 1.50 | 0.027 | 0.265 | 1.000 | 3300 | tags=38%, list=15%, signal=45% |
| 251 | GO\_CYCLIC\_NUCLEOTIDE\_CATABOLIC\_PROCESS |  | 16 | 0.66 | 1.50 | 0.033 | 0.264 | 1.000 | 1962 | tags=31%, list=9%, signal=34% |
| 252 | GO\_ORGAN\_MATURATION |  | 18 | 0.72 | 1.50 | 0.037 | 0.263 | 1.000 | 1530 | tags=39%, list=7%, signal=42% |
| 253 | GO\_HYPEROSMOTIC\_RESPONSE |  | 19 | 0.64 | 1.50 | 0.026 | 0.263 | 1.000 | 1096 | tags=21%, list=5%, signal=22% |
| 254 | GO\_REGULATION\_OF\_B\_CELL\_DIFFERENTIATION |  | 22 | 0.75 | 1.50 | 0.025 | 0.263 | 1.000 | 3526 | tags=68%, list=16%, signal=81% |
| 255 | GO\_REGULATION\_OF\_NUCLEOSIDE\_METABOLIC\_PROCESS |  | 46 | 0.49 | 1.49 | 0.041 | 0.264 | 1.000 | 3035 | tags=26%, list=14%, signal=30% |
| 256 | GO\_CARDIAC\_CONDUCTION |  | 79 | 0.52 | 1.49 | 0.054 | 0.266 | 1.000 | 2233 | tags=19%, list=10%, signal=21% |
| 257 | GO\_POSITIVE\_REGULATION\_OF\_MUSCLE\_CELL\_DIFFERENTIATION |  | 81 | 0.54 | 1.49 | 0.025 | 0.267 | 1.000 | 3531 | tags=33%, list=16%, signal=40% |
| 258 | GO\_REGULATION\_OF\_NEUROTRANSMITTER\_RECEPTOR\_ACTIVITY |  | 30 | 0.53 | 1.49 | 0.029 | 0.266 | 1.000 | 1435 | tags=17%, list=7%, signal=18% |
| 259 | GO\_REGULATION\_OF\_BIOMINERAL\_TISSUE\_DEVELOPMENT |  | 69 | 0.58 | 1.49 | 0.025 | 0.266 | 1.000 | 3170 | tags=38%, list=15%, signal=44% |
| 260 | GO\_NEGATIVE\_REGULATION\_OF\_REGULATED\_SECRETORY\_PATHWAY |  | 20 | 0.67 | 1.49 | 0.040 | 0.265 | 1.000 | 3506 | tags=45%, list=16%, signal=54% |
| 261 | GO\_RESPONSE\_TO\_HYDROPEROXIDE |  | 15 | 0.73 | 1.49 | 0.029 | 0.265 | 1.000 | 3085 | tags=60%, list=14%, signal=70% |
| 262 | GO\_POSITIVE\_REGULATION\_OF\_CALCIUM\_MEDIATED\_SIGNALING |  | 36 | 0.64 | 1.49 | 0.029 | 0.266 | 1.000 | 2133 | tags=33%, list=10%, signal=37% |
| 263 | GO\_LEUKOTRIENE\_BIOSYNTHETIC\_PROCESS |  | 20 | 0.70 | 1.49 | 0.036 | 0.266 | 1.000 | 3831 | tags=50%, list=18%, signal=61% |
| 264 | GO\_POSITIVE\_REGULATION\_OF\_OSSIFICATION |  | 82 | 0.55 | 1.49 | 0.028 | 0.267 | 1.000 | 2956 | tags=34%, list=14%, signal=39% |
| 265 | GO\_REGULATION\_OF\_INTERLEUKIN\_4\_PRODUCTION |  | 28 | 0.70 | 1.49 | 0.033 | 0.266 | 1.000 | 4288 | tags=64%, list=20%, signal=80% |
| 266 | GO\_CARDIAC\_VENTRICLE\_DEVELOPMENT |  | 104 | 0.57 | 1.49 | 0.028 | 0.267 | 1.000 | 3058 | tags=33%, list=14%, signal=38% |
| 267 | GO\_POSITIVE\_REGULATION\_OF\_AUTOPHAGY |  | 73 | 0.44 | 1.49 | 0.016 | 0.270 | 1.000 | 4492 | tags=38%, list=21%, signal=48% |
| 268 | GO\_REGULATION\_OF\_POTASSIUM\_ION\_TRANSPORT |  | 82 | 0.46 | 1.49 | 0.027 | 0.269 | 1.000 | 3584 | tags=24%, list=16%, signal=29% |
| 269 | GO\_POSITIVE\_REGULATION\_OF\_LIPID\_METABOLIC\_PROCESS |  | 122 | 0.48 | 1.49 | 0.006 | 0.270 | 1.000 | 3170 | tags=30%, list=15%, signal=34% |
| 270 | GO\_VACUOLE\_ORGANIZATION |  | 155 | 0.38 | 1.48 | 0.021 | 0.274 | 1.000 | 4495 | tags=32%, list=21%, signal=40% |
| 271 | GO\_CALCIUM\_ION\_IMPORT\_INTO\_CYTOSOL |  | 38 | 0.62 | 1.48 | 0.026 | 0.273 | 1.000 | 3852 | tags=53%, list=18%, signal=64% |
| 272 | GO\_B\_CELL\_PROLIFERATION |  | 37 | 0.66 | 1.48 | 0.082 | 0.274 | 1.000 | 2944 | tags=41%, list=14%, signal=47% |
| 273 | GO\_CYCLIC\_NUCLEOTIDE\_BIOSYNTHETIC\_PROCESS |  | 33 | 0.59 | 1.48 | 0.030 | 0.273 | 1.000 | 3648 | tags=42%, list=17%, signal=51% |
| 274 | GO\_REGULATION\_OF\_SMAD\_PROTEIN\_IMPORT\_INTO\_NUCLEUS |  | 15 | 0.67 | 1.48 | 0.055 | 0.275 | 1.000 | 1687 | tags=27%, list=8%, signal=29% |
| 275 | GO\_REGULATION\_OF\_B\_CELL\_PROLIFERATION |  | 54 | 0.64 | 1.48 | 0.057 | 0.275 | 1.000 | 3176 | tags=50%, list=15%, signal=58% |
| 276 | GO\_REGULATION\_OF\_PLATELET\_ACTIVATION |  | 30 | 0.66 | 1.48 | 0.026 | 0.274 | 1.000 | 3526 | tags=50%, list=16%, signal=60% |
| 277 | GO\_POSITIVE\_REGULATION\_OF\_MYOBLAST\_DIFFERENTIATION |  | 22 | 0.73 | 1.48 | 0.043 | 0.274 | 1.000 | 3347 | tags=50%, list=15%, signal=59% |
| 278 | GO\_OLIGODENDROCYTE\_DEVELOPMENT |  | 32 | 0.55 | 1.48 | 0.018 | 0.276 | 1.000 | 1957 | tags=25%, list=9%, signal=27% |
| 279 | GO\_KERATAN\_SULFATE\_METABOLIC\_PROCESS |  | 31 | 0.62 | 1.48 | 0.033 | 0.277 | 1.000 | 3802 | tags=45%, list=17%, signal=55% |
| 280 | GO\_HEART\_DEVELOPMENT |  | 443 | 0.51 | 1.48 | 0.023 | 0.276 | 1.000 | 3433 | tags=32%, list=16%, signal=37% |
| 281 | GO\_MIDBRAIN\_DEVELOPMENT |  | 85 | 0.47 | 1.48 | 0.027 | 0.275 | 1.000 | 2430 | tags=18%, list=11%, signal=20% |
| 282 | GO\_INNER\_EAR\_RECEPTOR\_STEREOCILIUM\_ORGANIZATION |  | 17 | 0.58 | 1.48 | 0.042 | 0.276 | 1.000 | 1188 | tags=18%, list=5%, signal=19% |
| 283 | GO\_SARCOMERE\_ORGANIZATION |  | 26 | 0.83 | 1.48 | 0.016 | 0.276 | 1.000 | 1847 | tags=50%, list=8%, signal=55% |
| 284 | GO\_MEMBRANE\_DEPOLARIZATION |  | 56 | 0.54 | 1.48 | 0.044 | 0.277 | 1.000 | 3233 | tags=29%, list=15%, signal=33% |
| 285 | GO\_BLOOD\_VESSEL\_REMODELING |  | 32 | 0.63 | 1.48 | 0.046 | 0.277 | 1.000 | 2343 | tags=34%, list=11%, signal=38% |
| 286 | GO\_GLUCAN\_BIOSYNTHETIC\_PROCESS |  | 24 | 0.60 | 1.48 | 0.079 | 0.276 | 1.000 | 4951 | tags=54%, list=23%, signal=70% |
| 287 | GO\_RESPONSE\_TO\_STIMULUS\_INVOLVED\_IN\_REGULATION\_OF\_MUSCLE\_ADAPTATION |  | 15 | 0.80 | 1.48 | 0.024 | 0.276 | 1.000 | 1112 | tags=47%, list=5%, signal=49% |
| 288 | GO\_CARDIAC\_MUSCLE\_CELL\_ACTION\_POTENTIAL |  | 36 | 0.60 | 1.47 | 0.026 | 0.279 | 1.000 | 3039 | tags=39%, list=14%, signal=45% |
| 289 | GO\_NEGATIVE\_REGULATION\_OF\_HEART\_CONTRACTION |  | 19 | 0.68 | 1.47 | 0.044 | 0.279 | 1.000 | 1335 | tags=21%, list=6%, signal=22% |
| 290 | GO\_MESENCHYME\_MORPHOGENESIS |  | 37 | 0.65 | 1.47 | 0.019 | 0.279 | 1.000 | 2430 | tags=43%, list=11%, signal=49% |
| 291 | GO\_BLOOD\_COAGULATION\_FIBRIN\_CLOT\_FORMATION |  | 24 | 0.57 | 1.47 | 0.067 | 0.279 | 1.000 | 1931 | tags=29%, list=9%, signal=32% |
| 292 | GO\_CENTRAL\_NERVOUS\_SYSTEM\_NEURON\_AXONOGENESIS |  | 27 | 0.65 | 1.47 | 0.040 | 0.278 | 1.000 | 889 | tags=22%, list=4%, signal=23% |
| 293 | GO\_ARTERY\_DEVELOPMENT |  | 75 | 0.55 | 1.47 | 0.043 | 0.278 | 1.000 | 2956 | tags=36%, list=14%, signal=42% |
| 294 | GO\_NEGATIVE\_REGULATION\_OF\_ENDOTHELIAL\_CELL\_MIGRATION |  | 36 | 0.55 | 1.47 | 0.060 | 0.277 | 1.000 | 2451 | tags=33%, list=11%, signal=38% |
| 295 | GO\_PHAGOCYTOSIS |  | 157 | 0.52 | 1.47 | 0.043 | 0.277 | 1.000 | 3095 | tags=32%, list=14%, signal=37% |
| 296 | GO\_OUTFLOW\_TRACT\_MORPHOGENESIS |  | 56 | 0.60 | 1.47 | 0.038 | 0.277 | 1.000 | 2058 | tags=34%, list=9%, signal=37% |
| 297 | GO\_REGULATION\_OF\_SYSTEMIC\_ARTERIAL\_BLOOD\_PRESSURE\_MEDIATED\_BY\_A\_CHEMICAL\_SIGNAL |  | 45 | 0.59 | 1.47 | 0.044 | 0.277 | 1.000 | 2504 | tags=31%, list=12%, signal=35% |
| 298 | GO\_INTEGRIN\_MEDIATED\_SIGNALING\_PATHWAY |  | 79 | 0.57 | 1.47 | 0.032 | 0.277 | 1.000 | 2474 | tags=34%, list=11%, signal=38% |
| 299 | GO\_NEGATIVE\_REGULATION\_OF\_OSTEOCLAST\_DIFFERENTIATION |  | 23 | 0.66 | 1.47 | 0.044 | 0.277 | 1.000 | 3679 | tags=43%, list=17%, signal=52% |
| 300 | GO\_PLATELET\_ACTIVATION |  | 138 | 0.48 | 1.47 | 0.038 | 0.276 | 1.000 | 3619 | tags=34%, list=17%, signal=41% |
| 301 | GO\_SECRETION\_BY\_CELL |  | 460 | 0.42 | 1.47 | 0.000 | 0.277 | 1.000 | 3663 | tags=26%, list=17%, signal=31% |
| 302 | GO\_RESPONSE\_TO\_TRANSFORMING\_GROWTH\_FACTOR\_BETA |  | 139 | 0.49 | 1.47 | 0.019 | 0.276 | 1.000 | 2230 | tags=26%, list=10%, signal=29% |
| 303 | GO\_PHAGOCYTOSIS\_RECOGNITION |  | 22 | 0.76 | 1.47 | 0.037 | 0.278 | 1.000 | 1802 | tags=50%, list=8%, signal=54% |
| 304 | GO\_REGULATION\_OF\_GLUCOSE\_IMPORT |  | 56 | 0.54 | 1.47 | 0.036 | 0.278 | 1.000 | 3755 | tags=43%, list=17%, signal=52% |
| 305 | GO\_POSITIVE\_REGULATION\_OF\_B\_CELL\_PROLIFERATION |  | 36 | 0.64 | 1.47 | 0.069 | 0.277 | 1.000 | 3176 | tags=50%, list=15%, signal=58% |
| 306 | GO\_NEGATIVE\_REGULATION\_OF\_MUSCLE\_CELL\_APOPTOTIC\_PROCESS |  | 29 | 0.59 | 1.47 | 0.026 | 0.279 | 1.000 | 3619 | tags=48%, list=17%, signal=58% |
| 307 | GO\_HEART\_MORPHOGENESIS |  | 205 | 0.54 | 1.47 | 0.029 | 0.279 | 1.000 | 3058 | tags=33%, list=14%, signal=38% |
| 308 | GO\_SKELETAL\_MUSCLE\_CONTRACTION |  | 31 | 0.83 | 1.47 | 0.043 | 0.279 | 1.000 | 1157 | tags=48%, list=5%, signal=51% |
| 309 | GO\_REGULATION\_OF\_CARBOHYDRATE\_METABOLIC\_PROCESS |  | 154 | 0.44 | 1.46 | 0.006 | 0.279 | 1.000 | 3222 | tags=24%, list=15%, signal=28% |
| 310 | GO\_NEGATIVE\_REGULATION\_OF\_RESPONSE\_TO\_CYTOKINE\_STIMULUS |  | 41 | 0.55 | 1.46 | 0.018 | 0.279 | 1.000 | 2912 | tags=37%, list=13%, signal=42% |
| 311 | GO\_INOSITOL\_LIPID\_MEDIATED\_SIGNALING |  | 116 | 0.45 | 1.46 | 0.006 | 0.279 | 1.000 | 3042 | tags=28%, list=14%, signal=32% |
| 312 | GO\_FOREBRAIN\_CELL\_MIGRATION |  | 60 | 0.48 | 1.46 | 0.033 | 0.278 | 1.000 | 2898 | tags=27%, list=13%, signal=31% |
| 313 | GO\_ACTIN\_MEDIATED\_CELL\_CONTRACTION |  | 72 | 0.79 | 1.46 | 0.036 | 0.282 | 1.000 | 1907 | tags=56%, list=9%, signal=61% |
| 314 | GO\_SULFUR\_COMPOUND\_CATABOLIC\_PROCESS |  | 38 | 0.58 | 1.46 | 0.048 | 0.282 | 1.000 | 2398 | tags=26%, list=11%, signal=30% |
| 315 | GO\_NEGATIVE\_REGULATION\_OF\_CHEMOTAXIS |  | 47 | 0.58 | 1.46 | 0.030 | 0.285 | 1.000 | 3194 | tags=43%, list=15%, signal=50% |
| 316 | GO\_PROTEIN\_TRIMERIZATION |  | 37 | 0.58 | 1.46 | 0.035 | 0.290 | 1.000 | 3917 | tags=41%, list=18%, signal=49% |
| 317 | GO\_EXTRACELLULAR\_MATRIX\_ASSEMBLY |  | 16 | 0.73 | 1.46 | 0.035 | 0.289 | 1.000 | 1726 | tags=50%, list=8%, signal=54% |
| 318 | GO\_POSITIVE\_REGULATION\_OF\_GLYCOPROTEIN\_METABOLIC\_PROCESS |  | 17 | 0.68 | 1.46 | 0.059 | 0.289 | 1.000 | 4440 | tags=65%, list=20%, signal=81% |
| 319 | GO\_REGULATION\_OF\_MEMBRANE\_REPOLARIZATION |  | 29 | 0.60 | 1.46 | 0.050 | 0.288 | 1.000 | 3039 | tags=38%, list=14%, signal=44% |
| 320 | GO\_REGULATION\_OF\_VESICLE\_FUSION |  | 57 | 0.46 | 1.46 | 0.032 | 0.288 | 1.000 | 4327 | tags=40%, list=20%, signal=50% |
| 321 | GO\_REGULATION\_OF\_CARDIAC\_MUSCLE\_CELL\_MEMBRANE\_REPOLARIZATION |  | 20 | 0.66 | 1.46 | 0.041 | 0.288 | 1.000 | 1290 | tags=25%, list=6%, signal=27% |
| 322 | GO\_NEGATIVE\_REGULATION\_OF\_SMOOTH\_MUSCLE\_CELL\_PROLIFERATION |  | 35 | 0.60 | 1.45 | 0.022 | 0.290 | 1.000 | 1468 | tags=26%, list=7%, signal=28% |
| 323 | GO\_POSITIVE\_REGULATION\_OF\_FAT\_CELL\_DIFFERENTIATION |  | 46 | 0.55 | 1.45 | 0.020 | 0.290 | 1.000 | 2791 | tags=28%, list=13%, signal=32% |
| 324 | GO\_NEGATIVE\_REGULATION\_OF\_CALCIUM\_ION\_TRANSPORT\_INTO\_CYTOSOL |  | 17 | 0.67 | 1.45 | 0.042 | 0.289 | 1.000 | 2133 | tags=41%, list=10%, signal=46% |
| 325 | GO\_CENTRAL\_NERVOUS\_SYSTEM\_NEURON\_DEVELOPMENT |  | 70 | 0.51 | 1.45 | 0.033 | 0.289 | 1.000 | 3043 | tags=26%, list=14%, signal=30% |
| 326 | GO\_CELLULAR\_RESPONSE\_TO\_PROSTAGLANDIN\_STIMULUS |  | 23 | 0.67 | 1.45 | 0.034 | 0.288 | 1.000 | 2646 | tags=43%, list=12%, signal=49% |
| 327 | GO\_BRAIN\_MORPHOGENESIS |  | 34 | 0.49 | 1.45 | 0.036 | 0.288 | 1.000 | 1595 | tags=15%, list=7%, signal=16% |
| 328 | GO\_REGULATION\_OF\_EXCRETION |  | 28 | 0.59 | 1.45 | 0.075 | 0.287 | 1.000 | 2646 | tags=29%, list=12%, signal=32% |
| 329 | GO\_VASODILATION |  | 26 | 0.59 | 1.45 | 0.046 | 0.286 | 1.000 | 818 | tags=19%, list=4%, signal=20% |
| 330 | GO\_ADENYLATE\_CYCLASE\_MODULATING\_G\_PROTEIN\_COUPLED\_RECEPTOR\_SIGNALING\_PATHWAY |  | 133 | 0.41 | 1.45 | 0.018 | 0.286 | 1.000 | 3584 | tags=22%, list=16%, signal=26% |
| 331 | GO\_CARDIAC\_MUSCLE\_TISSUE\_DEVELOPMENT |  | 134 | 0.59 | 1.45 | 0.041 | 0.286 | 1.000 | 2344 | tags=31%, list=11%, signal=34% |
| 332 | GO\_NEGATIVE\_REGULATION\_OF\_DEFENSE\_RESPONSE |  | 132 | 0.49 | 1.45 | 0.022 | 0.285 | 1.000 | 3241 | tags=30%, list=15%, signal=35% |
| 333 | GO\_POSITIVE\_REGULATION\_OF\_FILOPODIUM\_ASSEMBLY |  | 23 | 0.61 | 1.45 | 0.026 | 0.285 | 1.000 | 1199 | tags=30%, list=6%, signal=32% |
| 334 | GO\_NEGATIVE\_REGULATION\_OF\_ENDOTHELIAL\_CELL\_PROLIFERATION |  | 30 | 0.59 | 1.45 | 0.065 | 0.285 | 1.000 | 2353 | tags=33%, list=11%, signal=37% |
| 335 | GO\_NEGATIVE\_REGULATION\_OF\_INFLAMMATORY\_RESPONSE |  | 91 | 0.49 | 1.45 | 0.015 | 0.285 | 1.000 | 2829 | tags=29%, list=13%, signal=33% |
| 336 | GO\_CELL\_CELL\_SIGNALING\_INVOLVED\_IN\_CARDIAC\_CONDUCTION |  | 21 | 0.62 | 1.45 | 0.061 | 0.285 | 1.000 | 2233 | tags=29%, list=10%, signal=32% |
| 337 | GO\_REGULATION\_OF\_SKELETAL\_MUSCLE\_TISSUE\_DEVELOPMENT |  | 46 | 0.58 | 1.45 | 0.035 | 0.286 | 1.000 | 3290 | tags=37%, list=15%, signal=43% |
| 338 | GO\_REGULATION\_OF\_RENAL\_SYSTEM\_PROCESS |  | 37 | 0.56 | 1.45 | 0.049 | 0.285 | 1.000 | 2912 | tags=32%, list=13%, signal=37% |
| 339 | GO\_REGULATION\_OF\_SYSTEMIC\_ARTERIAL\_BLOOD\_PRESSURE |  | 82 | 0.50 | 1.45 | 0.028 | 0.285 | 1.000 | 2504 | tags=26%, list=12%, signal=29% |
| 340 | GO\_AORTA\_MORPHOGENESIS |  | 22 | 0.64 | 1.45 | 0.072 | 0.286 | 1.000 | 2732 | tags=41%, list=13%, signal=47% |
| 341 | GO\_NEGATIVE\_REGULATION\_OF\_CALCIUM\_ION\_IMPORT |  | 21 | 0.70 | 1.45 | 0.038 | 0.286 | 1.000 | 2133 | tags=38%, list=10%, signal=42% |
| 342 | GO\_NEGATIVE\_REGULATION\_OF\_TRANSCRIPTION\_FACTOR\_IMPORT\_INTO\_NUCLEUS |  | 36 | 0.47 | 1.45 | 0.030 | 0.286 | 1.000 | 3241 | tags=25%, list=15%, signal=29% |
| 343 | GO\_APOPTOTIC\_CELL\_CLEARANCE |  | 26 | 0.63 | 1.45 | 0.049 | 0.286 | 1.000 | 2912 | tags=38%, list=13%, signal=44% |
| 344 | GO\_RENAL\_SYSTEM\_VASCULATURE\_DEVELOPMENT |  | 19 | 0.65 | 1.45 | 0.070 | 0.286 | 1.000 | 2956 | tags=53%, list=14%, signal=61% |
| 345 | GO\_NEGATIVE\_REGULATION\_OF\_MUSCLE\_CONTRACTION |  | 20 | 0.67 | 1.45 | 0.047 | 0.286 | 1.000 | 1157 | tags=35%, list=5%, signal=37% |
| 346 | GO\_REGULATION\_OF\_PROTEIN\_KINASE\_A\_SIGNALING |  | 17 | 0.59 | 1.45 | 0.045 | 0.286 | 1.000 | 1962 | tags=24%, list=9%, signal=26% |
| 347 | GO\_RECEPTOR\_MEDIATED\_ENDOCYTOSIS |  | 200 | 0.46 | 1.45 | 0.012 | 0.285 | 1.000 | 3798 | tags=32%, list=17%, signal=38% |
| 348 | GO\_POSITIVE\_REGULATION\_OF\_ENDOCYTOSIS |  | 108 | 0.49 | 1.45 | 0.035 | 0.286 | 1.000 | 3800 | tags=38%, list=17%, signal=46% |
| 349 | GO\_REGULATION\_OF\_ACTIN\_FILAMENT\_BASED\_MOVEMENT |  | 32 | 0.57 | 1.45 | 0.038 | 0.286 | 1.000 | 2028 | tags=31%, list=9%, signal=34% |
| 350 | GO\_NEGATIVE\_REGULATION\_OF\_PROTEIN\_SECRETION |  | 101 | 0.48 | 1.45 | 0.008 | 0.285 | 1.000 | 3785 | tags=35%, list=17%, signal=42% |
| 351 | GO\_REGULATION\_OF\_OSTEOBLAST\_DIFFERENTIATION |  | 108 | 0.52 | 1.45 | 0.053 | 0.285 | 1.000 | 3001 | tags=32%, list=14%, signal=37% |
| 352 | GO\_NEGATIVE\_REGULATION\_OF\_OSTEOBLAST\_DIFFERENTIATION |  | 38 | 0.52 | 1.44 | 0.039 | 0.287 | 1.000 | 3001 | tags=37%, list=14%, signal=43% |
| 353 | GO\_EXTRACELLULAR\_STRUCTURE\_ORGANIZATION |  | 288 | 0.58 | 1.44 | 0.054 | 0.287 | 1.000 | 2568 | tags=38%, list=12%, signal=42% |
| 354 | GO\_ENDOCRINE\_PROCESS |  | 44 | 0.59 | 1.44 | 0.048 | 0.286 | 1.000 | 2504 | tags=34%, list=12%, signal=38% |
| 355 | GO\_ACTION\_POTENTIAL |  | 89 | 0.49 | 1.44 | 0.028 | 0.287 | 1.000 | 3039 | tags=25%, list=14%, signal=29% |
| 356 | GO\_GLIAL\_CELL\_MIGRATION |  | 33 | 0.57 | 1.44 | 0.066 | 0.290 | 1.000 | 3310 | tags=36%, list=15%, signal=43% |
| 357 | GO\_SINGLE\_ORGANISM\_CELL\_ADHESION |  | 441 | 0.50 | 1.44 | 0.035 | 0.290 | 1.000 | 2940 | tags=31%, list=14%, signal=35% |
| 358 | GO\_KERATAN\_SULFATE\_BIOSYNTHETIC\_PROCESS |  | 27 | 0.63 | 1.44 | 0.048 | 0.290 | 1.000 | 3802 | tags=44%, list=17%, signal=54% |
| 359 | GO\_ORGAN\_GROWTH |  | 65 | 0.52 | 1.44 | 0.040 | 0.289 | 1.000 | 3482 | tags=31%, list=16%, signal=37% |
| 360 | GO\_REGULATION\_OF\_SYSTEMIC\_ARTERIAL\_BLOOD\_PRESSURE\_BY\_RENIN\_ANGIOTENSIN |  | 22 | 0.70 | 1.44 | 0.052 | 0.289 | 1.000 | 2102 | tags=41%, list=10%, signal=45% |
| 361 | GO\_NEGATIVE\_REGULATION\_OF\_MACROAUTOPHAGY |  | 19 | 0.47 | 1.44 | 0.074 | 0.288 | 1.000 | 3031 | tags=21%, list=14%, signal=24% |
| 362 | GO\_CARDIAC\_MYOFIBRIL\_ASSEMBLY |  | 16 | 0.81 | 1.44 | 0.032 | 0.290 | 1.000 | 2344 | tags=63%, list=11%, signal=70% |
| 363 | GO\_MEGAKARYOCYTE\_DEVELOPMENT |  | 16 | 0.59 | 1.44 | 0.062 | 0.289 | 1.000 | 4065 | tags=44%, list=19%, signal=54% |
| 364 | GO\_REGULATION\_OF\_G\_PROTEIN\_COUPLED\_RECEPTOR\_PROTEIN\_SIGNALING\_PATHWAY |  | 119 | 0.46 | 1.44 | 0.020 | 0.289 | 1.000 | 3347 | tags=28%, list=15%, signal=33% |
| 365 | GO\_HEART\_TRABECULA\_MORPHOGENESIS |  | 24 | 0.58 | 1.44 | 0.033 | 0.291 | 1.000 | 2058 | tags=38%, list=9%, signal=41% |
| 366 | GO\_NEGATIVE\_REGULATION\_OF\_ENDOPLASMIC\_RETICULUM\_STRESS\_INDUCED\_INTRINSIC\_APOPTOTIC\_SIGNALING\_PATHWAY |  | 15 | 0.63 | 1.44 | 0.095 | 0.291 | 1.000 | 2478 | tags=33%, list=11%, signal=38% |
| 367 | GO\_REGULATION\_OF\_PROTEIN\_POLYMERIZATION |  | 164 | 0.40 | 1.44 | 0.006 | 0.292 | 1.000 | 3170 | tags=25%, list=15%, signal=29% |
| 368 | GO\_REGULATION\_OF\_BODY\_FLUID\_LEVELS |  | 478 | 0.41 | 1.44 | 0.010 | 0.292 | 1.000 | 3099 | tags=24%, list=14%, signal=28% |
| 369 | GO\_SKELETAL\_MUSCLE\_CELL\_DIFFERENTIATION |  | 49 | 0.60 | 1.44 | 0.029 | 0.291 | 1.000 | 4070 | tags=47%, list=19%, signal=58% |
| 370 | GO\_EMBRYONIC\_HEMOPOIESIS |  | 19 | 0.55 | 1.44 | 0.047 | 0.291 | 1.000 | 3221 | tags=47%, list=15%, signal=56% |
| 371 | GO\_ACTIVATION\_OF\_IMMUNE\_RESPONSE |  | 384 | 0.55 | 1.44 | 0.079 | 0.290 | 1.000 | 2932 | tags=32%, list=13%, signal=37% |
| 372 | GO\_REGULATION\_OF\_VESICLE\_MEDIATED\_TRANSPORT |  | 435 | 0.39 | 1.44 | 0.000 | 0.290 | 1.000 | 3907 | tags=25%, list=18%, signal=30% |
| 373 | GO\_REGULATION\_OF\_EPITHELIAL\_CELL\_APOPTOTIC\_PROCESS |  | 57 | 0.56 | 1.44 | 0.038 | 0.290 | 1.000 | 3153 | tags=42%, list=14%, signal=49% |
| 374 | GO\_REGULATION\_OF\_VASCULATURE\_DEVELOPMENT |  | 219 | 0.50 | 1.43 | 0.018 | 0.292 | 1.000 | 3290 | tags=38%, list=15%, signal=44% |
| 375 | GO\_RETINA\_VASCULATURE\_DEVELOPMENT\_IN\_CAMERA\_TYPE\_EYE |  | 16 | 0.69 | 1.43 | 0.071 | 0.291 | 1.000 | 2898 | tags=50%, list=13%, signal=58% |
| 376 | GO\_REGULATION\_OF\_RECEPTOR\_ACTIVITY |  | 112 | 0.46 | 1.43 | 0.032 | 0.291 | 1.000 | 1435 | tags=15%, list=7%, signal=16% |
| 377 | GO\_POSITIVE\_REGULATION\_OF\_CALCIUM\_ION\_TRANSPORT |  | 99 | 0.51 | 1.43 | 0.028 | 0.294 | 1.000 | 3080 | tags=33%, list=14%, signal=39% |
| 378 | GO\_HUMORAL\_IMMUNE\_RESPONSE\_MEDIATED\_BY\_CIRCULATING\_IMMUNOGLOBULIN |  | 41 | 0.70 | 1.43 | 0.076 | 0.294 | 1.000 | 3738 | tags=66%, list=17%, signal=79% |
| 379 | GO\_REGULATION\_OF\_TOLERANCE\_INDUCTION |  | 17 | 0.77 | 1.43 | 0.045 | 0.294 | 1.000 | 2231 | tags=53%, list=10%, signal=59% |
| 380 | GO\_REGULATION\_OF\_ATPASE\_ACTIVITY |  | 60 | 0.53 | 1.43 | 0.087 | 0.293 | 1.000 | 3142 | tags=32%, list=14%, signal=37% |
| 381 | GO\_VASCULAR\_PROCESS\_IN\_CIRCULATORY\_SYSTEM |  | 160 | 0.46 | 1.43 | 0.010 | 0.293 | 1.000 | 2142 | tags=24%, list=10%, signal=26% |
| 382 | GO\_REGULATION\_OF\_ANATOMICAL\_STRUCTURE\_SIZE |  | 450 | 0.40 | 1.43 | 0.002 | 0.297 | 1.000 | 3194 | tags=24%, list=15%, signal=28% |
| 383 | GO\_LEUKOCYTE\_PROLIFERATION |  | 86 | 0.60 | 1.43 | 0.079 | 0.298 | 1.000 | 3101 | tags=42%, list=14%, signal=49% |
| 384 | GO\_REGULATION\_OF\_SYSTEMIC\_ARTERIAL\_BLOOD\_PRESSURE\_BY\_HORMONE |  | 35 | 0.61 | 1.43 | 0.059 | 0.298 | 1.000 | 2504 | tags=34%, list=12%, signal=39% |
| 385 | GO\_PROTEIN\_DEGLYCOSYLATION |  | 20 | 0.57 | 1.43 | 0.090 | 0.298 | 1.000 | 2012 | tags=20%, list=9%, signal=22% |
| 386 | GO\_POSITIVE\_REGULATION\_OF\_BLOOD\_PRESSURE |  | 35 | 0.48 | 1.43 | 0.058 | 0.298 | 1.000 | 3800 | tags=29%, list=17%, signal=35% |
| 387 | GO\_B\_CELL\_RECEPTOR\_SIGNALING\_PATHWAY |  | 42 | 0.77 | 1.43 | 0.074 | 0.298 | 1.000 | 3526 | tags=76%, list=16%, signal=91% |
| 388 | GO\_REGULATION\_OF\_TRANSMEMBRANE\_RECEPTOR\_PROTEIN\_SERINE\_THREONINE\_KINASE\_SIGNALING\_PATHWAY |  | 196 | 0.49 | 1.43 | 0.043 | 0.298 | 1.000 | 3304 | tags=26%, list=15%, signal=30% |
| 389 | GO\_ACTIN\_FILAMENT\_POLYMERIZATION |  | 21 | 0.60 | 1.43 | 0.043 | 0.298 | 1.000 | 3709 | tags=57%, list=17%, signal=69% |
| 390 | GO\_MACROMOLECULAR\_COMPLEX\_REMODELING |  | 24 | 0.64 | 1.43 | 0.046 | 0.298 | 1.000 | 2363 | tags=29%, list=11%, signal=33% |
| 391 | GO\_REGULATION\_OF\_GENERATION\_OF\_PRECURSOR\_METABOLITES\_AND\_ENERGY |  | 83 | 0.43 | 1.43 | 0.020 | 0.299 | 1.000 | 3035 | tags=22%, list=14%, signal=25% |
| 392 | GO\_MYELOID\_CELL\_HOMEOSTASIS |  | 84 | 0.44 | 1.42 | 0.047 | 0.299 | 1.000 | 3176 | tags=26%, list=15%, signal=31% |
| 393 | GO\_NEUROTRANSMITTER\_UPTAKE |  | 15 | 0.63 | 1.42 | 0.051 | 0.298 | 1.000 | 3957 | tags=40%, list=18%, signal=49% |
| 394 | GO\_LOCOMOTORY\_BEHAVIOR |  | 170 | 0.43 | 1.42 | 0.022 | 0.298 | 1.000 | 3139 | tags=21%, list=14%, signal=25% |
| 395 | GO\_INTERFERON\_GAMMA\_PRODUCTION |  | 15 | 0.75 | 1.42 | 0.054 | 0.298 | 1.000 | 2349 | tags=53%, list=11%, signal=60% |
| 396 | GO\_HEART\_VALVE\_DEVELOPMENT |  | 32 | 0.62 | 1.42 | 0.062 | 0.299 | 1.000 | 2974 | tags=34%, list=14%, signal=40% |
| 397 | GO\_VASOCONSTRICTION |  | 28 | 0.55 | 1.42 | 0.070 | 0.299 | 1.000 | 3691 | tags=36%, list=17%, signal=43% |
| 398 | GO\_PEPTIDE\_HORMONE\_PROCESSING |  | 29 | 0.62 | 1.42 | 0.050 | 0.299 | 1.000 | 2102 | tags=38%, list=10%, signal=42% |
| 399 | GO\_NEGATIVE\_REGULATION\_OF\_IMMUNE\_SYSTEM\_PROCESS |  | 343 | 0.49 | 1.42 | 0.045 | 0.298 | 1.000 | 3814 | tags=36%, list=18%, signal=43% |
| 400 | GO\_RESPIRATORY\_GASEOUS\_EXCHANGE |  | 47 | 0.52 | 1.42 | 0.041 | 0.298 | 1.000 | 3691 | tags=32%, list=17%, signal=38% |
| 401 | GO\_REGULATION\_OF\_NEURONAL\_SYNAPTIC\_PLASTICITY |  | 46 | 0.48 | 1.42 | 0.048 | 0.297 | 1.000 | 1925 | tags=17%, list=9%, signal=19% |
| 402 | GO\_NUCLEUS\_LOCALIZATION |  | 20 | 0.53 | 1.42 | 0.049 | 0.297 | 1.000 | 3585 | tags=40%, list=16%, signal=48% |
| 403 | GO\_ADAPTIVE\_IMMUNE\_RESPONSE |  | 249 | 0.62 | 1.42 | 0.071 | 0.297 | 1.000 | 3775 | tags=51%, list=17%, signal=61% |
| 404 | GO\_REGULATION\_OF\_ESTABLISHMENT\_OR\_MAINTENANCE\_OF\_CELL\_POLARITY |  | 20 | 0.51 | 1.42 | 0.061 | 0.300 | 1.000 | 4933 | tags=45%, list=23%, signal=58% |
| 405 | GO\_IMMUNE\_RESPONSE\_REGULATING\_CELL\_SURFACE\_RECEPTOR\_SIGNALING\_PATHWAY |  | 285 | 0.53 | 1.42 | 0.088 | 0.300 | 1.000 | 3095 | tags=33%, list=14%, signal=38% |
| 406 | GO\_DEVELOPMENTAL\_MATURATION |  | 181 | 0.41 | 1.42 | 0.002 | 0.300 | 1.000 | 1873 | tags=18%, list=9%, signal=20% |
| 407 | GO\_REGULATION\_OF\_PROTEIN\_IMPORT |  | 172 | 0.44 | 1.42 | 0.013 | 0.300 | 1.000 | 3241 | tags=27%, list=15%, signal=31% |
| 408 | GO\_EMBRYONIC\_HINDLIMB\_MORPHOGENESIS |  | 28 | 0.59 | 1.42 | 0.056 | 0.300 | 1.000 | 3578 | tags=39%, list=16%, signal=47% |
| 409 | GO\_REGULATION\_OF\_MEMBRANE\_POTENTIAL |  | 327 | 0.41 | 1.42 | 0.020 | 0.300 | 1.000 | 3708 | tags=25%, list=17%, signal=30% |
| 410 | GO\_POSITIVE\_REGULATION\_OF\_CALCIUM\_ION\_TRANSPORT\_INTO\_CYTOSOL |  | 48 | 0.55 | 1.42 | 0.054 | 0.300 | 1.000 | 2956 | tags=33%, list=14%, signal=38% |
| 411 | GO\_FC\_GAMMA\_RECEPTOR\_SIGNALING\_PATHWAY |  | 76 | 0.52 | 1.42 | 0.092 | 0.302 | 1.000 | 3095 | tags=30%, list=14%, signal=35% |
| 412 | GO\_APOPTOTIC\_PROCESS\_INVOLVED\_IN\_DEVELOPMENT |  | 20 | 0.58 | 1.42 | 0.054 | 0.302 | 1.000 | 3058 | tags=40%, list=14%, signal=47% |
| 413 | GO\_CARDIAC\_MUSCLE\_TISSUE\_MORPHOGENESIS |  | 52 | 0.66 | 1.42 | 0.050 | 0.302 | 1.000 | 2116 | tags=38%, list=10%, signal=43% |
| 414 | GO\_B\_CELL\_HOMEOSTASIS |  | 21 | 0.58 | 1.42 | 0.067 | 0.302 | 1.000 | 2988 | tags=38%, list=14%, signal=44% |
| 415 | GO\_REGULATION\_OF\_BLOOD\_PRESSURE |  | 162 | 0.45 | 1.42 | 0.020 | 0.303 | 1.000 | 2504 | tags=23%, list=12%, signal=26% |
| 416 | GO\_RESPONSE\_TO\_ACID\_CHEMICAL |  | 301 | 0.43 | 1.41 | 0.004 | 0.303 | 1.000 | 2682 | tags=25%, list=12%, signal=28% |
| 417 | GO\_REGULATION\_OF\_VACUOLAR\_TRANSPORT |  | 29 | 0.53 | 1.41 | 0.069 | 0.303 | 1.000 | 249 | tags=10%, list=1%, signal=10% |
| 418 | GO\_NEGATIVE\_REGULATION\_OF\_I\_KAPPAB\_KINASE\_NF\_KAPPAB\_SIGNALING |  | 47 | 0.48 | 1.41 | 0.051 | 0.303 | 1.000 | 3814 | tags=30%, list=18%, signal=36% |
| 419 | GO\_HEMOSTASIS |  | 293 | 0.42 | 1.41 | 0.040 | 0.302 | 1.000 | 3016 | tags=24%, list=14%, signal=27% |
| 420 | GO\_SPECIFICATION\_OF\_SYMMETRY |  | 110 | 0.45 | 1.41 | 0.054 | 0.303 | 1.000 | 3433 | tags=23%, list=16%, signal=27% |
| 421 | GO\_REGULATION\_OF\_CARBOHYDRATE\_BIOSYNTHETIC\_PROCESS |  | 79 | 0.47 | 1.41 | 0.020 | 0.304 | 1.000 | 3170 | tags=27%, list=15%, signal=31% |
| 422 | GO\_SODIUM\_INDEPENDENT\_ORGANIC\_ANION\_TRANSPORT |  | 21 | 0.55 | 1.41 | 0.062 | 0.304 | 1.000 | 1067 | tags=14%, list=5%, signal=15% |
| 423 | GO\_SUBSTRATE\_ADHESION\_DEPENDENT\_CELL\_SPREADING |  | 38 | 0.57 | 1.41 | 0.062 | 0.305 | 1.000 | 3586 | tags=47%, list=16%, signal=57% |
| 424 | GO\_SOMITE\_DEVELOPMENT |  | 75 | 0.50 | 1.41 | 0.020 | 0.304 | 1.000 | 2259 | tags=23%, list=10%, signal=25% |
| 425 | GO\_NEGATIVE\_REGULATION\_OF\_MUSCLE\_CELL\_DIFFERENTIATION |  | 53 | 0.51 | 1.41 | 0.028 | 0.306 | 1.000 | 3290 | tags=38%, list=15%, signal=44% |
| 426 | GO\_NEGATIVE\_REGULATION\_OF\_GROWTH |  | 226 | 0.42 | 1.41 | 0.009 | 0.305 | 1.000 | 3682 | tags=30%, list=17%, signal=35% |
| 427 | GO\_POSITIVE\_REGULATION\_OF\_INTERLEUKIN\_4\_PRODUCTION |  | 21 | 0.71 | 1.41 | 0.072 | 0.309 | 1.000 | 4288 | tags=67%, list=20%, signal=83% |
| 428 | GO\_DETECTION\_OF\_ABIOTIC\_STIMULUS |  | 108 | 0.40 | 1.41 | 0.041 | 0.311 | 1.000 | 2524 | tags=15%, list=12%, signal=17% |
| 429 | GO\_THYMIC\_T\_CELL\_SELECTION |  | 19 | 0.70 | 1.41 | 0.105 | 0.310 | 1.000 | 2764 | tags=47%, list=13%, signal=54% |
| 430 | GO\_REGULATION\_OF\_ESTABLISHMENT\_OF\_PLANAR\_POLARITY |  | 107 | 0.46 | 1.41 | 0.068 | 0.310 | 1.000 | 2013 | tags=20%, list=9%, signal=22% |
| 431 | GO\_REGULATION\_OF\_RHO\_PROTEIN\_SIGNAL\_TRANSDUCTION |  | 104 | 0.44 | 1.41 | 0.025 | 0.310 | 1.000 | 3239 | tags=36%, list=15%, signal=42% |
| 432 | GO\_PURINE\_CONTAINING\_COMPOUND\_CATABOLIC\_PROCESS |  | 47 | 0.47 | 1.41 | 0.026 | 0.309 | 1.000 | 1962 | tags=21%, list=9%, signal=23% |
| 433 | GO\_DEVELOPMENTAL\_GROWTH\_INVOLVED\_IN\_MORPHOGENESIS |  | 101 | 0.50 | 1.41 | 0.048 | 0.310 | 1.000 | 2966 | tags=25%, list=14%, signal=29% |
| 434 | GO\_MUSCLE\_ORGAN\_MORPHOGENESIS |  | 68 | 0.64 | 1.41 | 0.047 | 0.310 | 1.000 | 2116 | tags=37%, list=10%, signal=41% |
| 435 | GO\_OLFACTORY\_LOBE\_DEVELOPMENT |  | 34 | 0.52 | 1.41 | 0.041 | 0.310 | 1.000 | 2850 | tags=35%, list=13%, signal=41% |
| 436 | GO\_RESPONSE\_TO\_AXON\_INJURY |  | 45 | 0.49 | 1.40 | 0.044 | 0.310 | 1.000 | 2445 | tags=24%, list=11%, signal=27% |
| 437 | GO\_LYMPHOCYTE\_DIFFERENTIATION |  | 204 | 0.54 | 1.40 | 0.076 | 0.310 | 1.000 | 3582 | tags=41%, list=16%, signal=49% |
| 438 | GO\_REGULATION\_OF\_SODIUM\_ION\_TRANSMEMBRANE\_TRANSPORT |  | 47 | 0.54 | 1.40 | 0.063 | 0.309 | 1.000 | 1523 | tags=19%, list=7%, signal=21% |
| 439 | GO\_ENSHEATHMENT\_OF\_NEURONS |  | 86 | 0.47 | 1.40 | 0.064 | 0.310 | 1.000 | 3755 | tags=30%, list=17%, signal=36% |
| 440 | GO\_NEGATIVE\_REGULATION\_OF\_INTERLEUKIN\_6\_PRODUCTION |  | 30 | 0.66 | 1.40 | 0.056 | 0.310 | 1.000 | 2912 | tags=53%, list=13%, signal=61% |
| 441 | GO\_REACTIVE\_OXYGEN\_SPECIES\_METABOLIC\_PROCESS |  | 94 | 0.49 | 1.40 | 0.033 | 0.311 | 1.000 | 2808 | tags=27%, list=13%, signal=30% |
| 442 | GO\_ADAPTIVE\_IMMUNE\_RESPONSE\_BASED\_ON\_SOMATIC\_RECOMBINATION\_OF\_IMMUNE\_RECEPTORS\_BUILT\_FROM\_IMMUNOGLOBULIN\_SUPERFAMILY\_DOMAINS |  | 126 | 0.59 | 1.40 | 0.083 | 0.310 | 1.000 | 3406 | tags=49%, list=16%, signal=58% |
| 443 | GO\_POSITIVE\_REGULATION\_OF\_VASODILATION |  | 31 | 0.56 | 1.40 | 0.057 | 0.309 | 1.000 | 1924 | tags=26%, list=9%, signal=28% |
| 444 | GO\_REGULATION\_OF\_CELL\_ACTIVATION |  | 449 | 0.53 | 1.40 | 0.073 | 0.310 | 1.000 | 3478 | tags=40%, list=16%, signal=46% |
| 445 | GO\_PROTEIN\_LIPID\_COMPLEX\_SUBUNIT\_ORGANIZATION |  | 36 | 0.56 | 1.40 | 0.059 | 0.309 | 1.000 | 3441 | tags=31%, list=16%, signal=36% |
| 446 | GO\_CELLULAR\_COMPONENT\_ASSEMBLY\_INVOLVED\_IN\_MORPHOGENESIS |  | 221 | 0.49 | 1.40 | 0.064 | 0.310 | 1.000 | 2346 | tags=19%, list=11%, signal=21% |
| 447 | GO\_REGULATION\_OF\_FILOPODIUM\_ASSEMBLY |  | 34 | 0.53 | 1.40 | 0.039 | 0.310 | 1.000 | 2503 | tags=32%, list=12%, signal=37% |
| 448 | GO\_NEGATIVE\_REGULATION\_OF\_EPITHELIAL\_CELL\_MIGRATION |  | 50 | 0.49 | 1.40 | 0.077 | 0.310 | 1.000 | 1925 | tags=26%, list=9%, signal=28% |
| 449 | GO\_REGULATION\_OF\_PROTEIN\_SECRETION |  | 368 | 0.42 | 1.40 | 0.016 | 0.309 | 1.000 | 3736 | tags=29%, list=17%, signal=35% |
| 450 | GO\_NEUROTROPHIN\_SIGNALING\_PATHWAY |  | 23 | 0.52 | 1.40 | 0.080 | 0.310 | 1.000 | 4187 | tags=48%, list=19%, signal=59% |
| 451 | GO\_TISSUE\_REGENERATION |  | 48 | 0.53 | 1.40 | 0.034 | 0.309 | 1.000 | 2079 | tags=29%, list=10%, signal=32% |
| 452 | GO\_REGULATION\_OF\_CARDIAC\_MUSCLE\_CONTRACTION |  | 64 | 0.50 | 1.40 | 0.042 | 0.309 | 1.000 | 3058 | tags=30%, list=14%, signal=34% |
| 453 | GO\_REGULATION\_OF\_CELL\_SUBSTRATE\_ADHESION |  | 163 | 0.48 | 1.40 | 0.050 | 0.309 | 1.000 | 2647 | tags=26%, list=12%, signal=29% |
| 454 | GO\_CARDIAC\_SEPTUM\_DEVELOPMENT |  | 84 | 0.54 | 1.40 | 0.071 | 0.309 | 1.000 | 4020 | tags=36%, list=18%, signal=44% |
| 455 | GO\_ASTROCYTE\_DIFFERENTIATION |  | 38 | 0.49 | 1.40 | 0.041 | 0.308 | 1.000 | 3614 | tags=29%, list=17%, signal=35% |
| 456 | GO\_CELLULAR\_RESPONSE\_TO\_AMINO\_ACID\_STIMULUS |  | 49 | 0.50 | 1.40 | 0.064 | 0.308 | 1.000 | 4002 | tags=43%, list=18%, signal=52% |
| 457 | GO\_REGULATION\_OF\_ENDOCYTOSIS |  | 189 | 0.43 | 1.40 | 0.045 | 0.308 | 1.000 | 3138 | tags=24%, list=14%, signal=28% |
| 458 | GO\_CELLULAR\_RESPONSE\_TO\_VASCULAR\_ENDOTHELIAL\_GROWTH\_FACTOR\_STIMULUS |  | 30 | 0.57 | 1.40 | 0.084 | 0.309 | 1.000 | 3101 | tags=47%, list=14%, signal=54% |
| 459 | GO\_HOMEOSTASIS\_OF\_NUMBER\_OF\_CELLS |  | 169 | 0.43 | 1.40 | 0.049 | 0.309 | 1.000 | 3554 | tags=31%, list=16%, signal=36% |
| 460 | GO\_NEGATIVE\_REGULATION\_OF\_BLOOD\_CIRCULATION |  | 33 | 0.54 | 1.40 | 0.094 | 0.309 | 1.000 | 1368 | tags=18%, list=6%, signal=19% |
| 461 | GO\_REGULATION\_OF\_TRANSCRIPTION\_REGULATORY\_REGION\_DNA\_BINDING |  | 35 | 0.52 | 1.40 | 0.062 | 0.309 | 1.000 | 2430 | tags=23%, list=11%, signal=26% |
| 462 | GO\_NEGATIVE\_REGULATION\_OF\_MUSCLE\_TISSUE\_DEVELOPMENT |  | 35 | 0.55 | 1.40 | 0.069 | 0.308 | 1.000 | 3985 | tags=40%, list=18%, signal=49% |
| 463 | GO\_NEGATIVE\_REGULATION\_OF\_TUMOR\_NECROSIS\_FACTOR\_SUPERFAMILY\_CYTOKINE\_PRODUCTION |  | 37 | 0.61 | 1.40 | 0.075 | 0.308 | 1.000 | 2912 | tags=46%, list=13%, signal=53% |
| 464 | GO\_CARDIAC\_ATRIUM\_DEVELOPMENT |  | 30 | 0.57 | 1.40 | 0.069 | 0.308 | 1.000 | 3433 | tags=33%, list=16%, signal=40% |
| 465 | GO\_REGULATION\_OF\_SYNCYTIUM\_FORMATION\_BY\_PLASMA\_MEMBRANE\_FUSION |  | 23 | 0.60 | 1.40 | 0.052 | 0.308 | 1.000 | 3531 | tags=52%, list=16%, signal=62% |
| 466 | GO\_REGULATION\_OF\_SMOOTH\_MUSCLE\_CELL\_DIFFERENTIATION |  | 19 | 0.63 | 1.40 | 0.086 | 0.308 | 1.000 | 3290 | tags=42%, list=15%, signal=50% |
| 467 | GO\_NEGATIVE\_REGULATION\_OF\_EMBRYONIC\_DEVELOPMENT |  | 26 | 0.63 | 1.40 | 0.089 | 0.309 | 1.000 | 2014 | tags=35%, list=9%, signal=38% |
| 468 | GO\_MUSCLE\_ADAPTATION |  | 29 | 0.62 | 1.39 | 0.094 | 0.309 | 1.000 | 3800 | tags=55%, list=17%, signal=67% |
| 469 | GO\_CELLULAR\_RESPONSE\_TO\_ACID\_CHEMICAL |  | 161 | 0.46 | 1.39 | 0.027 | 0.309 | 1.000 | 2682 | tags=28%, list=12%, signal=32% |
| 470 | GO\_POSITIVE\_REGULATION\_OF\_CELL\_ADHESION |  | 355 | 0.50 | 1.39 | 0.040 | 0.308 | 1.000 | 3170 | tags=34%, list=15%, signal=39% |
| 471 | GO\_BLOOD\_COAGULATION\_INTRINSIC\_PATHWAY |  | 17 | 0.55 | 1.39 | 0.123 | 0.308 | 1.000 | 1931 | tags=29%, list=9%, signal=32% |
| 472 | GO\_MYOBLAST\_FUSION |  | 18 | 0.59 | 1.39 | 0.087 | 0.308 | 1.000 | 1418 | tags=22%, list=7%, signal=24% |
| 473 | GO\_OSSIFICATION |  | 235 | 0.49 | 1.39 | 0.053 | 0.308 | 1.000 | 3035 | tags=31%, list=14%, signal=35% |
| 474 | GO\_REGULATION\_OF\_THE\_FORCE\_OF\_HEART\_CONTRACTION |  | 28 | 0.66 | 1.39 | 0.106 | 0.308 | 1.000 | 1255 | tags=29%, list=6%, signal=30% |
| 475 | GO\_POSITIVE\_REGULATION\_OF\_ENDOTHELIAL\_CELL\_DIFFERENTIATION |  | 15 | 0.66 | 1.39 | 0.089 | 0.308 | 1.000 | 3549 | tags=47%, list=16%, signal=56% |
| 476 | GO\_LYTIC\_VACUOLE\_ORGANIZATION |  | 49 | 0.44 | 1.39 | 0.054 | 0.309 | 1.000 | 4495 | tags=35%, list=21%, signal=44% |
| 477 | GO\_POSITIVE\_REGULATION\_OF\_CHONDROCYTE\_DIFFERENTIATION |  | 18 | 0.60 | 1.39 | 0.048 | 0.308 | 1.000 | 3407 | tags=39%, list=16%, signal=46% |
| 478 | GO\_NEGATIVE\_REGULATION\_OF\_LEUKOCYTE\_APOPTOTIC\_PROCESS |  | 42 | 0.65 | 1.39 | 0.056 | 0.308 | 1.000 | 3176 | tags=62%, list=15%, signal=72% |
| 479 | GO\_NEGATIVE\_REGULATION\_OF\_INTERLEUKIN\_12\_PRODUCTION |  | 15 | 0.77 | 1.39 | 0.076 | 0.307 | 1.000 | 2280 | tags=60%, list=10%, signal=67% |
| 480 | GO\_REGULATION\_OF\_CALCINEURIN\_NFAT\_SIGNALING\_CASCADE |  | 16 | 0.59 | 1.39 | 0.072 | 0.307 | 1.000 | 581 | tags=19%, list=3%, signal=19% |
| 481 | GO\_POTASSIUM\_ION\_TRANSPORT |  | 146 | 0.39 | 1.39 | 0.030 | 0.309 | 1.000 | 3984 | tags=24%, list=18%, signal=29% |
| 482 | GO\_NEGATIVE\_REGULATION\_OF\_MITOCHONDRION\_ORGANIZATION |  | 35 | 0.46 | 1.39 | 0.057 | 0.311 | 1.000 | 2386 | tags=29%, list=11%, signal=32% |
| 483 | GO\_REGULATION\_OF\_RECEPTOR\_MEDIATED\_ENDOCYTOSIS |  | 75 | 0.44 | 1.39 | 0.036 | 0.311 | 1.000 | 3561 | tags=25%, list=16%, signal=30% |
| 484 | GO\_NEGATIVE\_REGULATION\_OF\_CALCIUM\_MEDIATED\_SIGNALING |  | 16 | 0.65 | 1.39 | 0.089 | 0.312 | 1.000 | 3016 | tags=44%, list=14%, signal=51% |
| 485 | GO\_REGULATION\_OF\_PLASMA\_LIPOPROTEIN\_PARTICLE\_LEVELS |  | 45 | 0.54 | 1.39 | 0.056 | 0.312 | 1.000 | 3441 | tags=31%, list=16%, signal=37% |
| 486 | GO\_NEGATIVE\_REGULATION\_OF\_ACTIN\_FILAMENT\_BUNDLE\_ASSEMBLY |  | 19 | 0.54 | 1.39 | 0.084 | 0.312 | 1.000 | 1465 | tags=26%, list=7%, signal=28% |
| 487 | GO\_GLOMERULUS\_DEVELOPMENT |  | 49 | 0.55 | 1.39 | 0.064 | 0.311 | 1.000 | 2089 | tags=35%, list=10%, signal=38% |
| 488 | GO\_RESPONSE\_TO\_ELECTRICAL\_STIMULUS |  | 42 | 0.49 | 1.39 | 0.073 | 0.313 | 1.000 | 1556 | tags=17%, list=7%, signal=18% |
| 489 | GO\_POSITIVE\_REGULATION\_OF\_B\_CELL\_ACTIVATION |  | 73 | 0.61 | 1.39 | 0.123 | 0.312 | 1.000 | 3526 | tags=56%, list=16%, signal=67% |
| 490 | GO\_RESPONSE\_TO\_MUSCLE\_ACTIVITY |  | 18 | 0.69 | 1.39 | 0.081 | 0.312 | 1.000 | 1797 | tags=44%, list=8%, signal=48% |
| 491 | GO\_REGULATION\_OF\_ENDOTHELIAL\_CELL\_PROLIFERATION |  | 93 | 0.50 | 1.39 | 0.048 | 0.311 | 1.000 | 3967 | tags=42%, list=18%, signal=51% |
| 492 | GO\_CHONDROITIN\_SULFATE\_PROTEOGLYCAN\_METABOLIC\_PROCESS |  | 42 | 0.58 | 1.39 | 0.065 | 0.311 | 1.000 | 3553 | tags=40%, list=16%, signal=48% |
| 493 | GO\_REGULATION\_OF\_VASOCONSTRICTION |  | 65 | 0.49 | 1.39 | 0.033 | 0.311 | 1.000 | 2461 | tags=26%, list=11%, signal=29% |
| 494 | GO\_REGULATION\_OF\_ERAD\_PATHWAY |  | 27 | 0.47 | 1.39 | 0.114 | 0.310 | 1.000 | 4242 | tags=33%, list=20%, signal=41% |
| 495 | GO\_REGULATION\_OF\_MYOBLAST\_DIFFERENTIATION |  | 46 | 0.57 | 1.39 | 0.071 | 0.310 | 1.000 | 3347 | tags=39%, list=15%, signal=46% |
| 496 | GO\_MYD88\_DEPENDENT\_TOLL\_LIKE\_RECEPTOR\_SIGNALING\_PATHWAY |  | 32 | 0.62 | 1.39 | 0.109 | 0.309 | 1.000 | 2680 | tags=31%, list=12%, signal=36% |
| 497 | GO\_NEGATIVE\_REGULATION\_OF\_RESPONSE\_TO\_ENDOPLASMIC\_RETICULUM\_STRESS |  | 33 | 0.50 | 1.39 | 0.096 | 0.309 | 1.000 | 2478 | tags=21%, list=11%, signal=24% |
| 498 | GO\_ADULT\_LOCOMOTORY\_BEHAVIOR |  | 76 | 0.45 | 1.39 | 0.050 | 0.308 | 1.000 | 3139 | tags=21%, list=14%, signal=25% |
| 499 | GO\_MUSCLE\_FIBER\_DEVELOPMENT |  | 43 | 0.64 | 1.39 | 0.071 | 0.308 | 1.000 | 1969 | tags=35%, list=9%, signal=38% |
| 500 | GO\_COLLAGEN\_FIBRIL\_ORGANIZATION |  | 36 | 0.73 | 1.38 | 0.121 | 0.310 | 1.000 | 2253 | tags=50%, list=10%, signal=56% |
| 501 | GO\_NEGATIVE\_REGULATION\_OF\_EPITHELIAL\_CELL\_APOPTOTIC\_PROCESS |  | 35 | 0.57 | 1.38 | 0.052 | 0.311 | 1.000 | 3153 | tags=40%, list=14%, signal=47% |
| 502 | GO\_NATURAL\_KILLER\_CELL\_ACTIVATION |  | 53 | 0.60 | 1.38 | 0.113 | 0.310 | 1.000 | 2343 | tags=28%, list=11%, signal=32% |
| 503 | GO\_NEGATIVE\_REGULATION\_OF\_OSSIFICATION |  | 65 | 0.46 | 1.38 | 0.057 | 0.310 | 1.000 | 3355 | tags=34%, list=15%, signal=40% |
| 504 | GO\_ANTIGEN\_RECEPTOR\_MEDIATED\_SIGNALING\_PATHWAY |  | 173 | 0.58 | 1.38 | 0.102 | 0.311 | 1.000 | 3084 | tags=38%, list=14%, signal=43% |
| 505 | GO\_REGULATION\_OF\_RECEPTOR\_BINDING |  | 17 | 0.60 | 1.38 | 0.075 | 0.311 | 1.000 | 2898 | tags=47%, list=13%, signal=54% |
| 506 | GO\_POSITIVE\_REGULATION\_OF\_RECEPTOR\_MEDIATED\_ENDOCYTOSIS |  | 44 | 0.52 | 1.38 | 0.055 | 0.310 | 1.000 | 3798 | tags=41%, list=17%, signal=49% |
| 507 | GO\_DENDRITIC\_CELL\_MIGRATION |  | 21 | 0.77 | 1.38 | 0.079 | 0.310 | 1.000 | 2170 | tags=57%, list=10%, signal=63% |
| 508 | GO\_NEGATIVE\_REGULATION\_OF\_BIOMINERAL\_TISSUE\_DEVELOPMENT |  | 18 | 0.61 | 1.38 | 0.079 | 0.310 | 1.000 | 4338 | tags=50%, list=20%, signal=62% |
| 509 | GO\_CELLULAR\_RESPONSE\_TO\_NITROGEN\_COMPOUND |  | 479 | 0.37 | 1.38 | 0.006 | 0.309 | 1.000 | 2860 | tags=20%, list=13%, signal=22% |
| 510 | GO\_REGULATION\_OF\_SKELETAL\_MUSCLE\_CELL\_DIFFERENTIATION |  | 15 | 0.62 | 1.38 | 0.075 | 0.310 | 1.000 | 2052 | tags=33%, list=9%, signal=37% |
| 511 | GO\_SOMITOGENESIS |  | 60 | 0.49 | 1.38 | 0.034 | 0.311 | 1.000 | 2116 | tags=22%, list=10%, signal=24% |
| 512 | GO\_NEGATIVE\_REGULATION\_OF\_ADHERENS\_JUNCTION\_ORGANIZATION |  | 15 | 0.62 | 1.38 | 0.098 | 0.311 | 1.000 | 1282 | tags=27%, list=6%, signal=28% |
| 513 | GO\_CELL\_DIFFERENTIATION\_INVOLVED\_IN\_KIDNEY\_DEVELOPMENT |  | 35 | 0.52 | 1.38 | 0.050 | 0.310 | 1.000 | 3267 | tags=29%, list=15%, signal=34% |
| 514 | GO\_REGULATION\_OF\_B\_CELL\_ACTIVATION |  | 108 | 0.58 | 1.38 | 0.123 | 0.310 | 1.000 | 3526 | tags=51%, list=16%, signal=60% |
| 515 | GO\_TRANSMEMBRANE\_RECEPTOR\_PROTEIN\_SERINE\_THREONINE\_KINASE\_SIGNALING\_PATHWAY |  | 183 | 0.42 | 1.38 | 0.037 | 0.311 | 1.000 | 3080 | tags=24%, list=14%, signal=28% |
| 516 | GO\_POSITIVE\_REGULATION\_OF\_STEROL\_TRANSPORT |  | 17 | 0.61 | 1.38 | 0.083 | 0.311 | 1.000 | 3222 | tags=41%, list=15%, signal=48% |
| 517 | GO\_NEGATIVE\_REGULATION\_OF\_NF\_KAPPAB\_TRANSCRIPTION\_FACTOR\_ACTIVITY |  | 60 | 0.45 | 1.38 | 0.050 | 0.310 | 1.000 | 3620 | tags=23%, list=17%, signal=28% |
| 518 | GO\_CAMP\_METABOLIC\_PROCESS |  | 32 | 0.54 | 1.38 | 0.064 | 0.310 | 1.000 | 1962 | tags=28%, list=9%, signal=31% |
| 519 | GO\_NEGATIVE\_REGULATION\_OF\_PEPTIDYL\_SERINE\_PHOSPHORYLATION |  | 21 | 0.51 | 1.38 | 0.059 | 0.309 | 1.000 | 1855 | tags=19%, list=9%, signal=21% |
| 520 | GO\_CELL\_MORPHOGENESIS\_INVOLVED\_IN\_NEURON\_DIFFERENTIATION |  | 343 | 0.43 | 1.38 | 0.039 | 0.309 | 1.000 | 2089 | tags=16%, list=10%, signal=17% |
| 521 | GO\_REGULATION\_OF\_RAS\_PROTEIN\_SIGNAL\_TRANSDUCTION |  | 177 | 0.39 | 1.38 | 0.034 | 0.308 | 1.000 | 3239 | tags=29%, list=15%, signal=34% |
| 522 | GO\_ENDOCARDIAL\_CUSHION\_DEVELOPMENT |  | 32 | 0.61 | 1.38 | 0.079 | 0.309 | 1.000 | 2956 | tags=38%, list=14%, signal=43% |
| 523 | GO\_POSITIVE\_REGULATION\_OF\_WNT\_SIGNALING\_PATHWAY |  | 144 | 0.40 | 1.38 | 0.041 | 0.308 | 1.000 | 2125 | tags=17%, list=10%, signal=18% |
| 524 | GO\_LYMPHOCYTE\_COSTIMULATION |  | 73 | 0.64 | 1.38 | 0.106 | 0.308 | 1.000 | 2764 | tags=41%, list=13%, signal=47% |
| 525 | GO\_CHAPERONE\_MEDIATED\_PROTEIN\_FOLDING |  | 43 | 0.42 | 1.38 | 0.055 | 0.307 | 1.000 | 2396 | tags=19%, list=11%, signal=21% |
| 526 | GO\_TOLL\_LIKE\_RECEPTOR\_4\_SIGNALING\_PATHWAY |  | 17 | 0.68 | 1.38 | 0.100 | 0.307 | 1.000 | 1602 | tags=35%, list=7%, signal=38% |
| 527 | GO\_CARDIOCYTE\_DIFFERENTIATION |  | 91 | 0.54 | 1.38 | 0.073 | 0.307 | 1.000 | 3586 | tags=38%, list=16%, signal=46% |
| 528 | GO\_NEGATIVE\_REGULATION\_OF\_CELL\_DEVELOPMENT |  | 287 | 0.41 | 1.38 | 0.023 | 0.309 | 1.000 | 3010 | tags=22%, list=14%, signal=25% |
| 529 | GO\_MEMBRANE\_INVAGINATION |  | 36 | 0.63 | 1.38 | 0.110 | 0.308 | 1.000 | 3324 | tags=44%, list=15%, signal=52% |
| 530 | GO\_BODY\_MORPHOGENESIS |  | 42 | 0.52 | 1.38 | 0.072 | 0.309 | 1.000 | 3826 | tags=40%, list=18%, signal=49% |
| 531 | GO\_LYMPHOCYTE\_ACTIVATION |  | 331 | 0.55 | 1.38 | 0.099 | 0.310 | 1.000 | 3478 | tags=41%, list=16%, signal=48% |
| 532 | GO\_POSITIVE\_REGULATION\_OF\_BINDING |  | 122 | 0.40 | 1.38 | 0.024 | 0.309 | 1.000 | 3406 | tags=26%, list=16%, signal=31% |
| 533 | GO\_CELLULAR\_RESPONSE\_TO\_PEPTIDE |  | 263 | 0.36 | 1.38 | 0.013 | 0.310 | 1.000 | 2581 | tags=17%, list=12%, signal=19% |
| 534 | GO\_MYELOID\_CELL\_DIFFERENTIATION |  | 183 | 0.44 | 1.37 | 0.049 | 0.311 | 1.000 | 3457 | tags=30%, list=16%, signal=35% |
| 535 | GO\_TISSUE\_REMODELING |  | 86 | 0.49 | 1.37 | 0.055 | 0.310 | 1.000 | 2647 | tags=28%, list=12%, signal=32% |
| 536 | GO\_MESENCHYME\_DEVELOPMENT |  | 181 | 0.49 | 1.37 | 0.041 | 0.310 | 1.000 | 3424 | tags=32%, list=16%, signal=38% |
| 537 | GO\_NEGATIVE\_REGULATION\_OF\_CATION\_CHANNEL\_ACTIVITY |  | 31 | 0.51 | 1.37 | 0.078 | 0.310 | 1.000 | 2747 | tags=19%, list=13%, signal=22% |
| 538 | GO\_POSITIVE\_REGULATION\_OF\_BIOMINERAL\_TISSUE\_DEVELOPMENT |  | 36 | 0.56 | 1.37 | 0.044 | 0.309 | 1.000 | 2956 | tags=39%, list=14%, signal=45% |
| 539 | GO\_LYMPHOCYTE\_MEDIATED\_IMMUNITY |  | 115 | 0.58 | 1.37 | 0.105 | 0.310 | 1.000 | 3775 | tags=50%, list=17%, signal=61% |
| 540 | GO\_TRANSMEMBRANE\_RECEPTOR\_PROTEIN\_TYROSINE\_KINASE\_SIGNALING\_PATHWAY |  | 483 | 0.37 | 1.37 | 0.014 | 0.311 | 1.000 | 3652 | tags=26%, list=17%, signal=30% |
| 541 | GO\_REGULATION\_OF\_POTASSIUM\_ION\_TRANSMEMBRANE\_TRANSPORT |  | 60 | 0.45 | 1.37 | 0.068 | 0.311 | 1.000 | 2747 | tags=20%, list=13%, signal=23% |
| 542 | GO\_REGULATION\_OF\_CELLULAR\_COMPONENT\_SIZE |  | 318 | 0.38 | 1.37 | 0.010 | 0.311 | 1.000 | 3194 | tags=24%, list=15%, signal=28% |
| 543 | GO\_REGULATION\_OF\_MITOCHONDRIAL\_FISSION |  | 15 | 0.55 | 1.37 | 0.081 | 0.311 | 1.000 | 2386 | tags=33%, list=11%, signal=37% |
| 544 | GO\_REGULATION\_OF\_ALPHA\_AMINO\_3\_HYDROXY\_5\_METHYL\_4\_ISOXAZOLE\_PROPIONATE\_SELECTIVE\_GLUTAMATE\_RECEPTOR\_ACTIVITY |  | 19 | 0.56 | 1.37 | 0.102 | 0.311 | 1.000 | 1435 | tags=16%, list=7%, signal=17% |
| 545 | GO\_REGULATION\_OF\_HEART\_RATE\_BY\_CARDIAC\_CONDUCTION |  | 29 | 0.52 | 1.37 | 0.084 | 0.312 | 1.000 | 2233 | tags=28%, list=10%, signal=31% |
| 546 | GO\_B\_CELL\_ACTIVATION |  | 127 | 0.51 | 1.37 | 0.125 | 0.311 | 1.000 | 3448 | tags=38%, list=16%, signal=45% |
| 547 | GO\_POSITIVE\_REGULATION\_OF\_CELL\_PROJECTION\_ORGANIZATION |  | 288 | 0.40 | 1.37 | 0.026 | 0.311 | 1.000 | 3681 | tags=28%, list=17%, signal=33% |
| 548 | GO\_NEGATIVE\_REGULATION\_OF\_EPITHELIAL\_CELL\_PROLIFERATION |  | 110 | 0.44 | 1.37 | 0.042 | 0.311 | 1.000 | 3067 | tags=26%, list=14%, signal=31% |
| 549 | GO\_NEGATIVE\_REGULATION\_OF\_RESPONSE\_TO\_EXTERNAL\_STIMULUS |  | 254 | 0.42 | 1.37 | 0.020 | 0.311 | 1.000 | 3241 | tags=26%, list=15%, signal=31% |
| 550 | GO\_REGULATION\_OF\_INFLAMMATORY\_RESPONSE |  | 272 | 0.48 | 1.37 | 0.054 | 0.311 | 1.000 | 3241 | tags=34%, list=15%, signal=39% |
| 551 | GO\_COPULATION |  | 17 | 0.60 | 1.37 | 0.081 | 0.311 | 1.000 | 3691 | tags=35%, list=17%, signal=42% |
| 552 | GO\_TISSUE\_MIGRATION |  | 79 | 0.49 | 1.37 | 0.064 | 0.311 | 1.000 | 3298 | tags=37%, list=15%, signal=43% |
| 553 | GO\_POSITIVE\_REGULATION\_OF\_STRIATED\_MUSCLE\_CELL\_DIFFERENTIATION |  | 50 | 0.53 | 1.37 | 0.094 | 0.311 | 1.000 | 3531 | tags=38%, list=16%, signal=45% |
| 554 | GO\_CELLULAR\_RESPONSE\_TO\_KETONE |  | 71 | 0.47 | 1.37 | 0.046 | 0.311 | 1.000 | 1783 | tags=25%, list=8%, signal=28% |
| 555 | GO\_GLIOGENESIS |  | 166 | 0.42 | 1.37 | 0.040 | 0.310 | 1.000 | 3906 | tags=30%, list=18%, signal=36% |
| 556 | GO\_POSITIVE\_REGULATION\_OF\_OSTEOBLAST\_DIFFERENTIATION |  | 58 | 0.53 | 1.37 | 0.079 | 0.313 | 1.000 | 2646 | tags=31%, list=12%, signal=35% |
| 557 | GO\_REGULATION\_OF\_MITOCHONDRIAL\_DEPOLARIZATION |  | 17 | 0.64 | 1.37 | 0.048 | 0.312 | 1.000 | 2988 | tags=65%, list=14%, signal=75% |
| 558 | GO\_PROTEIN\_TARGETING\_TO\_PLASMA\_MEMBRANE |  | 23 | 0.54 | 1.37 | 0.084 | 0.312 | 1.000 | 4030 | tags=43%, list=19%, signal=53% |
| 559 | GO\_POSITIVE\_REGULATION\_OF\_RELEASE\_OF\_SEQUESTERED\_CALCIUM\_ION\_INTO\_CYTOSOL |  | 35 | 0.55 | 1.37 | 0.078 | 0.313 | 1.000 | 4129 | tags=46%, list=19%, signal=56% |
| 560 | GO\_B\_CELL\_MEDIATED\_IMMUNITY |  | 71 | 0.60 | 1.37 | 0.128 | 0.313 | 1.000 | 4002 | tags=58%, list=18%, signal=71% |
| 561 | GO\_MATURE\_B\_CELL\_DIFFERENTIATION |  | 16 | 0.62 | 1.37 | 0.103 | 0.313 | 1.000 | 4881 | tags=63%, list=22%, signal=81% |
| 562 | GO\_NEGATIVE\_REGULATION\_OF\_CELL\_GROWTH |  | 163 | 0.42 | 1.37 | 0.021 | 0.313 | 1.000 | 3682 | tags=29%, list=17%, signal=35% |
| 563 | GO\_REGULATION\_OF\_TUMOR\_NECROSIS\_FACTOR\_SUPERFAMILY\_CYTOKINE\_PRODUCTION |  | 91 | 0.57 | 1.37 | 0.091 | 0.313 | 1.000 | 3084 | tags=40%, list=14%, signal=46% |
| 564 | GO\_REGULATION\_OF\_ACTIN\_FILAMENT\_BUNDLE\_ASSEMBLY |  | 73 | 0.44 | 1.37 | 0.047 | 0.312 | 1.000 | 3655 | tags=32%, list=17%, signal=38% |
| 565 | GO\_REGULATION\_OF\_CELL\_SHAPE |  | 131 | 0.47 | 1.37 | 0.057 | 0.312 | 1.000 | 2503 | tags=30%, list=12%, signal=33% |
| 566 | GO\_VIRAL\_GENOME\_REPLICATION |  | 19 | 0.49 | 1.36 | 0.112 | 0.313 | 1.000 | 3080 | tags=37%, list=14%, signal=43% |
| 567 | GO\_REGULATION\_OF\_CELLULAR\_RESPONSE\_TO\_TRANSFORMING\_GROWTH\_FACTOR\_BETA\_STIMULUS |  | 95 | 0.48 | 1.36 | 0.086 | 0.312 | 1.000 | 3619 | tags=27%, list=17%, signal=33% |
| 568 | GO\_POTASSIUM\_ION\_HOMEOSTASIS |  | 17 | 0.54 | 1.36 | 0.116 | 0.312 | 1.000 | 2693 | tags=18%, list=12%, signal=20% |
| 569 | GO\_NEGATIVE\_REGULATION\_OF\_SECRETION |  | 188 | 0.42 | 1.36 | 0.008 | 0.313 | 1.000 | 3987 | tags=31%, list=18%, signal=37% |
| 570 | GO\_CELL\_COMMUNICATION\_INVOLVED\_IN\_CARDIAC\_CONDUCTION |  | 35 | 0.52 | 1.36 | 0.092 | 0.315 | 1.000 | 3039 | tags=29%, list=14%, signal=33% |
| 571 | GO\_ARTERY\_MORPHOGENESIS |  | 51 | 0.52 | 1.36 | 0.106 | 0.315 | 1.000 | 2956 | tags=39%, list=14%, signal=45% |
| 572 | GO\_ACTIN\_MYOSIN\_FILAMENT\_SLIDING |  | 38 | 0.90 | 1.36 | 0.050 | 0.314 | 1.000 | 1557 | tags=82%, list=7%, signal=88% |
| 573 | GO\_REGULATION\_OF\_LEUKOCYTE\_APOPTOTIC\_PROCESS |  | 74 | 0.57 | 1.36 | 0.077 | 0.314 | 1.000 | 3222 | tags=46%, list=15%, signal=54% |
| 574 | GO\_MUCOPOLYSACCHARIDE\_METABOLIC\_PROCESS |  | 104 | 0.49 | 1.36 | 0.070 | 0.314 | 1.000 | 3802 | tags=37%, list=17%, signal=44% |
| 575 | GO\_ADHERENS\_JUNCTION\_ASSEMBLY |  | 33 | 0.52 | 1.36 | 0.110 | 0.313 | 1.000 | 1350 | tags=24%, list=6%, signal=26% |
| 576 | GO\_REGULATION\_OF\_VOLTAGE\_GATED\_CALCIUM\_CHANNEL\_ACTIVITY |  | 23 | 0.54 | 1.36 | 0.111 | 0.313 | 1.000 | 2747 | tags=26%, list=13%, signal=30% |
| 577 | GO\_REGULATION\_OF\_FAT\_CELL\_DIFFERENTIATION |  | 97 | 0.46 | 1.36 | 0.034 | 0.313 | 1.000 | 2811 | tags=27%, list=13%, signal=31% |
| 578 | GO\_NEGATIVE\_REGULATION\_OF\_CELL\_ADHESION |  | 213 | 0.44 | 1.36 | 0.024 | 0.313 | 1.000 | 2993 | tags=28%, list=14%, signal=32% |
| 579 | GO\_NEGATIVE\_REGULATION\_OF\_NF\_KAPPAB\_IMPORT\_INTO\_NUCLEUS |  | 16 | 0.52 | 1.36 | 0.089 | 0.313 | 1.000 | 4236 | tags=38%, list=19%, signal=47% |
| 580 | GO\_REGULATION\_OF\_NEURON\_PROJECTION\_DEVELOPMENT |  | 391 | 0.40 | 1.36 | 0.036 | 0.316 | 1.000 | 3697 | tags=28%, list=17%, signal=34% |
| 581 | GO\_PROTEIN\_SECRETION |  | 108 | 0.45 | 1.36 | 0.040 | 0.316 | 1.000 | 2607 | tags=22%, list=12%, signal=25% |
| 582 | GO\_CYTOKINE\_PRODUCTION |  | 116 | 0.54 | 1.36 | 0.084 | 0.315 | 1.000 | 2920 | tags=35%, list=13%, signal=41% |
| 583 | GO\_VENTRICULAR\_SEPTUM\_DEVELOPMENT |  | 54 | 0.54 | 1.36 | 0.095 | 0.315 | 1.000 | 3058 | tags=30%, list=14%, signal=34% |
| 584 | GO\_GOLGI\_TO\_ENDOSOME\_TRANSPORT |  | 17 | 0.52 | 1.36 | 0.092 | 0.316 | 1.000 | 4443 | tags=59%, list=20%, signal=74% |
| 585 | GO\_REGULATION\_OF\_LIPID\_METABOLIC\_PROCESS |  | 260 | 0.40 | 1.36 | 0.010 | 0.315 | 1.000 | 3284 | tags=24%, list=15%, signal=28% |
| 586 | GO\_POSITIVE\_REGULATION\_OF\_CELL\_ACTIVATION |  | 285 | 0.54 | 1.36 | 0.113 | 0.316 | 1.000 | 3284 | tags=40%, list=15%, signal=47% |
| 587 | GO\_REGULATION\_OF\_PROTEIN\_TARGETING |  | 279 | 0.37 | 1.36 | 0.021 | 0.315 | 1.000 | 3274 | tags=22%, list=15%, signal=26% |
| 588 | GO\_CARDIAC\_MUSCLE\_CELL\_CONTRACTION |  | 28 | 0.57 | 1.36 | 0.073 | 0.315 | 1.000 | 3039 | tags=39%, list=14%, signal=46% |
| 589 | GO\_POSITIVE\_REGULATION\_OF\_BMP\_SIGNALING\_PATHWAY |  | 32 | 0.55 | 1.36 | 0.094 | 0.314 | 1.000 | 2956 | tags=34%, list=14%, signal=40% |
| 590 | GO\_CELL\_MORPHOGENESIS\_INVOLVED\_IN\_DIFFERENTIATION |  | 488 | 0.42 | 1.36 | 0.044 | 0.314 | 1.000 | 3353 | tags=23%, list=15%, signal=27% |
| 591 | GO\_ENDOCARDIAL\_CUSHION\_MORPHOGENESIS |  | 22 | 0.65 | 1.36 | 0.115 | 0.313 | 1.000 | 2430 | tags=41%, list=11%, signal=46% |
| 592 | GO\_G\_PROTEIN\_COUPLED\_RECEPTOR\_SIGNALING\_PATHWAY\_COUPLED\_TO\_CYCLIC\_NUCLEOTIDE\_SECOND\_MESSENGER |  | 159 | 0.37 | 1.36 | 0.030 | 0.313 | 1.000 | 3584 | tags=20%, list=16%, signal=24% |
| 593 | GO\_ORGANONITROGEN\_COMPOUND\_CATABOLIC\_PROCESS |  | 318 | 0.39 | 1.36 | 0.008 | 0.313 | 1.000 | 3379 | tags=24%, list=16%, signal=28% |
| 594 | GO\_RESPONSE\_TO\_PEPTIDE |  | 388 | 0.36 | 1.36 | 0.015 | 0.313 | 1.000 | 2504 | tags=17%, list=12%, signal=19% |
| 595 | GO\_REGULATION\_OF\_MYOBLAST\_FUSION |  | 19 | 0.63 | 1.36 | 0.066 | 0.313 | 1.000 | 3531 | tags=53%, list=16%, signal=63% |
| 596 | GO\_PARTURITION |  | 20 | 0.59 | 1.36 | 0.063 | 0.313 | 1.000 | 3691 | tags=45%, list=17%, signal=54% |
| 597 | GO\_EMBRYONIC\_DIGESTIVE\_TRACT\_DEVELOPMENT |  | 32 | 0.60 | 1.36 | 0.093 | 0.313 | 1.000 | 1829 | tags=28%, list=8%, signal=31% |
| 598 | GO\_POSITIVE\_REGULATION\_OF\_BEHAVIOR |  | 23 | 0.54 | 1.36 | 0.123 | 0.312 | 1.000 | 2646 | tags=17%, list=12%, signal=20% |
| 599 | GO\_REGULATION\_OF\_INTRACELLULAR\_PROTEIN\_TRANSPORT |  | 348 | 0.36 | 1.36 | 0.015 | 0.312 | 1.000 | 3305 | tags=21%, list=15%, signal=25% |
| 600 | GO\_REGULATION\_OF\_SODIUM\_ION\_TRANSPORT |  | 74 | 0.46 | 1.36 | 0.070 | 0.312 | 1.000 | 1556 | tags=16%, list=7%, signal=17% |
| 601 | GO\_NEGATIVE\_REGULATION\_OF\_CELL\_ACTIVATION |  | 150 | 0.51 | 1.36 | 0.082 | 0.312 | 1.000 | 3472 | tags=38%, list=16%, signal=45% |
| 602 | GO\_REGULATION\_OF\_LIPID\_CATABOLIC\_PROCESS |  | 48 | 0.46 | 1.36 | 0.074 | 0.313 | 1.000 | 2477 | tags=21%, list=11%, signal=23% |
| 603 | GO\_POSITIVE\_REGULATION\_OF\_TYROSINE\_PHOSPHORYLATION\_OF\_STAT5\_PROTEIN |  | 16 | 0.64 | 1.35 | 0.084 | 0.314 | 1.000 | 1755 | tags=31%, list=8%, signal=34% |
| 604 | GO\_POSITIVE\_REGULATION\_OF\_SODIUM\_ION\_TRANSMEMBRANE\_TRANSPORT |  | 16 | 0.57 | 1.35 | 0.111 | 0.314 | 1.000 | 2462 | tags=25%, list=11%, signal=28% |
| 605 | GO\_B\_CELL\_DIFFERENTIATION |  | 85 | 0.46 | 1.35 | 0.102 | 0.314 | 1.000 | 3448 | tags=33%, list=16%, signal=39% |
| 606 | GO\_REGULATION\_OF\_NUCLEOCYTOPLASMIC\_TRANSPORT |  | 210 | 0.40 | 1.35 | 0.023 | 0.315 | 1.000 | 3241 | tags=23%, list=15%, signal=27% |
| 607 | GO\_PHAGOCYTOSIS\_ENGULFMENT |  | 26 | 0.70 | 1.35 | 0.129 | 0.315 | 1.000 | 3208 | tags=54%, list=15%, signal=63% |
| 608 | GO\_NEURON\_MIGRATION |  | 102 | 0.45 | 1.35 | 0.063 | 0.315 | 1.000 | 2966 | tags=24%, list=14%, signal=27% |
| 609 | GO\_NEGATIVE\_REGULATION\_OF\_INTRACELLULAR\_SIGNAL\_TRANSDUCTION |  | 406 | 0.37 | 1.35 | 0.012 | 0.315 | 1.000 | 3241 | tags=22%, list=15%, signal=26% |
| 610 | GO\_ADENYLATE\_CYCLASE\_ACTIVATING\_G\_PROTEIN\_COUPLED\_RECEPTOR\_SIGNALING\_PATHWAY |  | 65 | 0.43 | 1.35 | 0.073 | 0.316 | 1.000 | 3584 | tags=22%, list=16%, signal=26% |
| 611 | GO\_POSITIVE\_REGULATION\_OF\_CYCLIC\_NUCLEOTIDE\_METABOLIC\_PROCESS |  | 102 | 0.43 | 1.35 | 0.066 | 0.316 | 1.000 | 3691 | tags=27%, list=17%, signal=33% |
| 612 | GO\_REGULATION\_OF\_PROTEIN\_MATURATION |  | 70 | 0.46 | 1.35 | 0.063 | 0.317 | 1.000 | 3217 | tags=31%, list=15%, signal=37% |
| 613 | GO\_OLIGOSACCHARIDE\_METABOLIC\_PROCESS |  | 63 | 0.48 | 1.35 | 0.076 | 0.318 | 1.000 | 4360 | tags=40%, list=20%, signal=49% |
| 614 | GO\_SKELETAL\_SYSTEM\_DEVELOPMENT |  | 440 | 0.44 | 1.35 | 0.058 | 0.318 | 1.000 | 3066 | tags=26%, list=14%, signal=30% |
| 615 | GO\_REGULATION\_OF\_OSTEOCLAST\_DIFFERENTIATION |  | 58 | 0.52 | 1.35 | 0.087 | 0.320 | 1.000 | 3679 | tags=36%, list=17%, signal=43% |
| 616 | GO\_POSITIVE\_REGULATION\_OF\_CALCIUM\_ION\_IMPORT |  | 48 | 0.51 | 1.35 | 0.083 | 0.320 | 1.000 | 3080 | tags=33%, list=14%, signal=39% |
| 617 | GO\_REGULATION\_OF\_MYELOID\_CELL\_DIFFERENTIATION |  | 168 | 0.41 | 1.35 | 0.063 | 0.319 | 1.000 | 3582 | tags=26%, list=16%, signal=31% |
| 618 | GO\_REGULATION\_OF\_LIPOPOLYSACCHARIDE\_MEDIATED\_SIGNALING\_PATHWAY |  | 17 | 0.57 | 1.35 | 0.098 | 0.319 | 1.000 | 3370 | tags=41%, list=15%, signal=49% |
| 619 | GO\_CARDIAC\_CELL\_DEVELOPMENT |  | 47 | 0.62 | 1.35 | 0.126 | 0.319 | 1.000 | 2344 | tags=38%, list=11%, signal=43% |
| 620 | GO\_NEGATIVE\_REGULATION\_OF\_POTASSIUM\_ION\_TRANSPORT |  | 31 | 0.52 | 1.35 | 0.072 | 0.318 | 1.000 | 3962 | tags=35%, list=18%, signal=43% |
| 621 | GO\_TRANSFORMING\_GROWTH\_FACTOR\_BETA\_RECEPTOR\_SIGNALING\_PATHWAY |  | 92 | 0.42 | 1.35 | 0.057 | 0.320 | 1.000 | 2230 | tags=21%, list=10%, signal=23% |
| 622 | GO\_REGULATION\_OF\_RESPONSE\_TO\_WOUNDING |  | 388 | 0.45 | 1.35 | 0.051 | 0.319 | 1.000 | 3241 | tags=30%, list=15%, signal=35% |
| 623 | GO\_REGULATION\_OF\_CHOLESTEROL\_EFFLUX |  | 19 | 0.56 | 1.35 | 0.091 | 0.319 | 1.000 | 4567 | tags=47%, list=21%, signal=60% |
| 624 | GO\_CAMP\_MEDIATED\_SIGNALING |  | 36 | 0.48 | 1.35 | 0.063 | 0.320 | 1.000 | 2670 | tags=22%, list=12%, signal=25% |
| 625 | GO\_REGULATION\_OF\_BINDING |  | 275 | 0.36 | 1.35 | 0.024 | 0.320 | 1.000 | 3406 | tags=24%, list=16%, signal=29% |
| 626 | GO\_METANEPHROS\_DEVELOPMENT |  | 80 | 0.48 | 1.35 | 0.054 | 0.320 | 1.000 | 3267 | tags=31%, list=15%, signal=37% |
| 627 | GO\_EPITHELIAL\_CELL\_DIFFERENTIATION\_INVOLVED\_IN\_KIDNEY\_DEVELOPMENT |  | 24 | 0.55 | 1.35 | 0.091 | 0.320 | 1.000 | 3019 | tags=33%, list=14%, signal=39% |
| 628 | GO\_NEGATIVE\_REGULATION\_OF\_PHOSPHORYLATION |  | 397 | 0.38 | 1.34 | 0.012 | 0.320 | 1.000 | 3290 | tags=24%, list=15%, signal=27% |
| 629 | GO\_REGULATION\_OF\_CARDIAC\_MUSCLE\_CELL\_CONTRACTION |  | 27 | 0.51 | 1.34 | 0.083 | 0.319 | 1.000 | 2028 | tags=26%, list=9%, signal=29% |
| 630 | GO\_ERK1\_AND\_ERK2\_CASCADE |  | 22 | 0.57 | 1.34 | 0.098 | 0.320 | 1.000 | 4288 | tags=45%, list=20%, signal=57% |
| 631 | GO\_NEGATIVE\_REGULATION\_OF\_AMINE\_TRANSPORT |  | 24 | 0.53 | 1.34 | 0.077 | 0.321 | 1.000 | 1872 | tags=21%, list=9%, signal=23% |
| 632 | GO\_REGULATION\_OF\_MUSCLE\_CELL\_APOPTOTIC\_PROCESS |  | 41 | 0.50 | 1.34 | 0.049 | 0.321 | 1.000 | 3290 | tags=39%, list=15%, signal=46% |
| 633 | GO\_LEUKOCYTE\_DIFFERENTIATION |  | 285 | 0.49 | 1.34 | 0.103 | 0.321 | 1.000 | 3526 | tags=38%, list=16%, signal=44% |
| 634 | GO\_HUMORAL\_IMMUNE\_RESPONSE |  | 151 | 0.50 | 1.34 | 0.084 | 0.321 | 1.000 | 3217 | tags=36%, list=15%, signal=42% |
| 635 | GO\_REGULATION\_OF\_NEUROTRANSMITTER\_TRANSPORT |  | 58 | 0.47 | 1.34 | 0.079 | 0.323 | 1.000 | 1810 | tags=16%, list=8%, signal=17% |
| 636 | GO\_CELL\_CELL\_RECOGNITION |  | 53 | 0.48 | 1.34 | 0.139 | 0.322 | 1.000 | 3129 | tags=23%, list=14%, signal=26% |
| 637 | GO\_REGULATION\_OF\_AXON\_GUIDANCE |  | 37 | 0.53 | 1.34 | 0.105 | 0.324 | 1.000 | 1352 | tags=19%, list=6%, signal=20% |
| 638 | GO\_DETECTION\_OF\_MECHANICAL\_STIMULUS\_INVOLVED\_IN\_SENSORY\_PERCEPTION |  | 22 | 0.56 | 1.34 | 0.105 | 0.325 | 1.000 | 1955 | tags=18%, list=9%, signal=20% |
| 639 | GO\_TRANSMISSION\_OF\_NERVE\_IMPULSE |  | 49 | 0.47 | 1.34 | 0.082 | 0.325 | 1.000 | 2441 | tags=18%, list=11%, signal=21% |
| 640 | GO\_NEGATIVE\_REGULATION\_OF\_KINASE\_ACTIVITY |  | 237 | 0.37 | 1.34 | 0.016 | 0.327 | 1.000 | 3290 | tags=22%, list=15%, signal=26% |
| 641 | GO\_LYSOSOME\_LOCALIZATION |  | 22 | 0.56 | 1.34 | 0.122 | 0.327 | 1.000 | 2920 | tags=27%, list=13%, signal=31% |
| 642 | GO\_ENDOPLASMIC\_RETICULUM\_TO\_CYTOSOL\_TRANSPORT |  | 20 | 0.53 | 1.34 | 0.145 | 0.328 | 1.000 | 2127 | tags=20%, list=10%, signal=22% |
| 643 | GO\_NEURON\_PROJECTION\_MORPHOGENESIS |  | 373 | 0.41 | 1.34 | 0.055 | 0.328 | 1.000 | 2763 | tags=18%, list=13%, signal=21% |
| 644 | GO\_AGING |  | 257 | 0.39 | 1.34 | 0.029 | 0.331 | 1.000 | 2231 | tags=19%, list=10%, signal=21% |
| 645 | GO\_REGULATION\_OF\_EPITHELIAL\_CELL\_DIFFERENTIATION\_INVOLVED\_IN\_KIDNEY\_DEVELOPMENT |  | 15 | 0.62 | 1.34 | 0.081 | 0.330 | 1.000 | 2020 | tags=27%, list=9%, signal=29% |
| 646 | GO\_AUDITORY\_RECEPTOR\_CELL\_DEVELOPMENT |  | 17 | 0.46 | 1.33 | 0.105 | 0.331 | 1.000 | 1437 | tags=12%, list=7%, signal=13% |
| 647 | GO\_T\_CELL\_SELECTION |  | 36 | 0.64 | 1.33 | 0.160 | 0.331 | 1.000 | 3526 | tags=50%, list=16%, signal=60% |
| 648 | GO\_POSITIVE\_REGULATION\_OF\_MUSCLE\_HYPERTROPHY |  | 21 | 0.51 | 1.33 | 0.111 | 0.330 | 1.000 | 125 | tags=10%, list=1%, signal=10% |
| 649 | GO\_POSITIVE\_REGULATION\_OF\_SKELETAL\_MUSCLE\_TISSUE\_DEVELOPMENT |  | 25 | 0.60 | 1.33 | 0.108 | 0.331 | 1.000 | 3250 | tags=32%, list=15%, signal=38% |
| 650 | GO\_REGULATION\_OF\_AUTOPHAGY |  | 239 | 0.32 | 1.33 | 0.037 | 0.331 | 1.000 | 4492 | tags=30%, list=21%, signal=37% |
| 651 | GO\_MESONEPHROS\_DEVELOPMENT |  | 89 | 0.47 | 1.33 | 0.059 | 0.331 | 1.000 | 2187 | tags=24%, list=10%, signal=26% |
| 652 | GO\_POSITIVE\_REGULATION\_OF\_CAMP\_METABOLIC\_PROCESS |  | 83 | 0.43 | 1.33 | 0.072 | 0.331 | 1.000 | 3691 | tags=28%, list=17%, signal=33% |
| 653 | GO\_POSITIVE\_REGULATION\_OF\_ENDOTHELIAL\_CELL\_PROLIFERATION |  | 65 | 0.51 | 1.33 | 0.079 | 0.331 | 1.000 | 3967 | tags=45%, list=18%, signal=54% |
| 654 | GO\_POSITIVE\_REGULATION\_OF\_POTASSIUM\_ION\_TRANSMEMBRANE\_TRANSPORT |  | 26 | 0.45 | 1.33 | 0.100 | 0.330 | 1.000 | 3562 | tags=27%, list=16%, signal=32% |
| 655 | GO\_RESPONSE\_TO\_MECHANICAL\_STIMULUS |  | 201 | 0.43 | 1.33 | 0.059 | 0.331 | 1.000 | 3309 | tags=28%, list=15%, signal=33% |
| 656 | GO\_RESPONSE\_TO\_AMINO\_ACID |  | 107 | 0.42 | 1.33 | 0.059 | 0.331 | 1.000 | 2682 | tags=26%, list=12%, signal=30% |
| 657 | GO\_PEPTIDYL\_TYROSINE\_MODIFICATION |  | 183 | 0.40 | 1.33 | 0.036 | 0.331 | 1.000 | 3880 | tags=32%, list=18%, signal=38% |
| 658 | GO\_POSITIVE\_REGULATION\_OF\_NEURON\_PROJECTION\_DEVELOPMENT |  | 224 | 0.40 | 1.33 | 0.052 | 0.331 | 1.000 | 3681 | tags=29%, list=17%, signal=35% |
| 659 | GO\_ALPHA\_BETA\_T\_CELL\_DIFFERENTIATION |  | 44 | 0.63 | 1.33 | 0.129 | 0.331 | 1.000 | 3526 | tags=50%, list=16%, signal=60% |
| 660 | GO\_VACUOLAR\_ACIDIFICATION |  | 15 | 0.53 | 1.33 | 0.121 | 0.331 | 1.000 | 4406 | tags=33%, list=20%, signal=42% |
| 661 | GO\_VENTRICULAR\_CARDIAC\_MUSCLE\_TISSUE\_DEVELOPMENT |  | 43 | 0.59 | 1.33 | 0.081 | 0.331 | 1.000 | 2116 | tags=35%, list=10%, signal=39% |
| 662 | GO\_NEGATIVE\_REGULATION\_OF\_HEMOPOIESIS |  | 117 | 0.40 | 1.33 | 0.062 | 0.331 | 1.000 | 3783 | tags=27%, list=17%, signal=33% |
| 663 | GO\_LEUKOCYTE\_ACTIVATION |  | 399 | 0.53 | 1.33 | 0.121 | 0.331 | 1.000 | 3196 | tags=37%, list=15%, signal=43% |
| 664 | GO\_CARDIAC\_MUSCLE\_CELL\_DIFFERENTIATION |  | 71 | 0.55 | 1.33 | 0.117 | 0.331 | 1.000 | 3586 | tags=41%, list=16%, signal=49% |
| 665 | GO\_POSITIVE\_REGULATION\_OF\_ADENYLATE\_CYCLASE\_ACTIVITY |  | 43 | 0.45 | 1.33 | 0.114 | 0.331 | 1.000 | 3691 | tags=30%, list=17%, signal=36% |
| 666 | GO\_POSITIVE\_REGULATION\_OF\_ERYTHROCYTE\_DIFFERENTIATION |  | 23 | 0.48 | 1.33 | 0.125 | 0.330 | 1.000 | 3049 | tags=30%, list=14%, signal=35% |
| 667 | GO\_OVULATION\_CYCLE |  | 109 | 0.44 | 1.33 | 0.082 | 0.332 | 1.000 | 2956 | tags=26%, list=14%, signal=30% |
| 668 | GO\_RAC\_PROTEIN\_SIGNAL\_TRANSDUCTION |  | 17 | 0.54 | 1.33 | 0.130 | 0.333 | 1.000 | 2100 | tags=29%, list=10%, signal=33% |
| 669 | GO\_T\_CELL\_DIFFERENTIATION |  | 120 | 0.54 | 1.33 | 0.125 | 0.332 | 1.000 | 3582 | tags=44%, list=16%, signal=53% |
| 670 | GO\_REGULATION\_OF\_LIPID\_STORAGE |  | 37 | 0.49 | 1.33 | 0.090 | 0.333 | 1.000 | 1165 | tags=19%, list=5%, signal=20% |
| 671 | GO\_POSITIVE\_REGULATION\_OF\_SODIUM\_ION\_TRANSPORT |  | 32 | 0.51 | 1.33 | 0.081 | 0.333 | 1.000 | 3058 | tags=25%, list=14%, signal=29% |
| 672 | GO\_WOUND\_HEALING |  | 441 | 0.40 | 1.33 | 0.065 | 0.333 | 1.000 | 3035 | tags=25%, list=14%, signal=28% |
| 673 | GO\_AMINO\_SUGAR\_METABOLIC\_PROCESS |  | 38 | 0.52 | 1.33 | 0.102 | 0.332 | 1.000 | 4937 | tags=55%, list=23%, signal=71% |
| 674 | GO\_POSITIVE\_REGULATION\_OF\_NEURON\_DIFFERENTIATION |  | 291 | 0.40 | 1.33 | 0.055 | 0.332 | 1.000 | 3785 | tags=31%, list=17%, signal=37% |
| 675 | GO\_NEUTRAL\_LIPID\_METABOLIC\_PROCESS |  | 78 | 0.45 | 1.33 | 0.053 | 0.332 | 1.000 | 2784 | tags=24%, list=13%, signal=28% |
| 676 | GO\_REGULATION\_OF\_TYROSINE\_PHOSPHORYLATION\_OF\_STAT5\_PROTEIN |  | 20 | 0.58 | 1.33 | 0.096 | 0.332 | 1.000 | 1755 | tags=25%, list=8%, signal=27% |
| 677 | GO\_CONNECTIVE\_TISSUE\_DEVELOPMENT |  | 187 | 0.48 | 1.33 | 0.067 | 0.332 | 1.000 | 3826 | tags=37%, list=18%, signal=44% |
| 678 | GO\_ALPHA\_BETA\_T\_CELL\_ACTIVATION |  | 52 | 0.61 | 1.33 | 0.135 | 0.333 | 1.000 | 3526 | tags=46%, list=16%, signal=55% |
| 679 | GO\_POSITIVE\_REGULATION\_OF\_SMOOTHENED\_SIGNALING\_PATHWAY |  | 24 | 0.63 | 1.33 | 0.122 | 0.332 | 1.000 | 2779 | tags=38%, list=13%, signal=43% |
| 680 | GO\_NEGATIVE\_REGULATION\_OF\_LEUKOCYTE\_DIFFERENTIATION |  | 78 | 0.44 | 1.33 | 0.110 | 0.332 | 1.000 | 3783 | tags=31%, list=17%, signal=37% |
| 681 | GO\_CELLULAR\_RESPONSE\_TO\_VITAMIN |  | 25 | 0.59 | 1.33 | 0.073 | 0.333 | 1.000 | 2912 | tags=44%, list=13%, signal=51% |
| 682 | GO\_NEUROMUSCULAR\_SYNAPTIC\_TRANSMISSION |  | 25 | 0.58 | 1.32 | 0.133 | 0.333 | 1.000 | 4196 | tags=36%, list=19%, signal=45% |
| 683 | GO\_REGULATION\_OF\_CYTOKINE\_PRODUCTION\_INVOLVED\_IN\_IMMUNE\_RESPONSE |  | 55 | 0.57 | 1.32 | 0.111 | 0.333 | 1.000 | 2428 | tags=36%, list=11%, signal=41% |
| 684 | GO\_PURINERGIC\_NUCLEOTIDE\_RECEPTOR\_SIGNALING\_PATHWAY |  | 21 | 0.71 | 1.32 | 0.148 | 0.333 | 1.000 | 2142 | tags=48%, list=10%, signal=53% |
| 685 | GO\_VASCULATURE\_DEVELOPMENT |  | 452 | 0.44 | 1.32 | 0.073 | 0.333 | 1.000 | 3298 | tags=31%, list=15%, signal=35% |
| 686 | GO\_REGULATION\_OF\_SENSORY\_PERCEPTION |  | 33 | 0.51 | 1.32 | 0.072 | 0.333 | 1.000 | 3663 | tags=33%, list=17%, signal=40% |
| 687 | GO\_AMINOGLYCAN\_METABOLIC\_PROCESS |  | 161 | 0.46 | 1.32 | 0.095 | 0.333 | 1.000 | 3802 | tags=35%, list=17%, signal=42% |
| 688 | GO\_NEGATIVE\_REGULATION\_OF\_PEPTIDE\_SECRETION |  | 48 | 0.45 | 1.32 | 0.072 | 0.333 | 1.000 | 2796 | tags=21%, list=13%, signal=24% |
| 689 | GO\_REGULATION\_OF\_CELL\_PROJECTION\_ASSEMBLY |  | 141 | 0.38 | 1.32 | 0.033 | 0.335 | 1.000 | 3149 | tags=22%, list=14%, signal=26% |
| 690 | GO\_POSITIVE\_REGULATION\_OF\_VASCULATURE\_DEVELOPMENT |  | 125 | 0.50 | 1.32 | 0.071 | 0.334 | 1.000 | 3370 | tags=41%, list=15%, signal=48% |
| 691 | GO\_REGULATION\_OF\_SYNAPTIC\_TRANSMISSION\_GLUTAMATERGIC |  | 48 | 0.47 | 1.32 | 0.103 | 0.334 | 1.000 | 1435 | tags=13%, list=7%, signal=13% |
| 692 | GO\_BLOOD\_VESSEL\_MORPHOGENESIS |  | 351 | 0.44 | 1.32 | 0.091 | 0.334 | 1.000 | 3298 | tags=32%, list=15%, signal=37% |
| 693 | GO\_RUFFLE\_ORGANIZATION |  | 19 | 0.56 | 1.32 | 0.099 | 0.333 | 1.000 | 2498 | tags=37%, list=11%, signal=42% |
| 694 | GO\_REGULATION\_OF\_PROTEIN\_LOCALIZATION\_TO\_NUCLEUS |  | 206 | 0.39 | 1.32 | 0.053 | 0.333 | 1.000 | 3241 | tags=23%, list=15%, signal=27% |
| 695 | GO\_CELLULAR\_DEFENSE\_RESPONSE |  | 56 | 0.61 | 1.32 | 0.141 | 0.335 | 1.000 | 3508 | tags=48%, list=16%, signal=57% |
| 696 | GO\_DENDRITIC\_CELL\_CHEMOTAXIS |  | 16 | 0.78 | 1.32 | 0.126 | 0.336 | 1.000 | 2170 | tags=63%, list=10%, signal=69% |
| 697 | GO\_NEGATIVE\_REGULATION\_OF\_SMALL\_GTPASE\_MEDIATED\_SIGNAL\_TRANSDUCTION |  | 39 | 0.45 | 1.32 | 0.085 | 0.336 | 1.000 | 3447 | tags=31%, list=16%, signal=36% |
| 698 | GO\_NEGATIVE\_REGULATION\_OF\_LIPID\_CATABOLIC\_PROCESS |  | 18 | 0.59 | 1.32 | 0.112 | 0.335 | 1.000 | 1516 | tags=28%, list=7%, signal=30% |
| 699 | GO\_POSITIVE\_REGULATION\_OF\_COAGULATION |  | 24 | 0.57 | 1.32 | 0.108 | 0.335 | 1.000 | 442 | tags=17%, list=2%, signal=17% |
| 700 | GO\_REGULATION\_OF\_MONOCYTE\_CHEMOTAXIS |  | 20 | 0.69 | 1.32 | 0.127 | 0.335 | 1.000 | 3138 | tags=55%, list=14%, signal=64% |
| 701 | GO\_NEGATIVE\_REGULATION\_OF\_NEURON\_DIFFERENTIATION |  | 182 | 0.40 | 1.32 | 0.051 | 0.335 | 1.000 | 2898 | tags=20%, list=13%, signal=23% |
| 702 | GO\_REGULATION\_OF\_EMBRYONIC\_DEVELOPMENT |  | 107 | 0.45 | 1.32 | 0.088 | 0.335 | 1.000 | 2074 | tags=21%, list=10%, signal=23% |
| 703 | GO\_RAS\_PROTEIN\_SIGNAL\_TRANSDUCTION |  | 136 | 0.37 | 1.32 | 0.047 | 0.335 | 1.000 | 3580 | tags=28%, list=16%, signal=33% |
| 704 | GO\_LEUKOCYTE\_CELL\_CELL\_ADHESION |  | 248 | 0.53 | 1.32 | 0.144 | 0.335 | 1.000 | 3163 | tags=36%, list=15%, signal=42% |
| 705 | GO\_ADHERENS\_JUNCTION\_ORGANIZATION |  | 64 | 0.46 | 1.32 | 0.104 | 0.335 | 1.000 | 1350 | tags=19%, list=6%, signal=20% |
| 706 | GO\_REGULATION\_OF\_SMOOTH\_MUSCLE\_CELL\_PROLIFERATION |  | 98 | 0.44 | 1.32 | 0.030 | 0.335 | 1.000 | 1924 | tags=20%, list=9%, signal=22% |
| 707 | GO\_REGULATION\_OF\_CELLULAR\_RESPONSE\_TO\_INSULIN\_STIMULUS |  | 55 | 0.43 | 1.32 | 0.072 | 0.335 | 1.000 | 3755 | tags=35%, list=17%, signal=42% |
| 708 | GO\_CELLULAR\_RESPONSE\_TO\_NUTRIENT |  | 38 | 0.52 | 1.32 | 0.064 | 0.335 | 1.000 | 3166 | tags=37%, list=15%, signal=43% |
| 709 | GO\_NEGATIVE\_REGULATION\_OF\_PRODUCTION\_OF\_MOLECULAR\_MEDIATOR\_OF\_IMMUNE\_RESPONSE |  | 29 | 0.58 | 1.32 | 0.126 | 0.335 | 1.000 | 3473 | tags=48%, list=16%, signal=57% |
| 710 | GO\_T\_CELL\_DIFFERENTIATION\_INVOLVED\_IN\_IMMUNE\_RESPONSE |  | 28 | 0.63 | 1.32 | 0.155 | 0.335 | 1.000 | 3406 | tags=57%, list=16%, signal=68% |
| 711 | GO\_POSITIVE\_REGULATION\_OF\_RECEPTOR\_ACTIVITY |  | 41 | 0.46 | 1.32 | 0.106 | 0.335 | 1.000 | 3138 | tags=24%, list=14%, signal=28% |
| 712 | GO\_MATING |  | 36 | 0.46 | 1.32 | 0.122 | 0.336 | 1.000 | 4354 | tags=28%, list=20%, signal=35% |
| 713 | GO\_UROGENITAL\_SYSTEM\_DEVELOPMENT |  | 293 | 0.43 | 1.32 | 0.073 | 0.336 | 1.000 | 3032 | tags=27%, list=14%, signal=31% |
| 714 | GO\_REGULATION\_OF\_RESPIRATORY\_SYSTEM\_PROCESS |  | 15 | 0.60 | 1.32 | 0.159 | 0.336 | 1.000 | 3823 | tags=33%, list=18%, signal=40% |
| 715 | GO\_PALATE\_DEVELOPMENT |  | 83 | 0.48 | 1.32 | 0.102 | 0.337 | 1.000 | 2063 | tags=23%, list=9%, signal=25% |
| 716 | GO\_NEGATIVE\_REGULATION\_OF\_TISSUE\_REMODELING |  | 17 | 0.59 | 1.31 | 0.103 | 0.337 | 1.000 | 2031 | tags=29%, list=9%, signal=32% |
| 717 | GO\_NEGATIVE\_REGULATION\_OF\_AXON\_GUIDANCE |  | 25 | 0.54 | 1.31 | 0.124 | 0.336 | 1.000 | 878 | tags=16%, list=4%, signal=17% |
| 718 | GO\_NEGATIVE\_REGULATION\_OF\_CELLULAR\_RESPONSE\_TO\_INSULIN\_STIMULUS |  | 31 | 0.48 | 1.31 | 0.098 | 0.337 | 1.000 | 3131 | tags=29%, list=14%, signal=34% |
| 719 | GO\_SECONDARY\_METABOLITE\_BIOSYNTHETIC\_PROCESS |  | 19 | 0.52 | 1.31 | 0.133 | 0.337 | 1.000 | 3018 | tags=21%, list=14%, signal=24% |
| 720 | GO\_LEUKOCYTE\_MEDIATED\_IMMUNITY |  | 154 | 0.52 | 1.31 | 0.141 | 0.337 | 1.000 | 2953 | tags=38%, list=14%, signal=44% |
| 721 | GO\_SEGMENT\_SPECIFICATION |  | 15 | 0.62 | 1.31 | 0.118 | 0.337 | 1.000 | 3582 | tags=47%, list=16%, signal=56% |
| 722 | GO\_SIGNAL\_TRANSDUCTION\_BY\_PROTEIN\_PHOSPHORYLATION |  | 389 | 0.34 | 1.31 | 0.030 | 0.336 | 1.000 | 2795 | tags=18%, list=13%, signal=21% |
| 723 | GO\_REGULATION\_OF\_HOMOTYPIC\_CELL\_CELL\_ADHESION |  | 291 | 0.50 | 1.31 | 0.124 | 0.336 | 1.000 | 3478 | tags=37%, list=16%, signal=44% |
| 724 | GO\_POSITIVE\_REGULATION\_OF\_LEUKOCYTE\_PROLIFERATION |  | 131 | 0.54 | 1.31 | 0.138 | 0.336 | 1.000 | 3196 | tags=37%, list=15%, signal=44% |
| 725 | GO\_NEGATIVE\_REGULATION\_OF\_PROTEIN\_SERINE\_THREONINE\_KINASE\_ACTIVITY |  | 122 | 0.40 | 1.31 | 0.025 | 0.336 | 1.000 | 3290 | tags=26%, list=15%, signal=31% |
| 726 | GO\_NEGATIVE\_REGULATION\_OF\_IMMUNE\_EFFECTOR\_PROCESS |  | 99 | 0.50 | 1.31 | 0.118 | 0.336 | 1.000 | 3473 | tags=33%, list=16%, signal=39% |
| 727 | GO\_POSITIVE\_REGULATION\_OF\_NATURAL\_KILLER\_CELL\_MEDIATED\_IMMUNITY |  | 19 | 0.71 | 1.31 | 0.148 | 0.335 | 1.000 | 1587 | tags=37%, list=7%, signal=40% |
| 728 | GO\_REGULATION\_OF\_CELLULAR\_EXTRAVASATION |  | 22 | 0.62 | 1.31 | 0.111 | 0.336 | 1.000 | 3478 | tags=55%, list=16%, signal=65% |
| 729 | GO\_MODULATION\_OF\_SYNAPTIC\_TRANSMISSION |  | 288 | 0.37 | 1.31 | 0.057 | 0.336 | 1.000 | 3957 | tags=23%, list=18%, signal=28% |
| 730 | GO\_DEVELOPMENTAL\_GROWTH |  | 315 | 0.40 | 1.31 | 0.057 | 0.336 | 1.000 | 2116 | tags=17%, list=10%, signal=18% |
| 731 | GO\_NEGATIVE\_REGULATION\_OF\_NUCLEOSIDE\_METABOLIC\_PROCESS |  | 18 | 0.53 | 1.31 | 0.121 | 0.339 | 1.000 | 3035 | tags=39%, list=14%, signal=45% |
| 732 | GO\_MAST\_CELL\_MEDIATED\_IMMUNITY |  | 17 | 0.63 | 1.31 | 0.153 | 0.339 | 1.000 | 3201 | tags=41%, list=15%, signal=48% |
| 733 | GO\_GLUCAN\_METABOLIC\_PROCESS |  | 54 | 0.48 | 1.31 | 0.145 | 0.339 | 1.000 | 4761 | tags=41%, list=22%, signal=52% |
| 734 | GO\_REGULATION\_OF\_INTERLEUKIN\_12\_PRODUCTION |  | 51 | 0.61 | 1.31 | 0.157 | 0.339 | 1.000 | 3347 | tags=43%, list=15%, signal=51% |
| 735 | GO\_MODULATION\_OF\_GROWTH\_OF\_SYMBIONT\_INVOLVED\_IN\_INTERACTION\_WITH\_HOST |  | 16 | 0.60 | 1.31 | 0.153 | 0.339 | 1.000 | 3084 | tags=38%, list=14%, signal=44% |
| 736 | GO\_NEGATIVE\_REGULATION\_OF\_REACTIVE\_OXYGEN\_SPECIES\_METABOLIC\_PROCESS |  | 40 | 0.40 | 1.31 | 0.076 | 0.339 | 1.000 | 2991 | tags=30%, list=14%, signal=35% |
| 737 | GO\_CELLULAR\_RESPONSE\_TO\_FATTY\_ACID |  | 48 | 0.51 | 1.31 | 0.072 | 0.340 | 1.000 | 2646 | tags=31%, list=12%, signal=35% |
| 738 | GO\_REGULATION\_OF\_VASODILATION |  | 46 | 0.51 | 1.31 | 0.082 | 0.339 | 1.000 | 2102 | tags=26%, list=10%, signal=29% |
| 739 | GO\_TOLL\_LIKE\_RECEPTOR\_SIGNALING\_PATHWAY |  | 83 | 0.53 | 1.31 | 0.157 | 0.340 | 1.000 | 2680 | tags=27%, list=12%, signal=30% |
| 740 | GO\_PHOSPHATIDIC\_ACID\_METABOLIC\_PROCESS |  | 30 | 0.53 | 1.31 | 0.100 | 0.340 | 1.000 | 4675 | tags=43%, list=21%, signal=55% |
| 741 | GO\_REGULATION\_OF\_LEUKOCYTE\_PROLIFERATION |  | 198 | 0.51 | 1.31 | 0.137 | 0.340 | 1.000 | 3526 | tags=39%, list=16%, signal=47% |
| 742 | GO\_JAK\_STAT\_CASCADE\_INVOLVED\_IN\_GROWTH\_HORMONE\_SIGNALING\_PATHWAY |  | 15 | 0.58 | 1.31 | 0.171 | 0.340 | 1.000 | 4204 | tags=40%, list=19%, signal=50% |
| 743 | GO\_REGULATION\_OF\_I\_KAPPAB\_KINASE\_NF\_KAPPAB\_SIGNALING |  | 219 | 0.41 | 1.31 | 0.089 | 0.340 | 1.000 | 3241 | tags=24%, list=15%, signal=28% |
| 744 | GO\_T\_HELPER\_1\_TYPE\_IMMUNE\_RESPONSE |  | 18 | 0.65 | 1.31 | 0.148 | 0.341 | 1.000 | 2863 | tags=44%, list=13%, signal=51% |
| 745 | GO\_NEURAL\_NUCLEUS\_DEVELOPMENT |  | 63 | 0.42 | 1.31 | 0.093 | 0.341 | 1.000 | 2261 | tags=16%, list=10%, signal=18% |
| 746 | GO\_RIBONUCLEOTIDE\_CATABOLIC\_PROCESS |  | 27 | 0.51 | 1.31 | 0.139 | 0.341 | 1.000 | 1962 | tags=22%, list=9%, signal=24% |
| 747 | GO\_POSITIVE\_REGULATION\_OF\_WOUND\_HEALING |  | 45 | 0.49 | 1.31 | 0.108 | 0.341 | 1.000 | 2646 | tags=22%, list=12%, signal=25% |
| 748 | GO\_NEGATIVE\_REGULATION\_OF\_HYDROLASE\_ACTIVITY |  | 362 | 0.37 | 1.31 | 0.030 | 0.341 | 1.000 | 3056 | tags=22%, list=14%, signal=25% |
| 749 | GO\_ANGIOGENESIS |  | 280 | 0.44 | 1.30 | 0.095 | 0.341 | 1.000 | 3298 | tags=32%, list=15%, signal=37% |
| 750 | GO\_REGULATION\_OF\_HEMOPOIESIS |  | 295 | 0.42 | 1.30 | 0.100 | 0.341 | 1.000 | 3582 | tags=31%, list=16%, signal=36% |
| 751 | GO\_NEGATIVE\_REGULATION\_OF\_SEQUENCE\_SPECIFIC\_DNA\_BINDING\_TRANSCRIPTION\_FACTOR\_ACTIVITY |  | 126 | 0.38 | 1.30 | 0.075 | 0.341 | 1.000 | 3241 | tags=20%, list=15%, signal=23% |
| 752 | GO\_ENDOTHELIAL\_CELL\_MIGRATION |  | 53 | 0.49 | 1.30 | 0.121 | 0.342 | 1.000 | 3298 | tags=34%, list=15%, signal=40% |
| 753 | GO\_CARBOHYDRATE\_HOMEOSTASIS |  | 161 | 0.38 | 1.30 | 0.053 | 0.342 | 1.000 | 4065 | tags=29%, list=19%, signal=35% |
| 754 | GO\_CD4\_POSITIVE\_ALPHA\_BETA\_T\_CELL\_ACTIVATION |  | 33 | 0.57 | 1.30 | 0.139 | 0.341 | 1.000 | 3406 | tags=42%, list=16%, signal=50% |
| 755 | GO\_CELLULAR\_SODIUM\_ION\_HOMEOSTASIS |  | 17 | 0.54 | 1.30 | 0.123 | 0.341 | 1.000 | 125 | tags=12%, list=1%, signal=12% |
| 756 | GO\_POSITIVE\_REGULATION\_OF\_BLOOD\_CIRCULATION |  | 92 | 0.43 | 1.30 | 0.037 | 0.341 | 1.000 | 2461 | tags=22%, list=11%, signal=24% |
| 757 | GO\_REGULATION\_OF\_REGULATED\_SECRETORY\_PATHWAY |  | 121 | 0.40 | 1.30 | 0.064 | 0.343 | 1.000 | 3630 | tags=23%, list=17%, signal=28% |
| 758 | GO\_NOTCH\_SIGNALING\_PATHWAY |  | 110 | 0.40 | 1.30 | 0.082 | 0.343 | 1.000 | 2818 | tags=21%, list=13%, signal=24% |
| 759 | GO\_NEUROEPITHELIAL\_CELL\_DIFFERENTIATION |  | 61 | 0.46 | 1.30 | 0.087 | 0.344 | 1.000 | 3404 | tags=26%, list=16%, signal=31% |
| 760 | GO\_T\_CELL\_PROLIFERATION |  | 34 | 0.63 | 1.30 | 0.162 | 0.344 | 1.000 | 2708 | tags=44%, list=12%, signal=50% |
| 761 | GO\_POSITIVE\_REGULATION\_OF\_CELL\_DEVELOPMENT |  | 449 | 0.39 | 1.30 | 0.065 | 0.344 | 1.000 | 3681 | tags=29%, list=17%, signal=34% |
| 762 | GO\_REGULATION\_OF\_NATURAL\_KILLER\_CELL\_MEDIATED\_IMMUNITY |  | 32 | 0.64 | 1.30 | 0.169 | 0.344 | 1.000 | 3474 | tags=44%, list=16%, signal=52% |
| 763 | GO\_REGULATION\_OF\_CAMP\_MEDIATED\_SIGNALING |  | 23 | 0.55 | 1.30 | 0.160 | 0.344 | 1.000 | 4072 | tags=43%, list=19%, signal=53% |
| 764 | GO\_NEGATIVE\_REGULATION\_OF\_TOR\_SIGNALING |  | 30 | 0.42 | 1.30 | 0.117 | 0.344 | 1.000 | 3222 | tags=23%, list=15%, signal=27% |
| 765 | GO\_NEGATIVE\_REGULATION\_OF\_LEUKOCYTE\_MEDIATED\_IMMUNITY |  | 46 | 0.58 | 1.30 | 0.145 | 0.344 | 1.000 | 3457 | tags=39%, list=16%, signal=46% |
| 766 | GO\_MUSCLE\_CELL\_MIGRATION |  | 17 | 0.62 | 1.30 | 0.155 | 0.346 | 1.000 | 1768 | tags=35%, list=8%, signal=38% |
| 767 | GO\_CYTOKINE\_SECRETION |  | 37 | 0.59 | 1.30 | 0.149 | 0.346 | 1.000 | 2413 | tags=38%, list=11%, signal=42% |
| 768 | GO\_REGULATION\_OF\_INTERFERON\_GAMMA\_BIOSYNTHETIC\_PROCESS |  | 16 | 0.61 | 1.30 | 0.167 | 0.346 | 1.000 | 1788 | tags=25%, list=8%, signal=27% |
| 769 | GO\_GROWTH |  | 386 | 0.39 | 1.30 | 0.079 | 0.346 | 1.000 | 3482 | tags=24%, list=16%, signal=28% |
| 770 | GO\_REGULATION\_OF\_POSTSYNAPTIC\_MEMBRANE\_POTENTIAL |  | 54 | 0.45 | 1.30 | 0.090 | 0.346 | 1.000 | 3913 | tags=26%, list=18%, signal=32% |
| 771 | GO\_RESPONSE\_TO\_MUSCLE\_STRETCH |  | 19 | 0.61 | 1.30 | 0.177 | 0.346 | 1.000 | 3293 | tags=42%, list=15%, signal=50% |
| 772 | GO\_GLIAL\_CELL\_DIFFERENTIATION |  | 131 | 0.40 | 1.30 | 0.093 | 0.346 | 1.000 | 3906 | tags=28%, list=18%, signal=34% |
| 773 | GO\_NEGATIVE\_REGULATION\_OF\_REPRODUCTIVE\_PROCESS |  | 47 | 0.48 | 1.30 | 0.061 | 0.347 | 1.000 | 3067 | tags=30%, list=14%, signal=35% |
| 774 | GO\_REGULATION\_OF\_CHONDROCYTE\_DIFFERENTIATION |  | 43 | 0.54 | 1.30 | 0.132 | 0.346 | 1.000 | 3482 | tags=40%, list=16%, signal=47% |
| 775 | GO\_OVULATION\_CYCLE\_PROCESS |  | 84 | 0.42 | 1.30 | 0.095 | 0.347 | 1.000 | 2956 | tags=25%, list=14%, signal=29% |
| 776 | GO\_CARTILAGE\_DEVELOPMENT |  | 141 | 0.48 | 1.30 | 0.109 | 0.347 | 1.000 | 3783 | tags=35%, list=17%, signal=43% |
| 777 | GO\_KIDNEY\_EPITHELIUM\_DEVELOPMENT |  | 124 | 0.45 | 1.30 | 0.091 | 0.348 | 1.000 | 3138 | tags=29%, list=14%, signal=34% |
| 778 | GO\_EAR\_MORPHOGENESIS |  | 108 | 0.44 | 1.29 | 0.091 | 0.348 | 1.000 | 3230 | tags=27%, list=15%, signal=31% |
| 779 | GO\_CELL\_FATE\_COMMITMENT |  | 219 | 0.40 | 1.29 | 0.061 | 0.349 | 1.000 | 3407 | tags=22%, list=16%, signal=26% |
| 780 | GO\_POSITIVE\_REGULATION\_OF\_EPITHELIAL\_CELL\_APOPTOTIC\_PROCESS |  | 22 | 0.56 | 1.29 | 0.143 | 0.350 | 1.000 | 2956 | tags=45%, list=14%, signal=53% |
| 781 | GO\_POSITIVE\_REGULATION\_OF\_ANTIGEN\_PROCESSING\_AND\_PRESENTATION |  | 16 | 0.66 | 1.29 | 0.164 | 0.350 | 1.000 | 1913 | tags=38%, list=9%, signal=41% |
| 782 | GO\_ASSOCIATIVE\_LEARNING |  | 67 | 0.38 | 1.29 | 0.095 | 0.350 | 1.000 | 3288 | tags=15%, list=15%, signal=18% |
| 783 | GO\_NEGATIVE\_REGULATION\_OF\_SMOOTH\_MUSCLE\_CELL\_MIGRATION |  | 16 | 0.63 | 1.29 | 0.147 | 0.349 | 1.000 | 2167 | tags=44%, list=10%, signal=49% |
| 784 | GO\_POSITIVE\_REGULATION\_OF\_TRANSMEMBRANE\_RECEPTOR\_PROTEIN\_SERINE\_THREONINE\_KINASE\_SIGNALING\_PATHWAY |  | 95 | 0.44 | 1.29 | 0.117 | 0.349 | 1.000 | 2733 | tags=22%, list=13%, signal=25% |
| 785 | GO\_GLYCEROLIPID\_CATABOLIC\_PROCESS |  | 34 | 0.51 | 1.29 | 0.089 | 0.349 | 1.000 | 2784 | tags=26%, list=13%, signal=30% |
| 786 | GO\_PHOSPHATIDYLSERINE\_ACYL\_CHAIN\_REMODELING |  | 15 | 0.66 | 1.29 | 0.150 | 0.349 | 1.000 | 160 | tags=13%, list=1%, signal=13% |
| 787 | GO\_NEGATIVE\_REGULATION\_OF\_B\_CELL\_ACTIVATION |  | 30 | 0.56 | 1.29 | 0.143 | 0.349 | 1.000 | 3176 | tags=43%, list=15%, signal=51% |
| 788 | GO\_REGULATION\_OF\_POTASSIUM\_ION\_TRANSMEMBRANE\_TRANSPORTER\_ACTIVITY |  | 41 | 0.46 | 1.29 | 0.124 | 0.349 | 1.000 | 716 | tags=12%, list=3%, signal=13% |
| 789 | GO\_ATRIOVENTRICULAR\_VALVE\_DEVELOPMENT |  | 19 | 0.61 | 1.29 | 0.172 | 0.349 | 1.000 | 2974 | tags=42%, list=14%, signal=49% |
| 790 | GO\_NEUTRAL\_LIPID\_BIOSYNTHETIC\_PROCESS |  | 26 | 0.55 | 1.29 | 0.107 | 0.349 | 1.000 | 3900 | tags=38%, list=18%, signal=47% |
| 791 | GO\_CELL\_FATE\_COMMITMENT\_INVOLVED\_IN\_FORMATION\_OF\_PRIMARY\_GERM\_LAYER |  | 26 | 0.51 | 1.29 | 0.131 | 0.350 | 1.000 | 2956 | tags=23%, list=14%, signal=27% |
| 792 | GO\_NEPHRON\_EPITHELIUM\_DEVELOPMENT |  | 92 | 0.47 | 1.29 | 0.083 | 0.349 | 1.000 | 3267 | tags=30%, list=15%, signal=36% |
| 793 | GO\_REGULATION\_OF\_SMOOTH\_MUSCLE\_CELL\_MIGRATION |  | 48 | 0.55 | 1.29 | 0.130 | 0.349 | 1.000 | 3194 | tags=44%, list=15%, signal=51% |
| 794 | GO\_CELLULAR\_EXTRAVASATION |  | 24 | 0.62 | 1.29 | 0.164 | 0.349 | 1.000 | 1368 | tags=38%, list=6%, signal=40% |
| 795 | GO\_REGULATION\_OF\_CELL\_FATE\_COMMITMENT |  | 24 | 0.56 | 1.29 | 0.122 | 0.350 | 1.000 | 2960 | tags=38%, list=14%, signal=43% |
| 796 | GO\_ATRIAL\_SEPTUM\_DEVELOPMENT |  | 18 | 0.54 | 1.29 | 0.172 | 0.349 | 1.000 | 3433 | tags=33%, list=16%, signal=40% |
| 797 | GO\_REGULATION\_OF\_DEVELOPMENTAL\_GROWTH |  | 278 | 0.36 | 1.29 | 0.048 | 0.350 | 1.000 | 3353 | tags=23%, list=15%, signal=27% |
| 798 | GO\_POSITIVE\_CHEMOTAXIS |  | 31 | 0.54 | 1.29 | 0.118 | 0.351 | 1.000 | 2956 | tags=39%, list=14%, signal=45% |
| 799 | GO\_RESPONSE\_TO\_INTERLEUKIN\_6 |  | 25 | 0.50 | 1.29 | 0.146 | 0.353 | 1.000 | 2424 | tags=28%, list=11%, signal=31% |
| 800 | GO\_GLOMERULAR\_EPITHELIUM\_DEVELOPMENT |  | 19 | 0.55 | 1.29 | 0.124 | 0.355 | 1.000 | 3019 | tags=37%, list=14%, signal=43% |
| 801 | GO\_REGULATION\_OF\_LEUKOCYTE\_DIFFERENTIATION |  | 220 | 0.46 | 1.29 | 0.130 | 0.355 | 1.000 | 3582 | tags=35%, list=16%, signal=41% |
| 802 | GO\_T\_CELL\_RECEPTOR\_SIGNALING\_PATHWAY |  | 136 | 0.50 | 1.29 | 0.220 | 0.355 | 1.000 | 2764 | tags=28%, list=13%, signal=32% |
| 803 | GO\_CORONARY\_VASCULATURE\_DEVELOPMENT |  | 36 | 0.52 | 1.29 | 0.163 | 0.355 | 1.000 | 1733 | tags=19%, list=8%, signal=21% |
| 804 | GO\_POSITIVE\_T\_CELL\_SELECTION |  | 21 | 0.65 | 1.29 | 0.212 | 0.355 | 1.000 | 3406 | tags=52%, list=16%, signal=62% |
| 805 | GO\_REGULATION\_OF\_MULTICELLULAR\_ORGANISMAL\_METABOLIC\_PROCESS |  | 35 | 0.49 | 1.29 | 0.119 | 0.355 | 1.000 | 3080 | tags=34%, list=14%, signal=40% |
| 806 | GO\_RESPONSE\_TO\_ACIDIC\_PH |  | 16 | 0.50 | 1.29 | 0.161 | 0.354 | 1.000 | 2350 | tags=25%, list=11%, signal=28% |
| 807 | GO\_REGULATION\_OF\_REACTIVE\_OXYGEN\_SPECIES\_BIOSYNTHETIC\_PROCESS |  | 63 | 0.48 | 1.28 | 0.077 | 0.355 | 1.000 | 3170 | tags=38%, list=15%, signal=44% |
| 808 | GO\_REGULATION\_OF\_NF\_KAPPAB\_IMPORT\_INTO\_NUCLEUS |  | 45 | 0.49 | 1.28 | 0.102 | 0.355 | 1.000 | 2042 | tags=24%, list=9%, signal=27% |
| 809 | GO\_PHOSPHATIDYLINOSITOL\_METABOLIC\_PROCESS |  | 183 | 0.37 | 1.28 | 0.029 | 0.355 | 1.000 | 3132 | tags=22%, list=14%, signal=26% |
| 810 | GO\_PLATELET\_MORPHOGENESIS |  | 19 | 0.43 | 1.28 | 0.154 | 0.354 | 1.000 | 4232 | tags=32%, list=19%, signal=39% |
| 811 | GO\_NEGATIVE\_REGULATION\_OF\_IMMUNE\_RESPONSE |  | 116 | 0.50 | 1.28 | 0.126 | 0.355 | 1.000 | 3473 | tags=32%, list=16%, signal=38% |
| 812 | GO\_POSITIVE\_REGULATION\_OF\_CYTOPLASMIC\_TRANSPORT |  | 263 | 0.37 | 1.28 | 0.047 | 0.355 | 1.000 | 3153 | tags=23%, list=14%, signal=26% |
| 813 | GO\_REGULATION\_OF\_CHROMATIN\_BINDING |  | 16 | 0.46 | 1.28 | 0.170 | 0.356 | 1.000 | 3697 | tags=31%, list=17%, signal=38% |
| 814 | GO\_REGULATION\_OF\_PRODUCTION\_OF\_MOLECULAR\_MEDIATOR\_OF\_IMMUNE\_RESPONSE |  | 98 | 0.49 | 1.28 | 0.126 | 0.356 | 1.000 | 3241 | tags=37%, list=15%, signal=43% |
| 815 | GO\_REGULATION\_OF\_BONE\_DEVELOPMENT |  | 18 | 0.59 | 1.28 | 0.140 | 0.357 | 1.000 | 4261 | tags=56%, list=20%, signal=69% |
| 816 | GO\_REGULATION\_OF\_CYTOKINE\_SECRETION |  | 136 | 0.51 | 1.28 | 0.124 | 0.357 | 1.000 | 3736 | tags=43%, list=17%, signal=51% |
| 817 | GO\_FOREBRAIN\_GENERATION\_OF\_NEURONS |  | 64 | 0.43 | 1.28 | 0.110 | 0.358 | 1.000 | 2898 | tags=20%, list=13%, signal=23% |
| 818 | GO\_FAT\_CELL\_DIFFERENTIATION |  | 99 | 0.42 | 1.28 | 0.074 | 0.358 | 1.000 | 3877 | tags=32%, list=18%, signal=39% |
| 819 | GO\_REGULATION\_OF\_GLUCOSE\_TRANSPORT |  | 93 | 0.39 | 1.28 | 0.109 | 0.359 | 1.000 | 2791 | tags=24%, list=13%, signal=27% |
| 820 | GO\_REGULATION\_OF\_MEMBRANE\_LIPID\_DISTRIBUTION |  | 35 | 0.45 | 1.28 | 0.108 | 0.359 | 1.000 | 3751 | tags=31%, list=17%, signal=38% |
| 821 | GO\_POSITIVE\_REGULATION\_OF\_CELL\_ADHESION\_MEDIATED\_BY\_INTEGRIN |  | 17 | 0.63 | 1.28 | 0.140 | 0.359 | 1.000 | 2030 | tags=41%, list=9%, signal=45% |
| 822 | GO\_NEGATIVE\_REGULATION\_OF\_CELL\_JUNCTION\_ASSEMBLY |  | 19 | 0.54 | 1.28 | 0.172 | 0.360 | 1.000 | 2647 | tags=32%, list=12%, signal=36% |
| 823 | GO\_REGULATION\_OF\_MYELOID\_LEUKOCYTE\_DIFFERENTIATION |  | 100 | 0.43 | 1.28 | 0.134 | 0.360 | 1.000 | 3783 | tags=31%, list=17%, signal=37% |
| 824 | GO\_FILOPODIUM\_ASSEMBLY |  | 17 | 0.53 | 1.28 | 0.115 | 0.360 | 1.000 | 2265 | tags=41%, list=10%, signal=46% |
| 825 | GO\_T\_CELL\_MEDIATED\_IMMUNITY |  | 28 | 0.57 | 1.28 | 0.165 | 0.360 | 1.000 | 2917 | tags=39%, list=13%, signal=45% |
| 826 | GO\_SIALYLATION |  | 20 | 0.56 | 1.28 | 0.138 | 0.361 | 1.000 | 3126 | tags=45%, list=14%, signal=53% |
| 827 | GO\_NEURONAL\_ACTION\_POTENTIAL |  | 26 | 0.50 | 1.28 | 0.137 | 0.365 | 1.000 | 4213 | tags=35%, list=19%, signal=43% |
| 828 | GO\_REGULATION\_OF\_EARLY\_ENDOSOME\_TO\_LATE\_ENDOSOME\_TRANSPORT |  | 16 | 0.46 | 1.28 | 0.160 | 0.365 | 1.000 | 1620 | tags=13%, list=7%, signal=13% |
| 829 | GO\_REGULATION\_OF\_TUMOR\_NECROSIS\_FACTOR\_BIOSYNTHETIC\_PROCESS |  | 16 | 0.63 | 1.28 | 0.224 | 0.365 | 1.000 | 1926 | tags=25%, list=9%, signal=27% |
| 830 | GO\_PURINERGIC\_RECEPTOR\_SIGNALING\_PATHWAY |  | 26 | 0.65 | 1.28 | 0.182 | 0.364 | 1.000 | 2142 | tags=42%, list=10%, signal=47% |
| 831 | GO\_NEGATIVE\_REGULATION\_OF\_NERVOUS\_SYSTEM\_DEVELOPMENT |  | 251 | 0.37 | 1.27 | 0.061 | 0.364 | 1.000 | 3105 | tags=22%, list=14%, signal=25% |
| 832 | GO\_BONE\_MORPHOGENESIS |  | 77 | 0.49 | 1.27 | 0.141 | 0.364 | 1.000 | 2956 | tags=34%, list=14%, signal=39% |
| 833 | GO\_REGULATION\_OF\_MUSCLE\_TISSUE\_DEVELOPMENT |  | 98 | 0.47 | 1.27 | 0.101 | 0.363 | 1.000 | 3290 | tags=35%, list=15%, signal=41% |
| 834 | GO\_REGULATION\_OF\_ANTIGEN\_RECEPTOR\_MEDIATED\_SIGNALING\_PATHWAY |  | 38 | 0.59 | 1.27 | 0.183 | 0.363 | 1.000 | 2122 | tags=37%, list=10%, signal=41% |
| 835 | GO\_LYMPHOCYTE\_ACTIVATION\_INVOLVED\_IN\_IMMUNE\_RESPONSE |  | 96 | 0.49 | 1.27 | 0.172 | 0.364 | 1.000 | 3406 | tags=38%, list=16%, signal=44% |
| 836 | GO\_NEGATIVE\_REGULATION\_OF\_DEVELOPMENTAL\_GROWTH |  | 81 | 0.41 | 1.27 | 0.078 | 0.364 | 1.000 | 3578 | tags=28%, list=16%, signal=34% |
| 837 | GO\_REGULATION\_OF\_ENDOTHELIAL\_CELL\_MIGRATION |  | 107 | 0.43 | 1.27 | 0.141 | 0.364 | 1.000 | 2956 | tags=29%, list=14%, signal=33% |
| 838 | GO\_REGULATION\_OF\_CELL\_CELL\_ADHESION |  | 358 | 0.46 | 1.27 | 0.133 | 0.363 | 1.000 | 3478 | tags=34%, list=16%, signal=40% |
| 839 | GO\_NEGATIVE\_REGULATION\_OF\_CELL\_PROJECTION\_ORGANIZATION |  | 137 | 0.39 | 1.27 | 0.084 | 0.363 | 1.000 | 2977 | tags=23%, list=14%, signal=27% |
| 840 | GO\_REGULATION\_OF\_CELL\_MORPHOGENESIS\_INVOLVED\_IN\_DIFFERENTIATION |  | 319 | 0.38 | 1.27 | 0.098 | 0.363 | 1.000 | 3489 | tags=25%, list=16%, signal=30% |
| 841 | GO\_NEGATIVE\_REGULATION\_OF\_CELL\_MORPHOGENESIS\_INVOLVED\_IN\_DIFFERENTIATION |  | 110 | 0.40 | 1.27 | 0.097 | 0.363 | 1.000 | 3732 | tags=25%, list=17%, signal=29% |
| 842 | GO\_POSITIVE\_REGULATION\_OF\_HOMEOSTATIC\_PROCESS |  | 201 | 0.37 | 1.27 | 0.058 | 0.364 | 1.000 | 3785 | tags=27%, list=17%, signal=32% |
| 843 | GO\_NEGATIVE\_REGULATION\_OF\_CYTOKINE\_PRODUCTION |  | 191 | 0.46 | 1.27 | 0.127 | 0.364 | 1.000 | 3814 | tags=35%, list=18%, signal=42% |
| 844 | GO\_MAINTENANCE\_OF\_LOCATION |  | 131 | 0.37 | 1.27 | 0.096 | 0.363 | 1.000 | 3825 | tags=24%, list=18%, signal=29% |
| 845 | GO\_REGULATION\_OF\_ADHERENS\_JUNCTION\_ORGANIZATION |  | 49 | 0.45 | 1.27 | 0.124 | 0.363 | 1.000 | 3757 | tags=37%, list=17%, signal=44% |
| 846 | GO\_HYDROGEN\_ION\_TRANSMEMBRANE\_TRANSPORT |  | 91 | 0.38 | 1.27 | 0.153 | 0.363 | 1.000 | 4867 | tags=24%, list=22%, signal=31% |
| 847 | GO\_ACTIN\_CYTOSKELETON\_REORGANIZATION |  | 54 | 0.41 | 1.27 | 0.143 | 0.363 | 1.000 | 1957 | tags=19%, list=9%, signal=20% |
| 848 | GO\_OTIC\_VESICLE\_DEVELOPMENT |  | 15 | 0.56 | 1.27 | 0.160 | 0.362 | 1.000 | 2908 | tags=40%, list=13%, signal=46% |
| 849 | GO\_CHONDROITIN\_SULFATE\_BIOSYNTHETIC\_PROCESS |  | 25 | 0.61 | 1.27 | 0.203 | 0.364 | 1.000 | 3005 | tags=32%, list=14%, signal=37% |
| 850 | GO\_NEGATIVE\_REGULATION\_OF\_RESPONSE\_TO\_WOUNDING |  | 146 | 0.42 | 1.27 | 0.065 | 0.364 | 1.000 | 2862 | tags=25%, list=13%, signal=28% |
| 851 | GO\_NEGATIVE\_REGULATION\_OF\_MYELOID\_CELL\_DIFFERENTIATION |  | 74 | 0.42 | 1.27 | 0.103 | 0.363 | 1.000 | 3783 | tags=27%, list=17%, signal=33% |
| 852 | GO\_REGULATION\_OF\_ANTIGEN\_PROCESSING\_AND\_PRESENTATION |  | 23 | 0.63 | 1.27 | 0.196 | 0.363 | 1.000 | 3945 | tags=52%, list=18%, signal=64% |
| 853 | GO\_REGULATION\_OF\_ACTIN\_NUCLEATION |  | 26 | 0.42 | 1.27 | 0.120 | 0.363 | 1.000 | 2606 | tags=23%, list=12%, signal=26% |
| 854 | GO\_NEGATIVE\_REGULATION\_OF\_CANONICAL\_WNT\_SIGNALING\_PATHWAY |  | 155 | 0.39 | 1.27 | 0.109 | 0.363 | 1.000 | 3221 | tags=21%, list=15%, signal=24% |
| 855 | GO\_POSITIVE\_REGULATION\_OF\_PROTEIN\_KINASE\_B\_SIGNALING |  | 76 | 0.46 | 1.27 | 0.095 | 0.364 | 1.000 | 2912 | tags=38%, list=13%, signal=44% |
| 856 | GO\_ERYTHROCYTE\_DEVELOPMENT |  | 22 | 0.46 | 1.27 | 0.186 | 0.364 | 1.000 | 3407 | tags=32%, list=16%, signal=38% |
| 857 | GO\_REGULATION\_OF\_CYTOKINE\_PRODUCTION\_INVOLVED\_IN\_INFLAMMATORY\_RESPONSE |  | 16 | 0.54 | 1.27 | 0.167 | 0.364 | 1.000 | 3473 | tags=44%, list=16%, signal=52% |
| 858 | GO\_NEGATIVE\_REGULATION\_OF\_ERK1\_AND\_ERK2\_CASCADE |  | 49 | 0.44 | 1.27 | 0.137 | 0.364 | 1.000 | 2708 | tags=22%, list=12%, signal=26% |
| 859 | GO\_CELLULAR\_RESPONSE\_TO\_ORGANIC\_CYCLIC\_COMPOUND |  | 439 | 0.35 | 1.27 | 0.040 | 0.364 | 1.000 | 2245 | tags=16%, list=10%, signal=18% |
| 860 | GO\_ENERGY\_RESERVE\_METABOLIC\_PROCESS |  | 67 | 0.44 | 1.27 | 0.173 | 0.364 | 1.000 | 4761 | tags=39%, list=22%, signal=50% |
| 861 | GO\_VENTRICULAR\_SEPTUM\_MORPHOGENESIS |  | 28 | 0.57 | 1.27 | 0.136 | 0.364 | 1.000 | 3058 | tags=39%, list=14%, signal=46% |
| 862 | GO\_FUCOSYLATION |  | 22 | 0.53 | 1.27 | 0.148 | 0.364 | 1.000 | 2598 | tags=23%, list=12%, signal=26% |
| 863 | GO\_REGULATION\_OF\_ALPHA\_BETA\_T\_CELL\_ACTIVATION |  | 68 | 0.55 | 1.27 | 0.181 | 0.364 | 1.000 | 3526 | tags=41%, list=16%, signal=49% |
| 864 | GO\_POSITIVE\_REGULATION\_OF\_KINASE\_ACTIVITY |  | 455 | 0.35 | 1.27 | 0.030 | 0.365 | 1.000 | 3579 | tags=28%, list=16%, signal=33% |
| 865 | GO\_REGULATION\_OF\_COAGULATION |  | 85 | 0.47 | 1.27 | 0.100 | 0.365 | 1.000 | 2464 | tags=27%, list=11%, signal=30% |
| 866 | GO\_TAXIS |  | 426 | 0.43 | 1.27 | 0.102 | 0.365 | 1.000 | 3095 | tags=27%, list=14%, signal=31% |
| 867 | GO\_NEGATIVE\_REGULATION\_OF\_POTASSIUM\_ION\_TRANSMEMBRANE\_TRANSPORT |  | 20 | 0.53 | 1.27 | 0.175 | 0.365 | 1.000 | 1410 | tags=20%, list=6%, signal=21% |
| 868 | GO\_PHOSPHOLIPASE\_C\_ACTIVATING\_G\_PROTEIN\_COUPLED\_RECEPTOR\_SIGNALING\_PATHWAY |  | 80 | 0.42 | 1.27 | 0.123 | 0.365 | 1.000 | 1378 | tags=11%, list=6%, signal=12% |
| 869 | GO\_REGULATION\_OF\_NEUROTRANSMITTER\_SECRETION |  | 45 | 0.44 | 1.27 | 0.124 | 0.366 | 1.000 | 3913 | tags=27%, list=18%, signal=32% |
| 870 | GO\_INNER\_EAR\_RECEPTOR\_CELL\_DEVELOPMENT |  | 31 | 0.44 | 1.27 | 0.127 | 0.366 | 1.000 | 1817 | tags=16%, list=8%, signal=18% |
| 871 | GO\_COGNITION |  | 236 | 0.36 | 1.26 | 0.092 | 0.366 | 1.000 | 3692 | tags=22%, list=17%, signal=26% |
| 872 | GO\_POSITIVE\_REGULATION\_OF\_CYTOKINE\_SECRETION |  | 90 | 0.50 | 1.26 | 0.143 | 0.366 | 1.000 | 3084 | tags=38%, list=14%, signal=44% |
| 873 | GO\_VACUOLE\_FUSION |  | 21 | 0.42 | 1.26 | 0.169 | 0.366 | 1.000 | 4771 | tags=33%, list=22%, signal=43% |
| 874 | GO\_NEGATIVE\_REGULATION\_OF\_STRESS\_FIBER\_ASSEMBLY |  | 16 | 0.51 | 1.26 | 0.157 | 0.366 | 1.000 | 1465 | tags=25%, list=7%, signal=27% |
| 875 | GO\_RESPONSE\_TO\_MERCURY\_ION |  | 15 | 0.53 | 1.26 | 0.173 | 0.366 | 1.000 | 1574 | tags=20%, list=7%, signal=22% |
| 876 | GO\_REGULATION\_OF\_ALPHA\_BETA\_T\_CELL\_PROLIFERATION |  | 23 | 0.65 | 1.26 | 0.207 | 0.366 | 1.000 | 2764 | tags=43%, list=13%, signal=50% |
| 877 | GO\_POSITIVE\_REGULATION\_OF\_PROTEIN\_SECRETION |  | 200 | 0.40 | 1.26 | 0.084 | 0.365 | 1.000 | 3293 | tags=26%, list=15%, signal=31% |
| 878 | GO\_CELL\_SUBSTRATE\_ADHERENS\_JUNCTION\_ASSEMBLY |  | 23 | 0.52 | 1.26 | 0.154 | 0.366 | 1.000 | 1251 | tags=26%, list=6%, signal=28% |
| 879 | GO\_DIGESTIVE\_SYSTEM\_DEVELOPMENT |  | 141 | 0.44 | 1.26 | 0.098 | 0.365 | 1.000 | 3482 | tags=30%, list=16%, signal=35% |
| 880 | GO\_REGULATION\_OF\_ENDOTHELIAL\_CELL\_APOPTOTIC\_PROCESS |  | 40 | 0.52 | 1.26 | 0.161 | 0.365 | 1.000 | 2956 | tags=43%, list=14%, signal=49% |
| 881 | GO\_RESPONSE\_TO\_OXYGEN\_LEVELS |  | 301 | 0.38 | 1.26 | 0.087 | 0.365 | 1.000 | 3489 | tags=27%, list=16%, signal=32% |
| 882 | GO\_REGULATION\_OF\_BEHAVIOR |  | 61 | 0.40 | 1.26 | 0.134 | 0.366 | 1.000 | 2908 | tags=18%, list=13%, signal=21% |
| 883 | GO\_NEGATIVE\_REGULATION\_OF\_AXON\_EXTENSION |  | 35 | 0.47 | 1.26 | 0.127 | 0.366 | 1.000 | 3194 | tags=26%, list=15%, signal=30% |
| 884 | GO\_RESPONSE\_TO\_TEMPERATURE\_STIMULUS |  | 140 | 0.39 | 1.26 | 0.089 | 0.366 | 1.000 | 2281 | tags=18%, list=10%, signal=20% |
| 885 | GO\_RENAL\_SYSTEM\_PROCESS\_INVOLVED\_IN\_REGULATION\_OF\_SYSTEMIC\_ARTERIAL\_BLOOD\_PRESSURE |  | 23 | 0.53 | 1.26 | 0.147 | 0.366 | 1.000 | 2912 | tags=30%, list=13%, signal=35% |
| 886 | GO\_POSITIVE\_REGULATION\_OF\_SECRETION |  | 353 | 0.36 | 1.26 | 0.049 | 0.365 | 1.000 | 3630 | tags=24%, list=17%, signal=28% |
| 887 | GO\_REGULATION\_OF\_DENDRITE\_DEVELOPMENT |  | 115 | 0.37 | 1.26 | 0.081 | 0.365 | 1.000 | 2694 | tags=20%, list=12%, signal=23% |
| 888 | GO\_RESPONSE\_TO\_UV\_B |  | 16 | 0.54 | 1.26 | 0.122 | 0.365 | 1.000 | 2089 | tags=31%, list=10%, signal=35% |
| 889 | GO\_SINGLE\_ORGANISM\_BEHAVIOR |  | 365 | 0.35 | 1.26 | 0.072 | 0.366 | 1.000 | 3347 | tags=18%, list=15%, signal=21% |
| 890 | GO\_NEPHRON\_DEVELOPMENT |  | 114 | 0.46 | 1.26 | 0.111 | 0.367 | 1.000 | 3267 | tags=32%, list=15%, signal=37% |
| 891 | GO\_REGULATION\_OF\_CELL\_GROWTH |  | 371 | 0.36 | 1.26 | 0.063 | 0.368 | 1.000 | 3353 | tags=24%, list=15%, signal=28% |
| 892 | GO\_CEREBRAL\_CORTEX\_CELL\_MIGRATION |  | 41 | 0.41 | 1.26 | 0.153 | 0.369 | 1.000 | 3310 | tags=27%, list=15%, signal=32% |
| 893 | GO\_MEMBRANE\_RAFT\_ORGANIZATION |  | 17 | 0.53 | 1.26 | 0.164 | 0.369 | 1.000 | 2469 | tags=35%, list=11%, signal=40% |
| 894 | GO\_MUSCLE\_CELL\_PROLIFERATION |  | 18 | 0.59 | 1.26 | 0.161 | 0.370 | 1.000 | 2116 | tags=28%, list=10%, signal=31% |
| 895 | GO\_REGULATION\_OF\_TRANSCRIPTION\_FACTOR\_IMPORT\_INTO\_NUCLEUS |  | 91 | 0.41 | 1.26 | 0.120 | 0.370 | 1.000 | 2526 | tags=20%, list=12%, signal=22% |
| 896 | GO\_POSITIVE\_REGULATION\_OF\_EMBRYONIC\_DEVELOPMENT |  | 29 | 0.51 | 1.26 | 0.124 | 0.370 | 1.000 | 2020 | tags=21%, list=9%, signal=23% |
| 897 | GO\_G\_PROTEIN\_COUPLED\_PURINERGIC\_RECEPTOR\_SIGNALING\_PATHWAY |  | 18 | 0.67 | 1.26 | 0.205 | 0.370 | 1.000 | 1045 | tags=44%, list=5%, signal=47% |
| 898 | GO\_NEURON\_RECOGNITION |  | 33 | 0.53 | 1.26 | 0.155 | 0.370 | 1.000 | 3472 | tags=39%, list=16%, signal=47% |
| 899 | GO\_IMMUNE\_EFFECTOR\_PROCESS |  | 435 | 0.47 | 1.26 | 0.193 | 0.372 | 1.000 | 3406 | tags=32%, list=16%, signal=38% |
| 900 | GO\_NEGATIVE\_REGULATION\_OF\_PLATELET\_ACTIVATION |  | 17 | 0.55 | 1.26 | 0.124 | 0.372 | 1.000 | 2862 | tags=41%, list=13%, signal=47% |
| 901 | GO\_POSITIVE\_REGULATION\_OF\_CARTILAGE\_DEVELOPMENT |  | 27 | 0.52 | 1.26 | 0.159 | 0.372 | 1.000 | 3407 | tags=33%, list=16%, signal=39% |
| 902 | GO\_POSITIVE\_REGULATION\_OF\_TUMOR\_NECROSIS\_FACTOR\_SUPERFAMILY\_CYTOKINE\_PRODUCTION |  | 54 | 0.56 | 1.25 | 0.205 | 0.373 | 1.000 | 4435 | tags=52%, list=20%, signal=65% |
| 903 | GO\_AMEBOIDAL\_TYPE\_CELL\_MIGRATION |  | 148 | 0.40 | 1.25 | 0.102 | 0.373 | 1.000 | 3298 | tags=26%, list=15%, signal=31% |
| 904 | GO\_GLYCEROLIPID\_METABOLIC\_PROCESS |  | 336 | 0.35 | 1.25 | 0.040 | 0.372 | 1.000 | 3969 | tags=26%, list=18%, signal=31% |
| 905 | GO\_NEGATIVE\_REGULATION\_OF\_GLIOGENESIS |  | 37 | 0.46 | 1.25 | 0.117 | 0.372 | 1.000 | 3105 | tags=30%, list=14%, signal=35% |
| 906 | GO\_NEGATIVE\_REGULATION\_OF\_STAT\_CASCADE |  | 42 | 0.44 | 1.25 | 0.149 | 0.372 | 1.000 | 1513 | tags=12%, list=7%, signal=13% |
| 907 | GO\_PROTEIN\_POLYMERIZATION |  | 64 | 0.40 | 1.25 | 0.099 | 0.372 | 1.000 | 1340 | tags=17%, list=6%, signal=18% |
| 908 | GO\_POSITIVE\_REGULATION\_OF\_CELL\_CELL\_ADHESION |  | 228 | 0.49 | 1.25 | 0.181 | 0.372 | 1.000 | 3129 | tags=34%, list=14%, signal=40% |
| 909 | GO\_POSITIVE\_REGULATION\_OF\_POTASSIUM\_ION\_TRANSMEMBRANE\_TRANSPORTER\_ACTIVITY |  | 16 | 0.50 | 1.25 | 0.174 | 0.372 | 1.000 | 124 | tags=13%, list=1%, signal=13% |
| 910 | GO\_STEM\_CELL\_DIFFERENTIATION |  | 186 | 0.41 | 1.25 | 0.090 | 0.372 | 1.000 | 3408 | tags=29%, list=16%, signal=34% |
| 911 | GO\_POSITIVE\_REGULATION\_OF\_SMOOTH\_MUSCLE\_CELL\_MIGRATION |  | 29 | 0.56 | 1.25 | 0.179 | 0.372 | 1.000 | 3194 | tags=48%, list=15%, signal=57% |
| 912 | GO\_PLASMA\_MEMBRANE\_ORGANIZATION |  | 198 | 0.35 | 1.25 | 0.090 | 0.373 | 1.000 | 3146 | tags=22%, list=14%, signal=25% |
| 913 | GO\_NEGATIVE\_REGULATION\_OF\_CYTOKINE\_SECRETION |  | 40 | 0.52 | 1.25 | 0.176 | 0.372 | 1.000 | 3355 | tags=45%, list=15%, signal=53% |
| 914 | GO\_CELLULAR\_RESPONSE\_TO\_LIPID |  | 427 | 0.37 | 1.25 | 0.077 | 0.372 | 1.000 | 2478 | tags=20%, list=11%, signal=22% |
| 915 | GO\_MEMORY |  | 90 | 0.37 | 1.25 | 0.108 | 0.372 | 1.000 | 3652 | tags=22%, list=17%, signal=27% |
| 916 | GO\_NEGATIVE\_REGULATION\_OF\_MYELOID\_LEUKOCYTE\_DIFFERENTIATION |  | 41 | 0.48 | 1.25 | 0.150 | 0.373 | 1.000 | 3783 | tags=32%, list=17%, signal=38% |
| 917 | GO\_NEGATIVE\_REGULATION\_OF\_RECEPTOR\_ACTIVITY |  | 27 | 0.48 | 1.25 | 0.125 | 0.373 | 1.000 | 2133 | tags=26%, list=10%, signal=29% |
| 918 | GO\_POLYSACCHARIDE\_CATABOLIC\_PROCESS |  | 23 | 0.48 | 1.25 | 0.143 | 0.374 | 1.000 | 4951 | tags=48%, list=23%, signal=62% |
| 919 | GO\_RESPONSE\_TO\_NITRIC\_OXIDE |  | 21 | 0.53 | 1.25 | 0.177 | 0.374 | 1.000 | 2956 | tags=38%, list=14%, signal=44% |
| 920 | GO\_CAMP\_BIOSYNTHETIC\_PROCESS |  | 16 | 0.55 | 1.25 | 0.186 | 0.374 | 1.000 | 3452 | tags=38%, list=16%, signal=45% |
| 921 | GO\_CELL\_ACTIVATION\_INVOLVED\_IN\_IMMUNE\_RESPONSE |  | 136 | 0.48 | 1.25 | 0.205 | 0.375 | 1.000 | 3406 | tags=36%, list=16%, signal=42% |
| 922 | GO\_PEPTIDYL\_TYROSINE\_AUTOPHOSPHORYLATION |  | 38 | 0.52 | 1.25 | 0.194 | 0.375 | 1.000 | 2787 | tags=39%, list=13%, signal=45% |
| 923 | GO\_PROTEIN\_N\_LINKED\_GLYCOSYLATION |  | 73 | 0.43 | 1.25 | 0.208 | 0.376 | 1.000 | 2780 | tags=21%, list=13%, signal=23% |
| 924 | GO\_RIBONUCLEOSIDE\_CATABOLIC\_PROCESS |  | 23 | 0.50 | 1.25 | 0.121 | 0.376 | 1.000 | 267 | tags=13%, list=1%, signal=13% |
| 925 | GO\_NEGATIVE\_REGULATION\_OF\_AUTOPHAGY |  | 50 | 0.36 | 1.25 | 0.130 | 0.377 | 1.000 | 5583 | tags=44%, list=26%, signal=59% |
| 926 | GO\_NEGATIVE\_REGULATION\_OF\_SMOOTHENED\_SIGNALING\_PATHWAY |  | 25 | 0.52 | 1.25 | 0.140 | 0.376 | 1.000 | 2014 | tags=28%, list=9%, signal=31% |
| 927 | GO\_REGULATION\_OF\_INTRACELLULAR\_STEROID\_HORMONE\_RECEPTOR\_SIGNALING\_PATHWAY |  | 55 | 0.37 | 1.25 | 0.091 | 0.376 | 1.000 | 2013 | tags=15%, list=9%, signal=16% |
| 928 | GO\_POSITIVE\_REGULATION\_OF\_I\_KAPPAB\_KINASE\_NF\_KAPPAB\_SIGNALING |  | 170 | 0.41 | 1.25 | 0.141 | 0.376 | 1.000 | 3166 | tags=25%, list=15%, signal=29% |
| 929 | GO\_REGULATION\_OF\_CANONICAL\_WNT\_SIGNALING\_PATHWAY |  | 224 | 0.37 | 1.25 | 0.109 | 0.375 | 1.000 | 3267 | tags=21%, list=15%, signal=25% |
| 930 | GO\_REGULATION\_OF\_MITOCHONDRIAL\_MEMBRANE\_POTENTIAL |  | 53 | 0.41 | 1.25 | 0.098 | 0.376 | 1.000 | 3304 | tags=34%, list=15%, signal=40% |
| 931 | GO\_REGULATION\_OF\_JUN\_KINASE\_ACTIVITY |  | 78 | 0.41 | 1.25 | 0.076 | 0.375 | 1.000 | 1921 | tags=21%, list=9%, signal=22% |
| 932 | GO\_CELLULAR\_RESPONSE\_TO\_CARBOHYDRATE\_STIMULUS |  | 72 | 0.41 | 1.25 | 0.130 | 0.375 | 1.000 | 4065 | tags=38%, list=19%, signal=46% |
| 933 | GO\_POSITIVE\_REGULATION\_OF\_ORGAN\_GROWTH |  | 36 | 0.49 | 1.25 | 0.127 | 0.375 | 1.000 | 2451 | tags=31%, list=11%, signal=34% |
| 934 | GO\_REGULATION\_OF\_ORGAN\_MORPHOGENESIS |  | 236 | 0.41 | 1.25 | 0.120 | 0.374 | 1.000 | 3309 | tags=25%, list=15%, signal=29% |
| 935 | GO\_REGULATION\_OF\_JNK\_CASCADE |  | 154 | 0.37 | 1.25 | 0.070 | 0.374 | 1.000 | 1979 | tags=18%, list=9%, signal=20% |
| 936 | GO\_CYTOLYSIS |  | 23 | 0.61 | 1.25 | 0.222 | 0.375 | 1.000 | 2612 | tags=39%, list=12%, signal=44% |
| 937 | GO\_CELLULAR\_RESPONSE\_TO\_INTERLEUKIN\_6 |  | 21 | 0.49 | 1.25 | 0.178 | 0.375 | 1.000 | 3080 | tags=33%, list=14%, signal=39% |
| 938 | GO\_MACROPHAGE\_ACTIVATION |  | 31 | 0.57 | 1.25 | 0.200 | 0.375 | 1.000 | 2477 | tags=42%, list=11%, signal=47% |
| 939 | GO\_DETECTION\_OF\_OTHER\_ORGANISM |  | 17 | 0.59 | 1.25 | 0.218 | 0.375 | 1.000 | 3945 | tags=47%, list=18%, signal=57% |
| 940 | GO\_CHONDROITIN\_SULFATE\_PROTEOGLYCAN\_BIOSYNTHETIC\_PROCESS |  | 29 | 0.58 | 1.25 | 0.195 | 0.375 | 1.000 | 1513 | tags=24%, list=7%, signal=26% |
| 941 | GO\_LIPID\_TRANSLOCATION |  | 21 | 0.50 | 1.25 | 0.177 | 0.376 | 1.000 | 3751 | tags=38%, list=17%, signal=46% |
| 942 | GO\_REGULATION\_OF\_CHEMOTAXIS |  | 172 | 0.48 | 1.25 | 0.158 | 0.376 | 1.000 | 3194 | tags=35%, list=15%, signal=41% |
| 943 | GO\_NEGATIVE\_REGULATION\_OF\_AXONOGENESIS |  | 61 | 0.44 | 1.24 | 0.135 | 0.376 | 1.000 | 3304 | tags=23%, list=15%, signal=27% |
| 944 | GO\_RESPONSE\_TO\_GROWTH\_FACTOR |  | 456 | 0.36 | 1.24 | 0.105 | 0.376 | 1.000 | 3101 | tags=23%, list=14%, signal=26% |
| 945 | GO\_REGULATION\_OF\_CELL\_ADHESION\_MEDIATED\_BY\_INTEGRIN |  | 39 | 0.51 | 1.24 | 0.136 | 0.376 | 1.000 | 2054 | tags=33%, list=9%, signal=37% |
| 946 | GO\_REGULATION\_OF\_WOUND\_HEALING |  | 122 | 0.43 | 1.24 | 0.101 | 0.376 | 1.000 | 2464 | tags=24%, list=11%, signal=27% |
| 947 | GO\_POSITIVE\_REGULATION\_OF\_NERVOUS\_SYSTEM\_DEVELOPMENT |  | 407 | 0.38 | 1.24 | 0.099 | 0.376 | 1.000 | 3785 | tags=27%, list=17%, signal=32% |
| 948 | GO\_AXONAL\_FASCICULATION |  | 20 | 0.57 | 1.24 | 0.161 | 0.376 | 1.000 | 3472 | tags=40%, list=16%, signal=48% |
| 949 | GO\_ENDOTHELIAL\_CELL\_PROLIFERATION |  | 24 | 0.47 | 1.24 | 0.149 | 0.376 | 1.000 | 3298 | tags=42%, list=15%, signal=49% |
| 950 | GO\_AUTOPHAGY |  | 363 | 0.29 | 1.24 | 0.077 | 0.377 | 1.000 | 3187 | tags=17%, list=15%, signal=20% |
| 951 | GO\_MONOVALENT\_INORGANIC\_CATION\_HOMEOSTASIS |  | 116 | 0.40 | 1.24 | 0.168 | 0.376 | 1.000 | 4406 | tags=27%, list=20%, signal=33% |
| 952 | GO\_GLYCOSPHINGOLIPID\_METABOLIC\_PROCESS |  | 62 | 0.41 | 1.24 | 0.115 | 0.377 | 1.000 | 4406 | tags=42%, list=20%, signal=52% |
| 953 | GO\_ENDOPLASMIC\_RETICULUM\_CALCIUM\_ION\_HOMEOSTASIS |  | 19 | 0.48 | 1.24 | 0.212 | 0.377 | 1.000 | 3016 | tags=32%, list=14%, signal=37% |
| 954 | GO\_POSITIVE\_REGULATION\_OF\_RESPONSE\_TO\_BIOTIC\_STIMULUS |  | 36 | 0.50 | 1.24 | 0.208 | 0.376 | 1.000 | 4204 | tags=36%, list=19%, signal=45% |
| 955 | GO\_MULTI\_ORGANISM\_MEMBRANE\_ORGANIZATION |  | 28 | 0.44 | 1.24 | 0.207 | 0.377 | 1.000 | 3032 | tags=25%, list=14%, signal=29% |
| 956 | GO\_REGULATION\_OF\_PHAGOCYTOSIS |  | 65 | 0.45 | 1.24 | 0.172 | 0.377 | 1.000 | 3800 | tags=37%, list=17%, signal=45% |
| 957 | GO\_REGULATION\_OF\_CELL\_SIZE |  | 162 | 0.35 | 1.24 | 0.081 | 0.377 | 1.000 | 3353 | tags=25%, list=15%, signal=30% |
| 958 | GO\_ORGAN\_FORMATION |  | 33 | 0.47 | 1.24 | 0.149 | 0.377 | 1.000 | 2182 | tags=24%, list=10%, signal=27% |
| 959 | GO\_REGULATION\_OF\_CORTICOSTEROID\_HORMONE\_SECRETION |  | 15 | 0.52 | 1.24 | 0.203 | 0.377 | 1.000 | 819 | tags=13%, list=4%, signal=14% |
| 960 | GO\_NEGATIVE\_REGULATION\_OF\_PEPTIDASE\_ACTIVITY |  | 223 | 0.38 | 1.24 | 0.100 | 0.377 | 1.000 | 3004 | tags=22%, list=14%, signal=25% |
| 961 | GO\_REGULATION\_OF\_LYMPHOCYTE\_DIFFERENTIATION |  | 128 | 0.47 | 1.24 | 0.184 | 0.378 | 1.000 | 3526 | tags=39%, list=16%, signal=46% |
| 962 | GO\_FOREBRAIN\_DEVELOPMENT |  | 348 | 0.35 | 1.24 | 0.097 | 0.378 | 1.000 | 3043 | tags=20%, list=14%, signal=22% |
| 963 | GO\_CELLULAR\_RESPONSE\_TO\_CAMP |  | 48 | 0.42 | 1.24 | 0.116 | 0.378 | 1.000 | 3562 | tags=31%, list=16%, signal=37% |
| 964 | GO\_POSITIVE\_REGULATION\_OF\_LOCOMOTION |  | 392 | 0.43 | 1.24 | 0.140 | 0.378 | 1.000 | 3119 | tags=29%, list=14%, signal=34% |
| 965 | GO\_POSITIVE\_REGULATION\_OF\_INTRACELLULAR\_TRANSPORT |  | 338 | 0.34 | 1.24 | 0.073 | 0.378 | 1.000 | 3153 | tags=22%, list=14%, signal=25% |
| 966 | GO\_NEGATIVE\_REGULATION\_OF\_CELL\_SUBSTRATE\_ADHESION |  | 49 | 0.46 | 1.24 | 0.157 | 0.378 | 1.000 | 1465 | tags=20%, list=7%, signal=22% |
| 967 | GO\_REGULATION\_OF\_T\_CELL\_MIGRATION |  | 24 | 0.61 | 1.24 | 0.199 | 0.378 | 1.000 | 1783 | tags=38%, list=8%, signal=41% |
| 968 | GO\_T\_CELL\_ACTIVATION\_INVOLVED\_IN\_IMMUNE\_RESPONSE |  | 59 | 0.52 | 1.24 | 0.222 | 0.378 | 1.000 | 3406 | tags=39%, list=16%, signal=46% |
| 969 | GO\_REGULATION\_OF\_REPRODUCTIVE\_PROCESS |  | 119 | 0.39 | 1.24 | 0.093 | 0.377 | 1.000 | 3584 | tags=26%, list=16%, signal=31% |
| 970 | GO\_POSITIVE\_REGULATION\_OF\_ALPHA\_BETA\_T\_CELL\_ACTIVATION |  | 51 | 0.55 | 1.24 | 0.201 | 0.377 | 1.000 | 3241 | tags=37%, list=15%, signal=44% |
| 971 | GO\_PHOSPHATIDYLCHOLINE\_METABOLIC\_PROCESS |  | 61 | 0.46 | 1.24 | 0.161 | 0.378 | 1.000 | 3891 | tags=28%, list=18%, signal=34% |
| 972 | GO\_RESPONSE\_TO\_KETONE |  | 174 | 0.40 | 1.24 | 0.076 | 0.378 | 1.000 | 2188 | tags=22%, list=10%, signal=24% |
| 973 | GO\_MAST\_CELL\_ACTIVATION |  | 21 | 0.60 | 1.24 | 0.203 | 0.377 | 1.000 | 2920 | tags=43%, list=13%, signal=49% |
| 974 | GO\_CARDIAC\_RIGHT\_VENTRICLE\_MORPHOGENESIS |  | 16 | 0.57 | 1.24 | 0.191 | 0.377 | 1.000 | 3058 | tags=31%, list=14%, signal=36% |
| 975 | GO\_REGULATION\_OF\_LYMPHOCYTE\_MIGRATION |  | 34 | 0.59 | 1.24 | 0.205 | 0.378 | 1.000 | 1783 | tags=32%, list=8%, signal=35% |
| 976 | GO\_BEHAVIOR |  | 487 | 0.34 | 1.24 | 0.075 | 0.377 | 1.000 | 3347 | tags=18%, list=15%, signal=21% |
| 977 | GO\_CENTRAL\_NERVOUS\_SYSTEM\_NEURON\_DIFFERENTIATION |  | 161 | 0.39 | 1.24 | 0.131 | 0.378 | 1.000 | 3139 | tags=20%, list=14%, signal=24% |
| 978 | GO\_PERIPHERAL\_NERVOUS\_SYSTEM\_AXON\_ENSHEATHMENT |  | 21 | 0.46 | 1.24 | 0.197 | 0.378 | 1.000 | 4266 | tags=38%, list=20%, signal=47% |
| 979 | GO\_ENDOTHELIUM\_DEVELOPMENT |  | 88 | 0.41 | 1.24 | 0.158 | 0.378 | 1.000 | 3508 | tags=36%, list=16%, signal=43% |
| 980 | GO\_REGULATION\_OF\_INTERLEUKIN\_17\_PRODUCTION |  | 22 | 0.61 | 1.24 | 0.206 | 0.378 | 1.000 | 804 | tags=27%, list=4%, signal=28% |
| 981 | GO\_SENSORY\_PERCEPTION\_OF\_MECHANICAL\_STIMULUS |  | 137 | 0.35 | 1.24 | 0.085 | 0.378 | 1.000 | 3230 | tags=21%, list=15%, signal=25% |
| 982 | GO\_REGULATION\_OF\_RECEPTOR\_INTERNALIZATION |  | 35 | 0.43 | 1.24 | 0.146 | 0.379 | 1.000 | 3561 | tags=37%, list=16%, signal=44% |
| 983 | GO\_REGULATION\_OF\_SMOOTHENED\_SIGNALING\_PATHWAY |  | 61 | 0.50 | 1.24 | 0.186 | 0.379 | 1.000 | 3280 | tags=31%, list=15%, signal=37% |
| 984 | GO\_REGULATION\_OF\_T\_CELL\_CYTOKINE\_PRODUCTION |  | 22 | 0.58 | 1.24 | 0.208 | 0.378 | 1.000 | 1983 | tags=27%, list=9%, signal=30% |
| 985 | GO\_PROTEIN\_AUTOPHOSPHORYLATION |  | 188 | 0.36 | 1.24 | 0.141 | 0.378 | 1.000 | 3652 | tags=28%, list=17%, signal=34% |
| 986 | GO\_POSITIVE\_REGULATION\_OF\_ACTIN\_NUCLEATION |  | 16 | 0.47 | 1.24 | 0.193 | 0.378 | 1.000 | 3580 | tags=31%, list=16%, signal=37% |
| 987 | GO\_REGULATION\_OF\_VASCULAR\_PERMEABILITY |  | 30 | 0.47 | 1.23 | 0.168 | 0.378 | 1.000 | 2122 | tags=27%, list=10%, signal=30% |
| 988 | GO\_REGULATION\_OF\_TISSUE\_REMODELING |  | 58 | 0.44 | 1.23 | 0.150 | 0.378 | 1.000 | 2042 | tags=17%, list=9%, signal=19% |
| 989 | GO\_SIGNAL\_PEPTIDE\_PROCESSING |  | 22 | 0.46 | 1.23 | 0.193 | 0.379 | 1.000 | 4564 | tags=36%, list=21%, signal=46% |
| 990 | GO\_MULTICELLULAR\_ORGANISMAL\_RESPONSE\_TO\_STRESS |  | 66 | 0.41 | 1.23 | 0.104 | 0.379 | 1.000 | 2406 | tags=17%, list=11%, signal=19% |
| 991 | GO\_POSITIVE\_REGULATION\_OF\_ALPHA\_BETA\_T\_CELL\_PROLIFERATION |  | 19 | 0.65 | 1.23 | 0.245 | 0.380 | 1.000 | 4288 | tags=58%, list=20%, signal=72% |
| 992 | GO\_CELL\_VOLUME\_HOMEOSTASIS |  | 26 | 0.49 | 1.23 | 0.216 | 0.380 | 1.000 | 2965 | tags=27%, list=14%, signal=31% |
| 993 | GO\_POSITIVE\_REGULATION\_OF\_DEVELOPMENTAL\_GROWTH |  | 149 | 0.35 | 1.23 | 0.084 | 0.379 | 1.000 | 3024 | tags=21%, list=14%, signal=25% |
| 994 | GO\_RENAL\_TUBULE\_DEVELOPMENT |  | 77 | 0.45 | 1.23 | 0.137 | 0.379 | 1.000 | 3267 | tags=30%, list=15%, signal=35% |
| 995 | GO\_REGULATION\_OF\_ENDOPLASMIC\_RETICULUM\_STRESS\_INDUCED\_INTRINSIC\_APOPTOTIC\_SIGNALING\_PATHWAY |  | 25 | 0.44 | 1.23 | 0.226 | 0.380 | 1.000 | 4839 | tags=36%, list=22%, signal=46% |
| 996 | GO\_MESENCHYMAL\_CELL\_DIFFERENTIATION |  | 132 | 0.44 | 1.23 | 0.135 | 0.381 | 1.000 | 3408 | tags=30%, list=16%, signal=35% |
| 997 | GO\_REGULATION\_OF\_PROTEIN\_KINASE\_B\_SIGNALING |  | 114 | 0.40 | 1.23 | 0.096 | 0.381 | 1.000 | 3447 | tags=33%, list=16%, signal=39% |
| 998 | GO\_NEGATIVE\_REGULATION\_OF\_WNT\_SIGNALING\_PATHWAY |  | 186 | 0.37 | 1.23 | 0.142 | 0.382 | 1.000 | 3221 | tags=20%, list=15%, signal=24% |
| 999 | GO\_POSITIVE\_REGULATION\_OF\_RESPONSE\_TO\_EXTERNAL\_STIMULUS |  | 283 | 0.44 | 1.23 | 0.162 | 0.382 | 1.000 | 3474 | tags=33%, list=16%, signal=38% |
| 1000 | GO\_REGULATION\_OF\_NITRIC\_OXIDE\_BIOSYNTHETIC\_PROCESS |  | 51 | 0.49 | 1.23 | 0.131 | 0.382 | 1.000 | 3170 | tags=41%, list=15%, signal=48% |
| 1001 | GO\_DENDRITE\_MORPHOGENESIS |  | 41 | 0.45 | 1.23 | 0.189 | 0.384 | 1.000 | 3530 | tags=24%, list=16%, signal=29% |
| 1002 | GO\_POSITIVE\_REGULATION\_OF\_CYTOKINE\_PRODUCTION\_INVOLVED\_IN\_IMMUNE\_RESPONSE |  | 28 | 0.55 | 1.23 | 0.197 | 0.384 | 1.000 | 2428 | tags=32%, list=11%, signal=36% |
| 1003 | GO\_POSITIVE\_REGULATION\_OF\_FATTY\_ACID\_METABOLIC\_PROCESS |  | 33 | 0.48 | 1.23 | 0.160 | 0.384 | 1.000 | 2376 | tags=24%, list=11%, signal=27% |
| 1004 | GO\_REGULATION\_OF\_STEM\_CELL\_DIFFERENTIATION |  | 109 | 0.39 | 1.23 | 0.141 | 0.385 | 1.000 | 3640 | tags=29%, list=17%, signal=35% |
| 1005 | GO\_NEGATIVE\_REGULATION\_OF\_TRANSFORMING\_GROWTH\_FACTOR\_BETA\_RECEPTOR\_SIGNALING\_PATHWAY |  | 63 | 0.45 | 1.23 | 0.202 | 0.385 | 1.000 | 3619 | tags=25%, list=17%, signal=30% |
| 1006 | GO\_WNT\_SIGNALING\_PATHWAY |  | 327 | 0.33 | 1.23 | 0.119 | 0.385 | 1.000 | 3058 | tags=18%, list=14%, signal=21% |
| 1007 | GO\_NEGATIVE\_REGULATION\_OF\_NEUROLOGICAL\_SYSTEM\_PROCESS |  | 15 | 0.51 | 1.23 | 0.165 | 0.386 | 1.000 | 4365 | tags=40%, list=20%, signal=50% |
| 1008 | GO\_POSITIVE\_REGULATION\_OF\_PROTEIN\_DEPOLYMERIZATION |  | 16 | 0.61 | 1.23 | 0.237 | 0.386 | 1.000 | 1358 | tags=25%, list=6%, signal=27% |
| 1009 | GO\_FC\_RECEPTOR\_SIGNALING\_PATHWAY |  | 181 | 0.38 | 1.23 | 0.210 | 0.386 | 1.000 | 2932 | tags=19%, list=13%, signal=22% |
| 1010 | GO\_MYELIN\_ASSEMBLY |  | 15 | 0.52 | 1.23 | 0.196 | 0.387 | 1.000 | 735 | tags=13%, list=3%, signal=14% |
| 1011 | GO\_ACTIVATION\_OF\_ADENYLATE\_CYCLASE\_ACTIVITY |  | 37 | 0.42 | 1.23 | 0.188 | 0.387 | 1.000 | 3691 | tags=27%, list=17%, signal=32% |
| 1012 | GO\_CEREBRAL\_CORTEX\_RADIALLY\_ORIENTED\_CELL\_MIGRATION |  | 27 | 0.41 | 1.23 | 0.206 | 0.386 | 1.000 | 3310 | tags=22%, list=15%, signal=26% |
| 1013 | GO\_NEGATIVE\_REGULATION\_OF\_INTRINSIC\_APOPTOTIC\_SIGNALING\_PATHWAY\_IN\_RESPONSE\_TO\_DNA\_DAMAGE |  | 23 | 0.47 | 1.23 | 0.161 | 0.386 | 1.000 | 603 | tags=13%, list=3%, signal=13% |
| 1014 | GO\_REGULATION\_OF\_HEART\_GROWTH |  | 41 | 0.48 | 1.23 | 0.171 | 0.386 | 1.000 | 2451 | tags=27%, list=11%, signal=30% |
| 1015 | GO\_SUBSTANTIA\_NIGRA\_DEVELOPMENT |  | 43 | 0.42 | 1.23 | 0.165 | 0.386 | 1.000 | 2261 | tags=16%, list=10%, signal=18% |
| 1016 | GO\_PHOSPHATIDYLINOSITOL\_3\_PHOSPHATE\_BIOSYNTHETIC\_PROCESS |  | 49 | 0.41 | 1.22 | 0.163 | 0.386 | 1.000 | 2907 | tags=20%, list=13%, signal=24% |
| 1017 | GO\_POSITIVE\_REGULATION\_OF\_T\_CELL\_PROLIFERATION |  | 92 | 0.53 | 1.22 | 0.215 | 0.386 | 1.000 | 2290 | tags=30%, list=11%, signal=34% |
| 1018 | GO\_POSITIVE\_REGULATION\_OF\_ACTIVATED\_T\_CELL\_PROLIFERATION |  | 27 | 0.51 | 1.22 | 0.184 | 0.386 | 1.000 | 2231 | tags=26%, list=10%, signal=29% |
| 1019 | GO\_MONOCYTE\_DIFFERENTIATION |  | 16 | 0.50 | 1.22 | 0.164 | 0.386 | 1.000 | 543 | tags=19%, list=2%, signal=19% |
| 1020 | GO\_SEGMENTATION |  | 85 | 0.41 | 1.22 | 0.113 | 0.387 | 1.000 | 3353 | tags=27%, list=15%, signal=32% |
| 1021 | GO\_REGULATION\_OF\_DENDRITE\_MORPHOGENESIS |  | 71 | 0.37 | 1.22 | 0.145 | 0.388 | 1.000 | 2694 | tags=21%, list=12%, signal=24% |
| 1022 | GO\_SMALL\_GTPASE\_MEDIATED\_SIGNAL\_TRANSDUCTION |  | 329 | 0.32 | 1.22 | 0.089 | 0.388 | 1.000 | 3447 | tags=22%, list=16%, signal=26% |
| 1023 | GO\_ENDODERMAL\_CELL\_DIFFERENTIATION |  | 38 | 0.55 | 1.22 | 0.243 | 0.388 | 1.000 | 3310 | tags=37%, list=15%, signal=43% |
| 1024 | GO\_POSITIVE\_REGULATION\_OF\_PHAGOCYTOSIS |  | 44 | 0.47 | 1.22 | 0.199 | 0.390 | 1.000 | 3800 | tags=43%, list=17%, signal=52% |
| 1025 | GO\_PHOSPHATIDYLINOSITOL\_DEPHOSPHORYLATION |  | 21 | 0.44 | 1.22 | 0.219 | 0.390 | 1.000 | 4387 | tags=38%, list=20%, signal=48% |
| 1026 | GO\_RESPONSE\_TO\_PAIN |  | 28 | 0.47 | 1.22 | 0.179 | 0.390 | 1.000 | 1367 | tags=14%, list=6%, signal=15% |
| 1027 | GO\_NEGATIVE\_REGULATION\_OF\_ALPHA\_BETA\_T\_CELL\_ACTIVATION |  | 23 | 0.53 | 1.22 | 0.212 | 0.390 | 1.000 | 3176 | tags=35%, list=15%, signal=41% |
| 1028 | GO\_RETINA\_DEVELOPMENT\_IN\_CAMERA\_TYPE\_EYE |  | 126 | 0.39 | 1.22 | 0.186 | 0.390 | 1.000 | 3652 | tags=25%, list=17%, signal=29% |
| 1029 | GO\_NEGATIVE\_REGULATION\_OF\_POTASSIUM\_ION\_TRANSMEMBRANE\_TRANSPORTER\_ACTIVITY |  | 16 | 0.55 | 1.22 | 0.240 | 0.392 | 1.000 | 716 | tags=19%, list=3%, signal=19% |
| 1030 | GO\_RESPONSE\_TO\_PURINE\_CONTAINING\_COMPOUND |  | 153 | 0.38 | 1.22 | 0.106 | 0.393 | 1.000 | 2142 | tags=20%, list=10%, signal=22% |
| 1031 | GO\_RESPONSE\_TO\_CARBOHYDRATE |  | 162 | 0.34 | 1.22 | 0.130 | 0.394 | 1.000 | 4170 | tags=30%, list=19%, signal=37% |
| 1032 | GO\_MYELOID\_LEUKOCYTE\_DIFFERENTIATION |  | 94 | 0.44 | 1.22 | 0.181 | 0.394 | 1.000 | 3049 | tags=32%, list=14%, signal=37% |
| 1033 | GO\_REGULATION\_OF\_DENDRITIC\_SPINE\_DEVELOPMENT |  | 53 | 0.40 | 1.22 | 0.136 | 0.394 | 1.000 | 2694 | tags=25%, list=12%, signal=28% |
| 1034 | GO\_REGULATION\_OF\_ORGAN\_GROWTH |  | 70 | 0.43 | 1.22 | 0.125 | 0.394 | 1.000 | 3221 | tags=29%, list=15%, signal=33% |
| 1035 | GO\_RESPONSE\_TO\_INSULIN |  | 198 | 0.33 | 1.22 | 0.098 | 0.394 | 1.000 | 3302 | tags=21%, list=15%, signal=25% |
| 1036 | GO\_NEGATIVE\_REGULATION\_OF\_CELL\_MATRIX\_ADHESION |  | 28 | 0.49 | 1.22 | 0.169 | 0.395 | 1.000 | 1465 | tags=25%, list=7%, signal=27% |
| 1037 | GO\_NEGATIVE\_CHEMOTAXIS |  | 38 | 0.46 | 1.22 | 0.166 | 0.397 | 1.000 | 878 | tags=13%, list=4%, signal=14% |
| 1038 | GO\_NEGATIVE\_REGULATION\_OF\_T\_CELL\_APOPTOTIC\_PROCESS |  | 16 | 0.60 | 1.22 | 0.201 | 0.397 | 1.000 | 2183 | tags=50%, list=10%, signal=56% |
| 1039 | GO\_REGULATION\_OF\_PH |  | 84 | 0.38 | 1.21 | 0.153 | 0.397 | 1.000 | 4406 | tags=29%, list=20%, signal=36% |
| 1040 | GO\_ANION\_HOMEOSTASIS |  | 40 | 0.42 | 1.21 | 0.124 | 0.397 | 1.000 | 2478 | tags=18%, list=11%, signal=20% |
| 1041 | GO\_REGULATION\_OF\_INTERLEUKIN\_13\_PRODUCTION |  | 17 | 0.57 | 1.21 | 0.179 | 0.398 | 1.000 | 4288 | tags=65%, list=20%, signal=81% |
| 1042 | GO\_POSITIVE\_REGULATION\_OF\_STEM\_CELL\_DIFFERENTIATION |  | 49 | 0.47 | 1.21 | 0.189 | 0.398 | 1.000 | 3459 | tags=41%, list=16%, signal=48% |
| 1043 | GO\_REGULATION\_OF\_IMMUNE\_EFFECTOR\_PROCESS |  | 400 | 0.42 | 1.21 | 0.174 | 0.397 | 1.000 | 3478 | tags=28%, list=16%, signal=33% |
| 1044 | GO\_HINDLIMB\_MORPHOGENESIS |  | 36 | 0.47 | 1.21 | 0.176 | 0.398 | 1.000 | 3578 | tags=31%, list=16%, signal=37% |
| 1045 | GO\_NEGATIVE\_REGULATION\_OF\_INTERFERON\_GAMMA\_PRODUCTION |  | 32 | 0.55 | 1.21 | 0.196 | 0.398 | 1.000 | 3783 | tags=47%, list=17%, signal=57% |
| 1046 | GO\_POSITIVE\_REGULATION\_OF\_INTERFERON\_GAMMA\_PRODUCTION |  | 59 | 0.57 | 1.21 | 0.251 | 0.399 | 1.000 | 2059 | tags=34%, list=9%, signal=37% |
| 1047 | GO\_NON\_CANONICAL\_WNT\_SIGNALING\_PATHWAY |  | 131 | 0.35 | 1.21 | 0.168 | 0.399 | 1.000 | 2641 | tags=18%, list=12%, signal=20% |
| 1048 | GO\_PHOSPHOLIPID\_DEPHOSPHORYLATION |  | 22 | 0.43 | 1.21 | 0.177 | 0.399 | 1.000 | 4387 | tags=36%, list=20%, signal=46% |
| 1049 | GO\_NEGATIVE\_REGULATION\_OF\_CYTOKINE\_PRODUCTION\_INVOLVED\_IN\_IMMUNE\_RESPONSE |  | 22 | 0.57 | 1.21 | 0.232 | 0.399 | 1.000 | 3473 | tags=50%, list=16%, signal=59% |
| 1050 | GO\_REGULATION\_OF\_PLASMA\_MEMBRANE\_ORGANIZATION |  | 69 | 0.38 | 1.21 | 0.112 | 0.399 | 1.000 | 1158 | tags=13%, list=5%, signal=14% |
| 1051 | GO\_ENDOCHONDRAL\_BONE\_MORPHOGENESIS |  | 44 | 0.49 | 1.21 | 0.218 | 0.399 | 1.000 | 2956 | tags=36%, list=14%, signal=42% |
| 1052 | GO\_POSITIVE\_REGULATION\_OF\_PATHWAY\_RESTRICTED\_SMAD\_PROTEIN\_PHOSPHORYLATION |  | 45 | 0.44 | 1.21 | 0.205 | 0.398 | 1.000 | 1687 | tags=16%, list=8%, signal=17% |
| 1053 | GO\_RESPONSE\_TO\_TRANSITION\_METAL\_NANOPARTICLE |  | 141 | 0.35 | 1.21 | 0.131 | 0.398 | 1.000 | 3298 | tags=21%, list=15%, signal=25% |
| 1054 | GO\_PROTEIN\_KINASE\_B\_SIGNALING |  | 33 | 0.47 | 1.21 | 0.146 | 0.398 | 1.000 | 4170 | tags=48%, list=19%, signal=60% |
| 1055 | GO\_HORMONE\_MEDIATED\_SIGNALING\_PATHWAY |  | 154 | 0.33 | 1.21 | 0.133 | 0.398 | 1.000 | 3533 | tags=18%, list=16%, signal=22% |
| 1056 | GO\_REGULATION\_OF\_WNT\_SIGNALING\_PATHWAY |  | 292 | 0.35 | 1.21 | 0.139 | 0.398 | 1.000 | 3267 | tags=21%, list=15%, signal=24% |
| 1057 | GO\_REGULATION\_OF\_GRANULOCYTE\_CHEMOTAXIS |  | 37 | 0.57 | 1.21 | 0.228 | 0.401 | 1.000 | 3080 | tags=49%, list=14%, signal=57% |
| 1058 | GO\_REGULATION\_OF\_GLUCONEOGENESIS |  | 33 | 0.45 | 1.21 | 0.138 | 0.400 | 1.000 | 1801 | tags=24%, list=8%, signal=26% |
| 1059 | GO\_REGULATION\_OF\_OSTEOBLAST\_PROLIFERATION |  | 22 | 0.54 | 1.21 | 0.187 | 0.401 | 1.000 | 3138 | tags=41%, list=14%, signal=48% |
| 1060 | GO\_PROTEIN\_LOCALIZATION\_TO\_MEMBRANE |  | 350 | 0.28 | 1.21 | 0.086 | 0.401 | 1.000 | 3125 | tags=15%, list=14%, signal=17% |
| 1061 | GO\_EXCITATORY\_POSTSYNAPTIC\_POTENTIAL |  | 27 | 0.47 | 1.21 | 0.221 | 0.401 | 1.000 | 3913 | tags=26%, list=18%, signal=32% |
| 1062 | GO\_EXPLORATION\_BEHAVIOR |  | 23 | 0.47 | 1.21 | 0.221 | 0.403 | 1.000 | 1288 | tags=13%, list=6%, signal=14% |
| 1063 | GO\_NEGATIVE\_REGULATION\_OF\_HOMOTYPIC\_CELL\_CELL\_ADHESION |  | 97 | 0.45 | 1.21 | 0.192 | 0.403 | 1.000 | 3457 | tags=34%, list=16%, signal=40% |
| 1064 | GO\_STEROID\_HORMONE\_MEDIATED\_SIGNALING\_PATHWAY |  | 121 | 0.34 | 1.21 | 0.140 | 0.403 | 1.000 | 2956 | tags=17%, list=14%, signal=19% |
| 1065 | GO\_LIPID\_MODIFICATION |  | 192 | 0.34 | 1.21 | 0.100 | 0.404 | 1.000 | 4695 | tags=33%, list=22%, signal=42% |
| 1066 | GO\_REGULATION\_OF\_CALCIUM\_ION\_DEPENDENT\_EXOCYTOSIS |  | 77 | 0.36 | 1.20 | 0.127 | 0.406 | 1.000 | 2081 | tags=10%, list=10%, signal=11% |
| 1067 | GO\_SCHWANN\_CELL\_DEVELOPMENT |  | 25 | 0.43 | 1.20 | 0.225 | 0.406 | 1.000 | 4887 | tags=44%, list=22%, signal=57% |
| 1068 | GO\_MACROAUTOPHAGY |  | 258 | 0.28 | 1.20 | 0.096 | 0.406 | 1.000 | 2888 | tags=16%, list=13%, signal=18% |
| 1069 | GO\_TRIGLYCERIDE\_CATABOLIC\_PROCESS |  | 19 | 0.54 | 1.20 | 0.185 | 0.406 | 1.000 | 2784 | tags=32%, list=13%, signal=36% |
| 1070 | GO\_REGULATION\_OF\_STEROID\_HORMONE\_SECRETION |  | 20 | 0.46 | 1.20 | 0.224 | 0.406 | 1.000 | 2862 | tags=20%, list=13%, signal=23% |
| 1071 | GO\_RECEPTOR\_CLUSTERING |  | 40 | 0.43 | 1.20 | 0.196 | 0.406 | 1.000 | 2259 | tags=23%, list=10%, signal=25% |
| 1072 | GO\_PATTERN\_RECOGNITION\_RECEPTOR\_SIGNALING\_PATHWAY |  | 105 | 0.47 | 1.20 | 0.242 | 0.406 | 1.000 | 2680 | tags=24%, list=12%, signal=27% |
| 1073 | GO\_SENSORY\_ORGAN\_DEVELOPMENT |  | 471 | 0.36 | 1.20 | 0.156 | 0.407 | 1.000 | 2430 | tags=18%, list=11%, signal=19% |
| 1074 | GO\_NEURON\_PROJECTION\_GUIDANCE |  | 187 | 0.40 | 1.20 | 0.160 | 0.407 | 1.000 | 2381 | tags=18%, list=11%, signal=20% |
| 1075 | GO\_BONE\_RESORPTION |  | 21 | 0.49 | 1.20 | 0.224 | 0.407 | 1.000 | 3379 | tags=33%, list=16%, signal=39% |
| 1076 | GO\_HYDROGEN\_TRANSPORT |  | 122 | 0.35 | 1.20 | 0.203 | 0.406 | 1.000 | 4046 | tags=20%, list=19%, signal=25% |
| 1077 | GO\_POSITIVE\_REGULATION\_OF\_T\_HELPER\_1\_TYPE\_IMMUNE\_RESPONSE |  | 15 | 0.59 | 1.20 | 0.232 | 0.407 | 1.000 | 771 | tags=20%, list=4%, signal=21% |
| 1078 | GO\_POSITIVE\_REGULATION\_OF\_RESPONSE\_TO\_WOUNDING |  | 152 | 0.46 | 1.20 | 0.193 | 0.407 | 1.000 | 3129 | tags=30%, list=14%, signal=35% |
| 1079 | GO\_REGULATION\_OF\_MACROPHAGE\_CHEMOTAXIS |  | 16 | 0.62 | 1.20 | 0.228 | 0.409 | 1.000 | 3170 | tags=63%, list=15%, signal=73% |
| 1080 | GO\_REGULATION\_OF\_RESPIRATORY\_GASEOUS\_EXCHANGE |  | 22 | 0.46 | 1.20 | 0.206 | 0.409 | 1.000 | 1998 | tags=14%, list=9%, signal=15% |
| 1081 | GO\_REGULATION\_OF\_CARDIAC\_MUSCLE\_CELL\_ACTION\_POTENTIAL |  | 19 | 0.48 | 1.20 | 0.194 | 0.409 | 1.000 | 2028 | tags=26%, list=9%, signal=29% |
| 1082 | GO\_VASCULOGENESIS |  | 58 | 0.44 | 1.20 | 0.210 | 0.409 | 1.000 | 1742 | tags=24%, list=8%, signal=26% |
| 1083 | GO\_REGULATION\_OF\_EPITHELIAL\_TO\_MESENCHYMAL\_TRANSITION |  | 64 | 0.43 | 1.20 | 0.170 | 0.409 | 1.000 | 3640 | tags=33%, list=17%, signal=39% |
| 1084 | GO\_MONOCARBOXYLIC\_ACID\_BIOSYNTHETIC\_PROCESS |  | 155 | 0.39 | 1.20 | 0.137 | 0.409 | 1.000 | 2478 | tags=20%, list=11%, signal=22% |
| 1085 | GO\_REGULATION\_OF\_MACROPHAGE\_ACTIVATION |  | 22 | 0.50 | 1.20 | 0.181 | 0.409 | 1.000 | 2059 | tags=32%, list=9%, signal=35% |
| 1086 | GO\_POLYSACCHARIDE\_METABOLIC\_PROCESS |  | 75 | 0.40 | 1.20 | 0.180 | 0.409 | 1.000 | 4609 | tags=36%, list=21%, signal=46% |
| 1087 | GO\_CELL\_CELL\_ADHESION\_VIA\_PLASMA\_MEMBRANE\_ADHESION\_MOLECULES |  | 170 | 0.39 | 1.20 | 0.128 | 0.409 | 1.000 | 2593 | tags=19%, list=12%, signal=21% |
| 1088 | GO\_PROTEIN\_LOCALIZATION\_TO\_CELL\_PERIPHERY |  | 149 | 0.34 | 1.20 | 0.125 | 0.409 | 1.000 | 3906 | tags=26%, list=18%, signal=32% |
| 1089 | GO\_ALDITOL\_PHOSPHATE\_METABOLIC\_PROCESS |  | 33 | 0.51 | 1.20 | 0.221 | 0.409 | 1.000 | 4428 | tags=36%, list=20%, signal=46% |
| 1090 | GO\_REGULATION\_OF\_CELL\_JUNCTION\_ASSEMBLY |  | 67 | 0.41 | 1.20 | 0.199 | 0.409 | 1.000 | 3757 | tags=33%, list=17%, signal=40% |
| 1091 | GO\_EAR\_DEVELOPMENT |  | 185 | 0.39 | 1.20 | 0.166 | 0.409 | 1.000 | 3280 | tags=25%, list=15%, signal=29% |
| 1092 | GO\_REGULATION\_OF\_INTERLEUKIN\_6\_PRODUCTION |  | 95 | 0.46 | 1.20 | 0.220 | 0.410 | 1.000 | 3107 | tags=35%, list=14%, signal=40% |
| 1093 | GO\_AMINOGLYCAN\_BIOSYNTHETIC\_PROCESS |  | 104 | 0.46 | 1.20 | 0.204 | 0.411 | 1.000 | 3164 | tags=29%, list=15%, signal=34% |
| 1094 | GO\_CELLULAR\_RESPONSE\_TO\_REACTIVE\_NITROGEN\_SPECIES |  | 19 | 0.46 | 1.20 | 0.227 | 0.411 | 1.000 | 1122 | tags=21%, list=5%, signal=22% |
| 1095 | GO\_ATRIOVENTRICULAR\_VALVE\_MORPHOGENESIS |  | 16 | 0.59 | 1.20 | 0.284 | 0.413 | 1.000 | 2974 | tags=44%, list=14%, signal=51% |
| 1096 | GO\_PHOSPHATIDYLSERINE\_METABOLIC\_PROCESS |  | 26 | 0.50 | 1.20 | 0.203 | 0.413 | 1.000 | 4428 | tags=35%, list=20%, signal=43% |
| 1097 | GO\_REGULATION\_OF\_EPITHELIAL\_CELL\_PROLIFERATION |  | 272 | 0.38 | 1.20 | 0.133 | 0.413 | 1.000 | 3614 | tags=29%, list=17%, signal=35% |
| 1098 | GO\_REGULATION\_OF\_ACUTE\_INFLAMMATORY\_RESPONSE |  | 67 | 0.48 | 1.19 | 0.222 | 0.414 | 1.000 | 3241 | tags=39%, list=15%, signal=45% |
| 1099 | GO\_POSITIVE\_REGULATION\_OF\_INTERLEUKIN\_10\_PRODUCTION |  | 28 | 0.53 | 1.19 | 0.246 | 0.414 | 1.000 | 2764 | tags=32%, list=13%, signal=37% |
| 1100 | GO\_FATTY\_ACID\_DERIVATIVE\_BIOSYNTHETIC\_PROCESS |  | 41 | 0.50 | 1.19 | 0.218 | 0.414 | 1.000 | 1924 | tags=27%, list=9%, signal=29% |
| 1101 | GO\_POSITIVE\_REGULATION\_OF\_DEPHOSPHORYLATION |  | 42 | 0.44 | 1.19 | 0.196 | 0.414 | 1.000 | 3084 | tags=31%, list=14%, signal=36% |
| 1102 | GO\_SENSORY\_PERCEPTION\_OF\_TEMPERATURE\_STIMULUS |  | 19 | 0.49 | 1.19 | 0.217 | 0.414 | 1.000 | 502 | tags=11%, list=2%, signal=11% |
| 1103 | GO\_NEGATIVE\_REGULATION\_OF\_ENDOTHELIAL\_CELL\_APOPTOTIC\_PROCESS |  | 27 | 0.52 | 1.19 | 0.231 | 0.415 | 1.000 | 2912 | tags=41%, list=13%, signal=47% |
| 1104 | GO\_REGULATION\_OF\_T\_CELL\_PROLIFERATION |  | 141 | 0.47 | 1.19 | 0.218 | 0.415 | 1.000 | 3526 | tags=35%, list=16%, signal=41% |
| 1105 | GO\_GANGLIOSIDE\_BIOSYNTHETIC\_PROCESS |  | 17 | 0.51 | 1.19 | 0.220 | 0.415 | 1.000 | 4738 | tags=59%, list=22%, signal=75% |
| 1106 | GO\_MULTICELLULAR\_ORGANISM\_METABOLIC\_PROCESS |  | 87 | 0.50 | 1.19 | 0.230 | 0.415 | 1.000 | 2647 | tags=39%, list=12%, signal=44% |
| 1107 | GO\_REGULATION\_OF\_INFLAMMATORY\_RESPONSE\_TO\_ANTIGENIC\_STIMULUS |  | 17 | 0.58 | 1.19 | 0.238 | 0.417 | 1.000 | 4293 | tags=59%, list=20%, signal=73% |
| 1108 | GO\_CELLULAR\_RESPONSE\_TO\_GAMMA\_RADIATION |  | 19 | 0.48 | 1.19 | 0.229 | 0.416 | 1.000 | 1869 | tags=21%, list=9%, signal=23% |
| 1109 | GO\_NEGATIVE\_REGULATION\_OF\_ENDOCYTOSIS |  | 37 | 0.39 | 1.19 | 0.237 | 0.416 | 1.000 | 1725 | tags=14%, list=8%, signal=15% |
| 1110 | GO\_REGULATION\_OF\_PEPTIDE\_TRANSPORT |  | 244 | 0.32 | 1.19 | 0.084 | 0.417 | 1.000 | 3814 | tags=23%, list=18%, signal=27% |
| 1111 | GO\_NEGATIVE\_REGULATION\_OF\_B\_CELL\_PROLIFERATION |  | 15 | 0.55 | 1.19 | 0.254 | 0.417 | 1.000 | 3025 | tags=47%, list=14%, signal=54% |
| 1112 | GO\_POTASSIUM\_ION\_IMPORT |  | 26 | 0.43 | 1.19 | 0.235 | 0.418 | 1.000 | 339 | tags=8%, list=2%, signal=8% |
| 1113 | GO\_RECEPTOR\_METABOLIC\_PROCESS |  | 75 | 0.39 | 1.19 | 0.166 | 0.418 | 1.000 | 3131 | tags=29%, list=14%, signal=34% |
| 1114 | GO\_REGULATION\_OF\_ALPHA\_BETA\_T\_CELL\_DIFFERENTIATION |  | 46 | 0.50 | 1.19 | 0.270 | 0.418 | 1.000 | 3526 | tags=37%, list=16%, signal=44% |
| 1115 | GO\_CELLULAR\_RESPONSE\_TO\_OXIDATIVE\_STRESS |  | 177 | 0.34 | 1.19 | 0.134 | 0.418 | 1.000 | 2966 | tags=22%, list=14%, signal=25% |
| 1116 | GO\_ENDOMEMBRANE\_SYSTEM\_ORGANIZATION |  | 445 | 0.28 | 1.19 | 0.091 | 0.418 | 1.000 | 3370 | tags=18%, list=15%, signal=20% |
| 1117 | GO\_NEGATIVE\_REGULATION\_OF\_CELL\_CELL\_ADHESION |  | 131 | 0.41 | 1.19 | 0.180 | 0.418 | 1.000 | 3457 | tags=31%, list=16%, signal=37% |
| 1118 | GO\_SULFUR\_COMPOUND\_BIOSYNTHETIC\_PROCESS |  | 188 | 0.38 | 1.19 | 0.174 | 0.418 | 1.000 | 3863 | tags=25%, list=18%, signal=30% |
| 1119 | GO\_INOSITOL\_PHOSPHATE\_METABOLIC\_PROCESS |  | 55 | 0.35 | 1.19 | 0.151 | 0.418 | 1.000 | 4387 | tags=38%, list=20%, signal=48% |
| 1120 | GO\_REGULATION\_OF\_ACTION\_POTENTIAL |  | 37 | 0.41 | 1.19 | 0.200 | 0.418 | 1.000 | 3825 | tags=30%, list=18%, signal=36% |
| 1121 | GO\_ACYLGLYCEROL\_HOMEOSTASIS |  | 28 | 0.44 | 1.19 | 0.196 | 0.417 | 1.000 | 3222 | tags=25%, list=15%, signal=29% |
| 1122 | GO\_CARDIAC\_EPITHELIAL\_TO\_MESENCHYMAL\_TRANSITION |  | 24 | 0.55 | 1.19 | 0.231 | 0.419 | 1.000 | 2974 | tags=38%, list=14%, signal=43% |
| 1123 | GO\_OSTEOBLAST\_DIFFERENTIATION |  | 118 | 0.41 | 1.19 | 0.221 | 0.420 | 1.000 | 3001 | tags=24%, list=14%, signal=27% |
| 1124 | GO\_REPLACEMENT\_OSSIFICATION |  | 25 | 0.52 | 1.19 | 0.226 | 0.420 | 1.000 | 2956 | tags=44%, list=14%, signal=51% |
| 1125 | GO\_REGULATION\_OF\_MYELOID\_CELL\_APOPTOTIC\_PROCESS |  | 21 | 0.51 | 1.19 | 0.225 | 0.419 | 1.000 | 3222 | tags=43%, list=15%, signal=50% |
| 1126 | GO\_FORELIMB\_MORPHOGENESIS |  | 37 | 0.45 | 1.19 | 0.207 | 0.419 | 1.000 | 2430 | tags=24%, list=11%, signal=27% |
| 1127 | GO\_EMBRYONIC\_FORELIMB\_MORPHOGENESIS |  | 29 | 0.47 | 1.19 | 0.225 | 0.419 | 1.000 | 2430 | tags=28%, list=11%, signal=31% |
| 1128 | GO\_WNT\_SIGNALING\_PATHWAY\_CALCIUM\_MODULATING\_PATHWAY |  | 34 | 0.40 | 1.19 | 0.219 | 0.419 | 1.000 | 3889 | tags=38%, list=18%, signal=46% |
| 1129 | GO\_INNER\_EAR\_MORPHOGENESIS |  | 88 | 0.40 | 1.19 | 0.180 | 0.419 | 1.000 | 3230 | tags=25%, list=15%, signal=29% |
| 1130 | GO\_CELLULAR\_RESPONSE\_TO\_STEROID\_HORMONE\_STIMULUS |  | 208 | 0.33 | 1.19 | 0.124 | 0.420 | 1.000 | 2113 | tags=14%, list=10%, signal=16% |
| 1131 | GO\_REGULATION\_OF\_EPITHELIAL\_CELL\_MIGRATION |  | 159 | 0.38 | 1.19 | 0.217 | 0.420 | 1.000 | 3184 | tags=28%, list=15%, signal=32% |
| 1132 | GO\_NEGATIVE\_REGULATION\_OF\_ANTIGEN\_RECEPTOR\_MEDIATED\_SIGNALING\_PATHWAY |  | 20 | 0.53 | 1.19 | 0.263 | 0.420 | 1.000 | 3732 | tags=50%, list=17%, signal=60% |
| 1133 | GO\_HEAD\_MORPHOGENESIS |  | 34 | 0.45 | 1.18 | 0.212 | 0.419 | 1.000 | 3292 | tags=32%, list=15%, signal=38% |
| 1134 | GO\_MAINTENANCE\_OF\_CELL\_NUMBER |  | 129 | 0.33 | 1.18 | 0.170 | 0.420 | 1.000 | 2800 | tags=15%, list=13%, signal=17% |
| 1135 | GO\_REGULATION\_OF\_MAST\_CELL\_ACTIVATION |  | 38 | 0.49 | 1.18 | 0.273 | 0.420 | 1.000 | 3619 | tags=42%, list=17%, signal=50% |
| 1136 | GO\_REGULATION\_OF\_LYMPHOCYTE\_APOPTOTIC\_PROCESS |  | 52 | 0.49 | 1.18 | 0.239 | 0.420 | 1.000 | 2294 | tags=33%, list=11%, signal=36% |
| 1137 | GO\_RESPONSE\_TO\_METAL\_ION |  | 317 | 0.32 | 1.18 | 0.119 | 0.420 | 1.000 | 3701 | tags=22%, list=17%, signal=27% |
| 1138 | GO\_REGULATION\_OF\_RESPONSE\_TO\_REACTIVE\_OXYGEN\_SPECIES |  | 32 | 0.42 | 1.18 | 0.187 | 0.421 | 1.000 | 2386 | tags=22%, list=11%, signal=25% |
| 1139 | GO\_POSITIVE\_REGULATION\_OF\_IMMUNE\_EFFECTOR\_PROCESS |  | 148 | 0.47 | 1.18 | 0.243 | 0.421 | 1.000 | 3241 | tags=33%, list=15%, signal=39% |
| 1140 | GO\_REGULATION\_OF\_MAP\_KINASE\_ACTIVITY |  | 306 | 0.34 | 1.18 | 0.123 | 0.421 | 1.000 | 3561 | tags=27%, list=16%, signal=32% |
| 1141 | GO\_POSITIVE\_REGULATION\_OF\_LYMPHOCYTE\_DIFFERENTIATION |  | 77 | 0.49 | 1.18 | 0.247 | 0.421 | 1.000 | 3526 | tags=44%, list=16%, signal=53% |
| 1142 | GO\_POSITIVE\_REGULATION\_OF\_CANONICAL\_WNT\_SIGNALING\_PATHWAY |  | 112 | 0.33 | 1.18 | 0.163 | 0.421 | 1.000 | 2125 | tags=14%, list=10%, signal=16% |
| 1143 | GO\_ENDODERM\_FORMATION |  | 48 | 0.49 | 1.18 | 0.254 | 0.422 | 1.000 | 3310 | tags=29%, list=15%, signal=34% |
| 1144 | GO\_ACTIVATION\_OF\_GTPASE\_ACTIVITY |  | 70 | 0.38 | 1.18 | 0.208 | 0.422 | 1.000 | 4187 | tags=40%, list=19%, signal=49% |
| 1145 | GO\_REGULATION\_OF\_PROTEIN\_COMPLEX\_ASSEMBLY |  | 348 | 0.31 | 1.18 | 0.101 | 0.422 | 1.000 | 3170 | tags=20%, list=15%, signal=24% |
| 1146 | GO\_POSITIVE\_REGULATION\_OF\_CYTOKINE\_PRODUCTION |  | 349 | 0.42 | 1.18 | 0.229 | 0.422 | 1.000 | 3166 | tags=28%, list=15%, signal=32% |
| 1147 | GO\_NEGATIVE\_REGULATION\_OF\_CELL\_KILLING |  | 17 | 0.60 | 1.18 | 0.296 | 0.423 | 1.000 | 4288 | tags=47%, list=20%, signal=59% |
| 1148 | GO\_RHO\_PROTEIN\_SIGNAL\_TRANSDUCTION |  | 45 | 0.43 | 1.18 | 0.218 | 0.423 | 1.000 | 3239 | tags=27%, list=15%, signal=31% |
| 1149 | GO\_GLYCEROPHOSPHOLIPID\_METABOLIC\_PROCESS |  | 282 | 0.33 | 1.18 | 0.114 | 0.423 | 1.000 | 4019 | tags=24%, list=18%, signal=30% |
| 1150 | GO\_POSITIVE\_REGULATION\_OF\_MAPK\_CASCADE |  | 438 | 0.36 | 1.18 | 0.152 | 0.423 | 1.000 | 3561 | tags=29%, list=16%, signal=34% |
| 1151 | GO\_GOLGI\_TO\_VACUOLE\_TRANSPORT |  | 25 | 0.40 | 1.18 | 0.249 | 0.423 | 1.000 | 4443 | tags=44%, list=20%, signal=55% |
| 1152 | GO\_MULTI\_MULTICELLULAR\_ORGANISM\_PROCESS |  | 207 | 0.36 | 1.18 | 0.143 | 0.423 | 1.000 | 2144 | tags=18%, list=10%, signal=20% |
| 1153 | GO\_RESPONSE\_TO\_OXIDATIVE\_STRESS |  | 337 | 0.33 | 1.18 | 0.122 | 0.424 | 1.000 | 3305 | tags=24%, list=15%, signal=28% |
| 1154 | GO\_NEUTRAL\_LIPID\_CATABOLIC\_PROCESS |  | 24 | 0.50 | 1.18 | 0.210 | 0.424 | 1.000 | 2784 | tags=25%, list=13%, signal=29% |
| 1155 | GO\_RESPIRATORY\_BURST |  | 15 | 0.63 | 1.18 | 0.303 | 0.423 | 1.000 | 1828 | tags=33%, list=8%, signal=36% |
| 1156 | GO\_REGULATION\_OF\_AXONOGENESIS |  | 160 | 0.37 | 1.18 | 0.170 | 0.423 | 1.000 | 3353 | tags=26%, list=15%, signal=30% |
| 1157 | GO\_NEGATIVE\_REGULATION\_OF\_FAT\_CELL\_DIFFERENTIATION |  | 38 | 0.46 | 1.18 | 0.194 | 0.424 | 1.000 | 3259 | tags=37%, list=15%, signal=43% |
| 1158 | GO\_REGENERATION |  | 152 | 0.38 | 1.18 | 0.152 | 0.424 | 1.000 | 3101 | tags=26%, list=14%, signal=30% |
| 1159 | GO\_POSITIVE\_REGULATION\_OF\_CELL\_KILLING |  | 37 | 0.55 | 1.18 | 0.294 | 0.424 | 1.000 | 1594 | tags=27%, list=7%, signal=29% |
| 1160 | GO\_CELLULAR\_RESPONSE\_TO\_ESTRADIOL\_STIMULUS |  | 29 | 0.45 | 1.18 | 0.226 | 0.424 | 1.000 | 1926 | tags=21%, list=9%, signal=23% |
| 1161 | GO\_BROWN\_FAT\_CELL\_DIFFERENTIATION |  | 29 | 0.48 | 1.18 | 0.204 | 0.424 | 1.000 | 3182 | tags=31%, list=15%, signal=36% |
| 1162 | GO\_PROTEIN\_HOMOOLIGOMERIZATION |  | 240 | 0.31 | 1.18 | 0.100 | 0.424 | 1.000 | 2477 | tags=15%, list=11%, signal=17% |
| 1163 | GO\_NEGATIVE\_REGULATION\_OF\_INNATE\_IMMUNE\_RESPONSE |  | 35 | 0.51 | 1.18 | 0.283 | 0.424 | 1.000 | 4288 | tags=37%, list=20%, signal=46% |
| 1164 | GO\_PIGMENTATION |  | 82 | 0.34 | 1.18 | 0.145 | 0.424 | 1.000 | 3655 | tags=21%, list=17%, signal=25% |
| 1165 | GO\_REGULATION\_OF\_ERYTHROCYTE\_DIFFERENTIATION |  | 36 | 0.41 | 1.18 | 0.223 | 0.424 | 1.000 | 3582 | tags=31%, list=16%, signal=37% |
| 1166 | GO\_LYMPHOCYTE\_HOMEOSTASIS |  | 50 | 0.43 | 1.18 | 0.226 | 0.424 | 1.000 | 3478 | tags=34%, list=16%, signal=40% |
| 1167 | GO\_CELLULAR\_GLUCOSE\_HOMEOSTASIS |  | 73 | 0.37 | 1.18 | 0.195 | 0.424 | 1.000 | 4065 | tags=34%, list=19%, signal=42% |
| 1168 | GO\_PHOSPHOLIPID\_METABOLIC\_PROCESS |  | 336 | 0.32 | 1.18 | 0.116 | 0.424 | 1.000 | 4428 | tags=27%, list=20%, signal=34% |
| 1169 | GO\_REGULATION\_OF\_REACTIVE\_OXYGEN\_SPECIES\_METABOLIC\_PROCESS |  | 146 | 0.37 | 1.18 | 0.160 | 0.425 | 1.000 | 3170 | tags=29%, list=15%, signal=34% |
| 1170 | GO\_EMBRYONIC\_CRANIAL\_SKELETON\_MORPHOGENESIS |  | 46 | 0.46 | 1.18 | 0.233 | 0.425 | 1.000 | 2956 | tags=28%, list=14%, signal=33% |
| 1171 | GO\_NEGATIVE\_REGULATION\_OF\_CARDIAC\_MUSCLE\_TISSUE\_DEVELOPMENT |  | 17 | 0.53 | 1.17 | 0.248 | 0.426 | 1.000 | 1742 | tags=18%, list=8%, signal=19% |
| 1172 | GO\_POSITIVE\_REGULATION\_OF\_DNA\_BINDING |  | 40 | 0.38 | 1.17 | 0.172 | 0.426 | 1.000 | 3406 | tags=23%, list=16%, signal=27% |
| 1173 | GO\_KIDNEY\_MESENCHYME\_DEVELOPMENT |  | 17 | 0.53 | 1.17 | 0.197 | 0.425 | 1.000 | 4551 | tags=41%, list=21%, signal=52% |
| 1174 | GO\_POSITIVE\_REGULATION\_OF\_EPITHELIAL\_CELL\_DIFFERENTIATION |  | 55 | 0.39 | 1.17 | 0.175 | 0.425 | 1.000 | 2089 | tags=18%, list=10%, signal=20% |
| 1175 | GO\_EYE\_DEVELOPMENT |  | 316 | 0.35 | 1.17 | 0.180 | 0.425 | 1.000 | 2401 | tags=16%, list=11%, signal=18% |
| 1176 | GO\_REGULATION\_OF\_NEUROLOGICAL\_SYSTEM\_PROCESS |  | 62 | 0.40 | 1.17 | 0.157 | 0.425 | 1.000 | 3663 | tags=29%, list=17%, signal=35% |
| 1177 | GO\_PROTEIN\_LOCALIZATION\_TO\_NUCLEUS |  | 148 | 0.30 | 1.17 | 0.191 | 0.425 | 1.000 | 3775 | tags=20%, list=17%, signal=24% |
| 1178 | GO\_PHOSPHATIDYLCHOLINE\_ACYL\_CHAIN\_REMODELING |  | 24 | 0.53 | 1.17 | 0.249 | 0.424 | 1.000 | 4408 | tags=33%, list=20%, signal=42% |
| 1179 | GO\_MESONEPHRIC\_TUBULE\_MORPHOGENESIS |  | 53 | 0.44 | 1.17 | 0.211 | 0.426 | 1.000 | 3138 | tags=28%, list=14%, signal=33% |
| 1180 | GO\_CHLORIDE\_TRANSPORT |  | 95 | 0.37 | 1.17 | 0.192 | 0.426 | 1.000 | 3477 | tags=21%, list=16%, signal=25% |
| 1181 | GO\_NEGATIVE\_REGULATION\_OF\_RELEASE\_OF\_CYTOCHROME\_C\_FROM\_MITOCHONDRIA |  | 17 | 0.42 | 1.17 | 0.228 | 0.426 | 1.000 | 2013 | tags=18%, list=9%, signal=19% |
| 1182 | GO\_CELL\_PROLIFERATION\_IN\_FOREBRAIN |  | 26 | 0.45 | 1.17 | 0.262 | 0.426 | 1.000 | 2926 | tags=27%, list=13%, signal=31% |
| 1183 | GO\_RESPONSE\_TO\_CAMP |  | 101 | 0.36 | 1.17 | 0.169 | 0.426 | 1.000 | 1697 | tags=15%, list=8%, signal=16% |
| 1184 | GO\_AMMONIUM\_TRANSMEMBRANE\_TRANSPORT |  | 22 | 0.49 | 1.17 | 0.238 | 0.426 | 1.000 | 578 | tags=14%, list=3%, signal=14% |
| 1185 | GO\_POSITIVE\_REGULATION\_OF\_PROTEIN\_BINDING |  | 69 | 0.36 | 1.17 | 0.189 | 0.425 | 1.000 | 3221 | tags=26%, list=15%, signal=31% |
| 1186 | GO\_CARDIAC\_SEPTUM\_MORPHOGENESIS |  | 49 | 0.49 | 1.17 | 0.255 | 0.425 | 1.000 | 3967 | tags=37%, list=18%, signal=45% |
| 1187 | GO\_GRANULOCYTE\_DIFFERENTIATION |  | 15 | 0.48 | 1.17 | 0.261 | 0.425 | 1.000 | 4743 | tags=47%, list=22%, signal=60% |
| 1188 | GO\_MOTOR\_NEURON\_AXON\_GUIDANCE |  | 27 | 0.45 | 1.17 | 0.227 | 0.425 | 1.000 | 3353 | tags=30%, list=15%, signal=35% |
| 1189 | GO\_REGULATION\_OF\_T\_HELPER\_1\_TYPE\_IMMUNE\_RESPONSE |  | 22 | 0.56 | 1.17 | 0.271 | 0.424 | 1.000 | 2183 | tags=32%, list=10%, signal=35% |
| 1190 | GO\_SUPEROXIDE\_METABOLIC\_PROCESS |  | 32 | 0.45 | 1.17 | 0.236 | 0.426 | 1.000 | 1924 | tags=19%, list=9%, signal=21% |
| 1191 | GO\_MYELOID\_LEUKOCYTE\_ACTIVATION |  | 94 | 0.50 | 1.17 | 0.270 | 0.426 | 1.000 | 3032 | tags=39%, list=14%, signal=46% |
| 1192 | GO\_REGULATION\_OF\_INTERFERON\_GAMMA\_PRODUCTION |  | 88 | 0.52 | 1.17 | 0.270 | 0.425 | 1.000 | 2343 | tags=32%, list=11%, signal=36% |
| 1193 | GO\_POSITIVE\_REGULATION\_OF\_MUSCLE\_TISSUE\_DEVELOPMENT |  | 54 | 0.46 | 1.17 | 0.221 | 0.426 | 1.000 | 3290 | tags=33%, list=15%, signal=39% |
| 1194 | GO\_GAMMA\_AMINOBUTYRIC\_ACID\_SIGNALING\_PATHWAY |  | 21 | 0.43 | 1.17 | 0.242 | 0.426 | 1.000 | 2821 | tags=24%, list=13%, signal=27% |
| 1195 | GO\_VASCULAR\_ENDOTHELIAL\_GROWTH\_FACTOR\_RECEPTOR\_SIGNALING\_PATHWAY |  | 73 | 0.40 | 1.17 | 0.205 | 0.426 | 1.000 | 3170 | tags=27%, list=15%, signal=32% |
| 1196 | GO\_MONOVALENT\_INORGANIC\_CATION\_TRANSPORT |  | 398 | 0.31 | 1.17 | 0.134 | 0.426 | 1.000 | 3998 | tags=20%, list=18%, signal=24% |
| 1197 | GO\_SULFUR\_COMPOUND\_METABOLIC\_PROCESS |  | 334 | 0.35 | 1.17 | 0.150 | 0.425 | 1.000 | 3566 | tags=23%, list=16%, signal=27% |
| 1198 | GO\_CYTOSOLIC\_TRANSPORT |  | 193 | 0.31 | 1.17 | 0.162 | 0.427 | 1.000 | 3768 | tags=24%, list=17%, signal=29% |
| 1199 | GO\_RESPONSE\_TO\_PH |  | 35 | 0.41 | 1.17 | 0.213 | 0.427 | 1.000 | 3285 | tags=26%, list=15%, signal=30% |
| 1200 | GO\_POSITIVE\_REGULATION\_OF\_EPITHELIAL\_TO\_MESENCHYMAL\_TRANSITION |  | 33 | 0.48 | 1.17 | 0.236 | 0.426 | 1.000 | 3459 | tags=42%, list=16%, signal=50% |
| 1201 | GO\_RESPONSE\_TO\_NICOTINE |  | 49 | 0.43 | 1.17 | 0.223 | 0.427 | 1.000 | 2089 | tags=16%, list=10%, signal=18% |
| 1202 | GO\_REGULATION\_OF\_LYMPHOCYTE\_CHEMOTAXIS |  | 18 | 0.63 | 1.17 | 0.310 | 0.427 | 1.000 | 3170 | tags=44%, list=15%, signal=52% |
| 1203 | GO\_BONE\_GROWTH |  | 19 | 0.54 | 1.17 | 0.252 | 0.426 | 1.000 | 1742 | tags=26%, list=8%, signal=29% |
| 1204 | GO\_NEGATIVE\_REGULATION\_OF\_SMOOTH\_MUSCLE\_CONTRACTION |  | 15 | 0.51 | 1.17 | 0.225 | 0.427 | 1.000 | 818 | tags=20%, list=4%, signal=21% |
| 1205 | GO\_REGULATION\_OF\_CARTILAGE\_DEVELOPMENT |  | 59 | 0.47 | 1.17 | 0.224 | 0.427 | 1.000 | 3482 | tags=32%, list=16%, signal=38% |
| 1206 | GO\_FATTY\_ACID\_BIOSYNTHETIC\_PROCESS |  | 98 | 0.41 | 1.17 | 0.203 | 0.426 | 1.000 | 2478 | tags=21%, list=11%, signal=24% |
| 1207 | GO\_PROTEIN\_STABILIZATION |  | 122 | 0.30 | 1.17 | 0.156 | 0.427 | 1.000 | 2500 | tags=15%, list=11%, signal=17% |
| 1208 | GO\_REGULATION\_OF\_SEQUENCE\_SPECIFIC\_DNA\_BINDING\_TRANSCRIPTION\_FACTOR\_ACTIVITY |  | 344 | 0.32 | 1.17 | 0.144 | 0.427 | 1.000 | 3290 | tags=19%, list=15%, signal=22% |
| 1209 | GO\_POSITIVE\_REGULATION\_OF\_INTERLEUKIN\_12\_PRODUCTION |  | 33 | 0.54 | 1.17 | 0.277 | 0.427 | 1.000 | 3347 | tags=36%, list=15%, signal=43% |
| 1210 | GO\_DETECTION\_OF\_BIOTIC\_STIMULUS |  | 24 | 0.55 | 1.17 | 0.299 | 0.428 | 1.000 | 3945 | tags=46%, list=18%, signal=56% |
| 1211 | GO\_SYNAPTIC\_VESICLE\_LOCALIZATION |  | 95 | 0.35 | 1.17 | 0.189 | 0.429 | 1.000 | 3085 | tags=20%, list=14%, signal=23% |
| 1212 | GO\_LEUKOCYTE\_MEDIATED\_CYTOTOXICITY |  | 31 | 0.49 | 1.16 | 0.256 | 0.430 | 1.000 | 2917 | tags=32%, list=13%, signal=37% |
| 1213 | GO\_REGULATION\_OF\_MAST\_CELL\_ACTIVATION\_INVOLVED\_IN\_IMMUNE\_RESPONSE |  | 31 | 0.50 | 1.16 | 0.283 | 0.430 | 1.000 | 3619 | tags=45%, list=17%, signal=54% |
| 1214 | GO\_DETECTION\_OF\_STIMULUS |  | 312 | 0.29 | 1.16 | 0.208 | 0.431 | 1.000 | 3021 | tags=12%, list=14%, signal=13% |
| 1215 | GO\_RESPONSE\_TO\_STEROID\_HORMONE |  | 478 | 0.33 | 1.16 | 0.118 | 0.431 | 1.000 | 2144 | tags=15%, list=10%, signal=17% |
| 1216 | GO\_DEPHOSPHORYLATION |  | 257 | 0.30 | 1.16 | 0.134 | 0.431 | 1.000 | 3742 | tags=23%, list=17%, signal=28% |
| 1217 | GO\_NEGATIVE\_REGULATION\_OF\_PROTEIN\_LOCALIZATION\_TO\_CELL\_PERIPHERY |  | 19 | 0.48 | 1.16 | 0.244 | 0.431 | 1.000 | 2503 | tags=26%, list=12%, signal=30% |
| 1218 | GO\_RESPONSE\_TO\_ACTIVITY |  | 66 | 0.42 | 1.16 | 0.231 | 0.431 | 1.000 | 3080 | tags=27%, list=14%, signal=32% |
| 1219 | GO\_NEGATIVE\_REGULATION\_OF\_LYMPHOCYTE\_MEDIATED\_IMMUNITY |  | 35 | 0.51 | 1.16 | 0.305 | 0.432 | 1.000 | 4288 | tags=37%, list=20%, signal=46% |
| 1220 | GO\_BONE\_REMODELING |  | 35 | 0.43 | 1.16 | 0.253 | 0.431 | 1.000 | 3379 | tags=31%, list=16%, signal=37% |
| 1221 | GO\_REGULATION\_OF\_INTERLEUKIN\_10\_PRODUCTION |  | 43 | 0.51 | 1.16 | 0.278 | 0.431 | 1.000 | 2764 | tags=30%, list=13%, signal=35% |
| 1222 | GO\_CELLULAR\_MONOVALENT\_INORGANIC\_CATION\_HOMEOSTASIS |  | 90 | 0.34 | 1.16 | 0.233 | 0.431 | 1.000 | 4406 | tags=27%, list=20%, signal=33% |
| 1223 | GO\_REGULATION\_OF\_SUBSTRATE\_ADHESION\_DEPENDENT\_CELL\_SPREADING |  | 39 | 0.39 | 1.16 | 0.223 | 0.431 | 1.000 | 2074 | tags=15%, list=10%, signal=17% |
| 1224 | GO\_POSITIVE\_REGULATION\_OF\_G\_PROTEIN\_COUPLED\_RECEPTOR\_PROTEIN\_SIGNALING\_PATHWAY |  | 23 | 0.49 | 1.16 | 0.249 | 0.431 | 1.000 | 3658 | tags=35%, list=17%, signal=42% |
| 1225 | GO\_SYNAPTIC\_SIGNALING |  | 403 | 0.32 | 1.16 | 0.181 | 0.433 | 1.000 | 3191 | tags=16%, list=15%, signal=18% |
| 1226 | GO\_NEGATIVE\_REGULATION\_OF\_LYMPHOCYTE\_APOPTOTIC\_PROCESS |  | 27 | 0.51 | 1.16 | 0.265 | 0.433 | 1.000 | 3176 | tags=52%, list=15%, signal=61% |
| 1227 | GO\_RESPONSE\_TO\_ALCOHOL |  | 354 | 0.33 | 1.16 | 0.116 | 0.433 | 1.000 | 2125 | tags=18%, list=10%, signal=19% |
| 1228 | GO\_AMINE\_METABOLIC\_PROCESS |  | 124 | 0.37 | 1.16 | 0.203 | 0.432 | 1.000 | 3893 | tags=22%, list=18%, signal=26% |
| 1229 | GO\_FATTY\_ACID\_METABOLIC\_PROCESS |  | 270 | 0.39 | 1.16 | 0.224 | 0.432 | 1.000 | 2642 | tags=19%, list=12%, signal=21% |
| 1230 | GO\_PROTEIN\_LIPID\_COMPLEX\_ASSEMBLY |  | 21 | 0.45 | 1.16 | 0.297 | 0.432 | 1.000 | 3441 | tags=29%, list=16%, signal=34% |
| 1231 | GO\_PHOSPHATIDYLETHANOLAMINE\_ACYL\_CHAIN\_REMODELING |  | 21 | 0.56 | 1.16 | 0.273 | 0.432 | 1.000 | 4408 | tags=38%, list=20%, signal=48% |
| 1232 | GO\_REGULATION\_OF\_NEUTROPHIL\_CHEMOTAXIS |  | 25 | 0.58 | 1.16 | 0.266 | 0.432 | 1.000 | 3331 | tags=56%, list=15%, signal=66% |
| 1233 | GO\_FEMALE\_SEX\_DIFFERENTIATION |  | 112 | 0.36 | 1.16 | 0.170 | 0.434 | 1.000 | 2956 | tags=21%, list=14%, signal=25% |
| 1234 | GO\_PLATELET\_DERIVED\_GROWTH\_FACTOR\_RECEPTOR\_SIGNALING\_PATHWAY |  | 34 | 0.44 | 1.16 | 0.269 | 0.434 | 1.000 | 3826 | tags=35%, list=18%, signal=43% |
| 1235 | GO\_POSITIVE\_REGULATION\_OF\_ESTABLISHMENT\_OF\_PROTEIN\_LOCALIZATION |  | 472 | 0.32 | 1.16 | 0.126 | 0.434 | 1.000 | 3153 | tags=21%, list=14%, signal=24% |
| 1236 | GO\_GAS\_TRANSPORT |  | 17 | 0.55 | 1.16 | 0.281 | 0.434 | 1.000 | 401 | tags=18%, list=2%, signal=18% |
| 1237 | GO\_RESPONSE\_TO\_ETHANOL |  | 134 | 0.35 | 1.16 | 0.186 | 0.435 | 1.000 | 4217 | tags=28%, list=19%, signal=35% |
| 1238 | GO\_HOMEOSTASIS\_OF\_NUMBER\_OF\_CELLS\_WITHIN\_A\_TISSUE |  | 30 | 0.42 | 1.16 | 0.261 | 0.436 | 1.000 | 3196 | tags=27%, list=15%, signal=31% |
| 1239 | GO\_EPITHELIAL\_TO\_MESENCHYMAL\_TRANSITION |  | 56 | 0.44 | 1.16 | 0.240 | 0.435 | 1.000 | 3408 | tags=32%, list=16%, signal=38% |
| 1240 | GO\_CELLULAR\_RESPONSE\_TO\_ALCOHOL |  | 111 | 0.37 | 1.16 | 0.168 | 0.435 | 1.000 | 2112 | tags=20%, list=10%, signal=22% |
| 1241 | GO\_NEGATIVE\_REGULATION\_OF\_OXIDOREDUCTASE\_ACTIVITY |  | 26 | 0.42 | 1.16 | 0.261 | 0.436 | 1.000 | 1319 | tags=15%, list=6%, signal=16% |
| 1242 | GO\_AXON\_REGENERATION |  | 22 | 0.49 | 1.16 | 0.238 | 0.436 | 1.000 | 2442 | tags=27%, list=11%, signal=31% |
| 1243 | GO\_PROTEIN\_MATURATION |  | 248 | 0.33 | 1.16 | 0.132 | 0.436 | 1.000 | 2647 | tags=19%, list=12%, signal=21% |
| 1244 | GO\_HOMOPHILIC\_CELL\_ADHESION\_VIA\_PLASMA\_MEMBRANE\_ADHESION\_MOLECULES |  | 125 | 0.39 | 1.16 | 0.185 | 0.435 | 1.000 | 1485 | tags=14%, list=7%, signal=15% |
| 1245 | GO\_POSITIVE\_REGULATION\_OF\_DEFENSE\_RESPONSE |  | 344 | 0.41 | 1.16 | 0.280 | 0.435 | 1.000 | 3137 | tags=25%, list=14%, signal=29% |
| 1246 | GO\_IMPORT\_INTO\_CELL |  | 37 | 0.41 | 1.16 | 0.227 | 0.435 | 1.000 | 3139 | tags=24%, list=14%, signal=28% |
| 1247 | GO\_REGULATION\_OF\_PROTEIN\_BINDING |  | 163 | 0.32 | 1.16 | 0.153 | 0.435 | 1.000 | 3221 | tags=23%, list=15%, signal=27% |
| 1248 | GO\_REGULATION\_OF\_TRIGLYCERIDE\_METABOLIC\_PROCESS |  | 33 | 0.42 | 1.16 | 0.275 | 0.435 | 1.000 | 4073 | tags=30%, list=19%, signal=37% |
| 1249 | GO\_CELL\_CHEMOTAXIS |  | 151 | 0.49 | 1.16 | 0.259 | 0.435 | 1.000 | 3095 | tags=38%, list=14%, signal=44% |
| 1250 | GO\_POSITIVE\_REGULATION\_OF\_RECEPTOR\_INTERNALIZATION |  | 22 | 0.49 | 1.16 | 0.265 | 0.435 | 1.000 | 3561 | tags=55%, list=16%, signal=65% |
| 1251 | GO\_POSITIVE\_REGULATION\_OF\_NUCLEOCYTOPLASMIC\_TRANSPORT |  | 116 | 0.38 | 1.16 | 0.198 | 0.435 | 1.000 | 3153 | tags=25%, list=14%, signal=29% |
| 1252 | GO\_CELLULAR\_RESPONSE\_TO\_OSMOTIC\_STRESS |  | 20 | 0.46 | 1.16 | 0.239 | 0.435 | 1.000 | 1966 | tags=20%, list=9%, signal=22% |
| 1253 | GO\_REGULATION\_OF\_NEUTROPHIL\_MIGRATION |  | 30 | 0.55 | 1.15 | 0.278 | 0.435 | 1.000 | 3331 | tags=53%, list=15%, signal=63% |
| 1254 | GO\_HETEROTYPIC\_CELL\_CELL\_ADHESION |  | 26 | 0.50 | 1.15 | 0.260 | 0.435 | 1.000 | 3125 | tags=38%, list=14%, signal=45% |
| 1255 | GO\_REGULATION\_OF\_SYNAPTIC\_PLASTICITY |  | 136 | 0.33 | 1.15 | 0.191 | 0.435 | 1.000 | 3288 | tags=18%, list=15%, signal=22% |
| 1256 | GO\_PROTEIN\_DEPHOSPHORYLATION |  | 177 | 0.30 | 1.15 | 0.175 | 0.436 | 1.000 | 2887 | tags=19%, list=13%, signal=21% |
| 1257 | GO\_STEM\_CELL\_DIVISION |  | 28 | 0.47 | 1.15 | 0.270 | 0.436 | 1.000 | 2863 | tags=29%, list=13%, signal=33% |
| 1258 | GO\_POSITIVE\_REGULATION\_OF\_ACTIN\_FILAMENT\_BUNDLE\_ASSEMBLY |  | 45 | 0.41 | 1.15 | 0.186 | 0.436 | 1.000 | 2085 | tags=22%, list=10%, signal=25% |
| 1259 | GO\_CRANIAL\_SKELETAL\_SYSTEM\_DEVELOPMENT |  | 55 | 0.44 | 1.15 | 0.268 | 0.435 | 1.000 | 2956 | tags=27%, list=14%, signal=31% |
| 1260 | GO\_INFLAMMATORY\_RESPONSE |  | 422 | 0.43 | 1.15 | 0.248 | 0.436 | 1.000 | 3176 | tags=29%, list=15%, signal=34% |
| 1261 | GO\_CELLULAR\_IRON\_ION\_HOMEOSTASIS |  | 43 | 0.42 | 1.15 | 0.251 | 0.436 | 1.000 | 3775 | tags=26%, list=17%, signal=31% |
| 1262 | GO\_POSITIVE\_REGULATION\_OF\_NF\_KAPPAB\_IMPORT\_INTO\_NUCLEUS |  | 27 | 0.53 | 1.15 | 0.269 | 0.437 | 1.000 | 2042 | tags=30%, list=9%, signal=33% |
| 1263 | GO\_ETHANOLAMINE\_CONTAINING\_COMPOUND\_METABOLIC\_PROCESS |  | 81 | 0.40 | 1.15 | 0.248 | 0.437 | 1.000 | 4408 | tags=27%, list=20%, signal=34% |
| 1264 | GO\_SENSORY\_ORGAN\_MORPHOGENESIS |  | 230 | 0.37 | 1.15 | 0.217 | 0.438 | 1.000 | 3090 | tags=22%, list=14%, signal=26% |
| 1265 | GO\_REGULATION\_OF\_SUPEROXIDE\_METABOLIC\_PROCESS |  | 22 | 0.46 | 1.15 | 0.211 | 0.437 | 1.000 | 3800 | tags=41%, list=17%, signal=50% |
| 1266 | GO\_POSITIVE\_REGULATION\_OF\_TOLL\_LIKE\_RECEPTOR\_SIGNALING\_PATHWAY |  | 18 | 0.56 | 1.15 | 0.281 | 0.437 | 1.000 | 3800 | tags=44%, list=17%, signal=54% |
| 1267 | GO\_REGULATION\_OF\_INTRINSIC\_APOPTOTIC\_SIGNALING\_PATHWAY\_IN\_RESPONSE\_TO\_DNA\_DAMAGE |  | 30 | 0.41 | 1.15 | 0.232 | 0.437 | 1.000 | 603 | tags=10%, list=3%, signal=10% |
| 1268 | GO\_POSITIVE\_REGULATION\_OF\_ERK1\_AND\_ERK2\_CASCADE |  | 157 | 0.42 | 1.15 | 0.235 | 0.439 | 1.000 | 3170 | tags=32%, list=15%, signal=38% |
| 1269 | GO\_REGULATION\_OF\_LEUKOCYTE\_MEDIATED\_IMMUNITY |  | 152 | 0.46 | 1.15 | 0.271 | 0.439 | 1.000 | 3526 | tags=36%, list=16%, signal=43% |
| 1270 | GO\_REGULATION\_OF\_EXOCYTOSIS |  | 176 | 0.33 | 1.15 | 0.151 | 0.439 | 1.000 | 3630 | tags=21%, list=17%, signal=25% |
| 1271 | GO\_REGULATION\_OF\_T\_CELL\_APOPTOTIC\_PROCESS |  | 31 | 0.49 | 1.15 | 0.269 | 0.440 | 1.000 | 2183 | tags=29%, list=10%, signal=32% |
| 1272 | GO\_FATTY\_ACID\_BETA\_OXIDATION\_USING\_ACYL\_COA\_DEHYDROGENASE |  | 17 | 0.53 | 1.15 | 0.359 | 0.440 | 1.000 | 5233 | tags=59%, list=24%, signal=77% |
| 1273 | GO\_STEM\_CELL\_PROLIFERATION |  | 58 | 0.43 | 1.15 | 0.230 | 0.441 | 1.000 | 2956 | tags=28%, list=14%, signal=32% |
| 1274 | GO\_REGULATION\_OF\_ESTABLISHMENT\_OF\_PROTEIN\_LOCALIZATION\_TO\_PLASMA\_MEMBRANE |  | 45 | 0.39 | 1.15 | 0.186 | 0.441 | 1.000 | 1158 | tags=18%, list=5%, signal=19% |
| 1275 | GO\_NEGATIVE\_REGULATION\_OF\_BINDING |  | 129 | 0.34 | 1.15 | 0.176 | 0.441 | 1.000 | 2863 | tags=19%, list=13%, signal=21% |
| 1276 | GO\_POSITIVE\_REGULATION\_OF\_MONOCYTE\_CHEMOTAXIS |  | 15 | 0.63 | 1.15 | 0.321 | 0.441 | 1.000 | 3080 | tags=47%, list=14%, signal=54% |
| 1277 | GO\_POSITIVE\_REGULATION\_OF\_INTRACELLULAR\_PROTEIN\_TRANSPORT |  | 231 | 0.31 | 1.15 | 0.163 | 0.441 | 1.000 | 3153 | tags=20%, list=14%, signal=23% |
| 1278 | GO\_LEARNING |  | 124 | 0.35 | 1.15 | 0.192 | 0.441 | 1.000 | 2112 | tags=13%, list=10%, signal=14% |
| 1279 | GO\_TELENCEPHALON\_DEVELOPMENT |  | 222 | 0.33 | 1.15 | 0.205 | 0.441 | 1.000 | 3655 | tags=24%, list=17%, signal=29% |
| 1280 | GO\_VISUAL\_BEHAVIOR |  | 46 | 0.35 | 1.15 | 0.238 | 0.442 | 1.000 | 232 | tags=4%, list=1%, signal=4% |
| 1281 | GO\_PURINE\_CONTAINING\_COMPOUND\_METABOLIC\_PROCESS |  | 365 | 0.29 | 1.15 | 0.168 | 0.441 | 1.000 | 1970 | tags=11%, list=9%, signal=12% |
| 1282 | GO\_POSITIVE\_REGULATION\_OF\_PEPTIDYL\_TYROSINE\_PHOSPHORYLATION |  | 154 | 0.38 | 1.15 | 0.158 | 0.441 | 1.000 | 3170 | tags=28%, list=15%, signal=32% |
| 1283 | GO\_EPITHELIAL\_CELL\_FATE\_COMMITMENT |  | 15 | 0.49 | 1.15 | 0.260 | 0.442 | 1.000 | 3967 | tags=33%, list=18%, signal=41% |
| 1284 | GO\_PEPTIDE\_CROSS\_LINKING |  | 39 | 0.60 | 1.15 | 0.361 | 0.442 | 1.000 | 2995 | tags=38%, list=14%, signal=45% |
| 1285 | GO\_RESPONSE\_TO\_ORGANOPHOSPHORUS |  | 134 | 0.35 | 1.14 | 0.196 | 0.443 | 1.000 | 2142 | tags=18%, list=10%, signal=20% |
| 1286 | GO\_MAINTENANCE\_OF\_LOCATION\_IN\_CELL |  | 91 | 0.32 | 1.14 | 0.218 | 0.443 | 1.000 | 3825 | tags=22%, list=18%, signal=27% |
| 1287 | GO\_ODONTOGENESIS |  | 101 | 0.41 | 1.14 | 0.229 | 0.443 | 1.000 | 3402 | tags=30%, list=16%, signal=35% |
| 1288 | GO\_REGULATION\_OF\_CYTOSKELETON\_ORGANIZATION |  | 471 | 0.31 | 1.14 | 0.185 | 0.444 | 1.000 | 3170 | tags=22%, list=15%, signal=25% |
| 1289 | GO\_LEUKOCYTE\_HOMEOSTASIS |  | 59 | 0.41 | 1.14 | 0.279 | 0.444 | 1.000 | 3478 | tags=36%, list=16%, signal=42% |
| 1290 | GO\_NEGATIVE\_REGULATION\_OF\_BLOOD\_VESSEL\_ENDOTHELIAL\_CELL\_MIGRATION |  | 23 | 0.44 | 1.14 | 0.279 | 0.444 | 1.000 | 2451 | tags=26%, list=11%, signal=29% |
| 1291 | GO\_LOCALIZATION\_WITHIN\_MEMBRANE |  | 118 | 0.32 | 1.14 | 0.212 | 0.444 | 1.000 | 2469 | tags=17%, list=11%, signal=19% |
| 1292 | GO\_REGULATION\_OF\_ERK1\_AND\_ERK2\_CASCADE |  | 221 | 0.39 | 1.14 | 0.231 | 0.445 | 1.000 | 3547 | tags=30%, list=16%, signal=36% |
| 1293 | GO\_REGULATION\_OF\_CILIUM\_ASSEMBLY |  | 46 | 0.34 | 1.14 | 0.260 | 0.446 | 1.000 | 5654 | tags=41%, list=26%, signal=56% |
| 1294 | GO\_LIPID\_PHOSPHORYLATION |  | 95 | 0.36 | 1.14 | 0.192 | 0.446 | 1.000 | 3066 | tags=24%, list=14%, signal=28% |
| 1295 | GO\_POSITIVE\_REGULATION\_OF\_ENDOTHELIAL\_CELL\_MIGRATION |  | 63 | 0.43 | 1.14 | 0.290 | 0.446 | 1.000 | 3549 | tags=37%, list=16%, signal=43% |
| 1296 | GO\_PROTEIN\_LOCALIZATION\_TO\_GOLGI\_APPARATUS |  | 28 | 0.36 | 1.14 | 0.285 | 0.447 | 1.000 | 5665 | tags=46%, list=26%, signal=63% |
| 1297 | GO\_BRANCHED\_CHAIN\_AMINO\_ACID\_METABOLIC\_PROCESS |  | 23 | 0.48 | 1.14 | 0.299 | 0.447 | 1.000 | 3474 | tags=43%, list=16%, signal=52% |
| 1298 | GO\_SYNAPTIC\_TRANSMISSION\_GLUTAMATERGIC |  | 21 | 0.42 | 1.14 | 0.258 | 0.447 | 1.000 | 2142 | tags=19%, list=10%, signal=21% |
| 1299 | GO\_ANTIGEN\_PROCESSING\_AND\_PRESENTATION\_VIA\_MHC\_CLASS\_IB |  | 15 | 0.58 | 1.14 | 0.332 | 0.447 | 1.000 | 2735 | tags=33%, list=13%, signal=38% |
| 1300 | GO\_ORGANIC\_HYDROXY\_COMPOUND\_TRANSPORT |  | 143 | 0.35 | 1.14 | 0.165 | 0.447 | 1.000 | 3105 | tags=21%, list=14%, signal=24% |
| 1301 | GO\_NEGATIVE\_REGULATION\_OF\_SYNAPTIC\_TRANSMISSION |  | 59 | 0.36 | 1.14 | 0.222 | 0.447 | 1.000 | 3506 | tags=20%, list=16%, signal=24% |
| 1302 | GO\_MONOVALENT\_INORGANIC\_ANION\_HOMEOSTASIS |  | 18 | 0.47 | 1.14 | 0.272 | 0.447 | 1.000 | 510 | tags=11%, list=2%, signal=11% |
| 1303 | GO\_ATP\_HYDROLYSIS\_COUPLED\_TRANSMEMBRANE\_TRANSPORT |  | 34 | 0.41 | 1.14 | 0.285 | 0.448 | 1.000 | 5140 | tags=38%, list=24%, signal=50% |
| 1304 | GO\_RESPONSE\_TO\_ANGIOTENSIN |  | 15 | 0.52 | 1.14 | 0.286 | 0.449 | 1.000 | 819 | tags=20%, list=4%, signal=21% |
| 1305 | GO\_REGULATION\_OF\_CELL\_MATRIX\_ADHESION |  | 87 | 0.38 | 1.14 | 0.231 | 0.448 | 1.000 | 3408 | tags=29%, list=16%, signal=34% |
| 1306 | GO\_MUSCLE\_CELL\_CELLULAR\_HOMEOSTASIS |  | 17 | 0.55 | 1.14 | 0.327 | 0.449 | 1.000 | 1358 | tags=29%, list=6%, signal=31% |
| 1307 | GO\_MORPHOGENESIS\_OF\_AN\_ENDOTHELIUM |  | 16 | 0.47 | 1.14 | 0.300 | 0.449 | 1.000 | 2956 | tags=31%, list=14%, signal=36% |
| 1308 | GO\_SYNAPSE\_ORGANIZATION |  | 139 | 0.38 | 1.14 | 0.227 | 0.449 | 1.000 | 3191 | tags=23%, list=15%, signal=27% |
| 1309 | GO\_LEUKOCYTE\_MIGRATION |  | 248 | 0.45 | 1.14 | 0.263 | 0.450 | 1.000 | 3125 | tags=32%, list=14%, signal=37% |
| 1310 | GO\_CELLULAR\_TRANSITION\_METAL\_ION\_HOMEOSTASIS |  | 74 | 0.35 | 1.14 | 0.243 | 0.449 | 1.000 | 3775 | tags=22%, list=17%, signal=26% |
| 1311 | GO\_EMBRYONIC\_ORGAN\_DEVELOPMENT |  | 394 | 0.35 | 1.14 | 0.219 | 0.449 | 1.000 | 3250 | tags=22%, list=15%, signal=25% |
| 1312 | GO\_REGULATION\_OF\_URINE\_VOLUME |  | 20 | 0.48 | 1.14 | 0.280 | 0.449 | 1.000 | 3502 | tags=25%, list=16%, signal=30% |
| 1313 | GO\_RESPONSE\_TO\_MONOAMINE |  | 35 | 0.42 | 1.14 | 0.269 | 0.449 | 1.000 | 2708 | tags=26%, list=12%, signal=29% |
| 1314 | GO\_NEGATIVE\_REGULATION\_OF\_NEURON\_DEATH |  | 162 | 0.36 | 1.14 | 0.212 | 0.449 | 1.000 | 3222 | tags=23%, list=15%, signal=27% |
| 1315 | GO\_REGULATION\_OF\_LYMPHOCYTE\_MEDIATED\_IMMUNITY |  | 110 | 0.45 | 1.14 | 0.293 | 0.448 | 1.000 | 3478 | tags=35%, list=16%, signal=42% |
| 1316 | GO\_LONG\_TERM\_MEMORY |  | 27 | 0.37 | 1.14 | 0.282 | 0.449 | 1.000 | 4516 | tags=22%, list=21%, signal=28% |
| 1317 | GO\_GLUCOSAMINE\_CONTAINING\_COMPOUND\_METABOLIC\_PROCESS |  | 24 | 0.47 | 1.14 | 0.278 | 0.449 | 1.000 | 4937 | tags=54%, list=23%, signal=70% |
| 1318 | GO\_REGULATION\_OF\_PROTEIN\_SERINE\_THREONINE\_KINASE\_ACTIVITY |  | 448 | 0.31 | 1.14 | 0.158 | 0.450 | 1.000 | 3579 | tags=26%, list=16%, signal=30% |
| 1319 | GO\_RESPONSE\_TO\_INORGANIC\_SUBSTANCE |  | 457 | 0.31 | 1.13 | 0.175 | 0.451 | 1.000 | 3170 | tags=19%, list=15%, signal=22% |
| 1320 | GO\_BIOMINERAL\_TISSUE\_DEVELOPMENT |  | 67 | 0.42 | 1.13 | 0.240 | 0.451 | 1.000 | 2125 | tags=19%, list=10%, signal=21% |
| 1321 | GO\_POSITIVE\_REGULATION\_OF\_INFLAMMATORY\_RESPONSE |  | 106 | 0.47 | 1.13 | 0.280 | 0.451 | 1.000 | 3129 | tags=36%, list=14%, signal=42% |
| 1322 | GO\_PANCREAS\_DEVELOPMENT |  | 70 | 0.38 | 1.13 | 0.247 | 0.452 | 1.000 | 5198 | tags=36%, list=24%, signal=47% |
| 1323 | GO\_PURINE\_RIBONUCLEOSIDE\_BISPHOSPHATE\_METABOLIC\_PROCESS |  | 18 | 0.49 | 1.13 | 0.268 | 0.451 | 1.000 | 5418 | tags=50%, list=25%, signal=67% |
| 1324 | GO\_DENDRITE\_DEVELOPMENT |  | 76 | 0.37 | 1.13 | 0.248 | 0.452 | 1.000 | 3681 | tags=25%, list=17%, signal=30% |
| 1325 | GO\_REGULATION\_OF\_EXTENT\_OF\_CELL\_GROWTH |  | 96 | 0.36 | 1.13 | 0.230 | 0.451 | 1.000 | 3353 | tags=27%, list=15%, signal=32% |
| 1326 | GO\_POSITIVE\_REGULATION\_OF\_PROTEASOMAL\_PROTEIN\_CATABOLIC\_PROCESS |  | 96 | 0.31 | 1.13 | 0.220 | 0.451 | 1.000 | 2478 | tags=17%, list=11%, signal=19% |
| 1327 | GO\_NEGATIVE\_REGULATION\_OF\_T\_CELL\_MEDIATED\_IMMUNITY |  | 16 | 0.57 | 1.13 | 0.334 | 0.452 | 1.000 | 2940 | tags=31%, list=14%, signal=36% |
| 1328 | GO\_REGULATION\_OF\_CELL\_KILLING |  | 60 | 0.50 | 1.13 | 0.307 | 0.452 | 1.000 | 3478 | tags=38%, list=16%, signal=46% |
| 1329 | GO\_EXTRACELLULAR\_MATRIX\_DISASSEMBLY |  | 72 | 0.47 | 1.13 | 0.317 | 0.452 | 1.000 | 2000 | tags=29%, list=9%, signal=32% |
| 1330 | GO\_REGULATION\_OF\_AMINO\_ACID\_TRANSPORT |  | 24 | 0.46 | 1.13 | 0.246 | 0.454 | 1.000 | 2376 | tags=29%, list=11%, signal=33% |
| 1331 | GO\_REGULATION\_OF\_LEUKOCYTE\_DEGRANULATION |  | 41 | 0.46 | 1.13 | 0.302 | 0.454 | 1.000 | 3619 | tags=37%, list=17%, signal=44% |
| 1332 | GO\_NEGATIVE\_REGULATION\_OF\_ALPHA\_BETA\_T\_CELL\_DIFFERENTIATION |  | 15 | 0.47 | 1.13 | 0.292 | 0.454 | 1.000 | 3176 | tags=27%, list=15%, signal=31% |
| 1333 | GO\_GENERATION\_OF\_PRECURSOR\_METABOLITES\_AND\_ENERGY |  | 272 | 0.29 | 1.13 | 0.272 | 0.454 | 1.000 | 2882 | tags=13%, list=13%, signal=15% |
| 1334 | GO\_CELLULAR\_RESPONSE\_TO\_RETINOIC\_ACID |  | 58 | 0.40 | 1.13 | 0.225 | 0.455 | 1.000 | 4234 | tags=36%, list=19%, signal=45% |
| 1335 | GO\_REGULATION\_OF\_PEPTIDYL\_TYROSINE\_PHOSPHORYLATION |  | 202 | 0.36 | 1.13 | 0.197 | 0.455 | 1.000 | 3170 | tags=25%, list=15%, signal=29% |
| 1336 | GO\_REGULATION\_OF\_NEUROTRANSMITTER\_LEVELS |  | 176 | 0.35 | 1.13 | 0.219 | 0.455 | 1.000 | 2348 | tags=14%, list=11%, signal=16% |
| 1337 | GO\_NATURAL\_KILLER\_CELL\_MEDIATED\_IMMUNITY |  | 22 | 0.53 | 1.13 | 0.340 | 0.455 | 1.000 | 3457 | tags=41%, list=16%, signal=49% |
| 1338 | GO\_CHONDROCYTE\_DIFFERENTIATION |  | 57 | 0.45 | 1.13 | 0.274 | 0.455 | 1.000 | 2020 | tags=25%, list=9%, signal=27% |
| 1339 | GO\_MYELOID\_CELL\_ACTIVATION\_INVOLVED\_IN\_IMMUNE\_RESPONSE |  | 40 | 0.49 | 1.13 | 0.316 | 0.455 | 1.000 | 2920 | tags=33%, list=13%, signal=37% |
| 1340 | GO\_NEGATIVE\_REGULATION\_OF\_STRIATED\_MUSCLE\_CELL\_APOPTOTIC\_PROCESS |  | 17 | 0.46 | 1.13 | 0.263 | 0.456 | 1.000 | 3619 | tags=41%, list=17%, signal=49% |
| 1341 | GO\_REGULATION\_OF\_RESPONSE\_TO\_OXIDATIVE\_STRESS |  | 61 | 0.34 | 1.13 | 0.210 | 0.457 | 1.000 | 2386 | tags=15%, list=11%, signal=17% |
| 1342 | GO\_POSITIVE\_REGULATION\_OF\_EPITHELIAL\_CELL\_PROLIFERATION |  | 146 | 0.39 | 1.13 | 0.232 | 0.457 | 1.000 | 3370 | tags=30%, list=15%, signal=35% |
| 1343 | GO\_MEGAKARYOCYTE\_DIFFERENTIATION |  | 20 | 0.44 | 1.13 | 0.300 | 0.457 | 1.000 | 4065 | tags=35%, list=19%, signal=43% |
| 1344 | GO\_PHOSPHATIDYLGLYCEROL\_ACYL\_CHAIN\_REMODELING |  | 16 | 0.54 | 1.13 | 0.321 | 0.457 | 1.000 | 4408 | tags=31%, list=20%, signal=39% |
| 1345 | GO\_NEGATIVE\_REGULATION\_OF\_TOLL\_LIKE\_RECEPTOR\_SIGNALING\_PATHWAY |  | 25 | 0.49 | 1.13 | 0.302 | 0.458 | 1.000 | 3619 | tags=40%, list=17%, signal=48% |
| 1346 | GO\_POSITIVE\_REGULATION\_OF\_VASOCONSTRICTION |  | 35 | 0.42 | 1.13 | 0.283 | 0.458 | 1.000 | 2461 | tags=23%, list=11%, signal=26% |
| 1347 | GO\_RESPONSE\_TO\_INTERLEUKIN\_1 |  | 102 | 0.42 | 1.13 | 0.285 | 0.458 | 1.000 | 3370 | tags=30%, list=15%, signal=36% |
| 1348 | GO\_POSITIVE\_REGULATION\_OF\_ALPHA\_BETA\_T\_CELL\_DIFFERENTIATION |  | 37 | 0.50 | 1.13 | 0.331 | 0.458 | 1.000 | 3783 | tags=38%, list=17%, signal=46% |
| 1349 | GO\_REGULATION\_OF\_STRESS\_ACTIVATED\_PROTEIN\_KINASE\_SIGNALING\_CASCADE |  | 190 | 0.33 | 1.13 | 0.172 | 0.459 | 1.000 | 1979 | tags=17%, list=9%, signal=19% |
| 1350 | GO\_SYNAPTIC\_VESICLE\_CYCLE |  | 82 | 0.36 | 1.12 | 0.230 | 0.459 | 1.000 | 2291 | tags=18%, list=11%, signal=20% |
| 1351 | GO\_REGULATION\_OF\_ANION\_TRANSPORT |  | 129 | 0.32 | 1.12 | 0.202 | 0.460 | 1.000 | 2481 | tags=16%, list=11%, signal=18% |
| 1352 | GO\_REGULATION\_OF\_MONONUCLEAR\_CELL\_MIGRATION |  | 16 | 0.60 | 1.12 | 0.329 | 0.461 | 1.000 | 3080 | tags=50%, list=14%, signal=58% |
| 1353 | GO\_REGULATION\_OF\_FATTY\_ACID\_METABOLIC\_PROCESS |  | 79 | 0.38 | 1.12 | 0.254 | 0.461 | 1.000 | 2376 | tags=18%, list=11%, signal=20% |
| 1354 | GO\_CELL\_MATURATION |  | 122 | 0.34 | 1.12 | 0.202 | 0.464 | 1.000 | 1823 | tags=16%, list=8%, signal=18% |
| 1355 | GO\_OLIGODENDROCYTE\_DIFFERENTIATION |  | 57 | 0.38 | 1.12 | 0.223 | 0.465 | 1.000 | 4266 | tags=30%, list=20%, signal=37% |
| 1356 | GO\_CELLULAR\_RESPONSE\_TO\_INSULIN\_STIMULUS |  | 140 | 0.31 | 1.12 | 0.238 | 0.465 | 1.000 | 3302 | tags=19%, list=15%, signal=23% |
| 1357 | GO\_SMOOTH\_MUSCLE\_CONTRACTION |  | 45 | 0.41 | 1.12 | 0.281 | 0.465 | 1.000 | 3785 | tags=33%, list=17%, signal=40% |
| 1358 | GO\_RESPONSE\_TO\_CAFFEINE |  | 18 | 0.54 | 1.12 | 0.277 | 0.467 | 1.000 | 2133 | tags=39%, list=10%, signal=43% |
| 1359 | GO\_MITOCHONDRION\_LOCALIZATION |  | 32 | 0.37 | 1.12 | 0.281 | 0.467 | 1.000 | 2966 | tags=19%, list=14%, signal=22% |
| 1360 | GO\_DEFENSE\_RESPONSE\_TO\_BACTERIUM |  | 192 | 0.41 | 1.12 | 0.290 | 0.467 | 1.000 | 4052 | tags=32%, list=19%, signal=39% |
| 1361 | GO\_POSITIVE\_REGULATION\_OF\_INNATE\_IMMUNE\_RESPONSE |  | 235 | 0.40 | 1.12 | 0.342 | 0.468 | 1.000 | 2735 | tags=21%, list=13%, signal=24% |
| 1362 | GO\_REGULATION\_OF\_LEUKOCYTE\_MIGRATION |  | 141 | 0.46 | 1.12 | 0.295 | 0.469 | 1.000 | 3478 | tags=38%, list=16%, signal=45% |
| 1363 | GO\_MULTICELLULAR\_ORGANISMAL\_MACROMOLECULE\_METABOLIC\_PROCESS |  | 73 | 0.51 | 1.12 | 0.337 | 0.469 | 1.000 | 2647 | tags=42%, list=12%, signal=48% |
| 1364 | GO\_POSITIVE\_REGULATION\_OF\_MYELOID\_LEUKOCYTE\_MEDIATED\_IMMUNITY |  | 18 | 0.51 | 1.12 | 0.323 | 0.469 | 1.000 | 2428 | tags=39%, list=11%, signal=44% |
| 1365 | GO\_REGULATION\_OF\_STEROL\_TRANSPORT |  | 37 | 0.40 | 1.12 | 0.292 | 0.470 | 1.000 | 3222 | tags=24%, list=15%, signal=29% |
| 1366 | GO\_REGULATION\_OF\_ACTIVATED\_T\_CELL\_PROLIFERATION |  | 39 | 0.48 | 1.12 | 0.308 | 0.470 | 1.000 | 3478 | tags=31%, list=16%, signal=37% |
| 1367 | GO\_NEGATIVE\_REGULATION\_OF\_PEPTIDYL\_TYROSINE\_PHOSPHORYLATION |  | 37 | 0.37 | 1.12 | 0.269 | 0.470 | 1.000 | 3561 | tags=27%, list=16%, signal=32% |
| 1368 | GO\_REGULATION\_OF\_EXECUTION\_PHASE\_OF\_APOPTOSIS |  | 23 | 0.44 | 1.12 | 0.301 | 0.470 | 1.000 | 2353 | tags=22%, list=11%, signal=24% |
| 1369 | GO\_KIDNEY\_MORPHOGENESIS |  | 81 | 0.41 | 1.12 | 0.267 | 0.471 | 1.000 | 3433 | tags=28%, list=16%, signal=34% |
| 1370 | GO\_REGULATION\_OF\_NEURON\_MIGRATION |  | 24 | 0.42 | 1.12 | 0.293 | 0.471 | 1.000 | 2670 | tags=25%, list=12%, signal=28% |
| 1371 | GO\_POSITIVE\_REGULATION\_OF\_CHEMOTAXIS |  | 117 | 0.46 | 1.11 | 0.308 | 0.472 | 1.000 | 3170 | tags=35%, list=15%, signal=41% |
| 1372 | GO\_POSITIVE\_REGULATION\_OF\_CYTOSKELETON\_ORGANIZATION |  | 167 | 0.32 | 1.11 | 0.223 | 0.472 | 1.000 | 3170 | tags=22%, list=15%, signal=26% |
| 1373 | GO\_SKELETAL\_SYSTEM\_MORPHOGENESIS |  | 195 | 0.39 | 1.11 | 0.284 | 0.473 | 1.000 | 3032 | tags=25%, list=14%, signal=28% |
| 1374 | GO\_ADULT\_BEHAVIOR |  | 129 | 0.34 | 1.11 | 0.257 | 0.472 | 1.000 | 3139 | tags=16%, list=14%, signal=18% |
| 1375 | GO\_POSITIVE\_REGULATION\_OF\_PROTEIN\_IMPORT |  | 99 | 0.38 | 1.11 | 0.257 | 0.472 | 1.000 | 3138 | tags=25%, list=14%, signal=29% |
| 1376 | GO\_POSITIVE\_REGULATION\_OF\_ERBB\_SIGNALING\_PATHWAY |  | 33 | 0.38 | 1.11 | 0.267 | 0.472 | 1.000 | 125 | tags=6%, list=1%, signal=6% |
| 1377 | GO\_RESPONSE\_TO\_BACTERIUM |  | 469 | 0.37 | 1.11 | 0.272 | 0.472 | 1.000 | 3783 | tags=29%, list=17%, signal=34% |
| 1378 | GO\_NEURON\_NEURON\_SYNAPTIC\_TRANSMISSION |  | 54 | 0.36 | 1.11 | 0.290 | 0.472 | 1.000 | 2648 | tags=15%, list=12%, signal=17% |
| 1379 | GO\_THYMOCYTE\_AGGREGATION |  | 44 | 0.41 | 1.11 | 0.312 | 0.472 | 1.000 | 4016 | tags=39%, list=18%, signal=47% |
| 1380 | GO\_SUBSTRATE\_DEPENDENT\_CELL\_MIGRATION |  | 26 | 0.43 | 1.11 | 0.309 | 0.472 | 1.000 | 2898 | tags=35%, list=13%, signal=40% |
| 1381 | GO\_NEGATIVE\_REGULATION\_OF\_RESPONSE\_TO\_EXTRACELLULAR\_STIMULUS |  | 32 | 0.34 | 1.11 | 0.278 | 0.472 | 1.000 | 3663 | tags=25%, list=17%, signal=30% |
| 1382 | GO\_POSITIVE\_REGULATION\_OF\_LEUKOCYTE\_DIFFERENTIATION |  | 124 | 0.42 | 1.11 | 0.324 | 0.472 | 1.000 | 3578 | tags=36%, list=16%, signal=43% |
| 1383 | GO\_REGULATION\_OF\_SYNAPTIC\_VESICLE\_TRANSPORT |  | 29 | 0.40 | 1.11 | 0.301 | 0.472 | 1.000 | 4387 | tags=28%, list=20%, signal=35% |
| 1384 | GO\_MACROPHAGE\_DIFFERENTIATION |  | 19 | 0.47 | 1.11 | 0.320 | 0.472 | 1.000 | 3222 | tags=42%, list=15%, signal=49% |
| 1385 | GO\_POSITIVE\_REGULATION\_OF\_PRODUCTION\_OF\_MOLECULAR\_MEDIATOR\_OF\_IMMUNE\_RESPONSE |  | 61 | 0.44 | 1.11 | 0.312 | 0.472 | 1.000 | 3241 | tags=33%, list=15%, signal=38% |
| 1386 | GO\_EMBRYONIC\_EYE\_MORPHOGENESIS |  | 33 | 0.45 | 1.11 | 0.281 | 0.472 | 1.000 | 1963 | tags=18%, list=9%, signal=20% |
| 1387 | GO\_REGULATION\_OF\_SYNAPSE\_ORGANIZATION |  | 100 | 0.41 | 1.11 | 0.270 | 0.472 | 1.000 | 2213 | tags=19%, list=10%, signal=21% |
| 1388 | GO\_POSITIVE\_REGULATION\_OF\_ATPASE\_ACTIVITY |  | 40 | 0.37 | 1.11 | 0.296 | 0.474 | 1.000 | 3142 | tags=25%, list=14%, signal=29% |
| 1389 | GO\_GLYCEROLIPID\_BIOSYNTHETIC\_PROCESS |  | 202 | 0.31 | 1.11 | 0.223 | 0.475 | 1.000 | 3969 | tags=22%, list=18%, signal=27% |
| 1390 | GO\_EMBRYONIC\_ORGAN\_MORPHOGENESIS |  | 271 | 0.35 | 1.11 | 0.268 | 0.474 | 1.000 | 3090 | tags=20%, list=14%, signal=23% |
| 1391 | GO\_TUBE\_MORPHOGENESIS |  | 317 | 0.36 | 1.11 | 0.280 | 0.475 | 1.000 | 3351 | tags=25%, list=15%, signal=29% |
| 1392 | GO\_RESPONSE\_TO\_COLD |  | 41 | 0.39 | 1.11 | 0.255 | 0.474 | 1.000 | 2107 | tags=17%, list=10%, signal=19% |
| 1393 | GO\_REGULATION\_OF\_NEUROTRANSMITTER\_UPTAKE |  | 15 | 0.53 | 1.11 | 0.357 | 0.475 | 1.000 | 1726 | tags=13%, list=8%, signal=14% |
| 1394 | GO\_POSITIVE\_REGULATION\_OF\_HEMOPOIESIS |  | 156 | 0.39 | 1.11 | 0.308 | 0.476 | 1.000 | 3578 | tags=33%, list=16%, signal=40% |
| 1395 | GO\_REGULATION\_OF\_LEUKOCYTE\_MEDIATED\_CYTOTOXICITY |  | 50 | 0.50 | 1.11 | 0.344 | 0.476 | 1.000 | 3478 | tags=40%, list=16%, signal=48% |
| 1396 | GO\_REGULATION\_OF\_TYPE\_2\_IMMUNE\_RESPONSE |  | 25 | 0.53 | 1.11 | 0.332 | 0.476 | 1.000 | 4338 | tags=52%, list=20%, signal=65% |
| 1397 | GO\_REGULATION\_OF\_ADAPTIVE\_IMMUNE\_RESPONSE |  | 121 | 0.42 | 1.11 | 0.316 | 0.476 | 1.000 | 3478 | tags=31%, list=16%, signal=37% |
| 1398 | GO\_INSULIN\_RECEPTOR\_SIGNALING\_PATHWAY |  | 76 | 0.32 | 1.11 | 0.262 | 0.476 | 1.000 | 3755 | tags=21%, list=17%, signal=25% |
| 1399 | GO\_NEGATIVE\_REGULATION\_OF\_CELLULAR\_CATABOLIC\_PROCESS |  | 145 | 0.28 | 1.11 | 0.231 | 0.476 | 1.000 | 3069 | tags=19%, list=14%, signal=22% |
| 1400 | GO\_REGULATION\_OF\_VACUOLE\_ORGANIZATION |  | 40 | 0.33 | 1.11 | 0.294 | 0.476 | 1.000 | 4387 | tags=25%, list=20%, signal=31% |
| 1401 | GO\_NEGATIVE\_REGULATION\_OF\_CATABOLIC\_PROCESS |  | 189 | 0.28 | 1.11 | 0.194 | 0.477 | 1.000 | 3563 | tags=22%, list=16%, signal=26% |
| 1402 | GO\_POSITIVE\_REGULATION\_OF\_CELLULAR\_COMPONENT\_BIOGENESIS |  | 369 | 0.30 | 1.11 | 0.210 | 0.477 | 1.000 | 3408 | tags=21%, list=16%, signal=24% |
| 1403 | GO\_CELLULAR\_PROTEIN\_COMPLEX\_ASSEMBLY |  | 310 | 0.27 | 1.11 | 0.235 | 0.477 | 1.000 | 2184 | tags=11%, list=10%, signal=12% |
| 1404 | GO\_PLASMA\_LIPOPROTEIN\_PARTICLE\_CLEARANCE |  | 21 | 0.50 | 1.11 | 0.321 | 0.477 | 1.000 | 3166 | tags=33%, list=15%, signal=39% |
| 1405 | GO\_POSITIVE\_REGULATION\_OF\_CELL\_MORPHOGENESIS\_INVOLVED\_IN\_DIFFERENTIATION |  | 155 | 0.35 | 1.11 | 0.284 | 0.477 | 1.000 | 3024 | tags=25%, list=14%, signal=28% |
| 1406 | GO\_REGULATION\_OF\_BONE\_REMODELING |  | 39 | 0.42 | 1.11 | 0.298 | 0.477 | 1.000 | 3547 | tags=28%, list=16%, signal=34% |
| 1407 | GO\_NEGATIVE\_REGULATION\_OF\_PROTEOLYSIS |  | 300 | 0.31 | 1.11 | 0.240 | 0.477 | 1.000 | 2918 | tags=18%, list=13%, signal=21% |
| 1408 | GO\_REGULATION\_OF\_TOLL\_LIKE\_RECEPTOR\_SIGNALING\_PATHWAY |  | 45 | 0.47 | 1.11 | 0.331 | 0.477 | 1.000 | 3800 | tags=38%, list=17%, signal=46% |
| 1409 | GO\_POSITIVE\_REGULATION\_OF\_PROTEIN\_TYROSINE\_KINASE\_ACTIVITY |  | 34 | 0.40 | 1.11 | 0.239 | 0.477 | 1.000 | 3138 | tags=32%, list=14%, signal=38% |
| 1410 | GO\_TRANSITION\_METAL\_ION\_HOMEOSTASIS |  | 102 | 0.32 | 1.11 | 0.280 | 0.477 | 1.000 | 3775 | tags=22%, list=17%, signal=26% |
| 1411 | GO\_GLYCOSYL\_COMPOUND\_CATABOLIC\_PROCESS |  | 39 | 0.39 | 1.10 | 0.269 | 0.477 | 1.000 | 3308 | tags=21%, list=15%, signal=24% |
| 1412 | GO\_REGULATION\_OF\_PEPTIDYL\_SERINE\_PHOSPHORYLATION |  | 113 | 0.33 | 1.10 | 0.257 | 0.477 | 1.000 | 3755 | tags=26%, list=17%, signal=31% |
| 1413 | GO\_REGULATION\_OF\_INTERLEUKIN\_1\_SECRETION |  | 28 | 0.52 | 1.10 | 0.357 | 0.477 | 1.000 | 3241 | tags=36%, list=15%, signal=42% |
| 1414 | GO\_POSITIVE\_REGULATION\_OF\_FIBROBLAST\_PROLIFERATION |  | 51 | 0.40 | 1.10 | 0.300 | 0.477 | 1.000 | 1913 | tags=20%, list=9%, signal=21% |
| 1415 | GO\_POSITIVE\_REGULATION\_OF\_STEROID\_METABOLIC\_PROCESS |  | 23 | 0.48 | 1.10 | 0.277 | 0.477 | 1.000 | 3084 | tags=30%, list=14%, signal=35% |
| 1416 | GO\_CELLULAR\_RESPONSE\_TO\_EXTERNAL\_STIMULUS |  | 252 | 0.32 | 1.10 | 0.195 | 0.477 | 1.000 | 3293 | tags=23%, list=15%, signal=27% |
| 1417 | GO\_REGULATION\_OF\_DOPAMINE\_SECRETION |  | 22 | 0.46 | 1.10 | 0.328 | 0.478 | 1.000 | 2386 | tags=14%, list=11%, signal=15% |
| 1418 | GO\_REGULATION\_OF\_LEUKOCYTE\_CHEMOTAXIS |  | 91 | 0.49 | 1.10 | 0.329 | 0.477 | 1.000 | 3170 | tags=40%, list=15%, signal=46% |
| 1419 | GO\_NEGATIVE\_REGULATION\_OF\_ANION\_TRANSPORT |  | 31 | 0.44 | 1.10 | 0.299 | 0.478 | 1.000 | 1925 | tags=23%, list=9%, signal=25% |
| 1420 | GO\_RESPONSE\_TO\_HEAT |  | 85 | 0.38 | 1.10 | 0.290 | 0.478 | 1.000 | 2281 | tags=18%, list=10%, signal=20% |
| 1421 | GO\_SPLEEN\_DEVELOPMENT |  | 37 | 0.40 | 1.10 | 0.277 | 0.478 | 1.000 | 3478 | tags=32%, list=16%, signal=39% |
| 1422 | GO\_REGULATION\_OF\_INCLUSION\_BODY\_ASSEMBLY |  | 15 | 0.46 | 1.10 | 0.307 | 0.478 | 1.000 | 1925 | tags=20%, list=9%, signal=22% |
| 1423 | GO\_NEURON\_PROJECTION\_REGENERATION |  | 30 | 0.44 | 1.10 | 0.306 | 0.478 | 1.000 | 2442 | tags=23%, list=11%, signal=26% |
| 1424 | GO\_POSITIVE\_REGULATION\_OF\_PROTEIN\_SERINE\_THREONINE\_KINASE\_ACTIVITY |  | 272 | 0.31 | 1.10 | 0.236 | 0.479 | 1.000 | 3404 | tags=26%, list=16%, signal=30% |
| 1425 | GO\_POST\_EMBRYONIC\_DEVELOPMENT |  | 86 | 0.32 | 1.10 | 0.258 | 0.479 | 1.000 | 3630 | tags=28%, list=17%, signal=33% |
| 1426 | GO\_SPROUTING\_ANGIOGENESIS |  | 42 | 0.44 | 1.10 | 0.309 | 0.480 | 1.000 | 3298 | tags=43%, list=15%, signal=50% |
| 1427 | GO\_MEMBRANE\_LIPID\_CATABOLIC\_PROCESS |  | 22 | 0.41 | 1.10 | 0.317 | 0.480 | 1.000 | 4354 | tags=32%, list=20%, signal=40% |
| 1428 | GO\_CELL\_KILLING |  | 50 | 0.44 | 1.10 | 0.336 | 0.479 | 1.000 | 2917 | tags=24%, list=13%, signal=28% |
| 1429 | GO\_MEMBRANE\_BIOGENESIS |  | 30 | 0.41 | 1.10 | 0.293 | 0.479 | 1.000 | 4442 | tags=33%, list=20%, signal=42% |
| 1430 | GO\_MULTICELLULAR\_ORGANISMAL\_HOMEOSTASIS |  | 252 | 0.35 | 1.10 | 0.281 | 0.480 | 1.000 | 3824 | tags=27%, list=18%, signal=32% |
| 1431 | GO\_POSITIVE\_REGULATION\_OF\_LYASE\_ACTIVITY |  | 55 | 0.35 | 1.10 | 0.300 | 0.480 | 1.000 | 3691 | tags=24%, list=17%, signal=28% |
| 1432 | GO\_DERMATAN\_SULFATE\_PROTEOGLYCAN\_METABOLIC\_PROCESS |  | 16 | 0.56 | 1.10 | 0.379 | 0.480 | 1.000 | 3778 | tags=50%, list=17%, signal=60% |
| 1433 | GO\_REGULATION\_OF\_FIBROBLAST\_GROWTH\_FACTOR\_RECEPTOR\_SIGNALING\_PATHWAY |  | 24 | 0.45 | 1.10 | 0.308 | 0.480 | 1.000 | 1948 | tags=21%, list=9%, signal=23% |
| 1434 | GO\_REGULATION\_OF\_CD4\_POSITIVE\_ALPHA\_BETA\_T\_CELL\_ACTIVATION |  | 38 | 0.50 | 1.10 | 0.352 | 0.483 | 1.000 | 3406 | tags=37%, list=16%, signal=44% |
| 1435 | GO\_REGULATION\_OF\_STAT\_CASCADE |  | 141 | 0.34 | 1.10 | 0.238 | 0.484 | 1.000 | 3170 | tags=19%, list=15%, signal=22% |
| 1436 | GO\_SKELETAL\_MUSCLE\_TISSUE\_REGENERATION |  | 25 | 0.47 | 1.10 | 0.350 | 0.484 | 1.000 | 1347 | tags=20%, list=6%, signal=21% |
| 1437 | GO\_PURINE\_CONTAINING\_COMPOUND\_SALVAGE |  | 16 | 0.46 | 1.10 | 0.321 | 0.484 | 1.000 | 267 | tags=13%, list=1%, signal=13% |
| 1438 | GO\_CELLULAR\_RESPONSE\_TO\_ESTROGEN\_STIMULUS |  | 38 | 0.39 | 1.10 | 0.287 | 0.484 | 1.000 | 1926 | tags=16%, list=9%, signal=17% |
| 1439 | GO\_NEURAL\_CREST\_CELL\_DIFFERENTIATION |  | 74 | 0.40 | 1.10 | 0.297 | 0.485 | 1.000 | 3194 | tags=23%, list=15%, signal=27% |
| 1440 | GO\_GLYCOPROTEIN\_METABOLIC\_PROCESS |  | 332 | 0.33 | 1.10 | 0.276 | 0.486 | 1.000 | 3802 | tags=24%, list=17%, signal=29% |
| 1441 | GO\_INOSITOL\_PHOSPHATE\_MEDIATED\_SIGNALING |  | 18 | 0.44 | 1.10 | 0.352 | 0.485 | 1.000 | 3697 | tags=44%, list=17%, signal=54% |
| 1442 | GO\_AMMONIUM\_TRANSPORT |  | 54 | 0.39 | 1.10 | 0.292 | 0.485 | 1.000 | 3891 | tags=28%, list=18%, signal=34% |
| 1443 | GO\_REGULATION\_OF\_AUTOPHAGOSOME\_ASSEMBLY |  | 34 | 0.34 | 1.10 | 0.317 | 0.485 | 1.000 | 3451 | tags=21%, list=16%, signal=24% |
| 1444 | GO\_RHYTHMIC\_PROCESS |  | 285 | 0.31 | 1.09 | 0.253 | 0.486 | 1.000 | 2956 | tags=19%, list=14%, signal=22% |
| 1445 | GO\_POLYOL\_TRANSPORT |  | 16 | 0.46 | 1.09 | 0.337 | 0.486 | 1.000 | 2965 | tags=25%, list=14%, signal=29% |
| 1446 | GO\_REGULATION\_OF\_SYNAPSE\_STRUCTURE\_OR\_ACTIVITY |  | 216 | 0.33 | 1.09 | 0.285 | 0.486 | 1.000 | 2250 | tags=14%, list=10%, signal=16% |
| 1447 | GO\_PHARYNGEAL\_SYSTEM\_DEVELOPMENT |  | 16 | 0.51 | 1.09 | 0.337 | 0.486 | 1.000 | 3353 | tags=31%, list=15%, signal=37% |
| 1448 | GO\_POSITIVE\_REGULATION\_OF\_STRESS\_ACTIVATED\_PROTEIN\_KINASE\_SIGNALING\_CASCADE |  | 129 | 0.34 | 1.09 | 0.259 | 0.490 | 1.000 | 1979 | tags=19%, list=9%, signal=21% |
| 1449 | GO\_POSITIVE\_REGULATION\_OF\_SMOOTH\_MUSCLE\_CELL\_PROLIFERATION |  | 59 | 0.39 | 1.09 | 0.307 | 0.490 | 1.000 | 1924 | tags=22%, list=9%, signal=24% |
| 1450 | GO\_REGULATION\_OF\_TOR\_SIGNALING |  | 65 | 0.31 | 1.09 | 0.292 | 0.491 | 1.000 | 3222 | tags=18%, list=15%, signal=22% |
| 1451 | GO\_REGULATION\_OF\_CHEMOKINE\_PRODUCTION |  | 61 | 0.41 | 1.09 | 0.310 | 0.491 | 1.000 | 2059 | tags=25%, list=9%, signal=27% |
| 1452 | GO\_POSITIVE\_REGULATION\_OF\_REACTIVE\_OXYGEN\_SPECIES\_BIOSYNTHETIC\_PROCESS |  | 47 | 0.43 | 1.09 | 0.345 | 0.491 | 1.000 | 3170 | tags=38%, list=15%, signal=45% |
| 1453 | GO\_REGULATION\_OF\_PROTEIN\_STABILITY |  | 204 | 0.26 | 1.09 | 0.264 | 0.492 | 1.000 | 2500 | tags=14%, list=11%, signal=15% |
| 1454 | GO\_GASTRULATION |  | 144 | 0.35 | 1.09 | 0.306 | 0.492 | 1.000 | 3049 | tags=19%, list=14%, signal=22% |
| 1455 | GO\_POSITIVE\_REGULATION\_OF\_LYMPHOCYTE\_MIGRATION |  | 25 | 0.55 | 1.09 | 0.390 | 0.492 | 1.000 | 1783 | tags=32%, list=8%, signal=35% |
| 1456 | GO\_REGULATION\_OF\_FIBROBLAST\_PROLIFERATION |  | 78 | 0.36 | 1.09 | 0.311 | 0.493 | 1.000 | 2912 | tags=21%, list=13%, signal=24% |
| 1457 | GO\_CARTILAGE\_DEVELOPMENT\_INVOLVED\_IN\_ENDOCHONDRAL\_BONE\_MORPHOGENESIS |  | 19 | 0.51 | 1.09 | 0.371 | 0.496 | 1.000 | 2125 | tags=32%, list=10%, signal=35% |
| 1458 | GO\_POSITIVE\_REGULATION\_OF\_CELLULAR\_RESPONSE\_TO\_INSULIN\_STIMULUS |  | 20 | 0.41 | 1.09 | 0.324 | 0.496 | 1.000 | 3755 | tags=35%, list=17%, signal=42% |
| 1459 | GO\_REGULATION\_OF\_PROTEIN\_LOCALIZATION\_TO\_CELL\_SURFACE |  | 25 | 0.43 | 1.09 | 0.319 | 0.496 | 1.000 | 3042 | tags=32%, list=14%, signal=37% |
| 1460 | GO\_DIGESTIVE\_TRACT\_MORPHOGENESIS |  | 48 | 0.42 | 1.09 | 0.313 | 0.496 | 1.000 | 3130 | tags=33%, list=14%, signal=39% |
| 1461 | GO\_REGULATION\_OF\_NOTCH\_SIGNALING\_PATHWAY |  | 63 | 0.38 | 1.09 | 0.294 | 0.496 | 1.000 | 2732 | tags=22%, list=13%, signal=25% |
| 1462 | GO\_ENDOTHELIAL\_CELL\_DIFFERENTIATION |  | 70 | 0.37 | 1.09 | 0.340 | 0.496 | 1.000 | 2898 | tags=29%, list=13%, signal=33% |
| 1463 | GO\_NITRIC\_OXIDE\_METABOLIC\_PROCESS |  | 15 | 0.51 | 1.09 | 0.325 | 0.496 | 1.000 | 1697 | tags=33%, list=8%, signal=36% |
| 1464 | GO\_REGULATION\_OF\_PATHWAY\_RESTRICTED\_SMAD\_PROTEIN\_PHOSPHORYLATION |  | 56 | 0.40 | 1.09 | 0.341 | 0.496 | 1.000 | 1687 | tags=13%, list=8%, signal=14% |
| 1465 | GO\_PHOSPHATIDYLINOSITOL\_BIOSYNTHETIC\_PROCESS |  | 117 | 0.29 | 1.08 | 0.278 | 0.499 | 1.000 | 3066 | tags=18%, list=14%, signal=21% |
| 1466 | GO\_SENSORY\_PERCEPTION\_OF\_PAIN |  | 73 | 0.38 | 1.08 | 0.282 | 0.501 | 1.000 | 2441 | tags=21%, list=11%, signal=23% |
| 1467 | GO\_RESPONSE\_TO\_ALKALOID |  | 132 | 0.35 | 1.08 | 0.291 | 0.501 | 1.000 | 2133 | tags=17%, list=10%, signal=18% |
| 1468 | GO\_GOLGI\_ORGANIZATION |  | 83 | 0.29 | 1.08 | 0.303 | 0.504 | 1.000 | 3899 | tags=20%, list=18%, signal=25% |
| 1469 | GO\_REGULATION\_OF\_RESPONSE\_TO\_BIOTIC\_STIMULUS |  | 220 | 0.33 | 1.08 | 0.319 | 0.504 | 1.000 | 2059 | tags=15%, list=9%, signal=16% |
| 1470 | GO\_MODULATION\_BY\_SYMBIONT\_OF\_HOST\_CELLULAR\_PROCESS |  | 26 | 0.38 | 1.08 | 0.369 | 0.504 | 1.000 | 1868 | tags=15%, list=9%, signal=17% |
| 1471 | GO\_FLUID\_TRANSPORT |  | 25 | 0.44 | 1.08 | 0.315 | 0.504 | 1.000 | 3032 | tags=24%, list=14%, signal=28% |
| 1472 | GO\_LONG\_CHAIN\_FATTY\_ACID\_TRANSPORT |  | 40 | 0.41 | 1.08 | 0.326 | 0.505 | 1.000 | 2714 | tags=25%, list=12%, signal=29% |
| 1473 | GO\_RESPONSE\_TO\_MOLECULE\_OF\_BACTERIAL\_ORIGIN |  | 306 | 0.38 | 1.08 | 0.311 | 0.505 | 1.000 | 2841 | tags=25%, list=13%, signal=28% |
| 1474 | GO\_REGULATION\_OF\_T\_CELL\_DIFFERENTIATION |  | 103 | 0.42 | 1.08 | 0.329 | 0.505 | 1.000 | 3526 | tags=35%, list=16%, signal=42% |
| 1475 | GO\_LUNG\_ALVEOLUS\_DEVELOPMENT |  | 41 | 0.41 | 1.08 | 0.324 | 0.505 | 1.000 | 3309 | tags=27%, list=15%, signal=32% |
| 1476 | GO\_NEGATIVE\_REGULATION\_OF\_ADAPTIVE\_IMMUNE\_RESPONSE |  | 37 | 0.46 | 1.08 | 0.375 | 0.505 | 1.000 | 3457 | tags=32%, list=16%, signal=38% |
| 1477 | GO\_ANTERIOR\_POSTERIOR\_PATTERN\_SPECIFICATION |  | 184 | 0.36 | 1.08 | 0.311 | 0.505 | 1.000 | 2116 | tags=17%, list=10%, signal=19% |
| 1478 | GO\_POSITIVE\_REGULATION\_OF\_CATABOLIC\_PROCESS |  | 379 | 0.25 | 1.08 | 0.268 | 0.505 | 1.000 | 3389 | tags=17%, list=16%, signal=20% |
| 1479 | GO\_POSITIVE\_REGULATION\_OF\_HEART\_GROWTH |  | 26 | 0.46 | 1.08 | 0.337 | 0.506 | 1.000 | 2451 | tags=31%, list=11%, signal=35% |
| 1480 | GO\_NUCLEOBASE\_CONTAINING\_SMALL\_MOLECULE\_METABOLIC\_PROCESS |  | 484 | 0.27 | 1.08 | 0.283 | 0.506 | 1.000 | 1970 | tags=10%, list=9%, signal=11% |
| 1481 | GO\_GLUCOSE\_METABOLIC\_PROCESS |  | 113 | 0.34 | 1.08 | 0.299 | 0.507 | 1.000 | 1773 | tags=14%, list=8%, signal=15% |
| 1482 | GO\_LEUKOCYTE\_DEGRANULATION |  | 28 | 0.46 | 1.08 | 0.378 | 0.507 | 1.000 | 2920 | tags=32%, list=13%, signal=37% |
| 1483 | GO\_REGULATION\_OF\_INTERFERON\_ALPHA\_PRODUCTION |  | 19 | 0.57 | 1.08 | 0.443 | 0.507 | 1.000 | 2059 | tags=26%, list=9%, signal=29% |
| 1484 | GO\_EMBRYONIC\_PATTERN\_SPECIFICATION |  | 55 | 0.37 | 1.08 | 0.312 | 0.507 | 1.000 | 718 | tags=9%, list=3%, signal=9% |
| 1485 | GO\_REGULATION\_OF\_MEMBRANE\_PROTEIN\_ECTODOMAIN\_PROTEOLYSIS |  | 20 | 0.49 | 1.08 | 0.333 | 0.508 | 1.000 | 3146 | tags=50%, list=14%, signal=58% |
| 1486 | GO\_BRANCHING\_INVOLVED\_IN\_SALIVARY\_GLAND\_MORPHOGENESIS |  | 16 | 0.47 | 1.08 | 0.359 | 0.507 | 1.000 | 2898 | tags=31%, list=13%, signal=36% |
| 1487 | GO\_EMBRYONIC\_CAMERA\_TYPE\_EYE\_DEVELOPMENT |  | 35 | 0.41 | 1.08 | 0.324 | 0.508 | 1.000 | 2259 | tags=17%, list=10%, signal=19% |
| 1488 | GO\_POSITIVE\_REGULATION\_OF\_CD4\_POSITIVE\_ALPHA\_BETA\_T\_CELL\_ACTIVATION |  | 27 | 0.52 | 1.08 | 0.398 | 0.508 | 1.000 | 804 | tags=22%, list=4%, signal=23% |
| 1489 | GO\_REGULATION\_OF\_EPITHELIAL\_CELL\_DIFFERENTIATION |  | 119 | 0.32 | 1.08 | 0.264 | 0.508 | 1.000 | 3084 | tags=19%, list=14%, signal=22% |
| 1490 | GO\_LYMPHOCYTE\_MIGRATION |  | 43 | 0.56 | 1.08 | 0.394 | 0.507 | 1.000 | 3125 | tags=49%, list=14%, signal=57% |
| 1491 | GO\_REGULATION\_OF\_PROTEIN\_IMPORT\_INTO\_NUCLEUS\_TRANSLOCATION |  | 21 | 0.43 | 1.08 | 0.337 | 0.507 | 1.000 | 3222 | tags=29%, list=15%, signal=34% |
| 1492 | GO\_ENDOPLASMIC\_RETICULUM\_ORGANIZATION |  | 36 | 0.37 | 1.08 | 0.341 | 0.507 | 1.000 | 1160 | tags=11%, list=5%, signal=12% |
| 1493 | GO\_NEURAL\_TUBE\_FORMATION |  | 94 | 0.35 | 1.08 | 0.318 | 0.507 | 1.000 | 3351 | tags=21%, list=15%, signal=25% |
| 1494 | GO\_IRON\_ION\_HOMEOSTASIS |  | 65 | 0.36 | 1.07 | 0.332 | 0.508 | 1.000 | 3775 | tags=23%, list=17%, signal=28% |
| 1495 | GO\_NEGATIVE\_REGULATION\_OF\_MAP\_KINASE\_ACTIVITY |  | 71 | 0.37 | 1.07 | 0.307 | 0.507 | 1.000 | 3032 | tags=23%, list=14%, signal=26% |
| 1496 | GO\_CELLULAR\_RESPONSE\_TO\_ABIOTIC\_STIMULUS |  | 249 | 0.29 | 1.07 | 0.290 | 0.507 | 1.000 | 3035 | tags=16%, list=14%, signal=19% |
| 1497 | GO\_REGULATION\_OF\_DEFENSE\_RESPONSE\_TO\_VIRUS\_BY\_VIRUS |  | 29 | 0.43 | 1.07 | 0.385 | 0.508 | 1.000 | 2764 | tags=31%, list=13%, signal=36% |
| 1498 | GO\_REGULATION\_OF\_MESENCHYMAL\_CELL\_PROLIFERATION |  | 34 | 0.46 | 1.07 | 0.366 | 0.511 | 1.000 | 3309 | tags=41%, list=15%, signal=48% |
| 1499 | GO\_NEGATIVE\_REGULATION\_OF\_TRANSFERASE\_ACTIVITY |  | 328 | 0.27 | 1.07 | 0.280 | 0.511 | 1.000 | 2784 | tags=14%, list=13%, signal=16% |
| 1500 | GO\_HEMATOPOIETIC\_PROGENITOR\_CELL\_DIFFERENTIATION |  | 96 | 0.34 | 1.07 | 0.298 | 0.511 | 1.000 | 3101 | tags=22%, list=14%, signal=25% |
| 1501 | GO\_LIPID\_LOCALIZATION |  | 247 | 0.31 | 1.07 | 0.279 | 0.513 | 1.000 | 2389 | tags=14%, list=11%, signal=16% |
| 1502 | GO\_REGULATION\_OF\_LIPID\_TRANSPORT |  | 92 | 0.32 | 1.07 | 0.303 | 0.513 | 1.000 | 3755 | tags=23%, list=17%, signal=27% |
| 1503 | GO\_PROTEIN\_OLIGOMERIZATION |  | 403 | 0.27 | 1.07 | 0.248 | 0.513 | 1.000 | 2507 | tags=14%, list=12%, signal=16% |
| 1504 | GO\_REGULATION\_OF\_DNA\_BINDING |  | 90 | 0.32 | 1.07 | 0.318 | 0.515 | 1.000 | 3406 | tags=22%, list=16%, signal=26% |
| 1505 | GO\_REGULATION\_OF\_INSULIN\_SECRETION\_INVOLVED\_IN\_CELLULAR\_RESPONSE\_TO\_GLUCOSE\_STIMULUS |  | 50 | 0.35 | 1.07 | 0.329 | 0.515 | 1.000 | 2578 | tags=16%, list=12%, signal=18% |
| 1506 | GO\_COCHLEA\_DEVELOPMENT |  | 38 | 0.42 | 1.07 | 0.367 | 0.515 | 1.000 | 2732 | tags=26%, list=13%, signal=30% |
| 1507 | GO\_POSITIVE\_REGULATION\_OF\_MAP\_KINASE\_ACTIVITY |  | 197 | 0.32 | 1.07 | 0.295 | 0.515 | 1.000 | 3404 | tags=26%, list=16%, signal=30% |
| 1508 | GO\_ACTIVATION\_OF\_PROTEIN\_KINASE\_ACTIVITY |  | 268 | 0.30 | 1.07 | 0.282 | 0.515 | 1.000 | 3561 | tags=25%, list=16%, signal=30% |
| 1509 | GO\_PROTEIN\_HOMOTRIMERIZATION |  | 19 | 0.43 | 1.07 | 0.344 | 0.514 | 1.000 | 5686 | tags=53%, list=26%, signal=71% |
| 1510 | GO\_REGULATION\_OF\_BONE\_RESORPTION |  | 31 | 0.43 | 1.07 | 0.326 | 0.514 | 1.000 | 2646 | tags=23%, list=12%, signal=26% |
| 1511 | GO\_PROTEIN\_HOMOTETRAMERIZATION |  | 57 | 0.34 | 1.07 | 0.333 | 0.517 | 1.000 | 2477 | tags=18%, list=11%, signal=20% |
| 1512 | GO\_PHOSPHOLIPID\_CATABOLIC\_PROCESS |  | 28 | 0.42 | 1.07 | 0.371 | 0.517 | 1.000 | 5085 | tags=43%, list=23%, signal=56% |
| 1513 | GO\_NEGATIVE\_REGULATION\_OF\_GLYCOPROTEIN\_METABOLIC\_PROCESS |  | 15 | 0.45 | 1.07 | 0.367 | 0.517 | 1.000 | 3289 | tags=40%, list=15%, signal=47% |
| 1514 | GO\_BRANCHING\_INVOLVED\_IN\_URETERIC\_BUD\_MORPHOGENESIS |  | 44 | 0.43 | 1.07 | 0.336 | 0.517 | 1.000 | 3138 | tags=27%, list=14%, signal=32% |
| 1515 | GO\_ORGANELLE\_DISASSEMBLY |  | 176 | 0.27 | 1.07 | 0.292 | 0.517 | 1.000 | 3097 | tags=18%, list=14%, signal=20% |
| 1516 | GO\_REGULATION\_OF\_ACTIN\_CYTOSKELETON\_REORGANIZATION |  | 31 | 0.39 | 1.07 | 0.364 | 0.520 | 1.000 | 3561 | tags=39%, list=16%, signal=46% |
| 1517 | GO\_NEGATIVE\_REGULATION\_OF\_CELLULAR\_AMIDE\_METABOLIC\_PROCESS |  | 117 | 0.27 | 1.06 | 0.336 | 0.519 | 1.000 | 3889 | tags=21%, list=18%, signal=26% |
| 1518 | GO\_REGULATION\_OF\_STEM\_CELL\_PROLIFERATION |  | 84 | 0.37 | 1.06 | 0.362 | 0.522 | 1.000 | 3330 | tags=30%, list=15%, signal=35% |
| 1519 | GO\_NEGATIVE\_REGULATION\_OF\_CD4\_POSITIVE\_ALPHA\_BETA\_T\_CELL\_ACTIVATION |  | 15 | 0.49 | 1.06 | 0.391 | 0.521 | 1.000 | 3176 | tags=33%, list=15%, signal=39% |
| 1520 | GO\_METANEPHRIC\_NEPHRON\_DEVELOPMENT |  | 31 | 0.44 | 1.06 | 0.356 | 0.522 | 1.000 | 3267 | tags=32%, list=15%, signal=38% |
| 1521 | GO\_RESPONSE\_TO\_STARVATION |  | 145 | 0.32 | 1.06 | 0.306 | 0.522 | 1.000 | 3069 | tags=20%, list=14%, signal=23% |
| 1522 | GO\_PYRIMIDINE\_RIBONUCLEOSIDE\_METABOLIC\_PROCESS |  | 26 | 0.38 | 1.06 | 0.336 | 0.523 | 1.000 | 3844 | tags=27%, list=18%, signal=33% |
| 1523 | GO\_MEMBRANE\_ASSEMBLY |  | 25 | 0.42 | 1.06 | 0.360 | 0.525 | 1.000 | 4442 | tags=36%, list=20%, signal=45% |
| 1524 | GO\_REGULATION\_OF\_CARDIAC\_MUSCLE\_TISSUE\_DEVELOPMENT |  | 46 | 0.42 | 1.06 | 0.380 | 0.526 | 1.000 | 3290 | tags=33%, list=15%, signal=38% |
| 1525 | GO\_REGULATION\_OF\_LONG\_TERM\_SYNAPTIC\_POTENTIATION |  | 19 | 0.45 | 1.06 | 0.397 | 0.526 | 1.000 | 6050 | tags=42%, list=28%, signal=58% |
| 1526 | GO\_B\_CELL\_ACTIVATION\_INVOLVED\_IN\_IMMUNE\_RESPONSE |  | 34 | 0.41 | 1.06 | 0.370 | 0.526 | 1.000 | 3308 | tags=35%, list=15%, signal=42% |
| 1527 | GO\_REGULATION\_OF\_SULFUR\_METABOLIC\_PROCESS |  | 19 | 0.38 | 1.06 | 0.353 | 0.527 | 1.000 | 3534 | tags=21%, list=16%, signal=25% |
| 1528 | GO\_EPITHELIAL\_STRUCTURE\_MAINTENANCE |  | 21 | 0.44 | 1.06 | 0.367 | 0.527 | 1.000 | 409 | tags=10%, list=2%, signal=10% |
| 1529 | GO\_POSITIVE\_REGULATION\_OF\_EPITHELIAL\_CELL\_MIGRATION |  | 99 | 0.38 | 1.06 | 0.382 | 0.526 | 1.000 | 3170 | tags=29%, list=15%, signal=34% |
| 1530 | GO\_NEGATIVE\_REGULATION\_OF\_MAPK\_CASCADE |  | 139 | 0.33 | 1.06 | 0.341 | 0.527 | 1.000 | 2734 | tags=19%, list=13%, signal=22% |
| 1531 | GO\_ORGANIC\_ACID\_BIOSYNTHETIC\_PROCESS |  | 246 | 0.31 | 1.06 | 0.309 | 0.527 | 1.000 | 2478 | tags=16%, list=11%, signal=18% |
| 1532 | GO\_RESPONSE\_TO\_CORTICOSTEROID |  | 171 | 0.33 | 1.06 | 0.327 | 0.528 | 1.000 | 2504 | tags=19%, list=12%, signal=22% |
| 1533 | GO\_CELLULAR\_METABOLIC\_COMPOUND\_SALVAGE |  | 35 | 0.37 | 1.06 | 0.365 | 0.528 | 1.000 | 2107 | tags=17%, list=10%, signal=19% |
| 1534 | GO\_RESPIRATORY\_SYSTEM\_DEVELOPMENT |  | 192 | 0.35 | 1.06 | 0.355 | 0.528 | 1.000 | 2297 | tags=18%, list=11%, signal=20% |
| 1535 | GO\_FACE\_DEVELOPMENT |  | 49 | 0.36 | 1.06 | 0.358 | 0.528 | 1.000 | 4292 | tags=35%, list=20%, signal=43% |
| 1536 | GO\_NEGATIVE\_REGULATION\_OF\_LYMPHOCYTE\_DIFFERENTIATION |  | 39 | 0.37 | 1.06 | 0.341 | 0.530 | 1.000 | 3176 | tags=26%, list=15%, signal=30% |
| 1537 | GO\_POSITIVE\_REGULATION\_OF\_ANION\_TRANSPORT |  | 55 | 0.36 | 1.06 | 0.370 | 0.530 | 1.000 | 2464 | tags=20%, list=11%, signal=22% |
| 1538 | GO\_POSITIVE\_REGULATION\_OF\_TRANSCRIPTION\_FROM\_RNA\_POLYMERASE\_II\_PROMOTER\_INVOLVED\_IN\_CELLULAR\_RESPONSE\_TO\_CHEMICAL\_STIMULUS |  | 26 | 0.38 | 1.05 | 0.363 | 0.532 | 1.000 | 2478 | tags=23%, list=11%, signal=26% |
| 1539 | GO\_COLUMNAR\_CUBOIDAL\_EPITHELIAL\_CELL\_DIFFERENTIATION |  | 107 | 0.34 | 1.05 | 0.321 | 0.532 | 1.000 | 3967 | tags=25%, list=18%, signal=31% |
| 1540 | GO\_REGULATION\_OF\_MULTICELLULAR\_ORGANISM\_GROWTH |  | 65 | 0.29 | 1.05 | 0.344 | 0.532 | 1.000 | 3823 | tags=18%, list=18%, signal=22% |
| 1541 | GO\_CELL\_DEATH\_IN\_RESPONSE\_TO\_OXIDATIVE\_STRESS |  | 17 | 0.42 | 1.05 | 0.386 | 0.534 | 1.000 | 2966 | tags=35%, list=14%, signal=41% |
| 1542 | GO\_TELENCEPHALON\_GLIAL\_CELL\_MIGRATION |  | 18 | 0.40 | 1.05 | 0.377 | 0.534 | 1.000 | 3310 | tags=22%, list=15%, signal=26% |
| 1543 | GO\_SODIUM\_ION\_HOMEOSTASIS |  | 28 | 0.44 | 1.05 | 0.404 | 0.534 | 1.000 | 1879 | tags=14%, list=9%, signal=16% |
| 1544 | GO\_SEMAPHORIN\_PLEXIN\_SIGNALING\_PATHWAY |  | 36 | 0.39 | 1.05 | 0.386 | 0.535 | 1.000 | 3353 | tags=25%, list=15%, signal=30% |
| 1545 | GO\_POSITIVE\_REGULATION\_OF\_SYNAPTIC\_TRANSMISSION\_GLUTAMATERGIC |  | 17 | 0.45 | 1.05 | 0.359 | 0.535 | 1.000 | 3957 | tags=41%, list=18%, signal=50% |
| 1546 | GO\_REGULATION\_OF\_ENDOPLASMIC\_RETICULUM\_UNFOLDED\_PROTEIN\_RESPONSE |  | 24 | 0.37 | 1.05 | 0.400 | 0.535 | 1.000 | 3096 | tags=21%, list=14%, signal=24% |
| 1547 | GO\_VENOUS\_BLOOD\_VESSEL\_DEVELOPMENT |  | 15 | 0.48 | 1.05 | 0.384 | 0.535 | 1.000 | 4493 | tags=60%, list=21%, signal=76% |
| 1548 | GO\_REGULATION\_OF\_INTERLEUKIN\_5\_PRODUCTION |  | 18 | 0.45 | 1.05 | 0.369 | 0.536 | 1.000 | 4414 | tags=50%, list=20%, signal=63% |
| 1549 | GO\_SOMATIC\_STEM\_CELL\_POPULATION\_MAINTENANCE |  | 64 | 0.33 | 1.05 | 0.361 | 0.536 | 1.000 | 2716 | tags=17%, list=12%, signal=20% |
| 1550 | GO\_EMBRYONIC\_SKELETAL\_SYSTEM\_DEVELOPMENT |  | 119 | 0.39 | 1.05 | 0.367 | 0.537 | 1.000 | 2259 | tags=21%, list=10%, signal=23% |
| 1551 | GO\_POSITIVE\_REGULATION\_OF\_TRANSCRIPTION\_FACTOR\_IMPORT\_INTO\_NUCLEUS |  | 51 | 0.41 | 1.05 | 0.393 | 0.537 | 1.000 | 2042 | tags=20%, list=9%, signal=22% |
| 1552 | GO\_REGULATION\_OF\_RESPONSE\_TO\_CYTOKINE\_STIMULUS |  | 135 | 0.33 | 1.05 | 0.383 | 0.537 | 1.000 | 2589 | tags=16%, list=12%, signal=18% |
| 1553 | GO\_INORGANIC\_ANION\_TRANSPORT |  | 124 | 0.32 | 1.05 | 0.342 | 0.538 | 1.000 | 4032 | tags=20%, list=19%, signal=25% |
| 1554 | GO\_REGULATION\_OF\_INNATE\_IMMUNE\_RESPONSE |  | 338 | 0.37 | 1.05 | 0.429 | 0.538 | 1.000 | 3526 | tags=23%, list=16%, signal=27% |
| 1555 | GO\_CEREBRAL\_CORTEX\_DEVELOPMENT |  | 102 | 0.33 | 1.05 | 0.339 | 0.540 | 1.000 | 3655 | tags=28%, list=17%, signal=34% |
| 1556 | GO\_REGULATION\_OF\_ARF\_PROTEIN\_SIGNAL\_TRANSDUCTION |  | 15 | 0.36 | 1.05 | 0.393 | 0.541 | 1.000 | 4784 | tags=33%, list=22%, signal=43% |
| 1557 | GO\_PHASIC\_SMOOTH\_MUSCLE\_CONTRACTION |  | 16 | 0.48 | 1.05 | 0.389 | 0.540 | 1.000 | 1881 | tags=25%, list=9%, signal=27% |
| 1558 | GO\_NADH\_METABOLIC\_PROCESS |  | 34 | 0.38 | 1.05 | 0.374 | 0.540 | 1.000 | 563 | tags=12%, list=3%, signal=12% |
| 1559 | GO\_UNSATURATED\_FATTY\_ACID\_BIOSYNTHETIC\_PROCESS |  | 52 | 0.42 | 1.05 | 0.372 | 0.540 | 1.000 | 1924 | tags=21%, list=9%, signal=23% |
| 1560 | GO\_OVARIAN\_FOLLICLE\_DEVELOPMENT |  | 57 | 0.35 | 1.05 | 0.365 | 0.540 | 1.000 | 2956 | tags=23%, list=14%, signal=26% |
| 1561 | GO\_SCHWANN\_CELL\_DIFFERENTIATION |  | 30 | 0.37 | 1.05 | 0.376 | 0.540 | 1.000 | 3755 | tags=30%, list=17%, signal=36% |
| 1562 | GO\_TONGUE\_DEVELOPMENT |  | 18 | 0.46 | 1.05 | 0.365 | 0.540 | 1.000 | 2956 | tags=28%, list=14%, signal=32% |
| 1563 | GO\_CELLULAR\_RESPONSE\_TO\_EXTRACELLULAR\_STIMULUS |  | 178 | 0.31 | 1.05 | 0.325 | 0.540 | 1.000 | 3290 | tags=23%, list=15%, signal=27% |
| 1564 | GO\_REACTIVE\_NITROGEN\_SPECIES\_METABOLIC\_PROCESS |  | 17 | 0.47 | 1.05 | 0.371 | 0.540 | 1.000 | 1697 | tags=29%, list=8%, signal=32% |
| 1565 | GO\_TISSUE\_HOMEOSTASIS |  | 155 | 0.34 | 1.05 | 0.347 | 0.542 | 1.000 | 3655 | tags=26%, list=17%, signal=31% |
| 1566 | GO\_NEURAL\_CREST\_CELL\_MIGRATION |  | 51 | 0.39 | 1.05 | 0.357 | 0.542 | 1.000 | 3404 | tags=25%, list=16%, signal=30% |
| 1567 | GO\_POSITIVE\_REGULATION\_OF\_SEQUENCE\_SPECIFIC\_DNA\_BINDING\_TRANSCRIPTION\_FACTOR\_ACTIVITY |  | 218 | 0.30 | 1.04 | 0.375 | 0.542 | 1.000 | 3290 | tags=19%, list=15%, signal=22% |
| 1568 | GO\_STRESS\_ACTIVATED\_PROTEIN\_KINASE\_SIGNALING\_CASCADE |  | 100 | 0.31 | 1.04 | 0.338 | 0.542 | 1.000 | 1978 | tags=14%, list=9%, signal=15% |
| 1569 | GO\_REGULATION\_OF\_INSULIN\_RECEPTOR\_SIGNALING\_PATHWAY |  | 41 | 0.34 | 1.04 | 0.343 | 0.543 | 1.000 | 3222 | tags=27%, list=15%, signal=31% |
| 1570 | GO\_POSITIVE\_REGULATION\_OF\_DENDRITE\_DEVELOPMENT |  | 62 | 0.33 | 1.04 | 0.372 | 0.543 | 1.000 | 3351 | tags=23%, list=15%, signal=27% |
| 1571 | GO\_REGULATION\_OF\_INTERLEUKIN\_1\_PRODUCTION |  | 53 | 0.44 | 1.04 | 0.414 | 0.544 | 1.000 | 3241 | tags=34%, list=15%, signal=40% |
| 1572 | GO\_NEGATIVE\_REGULATION\_OF\_LEUKOCYTE\_PROLIFERATION |  | 66 | 0.42 | 1.04 | 0.381 | 0.545 | 1.000 | 3457 | tags=36%, list=16%, signal=43% |
| 1573 | GO\_CELLULAR\_RESPONSE\_TO\_INORGANIC\_SUBSTANCE |  | 145 | 0.30 | 1.04 | 0.343 | 0.545 | 1.000 | 3831 | tags=23%, list=18%, signal=27% |
| 1574 | GO\_REGULATION\_OF\_CARDIAC\_MUSCLE\_CELL\_PROLIFERATION |  | 29 | 0.42 | 1.04 | 0.381 | 0.546 | 1.000 | 2451 | tags=28%, list=11%, signal=31% |
| 1575 | GO\_GLYCOSPHINGOLIPID\_BIOSYNTHETIC\_PROCESS |  | 23 | 0.42 | 1.04 | 0.383 | 0.547 | 1.000 | 3126 | tags=30%, list=14%, signal=36% |
| 1576 | GO\_MORPHOGENESIS\_OF\_AN\_EPITHELIUM |  | 388 | 0.33 | 1.04 | 0.360 | 0.548 | 1.000 | 3433 | tags=23%, list=16%, signal=27% |
| 1577 | GO\_POSITIVE\_REGULATION\_OF\_AMINE\_TRANSPORT |  | 31 | 0.41 | 1.04 | 0.402 | 0.548 | 1.000 | 2386 | tags=19%, list=11%, signal=22% |
| 1578 | GO\_PROTEOGLYCAN\_METABOLIC\_PROCESS |  | 82 | 0.41 | 1.04 | 0.409 | 0.548 | 1.000 | 3566 | tags=32%, list=16%, signal=38% |
| 1579 | GO\_RESPONSE\_TO\_ESTRADIOL |  | 144 | 0.32 | 1.04 | 0.352 | 0.548 | 1.000 | 3109 | tags=24%, list=14%, signal=27% |
| 1580 | GO\_REGULATION\_OF\_MORPHOGENESIS\_OF\_A\_BRANCHING\_STRUCTURE |  | 53 | 0.43 | 1.04 | 0.384 | 0.548 | 1.000 | 1855 | tags=23%, list=9%, signal=25% |
| 1581 | GO\_NEGATIVE\_REGULATION\_OF\_RESPONSE\_TO\_BIOTIC\_STIMULUS |  | 27 | 0.42 | 1.04 | 0.412 | 0.549 | 1.000 | 3814 | tags=37%, list=18%, signal=45% |
| 1582 | GO\_MAMMARY\_GLAND\_EPITHELIAL\_CELL\_DIFFERENTIATION |  | 16 | 0.47 | 1.04 | 0.404 | 0.550 | 1.000 | 1823 | tags=25%, list=8%, signal=27% |
| 1583 | GO\_APPENDAGE\_DEVELOPMENT |  | 161 | 0.36 | 1.04 | 0.388 | 0.550 | 1.000 | 2070 | tags=16%, list=10%, signal=18% |
| 1584 | GO\_REGULATION\_OF\_SYNAPTIC\_TRANSMISSION\_GABAERGIC |  | 28 | 0.33 | 1.04 | 0.412 | 0.550 | 1.000 | 1919 | tags=11%, list=9%, signal=12% |
| 1585 | GO\_LEUKOCYTE\_CHEMOTAXIS |  | 107 | 0.47 | 1.04 | 0.417 | 0.551 | 1.000 | 3084 | tags=35%, list=14%, signal=40% |
| 1586 | GO\_AMINO\_ACID\_IMPORT |  | 15 | 0.42 | 1.04 | 0.412 | 0.552 | 1.000 | 3775 | tags=33%, list=17%, signal=40% |
| 1587 | GO\_CHONDROCYTE\_DEVELOPMENT |  | 21 | 0.53 | 1.04 | 0.452 | 0.552 | 1.000 | 4493 | tags=43%, list=21%, signal=54% |
| 1588 | GO\_NEURAL\_TUBE\_DEVELOPMENT |  | 144 | 0.33 | 1.04 | 0.383 | 0.553 | 1.000 | 3353 | tags=20%, list=15%, signal=24% |
| 1589 | GO\_CELLULAR\_RESPONSE\_TO\_INTERLEUKIN\_1 |  | 77 | 0.41 | 1.04 | 0.409 | 0.553 | 1.000 | 1783 | tags=21%, list=8%, signal=23% |
| 1590 | GO\_HAIR\_CELL\_DIFFERENTIATION |  | 33 | 0.36 | 1.04 | 0.398 | 0.553 | 1.000 | 2058 | tags=12%, list=9%, signal=13% |
| 1591 | GO\_LYMPHOCYTE\_CHEMOTAXIS |  | 32 | 0.56 | 1.04 | 0.454 | 0.553 | 1.000 | 3080 | tags=44%, list=14%, signal=51% |
| 1592 | GO\_HIPPO\_SIGNALING |  | 27 | 0.37 | 1.04 | 0.408 | 0.553 | 1.000 | 220 | tags=7%, list=1%, signal=7% |
| 1593 | GO\_CELL\_SURFACE\_RECEPTOR\_SIGNALING\_PATHWAY\_INVOLVED\_IN\_CELL\_CELL\_SIGNALING |  | 67 | 0.35 | 1.04 | 0.409 | 0.553 | 1.000 | 3913 | tags=21%, list=18%, signal=25% |
| 1594 | GO\_REGULATION\_OF\_PEPTIDASE\_ACTIVITY |  | 363 | 0.30 | 1.04 | 0.353 | 0.553 | 1.000 | 3241 | tags=20%, list=15%, signal=24% |
| 1595 | GO\_REGULATION\_OF\_METANEPHROS\_DEVELOPMENT |  | 23 | 0.42 | 1.03 | 0.395 | 0.554 | 1.000 | 1250 | tags=13%, list=6%, signal=14% |
| 1596 | GO\_EMBRYONIC\_CAMERA\_TYPE\_EYE\_MORPHOGENESIS |  | 24 | 0.42 | 1.03 | 0.400 | 0.554 | 1.000 | 1963 | tags=17%, list=9%, signal=18% |
| 1597 | GO\_RESPONSE\_TO\_DRUG |  | 414 | 0.29 | 1.03 | 0.345 | 0.554 | 1.000 | 2646 | tags=16%, list=12%, signal=18% |
| 1598 | GO\_PROTEIN\_MANNOSYLATION |  | 17 | 0.44 | 1.03 | 0.442 | 0.555 | 1.000 | 2537 | tags=24%, list=12%, signal=27% |
| 1599 | GO\_NEGATIVE\_REGULATION\_OF\_VIRAL\_ENTRY\_INTO\_HOST\_CELL |  | 19 | 0.42 | 1.03 | 0.415 | 0.555 | 1.000 | 4259 | tags=26%, list=20%, signal=33% |
| 1600 | GO\_SMOOTH\_MUSCLE\_TISSUE\_DEVELOPMENT |  | 18 | 0.43 | 1.03 | 0.398 | 0.556 | 1.000 | 3309 | tags=44%, list=15%, signal=52% |
| 1601 | GO\_MONOCARBOXYLIC\_ACID\_METABOLIC\_PROCESS |  | 455 | 0.32 | 1.03 | 0.376 | 0.556 | 1.000 | 2692 | tags=16%, list=12%, signal=18% |
| 1602 | GO\_NEGATIVE\_REGULATION\_OF\_CARBOHYDRATE\_METABOLIC\_PROCESS |  | 45 | 0.37 | 1.03 | 0.386 | 0.556 | 1.000 | 1801 | tags=18%, list=8%, signal=19% |
| 1603 | GO\_WALKING\_BEHAVIOR |  | 30 | 0.34 | 1.03 | 0.391 | 0.556 | 1.000 | 3139 | tags=20%, list=14%, signal=23% |
| 1604 | GO\_ENERGY\_DERIVATION\_BY\_OXIDATION\_OF\_ORGANIC\_COMPOUNDS |  | 203 | 0.26 | 1.03 | 0.385 | 0.556 | 1.000 | 4996 | tags=25%, list=23%, signal=32% |
| 1605 | GO\_HEXOSE\_METABOLIC\_PROCESS |  | 147 | 0.31 | 1.03 | 0.362 | 0.556 | 1.000 | 2880 | tags=18%, list=13%, signal=21% |
| 1606 | GO\_ACTIVATION\_OF\_INNATE\_IMMUNE\_RESPONSE |  | 194 | 0.37 | 1.03 | 0.443 | 0.558 | 1.000 | 2709 | tags=20%, list=12%, signal=22% |
| 1607 | GO\_POSITIVE\_REGULATION\_OF\_REACTIVE\_OXYGEN\_SPECIES\_METABOLIC\_PROCESS |  | 83 | 0.37 | 1.03 | 0.385 | 0.558 | 1.000 | 3170 | tags=31%, list=15%, signal=37% |
| 1608 | GO\_CELLULAR\_RESPONSE\_TO\_MECHANICAL\_STIMULUS |  | 78 | 0.35 | 1.03 | 0.396 | 0.558 | 1.000 | 3001 | tags=23%, list=14%, signal=27% |
| 1609 | GO\_PEPTIDYL\_ASPARAGINE\_MODIFICATION |  | 38 | 0.38 | 1.03 | 0.431 | 0.558 | 1.000 | 4738 | tags=37%, list=22%, signal=47% |
| 1610 | GO\_NEGATIVE\_REGULATION\_OF\_MULTI\_ORGANISM\_PROCESS |  | 137 | 0.37 | 1.03 | 0.435 | 0.558 | 1.000 | 3084 | tags=20%, list=14%, signal=23% |
| 1611 | GO\_INTERFERON\_GAMMA\_MEDIATED\_SIGNALING\_PATHWAY |  | 65 | 0.49 | 1.03 | 0.458 | 0.559 | 1.000 | 4877 | tags=48%, list=22%, signal=61% |
| 1612 | GO\_REGULATION\_OF\_KIDNEY\_DEVELOPMENT |  | 55 | 0.38 | 1.03 | 0.385 | 0.561 | 1.000 | 3267 | tags=24%, list=15%, signal=28% |
| 1613 | GO\_GANGLIOSIDE\_METABOLIC\_PROCESS |  | 25 | 0.38 | 1.03 | 0.394 | 0.562 | 1.000 | 4738 | tags=44%, list=22%, signal=56% |
| 1614 | GO\_REGULATION\_OF\_SPROUTING\_ANGIOGENESIS |  | 27 | 0.40 | 1.03 | 0.407 | 0.562 | 1.000 | 2946 | tags=26%, list=14%, signal=30% |
| 1615 | GO\_ECTODERMAL\_PLACODE\_DEVELOPMENT |  | 15 | 0.46 | 1.03 | 0.450 | 0.562 | 1.000 | 4087 | tags=40%, list=19%, signal=49% |
| 1616 | GO\_POLYSACCHARIDE\_BIOSYNTHETIC\_PROCESS |  | 40 | 0.37 | 1.03 | 0.424 | 0.562 | 1.000 | 5314 | tags=45%, list=24%, signal=59% |
| 1617 | GO\_CELL\_CYCLE\_ARREST |  | 142 | 0.29 | 1.03 | 0.405 | 0.562 | 1.000 | 3583 | tags=24%, list=16%, signal=28% |
| 1618 | GO\_ESTABLISHMENT\_OF\_CELL\_POLARITY |  | 85 | 0.30 | 1.03 | 0.406 | 0.562 | 1.000 | 3056 | tags=21%, list=14%, signal=25% |
| 1619 | GO\_POSITIVE\_REGULATION\_OF\_INTERFERON\_ALPHA\_PRODUCTION |  | 16 | 0.55 | 1.03 | 0.494 | 0.562 | 1.000 | 1739 | tags=19%, list=8%, signal=20% |
| 1620 | GO\_POSITIVE\_REGULATION\_OF\_NEUTROPHIL\_MIGRATION |  | 26 | 0.49 | 1.03 | 0.433 | 0.563 | 1.000 | 3331 | tags=50%, list=15%, signal=59% |
| 1621 | GO\_REGULATION\_OF\_PROTEIN\_EXIT\_FROM\_ENDOPLASMIC\_RETICULUM |  | 18 | 0.43 | 1.03 | 0.454 | 0.563 | 1.000 | 4242 | tags=33%, list=20%, signal=41% |
| 1622 | GO\_MATING\_BEHAVIOR |  | 22 | 0.37 | 1.02 | 0.423 | 0.564 | 1.000 | 6200 | tags=41%, list=29%, signal=57% |
| 1623 | GO\_CALCIUM\_ION\_REGULATED\_EXOCYTOSIS |  | 72 | 0.34 | 1.02 | 0.404 | 0.564 | 1.000 | 2250 | tags=17%, list=10%, signal=19% |
| 1624 | GO\_POSITIVE\_REGULATION\_OF\_GROWTH |  | 226 | 0.28 | 1.02 | 0.377 | 0.564 | 1.000 | 3024 | tags=18%, list=14%, signal=21% |
| 1625 | GO\_CELL\_JUNCTION\_ORGANIZATION |  | 175 | 0.32 | 1.02 | 0.406 | 0.563 | 1.000 | 1719 | tags=14%, list=8%, signal=15% |
| 1626 | GO\_CELLULAR\_LIPID\_CATABOLIC\_PROCESS |  | 143 | 0.34 | 1.02 | 0.398 | 0.563 | 1.000 | 4883 | tags=35%, list=22%, signal=45% |
| 1627 | GO\_RESPONSE\_TO\_REACTIVE\_OXYGEN\_SPECIES |  | 186 | 0.32 | 1.02 | 0.380 | 0.563 | 1.000 | 3305 | tags=24%, list=15%, signal=28% |
| 1628 | GO\_PROTEOGLYCAN\_BIOSYNTHETIC\_PROCESS |  | 57 | 0.42 | 1.02 | 0.432 | 0.565 | 1.000 | 1513 | tags=16%, list=7%, signal=17% |
| 1629 | GO\_ORGANOPHOSPHATE\_CATABOLIC\_PROCESS |  | 107 | 0.29 | 1.02 | 0.397 | 0.566 | 1.000 | 4414 | tags=28%, list=20%, signal=35% |
| 1630 | GO\_REGULATION\_OF\_PEPTIDE\_SECRETION |  | 200 | 0.27 | 1.02 | 0.364 | 0.568 | 1.000 | 3785 | tags=21%, list=17%, signal=25% |
| 1631 | GO\_REGULATION\_OF\_SMOOTH\_MUSCLE\_CONTRACTION |  | 59 | 0.35 | 1.02 | 0.389 | 0.567 | 1.000 | 2785 | tags=19%, list=13%, signal=21% |
| 1632 | GO\_NEURAL\_PRECURSOR\_CELL\_PROLIFERATION |  | 69 | 0.35 | 1.02 | 0.412 | 0.568 | 1.000 | 2926 | tags=22%, list=13%, signal=25% |
| 1633 | GO\_SPHINGOLIPID\_METABOLIC\_PROCESS |  | 124 | 0.32 | 1.02 | 0.417 | 0.567 | 1.000 | 4428 | tags=33%, list=20%, signal=41% |
| 1634 | GO\_MODULATION\_OF\_EXCITATORY\_POSTSYNAPTIC\_POTENTIAL |  | 30 | 0.37 | 1.02 | 0.424 | 0.567 | 1.000 | 1435 | tags=13%, list=7%, signal=14% |
| 1635 | GO\_MESODERM\_DEVELOPMENT |  | 109 | 0.32 | 1.02 | 0.383 | 0.568 | 1.000 | 3049 | tags=21%, list=14%, signal=24% |
| 1636 | GO\_ARACHIDONIC\_ACID\_METABOLIC\_PROCESS |  | 49 | 0.47 | 1.02 | 0.447 | 0.568 | 1.000 | 2177 | tags=20%, list=10%, signal=23% |
| 1637 | GO\_NEGATIVE\_REGULATION\_OF\_INTRINSIC\_APOPTOTIC\_SIGNALING\_PATHWAY |  | 79 | 0.29 | 1.02 | 0.405 | 0.568 | 1.000 | 2478 | tags=14%, list=11%, signal=16% |
| 1638 | GO\_ADRENAL\_GLAND\_DEVELOPMENT |  | 23 | 0.42 | 1.02 | 0.414 | 0.567 | 1.000 | 2787 | tags=22%, list=13%, signal=25% |
| 1639 | GO\_DRUG\_TRANSMEMBRANE\_TRANSPORT |  | 19 | 0.48 | 1.02 | 0.426 | 0.567 | 1.000 | 1735 | tags=16%, list=8%, signal=17% |
| 1640 | GO\_GLYCOSYLATION |  | 246 | 0.31 | 1.02 | 0.404 | 0.568 | 1.000 | 4045 | tags=23%, list=19%, signal=28% |
| 1641 | GO\_REGULATION\_OF\_STRIATED\_MUSCLE\_CELL\_APOPTOTIC\_PROCESS |  | 21 | 0.39 | 1.02 | 0.404 | 0.567 | 1.000 | 3619 | tags=33%, list=17%, signal=40% |
| 1642 | GO\_REGULATION\_OF\_INTERLEUKIN\_1\_BETA\_PRODUCTION |  | 44 | 0.43 | 1.02 | 0.435 | 0.568 | 1.000 | 3241 | tags=30%, list=15%, signal=35% |
| 1643 | GO\_SYNCYTIUM\_FORMATION |  | 25 | 0.40 | 1.02 | 0.425 | 0.568 | 1.000 | 1418 | tags=16%, list=7%, signal=17% |
| 1644 | GO\_LIPID\_HOMEOSTASIS |  | 103 | 0.31 | 1.02 | 0.398 | 0.568 | 1.000 | 3222 | tags=19%, list=15%, signal=23% |
| 1645 | GO\_RETROGRADE\_TRANSPORT\_ENDOSOME\_TO\_GOLGI |  | 64 | 0.29 | 1.02 | 0.421 | 0.568 | 1.000 | 3745 | tags=27%, list=17%, signal=32% |
| 1646 | GO\_PHOSPHOLIPID\_TRANSPORT |  | 53 | 0.34 | 1.02 | 0.429 | 0.569 | 1.000 | 3751 | tags=26%, list=17%, signal=32% |
| 1647 | GO\_NEGATIVE\_REGULATION\_OF\_BEHAVIOR |  | 17 | 0.34 | 1.02 | 0.437 | 0.570 | 1.000 | 2746 | tags=18%, list=13%, signal=20% |
| 1648 | GO\_POSITIVE\_REGULATION\_OF\_CELL\_MATRIX\_ADHESION |  | 40 | 0.36 | 1.02 | 0.452 | 0.570 | 1.000 | 3408 | tags=30%, list=16%, signal=36% |
| 1649 | GO\_POSITIVE\_REGULATION\_OF\_CARDIAC\_MUSCLE\_CELL\_PROLIFERATION |  | 19 | 0.45 | 1.02 | 0.438 | 0.571 | 1.000 | 2451 | tags=37%, list=11%, signal=41% |
| 1650 | GO\_POSITIVE\_REGULATION\_OF\_PROTEIN\_LOCALIZATION\_TO\_NUCLEUS |  | 122 | 0.33 | 1.01 | 0.420 | 0.574 | 1.000 | 1739 | tags=14%, list=8%, signal=15% |
| 1651 | GO\_POSITIVE\_REGULATION\_OF\_LYMPHOCYTE\_MEDIATED\_IMMUNITY |  | 66 | 0.42 | 1.01 | 0.449 | 0.575 | 1.000 | 3241 | tags=32%, list=15%, signal=37% |
| 1652 | GO\_INTRACELLULAR\_STEROID\_HORMONE\_RECEPTOR\_SIGNALING\_PATHWAY |  | 67 | 0.28 | 1.01 | 0.419 | 0.576 | 1.000 | 2373 | tags=12%, list=11%, signal=13% |
| 1653 | GO\_CHEMOKINE\_MEDIATED\_SIGNALING\_PATHWAY |  | 62 | 0.51 | 1.01 | 0.478 | 0.576 | 1.000 | 3170 | tags=37%, list=15%, signal=43% |
| 1654 | GO\_LONG\_CHAIN\_FATTY\_ACID\_METABOLIC\_PROCESS |  | 84 | 0.41 | 1.01 | 0.422 | 0.576 | 1.000 | 2360 | tags=18%, list=11%, signal=20% |
| 1655 | GO\_CELLULAR\_RESPONSE\_TO\_STARVATION |  | 108 | 0.30 | 1.01 | 0.395 | 0.576 | 1.000 | 3222 | tags=21%, list=15%, signal=25% |
| 1656 | GO\_REGULATION\_OF\_T\_CELL\_RECEPTOR\_SIGNALING\_PATHWAY |  | 27 | 0.45 | 1.01 | 0.472 | 0.576 | 1.000 | 2734 | tags=30%, list=13%, signal=34% |
| 1657 | GO\_HINDBRAIN\_DEVELOPMENT |  | 134 | 0.30 | 1.01 | 0.425 | 0.577 | 1.000 | 3582 | tags=19%, list=16%, signal=22% |
| 1658 | GO\_POSITIVE\_REGULATION\_OF\_CELL\_JUNCTION\_ASSEMBLY |  | 24 | 0.39 | 1.01 | 0.433 | 0.577 | 1.000 | 3757 | tags=38%, list=17%, signal=45% |
| 1659 | GO\_POSITIVE\_REGULATION\_OF\_LIPID\_BIOSYNTHETIC\_PROCESS |  | 61 | 0.34 | 1.01 | 0.412 | 0.577 | 1.000 | 3084 | tags=23%, list=14%, signal=27% |
| 1660 | GO\_PROTEIN\_ADP\_RIBOSYLATION |  | 20 | 0.33 | 1.01 | 0.436 | 0.578 | 1.000 | 5037 | tags=40%, list=23%, signal=52% |
| 1661 | GO\_RESPONSE\_TO\_FLUID\_SHEAR\_STRESS |  | 33 | 0.41 | 1.01 | 0.453 | 0.578 | 1.000 | 3170 | tags=30%, list=15%, signal=35% |
| 1662 | GO\_ORGANELLE\_ASSEMBLY |  | 447 | 0.28 | 1.01 | 0.422 | 0.578 | 1.000 | 2908 | tags=15%, list=13%, signal=17% |
| 1663 | GO\_POSITIVE\_REGULATION\_OF\_MESENCHYMAL\_CELL\_PROLIFERATION |  | 27 | 0.44 | 1.01 | 0.450 | 0.578 | 1.000 | 1742 | tags=30%, list=8%, signal=32% |
| 1664 | GO\_PROTEIN\_TARGETING\_TO\_MEMBRANE |  | 138 | 0.21 | 1.01 | 0.439 | 0.579 | 1.000 | 3003 | tags=9%, list=14%, signal=10% |
| 1665 | GO\_DENDRITIC\_CELL\_DIFFERENTIATION |  | 32 | 0.49 | 1.01 | 0.471 | 0.579 | 1.000 | 3406 | tags=50%, list=16%, signal=59% |
| 1666 | GO\_DORSAL\_SPINAL\_CORD\_DEVELOPMENT |  | 17 | 0.40 | 1.01 | 0.481 | 0.578 | 1.000 | 4501 | tags=35%, list=21%, signal=44% |
| 1667 | GO\_RESPONSE\_TO\_CALCIUM\_ION |  | 109 | 0.30 | 1.01 | 0.409 | 0.579 | 1.000 | 3831 | tags=22%, list=18%, signal=27% |
| 1668 | GO\_REGULATION\_OF\_ORGANIC\_ACID\_TRANSPORT |  | 46 | 0.35 | 1.01 | 0.444 | 0.578 | 1.000 | 2376 | tags=20%, list=11%, signal=22% |
| 1669 | GO\_TUBE\_FORMATION |  | 129 | 0.32 | 1.01 | 0.418 | 0.578 | 1.000 | 3351 | tags=22%, list=15%, signal=26% |
| 1670 | GO\_LEUKOTRIENE\_METABOLIC\_PROCESS |  | 30 | 0.47 | 1.01 | 0.450 | 0.579 | 1.000 | 3831 | tags=37%, list=18%, signal=44% |
| 1671 | GO\_FORMATION\_OF\_PRIMARY\_GERM\_LAYER |  | 105 | 0.35 | 1.01 | 0.452 | 0.580 | 1.000 | 3049 | tags=22%, list=14%, signal=25% |
| 1672 | GO\_POSITIVE\_REGULATION\_OF\_LEUKOCYTE\_MEDIATED\_IMMUNITY |  | 82 | 0.41 | 1.01 | 0.453 | 0.581 | 1.000 | 3526 | tags=34%, list=16%, signal=41% |
| 1673 | GO\_PATTERN\_SPECIFICATION\_PROCESS |  | 395 | 0.31 | 1.01 | 0.437 | 0.583 | 1.000 | 3353 | tags=20%, list=15%, signal=24% |
| 1674 | GO\_NEGATIVE\_REGULATION\_OF\_CYCLIN\_DEPENDENT\_PROTEIN\_KINASE\_ACTIVITY |  | 29 | 0.32 | 1.01 | 0.455 | 0.583 | 1.000 | 3290 | tags=28%, list=15%, signal=32% |
| 1675 | GO\_REGULATION\_OF\_CIRCADIAN\_SLEEP\_WAKE\_CYCLE |  | 24 | 0.37 | 1.01 | 0.440 | 0.583 | 1.000 | 2646 | tags=17%, list=12%, signal=19% |
| 1676 | GO\_COCHLEA\_MORPHOGENESIS |  | 21 | 0.43 | 1.01 | 0.446 | 0.583 | 1.000 | 1817 | tags=24%, list=8%, signal=26% |
| 1677 | GO\_PHOSPHOLIPID\_BIOSYNTHETIC\_PROCESS |  | 224 | 0.27 | 1.01 | 0.430 | 0.583 | 1.000 | 4428 | tags=23%, list=20%, signal=28% |
| 1678 | GO\_POSITIVE\_REGULATION\_OF\_INTERFERON\_BETA\_PRODUCTION |  | 30 | 0.44 | 1.01 | 0.506 | 0.583 | 1.000 | 1739 | tags=13%, list=8%, signal=14% |
| 1679 | GO\_ACTIVATION\_OF\_MAPK\_ACTIVITY |  | 130 | 0.31 | 1.01 | 0.404 | 0.583 | 1.000 | 2787 | tags=21%, list=13%, signal=24% |
| 1680 | GO\_MULTICELLULAR\_ORGANISMAL\_WATER\_HOMEOSTASIS |  | 55 | 0.44 | 1.01 | 0.468 | 0.583 | 1.000 | 2450 | tags=25%, list=11%, signal=29% |
| 1681 | GO\_ENDODERM\_DEVELOPMENT |  | 68 | 0.37 | 1.00 | 0.455 | 0.585 | 1.000 | 3310 | tags=24%, list=15%, signal=28% |
| 1682 | GO\_REGULATION\_OF\_ENDOTHELIAL\_CELL\_DIFFERENTIATION |  | 27 | 0.40 | 1.00 | 0.440 | 0.585 | 1.000 | 2956 | tags=30%, list=14%, signal=34% |
| 1683 | GO\_DISRUPTION\_OF\_CELLS\_OF\_OTHER\_ORGANISM |  | 24 | 0.44 | 1.00 | 0.484 | 0.584 | 1.000 | 1444 | tags=13%, list=7%, signal=13% |
| 1684 | GO\_POSITIVE\_REGULATION\_OF\_REPRODUCTIVE\_PROCESS |  | 51 | 0.35 | 1.00 | 0.431 | 0.585 | 1.000 | 3584 | tags=25%, list=16%, signal=30% |
| 1685 | GO\_EMBRYONIC\_SKELETAL\_SYSTEM\_MORPHOGENESIS |  | 92 | 0.37 | 1.00 | 0.449 | 0.586 | 1.000 | 3014 | tags=25%, list=14%, signal=29% |
| 1686 | GO\_REGULATION\_OF\_TRANSCRIPTION\_INVOLVED\_IN\_CELL\_FATE\_COMMITMENT |  | 19 | 0.45 | 1.00 | 0.433 | 0.587 | 1.000 | 4552 | tags=42%, list=21%, signal=53% |
| 1687 | GO\_NEGATIVE\_REGULATION\_OF\_NEURON\_APOPTOTIC\_PROCESS |  | 129 | 0.34 | 1.00 | 0.455 | 0.586 | 1.000 | 3170 | tags=24%, list=15%, signal=28% |
| 1688 | GO\_NEGATIVE\_REGULATION\_OF\_ORGANELLE\_ASSEMBLY |  | 22 | 0.37 | 1.00 | 0.424 | 0.586 | 1.000 | 3345 | tags=27%, list=15%, signal=32% |
| 1689 | GO\_POSITIVE\_REGULATION\_OF\_AXON\_EXTENSION |  | 35 | 0.37 | 1.00 | 0.448 | 0.587 | 1.000 | 4187 | tags=40%, list=19%, signal=49% |
| 1690 | GO\_PURINE\_CONTAINING\_COMPOUND\_BIOSYNTHETIC\_PROCESS |  | 127 | 0.28 | 1.00 | 0.422 | 0.587 | 1.000 | 4046 | tags=23%, list=19%, signal=28% |
| 1691 | GO\_REGULATION\_OF\_PROTEIN\_AUTOPHOSPHORYLATION |  | 34 | 0.38 | 1.00 | 0.450 | 0.587 | 1.000 | 2102 | tags=18%, list=10%, signal=20% |
| 1692 | GO\_PRESYNAPTIC\_PROCESS\_INVOLVED\_IN\_SYNAPTIC\_TRANSMISSION |  | 107 | 0.32 | 1.00 | 0.421 | 0.587 | 1.000 | 2348 | tags=15%, list=11%, signal=17% |
| 1693 | GO\_POSITIVE\_REGULATION\_OF\_SYNAPTIC\_TRANSMISSION |  | 108 | 0.31 | 1.00 | 0.447 | 0.588 | 1.000 | 3288 | tags=22%, list=15%, signal=26% |
| 1694 | GO\_REGULATION\_OF\_BLOOD\_VESSEL\_ENDOTHELIAL\_CELL\_MIGRATION |  | 50 | 0.35 | 1.00 | 0.446 | 0.588 | 1.000 | 1999 | tags=20%, list=9%, signal=22% |
| 1695 | GO\_APOPTOTIC\_PROCESS\_INVOLVED\_IN\_MORPHOGENESIS |  | 16 | 0.42 | 1.00 | 0.457 | 0.588 | 1.000 | 3058 | tags=31%, list=14%, signal=36% |
| 1696 | GO\_REGULATION\_OF\_POSITIVE\_CHEMOTAXIS |  | 22 | 0.44 | 1.00 | 0.467 | 0.588 | 1.000 | 808 | tags=18%, list=4%, signal=19% |
| 1697 | GO\_ACTIVIN\_RECEPTOR\_SIGNALING\_PATHWAY |  | 21 | 0.39 | 1.00 | 0.470 | 0.588 | 1.000 | 2733 | tags=24%, list=13%, signal=27% |
| 1698 | GO\_RESPONSE\_TO\_LIPOPROTEIN\_PARTICLE |  | 20 | 0.42 | 1.00 | 0.467 | 0.590 | 1.000 | 3526 | tags=35%, list=16%, signal=42% |
| 1699 | GO\_PATTERNING\_OF\_BLOOD\_VESSELS |  | 32 | 0.38 | 1.00 | 0.465 | 0.590 | 1.000 | 3221 | tags=31%, list=15%, signal=37% |
| 1700 | GO\_ADIPOSE\_TISSUE\_DEVELOPMENT |  | 31 | 0.40 | 1.00 | 0.464 | 0.590 | 1.000 | 3826 | tags=39%, list=18%, signal=47% |
| 1701 | GO\_REGULATION\_OF\_CELL\_MATURATION |  | 17 | 0.42 | 1.00 | 0.456 | 0.590 | 1.000 | 2089 | tags=29%, list=10%, signal=33% |
| 1702 | GO\_NEUROTRANSMITTER\_TRANSPORT |  | 145 | 0.30 | 1.00 | 0.439 | 0.590 | 1.000 | 3139 | tags=17%, list=14%, signal=19% |
| 1703 | GO\_REGULATION\_OF\_PROTEIN\_HOMODIMERIZATION\_ACTIVITY |  | 22 | 0.38 | 1.00 | 0.441 | 0.590 | 1.000 | 2680 | tags=27%, list=12%, signal=31% |
| 1704 | GO\_AUTONOMIC\_NERVOUS\_SYSTEM\_DEVELOPMENT |  | 41 | 0.40 | 1.00 | 0.476 | 0.591 | 1.000 | 1829 | tags=17%, list=8%, signal=19% |
| 1705 | GO\_POSITIVE\_REGULATION\_OF\_ADHERENS\_JUNCTION\_ORGANIZATION |  | 21 | 0.38 | 1.00 | 0.443 | 0.591 | 1.000 | 3757 | tags=43%, list=17%, signal=52% |
| 1706 | GO\_NEGATIVE\_REGULATION\_OF\_JNK\_CASCADE |  | 32 | 0.37 | 1.00 | 0.475 | 0.591 | 1.000 | 2386 | tags=19%, list=11%, signal=21% |
| 1707 | GO\_LIPID\_CATABOLIC\_PROCESS |  | 228 | 0.30 | 1.00 | 0.444 | 0.593 | 1.000 | 4935 | tags=33%, list=23%, signal=42% |
| 1708 | GO\_MECHANORECEPTOR\_DIFFERENTIATION |  | 46 | 0.34 | 1.00 | 0.448 | 0.593 | 1.000 | 2956 | tags=17%, list=14%, signal=20% |
| 1709 | GO\_BILE\_ACID\_BIOSYNTHETIC\_PROCESS |  | 20 | 0.42 | 1.00 | 0.421 | 0.593 | 1.000 | 1518 | tags=20%, list=7%, signal=21% |
| 1710 | GO\_POSITIVE\_REGULATION\_OF\_LEUKOCYTE\_APOPTOTIC\_PROCESS |  | 25 | 0.43 | 1.00 | 0.473 | 0.593 | 1.000 | 3222 | tags=28%, list=15%, signal=33% |
| 1711 | GO\_EMBRYONIC\_HEART\_TUBE\_DEVELOPMENT |  | 68 | 0.32 | 0.99 | 0.463 | 0.593 | 1.000 | 3058 | tags=18%, list=14%, signal=20% |
| 1712 | GO\_CELL\_JUNCTION\_ASSEMBLY |  | 125 | 0.31 | 0.99 | 0.466 | 0.594 | 1.000 | 1574 | tags=14%, list=7%, signal=15% |
| 1713 | GO\_BETA\_CATENIN\_DESTRUCTION\_COMPLEX\_DISASSEMBLY |  | 20 | 0.37 | 0.99 | 0.475 | 0.596 | 1.000 | 2851 | tags=20%, list=13%, signal=23% |
| 1714 | GO\_NEGATIVE\_REGULATION\_OF\_STEM\_CELL\_DIFFERENTIATION |  | 41 | 0.33 | 0.99 | 0.458 | 0.600 | 1.000 | 664 | tags=7%, list=3%, signal=8% |
| 1715 | GO\_DEVELOPMENT\_OF\_PRIMARY\_SEXUAL\_CHARACTERISTICS |  | 202 | 0.29 | 0.99 | 0.462 | 0.600 | 1.000 | 2800 | tags=17%, list=13%, signal=19% |
| 1716 | GO\_REGULATION\_OF\_PROTEIN\_TYROSINE\_KINASE\_ACTIVITY |  | 57 | 0.32 | 0.99 | 0.466 | 0.600 | 1.000 | 3138 | tags=23%, list=14%, signal=27% |
| 1717 | GO\_REGULATION\_OF\_DIGESTIVE\_SYSTEM\_PROCESS |  | 34 | 0.31 | 0.99 | 0.451 | 0.600 | 1.000 | 1410 | tags=9%, list=6%, signal=9% |
| 1718 | GO\_FATTY\_ACID\_DERIVATIVE\_METABOLIC\_PROCESS |  | 89 | 0.42 | 0.99 | 0.476 | 0.600 | 1.000 | 4408 | tags=35%, list=20%, signal=44% |
| 1719 | GO\_POSITIVE\_REGULATION\_OF\_CELLULAR\_PROTEIN\_LOCALIZATION |  | 327 | 0.26 | 0.99 | 0.484 | 0.601 | 1.000 | 3153 | tags=18%, list=14%, signal=21% |
| 1720 | GO\_MONOCARBOXYLIC\_ACID\_TRANSPORT |  | 111 | 0.32 | 0.99 | 0.465 | 0.600 | 1.000 | 2360 | tags=17%, list=11%, signal=19% |
| 1721 | GO\_REGULATION\_OF\_SYNAPSE\_ASSEMBLY |  | 68 | 0.38 | 0.99 | 0.463 | 0.600 | 1.000 | 2187 | tags=15%, list=10%, signal=16% |
| 1722 | GO\_PROSTANOID\_BIOSYNTHETIC\_PROCESS |  | 18 | 0.45 | 0.99 | 0.455 | 0.601 | 1.000 | 1913 | tags=28%, list=9%, signal=30% |
| 1723 | GO\_POSTSYNAPTIC\_MEMBRANE\_ORGANIZATION |  | 25 | 0.41 | 0.99 | 0.470 | 0.603 | 1.000 | 2201 | tags=24%, list=10%, signal=27% |
| 1724 | GO\_INTRACELLULAR\_RECEPTOR\_SIGNALING\_PATHWAY |  | 159 | 0.26 | 0.99 | 0.492 | 0.603 | 1.000 | 2616 | tags=12%, list=12%, signal=13% |
| 1725 | GO\_MAMMARY\_GLAND\_DEVELOPMENT |  | 109 | 0.32 | 0.99 | 0.475 | 0.603 | 1.000 | 3042 | tags=26%, list=14%, signal=30% |
| 1726 | GO\_RESPONSE\_TO\_FOLIC\_ACID |  | 15 | 0.43 | 0.99 | 0.503 | 0.604 | 1.000 | 4217 | tags=40%, list=19%, signal=50% |
| 1727 | GO\_REGULATION\_OF\_FATTY\_ACID\_BIOSYNTHETIC\_PROCESS |  | 35 | 0.35 | 0.99 | 0.461 | 0.604 | 1.000 | 2376 | tags=17%, list=11%, signal=19% |
| 1728 | GO\_REGULATION\_OF\_ORGANELLE\_ASSEMBLY |  | 140 | 0.25 | 0.99 | 0.471 | 0.604 | 1.000 | 4432 | tags=27%, list=20%, signal=34% |
| 1729 | GO\_DEFENSE\_RESPONSE\_TO\_GRAM\_NEGATIVE\_BACTERIUM |  | 42 | 0.36 | 0.99 | 0.452 | 0.604 | 1.000 | 1625 | tags=14%, list=7%, signal=15% |
| 1730 | GO\_WATER\_TRANSPORT |  | 18 | 0.41 | 0.99 | 0.485 | 0.604 | 1.000 | 3032 | tags=22%, list=14%, signal=26% |
| 1731 | GO\_ACUTE\_INFLAMMATORY\_RESPONSE |  | 71 | 0.35 | 0.99 | 0.479 | 0.604 | 1.000 | 1690 | tags=14%, list=8%, signal=15% |
| 1732 | GO\_NEGATIVE\_REGULATION\_OF\_ANOIKIS |  | 17 | 0.41 | 0.99 | 0.480 | 0.604 | 1.000 | 2669 | tags=18%, list=12%, signal=20% |
| 1733 | GO\_RESPONSE\_TO\_ANTIBIOTIC |  | 45 | 0.34 | 0.99 | 0.478 | 0.604 | 1.000 | 3080 | tags=27%, list=14%, signal=31% |
| 1734 | GO\_REGULATION\_OF\_MULTI\_ORGANISM\_PROCESS |  | 434 | 0.28 | 0.99 | 0.467 | 0.604 | 1.000 | 3097 | tags=16%, list=14%, signal=18% |
| 1735 | GO\_REGULATION\_OF\_INTERLEUKIN\_8\_SECRETION |  | 18 | 0.44 | 0.99 | 0.487 | 0.604 | 1.000 | 3032 | tags=28%, list=14%, signal=32% |
| 1736 | GO\_POSITIVE\_REGULATION\_OF\_PHOSPHATASE\_ACTIVITY |  | 26 | 0.41 | 0.99 | 0.490 | 0.604 | 1.000 | 3478 | tags=31%, list=16%, signal=37% |
| 1737 | GO\_CELLULAR\_RESPONSE\_TO\_BIOTIC\_STIMULUS |  | 153 | 0.37 | 0.98 | 0.479 | 0.605 | 1.000 | 3308 | tags=30%, list=15%, signal=35% |
| 1738 | GO\_EYE\_MORPHOGENESIS |  | 131 | 0.32 | 0.98 | 0.463 | 0.608 | 1.000 | 2965 | tags=18%, list=14%, signal=21% |
| 1739 | GO\_PROSTANOID\_METABOLIC\_PROCESS |  | 25 | 0.41 | 0.98 | 0.483 | 0.607 | 1.000 | 1913 | tags=24%, list=9%, signal=26% |
| 1740 | GO\_POSITIVE\_REGULATION\_OF\_INTERLEUKIN\_1\_SECRETION |  | 22 | 0.48 | 0.98 | 0.487 | 0.607 | 1.000 | 1556 | tags=18%, list=7%, signal=20% |
| 1741 | GO\_REGULATION\_OF\_HEAT\_GENERATION |  | 15 | 0.48 | 0.98 | 0.482 | 0.608 | 1.000 | 1940 | tags=40%, list=9%, signal=44% |
| 1742 | GO\_I\_KAPPAB\_KINASE\_NF\_KAPPAB\_SIGNALING |  | 66 | 0.35 | 0.98 | 0.490 | 0.608 | 1.000 | 1739 | tags=12%, list=8%, signal=13% |
| 1743 | GO\_REGULATION\_OF\_PROTEIN\_ACETYLATION |  | 62 | 0.29 | 0.98 | 0.480 | 0.607 | 1.000 | 4551 | tags=34%, list=21%, signal=43% |
| 1744 | GO\_ESTABLISHMENT\_OR\_MAINTENANCE\_OF\_CELL\_POLARITY |  | 134 | 0.28 | 0.98 | 0.511 | 0.609 | 1.000 | 3056 | tags=19%, list=14%, signal=22% |
| 1745 | GO\_CAMERA\_TYPE\_EYE\_MORPHOGENESIS |  | 97 | 0.33 | 0.98 | 0.476 | 0.609 | 1.000 | 2965 | tags=21%, list=14%, signal=24% |
| 1746 | GO\_DEFENSE\_RESPONSE\_TO\_GRAM\_POSITIVE\_BACTERIUM |  | 65 | 0.37 | 0.98 | 0.488 | 0.611 | 1.000 | 2749 | tags=20%, list=13%, signal=23% |
| 1747 | GO\_NEGATIVE\_REGULATION\_OF\_MYOBLAST\_DIFFERENTIATION |  | 23 | 0.41 | 0.98 | 0.482 | 0.611 | 1.000 | 2956 | tags=26%, list=14%, signal=30% |
| 1748 | GO\_MYELOID\_LEUKOCYTE\_MIGRATION |  | 90 | 0.43 | 0.98 | 0.514 | 0.610 | 1.000 | 1428 | tags=20%, list=7%, signal=21% |
| 1749 | GO\_POSITIVE\_REGULATION\_OF\_LIPID\_TRANSPORT |  | 49 | 0.34 | 0.98 | 0.480 | 0.610 | 1.000 | 3222 | tags=27%, list=15%, signal=31% |
| 1750 | GO\_POSITIVE\_REGULATION\_OF\_MAST\_CELL\_ACTIVATION |  | 15 | 0.46 | 0.98 | 0.508 | 0.611 | 1.000 | 2428 | tags=33%, list=11%, signal=37% |
| 1751 | GO\_NEURONAL\_STEM\_CELL\_POPULATION\_MAINTENANCE |  | 19 | 0.41 | 0.98 | 0.479 | 0.612 | 1.000 | 3967 | tags=32%, list=18%, signal=39% |
| 1752 | GO\_POSITIVE\_REGULATION\_OF\_AXONOGENESIS |  | 66 | 0.33 | 0.98 | 0.465 | 0.612 | 1.000 | 3256 | tags=27%, list=15%, signal=32% |
| 1753 | GO\_CELLULAR\_RESPONSE\_TO\_INTERFERON\_GAMMA |  | 109 | 0.45 | 0.98 | 0.514 | 0.614 | 1.000 | 4778 | tags=44%, list=22%, signal=56% |
| 1754 | GO\_RETINA\_MORPHOGENESIS\_IN\_CAMERA\_TYPE\_EYE |  | 43 | 0.38 | 0.98 | 0.479 | 0.615 | 1.000 | 2908 | tags=21%, list=13%, signal=24% |
| 1755 | GO\_VACUOLAR\_TRANSPORT |  | 228 | 0.24 | 0.98 | 0.494 | 0.616 | 1.000 | 4593 | tags=26%, list=21%, signal=32% |
| 1756 | GO\_CELLULAR\_RESPONSE\_TO\_NITRIC\_OXIDE |  | 15 | 0.41 | 0.98 | 0.513 | 0.616 | 1.000 | 2442 | tags=27%, list=11%, signal=30% |
| 1757 | GO\_REGULATION\_OF\_NEURON\_DEATH |  | 239 | 0.29 | 0.98 | 0.521 | 0.617 | 1.000 | 3223 | tags=20%, list=15%, signal=23% |
| 1758 | GO\_DEFENSE\_RESPONSE\_TO\_OTHER\_ORGANISM |  | 441 | 0.33 | 0.97 | 0.482 | 0.618 | 1.000 | 3406 | tags=21%, list=16%, signal=25% |
| 1759 | GO\_REGULATION\_OF\_INTERLEUKIN\_2\_PRODUCTION |  | 45 | 0.42 | 0.97 | 0.491 | 0.620 | 1.000 | 3474 | tags=36%, list=16%, signal=42% |
| 1760 | GO\_EPITHELIAL\_CELL\_PROLIFERATION |  | 88 | 0.33 | 0.97 | 0.507 | 0.620 | 1.000 | 3298 | tags=27%, list=15%, signal=32% |
| 1761 | GO\_REGULATION\_OF\_B\_CELL\_MEDIATED\_IMMUNITY |  | 40 | 0.39 | 0.97 | 0.523 | 0.623 | 1.000 | 3457 | tags=35%, list=16%, signal=42% |
| 1762 | GO\_PHENOL\_CONTAINING\_COMPOUND\_BIOSYNTHETIC\_PROCESS |  | 31 | 0.36 | 0.97 | 0.483 | 0.624 | 1.000 | 3120 | tags=13%, list=14%, signal=15% |
| 1763 | GO\_CELL\_PROJECTION\_ASSEMBLY |  | 239 | 0.28 | 0.97 | 0.508 | 0.623 | 1.000 | 3506 | tags=20%, list=16%, signal=23% |
| 1764 | GO\_REGULATION\_OF\_HORMONE\_SECRETION |  | 252 | 0.27 | 0.97 | 0.548 | 0.626 | 1.000 | 3785 | tags=21%, list=17%, signal=25% |
| 1765 | GO\_PRIMARY\_ALCOHOL\_METABOLIC\_PROCESS |  | 43 | 0.42 | 0.97 | 0.503 | 0.626 | 1.000 | 1426 | tags=9%, list=7%, signal=10% |
| 1766 | GO\_RESPONSE\_TO\_ESTROGEN |  | 211 | 0.29 | 0.97 | 0.520 | 0.626 | 1.000 | 3201 | tags=22%, list=15%, signal=25% |
| 1767 | GO\_LYMPH\_NODE\_DEVELOPMENT |  | 17 | 0.43 | 0.97 | 0.513 | 0.625 | 1.000 | 3478 | tags=47%, list=16%, signal=56% |
| 1768 | GO\_REGULATION\_OF\_PROTEIN\_TARGETING\_TO\_MITOCHONDRION |  | 90 | 0.26 | 0.97 | 0.514 | 0.626 | 1.000 | 2875 | tags=16%, list=13%, signal=18% |
| 1769 | GO\_MODIFICATION\_BY\_SYMBIONT\_OF\_HOST\_MORPHOLOGY\_OR\_PHYSIOLOGY |  | 43 | 0.30 | 0.97 | 0.511 | 0.628 | 1.000 | 3201 | tags=21%, list=15%, signal=24% |
| 1770 | GO\_SECONDARY\_METABOLIC\_PROCESS |  | 44 | 0.38 | 0.97 | 0.522 | 0.628 | 1.000 | 3018 | tags=23%, list=14%, signal=26% |
| 1771 | GO\_POSITIVE\_REGULATION\_OF\_HEART\_RATE |  | 22 | 0.40 | 0.97 | 0.517 | 0.629 | 1.000 | 2376 | tags=23%, list=11%, signal=25% |
| 1772 | GO\_RESPONSE\_TO\_GROWTH\_HORMONE |  | 30 | 0.37 | 0.97 | 0.513 | 0.629 | 1.000 | 2183 | tags=23%, list=10%, signal=26% |
| 1773 | GO\_NEGATIVE\_REGULATION\_OF\_INTERLEUKIN\_1\_PRODUCTION |  | 16 | 0.44 | 0.97 | 0.530 | 0.630 | 1.000 | 3241 | tags=38%, list=15%, signal=44% |
| 1774 | GO\_REGULATION\_OF\_HEART\_MORPHOGENESIS |  | 27 | 0.43 | 0.97 | 0.514 | 0.631 | 1.000 | 2956 | tags=30%, list=14%, signal=34% |
| 1775 | GO\_POSITIVE\_REGULATION\_OF\_INTERLEUKIN\_1\_BETA\_PRODUCTION |  | 29 | 0.43 | 0.96 | 0.511 | 0.632 | 1.000 | 1556 | tags=17%, list=7%, signal=19% |
| 1776 | GO\_NEGATIVE\_REGULATION\_OF\_HORMONE\_SECRETION |  | 73 | 0.32 | 0.96 | 0.534 | 0.635 | 1.000 | 1568 | tags=12%, list=7%, signal=13% |
| 1777 | GO\_TRANSCRIPTION\_INITIATION\_FROM\_RNA\_POLYMERASE\_II\_PROMOTER |  | 150 | 0.23 | 0.96 | 0.529 | 0.634 | 1.000 | 2673 | tags=12%, list=12%, signal=14% |
| 1778 | GO\_EMBRYONIC\_PLACENTA\_MORPHOGENESIS |  | 22 | 0.35 | 0.96 | 0.498 | 0.634 | 1.000 | 2863 | tags=27%, list=13%, signal=31% |
| 1779 | GO\_LIPOSACCHARIDE\_METABOLIC\_PROCESS |  | 106 | 0.28 | 0.96 | 0.498 | 0.636 | 1.000 | 4406 | tags=29%, list=20%, signal=36% |
| 1780 | GO\_AXIS\_ELONGATION |  | 26 | 0.40 | 0.96 | 0.523 | 0.638 | 1.000 | 1948 | tags=19%, list=9%, signal=21% |
| 1781 | GO\_PHAGOSOME\_MATURATION |  | 35 | 0.34 | 0.96 | 0.516 | 0.638 | 1.000 | 4622 | tags=29%, list=21%, signal=36% |
| 1782 | GO\_FEAR\_RESPONSE |  | 28 | 0.35 | 0.96 | 0.495 | 0.637 | 1.000 | 3209 | tags=21%, list=15%, signal=25% |
| 1783 | GO\_COPPER\_ION\_HOMEOSTASIS |  | 16 | 0.36 | 0.96 | 0.494 | 0.638 | 1.000 | 3480 | tags=25%, list=16%, signal=30% |
| 1784 | GO\_RESPONSE\_TO\_RETINOIC\_ACID |  | 100 | 0.31 | 0.96 | 0.539 | 0.638 | 1.000 | 3170 | tags=23%, list=15%, signal=27% |
| 1785 | GO\_REGULATION\_OF\_SYNAPTIC\_VESICLE\_EXOCYTOSIS |  | 19 | 0.38 | 0.96 | 0.504 | 0.640 | 1.000 | 3873 | tags=26%, list=18%, signal=32% |
| 1786 | GO\_NEGATIVE\_REGULATION\_OF\_LIPID\_METABOLIC\_PROCESS |  | 69 | 0.32 | 0.96 | 0.569 | 0.642 | 1.000 | 1940 | tags=14%, list=9%, signal=16% |
| 1787 | GO\_POSITIVE\_REGULATION\_OF\_ADAPTIVE\_IMMUNE\_RESPONSE |  | 71 | 0.38 | 0.96 | 0.512 | 0.642 | 1.000 | 3241 | tags=28%, list=15%, signal=33% |
| 1788 | GO\_NEUROTROPHIN\_TRK\_RECEPTOR\_SIGNALING\_PATHWAY |  | 15 | 0.40 | 0.96 | 0.503 | 0.642 | 1.000 | 2811 | tags=27%, list=13%, signal=31% |
| 1789 | GO\_VESICLE\_LOCALIZATION |  | 210 | 0.25 | 0.96 | 0.557 | 0.643 | 1.000 | 3093 | tags=16%, list=14%, signal=19% |
| 1790 | GO\_AUDITORY\_RECEPTOR\_CELL\_DIFFERENTIATION |  | 27 | 0.34 | 0.96 | 0.513 | 0.643 | 1.000 | 2058 | tags=11%, list=9%, signal=12% |
| 1791 | GO\_POSITIVE\_REGULATION\_OF\_STAT\_CASCADE |  | 72 | 0.34 | 0.96 | 0.527 | 0.643 | 1.000 | 3170 | tags=26%, list=15%, signal=31% |
| 1792 | GO\_CERAMIDE\_BIOSYNTHETIC\_PROCESS |  | 40 | 0.36 | 0.96 | 0.530 | 0.644 | 1.000 | 3139 | tags=28%, list=14%, signal=32% |
| 1793 | GO\_EMBRYONIC\_DIGIT\_MORPHOGENESIS |  | 59 | 0.35 | 0.96 | 0.515 | 0.644 | 1.000 | 2430 | tags=14%, list=11%, signal=15% |
| 1794 | GO\_CELLULAR\_RESPONSE\_TO\_GROWTH\_HORMONE\_STIMULUS |  | 20 | 0.39 | 0.96 | 0.534 | 0.645 | 1.000 | 2183 | tags=25%, list=10%, signal=28% |
| 1795 | GO\_T\_CELL\_LINEAGE\_COMMITMENT |  | 15 | 0.47 | 0.95 | 0.524 | 0.646 | 1.000 | 3406 | tags=40%, list=16%, signal=47% |
| 1796 | GO\_DETOXIFICATION |  | 70 | 0.34 | 0.95 | 0.515 | 0.645 | 1.000 | 2642 | tags=20%, list=12%, signal=23% |
| 1797 | GO\_NLS\_BEARING\_PROTEIN\_IMPORT\_INTO\_NUCLEUS |  | 21 | 0.31 | 0.95 | 0.538 | 0.646 | 1.000 | 2273 | tags=19%, list=10%, signal=21% |
| 1798 | GO\_ANDROGEN\_RECEPTOR\_SIGNALING\_PATHWAY |  | 40 | 0.30 | 0.95 | 0.518 | 0.646 | 1.000 | 2113 | tags=13%, list=10%, signal=14% |
| 1799 | GO\_REGULATION\_OF\_PRI\_MIRNA\_TRANSCRIPTION\_FROM\_RNA\_POLYMERASE\_II\_PROMOTER |  | 17 | 0.35 | 0.95 | 0.499 | 0.645 | 1.000 | 3972 | tags=35%, list=18%, signal=43% |
| 1800 | GO\_REGULATION\_OF\_CARDIAC\_MUSCLE\_CELL\_DIFFERENTIATION |  | 17 | 0.47 | 0.95 | 0.539 | 0.646 | 1.000 | 3290 | tags=41%, list=15%, signal=48% |
| 1801 | GO\_BONE\_MINERALIZATION |  | 34 | 0.38 | 0.95 | 0.518 | 0.646 | 1.000 | 2112 | tags=18%, list=10%, signal=20% |
| 1802 | GO\_PHOSPHATIDYLCHOLINE\_BIOSYNTHETIC\_PROCESS |  | 26 | 0.38 | 0.95 | 0.519 | 0.646 | 1.000 | 3891 | tags=27%, list=18%, signal=33% |
| 1803 | GO\_CELLULAR\_MODIFIED\_AMINO\_ACID\_METABOLIC\_PROCESS |  | 199 | 0.31 | 0.95 | 0.548 | 0.646 | 1.000 | 2532 | tags=16%, list=12%, signal=17% |
| 1804 | GO\_XENOPHAGY |  | 98 | 0.26 | 0.95 | 0.561 | 0.646 | 1.000 | 2057 | tags=10%, list=9%, signal=11% |
| 1805 | GO\_MYELOID\_DENDRITIC\_CELL\_ACTIVATION |  | 25 | 0.47 | 0.95 | 0.530 | 0.648 | 1.000 | 3779 | tags=48%, list=17%, signal=58% |
| 1806 | GO\_BRANCHING\_MORPHOGENESIS\_OF\_AN\_EPITHELIAL\_TUBE |  | 129 | 0.33 | 0.95 | 0.527 | 0.648 | 1.000 | 3221 | tags=24%, list=15%, signal=28% |
| 1807 | GO\_WATER\_HOMEOSTASIS |  | 65 | 0.40 | 0.95 | 0.518 | 0.649 | 1.000 | 3032 | tags=25%, list=14%, signal=29% |
| 1808 | GO\_ESTABLISHMENT\_OF\_PROTEIN\_LOCALIZATION\_TO\_PLASMA\_MEMBRANE |  | 89 | 0.28 | 0.95 | 0.542 | 0.648 | 1.000 | 4030 | tags=26%, list=19%, signal=32% |
| 1809 | GO\_REGULATION\_OF\_ENDOTHELIAL\_CELL\_CHEMOTAXIS |  | 17 | 0.43 | 0.95 | 0.548 | 0.649 | 1.000 | 2760 | tags=35%, list=13%, signal=40% |
| 1810 | GO\_NEGATIVE\_REGULATION\_OF\_TRANSLATIONAL\_INITIATION |  | 18 | 0.35 | 0.95 | 0.531 | 0.649 | 1.000 | 1180 | tags=11%, list=5%, signal=12% |
| 1811 | GO\_ESTROUS\_CYCLE |  | 19 | 0.43 | 0.95 | 0.528 | 0.650 | 1.000 | 2829 | tags=21%, list=13%, signal=24% |
| 1812 | GO\_RESPONSE\_TO\_LITHIUM\_ION |  | 27 | 0.37 | 0.95 | 0.530 | 0.649 | 1.000 | 3170 | tags=30%, list=15%, signal=35% |
| 1813 | GO\_GLAND\_MORPHOGENESIS |  | 95 | 0.33 | 0.95 | 0.541 | 0.651 | 1.000 | 2956 | tags=23%, list=14%, signal=27% |
| 1814 | GO\_SEX\_DIFFERENTIATION |  | 249 | 0.27 | 0.95 | 0.575 | 0.651 | 1.000 | 2800 | tags=16%, list=13%, signal=18% |
| 1815 | GO\_CELLULAR\_AMINO\_ACID\_CATABOLIC\_PROCESS |  | 104 | 0.33 | 0.95 | 0.539 | 0.653 | 1.000 | 3182 | tags=21%, list=15%, signal=25% |
| 1816 | GO\_RESPONSE\_TO\_INTERLEUKIN\_4 |  | 30 | 0.37 | 0.95 | 0.517 | 0.653 | 1.000 | 3673 | tags=37%, list=17%, signal=44% |
| 1817 | GO\_REGULATION\_OF\_GLIOGENESIS |  | 89 | 0.31 | 0.95 | 0.546 | 0.653 | 1.000 | 3130 | tags=21%, list=14%, signal=25% |
| 1818 | GO\_ORGANELLE\_MEMBRANE\_FUSION |  | 88 | 0.27 | 0.95 | 0.542 | 0.654 | 1.000 | 3085 | tags=16%, list=14%, signal=18% |
| 1819 | GO\_POSITIVE\_REGULATION\_OF\_INTERLEUKIN\_6\_PRODUCTION |  | 64 | 0.37 | 0.95 | 0.544 | 0.654 | 1.000 | 3107 | tags=28%, list=14%, signal=33% |
| 1820 | GO\_REGULATION\_OF\_ORGAN\_FORMATION |  | 30 | 0.40 | 0.95 | 0.548 | 0.654 | 1.000 | 2956 | tags=27%, list=14%, signal=31% |
| 1821 | GO\_REGULATION\_OF\_ERBB\_SIGNALING\_PATHWAY |  | 78 | 0.27 | 0.95 | 0.538 | 0.655 | 1.000 | 3106 | tags=17%, list=14%, signal=19% |
| 1822 | GO\_EPITHELIAL\_CELL\_DIFFERENTIATION |  | 457 | 0.29 | 0.95 | 0.558 | 0.655 | 1.000 | 3508 | tags=22%, list=16%, signal=25% |
| 1823 | GO\_REGULATION\_OF\_OXIDOREDUCTASE\_ACTIVITY |  | 87 | 0.28 | 0.94 | 0.607 | 0.656 | 1.000 | 2061 | tags=15%, list=9%, signal=16% |
| 1824 | GO\_NEGATIVE\_REGULATION\_OF\_ORGAN\_GROWTH |  | 21 | 0.36 | 0.94 | 0.548 | 0.657 | 1.000 | 3221 | tags=24%, list=15%, signal=28% |
| 1825 | GO\_POSITIVE\_REGULATION\_OF\_INTERLEUKIN\_2\_PRODUCTION |  | 29 | 0.43 | 0.94 | 0.532 | 0.658 | 1.000 | 3474 | tags=38%, list=16%, signal=45% |
| 1826 | GO\_CELLULAR\_RESPONSE\_TO\_DRUG |  | 66 | 0.29 | 0.94 | 0.575 | 0.660 | 1.000 | 1737 | tags=11%, list=8%, signal=11% |
| 1827 | GO\_POSITIVE\_REGULATION\_OF\_LEUKOCYTE\_CHEMOTAXIS |  | 78 | 0.42 | 0.94 | 0.536 | 0.660 | 1.000 | 3404 | tags=36%, list=16%, signal=42% |
| 1828 | GO\_RESPONSE\_TO\_TUMOR\_NECROSIS\_FACTOR |  | 210 | 0.33 | 0.94 | 0.537 | 0.663 | 1.000 | 3420 | tags=22%, list=16%, signal=26% |
| 1829 | GO\_UNSATURATED\_FATTY\_ACID\_METABOLIC\_PROCESS |  | 100 | 0.37 | 0.94 | 0.563 | 0.663 | 1.000 | 4408 | tags=31%, list=20%, signal=39% |
| 1830 | GO\_SULFUR\_AMINO\_ACID\_BIOSYNTHETIC\_PROCESS |  | 19 | 0.36 | 0.94 | 0.505 | 0.665 | 1.000 | 2442 | tags=16%, list=11%, signal=18% |
| 1831 | GO\_POSITIVE\_REGULATION\_OF\_JUN\_KINASE\_ACTIVITY |  | 60 | 0.31 | 0.94 | 0.588 | 0.666 | 1.000 | 1921 | tags=17%, list=9%, signal=18% |
| 1832 | GO\_NEGATIVE\_REGULATION\_OF\_PROTEIN\_BINDING |  | 78 | 0.29 | 0.94 | 0.580 | 0.666 | 1.000 | 2863 | tags=17%, list=13%, signal=19% |
| 1833 | GO\_SMAD\_PROTEIN\_SIGNAL\_TRANSDUCTION |  | 53 | 0.32 | 0.94 | 0.546 | 0.666 | 1.000 | 2956 | tags=17%, list=14%, signal=20% |
| 1834 | GO\_REGULATION\_OF\_CATENIN\_IMPORT\_INTO\_NUCLEUS |  | 25 | 0.40 | 0.94 | 0.565 | 0.666 | 1.000 | 3640 | tags=36%, list=17%, signal=43% |
| 1835 | GO\_POSITIVE\_REGULATION\_OF\_ACTIN\_CYTOSKELETON\_REORGANIZATION |  | 16 | 0.39 | 0.94 | 0.558 | 0.666 | 1.000 | 4187 | tags=50%, list=19%, signal=62% |
| 1836 | GO\_JNK\_CASCADE |  | 80 | 0.28 | 0.94 | 0.586 | 0.666 | 1.000 | 1892 | tags=14%, list=9%, signal=15% |
| 1837 | GO\_CALCIUM\_ION\_REGULATED\_EXOCYTOSIS\_OF\_NEUROTRANSMITTER |  | 30 | 0.36 | 0.94 | 0.547 | 0.669 | 1.000 | 1708 | tags=13%, list=8%, signal=14% |
| 1838 | GO\_ORGANOPHOSPHATE\_BIOSYNTHETIC\_PROCESS |  | 418 | 0.23 | 0.94 | 0.657 | 0.668 | 1.000 | 4046 | tags=19%, list=19%, signal=23% |
| 1839 | GO\_VESICLE\_ORGANIZATION |  | 261 | 0.23 | 0.94 | 0.583 | 0.668 | 1.000 | 4008 | tags=19%, list=18%, signal=23% |
| 1840 | GO\_NUCLEOTIDE\_SUGAR\_METABOLIC\_PROCESS |  | 31 | 0.34 | 0.94 | 0.574 | 0.668 | 1.000 | 5559 | tags=45%, list=26%, signal=61% |
| 1841 | GO\_MACROMITOPHAGY |  | 126 | 0.25 | 0.94 | 0.606 | 0.669 | 1.000 | 3097 | tags=15%, list=14%, signal=17% |
| 1842 | GO\_NEGATIVE\_REGULATION\_OF\_NEURAL\_PRECURSOR\_CELL\_PROLIFERATION |  | 20 | 0.36 | 0.94 | 0.560 | 0.670 | 1.000 | 4845 | tags=50%, list=22%, signal=64% |
| 1843 | GO\_POSITIVE\_REGULATION\_OF\_MULTICELLULAR\_ORGANISMAL\_METABOLIC\_PROCESS |  | 20 | 0.39 | 0.93 | 0.560 | 0.670 | 1.000 | 3080 | tags=30%, list=14%, signal=35% |
| 1844 | GO\_PALLIUM\_DEVELOPMENT |  | 149 | 0.27 | 0.93 | 0.600 | 0.670 | 1.000 | 3970 | tags=25%, list=18%, signal=30% |
| 1845 | GO\_REGULATION\_OF\_CELL\_PROLIFERATION\_INVOLVED\_IN\_HEART\_MORPHOGENESIS |  | 15 | 0.42 | 0.93 | 0.547 | 0.670 | 1.000 | 2956 | tags=27%, list=14%, signal=31% |
| 1846 | GO\_SODIUM\_ION\_TRANSMEMBRANE\_TRANSPORT |  | 79 | 0.30 | 0.93 | 0.575 | 0.671 | 1.000 | 3039 | tags=15%, list=14%, signal=18% |
| 1847 | GO\_REGULATION\_OF\_CELLULAR\_KETONE\_METABOLIC\_PROCESS |  | 160 | 0.25 | 0.93 | 0.591 | 0.672 | 1.000 | 1569 | tags=9%, list=7%, signal=10% |
| 1848 | GO\_LIPID\_BIOSYNTHETIC\_PROCESS |  | 498 | 0.27 | 0.93 | 0.606 | 0.672 | 1.000 | 3284 | tags=17%, list=15%, signal=20% |
| 1849 | GO\_POSITIVE\_REGULATION\_OF\_DENDRITIC\_SPINE\_DEVELOPMENT |  | 33 | 0.32 | 0.93 | 0.560 | 0.674 | 1.000 | 2694 | tags=24%, list=12%, signal=28% |
| 1850 | GO\_POSITIVE\_REGULATION\_OF\_NOTCH\_SIGNALING\_PATHWAY |  | 33 | 0.32 | 0.93 | 0.565 | 0.674 | 1.000 | 2732 | tags=15%, list=13%, signal=17% |
| 1851 | GO\_FATTY\_ACID\_TRANSPORT |  | 54 | 0.33 | 0.93 | 0.601 | 0.675 | 1.000 | 2714 | tags=20%, list=12%, signal=23% |
| 1852 | GO\_MORPHOGENESIS\_OF\_A\_BRANCHING\_STRUCTURE |  | 164 | 0.31 | 0.93 | 0.584 | 0.675 | 1.000 | 3221 | tags=23%, list=15%, signal=27% |
| 1853 | GO\_ANION\_TRANSPORT |  | 467 | 0.25 | 0.93 | 0.672 | 0.675 | 1.000 | 2797 | tags=14%, list=13%, signal=16% |
| 1854 | GO\_NEGATIVE\_REGULATION\_OF\_NOTCH\_SIGNALING\_PATHWAY |  | 26 | 0.38 | 0.93 | 0.595 | 0.675 | 1.000 | 3176 | tags=31%, list=15%, signal=36% |
| 1855 | GO\_REGULATION\_OF\_RECEPTOR\_RECYCLING |  | 19 | 0.36 | 0.93 | 0.567 | 0.675 | 1.000 | 383 | tags=11%, list=2%, signal=11% |
| 1856 | GO\_NUCLEOSIDE\_TRIPHOSPHATE\_METABOLIC\_PROCESS |  | 204 | 0.24 | 0.93 | 0.567 | 0.675 | 1.000 | 1654 | tags=7%, list=8%, signal=7% |
| 1857 | GO\_RESPONSE\_TO\_OSMOTIC\_STRESS |  | 62 | 0.30 | 0.93 | 0.624 | 0.675 | 1.000 | 1966 | tags=15%, list=9%, signal=16% |
| 1858 | GO\_POSITIVE\_REGULATION\_OF\_LEUKOCYTE\_MIGRATION |  | 105 | 0.40 | 0.93 | 0.549 | 0.678 | 1.000 | 3170 | tags=31%, list=15%, signal=37% |
| 1859 | GO\_STAT\_CASCADE |  | 46 | 0.35 | 0.93 | 0.569 | 0.678 | 1.000 | 3080 | tags=24%, list=14%, signal=28% |
| 1860 | GO\_SALIVARY\_GLAND\_DEVELOPMENT |  | 32 | 0.38 | 0.93 | 0.587 | 0.678 | 1.000 | 2898 | tags=28%, list=13%, signal=32% |
| 1861 | GO\_CELLULAR\_MODIFIED\_AMINO\_ACID\_BIOSYNTHETIC\_PROCESS |  | 48 | 0.31 | 0.93 | 0.587 | 0.678 | 1.000 | 1940 | tags=15%, list=9%, signal=16% |
| 1862 | GO\_POSITIVE\_REGULATION\_OF\_INTERLEUKIN\_1\_PRODUCTION |  | 34 | 0.42 | 0.93 | 0.572 | 0.678 | 1.000 | 2059 | tags=21%, list=9%, signal=23% |
| 1863 | GO\_SOMATIC\_STEM\_CELL\_DIVISION |  | 21 | 0.41 | 0.93 | 0.557 | 0.678 | 1.000 | 2863 | tags=33%, list=13%, signal=38% |
| 1864 | GO\_REGULATION\_OF\_TRIGLYCERIDE\_BIOSYNTHETIC\_PROCESS |  | 17 | 0.38 | 0.93 | 0.552 | 0.678 | 1.000 | 4650 | tags=35%, list=21%, signal=45% |
| 1865 | GO\_GLUTATHIONE\_METABOLIC\_PROCESS |  | 54 | 0.40 | 0.93 | 0.561 | 0.678 | 1.000 | 2410 | tags=20%, list=11%, signal=23% |
| 1866 | GO\_CORTICAL\_CYTOSKELETON\_ORGANIZATION |  | 35 | 0.33 | 0.93 | 0.586 | 0.678 | 1.000 | 3399 | tags=26%, list=16%, signal=30% |
| 1867 | GO\_NEURON\_CELL\_CELL\_ADHESION |  | 16 | 0.42 | 0.93 | 0.580 | 0.681 | 1.000 | 2079 | tags=19%, list=10%, signal=21% |
| 1868 | GO\_INTERACTION\_WITH\_HOST |  | 122 | 0.30 | 0.92 | 0.552 | 0.681 | 1.000 | 3201 | tags=23%, list=15%, signal=27% |
| 1869 | GO\_MEMBRANE\_LIPID\_METABOLIC\_PROCESS |  | 168 | 0.27 | 0.92 | 0.608 | 0.682 | 1.000 | 4428 | tags=28%, list=20%, signal=35% |
| 1870 | GO\_NEGATIVE\_REGULATION\_OF\_GLUCOSE\_TRANSPORT |  | 15 | 0.41 | 0.92 | 0.562 | 0.682 | 1.000 | 700 | tags=20%, list=3%, signal=21% |
| 1871 | GO\_RESPONSE\_TO\_STEROL |  | 22 | 0.36 | 0.92 | 0.610 | 0.684 | 1.000 | 2280 | tags=23%, list=10%, signal=25% |
| 1872 | GO\_POSITIVE\_REGULATION\_OF\_PROTEIN\_COMPLEX\_DISASSEMBLY |  | 21 | 0.44 | 0.92 | 0.583 | 0.686 | 1.000 | 1358 | tags=19%, list=6%, signal=20% |
| 1873 | GO\_POSITIVE\_REGULATION\_OF\_CARDIAC\_MUSCLE\_TISSUE\_DEVELOPMENT |  | 27 | 0.38 | 0.92 | 0.562 | 0.688 | 1.000 | 3290 | tags=37%, list=15%, signal=44% |
| 1874 | GO\_CYTOKINE\_MEDIATED\_SIGNALING\_PATHWAY |  | 426 | 0.34 | 0.92 | 0.578 | 0.688 | 1.000 | 3514 | tags=23%, list=16%, signal=27% |
| 1875 | GO\_REGULATION\_OF\_NEURAL\_PRECURSOR\_CELL\_PROLIFERATION |  | 69 | 0.31 | 0.92 | 0.621 | 0.690 | 1.000 | 3967 | tags=28%, list=18%, signal=34% |
| 1876 | GO\_PLATELET\_AGGREGATION |  | 37 | 0.32 | 0.92 | 0.594 | 0.691 | 1.000 | 1447 | tags=14%, list=7%, signal=14% |
| 1877 | GO\_CEREBRAL\_CORTEX\_NEURON\_DIFFERENTIATION |  | 22 | 0.36 | 0.92 | 0.596 | 0.691 | 1.000 | 2643 | tags=18%, list=12%, signal=21% |
| 1878 | GO\_INTERLEUKIN\_1\_PRODUCTION |  | 15 | 0.47 | 0.92 | 0.585 | 0.693 | 1.000 | 3241 | tags=40%, list=15%, signal=47% |
| 1879 | GO\_EPITHELIAL\_TUBE\_BRANCHING\_INVOLVED\_IN\_LUNG\_MORPHOGENESIS |  | 25 | 0.38 | 0.92 | 0.576 | 0.693 | 1.000 | 1948 | tags=20%, list=9%, signal=22% |
| 1880 | GO\_METANEPHRIC\_EPITHELIUM\_DEVELOPMENT |  | 19 | 0.40 | 0.92 | 0.591 | 0.695 | 1.000 | 3267 | tags=26%, list=15%, signal=31% |
| 1881 | GO\_CANONICAL\_WNT\_SIGNALING\_PATHWAY |  | 89 | 0.30 | 0.92 | 0.585 | 0.695 | 1.000 | 1347 | tags=12%, list=6%, signal=13% |
| 1882 | GO\_CELL\_AGGREGATION |  | 19 | 0.46 | 0.92 | 0.597 | 0.696 | 1.000 | 3250 | tags=32%, list=15%, signal=37% |
| 1883 | GO\_CELLULAR\_RESPONSE\_TO\_IONIZING\_RADIATION |  | 50 | 0.31 | 0.91 | 0.579 | 0.697 | 1.000 | 803 | tags=10%, list=4%, signal=10% |
| 1884 | GO\_MEMBRANE\_FUSION |  | 139 | 0.24 | 0.91 | 0.651 | 0.698 | 1.000 | 4003 | tags=21%, list=18%, signal=25% |
| 1885 | GO\_REGULATION\_OF\_INTRINSIC\_APOPTOTIC\_SIGNALING\_PATHWAY |  | 131 | 0.25 | 0.91 | 0.644 | 0.698 | 1.000 | 2658 | tags=12%, list=12%, signal=14% |
| 1886 | GO\_CERAMIDE\_METABOLIC\_PROCESS |  | 69 | 0.31 | 0.91 | 0.598 | 0.699 | 1.000 | 4406 | tags=33%, list=20%, signal=42% |
| 1887 | GO\_POSITIVE\_REGULATION\_OF\_CHEMOKINE\_PRODUCTION |  | 47 | 0.37 | 0.91 | 0.610 | 0.700 | 1.000 | 3166 | tags=32%, list=15%, signal=37% |
| 1888 | GO\_MODULATION\_BY\_VIRUS\_OF\_HOST\_MORPHOLOGY\_OR\_PHYSIOLOGY |  | 35 | 0.29 | 0.91 | 0.575 | 0.700 | 1.000 | 3032 | tags=20%, list=14%, signal=23% |
| 1889 | GO\_GLUTAMATE\_SECRETION |  | 27 | 0.36 | 0.91 | 0.563 | 0.700 | 1.000 | 3652 | tags=30%, list=17%, signal=36% |
| 1890 | GO\_NEGATIVE\_REGULATION\_OF\_CARTILAGE\_DEVELOPMENT |  | 25 | 0.41 | 0.91 | 0.567 | 0.699 | 1.000 | 3936 | tags=36%, list=18%, signal=44% |
| 1891 | GO\_NEGATIVE\_REGULATION\_OF\_RESPONSE\_TO\_DNA\_DAMAGE\_STIMULUS |  | 45 | 0.29 | 0.91 | 0.613 | 0.699 | 1.000 | 1206 | tags=9%, list=6%, signal=9% |
| 1892 | GO\_REGULATION\_OF\_HORMONE\_LEVELS |  | 448 | 0.26 | 0.91 | 0.711 | 0.700 | 1.000 | 3785 | tags=20%, list=17%, signal=24% |
| 1893 | GO\_REGIONALIZATION |  | 294 | 0.29 | 0.91 | 0.654 | 0.700 | 1.000 | 3353 | tags=20%, list=15%, signal=23% |
| 1894 | GO\_ALPHA\_AMINO\_ACID\_CATABOLIC\_PROCESS |  | 87 | 0.31 | 0.91 | 0.627 | 0.700 | 1.000 | 3182 | tags=20%, list=15%, signal=23% |
| 1895 | GO\_MOVEMENT\_IN\_ENVIRONMENT\_OF\_OTHER\_ORGANISM\_INVOLVED\_IN\_SYMBIOTIC\_INTERACTION |  | 78 | 0.32 | 0.91 | 0.585 | 0.701 | 1.000 | 3125 | tags=27%, list=14%, signal=31% |
| 1896 | GO\_POSITIVE\_REGULATION\_OF\_DNA\_TEMPLATED\_TRANSCRIPTION\_INITIATION |  | 24 | 0.31 | 0.91 | 0.591 | 0.703 | 1.000 | 1206 | tags=13%, list=6%, signal=13% |
| 1897 | GO\_NEGATIVE\_REGULATION\_OF\_T\_CELL\_RECEPTOR\_SIGNALING\_PATHWAY |  | 16 | 0.38 | 0.91 | 0.559 | 0.702 | 1.000 | 3732 | tags=38%, list=17%, signal=45% |
| 1898 | GO\_PEPTIDYL\_PROLINE\_MODIFICATION |  | 46 | 0.29 | 0.91 | 0.632 | 0.706 | 1.000 | 2652 | tags=17%, list=12%, signal=20% |
| 1899 | GO\_PYRIMIDINE\_CONTAINING\_COMPOUND\_METABOLIC\_PROCESS |  | 66 | 0.28 | 0.91 | 0.663 | 0.706 | 1.000 | 3308 | tags=23%, list=15%, signal=27% |
| 1900 | GO\_GRANULOCYTE\_ACTIVATION |  | 18 | 0.45 | 0.91 | 0.577 | 0.708 | 1.000 | 2464 | tags=39%, list=11%, signal=44% |
| 1901 | GO\_MAMMARY\_GLAND\_MORPHOGENESIS |  | 38 | 0.35 | 0.91 | 0.593 | 0.707 | 1.000 | 2956 | tags=24%, list=14%, signal=27% |
| 1902 | GO\_REGULATION\_OF\_IMMUNOGLOBULIN\_PRODUCTION |  | 47 | 0.36 | 0.91 | 0.595 | 0.709 | 1.000 | 3176 | tags=30%, list=15%, signal=35% |
| 1903 | GO\_POSITIVE\_REGULATION\_OF\_NF\_KAPPAB\_TRANSCRIPTION\_FACTOR\_ACTIVITY |  | 127 | 0.30 | 0.90 | 0.630 | 0.711 | 1.000 | 1428 | tags=11%, list=7%, signal=12% |
| 1904 | GO\_POSITIVE\_REGULATION\_OF\_KIDNEY\_DEVELOPMENT |  | 41 | 0.36 | 0.90 | 0.653 | 0.712 | 1.000 | 1608 | tags=17%, list=7%, signal=18% |
| 1905 | GO\_RETINA\_LAYER\_FORMATION |  | 21 | 0.35 | 0.90 | 0.615 | 0.713 | 1.000 | 1445 | tags=14%, list=7%, signal=15% |
| 1906 | GO\_REGULATION\_OF\_B\_CELL\_APOPTOTIC\_PROCESS |  | 18 | 0.39 | 0.90 | 0.624 | 0.713 | 1.000 | 2294 | tags=33%, list=11%, signal=37% |
| 1907 | GO\_NEGATIVE\_REGULATION\_OF\_LIPID\_TRANSPORT |  | 25 | 0.35 | 0.90 | 0.602 | 0.713 | 1.000 | 3755 | tags=24%, list=17%, signal=29% |
| 1908 | GO\_REGULATION\_OF\_RESPONSE\_TO\_INTERFERON\_GAMMA |  | 22 | 0.41 | 0.90 | 0.575 | 0.713 | 1.000 | 3084 | tags=27%, list=14%, signal=32% |
| 1909 | GO\_POSITIVE\_REGULATION\_OF\_IMMUNOGLOBULIN\_PRODUCTION |  | 31 | 0.38 | 0.90 | 0.588 | 0.716 | 1.000 | 3084 | tags=32%, list=14%, signal=38% |
| 1910 | GO\_NEGATIVE\_REGULATION\_OF\_T\_CELL\_PROLIFERATION |  | 49 | 0.36 | 0.90 | 0.611 | 0.715 | 1.000 | 3735 | tags=33%, list=17%, signal=39% |
| 1911 | GO\_GLANDULAR\_EPITHELIAL\_CELL\_DEVELOPMENT |  | 17 | 0.37 | 0.90 | 0.615 | 0.715 | 1.000 | 5101 | tags=53%, list=23%, signal=69% |
| 1912 | GO\_RESPONSE\_TO\_ISOQUINOLINE\_ALKALOID |  | 30 | 0.30 | 0.90 | 0.633 | 0.715 | 1.000 | 3691 | tags=23%, list=17%, signal=28% |
| 1913 | GO\_INTESTINAL\_ABSORPTION |  | 26 | 0.38 | 0.90 | 0.611 | 0.715 | 1.000 | 442 | tags=8%, list=2%, signal=8% |
| 1914 | GO\_REGULATION\_OF\_ODONTOGENESIS |  | 23 | 0.38 | 0.90 | 0.605 | 0.715 | 1.000 | 3691 | tags=30%, list=17%, signal=37% |
| 1915 | GO\_SIGNAL\_RELEASE |  | 161 | 0.26 | 0.90 | 0.699 | 0.715 | 1.000 | 3785 | tags=20%, list=17%, signal=24% |
| 1916 | GO\_REACTIVE\_OXYGEN\_SPECIES\_BIOSYNTHETIC\_PROCESS |  | 23 | 0.41 | 0.90 | 0.610 | 0.715 | 1.000 | 2748 | tags=26%, list=13%, signal=30% |
| 1917 | GO\_RESPONSE\_TO\_INTERFERON\_GAMMA |  | 131 | 0.40 | 0.90 | 0.602 | 0.716 | 1.000 | 4778 | tags=40%, list=22%, signal=51% |
| 1918 | GO\_POSITIVE\_REGULATION\_OF\_BLOOD\_VESSEL\_ENDOTHELIAL\_CELL\_MIGRATION |  | 25 | 0.38 | 0.90 | 0.610 | 0.716 | 1.000 | 3370 | tags=32%, list=15%, signal=38% |
| 1919 | GO\_POSITIVE\_REGULATION\_OF\_STEM\_CELL\_PROLIFERATION |  | 61 | 0.34 | 0.90 | 0.618 | 0.717 | 1.000 | 3761 | tags=33%, list=17%, signal=40% |
| 1920 | GO\_CELLULAR\_RESPONSE\_TO\_INTERLEUKIN\_4 |  | 25 | 0.37 | 0.90 | 0.586 | 0.717 | 1.000 | 2863 | tags=32%, list=13%, signal=37% |
| 1921 | GO\_LIPID\_OXIDATION |  | 68 | 0.31 | 0.90 | 0.597 | 0.718 | 1.000 | 4880 | tags=34%, list=22%, signal=43% |
| 1922 | GO\_CYTOKINE\_PRODUCTION\_INVOLVED\_IN\_IMMUNE\_RESPONSE |  | 17 | 0.39 | 0.90 | 0.632 | 0.720 | 1.000 | 3775 | tags=35%, list=17%, signal=43% |
| 1923 | GO\_RETINAL\_GANGLION\_CELL\_AXON\_GUIDANCE |  | 18 | 0.40 | 0.90 | 0.635 | 0.720 | 1.000 | 2898 | tags=22%, list=13%, signal=26% |
| 1924 | GO\_RESPONSE\_TO\_PLATELET\_DERIVED\_GROWTH\_FACTOR |  | 18 | 0.41 | 0.90 | 0.621 | 0.720 | 1.000 | 2112 | tags=28%, list=10%, signal=31% |
| 1925 | GO\_CAMERA\_TYPE\_EYE\_PHOTORECEPTOR\_CELL\_DIFFERENTIATION |  | 15 | 0.42 | 0.90 | 0.617 | 0.720 | 1.000 | 2112 | tags=20%, list=10%, signal=22% |
| 1926 | GO\_NEURON\_MATURATION |  | 29 | 0.35 | 0.90 | 0.619 | 0.722 | 1.000 | 1465 | tags=14%, list=7%, signal=15% |
| 1927 | GO\_REGULATION\_OF\_GLIAL\_CELL\_PROLIFERATION |  | 21 | 0.35 | 0.89 | 0.623 | 0.722 | 1.000 | 2716 | tags=19%, list=12%, signal=22% |
| 1928 | GO\_PROTEIN\_KINASE\_C\_ACTIVATING\_G\_PROTEIN\_COUPLED\_RECEPTOR\_SIGNALING\_PATHWAY |  | 31 | 0.32 | 0.89 | 0.620 | 0.722 | 1.000 | 4144 | tags=29%, list=19%, signal=36% |
| 1929 | GO\_REGULATION\_OF\_CYTOKINE\_BIOSYNTHETIC\_PROCESS |  | 91 | 0.35 | 0.89 | 0.601 | 0.724 | 1.000 | 3736 | tags=31%, list=17%, signal=37% |
| 1930 | GO\_MORPHOGENESIS\_OF\_EMBRYONIC\_EPITHELIUM |  | 133 | 0.29 | 0.89 | 0.641 | 0.723 | 1.000 | 3351 | tags=20%, list=15%, signal=24% |
| 1931 | GO\_REGULATION\_OF\_EXTRACELLULAR\_MATRIX\_ORGANIZATION |  | 24 | 0.37 | 0.89 | 0.643 | 0.723 | 1.000 | 1049 | tags=13%, list=5%, signal=13% |
| 1932 | GO\_EXOCRINE\_SYSTEM\_DEVELOPMENT |  | 44 | 0.35 | 0.89 | 0.648 | 0.724 | 1.000 | 3130 | tags=27%, list=14%, signal=32% |
| 1933 | GO\_REGULATION\_OF\_CIRCADIAN\_RHYTHM |  | 98 | 0.25 | 0.89 | 0.707 | 0.726 | 1.000 | 2925 | tags=14%, list=13%, signal=16% |
| 1934 | GO\_REGULATION\_OF\_CELLULAR\_PH |  | 71 | 0.27 | 0.89 | 0.649 | 0.726 | 1.000 | 4622 | tags=27%, list=21%, signal=34% |
| 1935 | GO\_BLOOD\_VESSEL\_ENDOTHELIAL\_CELL\_MIGRATION |  | 23 | 0.36 | 0.89 | 0.627 | 0.728 | 1.000 | 4442 | tags=48%, list=20%, signal=60% |
| 1936 | GO\_PHOSPHATIDYLETHANOLAMINE\_METABOLIC\_PROCESS |  | 17 | 0.36 | 0.89 | 0.601 | 0.728 | 1.000 | 3651 | tags=29%, list=17%, signal=35% |
| 1937 | GO\_PHOSPHATIDYLGLYCEROL\_METABOLIC\_PROCESS |  | 30 | 0.34 | 0.89 | 0.623 | 0.728 | 1.000 | 4408 | tags=27%, list=20%, signal=33% |
| 1938 | GO\_SMOOTHENED\_SIGNALING\_PATHWAY |  | 68 | 0.32 | 0.89 | 0.659 | 0.730 | 1.000 | 3318 | tags=21%, list=15%, signal=24% |
| 1939 | GO\_REGULATION\_OF\_TOLL\_LIKE\_RECEPTOR\_4\_SIGNALING\_PATHWAY |  | 16 | 0.39 | 0.89 | 0.637 | 0.730 | 1.000 | 3511 | tags=38%, list=16%, signal=45% |
| 1940 | GO\_CIRCADIAN\_REGULATION\_OF\_GENE\_EXPRESSION |  | 53 | 0.26 | 0.89 | 0.639 | 0.730 | 1.000 | 3372 | tags=23%, list=16%, signal=27% |
| 1941 | GO\_ANION\_TRANSMEMBRANE\_TRANSPORT |  | 231 | 0.24 | 0.89 | 0.773 | 0.733 | 1.000 | 3233 | tags=15%, list=15%, signal=17% |
| 1942 | GO\_METANEPHRIC\_NEPHRON\_MORPHOGENESIS |  | 20 | 0.38 | 0.89 | 0.646 | 0.732 | 1.000 | 3267 | tags=30%, list=15%, signal=35% |
| 1943 | GO\_REGULATION\_OF\_T\_CELL\_MEDIATED\_IMMUNITY |  | 50 | 0.36 | 0.89 | 0.648 | 0.734 | 1.000 | 4052 | tags=32%, list=19%, signal=39% |
| 1944 | GO\_REGULATION\_OF\_DNA\_TEMPLATED\_TRANSCRIPTION\_INITIATION |  | 31 | 0.29 | 0.89 | 0.631 | 0.734 | 1.000 | 1733 | tags=13%, list=8%, signal=14% |
| 1945 | GO\_REGULATION\_OF\_DEFENSE\_RESPONSE\_TO\_VIRUS |  | 179 | 0.27 | 0.89 | 0.634 | 0.735 | 1.000 | 2057 | tags=12%, list=9%, signal=13% |
| 1946 | GO\_REGULATION\_OF\_PROTEIN\_DEACETYLATION |  | 32 | 0.30 | 0.89 | 0.630 | 0.735 | 1.000 | 4601 | tags=34%, list=21%, signal=44% |
| 1947 | GO\_DRUG\_TRANSPORT |  | 25 | 0.39 | 0.88 | 0.651 | 0.738 | 1.000 | 1735 | tags=12%, list=8%, signal=13% |
| 1948 | GO\_GLAND\_DEVELOPMENT |  | 379 | 0.26 | 0.88 | 0.794 | 0.738 | 1.000 | 3058 | tags=19%, list=14%, signal=22% |
| 1949 | GO\_POSITIVE\_REGULATION\_OF\_B\_CELL\_MEDIATED\_IMMUNITY |  | 25 | 0.39 | 0.88 | 0.641 | 0.738 | 1.000 | 3084 | tags=36%, list=14%, signal=42% |
| 1950 | GO\_CELLULAR\_RESPONSE\_TO\_HYDROGEN\_PEROXIDE |  | 60 | 0.29 | 0.88 | 0.626 | 0.738 | 1.000 | 4161 | tags=30%, list=19%, signal=37% |
| 1951 | GO\_NEGATIVE\_REGULATION\_OF\_HISTONE\_ACETYLATION |  | 15 | 0.33 | 0.88 | 0.618 | 0.741 | 1.000 | 3222 | tags=27%, list=15%, signal=31% |
| 1952 | GO\_LABYRINTHINE\_LAYER\_BLOOD\_VESSEL\_DEVELOPMENT |  | 18 | 0.34 | 0.88 | 0.649 | 0.741 | 1.000 | 2486 | tags=28%, list=11%, signal=31% |
| 1953 | GO\_PARASYMPATHETIC\_NERVOUS\_SYSTEM\_DEVELOPMENT |  | 16 | 0.40 | 0.88 | 0.621 | 0.741 | 1.000 | 2898 | tags=25%, list=13%, signal=29% |
| 1954 | GO\_PROSTATE\_GLAND\_MORPHOGENESIS |  | 23 | 0.38 | 0.88 | 0.667 | 0.742 | 1.000 | 1528 | tags=17%, list=7%, signal=19% |
| 1955 | GO\_PROTEIN\_LOCALIZATION\_TO\_CILIUM |  | 24 | 0.32 | 0.88 | 0.613 | 0.741 | 1.000 | 6275 | tags=54%, list=29%, signal=76% |
| 1956 | GO\_INFLAMMATORY\_RESPONSE\_TO\_ANTIGENIC\_STIMULUS |  | 25 | 0.38 | 0.88 | 0.633 | 0.743 | 1.000 | 3735 | tags=36%, list=17%, signal=43% |
| 1957 | GO\_REPRODUCTIVE\_BEHAVIOR |  | 28 | 0.30 | 0.88 | 0.655 | 0.744 | 1.000 | 6200 | tags=36%, list=29%, signal=50% |
| 1958 | GO\_OSTEOCLAST\_DIFFERENTIATION |  | 28 | 0.33 | 0.88 | 0.631 | 0.744 | 1.000 | 2280 | tags=21%, list=10%, signal=24% |
| 1959 | GO\_MYOBLAST\_DIFFERENTIATION |  | 37 | 0.30 | 0.88 | 0.667 | 0.744 | 1.000 | 3290 | tags=19%, list=15%, signal=22% |
| 1960 | GO\_NEGATIVE\_REGULATION\_OF\_PROTEIN\_MATURATION |  | 32 | 0.32 | 0.88 | 0.673 | 0.744 | 1.000 | 2911 | tags=19%, list=13%, signal=22% |
| 1961 | GO\_PYRIMIDINE\_CONTAINING\_COMPOUND\_CATABOLIC\_PROCESS |  | 28 | 0.30 | 0.88 | 0.707 | 0.744 | 1.000 | 3308 | tags=21%, list=15%, signal=25% |
| 1962 | GO\_ACTIVATION\_OF\_JUN\_KINASE\_ACTIVITY |  | 33 | 0.31 | 0.88 | 0.702 | 0.744 | 1.000 | 1531 | tags=12%, list=7%, signal=13% |
| 1963 | GO\_PROTEIN\_O\_LINKED\_GLYCOSYLATION |  | 89 | 0.32 | 0.88 | 0.632 | 0.745 | 1.000 | 3937 | tags=25%, list=18%, signal=30% |
| 1964 | GO\_REGULATION\_OF\_RECEPTOR\_BIOSYNTHETIC\_PROCESS |  | 21 | 0.33 | 0.88 | 0.615 | 0.744 | 1.000 | 4152 | tags=24%, list=19%, signal=29% |
| 1965 | GO\_POSITIVE\_REGULATION\_OF\_LYMPHOCYTE\_APOPTOTIC\_PROCESS |  | 18 | 0.41 | 0.88 | 0.648 | 0.745 | 1.000 | 1926 | tags=22%, list=9%, signal=24% |
| 1966 | GO\_REGULATION\_OF\_NATURAL\_KILLER\_CELL\_ACTIVATION |  | 26 | 0.36 | 0.88 | 0.634 | 0.744 | 1.000 | 4373 | tags=42%, list=20%, signal=53% |
| 1967 | GO\_VESICLE\_DOCKING\_INVOLVED\_IN\_EXOCYTOSIS |  | 34 | 0.28 | 0.88 | 0.650 | 0.744 | 1.000 | 2772 | tags=18%, list=13%, signal=20% |
| 1968 | GO\_DEFENSE\_RESPONSE\_TO\_FUNGUS |  | 35 | 0.35 | 0.88 | 0.669 | 0.744 | 1.000 | 4017 | tags=23%, list=18%, signal=28% |
| 1969 | GO\_NEGATIVE\_REGULATION\_OF\_EPITHELIAL\_TO\_MESENCHYMAL\_TRANSITION |  | 22 | 0.34 | 0.88 | 0.692 | 0.745 | 1.000 | 664 | tags=9%, list=3%, signal=9% |
| 1970 | GO\_POSITIVE\_REGULATION\_OF\_CYTOKINE\_BIOSYNTHETIC\_PROCESS |  | 57 | 0.38 | 0.88 | 0.632 | 0.745 | 1.000 | 3736 | tags=33%, list=17%, signal=40% |
| 1971 | GO\_RESPONSE\_TO\_PROGESTERONE |  | 46 | 0.34 | 0.88 | 0.688 | 0.745 | 1.000 | 2112 | tags=22%, list=10%, signal=24% |
| 1972 | GO\_CELL\_FATE\_SPECIFICATION |  | 68 | 0.31 | 0.88 | 0.681 | 0.745 | 1.000 | 2898 | tags=13%, list=13%, signal=15% |
| 1973 | GO\_NEGATIVE\_REGULATION\_OF\_CYTOKINE\_BIOSYNTHETIC\_PROCESS |  | 27 | 0.35 | 0.88 | 0.675 | 0.745 | 1.000 | 2733 | tags=22%, list=13%, signal=25% |
| 1974 | GO\_REVERSE\_CHOLESTEROL\_TRANSPORT |  | 17 | 0.36 | 0.87 | 0.632 | 0.745 | 1.000 | 2363 | tags=18%, list=11%, signal=20% |
| 1975 | GO\_REGULATION\_OF\_MICROTUBULE\_POLYMERIZATION |  | 31 | 0.26 | 0.87 | 0.701 | 0.746 | 1.000 | 3599 | tags=23%, list=17%, signal=27% |
| 1976 | GO\_PEPTIDYL\_THREONINE\_MODIFICATION |  | 44 | 0.25 | 0.87 | 0.705 | 0.746 | 1.000 | 3962 | tags=23%, list=18%, signal=28% |
| 1977 | GO\_SULFUR\_AMINO\_ACID\_METABOLIC\_PROCESS |  | 39 | 0.30 | 0.87 | 0.679 | 0.745 | 1.000 | 2874 | tags=15%, list=13%, signal=18% |
| 1978 | GO\_GENETIC\_IMPRINTING |  | 19 | 0.37 | 0.87 | 0.640 | 0.745 | 1.000 | 4363 | tags=42%, list=20%, signal=53% |
| 1979 | GO\_REGULATION\_OF\_NITRIC\_OXIDE\_SYNTHASE\_ACTIVITY |  | 47 | 0.28 | 0.87 | 0.741 | 0.746 | 1.000 | 1925 | tags=17%, list=9%, signal=19% |
| 1980 | GO\_ORGANIC\_ANION\_TRANSPORT |  | 352 | 0.24 | 0.87 | 0.837 | 0.747 | 1.000 | 2797 | tags=15%, list=13%, signal=17% |
| 1981 | GO\_REGULATION\_OF\_INTERLEUKIN\_8\_PRODUCTION |  | 56 | 0.33 | 0.87 | 0.646 | 0.747 | 1.000 | 2680 | tags=23%, list=12%, signal=26% |
| 1982 | GO\_NEGATIVE\_REGULATION\_OF\_TRANSCRIPTION\_REGULATORY\_REGION\_DNA\_BINDING |  | 17 | 0.40 | 0.87 | 0.618 | 0.747 | 1.000 | 2430 | tags=24%, list=11%, signal=26% |
| 1983 | GO\_POSITIVE\_REGULATION\_OF\_PROTEIN\_ACETYLATION |  | 36 | 0.29 | 0.87 | 0.745 | 0.751 | 1.000 | 4204 | tags=33%, list=19%, signal=41% |
| 1984 | GO\_CELLULAR\_RESPONSE\_TO\_VIRUS |  | 21 | 0.40 | 0.87 | 0.652 | 0.751 | 1.000 | 1288 | tags=10%, list=6%, signal=10% |
| 1985 | GO\_ORGAN\_INDUCTION |  | 15 | 0.42 | 0.87 | 0.664 | 0.751 | 1.000 | 1608 | tags=27%, list=7%, signal=29% |
| 1986 | GO\_CYTOKINE\_METABOLIC\_PROCESS |  | 17 | 0.38 | 0.87 | 0.681 | 0.754 | 1.000 | 2764 | tags=24%, list=13%, signal=27% |
| 1987 | GO\_NEGATIVE\_REGULATION\_OF\_DNA\_BINDING |  | 46 | 0.30 | 0.87 | 0.697 | 0.753 | 1.000 | 3166 | tags=24%, list=15%, signal=28% |
| 1988 | GO\_COLUMNAR\_CUBOIDAL\_EPITHELIAL\_CELL\_DEVELOPMENT |  | 45 | 0.29 | 0.87 | 0.766 | 0.755 | 1.000 | 4659 | tags=29%, list=21%, signal=37% |
| 1989 | GO\_ANTIGEN\_PROCESSING\_AND\_PRESENTATION\_OF\_ENDOGENOUS\_ANTIGEN |  | 17 | 0.39 | 0.87 | 0.618 | 0.757 | 1.000 | 2735 | tags=24%, list=13%, signal=27% |
| 1990 | GO\_EPITHELIAL\_CELL\_DEVELOPMENT |  | 180 | 0.25 | 0.87 | 0.820 | 0.758 | 1.000 | 2670 | tags=16%, list=12%, signal=18% |
| 1991 | GO\_REGULATION\_OF\_VIRAL\_ENTRY\_INTO\_HOST\_CELL |  | 27 | 0.33 | 0.87 | 0.631 | 0.757 | 1.000 | 4362 | tags=26%, list=20%, signal=32% |
| 1992 | GO\_REGULATION\_OF\_CELL\_MIGRATION\_INVOLVED\_IN\_SPROUTING\_ANGIOGENESIS |  | 19 | 0.35 | 0.87 | 0.685 | 0.757 | 1.000 | 1999 | tags=21%, list=9%, signal=23% |
| 1993 | GO\_POSITIVE\_REGULATION\_OF\_LEUKOCYTE\_DEGRANULATION |  | 18 | 0.37 | 0.87 | 0.628 | 0.757 | 1.000 | 2428 | tags=28%, list=11%, signal=31% |
| 1994 | GO\_PYRIMIDINE\_NUCLEOSIDE\_CATABOLIC\_PROCESS |  | 18 | 0.37 | 0.87 | 0.682 | 0.757 | 1.000 | 3308 | tags=22%, list=15%, signal=26% |
| 1995 | GO\_CELL\_SURFACE\_RECEPTOR\_SIGNALING\_PATHWAY\_INVOLVED\_IN\_HEART\_DEVELOPMENT |  | 15 | 0.40 | 0.86 | 0.624 | 0.759 | 1.000 | 2430 | tags=27%, list=11%, signal=30% |
| 1996 | GO\_REFLEX |  | 19 | 0.35 | 0.86 | 0.656 | 0.760 | 1.000 | 4068 | tags=21%, list=19%, signal=26% |
| 1997 | GO\_MYELOID\_LEUKOCYTE\_MEDIATED\_IMMUNITY |  | 40 | 0.35 | 0.86 | 0.671 | 0.761 | 1.000 | 3201 | tags=28%, list=15%, signal=32% |
| 1998 | GO\_RESPONSE\_TO\_EPINEPHRINE |  | 16 | 0.39 | 0.86 | 0.646 | 0.762 | 1.000 | 2040 | tags=25%, list=9%, signal=28% |
| 1999 | GO\_REGULATION\_OF\_T\_HELPER\_CELL\_DIFFERENTIATION |  | 26 | 0.38 | 0.86 | 0.654 | 0.762 | 1.000 | 3783 | tags=35%, list=17%, signal=42% |
| 2000 | GO\_CYTOPLASMIC\_MICROTUBULE\_ORGANIZATION |  | 38 | 0.28 | 0.86 | 0.669 | 0.766 | 1.000 | 3305 | tags=18%, list=15%, signal=22% |
| 2001 | GO\_NUCLEOSIDE\_PHOSPHATE\_CATABOLIC\_PROCESS |  | 65 | 0.25 | 0.86 | 0.764 | 0.765 | 1.000 | 1962 | tags=12%, list=9%, signal=13% |
| 2002 | GO\_NUCLEOSIDE\_BISPHOSPHATE\_METABOLIC\_PROCESS |  | 35 | 0.32 | 0.86 | 0.685 | 0.767 | 1.000 | 3555 | tags=23%, list=16%, signal=27% |
| 2003 | GO\_ANTIGEN\_PROCESSING\_AND\_PRESENTATION |  | 197 | 0.28 | 0.86 | 0.663 | 0.768 | 1.000 | 3104 | tags=21%, list=14%, signal=24% |
| 2004 | GO\_FC\_EPSILON\_RECEPTOR\_SIGNALING\_PATHWAY |  | 122 | 0.26 | 0.86 | 0.662 | 0.768 | 1.000 | 2889 | tags=15%, list=13%, signal=17% |
| 2005 | GO\_POSITIVE\_REGULATION\_OF\_SYNAPSE\_ASSEMBLY |  | 51 | 0.35 | 0.86 | 0.691 | 0.769 | 1.000 | 2001 | tags=14%, list=9%, signal=15% |
| 2006 | GO\_ORGANOPHOSPHATE\_ESTER\_TRANSPORT |  | 83 | 0.25 | 0.86 | 0.763 | 0.769 | 1.000 | 3751 | tags=18%, list=17%, signal=22% |
| 2007 | GO\_RESPONSE\_TO\_TOPOLOGICALLY\_INCORRECT\_PROTEIN |  | 153 | 0.24 | 0.86 | 0.715 | 0.769 | 1.000 | 4576 | tags=24%, list=21%, signal=30% |
| 2008 | GO\_RESPONSE\_TO\_ISCHEMIA |  | 26 | 0.31 | 0.86 | 0.683 | 0.772 | 1.000 | 2089 | tags=15%, list=10%, signal=17% |
| 2009 | GO\_NEGATIVE\_REGULATION\_OF\_DENDRITE\_MORPHOGENESIS |  | 15 | 0.35 | 0.86 | 0.675 | 0.772 | 1.000 | 4170 | tags=47%, list=19%, signal=58% |
| 2010 | GO\_LUNG\_MORPHOGENESIS |  | 45 | 0.31 | 0.86 | 0.717 | 0.772 | 1.000 | 1948 | tags=16%, list=9%, signal=17% |
| 2011 | GO\_NUCLEOSIDE\_BISPHOSPHATE\_BIOSYNTHETIC\_PROCESS |  | 16 | 0.34 | 0.85 | 0.645 | 0.773 | 1.000 | 3323 | tags=25%, list=15%, signal=29% |
| 2012 | GO\_POSITIVE\_REGULATION\_OF\_DENDRITE\_MORPHOGENESIS |  | 31 | 0.29 | 0.85 | 0.696 | 0.773 | 1.000 | 3256 | tags=23%, list=15%, signal=27% |
| 2013 | GO\_MONOCYTE\_CHEMOTAXIS |  | 36 | 0.42 | 0.85 | 0.680 | 0.774 | 1.000 | 1783 | tags=22%, list=8%, signal=24% |
| 2014 | GO\_EMBRYONIC\_HEART\_TUBE\_MORPHOGENESIS |  | 58 | 0.27 | 0.85 | 0.679 | 0.773 | 1.000 | 3655 | tags=17%, list=17%, signal=21% |
| 2015 | GO\_REGULATION\_OF\_ENDOCRINE\_PROCESS |  | 47 | 0.30 | 0.85 | 0.700 | 0.777 | 1.000 | 2862 | tags=19%, list=13%, signal=22% |
| 2016 | GO\_NEGATIVE\_REGULATION\_OF\_INTERLEUKIN\_2\_PRODUCTION |  | 16 | 0.40 | 0.85 | 0.677 | 0.776 | 1.000 | 2946 | tags=31%, list=14%, signal=36% |
| 2017 | GO\_POSITIVE\_REGULATION\_OF\_MYELOID\_CELL\_DIFFERENTIATION |  | 77 | 0.28 | 0.85 | 0.783 | 0.777 | 1.000 | 3578 | tags=25%, list=16%, signal=29% |
| 2018 | GO\_LENS\_DEVELOPMENT\_IN\_CAMERA\_TYPE\_EYE |  | 63 | 0.30 | 0.85 | 0.778 | 0.777 | 1.000 | 1829 | tags=17%, list=8%, signal=19% |
| 2019 | GO\_IRON\_ION\_TRANSPORT |  | 51 | 0.29 | 0.85 | 0.727 | 0.777 | 1.000 | 3775 | tags=24%, list=17%, signal=28% |
| 2020 | GO\_RESPONSE\_TO\_IMMOBILIZATION\_STRESS |  | 22 | 0.34 | 0.85 | 0.703 | 0.779 | 1.000 | 3170 | tags=23%, list=15%, signal=27% |
| 2021 | GO\_DOPAMINERGIC\_NEURON\_DIFFERENTIATION |  | 26 | 0.34 | 0.85 | 0.702 | 0.779 | 1.000 | 1316 | tags=12%, list=6%, signal=12% |
| 2022 | GO\_SPHINGOLIPID\_BIOSYNTHETIC\_PROCESS |  | 67 | 0.30 | 0.85 | 0.721 | 0.781 | 1.000 | 4360 | tags=30%, list=20%, signal=37% |
| 2023 | GO\_ORGANIC\_ACID\_CATABOLIC\_PROCESS |  | 192 | 0.28 | 0.85 | 0.737 | 0.781 | 1.000 | 4937 | tags=32%, list=23%, signal=41% |
| 2024 | GO\_REGULATION\_OF\_HYDROGEN\_PEROXIDE\_INDUCED\_CELL\_DEATH |  | 17 | 0.31 | 0.85 | 0.735 | 0.781 | 1.000 | 2386 | tags=18%, list=11%, signal=20% |
| 2025 | GO\_MONOSACCHARIDE\_METABOLIC\_PROCESS |  | 179 | 0.26 | 0.85 | 0.772 | 0.782 | 1.000 | 2598 | tags=15%, list=12%, signal=17% |
| 2026 | GO\_REGULATION\_OF\_INTERFERON\_BETA\_PRODUCTION |  | 43 | 0.35 | 0.85 | 0.671 | 0.784 | 1.000 | 1739 | tags=12%, list=8%, signal=13% |
| 2027 | GO\_MAINTENANCE\_OF\_GASTROINTESTINAL\_EPITHELIUM |  | 15 | 0.40 | 0.85 | 0.715 | 0.785 | 1.000 | 361 | tags=7%, list=2%, signal=7% |
| 2028 | GO\_POSITIVE\_REGULATION\_OF\_TOR\_SIGNALING |  | 27 | 0.28 | 0.84 | 0.697 | 0.786 | 1.000 | 2912 | tags=19%, list=13%, signal=21% |
| 2029 | GO\_CELLULAR\_RESPONSE\_TO\_REACTIVE\_OXYGEN\_SPECIES |  | 102 | 0.26 | 0.84 | 0.712 | 0.788 | 1.000 | 2860 | tags=18%, list=13%, signal=20% |
| 2030 | GO\_RESPONSE\_TO\_EXOGENOUS\_DSRNA |  | 44 | 0.32 | 0.84 | 0.677 | 0.788 | 1.000 | 549 | tags=7%, list=3%, signal=7% |
| 2031 | GO\_INTERACTION\_WITH\_SYMBIONT |  | 50 | 0.26 | 0.84 | 0.749 | 0.789 | 1.000 | 2863 | tags=16%, list=13%, signal=18% |
| 2032 | GO\_HISTONE\_H3\_DEACETYLATION |  | 21 | 0.28 | 0.84 | 0.676 | 0.790 | 1.000 | 4152 | tags=29%, list=19%, signal=35% |
| 2033 | GO\_SYNAPSE\_ASSEMBLY |  | 64 | 0.32 | 0.84 | 0.767 | 0.790 | 1.000 | 1811 | tags=17%, list=8%, signal=19% |
| 2034 | GO\_NEGATIVE\_REGULATION\_OF\_COAGULATION |  | 47 | 0.32 | 0.84 | 0.757 | 0.792 | 1.000 | 2862 | tags=23%, list=13%, signal=27% |
| 2035 | GO\_MODIFICATION\_OF\_MORPHOLOGY\_OR\_PHYSIOLOGY\_OF\_OTHER\_ORGANISM |  | 95 | 0.26 | 0.84 | 0.702 | 0.792 | 1.000 | 3201 | tags=18%, list=15%, signal=21% |
| 2036 | GO\_ACROSOME\_REACTION |  | 16 | 0.35 | 0.84 | 0.714 | 0.794 | 1.000 | 6341 | tags=50%, list=29%, signal=71% |
| 2037 | GO\_POSITIVE\_REGULATION\_OF\_T\_HELPER\_CELL\_DIFFERENTIATION |  | 18 | 0.41 | 0.84 | 0.695 | 0.794 | 1.000 | 804 | tags=17%, list=4%, signal=17% |
| 2038 | GO\_T\_CELL\_HOMEOSTASIS |  | 34 | 0.32 | 0.84 | 0.694 | 0.795 | 1.000 | 3478 | tags=29%, list=16%, signal=35% |
| 2039 | GO\_DOPAMINE\_RECEPTOR\_SIGNALING\_PATHWAY |  | 29 | 0.26 | 0.84 | 0.673 | 0.797 | 1.000 | 4596 | tags=21%, list=21%, signal=26% |
| 2040 | GO\_NEPHRON\_TUBULE\_FORMATION |  | 18 | 0.38 | 0.84 | 0.734 | 0.797 | 1.000 | 3138 | tags=28%, list=14%, signal=32% |
| 2041 | GO\_RESPONSE\_TO\_SALT\_STRESS |  | 19 | 0.35 | 0.84 | 0.743 | 0.798 | 1.000 | 2692 | tags=21%, list=12%, signal=24% |
| 2042 | GO\_POSITIVE\_REGULATION\_OF\_STRIATED\_MUSCLE\_CONTRACTION |  | 15 | 0.35 | 0.84 | 0.699 | 0.798 | 1.000 | 1703 | tags=13%, list=8%, signal=14% |
| 2043 | GO\_FATTY\_ACID\_CATABOLIC\_PROCESS |  | 71 | 0.30 | 0.83 | 0.690 | 0.802 | 1.000 | 4880 | tags=35%, list=22%, signal=45% |
| 2044 | GO\_HOMOTYPIC\_CELL\_CELL\_ADHESION |  | 48 | 0.28 | 0.83 | 0.822 | 0.801 | 1.000 | 3578 | tags=25%, list=16%, signal=30% |
| 2045 | GO\_POSITIVE\_REGULATION\_OF\_EXOCYTOSIS |  | 80 | 0.24 | 0.83 | 0.812 | 0.801 | 1.000 | 3630 | tags=21%, list=17%, signal=25% |
| 2046 | GO\_NEGATIVE\_REGULATION\_OF\_PROTEIN\_ACETYLATION |  | 20 | 0.31 | 0.83 | 0.697 | 0.801 | 1.000 | 3222 | tags=25%, list=15%, signal=29% |
| 2047 | GO\_MICROVILLUS\_ORGANIZATION |  | 19 | 0.30 | 0.83 | 0.722 | 0.802 | 1.000 | 3506 | tags=26%, list=16%, signal=31% |
| 2048 | GO\_PROSTATE\_GLAND\_DEVELOPMENT |  | 41 | 0.30 | 0.83 | 0.820 | 0.802 | 1.000 | 1528 | tags=12%, list=7%, signal=13% |
| 2049 | GO\_MYELOID\_DENDRITIC\_CELL\_DIFFERENTIATION |  | 19 | 0.42 | 0.83 | 0.699 | 0.804 | 1.000 | 3779 | tags=53%, list=17%, signal=64% |
| 2050 | GO\_NEUROMUSCULAR\_PROCESS\_CONTROLLING\_BALANCE |  | 47 | 0.24 | 0.83 | 0.770 | 0.805 | 1.000 | 3379 | tags=17%, list=16%, signal=20% |
| 2051 | GO\_HYPOTHALAMUS\_DEVELOPMENT |  | 23 | 0.28 | 0.83 | 0.691 | 0.805 | 1.000 | 2898 | tags=17%, list=13%, signal=20% |
| 2052 | GO\_CELL\_SUBSTRATE\_JUNCTION\_ASSEMBLY |  | 40 | 0.36 | 0.83 | 0.680 | 0.804 | 1.000 | 1574 | tags=20%, list=7%, signal=22% |
| 2053 | GO\_LONG\_TERM\_SYNAPTIC\_POTENTIATION |  | 39 | 0.30 | 0.83 | 0.733 | 0.804 | 1.000 | 3652 | tags=26%, list=17%, signal=31% |
| 2054 | GO\_HORMONE\_TRANSPORT |  | 75 | 0.27 | 0.83 | 0.819 | 0.804 | 1.000 | 3785 | tags=23%, list=17%, signal=27% |
| 2055 | GO\_MAINTENANCE\_OF\_PROTEIN\_LOCALIZATION\_IN\_ORGANELLE |  | 28 | 0.25 | 0.83 | 0.680 | 0.806 | 1.000 | 3754 | tags=18%, list=17%, signal=22% |
| 2056 | GO\_POSITIVE\_REGULATION\_OF\_NATURAL\_KILLER\_CELL\_ACTIVATION |  | 17 | 0.35 | 0.83 | 0.724 | 0.807 | 1.000 | 4373 | tags=47%, list=20%, signal=59% |
| 2057 | GO\_REGULATION\_OF\_MACROPHAGE\_DIFFERENTIATION |  | 20 | 0.32 | 0.83 | 0.701 | 0.807 | 1.000 | 2240 | tags=20%, list=10%, signal=22% |
| 2058 | GO\_LYMPH\_VESSEL\_DEVELOPMENT |  | 19 | 0.37 | 0.83 | 0.696 | 0.808 | 1.000 | 4493 | tags=53%, list=21%, signal=66% |
| 2059 | GO\_MESENCHYMAL\_TO\_EPITHELIAL\_TRANSITION |  | 15 | 0.39 | 0.83 | 0.744 | 0.807 | 1.000 | 3138 | tags=27%, list=14%, signal=31% |
| 2060 | GO\_THYROID\_HORMONE\_METABOLIC\_PROCESS |  | 15 | 0.42 | 0.83 | 0.724 | 0.810 | 1.000 | 148 | tags=7%, list=1%, signal=7% |
| 2061 | GO\_DIGESTIVE\_SYSTEM\_PROCESS |  | 60 | 0.34 | 0.83 | 0.709 | 0.810 | 1.000 | 442 | tags=7%, list=2%, signal=7% |
| 2062 | GO\_NADP\_METABOLIC\_PROCESS |  | 27 | 0.32 | 0.83 | 0.687 | 0.809 | 1.000 | 3373 | tags=19%, list=16%, signal=22% |
| 2063 | GO\_GOLGI\_TO\_PLASMA\_MEMBRANE\_TRANSPORT |  | 40 | 0.24 | 0.83 | 0.798 | 0.809 | 1.000 | 3825 | tags=25%, list=18%, signal=30% |
| 2064 | GO\_NEGATIVE\_REGULATION\_OF\_T\_CELL\_DIFFERENTIATION |  | 31 | 0.30 | 0.82 | 0.765 | 0.813 | 1.000 | 3176 | tags=23%, list=15%, signal=26% |
| 2065 | GO\_RESPONSE\_TO\_AMINE |  | 47 | 0.27 | 0.82 | 0.815 | 0.813 | 1.000 | 1881 | tags=13%, list=9%, signal=14% |
| 2066 | GO\_NEURAL\_RETINA\_DEVELOPMENT |  | 49 | 0.28 | 0.82 | 0.739 | 0.813 | 1.000 | 2182 | tags=14%, list=10%, signal=16% |
| 2067 | GO\_SENSORY\_PERCEPTION\_OF\_LIGHT\_STIMULUS |  | 200 | 0.21 | 0.82 | 0.877 | 0.814 | 1.000 | 2433 | tags=9%, list=11%, signal=10% |
| 2068 | GO\_TRACHEA\_DEVELOPMENT |  | 20 | 0.31 | 0.82 | 0.766 | 0.815 | 1.000 | 4204 | tags=30%, list=19%, signal=37% |
| 2069 | GO\_QUATERNARY\_AMMONIUM\_GROUP\_TRANSPORT |  | 17 | 0.37 | 0.82 | 0.723 | 0.815 | 1.000 | 2192 | tags=18%, list=10%, signal=20% |
| 2070 | GO\_NEGATIVE\_REGULATION\_OF\_FIBROBLAST\_PROLIFERATION |  | 26 | 0.29 | 0.82 | 0.738 | 0.819 | 1.000 | 2782 | tags=19%, list=13%, signal=22% |
| 2071 | GO\_REGULATION\_OF\_MICROTUBULE\_BASED\_MOVEMENT |  | 16 | 0.28 | 0.82 | 0.692 | 0.821 | 1.000 | 4489 | tags=31%, list=21%, signal=39% |
| 2072 | GO\_ACTIN\_NUCLEATION |  | 22 | 0.28 | 0.82 | 0.752 | 0.821 | 1.000 | 3008 | tags=18%, list=14%, signal=21% |
| 2073 | GO\_TRANSITION\_METAL\_ION\_TRANSPORT |  | 100 | 0.25 | 0.82 | 0.818 | 0.822 | 1.000 | 3838 | tags=20%, list=18%, signal=24% |
| 2074 | GO\_INTRACELLULAR\_LIPID\_TRANSPORT |  | 19 | 0.32 | 0.81 | 0.745 | 0.825 | 1.000 | 578 | tags=11%, list=3%, signal=11% |
| 2075 | GO\_STEROL\_TRANSPORT |  | 49 | 0.27 | 0.81 | 0.818 | 0.825 | 1.000 | 3759 | tags=20%, list=17%, signal=25% |
| 2076 | GO\_RESPONSE\_TO\_ENDOPLASMIC\_RETICULUM\_STRESS |  | 220 | 0.20 | 0.81 | 0.800 | 0.825 | 1.000 | 4576 | tags=22%, list=21%, signal=28% |
| 2077 | GO\_MEMBRANE\_PROTEIN\_ECTODOMAIN\_PROTEOLYSIS |  | 20 | 0.31 | 0.81 | 0.775 | 0.828 | 1.000 | 1556 | tags=15%, list=7%, signal=16% |
| 2078 | GO\_REGULATION\_OF\_INTRACELLULAR\_ESTROGEN\_RECEPTOR\_SIGNALING\_PATHWAY |  | 25 | 0.29 | 0.81 | 0.794 | 0.828 | 1.000 | 1583 | tags=12%, list=7%, signal=13% |
| 2079 | GO\_NEGATIVE\_REGULATION\_OF\_CHONDROCYTE\_DIFFERENTIATION |  | 19 | 0.39 | 0.81 | 0.725 | 0.829 | 1.000 | 3936 | tags=37%, list=18%, signal=45% |
| 2080 | GO\_ANTIGEN\_PROCESSING\_AND\_PRESENTATION\_OF\_PEPTIDE\_ANTIGEN\_VIA\_MHC\_CLASS\_I |  | 87 | 0.27 | 0.81 | 0.652 | 0.829 | 1.000 | 2428 | tags=13%, list=11%, signal=14% |
| 2081 | GO\_STEROL\_HOMEOSTASIS |  | 55 | 0.27 | 0.81 | 0.837 | 0.830 | 1.000 | 3441 | tags=18%, list=16%, signal=22% |
| 2082 | GO\_NEGATIVE\_REGULATION\_OF\_GLIAL\_CELL\_DIFFERENTIATION |  | 26 | 0.31 | 0.81 | 0.755 | 0.830 | 1.000 | 4552 | tags=27%, list=21%, signal=34% |
| 2083 | GO\_POST\_GOLGI\_VESICLE\_MEDIATED\_TRANSPORT |  | 80 | 0.22 | 0.81 | 0.824 | 0.831 | 1.000 | 3825 | tags=24%, list=18%, signal=29% |
| 2084 | GO\_MAMMARY\_GLAND\_EPITHELIUM\_DEVELOPMENT |  | 51 | 0.30 | 0.81 | 0.795 | 0.833 | 1.000 | 2795 | tags=22%, list=13%, signal=25% |
| 2085 | GO\_NEGATIVE\_REGULATION\_OF\_RESPONSE\_TO\_REACTIVE\_OXYGEN\_SPECIES |  | 17 | 0.29 | 0.81 | 0.796 | 0.834 | 1.000 | 2386 | tags=18%, list=11%, signal=20% |
| 2086 | GO\_ARP2\_3\_COMPLEX\_MEDIATED\_ACTIN\_NUCLEATION |  | 16 | 0.31 | 0.81 | 0.748 | 0.834 | 1.000 | 1450 | tags=13%, list=7%, signal=13% |
| 2087 | GO\_RENAL\_SYSTEM\_PROCESS\_INVOLVED\_IN\_REGULATION\_OF\_BLOOD\_VOLUME |  | 17 | 0.33 | 0.81 | 0.769 | 0.834 | 1.000 | 4760 | tags=47%, list=22%, signal=60% |
| 2088 | GO\_RESPONSE\_TO\_OXYGEN\_RADICAL |  | 18 | 0.30 | 0.81 | 0.746 | 0.833 | 1.000 | 911 | tags=11%, list=4%, signal=12% |
| 2089 | GO\_REGULATION\_OF\_VASCULAR\_ENDOTHELIAL\_GROWTH\_FACTOR\_RECEPTOR\_SIGNALING\_PATHWAY |  | 25 | 0.33 | 0.81 | 0.727 | 0.833 | 1.000 | 1564 | tags=16%, list=7%, signal=17% |
| 2090 | GO\_GASTRULATION\_WITH\_MOUTH\_FORMING\_SECOND |  | 26 | 0.27 | 0.80 | 0.754 | 0.836 | 1.000 | 1228 | tags=8%, list=6%, signal=8% |
| 2091 | GO\_RESPONSE\_TO\_DIETARY\_EXCESS |  | 21 | 0.34 | 0.80 | 0.829 | 0.836 | 1.000 | 2401 | tags=19%, list=11%, signal=21% |
| 2092 | GO\_MEMBRANE\_BUDDING |  | 109 | 0.20 | 0.80 | 0.840 | 0.839 | 1.000 | 4751 | tags=23%, list=22%, signal=29% |
| 2093 | GO\_REGULATION\_OF\_LIPOPROTEIN\_LIPASE\_ACTIVITY |  | 15 | 0.34 | 0.80 | 0.767 | 0.840 | 1.000 | 2811 | tags=27%, list=13%, signal=31% |
| 2094 | GO\_RESPONSE\_TO\_COCAINE |  | 45 | 0.25 | 0.80 | 0.845 | 0.845 | 1.000 | 2099 | tags=13%, list=10%, signal=15% |
| 2095 | GO\_ODONTOGENESIS\_OF\_DENTIN\_CONTAINING\_TOOTH |  | 73 | 0.29 | 0.80 | 0.832 | 0.847 | 1.000 | 3039 | tags=19%, list=14%, signal=22% |
| 2096 | GO\_RESPONSE\_TO\_NERVE\_GROWTH\_FACTOR |  | 36 | 0.25 | 0.80 | 0.794 | 0.849 | 1.000 | 4274 | tags=28%, list=20%, signal=35% |
| 2097 | GO\_LUNG\_CELL\_DIFFERENTIATION |  | 24 | 0.34 | 0.80 | 0.775 | 0.849 | 1.000 | 2732 | tags=21%, list=13%, signal=24% |
| 2098 | GO\_INSULIN\_SECRETION |  | 36 | 0.29 | 0.79 | 0.787 | 0.851 | 1.000 | 5366 | tags=39%, list=25%, signal=52% |
| 2099 | GO\_PERIPHERAL\_NERVOUS\_SYSTEM\_DEVELOPMENT |  | 64 | 0.28 | 0.79 | 0.886 | 0.851 | 1.000 | 3755 | tags=27%, list=17%, signal=32% |
| 2100 | GO\_ENDOSOME\_TO\_LYSOSOME\_TRANSPORT |  | 40 | 0.25 | 0.79 | 0.782 | 0.852 | 1.000 | 3557 | tags=20%, list=16%, signal=24% |
| 2101 | GO\_MANNOSYLATION |  | 27 | 0.30 | 0.79 | 0.734 | 0.854 | 1.000 | 3081 | tags=19%, list=14%, signal=22% |
| 2102 | GO\_NEGATIVE\_REGULATION\_OF\_ERBB\_SIGNALING\_PATHWAY |  | 44 | 0.24 | 0.79 | 0.781 | 0.854 | 1.000 | 3106 | tags=20%, list=14%, signal=24% |
| 2103 | GO\_RETINA\_HOMEOSTASIS |  | 58 | 0.30 | 0.79 | 0.738 | 0.856 | 1.000 | 3824 | tags=29%, list=18%, signal=35% |
| 2104 | GO\_REGULATION\_OF\_NEURON\_PROJECTION\_REGENERATION |  | 20 | 0.31 | 0.79 | 0.812 | 0.856 | 1.000 | 4187 | tags=35%, list=19%, signal=43% |
| 2105 | GO\_REGULATION\_OF\_NITRIC\_OXIDE\_SYNTHASE\_BIOSYNTHETIC\_PROCESS |  | 18 | 0.35 | 0.79 | 0.783 | 0.857 | 1.000 | 3080 | tags=33%, list=14%, signal=39% |
| 2106 | GO\_CELLULAR\_RESPONSE\_TO\_FLUID\_SHEAR\_STRESS |  | 19 | 0.34 | 0.79 | 0.801 | 0.859 | 1.000 | 3170 | tags=26%, list=15%, signal=31% |
| 2107 | GO\_POSITIVE\_REGULATION\_OF\_ACUTE\_INFLAMMATORY\_RESPONSE |  | 26 | 0.39 | 0.79 | 0.792 | 0.859 | 1.000 | 2428 | tags=38%, list=11%, signal=43% |
| 2108 | GO\_EPIDERMIS\_MORPHOGENESIS |  | 28 | 0.35 | 0.79 | 0.782 | 0.858 | 1.000 | 2453 | tags=18%, list=11%, signal=20% |
| 2109 | GO\_PEPTIDE\_TRANSPORT |  | 69 | 0.26 | 0.79 | 0.860 | 0.858 | 1.000 | 4662 | tags=28%, list=21%, signal=35% |
| 2110 | GO\_RESPONSE\_TO\_VIRUS |  | 228 | 0.31 | 0.79 | 0.732 | 0.858 | 1.000 | 3084 | tags=18%, list=14%, signal=21% |
| 2111 | GO\_NATURAL\_KILLER\_CELL\_ACTIVATION\_INVOLVED\_IN\_IMMUNE\_RESPONSE |  | 24 | 0.29 | 0.79 | 0.753 | 0.860 | 1.000 | 2917 | tags=13%, list=13%, signal=14% |
| 2112 | GO\_REGULATION\_OF\_DENDRITIC\_SPINE\_MORPHOGENESIS |  | 29 | 0.29 | 0.79 | 0.850 | 0.859 | 1.000 | 1921 | tags=17%, list=9%, signal=19% |
| 2113 | GO\_CARBOHYDRATE\_BIOSYNTHETIC\_PROCESS |  | 117 | 0.24 | 0.79 | 0.893 | 0.860 | 1.000 | 2230 | tags=12%, list=10%, signal=13% |
| 2114 | GO\_ZINC\_ION\_HOMEOSTASIS |  | 21 | 0.29 | 0.78 | 0.790 | 0.861 | 1.000 | 4371 | tags=24%, list=20%, signal=30% |
| 2115 | GO\_POSITIVE\_REGULATION\_OF\_CIRCADIAN\_RHYTHM |  | 20 | 0.30 | 0.78 | 0.776 | 0.862 | 1.000 | 2646 | tags=15%, list=12%, signal=17% |
| 2116 | GO\_REGULATION\_OF\_ISOTYPE\_SWITCHING |  | 24 | 0.33 | 0.78 | 0.746 | 0.863 | 1.000 | 3176 | tags=33%, list=15%, signal=39% |
| 2117 | GO\_NEGATIVE\_REGULATION\_OF\_WOUND\_HEALING |  | 57 | 0.29 | 0.78 | 0.897 | 0.863 | 1.000 | 2464 | tags=19%, list=11%, signal=22% |
| 2118 | GO\_NEUROBLAST\_PROLIFERATION |  | 28 | 0.32 | 0.78 | 0.764 | 0.863 | 1.000 | 2863 | tags=21%, list=13%, signal=25% |
| 2119 | GO\_EPITHELIAL\_CELL\_APOPTOTIC\_PROCESS |  | 25 | 0.28 | 0.78 | 0.773 | 0.863 | 1.000 | 1957 | tags=16%, list=9%, signal=18% |
| 2120 | GO\_POSITIVE\_REGULATION\_OF\_SUBSTRATE\_ADHESION\_DEPENDENT\_CELL\_SPREADING |  | 26 | 0.29 | 0.78 | 0.828 | 0.865 | 1.000 | 2074 | tags=12%, list=10%, signal=13% |
| 2121 | GO\_PROTEIN\_LOCALIZATION\_TO\_ENDOPLASMIC\_RETICULUM |  | 106 | 0.16 | 0.78 | 0.715 | 0.865 | 1.000 | 91 | tags=1%, list=0%, signal=1% |
| 2122 | GO\_CELLULAR\_CARBOHYDRATE\_CATABOLIC\_PROCESS |  | 31 | 0.27 | 0.78 | 0.842 | 0.865 | 1.000 | 4951 | tags=32%, list=23%, signal=42% |
| 2123 | GO\_POSITIVE\_REGULATION\_OF\_INTERLEUKIN\_8\_PRODUCTION |  | 44 | 0.32 | 0.78 | 0.797 | 0.868 | 1.000 | 3032 | tags=25%, list=14%, signal=29% |
| 2124 | GO\_PYRIMIDINE\_NUCLEOSIDE\_METABOLIC\_PROCESS |  | 41 | 0.26 | 0.78 | 0.851 | 0.867 | 1.000 | 3308 | tags=20%, list=15%, signal=23% |
| 2125 | GO\_CELLULAR\_RESPONSE\_TO\_TOXIC\_SUBSTANCE |  | 25 | 0.28 | 0.78 | 0.884 | 0.868 | 1.000 | 4583 | tags=36%, list=21%, signal=46% |
| 2126 | GO\_ANTIGEN\_PROCESSING\_AND\_PRESENTATION\_OF\_PEPTIDE\_ANTIGEN |  | 165 | 0.25 | 0.78 | 0.748 | 0.869 | 1.000 | 2662 | tags=16%, list=12%, signal=19% |
| 2127 | GO\_MEMBRANE\_PROTEIN\_PROTEOLYSIS |  | 33 | 0.27 | 0.78 | 0.798 | 0.870 | 1.000 | 3289 | tags=21%, list=15%, signal=25% |
| 2128 | GO\_SYNAPTIC\_TRANSMISSION\_CHOLINERGIC |  | 33 | 0.31 | 0.78 | 0.816 | 0.870 | 1.000 | 1969 | tags=12%, list=9%, signal=13% |
| 2129 | GO\_AMINO\_ACID\_BETAINE\_METABOLIC\_PROCESS |  | 18 | 0.31 | 0.77 | 0.816 | 0.872 | 1.000 | 4695 | tags=33%, list=22%, signal=42% |
| 2130 | GO\_ESTABLISHMENT\_OF\_PROTEIN\_LOCALIZATION\_TO\_GOLGI |  | 15 | 0.29 | 0.77 | 0.749 | 0.872 | 1.000 | 5665 | tags=47%, list=26%, signal=63% |
| 2131 | GO\_REGULATION\_OF\_ANOIKIS |  | 24 | 0.29 | 0.77 | 0.813 | 0.872 | 1.000 | 44 | tags=4%, list=0%, signal=4% |
| 2132 | GO\_CELLULAR\_RESPONSE\_TO\_CALCIUM\_ION |  | 44 | 0.26 | 0.77 | 0.879 | 0.873 | 1.000 | 658 | tags=5%, list=3%, signal=5% |
| 2133 | GO\_RESPONSE\_TO\_WATER |  | 17 | 0.31 | 0.77 | 0.786 | 0.873 | 1.000 | 2376 | tags=18%, list=11%, signal=20% |
| 2134 | GO\_ALCOHOL\_METABOLIC\_PROCESS |  | 333 | 0.22 | 0.77 | 0.938 | 0.873 | 1.000 | 4170 | tags=20%, list=19%, signal=25% |
| 2135 | GO\_REGULATION\_OF\_CGMP\_BIOSYNTHETIC\_PROCESS |  | 21 | 0.31 | 0.77 | 0.838 | 0.874 | 1.000 | 3502 | tags=24%, list=16%, signal=28% |
| 2136 | GO\_REGULATION\_OF\_OLIGODENDROCYTE\_DIFFERENTIATION |  | 28 | 0.30 | 0.77 | 0.853 | 0.875 | 1.000 | 2956 | tags=18%, list=14%, signal=21% |
| 2137 | GO\_HYDROGEN\_PEROXIDE\_METABOLIC\_PROCESS |  | 29 | 0.32 | 0.77 | 0.807 | 0.876 | 1.000 | 4682 | tags=31%, list=22%, signal=39% |
| 2138 | GO\_SPINAL\_CORD\_DEVELOPMENT |  | 98 | 0.26 | 0.77 | 0.880 | 0.877 | 1.000 | 3139 | tags=18%, list=14%, signal=21% |
| 2139 | GO\_TERPENOID\_METABOLIC\_PROCESS |  | 95 | 0.28 | 0.77 | 0.880 | 0.879 | 1.000 | 2433 | tags=15%, list=11%, signal=17% |
| 2140 | GO\_NEGATIVE\_REGULATION\_OF\_DEFENSE\_RESPONSE\_TO\_VIRUS |  | 17 | 0.34 | 0.77 | 0.770 | 0.881 | 1.000 | 4362 | tags=35%, list=20%, signal=44% |
| 2141 | GO\_RESPONSE\_TO\_FUNGUS |  | 47 | 0.30 | 0.77 | 0.882 | 0.881 | 1.000 | 3623 | tags=23%, list=17%, signal=28% |
| 2142 | GO\_RESPONSE\_TO\_DSRNA |  | 66 | 0.25 | 0.77 | 0.811 | 0.881 | 1.000 | 1635 | tags=8%, list=8%, signal=8% |
| 2143 | GO\_REGULATION\_OF\_CELLULAR\_RESPIRATION |  | 22 | 0.28 | 0.77 | 0.845 | 0.881 | 1.000 | 3026 | tags=18%, list=14%, signal=21% |
| 2144 | GO\_GRANULOCYTE\_MIGRATION |  | 66 | 0.36 | 0.77 | 0.785 | 0.881 | 1.000 | 3526 | tags=32%, list=16%, signal=38% |
| 2145 | GO\_GTP\_METABOLIC\_PROCESS |  | 19 | 0.31 | 0.76 | 0.885 | 0.882 | 1.000 | 4391 | tags=42%, list=20%, signal=53% |
| 2146 | GO\_DNA\_CATABOLIC\_PROCESS\_ENDONUCLEOLYTIC |  | 19 | 0.29 | 0.76 | 0.827 | 0.883 | 1.000 | 2146 | tags=16%, list=10%, signal=18% |
| 2147 | GO\_POSITIVE\_REGULATION\_OF\_REGULATED\_SECRETORY\_PATHWAY |  | 47 | 0.24 | 0.76 | 0.921 | 0.888 | 1.000 | 3630 | tags=21%, list=17%, signal=25% |
| 2148 | GO\_CELLULAR\_RESPONSE\_TO\_ANTIBIOTIC |  | 16 | 0.32 | 0.76 | 0.866 | 0.889 | 1.000 | 3067 | tags=25%, list=14%, signal=29% |
| 2149 | GO\_PROTEIN\_PALMITOYLATION |  | 23 | 0.27 | 0.76 | 0.795 | 0.888 | 1.000 | 1601 | tags=9%, list=7%, signal=9% |
| 2150 | GO\_MESODERMAL\_CELL\_DIFFERENTIATION |  | 25 | 0.31 | 0.76 | 0.846 | 0.888 | 1.000 | 3049 | tags=24%, list=14%, signal=28% |
| 2151 | GO\_LIPID\_STORAGE |  | 25 | 0.31 | 0.76 | 0.855 | 0.888 | 1.000 | 692 | tags=12%, list=3%, signal=12% |
| 2152 | GO\_CHOLESTEROL\_EFFLUX |  | 26 | 0.27 | 0.76 | 0.790 | 0.890 | 1.000 | 3759 | tags=23%, list=17%, signal=28% |
| 2153 | GO\_NEGATIVE\_REGULATION\_OF\_STEM\_CELL\_PROLIFERATION |  | 15 | 0.33 | 0.76 | 0.854 | 0.891 | 1.000 | 2956 | tags=27%, list=14%, signal=31% |
| 2154 | GO\_REGULATION\_OF\_ACTIVIN\_RECEPTOR\_SIGNALING\_PATHWAY |  | 23 | 0.31 | 0.76 | 0.786 | 0.891 | 1.000 | 2716 | tags=17%, list=12%, signal=20% |
| 2155 | GO\_REGULATION\_OF\_IMMUNOGLOBULIN\_SECRETION |  | 17 | 0.34 | 0.75 | 0.835 | 0.894 | 1.000 | 2944 | tags=24%, list=14%, signal=27% |
| 2156 | GO\_BICARBONATE\_TRANSPORT |  | 41 | 0.26 | 0.75 | 0.874 | 0.894 | 1.000 | 3477 | tags=17%, list=16%, signal=20% |
| 2157 | GO\_REGULATION\_OF\_EXTRINSIC\_APOPTOTIC\_SIGNALING\_PATHWAY\_VIA\_DEATH\_DOMAIN\_RECEPTORS |  | 52 | 0.26 | 0.75 | 0.888 | 0.895 | 1.000 | 4775 | tags=40%, list=22%, signal=52% |
| 2158 | GO\_MONOCARBOXYLIC\_ACID\_CATABOLIC\_PROCESS |  | 91 | 0.27 | 0.75 | 0.837 | 0.895 | 1.000 | 4880 | tags=33%, list=22%, signal=42% |
| 2159 | GO\_NEUROMUSCULAR\_PROCESS\_CONTROLLING\_POSTURE |  | 15 | 0.32 | 0.75 | 0.823 | 0.894 | 1.000 | 5167 | tags=47%, list=24%, signal=61% |
| 2160 | GO\_LIVER\_REGENERATION |  | 19 | 0.28 | 0.75 | 0.884 | 0.895 | 1.000 | 4002 | tags=32%, list=18%, signal=39% |
| 2161 | GO\_POSITIVE\_REGULATION\_OF\_DNA\_RECOMBINATION |  | 18 | 0.34 | 0.75 | 0.767 | 0.894 | 1.000 | 3084 | tags=33%, list=14%, signal=39% |
| 2162 | GO\_DNA\_DEALKYLATION |  | 19 | 0.28 | 0.75 | 0.858 | 0.895 | 1.000 | 145 | tags=5%, list=1%, signal=5% |
| 2163 | GO\_2\_OXOGLUTARATE\_METABOLIC\_PROCESS |  | 19 | 0.30 | 0.75 | 0.783 | 0.896 | 1.000 | 5185 | tags=42%, list=24%, signal=55% |
| 2164 | GO\_POSITIVE\_REGULATION\_OF\_TYPE\_I\_INTERFERON\_PRODUCTION |  | 70 | 0.27 | 0.75 | 0.757 | 0.898 | 1.000 | 1877 | tags=10%, list=9%, signal=11% |
| 2165 | GO\_SCF\_DEPENDENT\_PROTEASOMAL\_UBIQUITIN\_DEPENDENT\_PROTEIN\_CATABOLIC\_PROCESS |  | 23 | 0.27 | 0.75 | 0.885 | 0.900 | 1.000 | 4073 | tags=26%, list=19%, signal=32% |
| 2166 | GO\_RESPONSE\_TO\_ATP |  | 28 | 0.32 | 0.75 | 0.827 | 0.899 | 1.000 | 3080 | tags=25%, list=14%, signal=29% |
| 2167 | GO\_LIPOPROTEIN\_METABOLIC\_PROCESS |  | 121 | 0.21 | 0.74 | 0.937 | 0.900 | 1.000 | 4667 | tags=22%, list=21%, signal=28% |
| 2168 | GO\_HYDROGEN\_PEROXIDE\_CATABOLIC\_PROCESS |  | 19 | 0.31 | 0.74 | 0.864 | 0.903 | 1.000 | 4682 | tags=32%, list=22%, signal=40% |
| 2169 | GO\_SINGLE\_ORGANISM\_MEMBRANE\_BUDDING |  | 70 | 0.21 | 0.74 | 0.890 | 0.903 | 1.000 | 4751 | tags=26%, list=22%, signal=33% |
| 2170 | GO\_ISOPRENOID\_METABOLIC\_PROCESS |  | 114 | 0.26 | 0.74 | 0.943 | 0.904 | 1.000 | 2167 | tags=12%, list=10%, signal=14% |
| 2171 | GO\_HORMONE\_METABOLIC\_PROCESS |  | 146 | 0.26 | 0.74 | 0.958 | 0.906 | 1.000 | 1426 | tags=9%, list=7%, signal=9% |
| 2172 | GO\_POSITIVE\_REGULATION\_OF\_HEART\_CONTRACTION |  | 35 | 0.27 | 0.74 | 0.948 | 0.906 | 1.000 | 3404 | tags=23%, list=16%, signal=27% |
| 2173 | GO\_MODULATION\_BY\_HOST\_OF\_VIRAL\_PROCESS |  | 17 | 0.30 | 0.74 | 0.824 | 0.907 | 1.000 | 2753 | tags=24%, list=13%, signal=27% |
| 2174 | GO\_POSITIVE\_REGULATION\_OF\_FATTY\_ACID\_BIOSYNTHETIC\_PROCESS |  | 17 | 0.31 | 0.74 | 0.848 | 0.907 | 1.000 | 2376 | tags=18%, list=11%, signal=20% |
| 2175 | GO\_INTERMEDIATE\_FILAMENT\_BASED\_PROCESS |  | 42 | 0.28 | 0.74 | 0.893 | 0.908 | 1.000 | 1843 | tags=14%, list=8%, signal=16% |
| 2176 | GO\_CELLULAR\_RESPONSE\_TO\_LITHIUM\_ION |  | 17 | 0.30 | 0.74 | 0.857 | 0.908 | 1.000 | 4002 | tags=35%, list=18%, signal=43% |
| 2177 | GO\_ER\_TO\_GOLGI\_VESICLE\_MEDIATED\_TRANSPORT |  | 157 | 0.18 | 0.73 | 0.930 | 0.910 | 1.000 | 5197 | tags=25%, list=24%, signal=32% |
| 2178 | GO\_DEVELOPMENTAL\_INDUCTION |  | 25 | 0.32 | 0.73 | 0.932 | 0.913 | 1.000 | 1608 | tags=16%, list=7%, signal=17% |
| 2179 | GO\_CRANIAL\_NERVE\_DEVELOPMENT |  | 40 | 0.29 | 0.73 | 0.875 | 0.914 | 1.000 | 2057 | tags=13%, list=9%, signal=14% |
| 2180 | GO\_GLUTAMINE\_FAMILY\_AMINO\_ACID\_CATABOLIC\_PROCESS |  | 23 | 0.28 | 0.73 | 0.907 | 0.914 | 1.000 | 1789 | tags=17%, list=8%, signal=19% |
| 2181 | GO\_REGULATION\_OF\_NUCLEASE\_ACTIVITY |  | 21 | 0.30 | 0.73 | 0.775 | 0.915 | 1.000 | 3579 | tags=24%, list=16%, signal=28% |
| 2182 | GO\_TRICARBOXYLIC\_ACID\_METABOLIC\_PROCESS |  | 37 | 0.22 | 0.73 | 0.827 | 0.917 | 1.000 | 5185 | tags=30%, list=24%, signal=39% |
| 2183 | GO\_PHENOL\_CONTAINING\_COMPOUND\_METABOLIC\_PROCESS |  | 76 | 0.24 | 0.73 | 0.957 | 0.917 | 1.000 | 3555 | tags=14%, list=16%, signal=17% |
| 2184 | GO\_GLUCOCORTICOID\_METABOLIC\_PROCESS |  | 15 | 0.33 | 0.73 | 0.814 | 0.917 | 1.000 | 3105 | tags=20%, list=14%, signal=23% |
| 2185 | GO\_NEGATIVE\_REGULATION\_OF\_DNA\_BIOSYNTHETIC\_PROCESS |  | 31 | 0.24 | 0.73 | 0.925 | 0.917 | 1.000 | 3293 | tags=16%, list=15%, signal=19% |
| 2186 | GO\_EXCRETION |  | 41 | 0.27 | 0.73 | 0.891 | 0.917 | 1.000 | 3230 | tags=22%, list=15%, signal=26% |
| 2187 | GO\_ORGANIC\_HYDROXY\_COMPOUND\_METABOLIC\_PROCESS |  | 462 | 0.21 | 0.72 | 0.994 | 0.918 | 1.000 | 4019 | tags=18%, list=18%, signal=21% |
| 2188 | GO\_ENDOCRINE\_SYSTEM\_DEVELOPMENT |  | 121 | 0.23 | 0.72 | 0.981 | 0.923 | 1.000 | 3058 | tags=15%, list=14%, signal=17% |
| 2189 | GO\_ENTEROENDOCRINE\_CELL\_DIFFERENTIATION |  | 19 | 0.31 | 0.72 | 0.925 | 0.925 | 1.000 | 5198 | tags=42%, list=24%, signal=55% |
| 2190 | GO\_REGULATION\_OF\_MESONEPHROS\_DEVELOPMENT |  | 26 | 0.31 | 0.72 | 0.917 | 0.926 | 1.000 | 3267 | tags=27%, list=15%, signal=32% |
| 2191 | GO\_POSITIVE\_REGULATION\_OF\_RNA\_SPLICING |  | 19 | 0.25 | 0.71 | 0.838 | 0.929 | 1.000 | 4161 | tags=21%, list=19%, signal=26% |
| 2192 | GO\_SENSORY\_PERCEPTION\_OF\_TASTE |  | 52 | 0.28 | 0.71 | 0.862 | 0.929 | 1.000 | 3021 | tags=15%, list=14%, signal=18% |
| 2193 | GO\_OVULATION |  | 17 | 0.32 | 0.71 | 0.894 | 0.930 | 1.000 | 3222 | tags=35%, list=15%, signal=41% |
| 2194 | GO\_ENDOSOME\_ORGANIZATION |  | 58 | 0.20 | 0.71 | 0.869 | 0.930 | 1.000 | 4200 | tags=19%, list=19%, signal=23% |
| 2195 | GO\_AMIDE\_TRANSPORT |  | 90 | 0.22 | 0.71 | 0.982 | 0.931 | 1.000 | 4662 | tags=26%, list=21%, signal=32% |
| 2196 | GO\_AXONEME\_ASSEMBLY |  | 34 | 0.25 | 0.71 | 0.896 | 0.931 | 1.000 | 5399 | tags=26%, list=25%, signal=35% |
| 2197 | GO\_REGULATION\_OF\_TYROSINE\_PHOSPHORYLATION\_OF\_STAT1\_PROTEIN |  | 16 | 0.32 | 0.71 | 0.918 | 0.932 | 1.000 | 3084 | tags=25%, list=14%, signal=29% |
| 2198 | GO\_ARGININE\_METABOLIC\_PROCESS |  | 17 | 0.31 | 0.71 | 0.920 | 0.932 | 1.000 | 1711 | tags=24%, list=8%, signal=26% |
| 2199 | GO\_CRANIAL\_NERVE\_MORPHOGENESIS |  | 22 | 0.31 | 0.71 | 0.870 | 0.933 | 1.000 | 2898 | tags=18%, list=13%, signal=21% |
| 2200 | GO\_REGULATION\_OF\_INTERLEUKIN\_2\_BIOSYNTHETIC\_PROCESS |  | 17 | 0.36 | 0.70 | 0.850 | 0.936 | 1.000 | 3474 | tags=47%, list=16%, signal=56% |
| 2201 | GO\_RESPONSE\_TO\_PROTOZOAN |  | 19 | 0.34 | 0.70 | 0.863 | 0.939 | 1.000 | 3775 | tags=37%, list=17%, signal=45% |
| 2202 | GO\_VESICLE\_MEDIATED\_TRANSPORT\_BETWEEN\_ENDOSOMAL\_COMPARTMENTS |  | 19 | 0.25 | 0.70 | 0.857 | 0.939 | 1.000 | 4442 | tags=32%, list=20%, signal=40% |
| 2203 | GO\_REGULATION\_OF\_TYPE\_I\_INTERFERON\_PRODUCTION |  | 107 | 0.25 | 0.70 | 0.805 | 0.939 | 1.000 | 4362 | tags=22%, list=20%, signal=28% |
| 2204 | GO\_CATECHOLAMINE\_METABOLIC\_PROCESS |  | 39 | 0.24 | 0.70 | 0.965 | 0.940 | 1.000 | 3987 | tags=21%, list=18%, signal=25% |
| 2205 | GO\_REGULATION\_OF\_ASTROCYTE\_DIFFERENTIATION |  | 27 | 0.27 | 0.70 | 0.938 | 0.940 | 1.000 | 2290 | tags=19%, list=11%, signal=21% |
| 2206 | GO\_EPOXYGENASE\_P450\_PATHWAY |  | 18 | 0.38 | 0.70 | 0.853 | 0.940 | 1.000 | 344 | tags=6%, list=2%, signal=6% |
| 2207 | GO\_IRE1\_MEDIATED\_UNFOLDED\_PROTEIN\_RESPONSE |  | 51 | 0.22 | 0.69 | 0.836 | 0.945 | 1.000 | 4946 | tags=31%, list=23%, signal=41% |
| 2208 | GO\_NEGATIVE\_REGULATION\_OF\_INTERLEUKIN\_10\_PRODUCTION |  | 16 | 0.36 | 0.69 | 0.835 | 0.946 | 1.000 | 2183 | tags=19%, list=10%, signal=21% |
| 2209 | GO\_RESPONSE\_TO\_AUDITORY\_STIMULUS |  | 22 | 0.28 | 0.69 | 0.955 | 0.946 | 1.000 | 3652 | tags=27%, list=17%, signal=33% |
| 2210 | GO\_INTRACELLULAR\_ESTROGEN\_RECEPTOR\_SIGNALING\_PATHWAY |  | 18 | 0.24 | 0.69 | 0.848 | 0.946 | 1.000 | 796 | tags=6%, list=4%, signal=6% |
| 2211 | GO\_GLYOXYLATE\_METABOLIC\_PROCESS |  | 26 | 0.26 | 0.69 | 0.828 | 0.946 | 1.000 | 440 | tags=4%, list=2%, signal=4% |
| 2212 | GO\_PH\_REDUCTION |  | 37 | 0.23 | 0.69 | 0.931 | 0.946 | 1.000 | 4622 | tags=27%, list=21%, signal=34% |
| 2213 | GO\_POSITIVE\_REGULATION\_OF\_MICROTUBULE\_POLYMERIZATION |  | 18 | 0.25 | 0.69 | 0.926 | 0.947 | 1.000 | 3067 | tags=22%, list=14%, signal=26% |
| 2214 | GO\_GLUTATHIONE\_DERIVATIVE\_METABOLIC\_PROCESS |  | 20 | 0.36 | 0.69 | 0.859 | 0.947 | 1.000 | 2410 | tags=20%, list=11%, signal=22% |
| 2215 | GO\_NERVE\_DEVELOPMENT |  | 63 | 0.25 | 0.68 | 0.957 | 0.947 | 1.000 | 2898 | tags=16%, list=13%, signal=18% |
| 2216 | GO\_FATTY\_ACID\_BETA\_OXIDATION |  | 49 | 0.24 | 0.68 | 0.925 | 0.947 | 1.000 | 4880 | tags=33%, list=22%, signal=42% |
| 2217 | GO\_CALCIUM\_DEPENDENT\_CELL\_CELL\_ADHESION\_VIA\_PLASMA\_MEMBRANE\_CELL\_ADHESION\_MOLECULES |  | 23 | 0.30 | 0.68 | 0.936 | 0.948 | 1.000 | 3906 | tags=22%, list=18%, signal=26% |
| 2218 | GO\_DICARBOXYLIC\_ACID\_CATABOLIC\_PROCESS |  | 15 | 0.29 | 0.68 | 0.919 | 0.947 | 1.000 | 4696 | tags=33%, list=22%, signal=42% |
| 2219 | GO\_ANTIGEN\_PROCESSING\_AND\_PRESENTATION\_OF\_EXOGENOUS\_PEPTIDE\_ANTIGEN\_VIA\_MHC\_CLASS\_I |  | 62 | 0.25 | 0.68 | 0.791 | 0.948 | 1.000 | 1828 | tags=10%, list=8%, signal=11% |
| 2220 | GO\_CELLULAR\_RESPONSE\_TO\_TOPOLOGICALLY\_INCORRECT\_PROTEIN |  | 114 | 0.19 | 0.68 | 0.951 | 0.948 | 1.000 | 5005 | tags=29%, list=23%, signal=37% |
| 2221 | GO\_RESPONSE\_TO\_XENOBIOTIC\_STIMULUS |  | 90 | 0.29 | 0.68 | 0.913 | 0.951 | 1.000 | 3555 | tags=19%, list=16%, signal=22% |
| 2222 | GO\_PROTEIN\_LOCALIZATION\_TO\_SYNAPSE |  | 15 | 0.26 | 0.68 | 0.908 | 0.950 | 1.000 | 3157 | tags=20%, list=15%, signal=23% |
| 2223 | GO\_POSITIVE\_REGULATION\_OF\_MESONEPHROS\_DEVELOPMENT |  | 22 | 0.31 | 0.68 | 0.974 | 0.951 | 1.000 | 3267 | tags=27%, list=15%, signal=32% |
| 2224 | GO\_RETROGRADE\_TRANSPORT\_VESICLE\_RECYCLING\_WITHIN\_GOLGI |  | 19 | 0.25 | 0.67 | 0.889 | 0.956 | 1.000 | 4121 | tags=26%, list=19%, signal=32% |
| 2225 | GO\_CLATHRIN\_MEDIATED\_ENDOCYTOSIS |  | 35 | 0.23 | 0.67 | 0.966 | 0.957 | 1.000 | 3798 | tags=20%, list=17%, signal=24% |
| 2226 | GO\_OSTEOBLAST\_DEVELOPMENT |  | 17 | 0.30 | 0.67 | 0.934 | 0.957 | 1.000 | 3001 | tags=24%, list=14%, signal=27% |
| 2227 | GO\_REGULATION\_OF\_HYDROGEN\_PEROXIDE\_METABOLIC\_PROCESS |  | 15 | 0.26 | 0.66 | 0.959 | 0.959 | 1.000 | 4152 | tags=33%, list=19%, signal=41% |
| 2228 | GO\_REGULATION\_OF\_T\_CELL\_MEDIATED\_CYTOTOXICITY |  | 22 | 0.29 | 0.66 | 0.947 | 0.960 | 1.000 | 4560 | tags=36%, list=21%, signal=46% |
| 2229 | GO\_HIGH\_DENSITY\_LIPOPROTEIN\_PARTICLE\_REMODELING |  | 15 | 0.30 | 0.66 | 0.906 | 0.960 | 1.000 | 2363 | tags=20%, list=11%, signal=22% |
| 2230 | GO\_PROTEIN\_LOCALIZATION\_TO\_LYSOSOME |  | 16 | 0.26 | 0.66 | 0.903 | 0.960 | 1.000 | 4462 | tags=31%, list=21%, signal=39% |
| 2231 | GO\_PEPTIDE\_SECRETION |  | 56 | 0.22 | 0.66 | 0.964 | 0.960 | 1.000 | 4365 | tags=25%, list=20%, signal=31% |
| 2232 | GO\_RESPONSE\_TO\_MAGNESIUM\_ION |  | 22 | 0.28 | 0.66 | 0.960 | 0.961 | 1.000 | 900 | tags=9%, list=4%, signal=9% |
| 2233 | GO\_REGULATION\_OF\_FIBROBLAST\_MIGRATION |  | 27 | 0.25 | 0.65 | 0.959 | 0.968 | 1.000 | 3032 | tags=22%, list=14%, signal=26% |
| 2234 | GO\_SKIN\_DEVELOPMENT |  | 186 | 0.26 | 0.65 | 0.972 | 0.968 | 1.000 | 3667 | tags=25%, list=17%, signal=29% |
| 2235 | GO\_DENTATE\_GYRUS\_DEVELOPMENT |  | 15 | 0.29 | 0.64 | 0.972 | 0.968 | 1.000 | 4686 | tags=40%, list=22%, signal=51% |
| 2236 | GO\_PEPTIDE\_CATABOLIC\_PROCESS |  | 22 | 0.26 | 0.64 | 0.955 | 0.968 | 1.000 | 4843 | tags=41%, list=22%, signal=53% |
| 2237 | GO\_CATECHOLAMINE\_BIOSYNTHETIC\_PROCESS |  | 17 | 0.26 | 0.64 | 0.981 | 0.968 | 1.000 | 885 | tags=6%, list=4%, signal=6% |
| 2238 | GO\_REGULATION\_OF\_RUFFLE\_ASSEMBLY |  | 19 | 0.25 | 0.64 | 0.978 | 0.968 | 1.000 | 3351 | tags=21%, list=15%, signal=25% |
| 2239 | GO\_O\_GLYCAN\_PROCESSING |  | 55 | 0.27 | 0.64 | 0.956 | 0.969 | 1.000 | 3937 | tags=27%, list=18%, signal=33% |
| 2240 | GO\_REGULATION\_OF\_INTERLEUKIN\_6\_BIOSYNTHETIC\_PROCESS |  | 15 | 0.31 | 0.64 | 0.960 | 0.970 | 1.000 | 3736 | tags=27%, list=17%, signal=32% |
| 2241 | GO\_RETINOL\_METABOLIC\_PROCESS |  | 25 | 0.30 | 0.64 | 0.944 | 0.969 | 1.000 | 2433 | tags=12%, list=11%, signal=13% |
| 2242 | GO\_DIGESTION |  | 117 | 0.23 | 0.63 | 0.987 | 0.971 | 1.000 | 2305 | tags=9%, list=11%, signal=10% |
| 2243 | GO\_DIENCEPHALON\_DEVELOPMENT |  | 76 | 0.21 | 0.63 | 0.980 | 0.971 | 1.000 | 2956 | tags=14%, list=14%, signal=17% |
| 2244 | GO\_ENDOCRINE\_PANCREAS\_DEVELOPMENT |  | 39 | 0.23 | 0.63 | 0.996 | 0.972 | 1.000 | 5198 | tags=31%, list=24%, signal=40% |
| 2245 | GO\_GLYCOPROTEIN\_CATABOLIC\_PROCESS |  | 15 | 0.24 | 0.63 | 0.975 | 0.974 | 1.000 | 4576 | tags=33%, list=21%, signal=42% |
| 2246 | GO\_REGULATION\_OF\_PROTEIN\_SUMOYLATION |  | 19 | 0.25 | 0.63 | 0.981 | 0.973 | 1.000 | 4573 | tags=26%, list=21%, signal=33% |
| 2247 | GO\_METANEPHROS\_MORPHOGENESIS |  | 27 | 0.26 | 0.62 | 0.996 | 0.974 | 1.000 | 3267 | tags=22%, list=15%, signal=26% |
| 2248 | GO\_INNERVATION |  | 22 | 0.26 | 0.62 | 0.988 | 0.974 | 1.000 | 3543 | tags=27%, list=16%, signal=33% |
| 2249 | GO\_NEURAL\_TUBE\_PATTERNING |  | 31 | 0.24 | 0.62 | 0.958 | 0.975 | 1.000 | 3614 | tags=19%, list=17%, signal=23% |
| 2250 | GO\_ERAD\_PATHWAY |  | 71 | 0.17 | 0.62 | 0.974 | 0.975 | 1.000 | 4576 | tags=21%, list=21%, signal=27% |
| 2251 | GO\_BRANCH\_ELONGATION\_OF\_AN\_EPITHELIUM |  | 17 | 0.27 | 0.62 | 0.983 | 0.975 | 1.000 | 2956 | tags=18%, list=14%, signal=20% |
| 2252 | GO\_LIPOPOLYSACCHARIDE\_MEDIATED\_SIGNALING\_PATHWAY |  | 30 | 0.28 | 0.61 | 0.923 | 0.976 | 1.000 | 4448 | tags=37%, list=20%, signal=46% |
| 2253 | GO\_POSITIVE\_REGULATION\_OF\_TRIGLYCERIDE\_METABOLIC\_PROCESS |  | 20 | 0.25 | 0.61 | 0.965 | 0.977 | 1.000 | 4650 | tags=25%, list=21%, signal=32% |
| 2254 | GO\_REGULATION\_OF\_DELAYED\_RECTIFIER\_POTASSIUM\_CHANNEL\_ACTIVITY |  | 18 | 0.22 | 0.61 | 0.978 | 0.978 | 1.000 | 5135 | tags=33%, list=24%, signal=44% |
| 2255 | GO\_EPITHELIAL\_CILIUM\_MOVEMENT |  | 16 | 0.26 | 0.61 | 0.974 | 0.979 | 1.000 | 3433 | tags=25%, list=16%, signal=30% |
| 2256 | GO\_DRUG\_METABOLIC\_PROCESS |  | 33 | 0.31 | 0.60 | 0.967 | 0.979 | 1.000 | 2729 | tags=15%, list=13%, signal=17% |
| 2257 | GO\_MYD88\_INDEPENDENT\_TOLL\_LIKE\_RECEPTOR\_SIGNALING\_PATHWAY |  | 29 | 0.25 | 0.60 | 0.940 | 0.981 | 1.000 | 1602 | tags=7%, list=7%, signal=7% |
| 2258 | GO\_DOPAMINE\_METABOLIC\_PROCESS |  | 25 | 0.21 | 0.59 | 0.986 | 0.984 | 1.000 | 5642 | tags=32%, list=26%, signal=43% |
| 2259 | GO\_CALCIUM\_INDEPENDENT\_CELL\_CELL\_ADHESION\_VIA\_PLASMA\_MEMBRANE\_CELL\_ADHESION\_MOLECULES |  | 20 | 0.27 | 0.58 | 0.987 | 0.985 | 1.000 | 3266 | tags=25%, list=15%, signal=29% |
| 2260 | GO\_VERY\_LONG\_CHAIN\_FATTY\_ACID\_METABOLIC\_PROCESS |  | 23 | 0.26 | 0.57 | 0.994 | 0.987 | 1.000 | 5233 | tags=43%, list=24%, signal=57% |
| 2261 | GO\_DEFENSE\_RESPONSE\_TO\_VIRUS |  | 150 | 0.24 | 0.56 | 0.930 | 0.989 | 1.000 | 3084 | tags=17%, list=14%, signal=19% |
| 2262 | GO\_CILIUM\_MORPHOGENESIS |  | 180 | 0.16 | 0.55 | 1.000 | 0.991 | 1.000 | 3676 | tags=16%, list=17%, signal=19% |
| 2263 | GO\_DETECTION\_OF\_CHEMICAL\_STIMULUS\_INVOLVED\_IN\_SENSORY\_PERCEPTION\_OF\_TASTE |  | 34 | 0.24 | 0.55 | 0.954 | 0.992 | 1.000 | 4320 | tags=18%, list=20%, signal=22% |
| 2264 | GO\_PHAGOSOME\_ACIDIFICATION |  | 25 | 0.21 | 0.54 | 0.989 | 0.991 | 1.000 | 5040 | tags=28%, list=23%, signal=36% |
| 2265 | GO\_KERATINIZATION |  | 31 | 0.32 | 0.52 | 0.964 | 0.994 | 1.000 | 2453 | tags=19%, list=11%, signal=22% |
| 2266 | GO\_DORSAL\_VENTRAL\_NEURAL\_TUBE\_PATTERNING |  | 15 | 0.24 | 0.52 | 0.989 | 0.994 | 1.000 | 3043 | tags=20%, list=14%, signal=23% |
| 2267 | GO\_NEGATIVE\_REGULATION\_OF\_TYPE\_I\_INTERFERON\_PRODUCTION |  | 37 | 0.21 | 0.49 | 0.983 | 0.996 | 1.000 | 4361 | tags=24%, list=20%, signal=30% |
| 2268 | GO\_SENSORY\_PERCEPTION\_OF\_CHEMICAL\_STIMULUS |  | 165 | 0.12 | 0.47 | 1.000 | 0.997 | 1.000 | 3824 | tags=9%, list=18%, signal=11% |
Table: Gene sets enriched in phenotype **H (36 samples)**[plain text format]****

  
